# Can Omega-3 prevent the accidence of stroke: a mendelian randomization study

**DOI:** 10.1186/s41065-024-00329-9

**Published:** 2024-09-05

**Authors:** Chongcheng Xi, Jie Zhang, Haihui Liu, Sian Tao, Ying Xie, Jibin Liu, Changqing Tong, Dong Tian, Hua Ye, Xiaobo Zhang

**Affiliations:** 1 School of Basic Medical Sciences, Chengdu University of Traditional Chinese Medicine, Chengdu, China; 2 School of Nursing, Chengdu University of Traditional Chinese Medicine, Chengdu, China; 3 School of Acupuncture- Moxibustion and Tuina, Beijing University of Traditional Chinese Medicine, Beijing, China; 4 School of Traditional Chinese Medicine, Beijing University of Traditional Chinese Medicine, Beijing, China; 5 School of Intelligent Medicine, Chengdu University of Traditional Chinese Medicine, Chengdu, China

**Keywords:** Fatty acids, Blood pressure, Ischemic stroke, Omega-3, Mendelian randomization

## Abstract

**Background:**

The lipid-lowering effects of Omega-3 fatty acids have been widely reported, yet their impact on ischemic stroke remains controversial. Reports on the protective effects of unsaturated fatty acids, such as Omega-6 and Omega-7, as well as saturated fatty acids in cardiovascular diseases, including hypertension and ischemic stroke, are less frequent.

**Objectives:**

This study aims to identify fatty acids associated with blood pressure and ischemic stroke through Mendelian randomization. Besides, it seeks to determine whether specific fatty acids can prevent ischemic stroke by managing blood pressure and revealing the specific mechanisms of this action.

**Methods:**

This research involved downloading relevant data from websites and extracting SNPs that met the standard criteria as instrumental variables. Simultaneously, the ‘MR-PRESSO’ package and ‘Mendelian Randomization’ package were used to eliminate confounding SNPs that could bias the study results. Then, inverse variance weighting and the weighted median were employed as primary analysis methods, accompanied by sensitivity analysis to assess the validity of the causal relationships. Initially, multivariable Mendelian randomization was used to identify fatty acids linked to blood pressure and the incidence of ischemic stroke. The causal link between certain fatty acids and the initiation of ischemic stroke was then investigated using bidirectional and mediator Mendelian randomization techniques. Stepwise Regression and the Product of Coefficients Method in mediator Mendelian randomization were utilized to ascertain whether specific fatty acids reduce ischemic stroke risk by lowering blood pressure.

**Results:**

Multivariable Mendelian randomization analysis indicated a potential inverse correlation between Omega-3 intake and both blood pressure and ischemic stroke. Consequently, Omega-3 was selected as the exposure, with blood pressure and ischemic stroke-related data as outcomes, for further bidirectional and mediation Mendelian Randomization analyses. Bidirectional Mendelian Randomization revealed that Omega-3 significantly influences DBP (*P* = 1.01e-04) and IS (*P* = 0.016). It also showed that DBP and SBP significantly affect LAS, SVS, CES, IS, and LS. Mediator Mendelian Randomization identified five established mediating pathways: Omega-3-Diastolic blood pressure-Small vessel stroke, Omega-3-Diastolic blood pressure-Cardioembolic stroke, Omega-3-Diastolic blood pressure-Lacunar stroke, Omega-3-Diastolic blood pressure-Large artery atherosclerosis stroke, and Omega-3-Diastolic blood pressure-Ischemic stroke. Of these, four pathways are complete mediation, and one pathway is partial mediation.

**Conclusions:**

The findings suggest that Omega-3 may indirectly reduce the incidence of ischemic stroke by lowering blood pressure. Thus, blood pressure modulation might be one of the mechanisms through which Omega-3 prevents ischemic stroke. In summary, incorporating an increased intake of Omega-3 in the diet can serve as one of the dietary intervention strategies for patients with hypertension. Additionally, it can act as an adjunctive therapy for the prevention of ischemic strokes and their complications.

**Supplementary Information:**

The online version contains supplementary material available at 10.1186/s41065-024-00329-9.

A report by the World Health Organization (WHO) stated that cardiovascular diseases (CVDS) comprise various heart and vascular disorders. These encompass various disorders, not limited to coronary artery disease (CAD), cerebrovascular disease (CBD), peripheral arterial disease (PAD), rheumatic heart disease (RHD), congenital heart disease (CHD), deep vein thrombosis (DVT), and pulmonary embolism (PE) [1]. Cerebral stroke, a prevalent cerebrovascular accident, occurs when brain tissue is damaged due to sudden rupture or blockage of cerebral vessels, leading to compromised blood flow to the brain. Globally, stroke remains the second leading cause of death, accounting for 11.6% of total fatalities. Furthermore, it is the third largest contributor to disability-adjusted life years (DALYs), representing 5.7% of the total global disease burden [2]. Based on the WHO report, as of 2019, there were more than 101 million people globally living with the effects of stroke, and approximately 15 million new cases are reported annually [3].

Hypertension is identified by the WHO [4] and numerous scholars [5, 6] as a major precipitant of stroke [7], a finding corroborated in clinical practice [8]. In patients with poorly managed hypertension, the risk of stroke escalates [9] due to mechanisms such as the elevation of the baseline triglyceride-glucose index [10], which contributes to atherosclerosis and lipid-amine deposition [11, 12]. Consequently, the WHO recommends optimal blood pressure control as an effective strategy to reduce stroke risk across various ages, genders, and races [13]. However, achieving ideal blood pressure is not solely reliant on medication, as evidenced by stroke cases linked to poorly managed blood pressure [14, 15]. Thus, the identification of dietary supplements that can effectively regulate blood pressure is vital. These interventions not only aid in lowering hypertension incidence but also in preventing related complications, including ischemic stroke. Stroke is primarily categorized into hemorrhagic and ischemic types, with ischemic stroke constituting three-quarters of all cases [16]. While studies on the causal link between blood pressure and ischemic stroke incidence are less frequent, more research has focused on the relationship between blood pressure and functional outcomes in ischemic stroke patients [17]. Hence, this study zeroes in on ischemic stroke to investigate the influence of fatty acids and blood pressure on its occurrence.

Fatty acids are classified based on hydrogen atom counts in their carbon chains and carbon atom bond characteristics. These include total fatty acids (TFAs), saturated fatty acids (SFAs), polyunsaturated fatty acids (PUFAs), and monounsaturated fatty acids (MUFAs) [18]. PUFAs, containing two or more carbon-carbon double bonds [19], are further categorized based on the last double bond’s position in the carbon chain, leading to Omega-3, Omega-6, Omega-7, Omega-9, etc. Research shows PUFAs, through anti-inflammatory and anti-atherosclerosis actions [20] significantly reduce CVDS incidence [21, 22], including myocardial infarction [23], sudden cardiac death [24; 25], and stroke. Mendelian randomization(MR) studies demonstrate the protective effects of Omega-3 and Omega-6 on CVDS risk [26]. PUFAs effectively lower hypertension incidence [27], with specific fatty acids like linoleic acid [28] potentially preventing ischemic stroke via blood pressure reduction. Combining Omega-3 rich diets with antihypertensive drugs can control blood pressure [29] and reduce CVDS risk [30; 31]. However, some studies question the long-term link between Omega-3 intake and CVDS, including hypertension [31–35]. Less is known about the causal relationship between fatty acids like Omega-7, Omega-9, saturated fatty acids, and CVDS. A study found Omega-7 ineffective in reducing inflammatory biomarkers [36]. Omega-9 might reduce hypertension and ischemic stroke incidence through its anti-inflammatory effect [37], but data supporting the impact of Omega-7, Omega-9, and other fatty acids on blood pressure and heart disease are lacking.

In conclusion, the role of fatty acids in reducing ischemic stroke risk [38–41] and their protective effects against CVDs [42; 43] remain contentious, with no definitive consensus reached. MR studies, which use single nucleotide polymorphisms (SNPs) closely associated with the exposure of interest as instrumental variables [44], are instrumental in establishing causal relationships and examining the links between exposures and outcomes [45]. These studies effectively overcome the limitations of cost and confounding factors inherent in clinical observational studies. MR provides robust evidence to either confirm or refute causal hypotheses linking environmental exposures to diseases [46]. Therefore, some studies have employed MR to investigate the association between omega-3 and other PUFAs with brain diseases such as epilepsy [47], hydrocephalus [48], Alzheimer’s disease [49], Parkinson’s disease [50] and so on. These studies provide methodological examples and feasibility assurance for the conduct of this research. Hence, this study employed MR to explore the influence of fatty acids on ischemic stroke and test the hypothesis that fatty acids can prevent ischemic stroke by regulating blood pressure. This offers theoretical support and guidance for ischemic stroke prevention in clinical practice, particularly in managing ischemic stroke sequelae in patients with abnormal blood pressure.

## Materials and methods

### Data source

This research utilized publicly accessible GWAS data to acquire relevant exposure, mediator, and outcome datasets. Given previous findings that the effects of Omega-3 fatty acids on cardiovascular disease may vary among different populations [51], this study specifically targeted individuals of European descent to ensure a more focused population sample, aiming to reduce potential biases in the MR study results. The IEU Open GWAS Project provided the GWAS data for Omega-3, Omega-6, Omega-7, Omega-9, and SFAs, as well as the ratios of Omega-3 to total fatty acids and Omega-6 to Omega-3 fatty acids. Additionally, The GWAS datasets for systolic blood pressure (SBP) and diastolic blood pressure (DBP), derived from a meta-analysis of 1.3 million individuals, were utilized as measures for blood pressure evaluation, with hypertensive patients serving as the observation group [52], were obtained as measures for blood pressure evaluation. This study also incorporated overall GWAS data for ischemic stroke (IS) and included four common ischemic stroke subtypes – large artery atherosclerosis stroke (LAS), cardioembolic stroke (CES), small vessel stroke (SVS), and lacunar stroke (LS) – in the analysis, acquiring the relevant GWAS data from the IEU Open GWAS Project. Details of each dataset are presented in Table 1.

**Table 1:** Overview of GWAs data used in MR

	Phenotype	Sample size	Ancestry	Year of publication	Category
Fatty acid	Omega-3	115,006	European	2022	Continuous
	Omega-6	115,006	European	2022	Continuous
	Omega-7, 9, SFAs	13,506	European	2016	Continuous
Omega-3 level to other acids	Omega-3 rate	115,006	European	2022	Continuous
	Omega-3/ Omega-6	115,006	European	2022	Continuous
Blood pressure	DBP	340,162	European	2021	Continuous
	SBP	340,159	European	2021	Continuous
Ischemic Stroke	IS	440,328	European	2018	Binary
	LS	232,596	European	2021	Binary
	LAS	150,765	European	2018	Binary
	SVS	198,048	European	2018	Binary
	CES	211,763	European	2018	Binary

### Overall study design

**Fig. 1:**
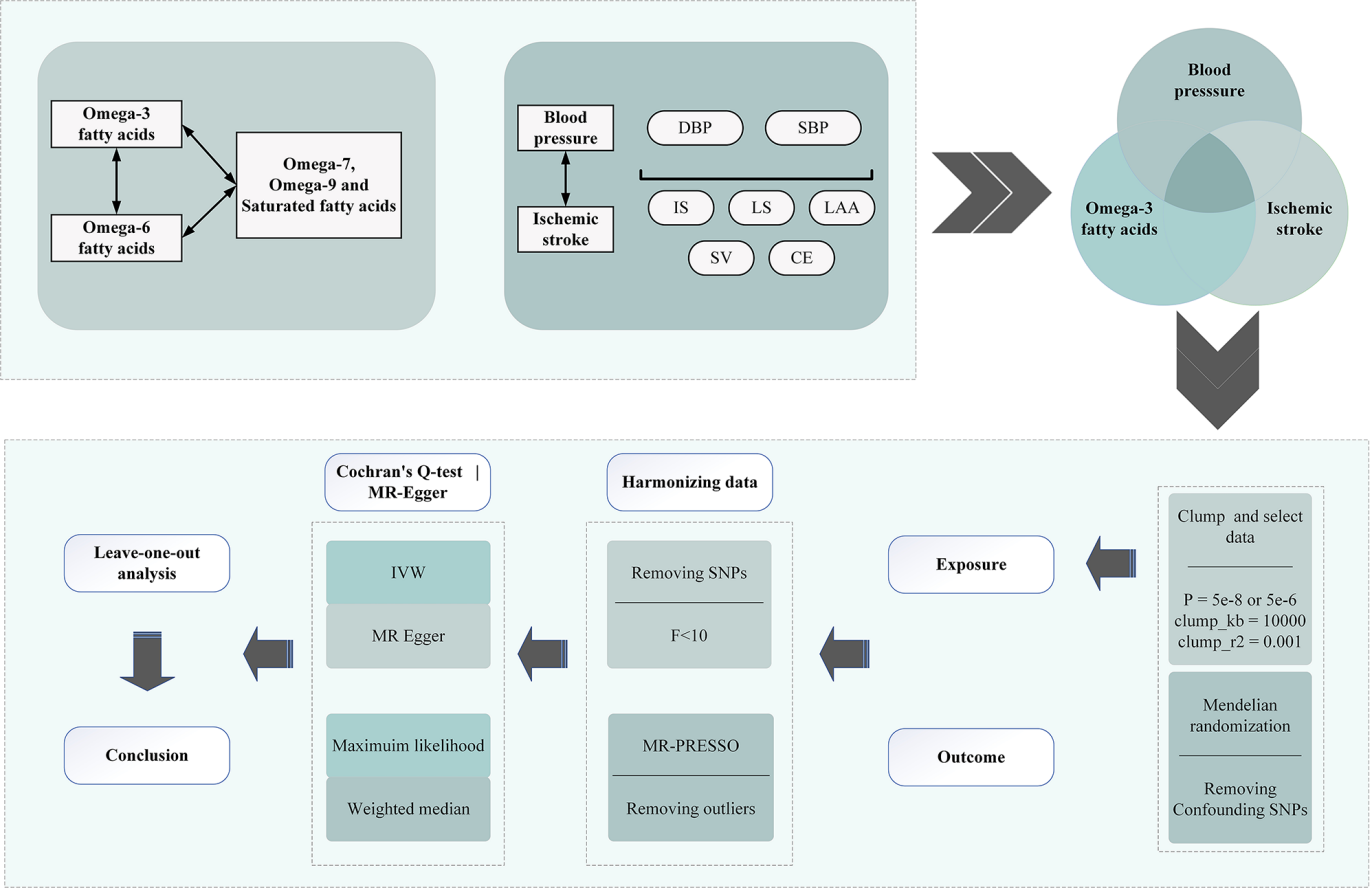
Overview of the present study design

As depicted in Fig. 1, this study employed a combination of multivariable Mendelian randomization (MVMR), mediation MR analysis, and bidirectional MR analysis to investigate the causal relationship between fatty acids and ischemic stroke. Initially, three datasets representing Omega-3, Omega-6, (Omega-7, Omega-9, and SFAs) fatty acids were included as exposures, with blood pressure and ischemic stroke data serving as outcomes. MVMR accounts for the relationships between multiple genetic variants and multiple exposures, offering improved control over confounding factors [53], thereby enabling more precise estimation of the “direct” causal effects [54] of each exposure (fatty acids) on the mediator (blood pressure) and the outcome (ischemic stroke).

Following the MVMR results, the study advanced to mediation MR analysis, employing a stepwise testing approach and the product of coefficients method. This analysis incorporated one dataset for Omega-3 fatty acids, two for blood pressure, and five for ischemic stroke, aiming to determine if blood pressure mediates the relationship between Omega-3 fatty acids and ischemic stroke incidence. Mediation MR typically involves three steps. Firstly, SNPs predict the genetic risk score for Omega-3 fatty acids, estimating its causal impact on blood pressure and ischemic stroke. Secondly, SNPs associated with blood pressure are used to estimate its causal effect on ischemic stroke. Thirdly, Omega-3 fatty acids’ total effect on ischemic stroke is dissected into direct (independent of blood pressure) and indirect (mediated via blood pressure) effects. The mediation coefficient and corresponding p-value were calculated using the product of coefficients method. Concurrently, reverse MR analysis was conducted in the mediation MR phase to explore potential reverse causality. Finally, As A Supplement, ratios of Omega-3 to total fatty acids and Omega-6 to Omega-3 fatty acids were utilized as indicators of the Omega-3 rate relative to other acids, to further investigate the causal link between changes in the Omega-3 ratio and variations in blood pressure and ischemic stroke risk.

### MR methods

#### Instrument selection

Instrument selection for this study involved procuring the latest GWAS data and carefully selecting genetic association data based on criteria such as sample size, number of SNPs, publication year, and ancestry (as detailed in Table 1). Adhering to the three foundational assumptions of MR [55], the study first ensured that the instrumental variables, specifically SNPs, had a strong correlation with the exposure factors. Relevant phenotypes of selected SNPs, all independent (r2 < 0.001) and strongly associated (*P* < 5E-8), were sourced from the GWAS database. Due to the limited number of SNPs meeting the *P* < 5E-8 criterion for fatty acids, the threshold was relaxed to *P* < 5E-6 [56] for these exposures. The effectiveness of these instrumental variables was assessed with the F-statistic(F = R2×(n − k−1)/(1 − R2)×K), selecting only those with F > 10 [57] to exclude weak instruments from influencing MR results. Detailed SNP information is provided in Supplementary material 1.

The second MR assumption ensures that selected SNPs are not associated with confounding factors. The SNPs in this study, adhering to the law of independent assortment, were strongly correlated with the exposure while being unrelated to potential confounders. The third assumption mandates that instrumental variables influence the outcome exclusively through the exposure. To satisfy these latter two assumptions, the study used the ‘Mendelian randomization’ package to eliminate SNPs potentially affecting outcomes through other pathways and restricted the study population to Europeans to minimize bias. Besides, we use the ‘MR-PRESSO’ package, setting the NbDistribution value to 1000, to identify SNPs that are outliers. These outlier SNPs are then removed before conducting the MR analysis. By identifying SNPs using the ‘Mendelian randomization’ and ‘MR-PRESSO’ packages, we can effectively eliminate confounding factors.

Additionally, the study employed four different methods — Maximum likelihood, inverse variance weighted (IVW), MR-Egger, and Weighted median — to detect and adjust for any direct associations between the instrumental variables and the outcomes. Sensitivity analyses were performed to evaluate heterogeneity and pleiotropy as well, further ensuring the robustness of the study’s findings against potential deviations from the MR assumptions.

#### Random-effect method

This study employed four MR approaches to explore causal connections. The inverse variance weighted (IVW) method was selected as the primary technique in situations where there was no heterogeneity or pleiotropy among the instrumental variables (IVs), as it offers consistent and high statistical power [58]. This method provides an accurate estimation of the causal effect, minimizing the impact of confounding factors and measurement errors. In cases of heterogeneity without pleiotropy, the Weighted median method was predominantly used [59], with the other three methods serving as supplementary approaches to estimate the MR effect. When pleiotropy was present, the MR-Egger method’s results were given priority [60]. Employing multiple methods allowed the study to assess the robustness and reliability of causal estimates under varying assumptions and potential biases.

#### Sensitivity analysis

Sensitivity analysis played a crucial role in assessing the robustness of the MR analysis, with a focus on heterogeneity and pleiotropy. Heterogeneity was evaluated using Cochran’s Q_pval statistic [61]. Q_pval value exceeding 0.05 indicates negligible heterogeneity among the included studies. To evaluate pleiotropy, the study analyzed the MR-Egger’s intercept, with the p-value of the intercept indicating the presence of pleiotropy. A p-value below 0.05 for the intercept rejects the null hypothesis, suggesting notable pleiotropy. This indicates that the chosen instrumental variables might affect not only the specific exposure under study but also other exposures, potentially introducing bias. The results of the sensitivity analysis and the specific MR methods utilized are thoroughly detailed in Supplementary material 2–7.

#### Visualizations

In this study, scatter plots, forest plots, and funnel plots were used to visualize and evaluate the efficacy and robustness of the MR analysis. Scatter plots were used to depict the effect sizes determined by each MR method, representing them as dots. This graphical approach allows for a straightforward visualization of the strength and direction of the associations between genetic variations, exposures, and outcomes, offering a visual insight into the relationships among these variables.

Forest plots were utilized to estimate the causal effects derived from multiple genetic variations. These plots visually represent the estimated effects of each genetic variant, facilitating a comparison of the consistency and directionality of the causal effects across different variants. Forest plots are instrumental in determining whether the causal effects uniformly point in a similar direction, and they provide a comprehensive view of the overall causal effect.

Funnel plots were employed to evaluate potential biases within the study, aiming to assess the precision and reliability of the results. These plots position the genetic variations around the overall effect estimate, creating a symmetrical distribution when there is no bias. The symmetry of the funnel plot is a crucial factor in detecting potential biases in the study.

All these plots, including scatter, forest, and funnel plots, are detailed in Supplementary material 8, providing a comprehensive visual representation and assessment of the MR study’s findings.

### Statistical analysis

In this study, MR analysis was carried out using the R programming language. Specifically, R packages such as TwoSampleMR and ggplot were employed for the MR analysis. The selection of MR methods was based on the results obtained from the sensitivity analysis.

For determining the statistical significance in estimating causal relationships, a p-value threshold of 0.05 was adopted. This threshold is a commonly used criterion in statistical analyses to denote significance, implying that results with a p-value below 0.05 are considered statistically significant and less likely to be due to chance.

Both pleiotropy and heterogeneity tests in this study also adhered to the p-value threshold of 0.05 for statistical significance. This consistency in the threshold across different tests ensures a standardized approach to evaluating the robustness and reliability of the study’s findings. The use of such thresholds is crucial in MR analysis for determining the validity of the causal inferences drawn from the genetic data.

## Result

During the MVMR study, this research explored the causal relationships among Omega-3 fatty acids, Omega-6 fatty acids, Omega-7 fatty acids, Omega-9 fatty acids, and saturated fats in relation to blood pressure and ischemic stroke. Redundancy in overlapping samples was minimized using the “mv_extract_exposures” function. Notably, all chosen SNPs exhibited an F-value greater than 10, affirming their robustness as instrumental variables. For comprehensive details, Supplementary material 1 is recommended, which offers further insights and specifics about the selected SNPs and their attributes.

### Sensitivity analysis result

To evaluate the stability of the MR analysis, this study included a sensitivity analysis addressing heterogeneity and pleiotropy. Concurrently, the leave-one-out method was employed to ascertain the impact of each SNP on the results, and funnel plots were utilized to investigate potential pleiotropy. For comprehensive information regarding the sensitivity analysis outcomes and the chosen final MR model, please refer to Supplementary material 2–7.

### MR analysis

#### Effect of fatty acids on mediators and outcomes

**Fig. 2:**
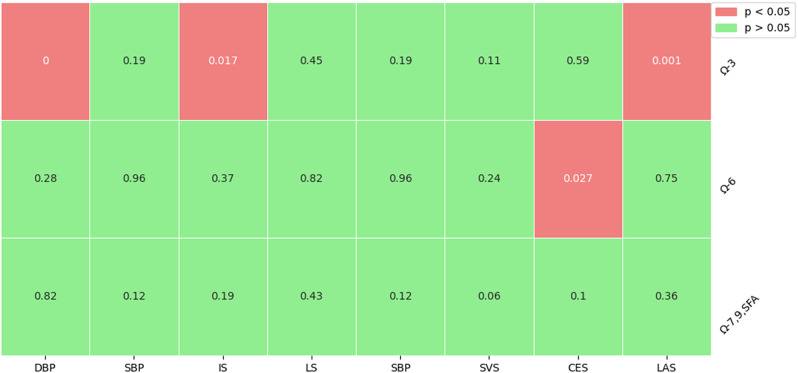
Causal relationship between fatty acids and blood pressure and ischemic stroke

As illustrated in Fig. 2, the analysis identified a significant correlation between Omega-3 fatty acids and DBP(*P* = 2.57e-7, Beta = -0.032), LAS(*P* = 0.001, OR = 1.269) and IS(*P* = 0.017, OR = 1.080).With regard to Omega-6 fatty acids, the analysis suggests a significant causal link with CES(*P* = 0.027, OR = 0.884). For Omega-7, Omega-9, and SFAs, no significant causal relationship was found. In summary, the MVMR approach has identified a potentially significant causal link between Omega-3 fatty acids and blood pressure, as well as between Omega-3 fatty acids and ischemic stroke. To further probe the causal effects of Omega-3 on blood pressure and ischemic stroke, this study implemented Bidirectional MR and Mediation MR studies. These studies used Omega-3 as the exposure, blood pressure as the mediator, and ischemic stroke as the outcome, aiming to explore and validate this causal relationship.

#### Effect of Omega-3 on blood pressure

**Fig. 3:**
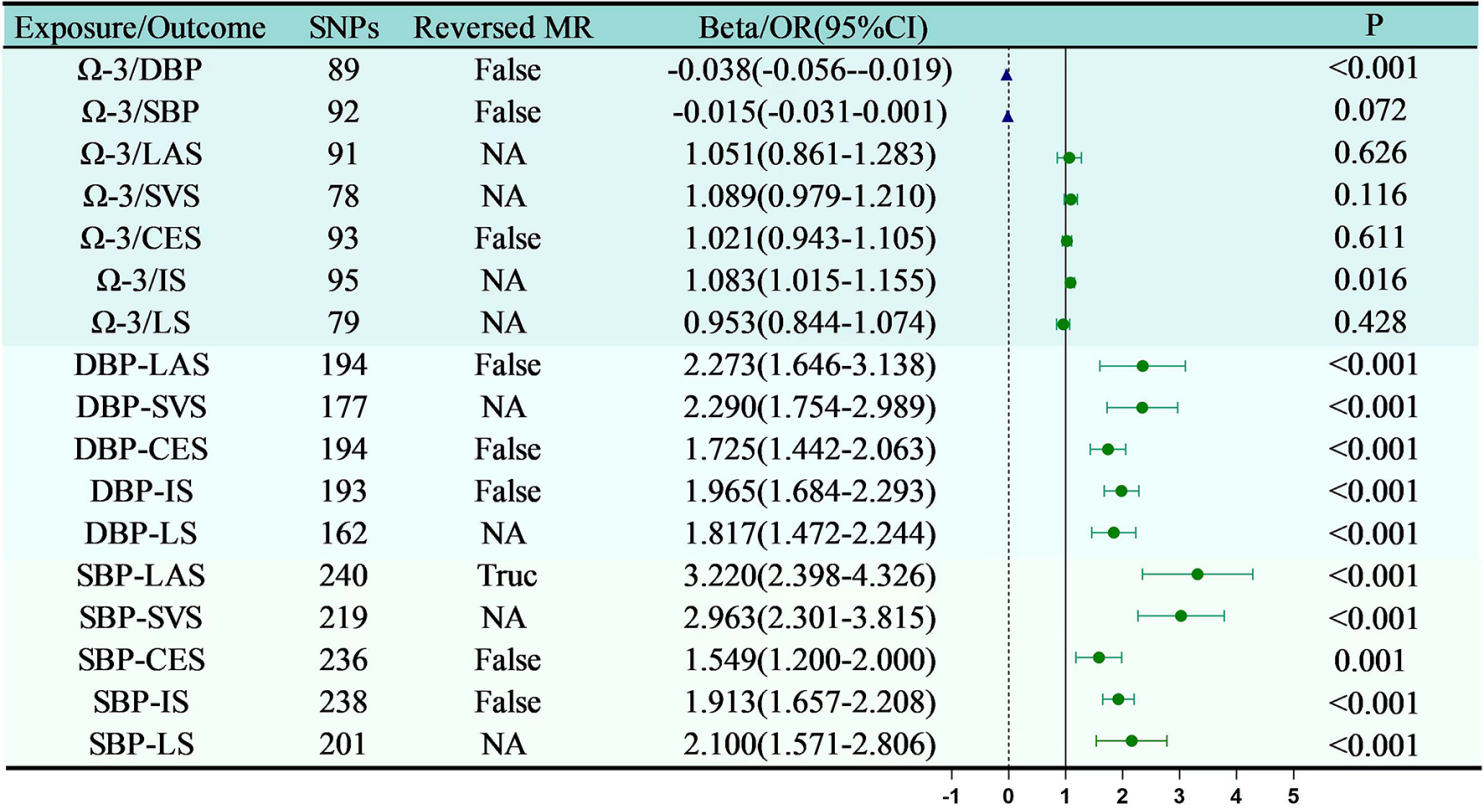
MR Results of Omega-3, blood pressure, and ischemic stroke

Figure 3 demonstrates that Omega-3 exerts a significant negative causal effect on DBP(*P* = 1.01e-04 < 0.05), signifying statistical significance. However, Omega-3 does not show a significant causal effect on SBP(*P* = 0.072). The Weighted Median analysis indicates that an increase in Omega-3 levels substantially reduces DBP (Beta = -0.038, 95% CI: -0.056 to -0.019). Additionally, reverse MR analysis confirms the absence of a significant reverse causal relationship between Omega-3 and DBP (*P* = 0.237). In conclusion, MR analysis suggests that elevating Omega-3 levels can lower DBP, but appears to have little significant impact on SBP.

#### Effect of Omega-3 on ischemic stroke

Figure 3 demonstrates that there is a likely positive causal relationship between Omega-3 and IS(*P* = 0.016, 95% CI: 1.015 to 1.155). Additionally, the research found no causal connection between Omega-3 and LAS(*P* = 0.626),SVS(*P* = 0.116 ), CES(*P* = 0.611 ), and LS(*P* = 0.428 ).In conclusion, the study, employing bidirectional MR, suggests that changes in Omega-3 levels in the human body may have a significant causal effect on IS.

#### Overview of intermediary MR

**Fig. 4:**
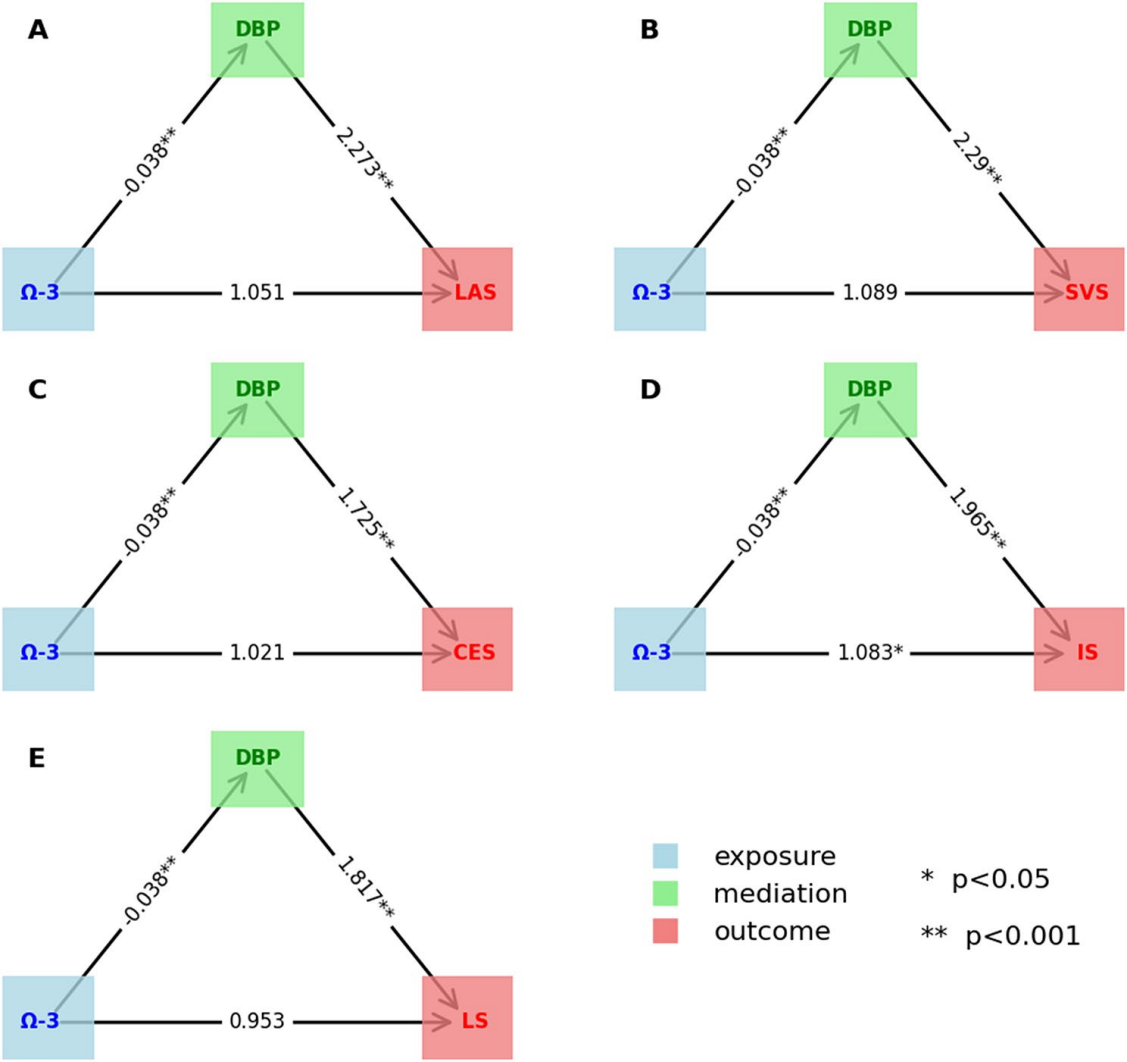
Results of Mediation Mendelian Randomization

**Fig. 5:**
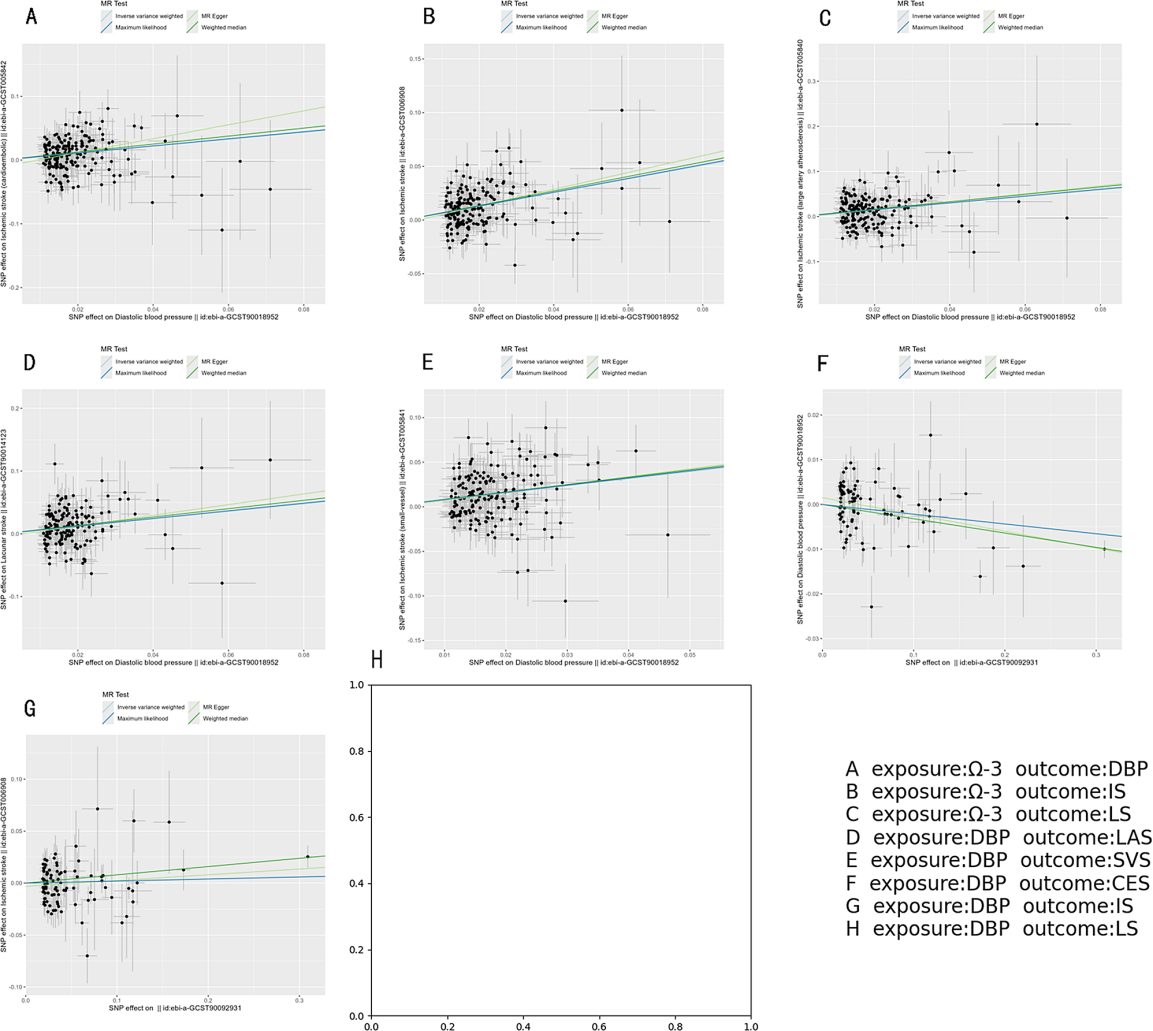
The scatter plot presents the associations between SNPs linked to Omega-3 and both blood pressure and ischemic stroke, following the exclusion of outliers using MR-PRESSO. The analysis utilized several methods, including inverse variance-weighted, weighted median, MR-Egger, and maximum likelihood. The slope of each line in the plot reflects the estimated MR effect as determined by each method

From Figs. 4 and 5, and Table 2, this study identified five mediating pathways. Among these, Omega-3-DBP-SVS, Omega-3-DBP-CES, Omega-3-DBP-LAS and Omega-3-DBP-LS were complete mediations, while Omega-3-DBP-IS were partial mediations. Coefficient testing revealed that all mediation effect coefficients were less than 0, with p-values below 0.05 (as shown in Table 2), indicating significant mediating effects of DBP in the causal relationship between Omega-3 and ischemic stroke. Therefore, the results of mediation MR suggest that DBP likely serves as a mediator between Omega-3 and ischemic stroke; that is, an increase in Omega-3 levels in the body can reduce DBP, thereby lowering the risk of LAS, SVS, CES, IS, and LS.

**Table 2:** Overview of the results of mediation MR

	exposure	mediation	outcome	mediation type	β(95% CI)	*P*
A	Ω-3	DBP	LAS	Complete	-3.084e-02 (-3.602e-02,-3.535e-02)	< 0.001
B	Ω-3	DBP	SVS	Complete	-3.112e-02 (-3.585e-02,-3.552e-02)	< 0.001
C	Ω-3	DBP	CES	Complete	-2.048e-02 (-3.569e-02,-3.568e-02)	< 0.001
D	Ω-3	DBP	IS	Partial	-2.537e-02 (-3.569e-02,-3.568e-02)	< 0.001
E	Ω-3	DBP	LS	Complete	-2.244e-02 (-3.570e-02,-3.567e-02)	< 0.001

**Fig. 6:**
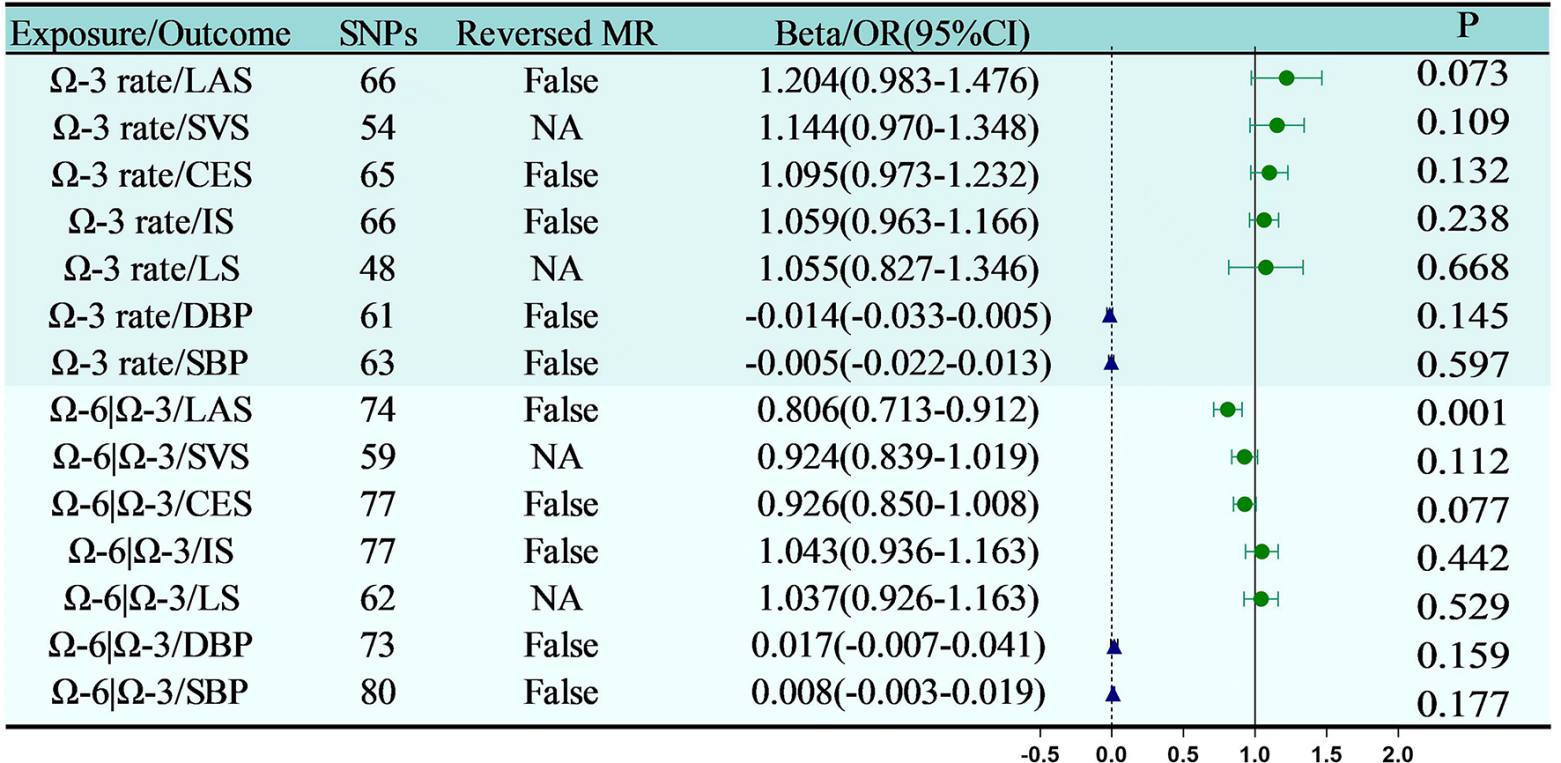
MR Results of Omega-3 rate, Blood pressure and Ischemic Stroke

**Fig. 7:**
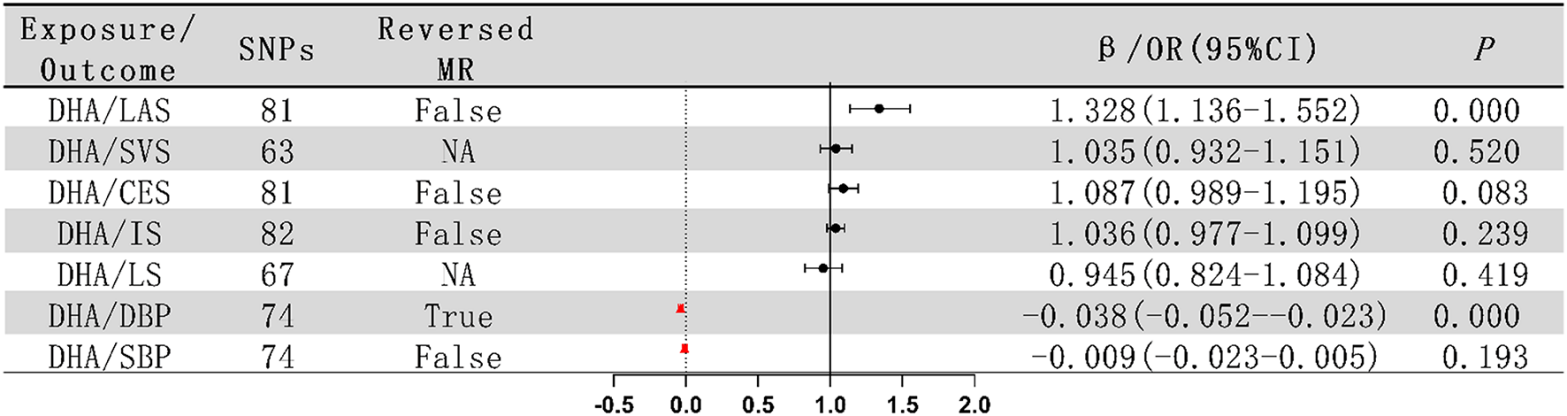
MR Results of DHA, blood pressure and ischemic stroke

#### Effect of Omega-3 rate on blood pressure and ischemic stroke

Figure 6 clearly demonstrates that the ratio of Omega-3 fatty acids to total fatty acids does not have a significant causal relationship with several cardiovascular and cerebrovascular conditions and measurements, including DBP (*P* = 0.145), SBP (*P* = 0.597), IS (*P* = 0.238), LAS (*P* = 0.073), SVS (*P* = 0.109), CES (*P* = 0.132), and LS (*P* = 0.238). However, the ratio of Omega-6 fatty acids to Omega-3 fatty acids shows a significant causal relationship with LAS (*P* = 0.001, 95% CI: 0.713 to 0.912), indicating a potential impact on these health outcomes. Conversely, there is no significant relationship between the ratio of Omega-6 fatty acids to Omega-3 fatty acids with SVS (*P* = 0.112), CES (*P* = 0.077), IS(*P* = 0.442),LS (*P* = 0.529),DBP(*P* = 0.159) or SBP (*P* = 0.177). In summary, bidirectional MR has demonstrated that the ratio of Omega-3 fatty acids to total fatty acids appears to have no causal relationship with blood pressure or ischemic stroke. Conversely, an increased ratio of Omega-6 fatty acids to Omega-3 fatty acids is associated with a higher incidence of LAS.

## Discussion

This research utilized MR, specifically MVMR, bidirectional MR, and mediation MR, to investigate the association between prevalent fatty acids, blood pressure, and ischemic stroke. Among the commonly studied PUFAs, Omega-3 has been shown to effectively regulate blood pressure [29, 62], enhance vascular compliance [63], and improve vascular physiological activity [64], aligning with previous research findings. In contrast, the protective impact of Omega-6 on blood pressure and cerebral vessels appears less pronounced. Excessive consumption of Omega-6 has been linked to promoting inflammatory responses [65], potentially increasing the risks of hypertension and stroke. Therefore, monitoring the intake ratio of Omega-3 to Omega-6 is advisable [66, 67] in preventative measures against CVDs.

To delve deeper into the causal impacts of Omega-3 on blood pressure and ischemic stroke, this investigation employed two-sample MR. It specifically examined the ratio of Omega-3 fatty acids to total fatty acids and the ratio of Omega-6 fatty acids to Omega-3 fatty acids as the exposures, with ischemic stroke as the outcome. The results showed no clear causal relationship between the variation in the ratio of Omega-3 fatty acids to total fatty acids and blood pressure or types of ischemic stroke (Fig. 6). However, a significant causal relationship was found between the ratio of Omega-6 fatty acids to Omega-3 fatty acids and LAS (*P* = 0.001, OR = 0.806, 95% CI: 0.713 to 0.912), with no causal relationship observed with other types of ischemic stroke (Fig. 6). In conclusion, additional research is necessary to investigate the potential protective roles of Omega-6, Omega-7, Omega-9 fatty acids, and SFAs on the cardiovascular and cerebrovascular system.

Experimental research has consistently shown that dietary supplementation with Omega-3 PUFAs can potentially control thrombus formation and vascular occlusion by reducing platelet function [68], thereby lowering the risk of stroke [69]. Additionally, Omega-3 has been observed to aid in neurovascular recovery dynamics and brain repair, ultimately enhancing neurological function post-stroke [70]. Nevertheless, this study’s findings suggest that while Omega-3 may not directly reduce ischemic stroke risk, it can effectively regulate blood pressure within an optimal range, thus diminishing the incidence of ischemic stroke—a mechanism similar to blood pressure’s protective role against ischemic stroke. As for the mechanism of action, prior studies have established that Omega-3 can effectively lower triglyceride-glucose levels in individuals with hypertension [71] and reduce vascular inflammation, thereby mitigating arteriosclerosis [72]. This is achieved through the production of anti-inflammatory and anti-inflammatory lysins [73, 74]. Elevated triglyceride-glucose levels [9] and arteriosclerosis [10] are key factors in stroke development among hypertensive individuals. As a result, while the direct anti-stroke effects of Omega-3 fatty acids may be limited, their role in indirectly reducing stroke risk through multiple biological mechanisms cannot be overlooked. In summary, Omega-3 PUFAs play a significant role in the prevention and treatment of cardiovascular diseases, and their potential as an adjunctive therapeutic approach should be considered in clinical practice. Therefore, in the clinical management of hypertension, it may be beneficial to increase the intake of Omega-3 and reduce Omega-6 in the diet of hypertensive patients, incorporating Omega-3 fatty acids as a part of the adjunctive treatment. This offers a safe and effective method to supplement pharmacological treatments for hypertensive patients. Additionally, given the positive role of Omega-3 in promoting neurovascular recovery and brain repair, it can be considered as a potential nutritional intervention to prevent ischemic strokes (especially LAS and IS) and to reduce complications post-ischemic stroke.

The numerous studies establishing a link between inflammation and the development of hypertension [75, 76] are critical to understanding the broader context of cardiovascular health. Stroke, often a consequence of arteriosclerosis (AS), is primarily characterized by disorders in lipid metabolism, oxidative stress, and inflammation [77, 78]. These inflammatory responses and metabolic irregularities might interfere with the utilization of Omega-3 fatty acids, potentially leading to reduced blood concentrations. However, this study, utilizing reversed MR, did not identify a significant causal relationship between Omega-3 fatty acids and conditions such as blood pressure and ischemic stroke. This could be attributed to the fact that Omega-3 fatty acids are not endogenously produced in the human body and must be ingested through diet, including foods like vegetable oils, fish, and algae. Another possible reason is that the metabolism of Omega-3 in the body is relatively stable, not easily affected by the inflammatory responses and metabolic abnormalities associated with diseases, or the impact of these diseases on Omega-3 is minimal, not reaching the threshold that would affect its physiological functions. Consequently, conditions such as hypertension and ischemic stroke appear to have a minimal impact on the metabolism of Omega-3 fatty acids.

In summary, this research, employing the MR approach, has affirmed the causal relationship between Omega-3 fatty acids and both blood pressure and ischemic stroke. It underscores the beneficial regulatory role of Omega-3 in the cardiovascular and cerebrovascular systems. ALL in all, this finding further emphasizes the important role of Omega-3 in maintaining cardiovascular and cerebrovascular health, particularly its beneficial effects in regulating blood pressure and reducing the risk of ischemic stroke.

### Innovation and limitation

This study comprehensively applies bidirectional MR and mediator MR methods, with strict selection of SNPs and rigorous screening standards and analytical strategies to ensure the credibility of the results. It elucidated the preventive role of fatty acids, especially Omega-3 fatty acids, in relation to ischemic stroke, and provided evidence for the mechanism by which Omega-3 fatty acids may reduce ischemic stroke risk by protecting blood pressure, particularly DBP, using the MR approach. Consequently, this study establishes a foundation for using the MR method in future research efforts aimed at investigating the causal links between fatty acids and other CVDs, such as coronary heart disease and myocardial infarction and offers a robust framework for understanding the potential therapeutic benefits of Omega-3 fatty acids in the context of heart and brain health. Besides, the framework of this study can be applied to research on the causal relationships between other nutrients and various diseases, promoting the development of personalized medicine and precise prevention strategies. However, it does have limitations.

#### Selection of instrumental variables

Adopting a strict p-value threshold of < 5e-8 for selecting instrumental variables would yield only a limited number of SNPs. Consequently, this study chose a more lenient threshold (*P* < 5e-6) for selecting instrumental variables related to fatty acids.

#### Influence of various factors

An expanding volume of research indicates that the effectiveness of Omega-3 fatty acids in guarding against cardiovascular and cerebrovascular diseases may be influenced by dosage [79, 80], ethnicity [51], gender [81], and dietary culture [82]. Unfortunately, this study could not locate datasets stratified by these factors for Omega-3 content, thus limiting the scope for further research.

#### The lack of analysis of SNPs

The main objective of this study is to apply MR to uncover the causal relationship between Omega-3 and both blood pressure and stroke. During the research process, this study identified some SNPs associated with blood pressure and stroke. These SNPs can help elucidate the specific mechanisms and potential targets of Omega-3, providing guidance for the prevention of clinical stroke and hypertension. However, to further analyze these SNPs, it is necessary to apply bioinformatics methods such as functional annotation, expression quantitative trait loci (eQTL) analysis, and pathway analysis to deeply investigate the biological functions and mechanisms of these SNPs. Nevertheless, these bioinformatics methods are entirely different from the MR and are not the focus of this study, hence there is a lack of detailed analysis in this area.

#### Role of EPA and DHA

Some studies report that Eicosapentaenoic acid (EPA) and Docosahexaenoic acid (DHA) effectively reduce the incidence and mortality rates of CVDs [83], while others find no significant association between EPA, DHA, and CVDs [69]. In response, this study conducted two-sample MR with DHA as the exposure, examining its impact on blood pressure and ischemic stroke. According to Fig. 7, DHA shows a significant association with LAS (*P* = 3.73e-04, 95% CI: 1.136 to 1.552) and DBP (*P* = 6.91e-07, 95% CI: -0.052 to -0.023). However, the discovery of a bidirectional causal relationship between DHA and DBP (*P* = 0.028 < 0.05) challenges the initial hypothesis of a causal link between DHA and DBP. No evident causal relationship was found between DHA and other blood pressure aspects or the incidence of ischemic stroke (Supplementary material 7). Additionally, no datasets for EPA were found in this study. As the database continues to be updated, further research can be conducted on the preventive and therapeutic roles of common types of Omega-3 fatty acids in managing blood pressure and ischemic stroke. This will provide more scientific evidence and reasonable guidance for the prevention and dietary management of ischemic stroke in clinical practice.

## Conclusions

This study, utilizing the MR approach, has substantiated the influence of PUFAs on blood pressure and ischemic stroke. The findings confirm the effective regulatory action of Omega-3 on blood pressure, assisting patients with hypertension in maintaining their blood pressure within an optimal range. Additionally, by employing mediation MR and bidirectional MR methods, the study revealed that while Omega-3 may not directly diminish the incidence of ischemic stroke, it potentially reduces the risk of ischemic stroke by lowering DBP. This indirect mechanism suggests a significant role for Omega-3 in the management of factors contributing to ischemic stroke, highlighting its importance in cardiovascular health strategies.

## Electronic supplementary material

Below is the link to the electronic supplementary material.

Supplementary Material 1

Supplementary Material 2

Supplementary Material 3

Supplementary Material 4

Supplementary Material 5

Supplementary Material 6

Supplementary Material 7

Supplementary Material 8

## Data Availability

The datasets utilized in this study are available in online repositories, specifically at www.gwas.mrcieu.ac.uk. Detailed information regarding SNPs and their associations with unsaturated fatty acids, blood pressure, and ischemic stroke can be found in the supplementary materials. For additional information or specific inquiries, please directly contact the corresponding author of the study.

## Abbreviations

IVW: Inverse variance weighted
IVs: Instrumental variables
MR: Mendelian randomization
MVMR: Multivariable Mendelian randomization
GWAS: Genome-wide association study
SNPs: Single nucleotide polymers
WHO: World Health Organization
CVDS: Cardiovascular diseases
CAD: Coronary artery disease
CBD: Cerebrovascular disease
PAD: Peripheral arterial disease
RHD: Rheumatic heart disease
CHD: Congenital heart disease
DVT: Deep vein thrombosis
PE: Pulmonary embolism
DALYs: Disability-adjusted life years
TFAs: Total fatty acids
SFAs: Saturated fatty acids
PUFAs: Polyunsaturated fatty acids
MUFAs: Monounsaturated fatty acids
AS: Arteriosclerosis
EPA: Eicosapentaenoic acid
DHA: Docosahexaenoic acid
CES: Cardioembolic stroke
SVS: Small vessel stroke
LAS: Large artery atherosclerosis stroke
Ω-3: Omega-3
DBP: Diastolic blood pressure
SBP: Systolic blood pressure
IS: Ischemic stroke
LS: Lacunar stroke
Ω-3 rate: Ratio of Omega-3 fatty acids to total fatty acids
Ω-6/Ω-3: Ratio of Omega-6 fatty acids to Omega-3 fatty acids
