# Supplementary material for: Can Omega-3 prevent the accidence of stroke: a mendelian randomization study
Source: Hereditas. 2024 Sep 5;161:30. doi: 10.1186/s41065-024-00329-9 (PMC11375838; doi:10.1186/s41065-024-00329-9)
Supplement: Supplementary file 8 — Supplementary Material 8 [file 41065_2024_329_MOESM8_ESM.pdf]

omegas-DBP

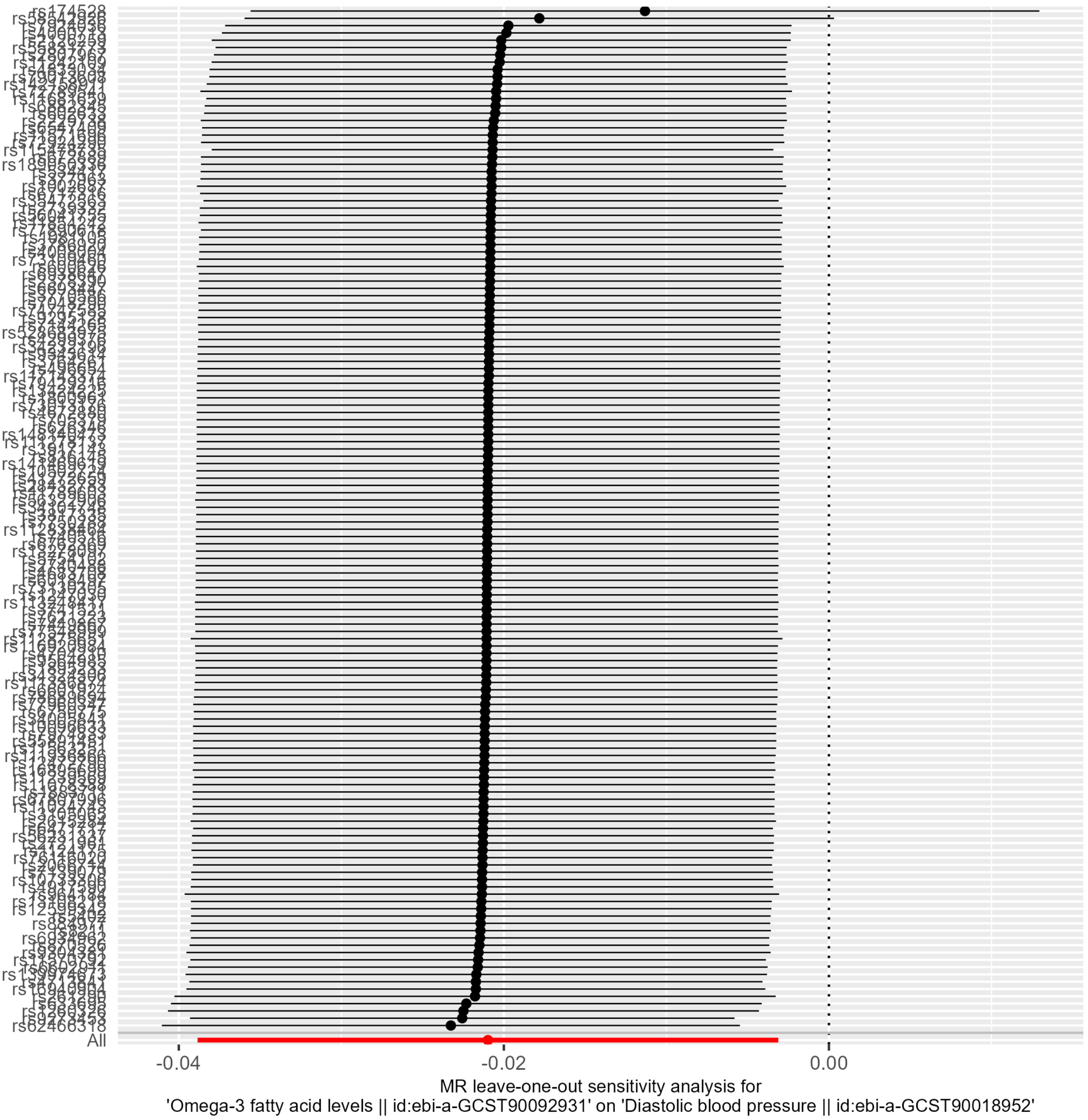

omegas-SBP

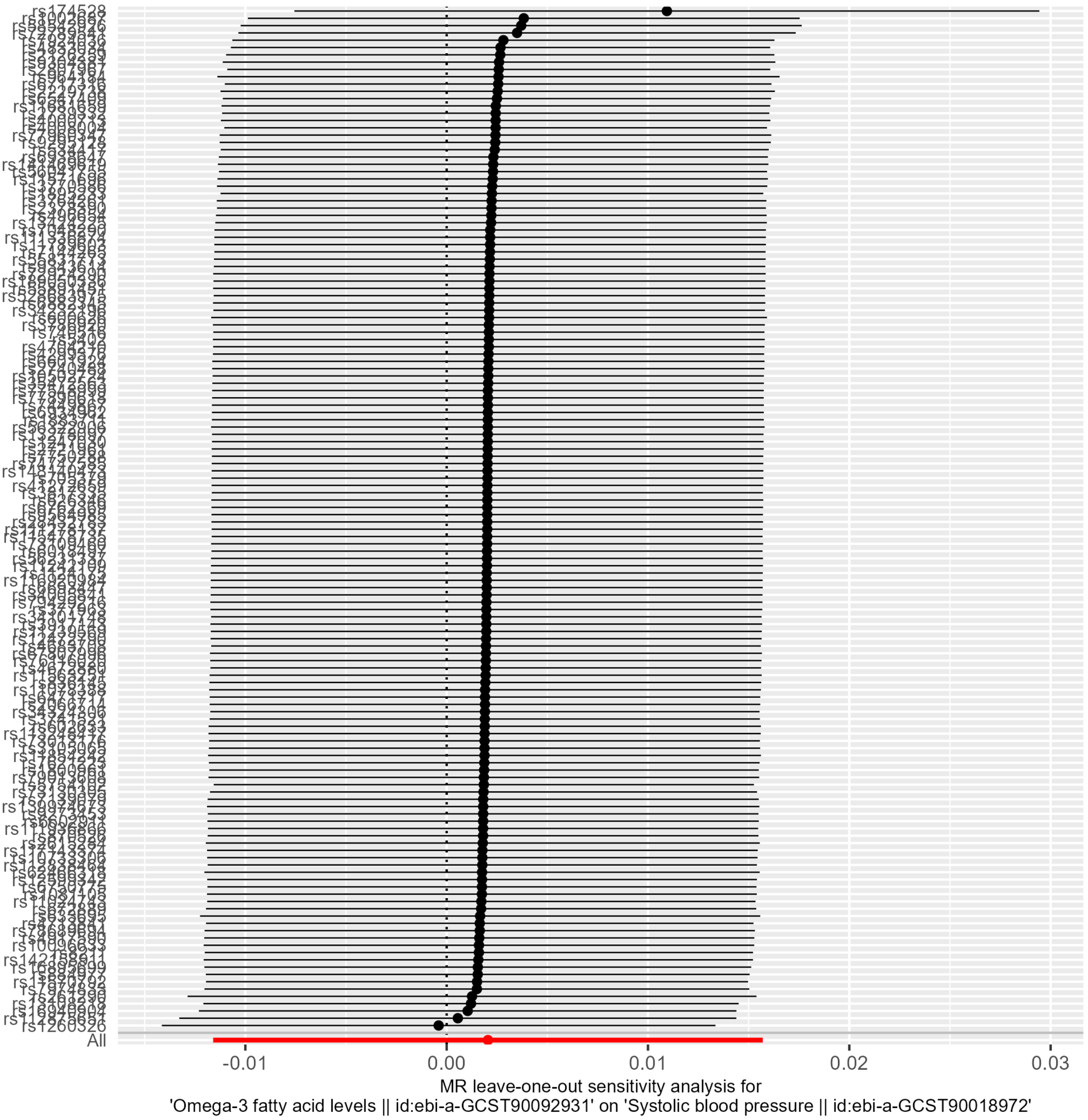
rs174528
All
-0.01
0.00
0.01
0.02
0.03
MR leave-one-out sensitivity analysis for
'Omega-3 fatty acid levels || id:ebi-a-GCST90092931' on 'Systolic blood pressure || id:ebi-a-GCST90018972'

omegas-EH

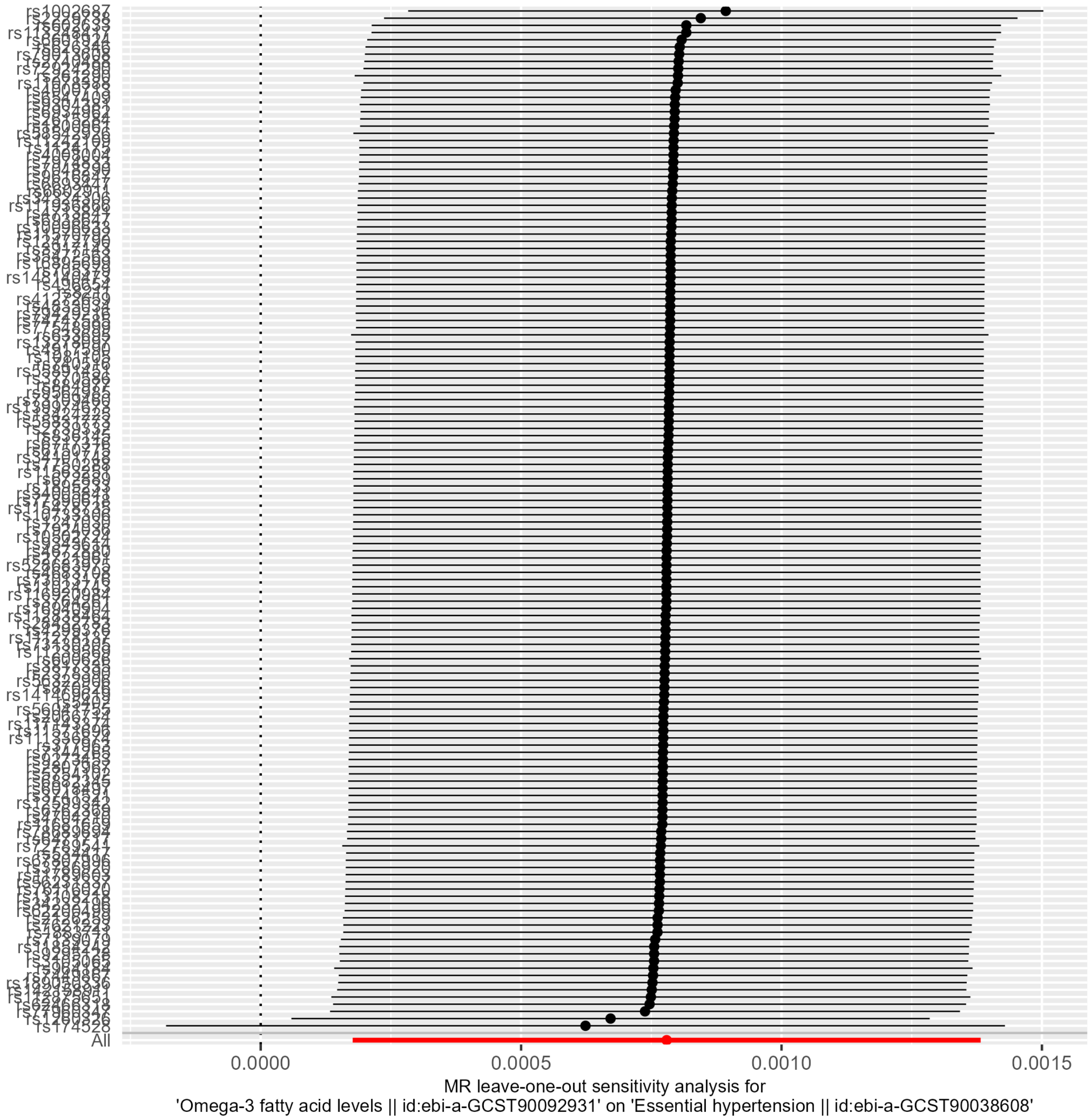
All
0.0000
0.0005
0.0010
0.0015
MR leave-one-out sensitivity analysis for
'Omega-3 fatty acid levels || id:ebi-a-GCST90092931' on 'Essential hypertension || id:ebi-a-GCST90038608'

omegas-DBP

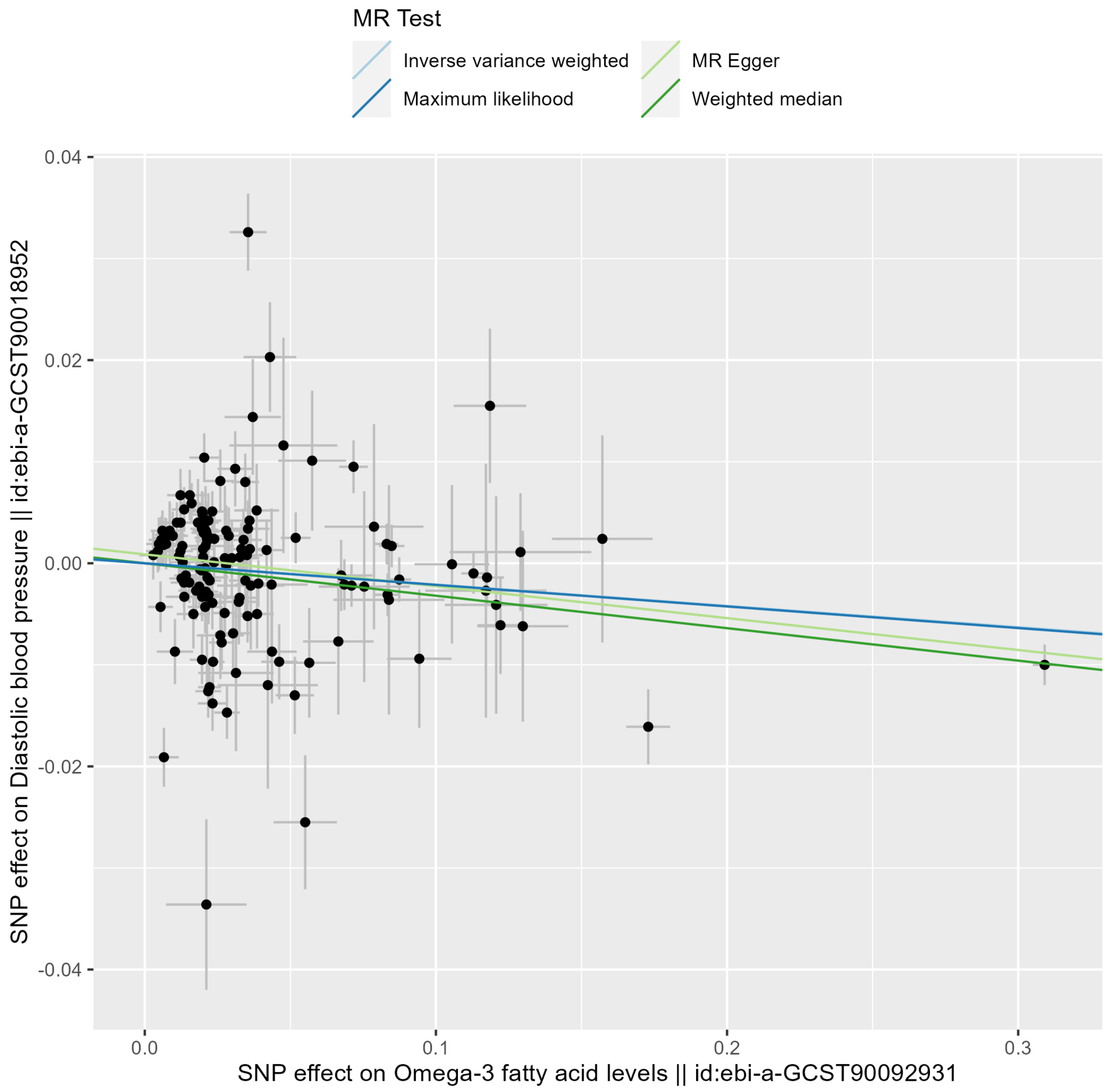
MR Test
Inverse variance weighted
Maximum likelihood
MR Egger
Weighted median
0.04
0.02
0.00
-0.02
-0.04
SNP effect on Diastolic blood pressure || id:ebi-a-GCST90018952
0.0
0.1
0.2
0.3
SNP effect on Omega-3 fatty acid levels || id:ebi-a-GCST90092931

omegas-SBP

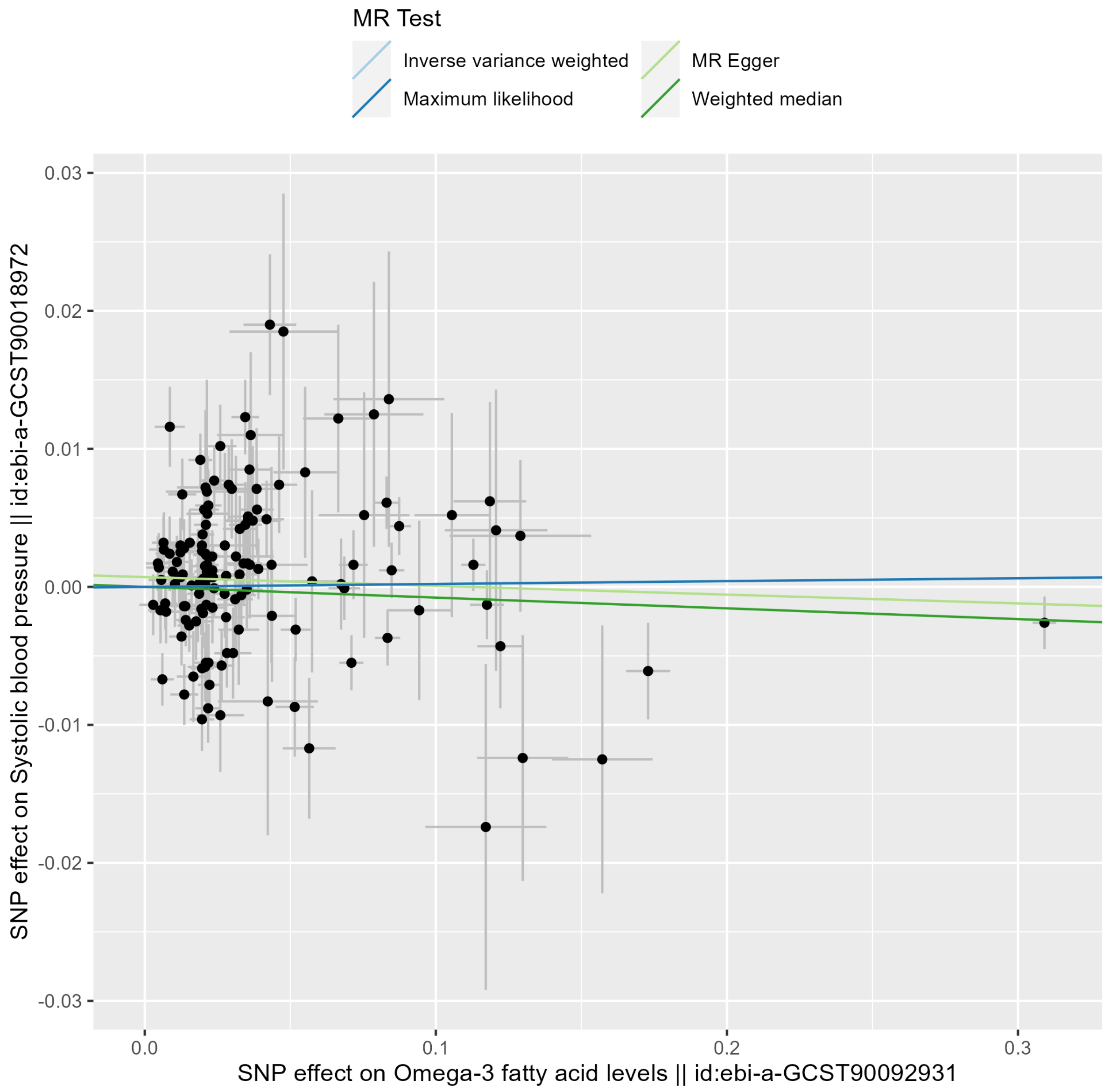
MR Test
Inverse variance weighted
Maximum likelihood
MR Egger
Weighted median
0.03
0.02
0.01
0.00
-0.01
-0.02
-0.03
0.0
0.1
0.2
0.3
SNP effect on Systolic blood pressure || id:ebi-a-GCST90018972
SNP effect on Omega-3 fatty acid levels || id:ebi-a-GCST90092931

omegas-EH

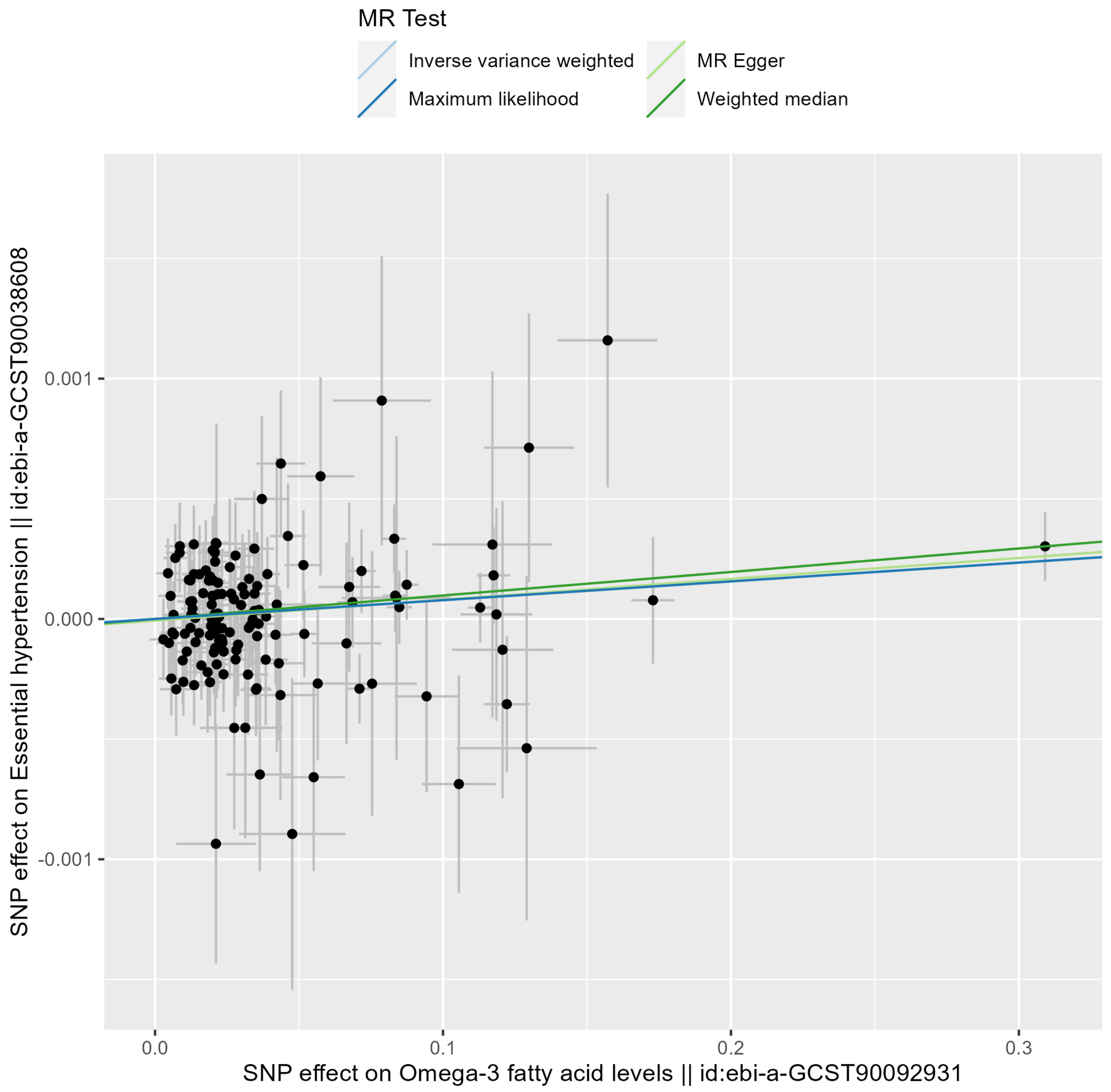
MR Test
Inverse variance weighted
Maximum likelihood
MR Egger
Weighted median
SNP effect on Essential hypertension || id:ebi-a-GCST90038608
0.001
0.000
-0.001
0.0
0.1
0.2
0.3
SNP effect on Omega-3 fatty acid levels || id:ebi-a-GCST90092931

omegas-DBP

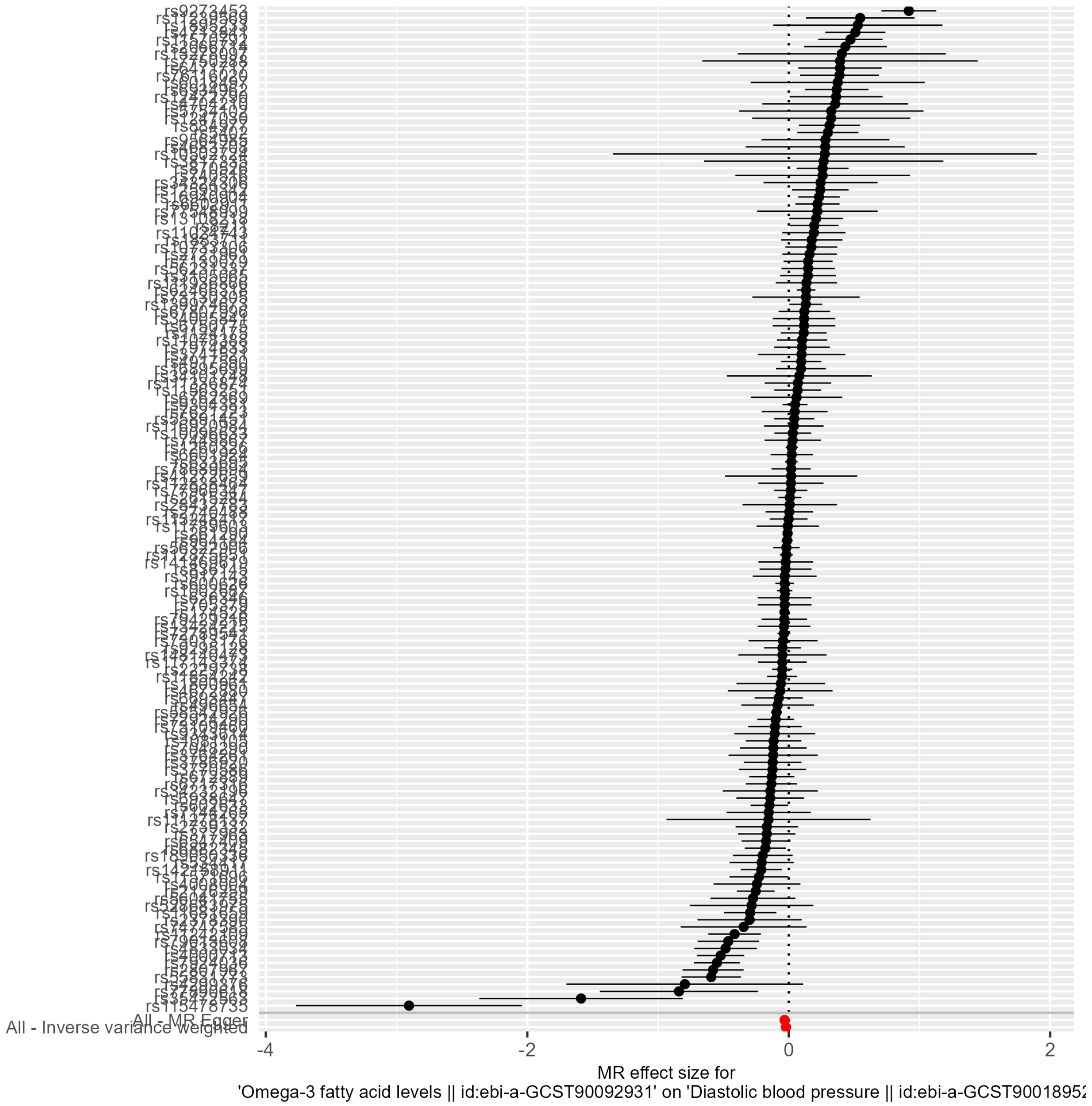
rs9273452
rs115478735
All - MR Egger
All - Inverse variance weighted
-4
-2
0
2
MR effect size for
'Omega-3 fatty acid levels || id:ebi-a-GCST90092931' on 'Diastolic blood pressure || id:ebi-a-GCST9001895

omegas-SBP

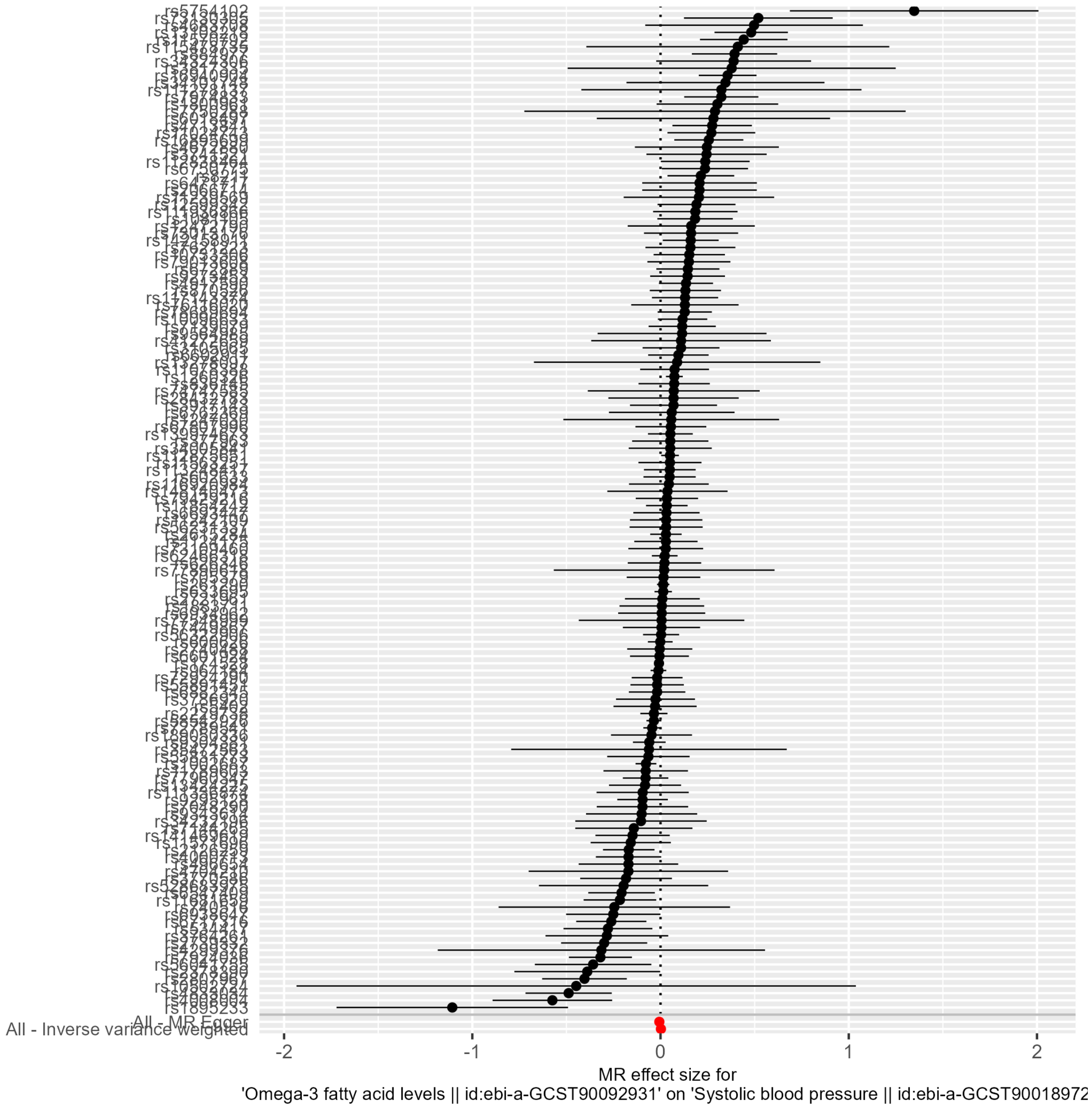
rs5754102
rs1895233
All - MR Egger
All - Inverse variance weighted
-2
-1
0
1
2
MR effect size for
'Omega-3 fatty acid levels || id:ebi-a-GCST90092931' on 'Systolic blood pressure || id:ebi-a-GCST90018972

omegas-EH

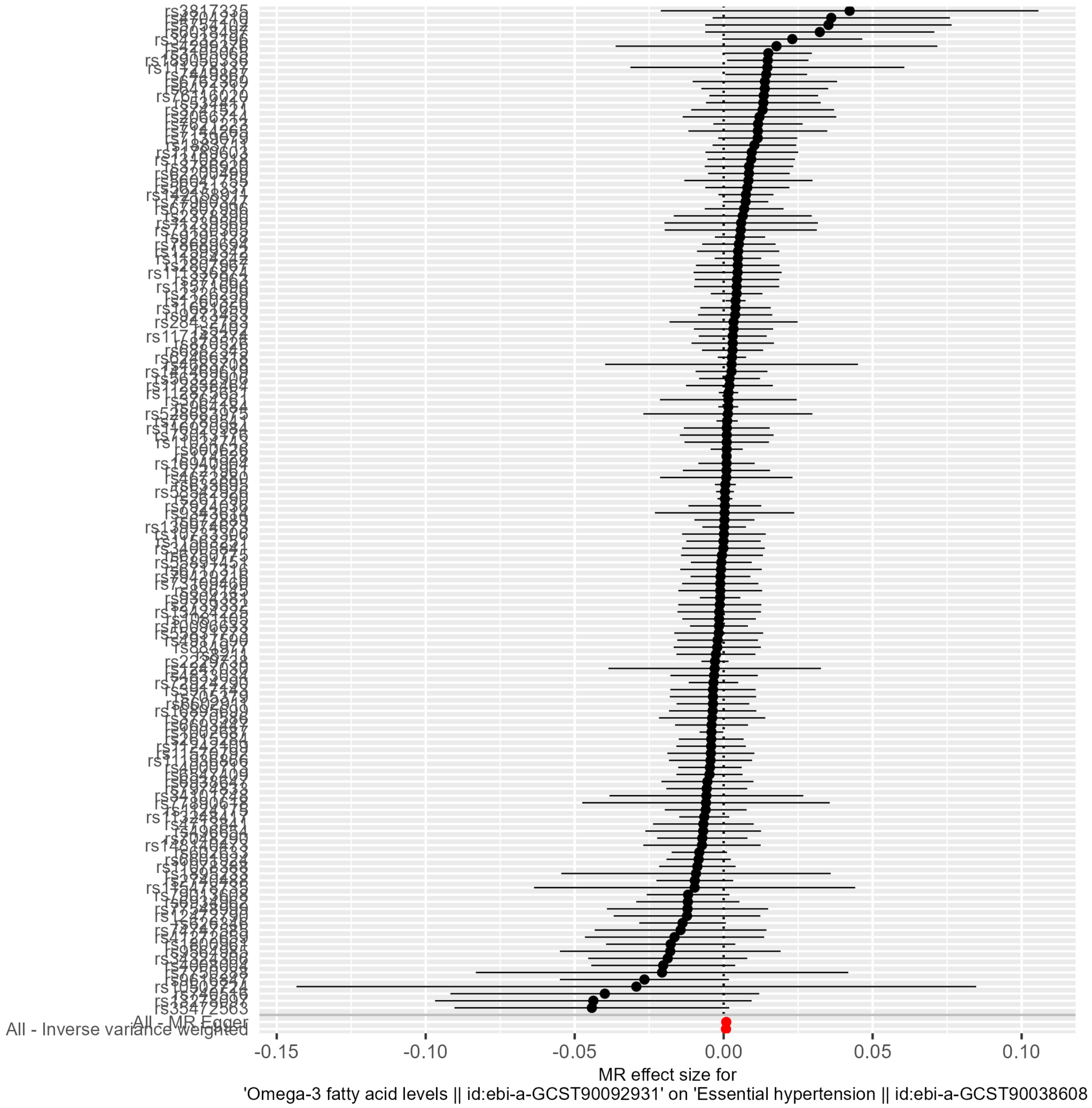
All - MR Egger
All - Inverse variance weighted
-0.15
-0.10
-0.05
0.00
0.05
0.10
MR effect size for
'Omega-3 fatty acid levels || id:ebi-a-GCST90092931' on 'Essential hypertension || id:ebi-a-GCST90038608

omegas-DBP

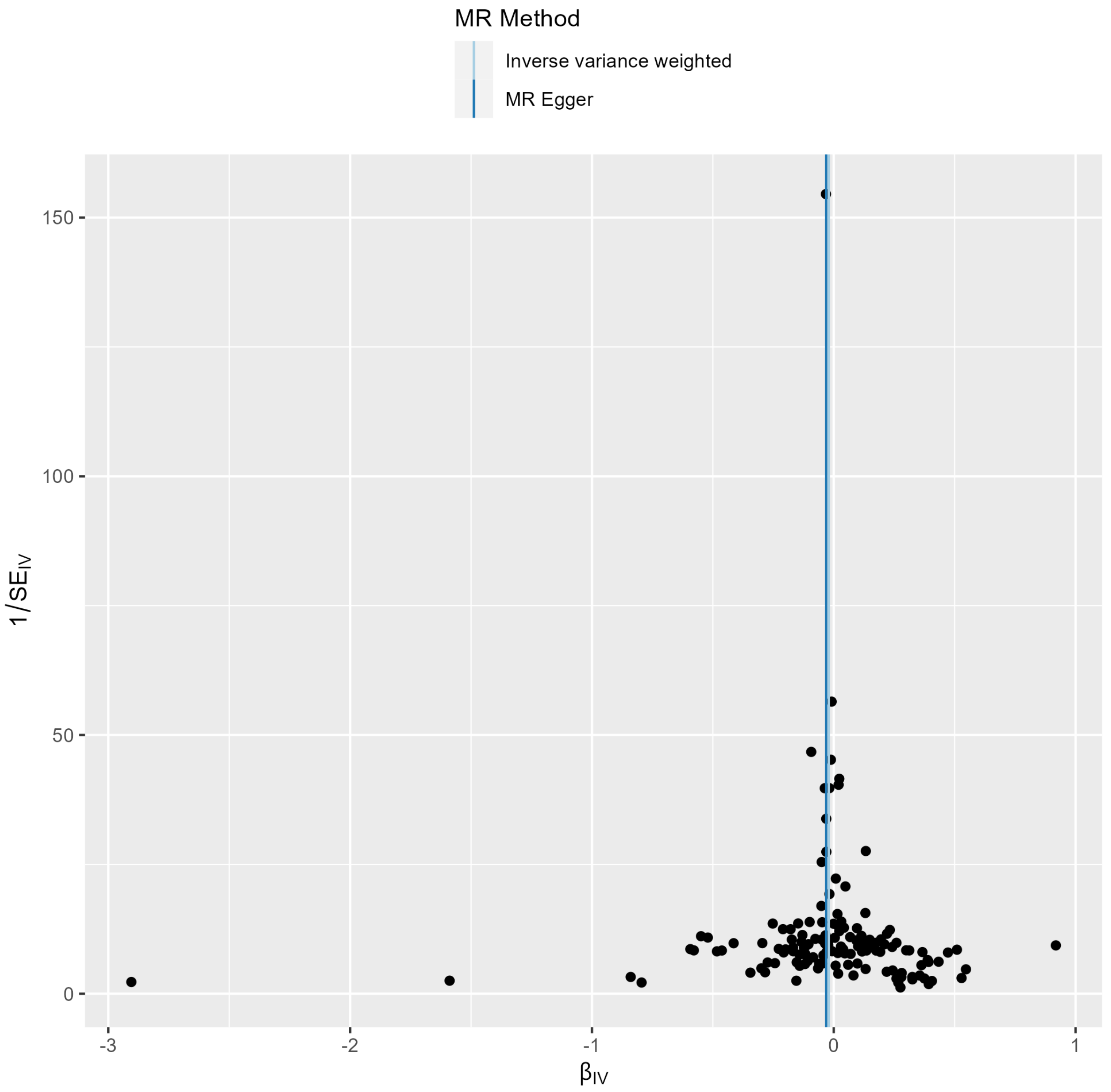
MR Method
Inverse variance weighted
MR Egger
150
100
50
0
1/SE$_{IV}$
-3
-2
-1
0
1
β_{IV}

omegas-SBP

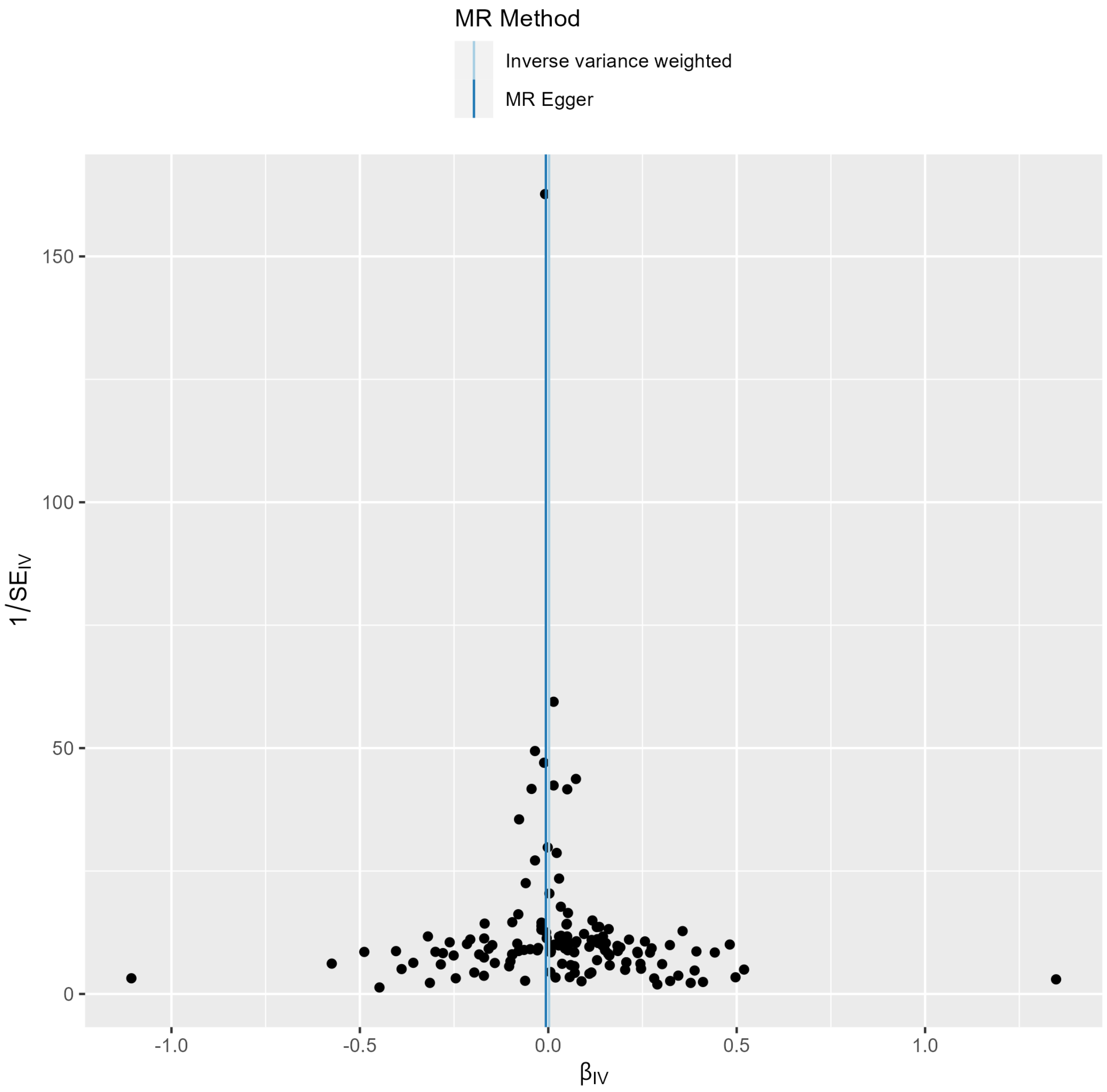
MR Method
Inverse variance weighted
MR Egger
150
100
50
0
1/SE_IV
-1.0
-0.5
0.0
0.5
1.0
β_IV

omegas-EH

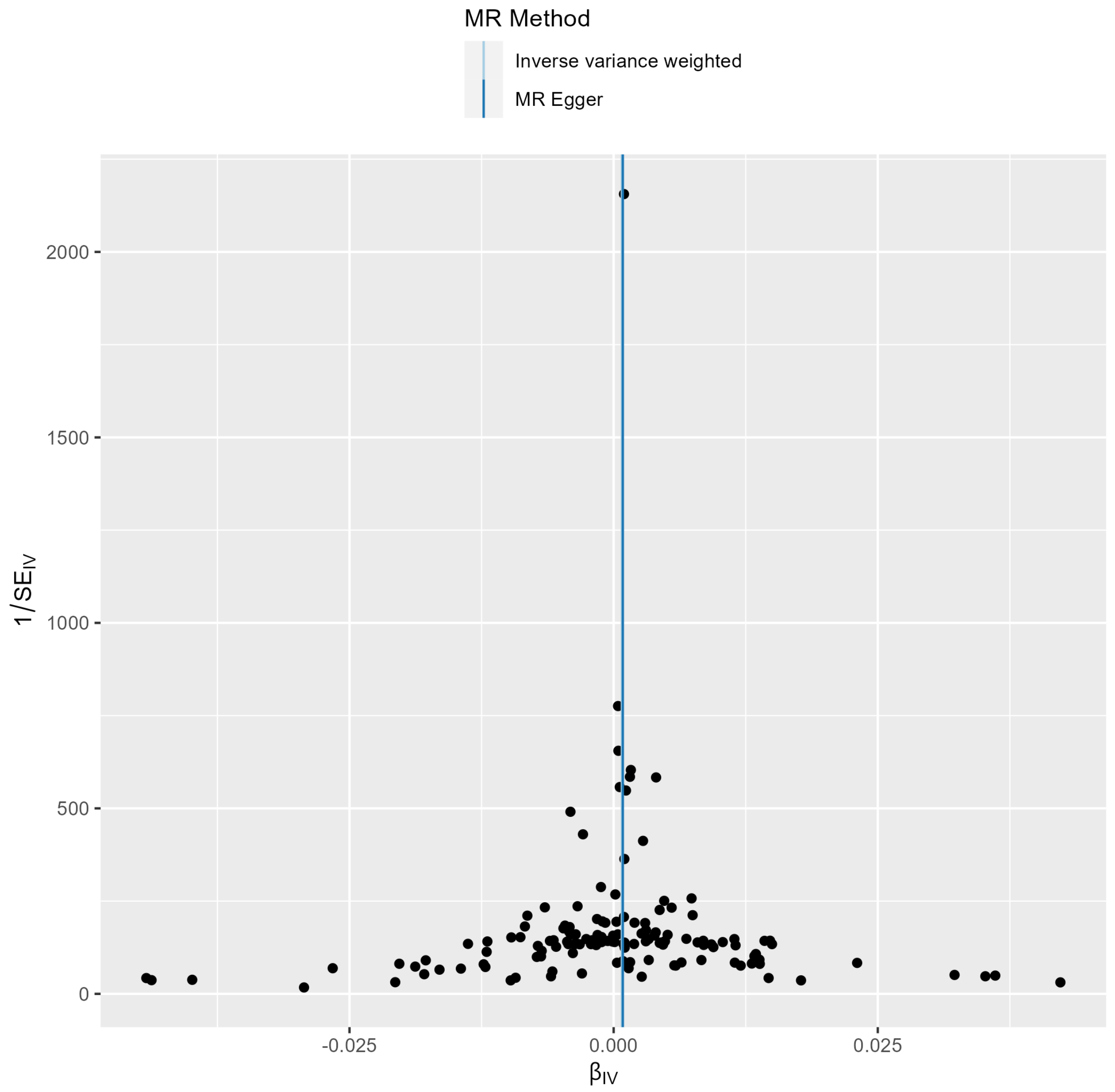
MR Method
Inverse variance weighted
MR Egger
2000
1500
1000
500
0
1/SE_IV
-0.025
0.000
0.025
β_IV

omegas-LAS

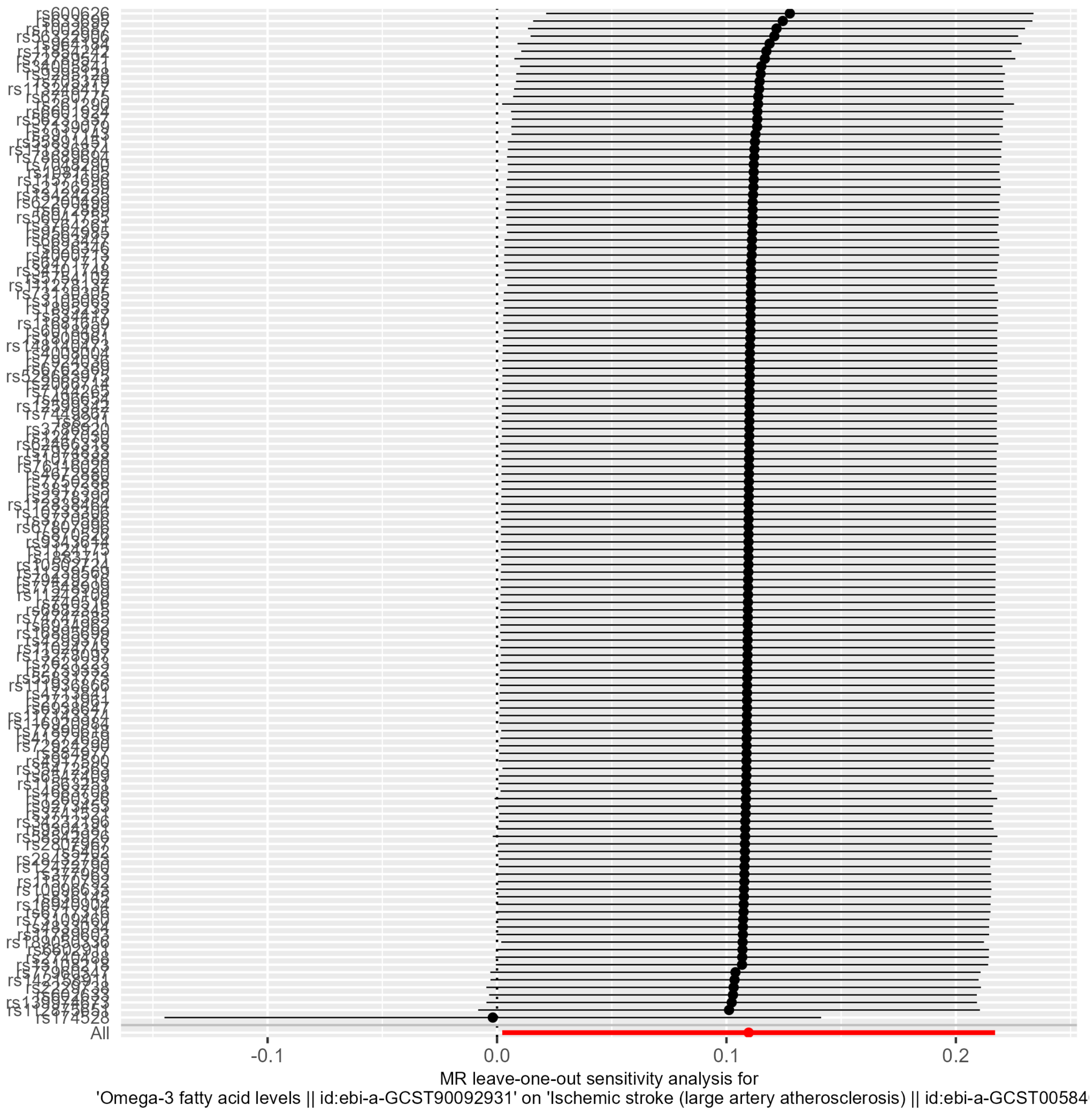
rs600626
rs174528
All
-0.1
0.0
0.1
0.2
MR leave-one-out sensitivity analysis for
'Omega-3 fatty acid levels || id:ebi-a-GCST90092931' on 'Ischemic stroke (large artery atherosclerosis) || id:ebi-a-GCST00584

omegas-SVS

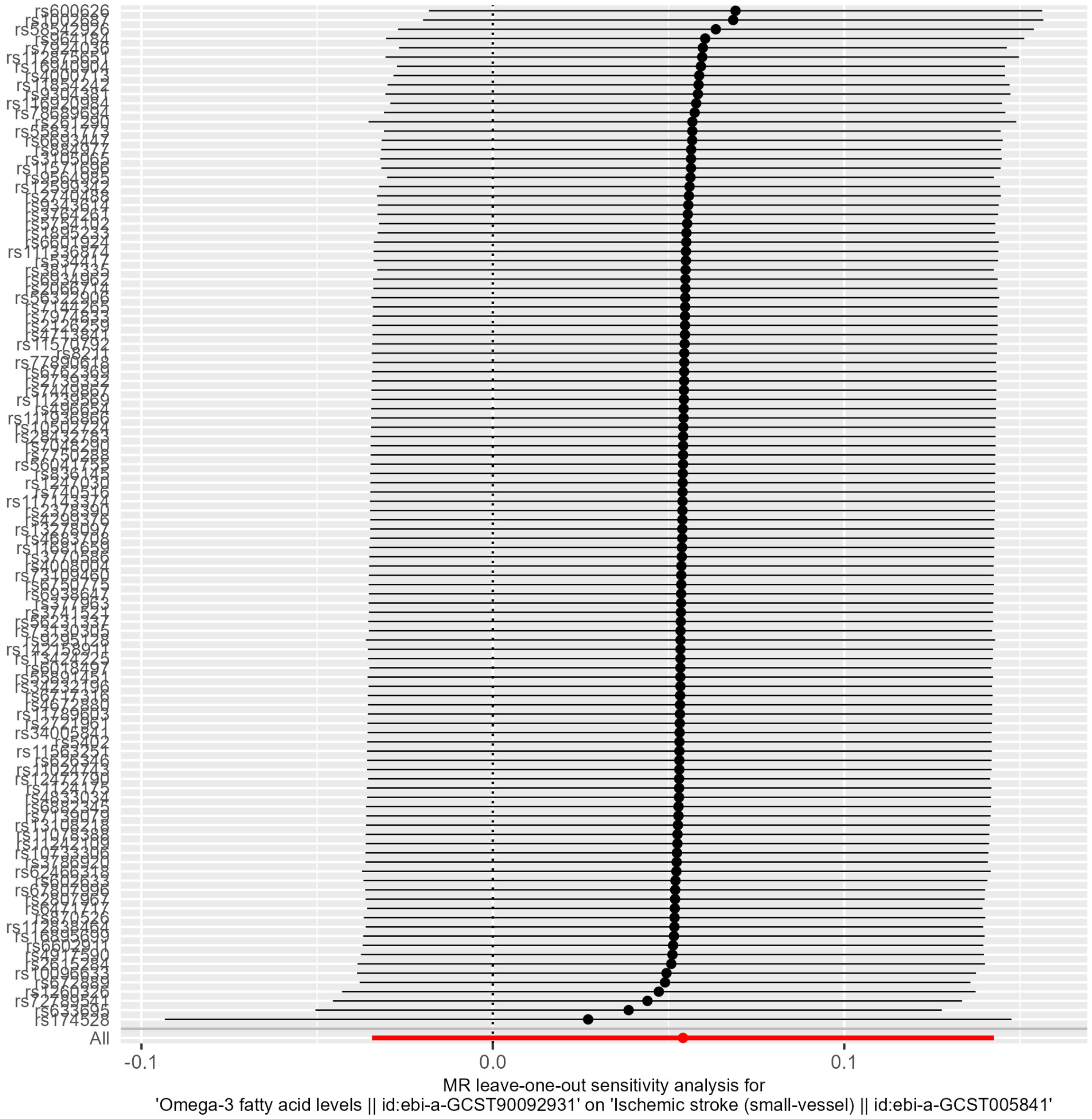
rs600626
rs174528
All
-0.1
0.0
0.1
MR leave-one-out sensitivity analysis for
'Omega-3 fatty acid levels || id:ebi-a-GCST90092931' on 'Ischemic stroke (small-vessel) || id:ebi-a-GCST005841'

omegas-CES

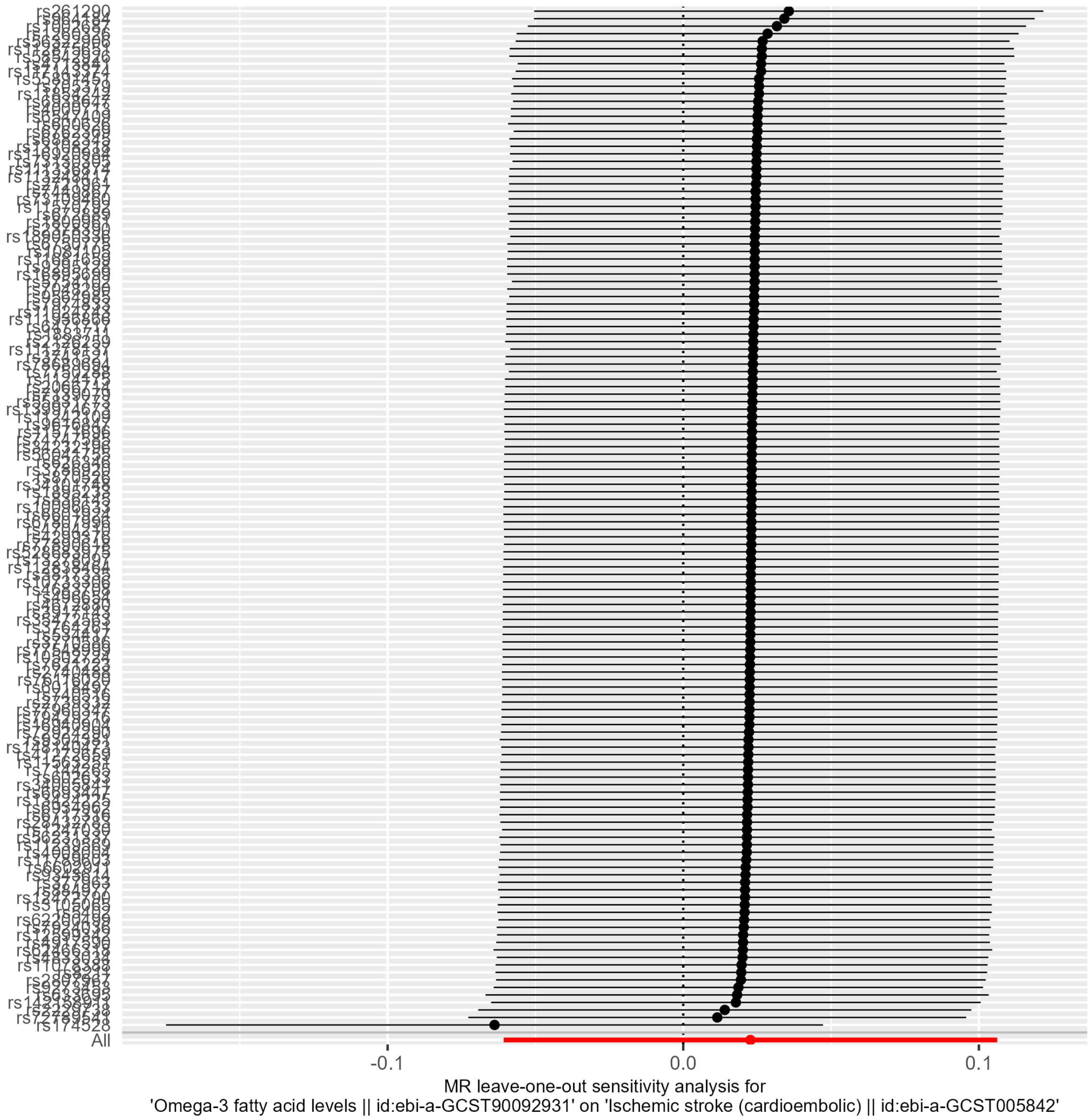
rs261290
rs174528
All
-0.1
0.0
0.1
MR leave-one-out sensitivity analysis for
'Omega-3 fatty acid levels || id:ebi-a-GCST90092931' on 'Ischemic stroke (cardioembolic) || id:ebi-a-GCST005842'

omegas-IS

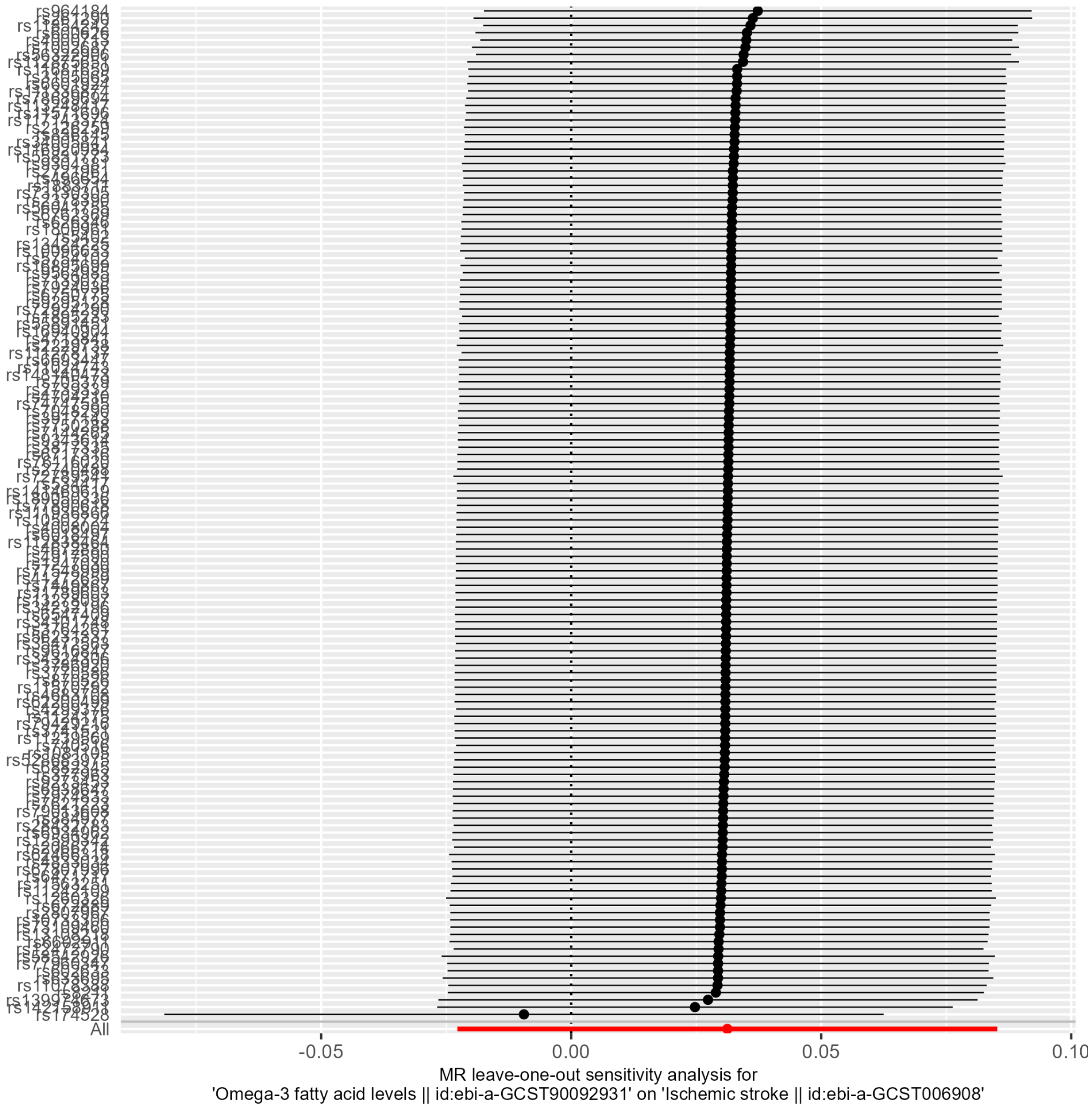
rs964184
All
-0.05
0.00
0.05
0.10
MR leave-one-out sensitivity analysis for
'Omega-3 fatty acid levels || id:ebi-a-GCST90092931' on 'Ischemic stroke || id:ebi-a-GCST006908'

omegas-LS

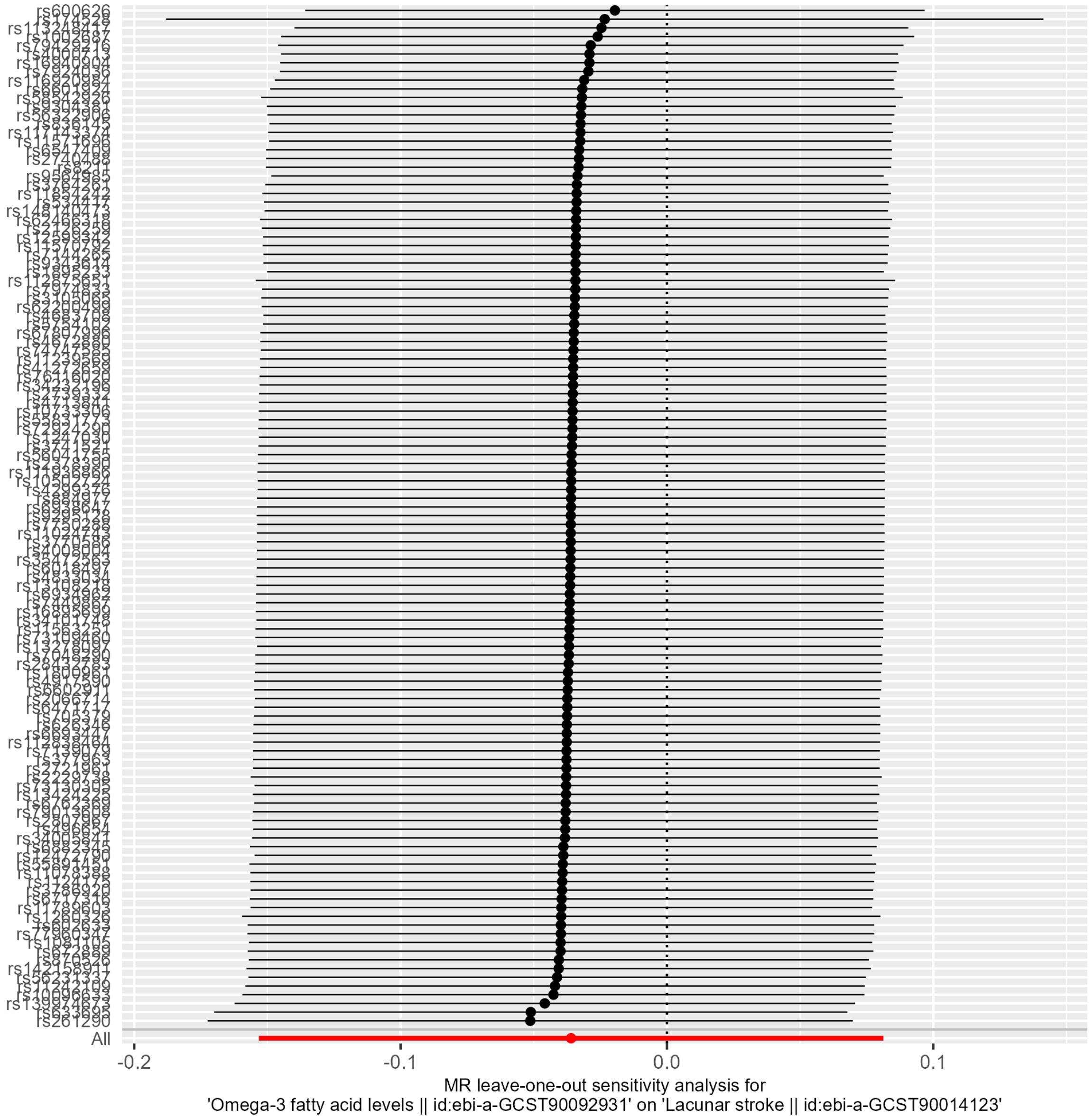
rs600626
rs261290
All
-0.2
-0.1
0.0
0.1
MR leave-one-out sensitivity analysis for
'Omega-3 fatty acid levels || id:ebi-a-GCST90092931' on 'Lacunar stroke || id:ebi-a-GCST90014123'

omegas-LAS

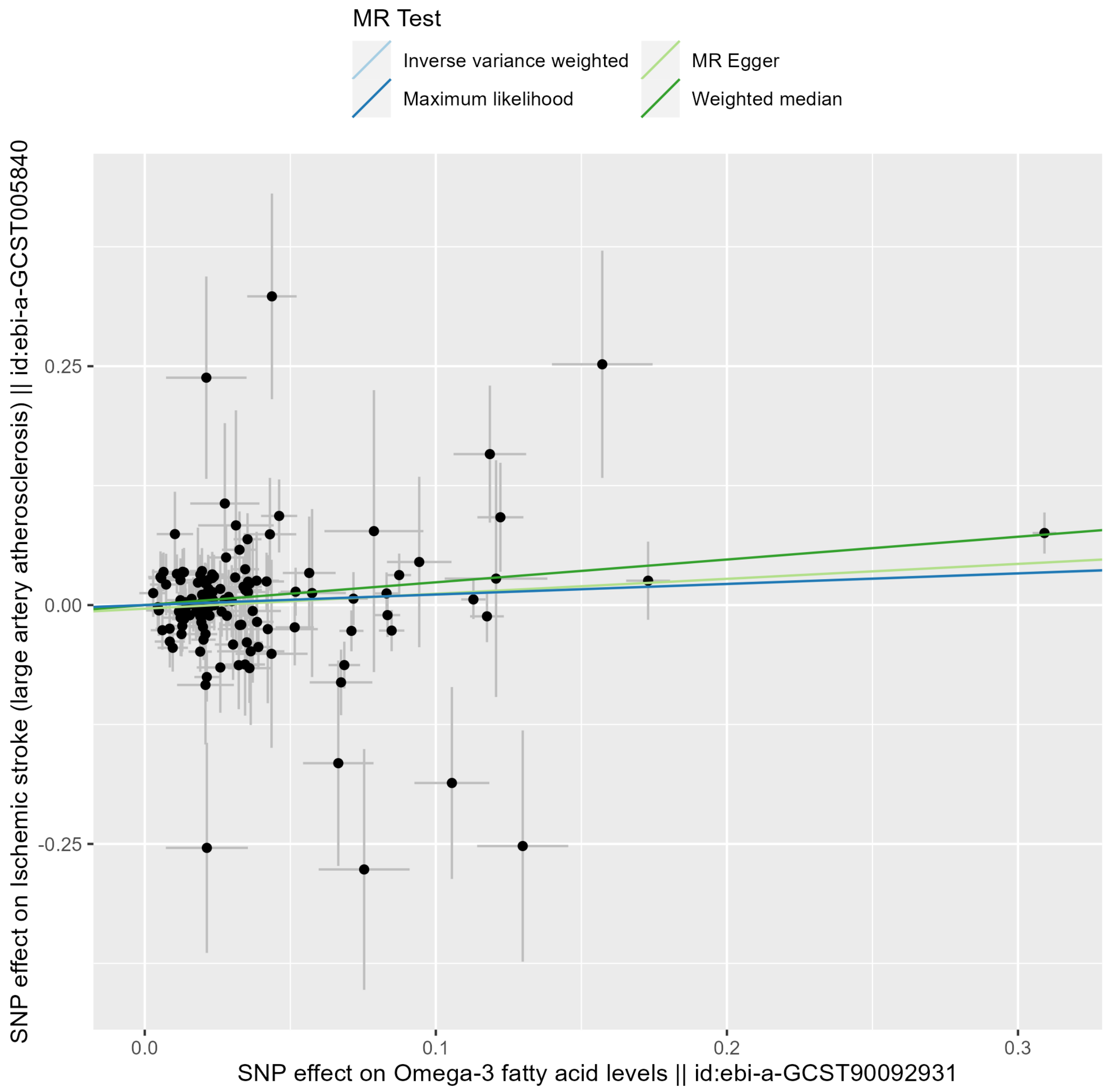
MR Test
Inverse variance weighted
Maximum likelihood
MR Egger
Weighted median
SNP effect on Ischemic stroke (large artery atherosclerosis) || id:ebi-a-GCST005840
0.25
0.00
-0.25
0.0
0.1
0.2
0.3
SNP effect on Omega-3 fatty acid levels || id:ebi-a-GCST90092931

omegas-SVS

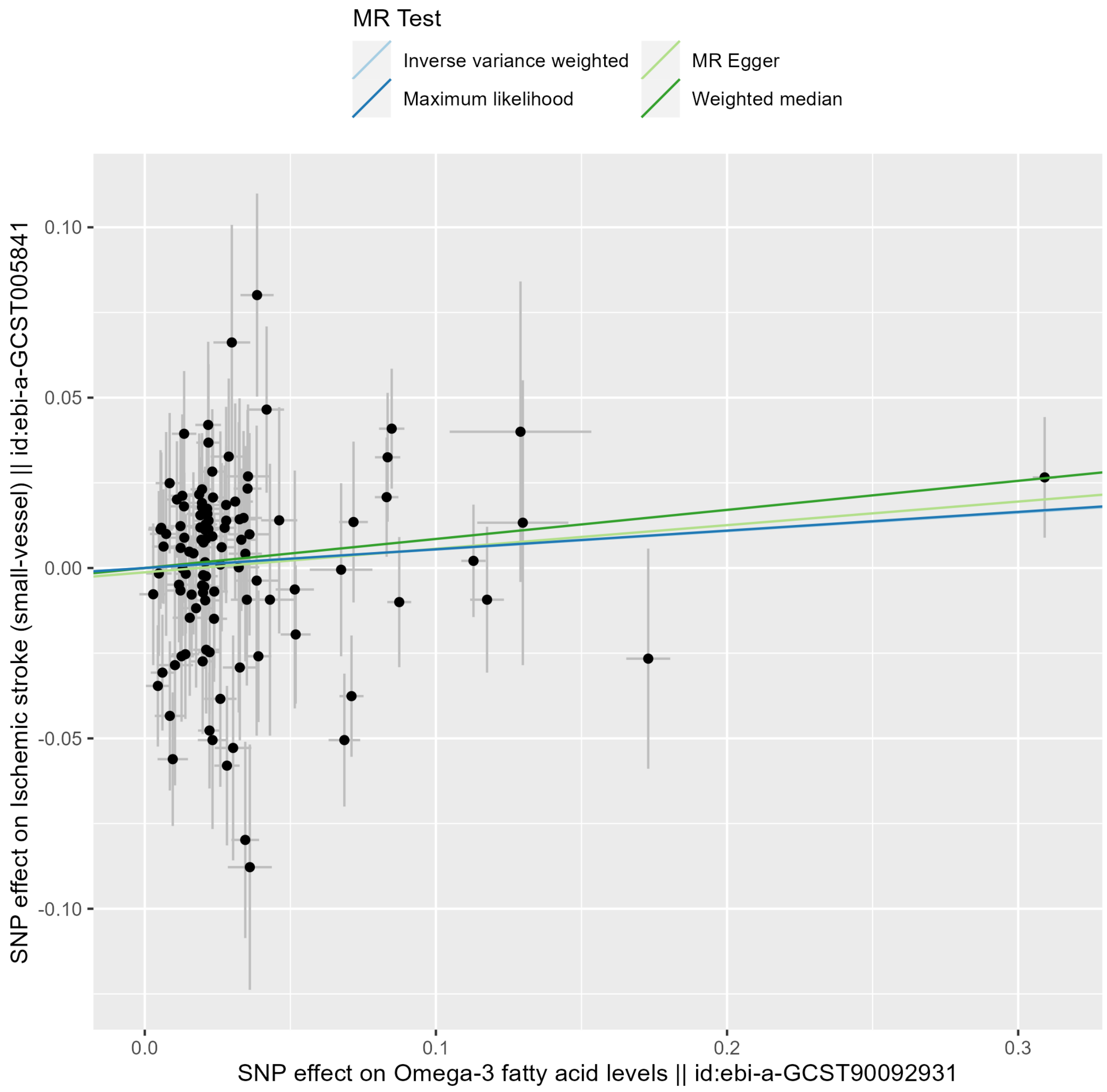

MR Test
Inverse variance weighted
Maximum likelihood
MR Egger
Weighted median
SNP effect on Ischemic stroke (small-vessel) || id:ebi-a-GCST005841
0.10
0.05
0.00
-0.05
-0.10
0.0
0.1
0.2
0.3
SNP effect on Omega-3 fatty acid levels || id:ebi-a-GCST90092931

omegas-CES

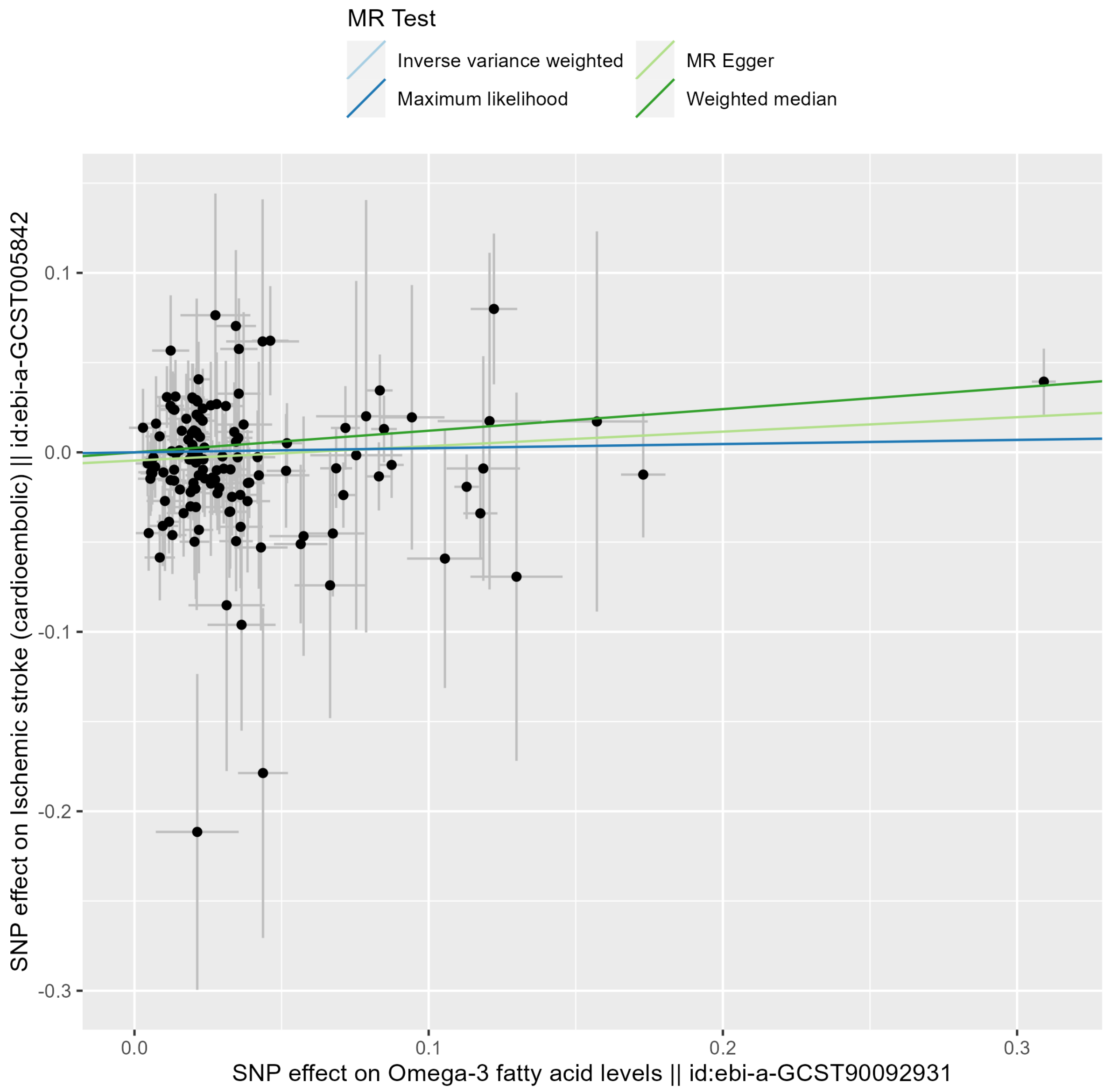
MR Test
Inverse variance weighted
Maximum likelihood
MR Egger
Weighted median
SNP effect on Ischemic stroke (cardioembolic) || id:ebi-a-GCST005842
0.1
0.0
-0.1
-0.2
-0.3
0.0
0.1
0.2
0.3
SNP effect on Omega-3 fatty acid levels || id:ebi-a-GCST90092931

omegas-IS

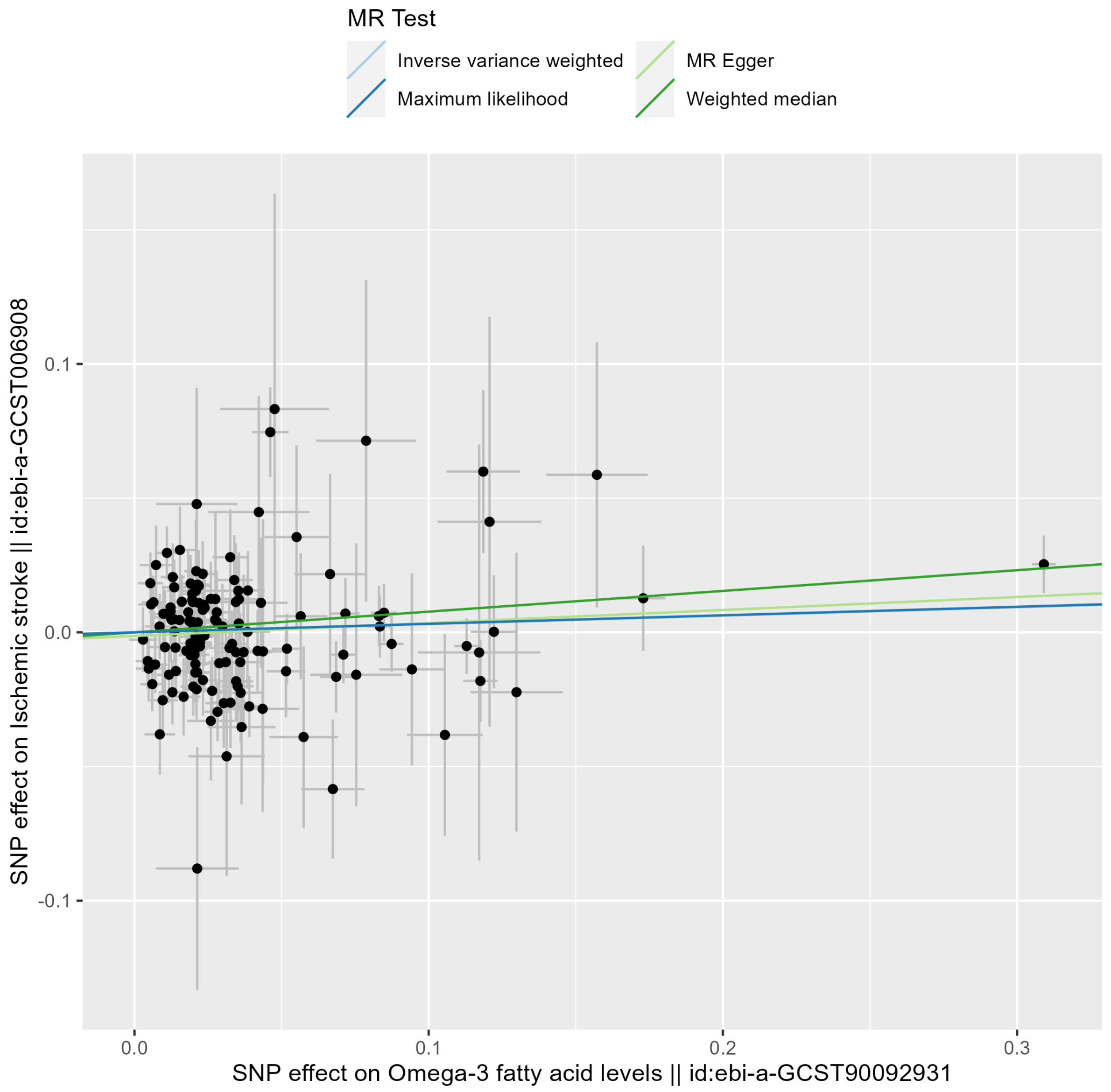
MR Test
Inverse variance weighted
Maximum likelihood
MR Egger
Weighted median
SNP effect on Ischemic stroke || id:ebi-a-GCST006908
0.1
0.0
-0.1
0.0
0.1
0.2
0.3
SNP effect on Omega-3 fatty acid levels || id:ebi-a-GCST90092931

omegas-LS

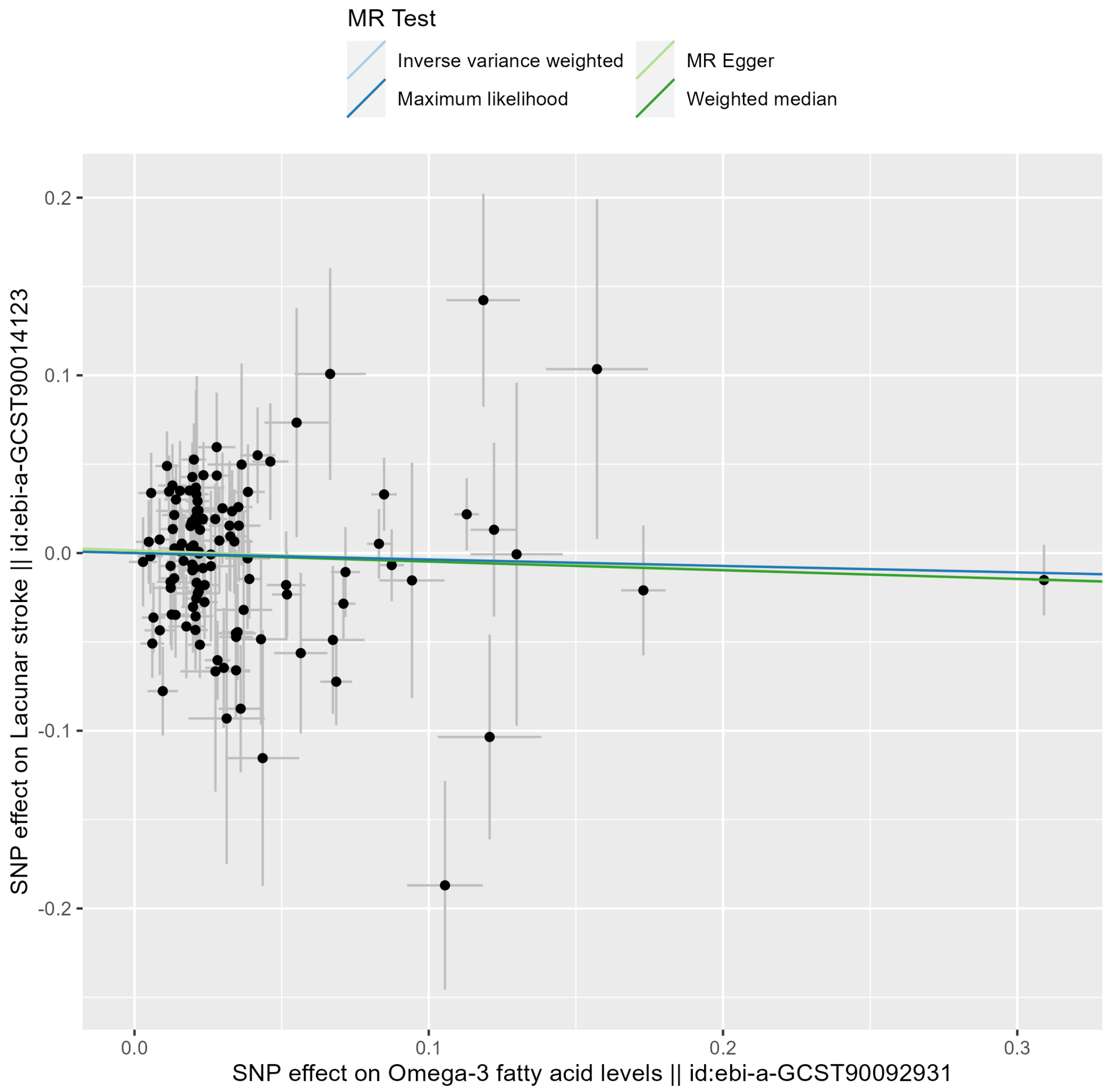
MR Test
Inverse variance weighted
Maximum likelihood
MR Egger
Weighted median
SNP effect on Lacunar stroke || id:ebi-a-GCST90014123
SNP effect on Omega-3 fatty acid levels || id:ebi-a-GCST90092931
0.2
0.1
0.0
-0.1
-0.2
0.0
0.1
0.2
0.3

omegas-LAS

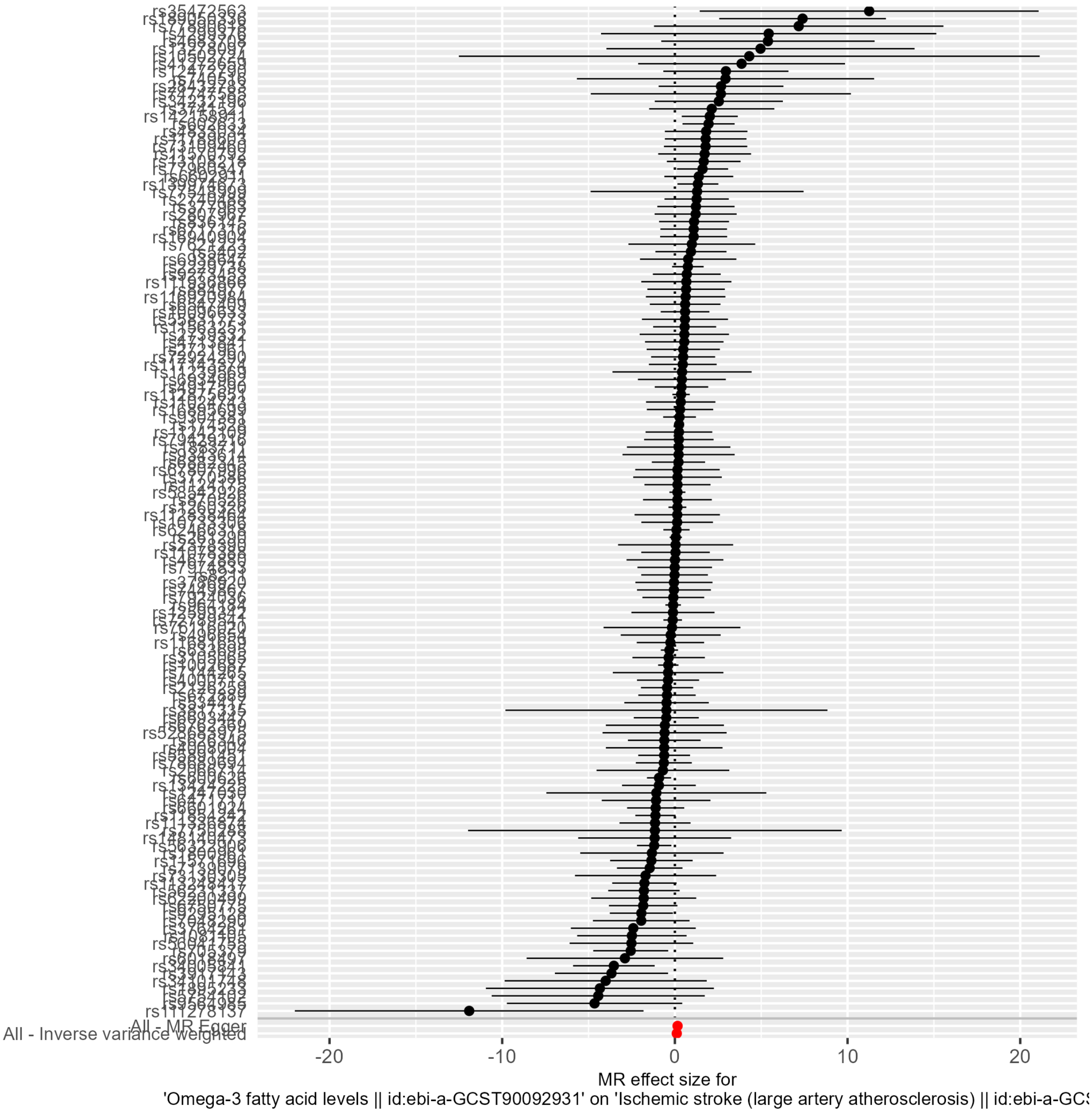
rs35472563
rs111278137
All - MR Egger
All - Inverse variance weighted
-20
-10
0
10
20
MR effect size for
'Omega-3 fatty acid levels || id:ebi-a-GCST90092931' on 'Ischemic stroke (large artery atherosclerosis) || id:ebi-a-GC

omegas-SVS

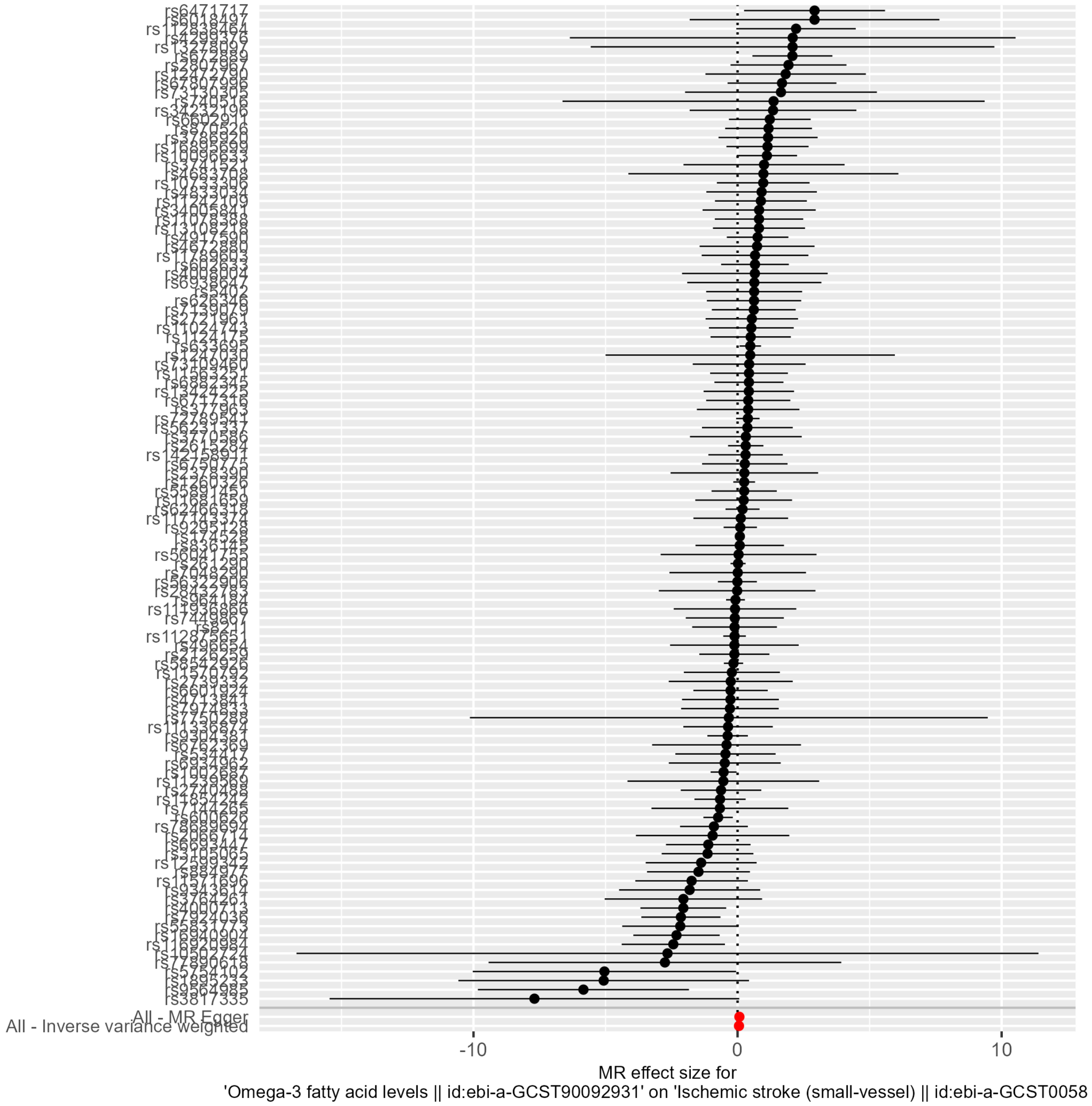
All - MR Egger
All - Inverse variance weighted
-10
0
10
MR effect size for
'Omega-3 fatty acid levels || id:ebi-a-GCST90092931' on 'Ischemic stroke (small-vessel) || id:ebi-a-GCST0058

omegas-CES

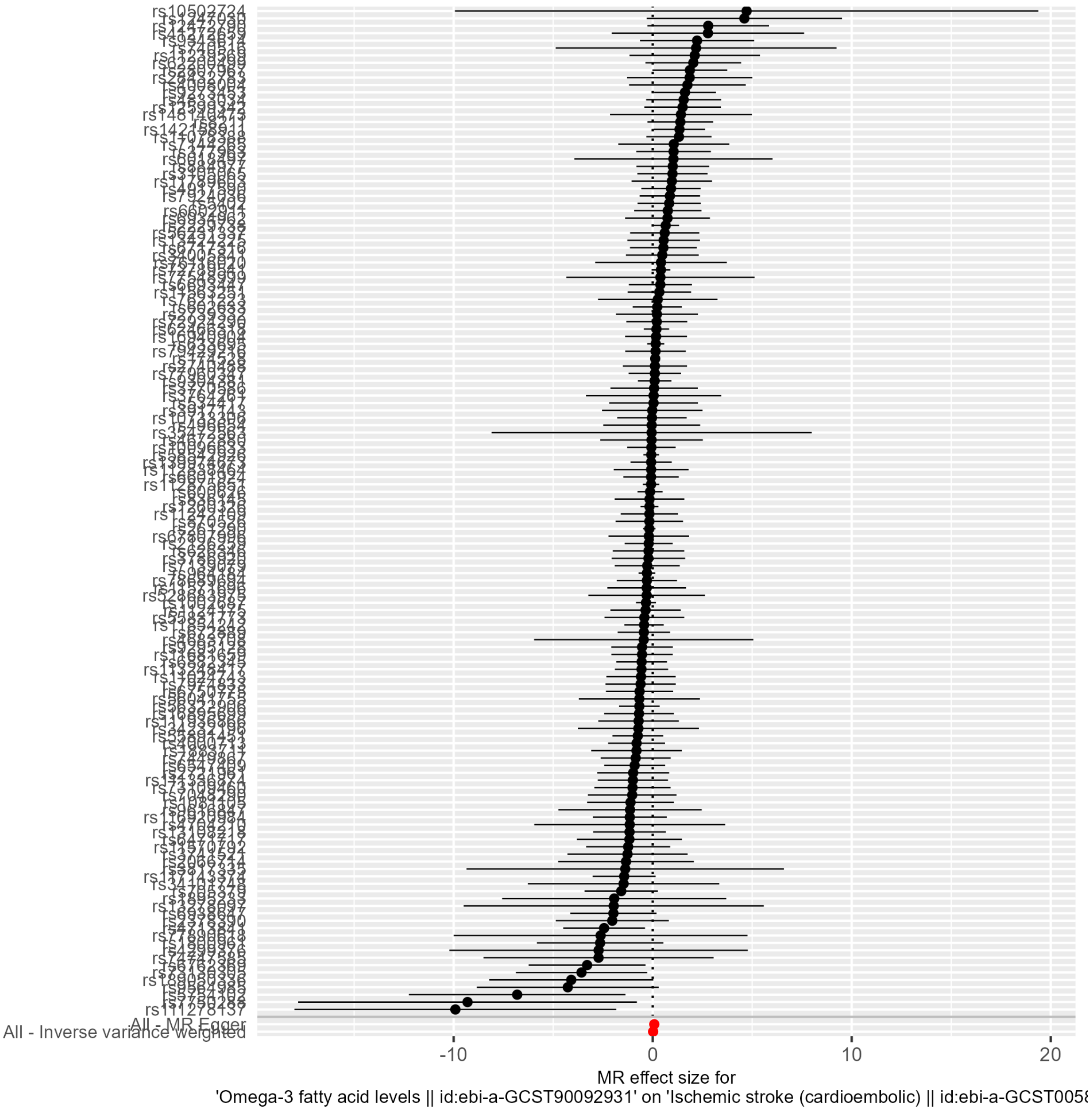
rs10502724
rs111278137
All - MR Egger
All - Inverse variance weighted
-10
0
10
20
MR effect size for
'Omega-3 fatty acid levels || id:ebi-a-GCST90092931' on 'Ischemic stroke (cardioembolic) || id:ebi-a-GCST005

omegas-IS

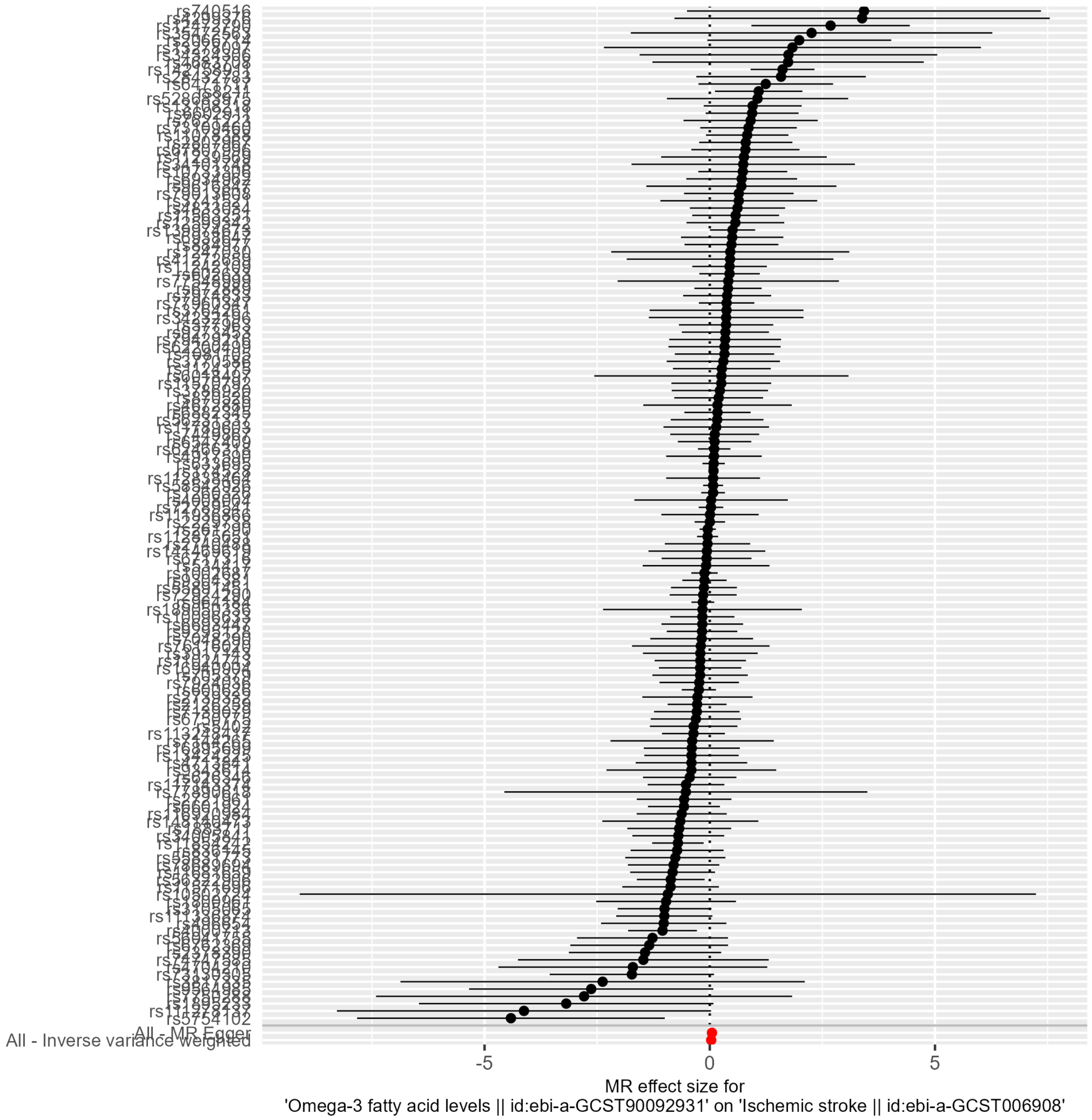
rs740516
rs5754102
All - MR Egger
All - Inverse variance weighted
-5
0
5
MR effect size for
'Omega-3 fatty acid levels || id:ebi-a-GCST90092931' on 'Ischemic stroke || id:ebi-a-GCST006908'

omegas-LS

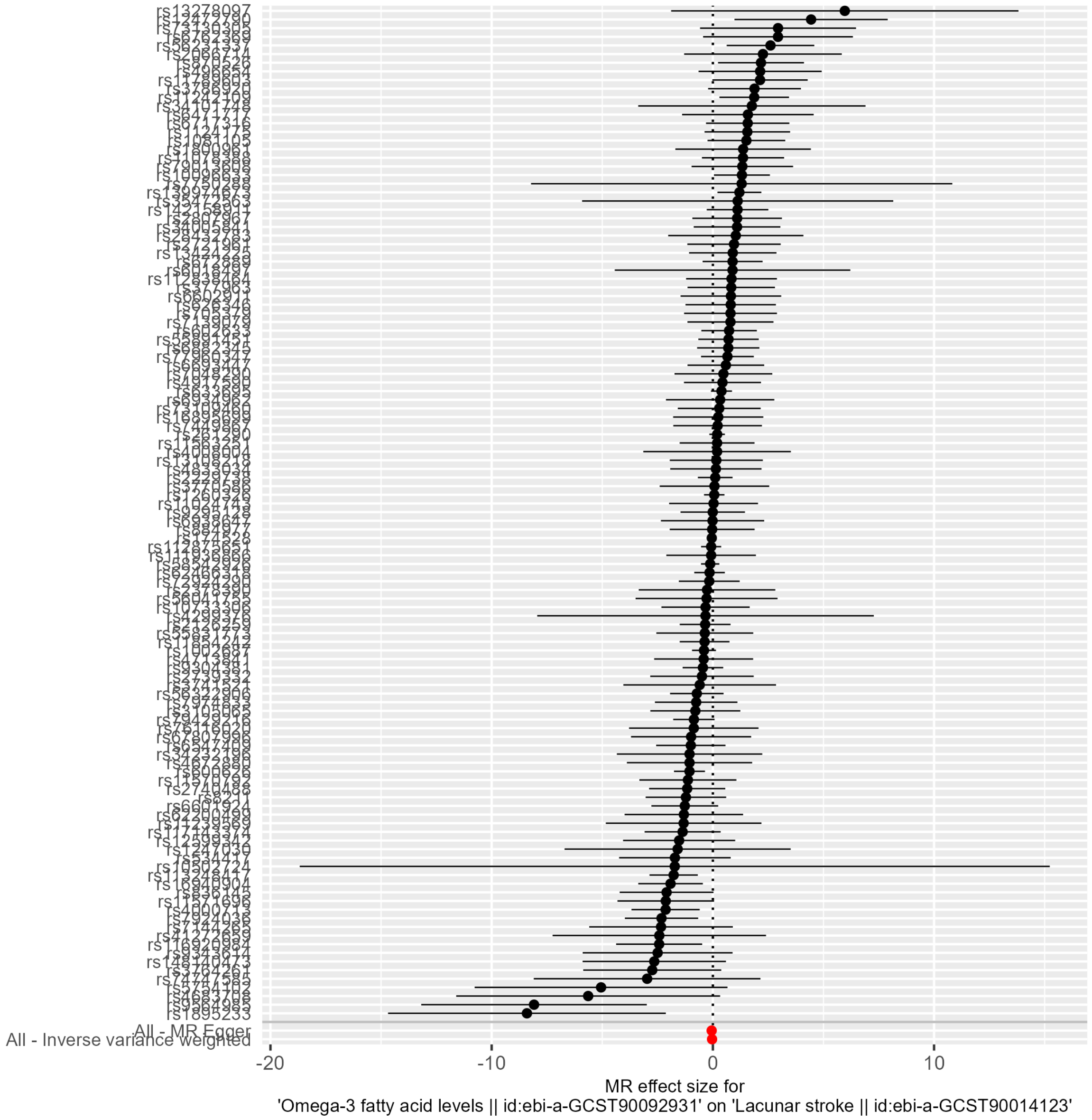
rs13278097
rs1893233
All - MR Egger
All - Inverse variance weighted
-20
-10
0
10
MR effect size for
'Omega-3 fatty acid levels || id:ebi-a-GCST90092931' on 'Lacunar stroke || id:ebi-a-GCST90014123'

omegas-LAS

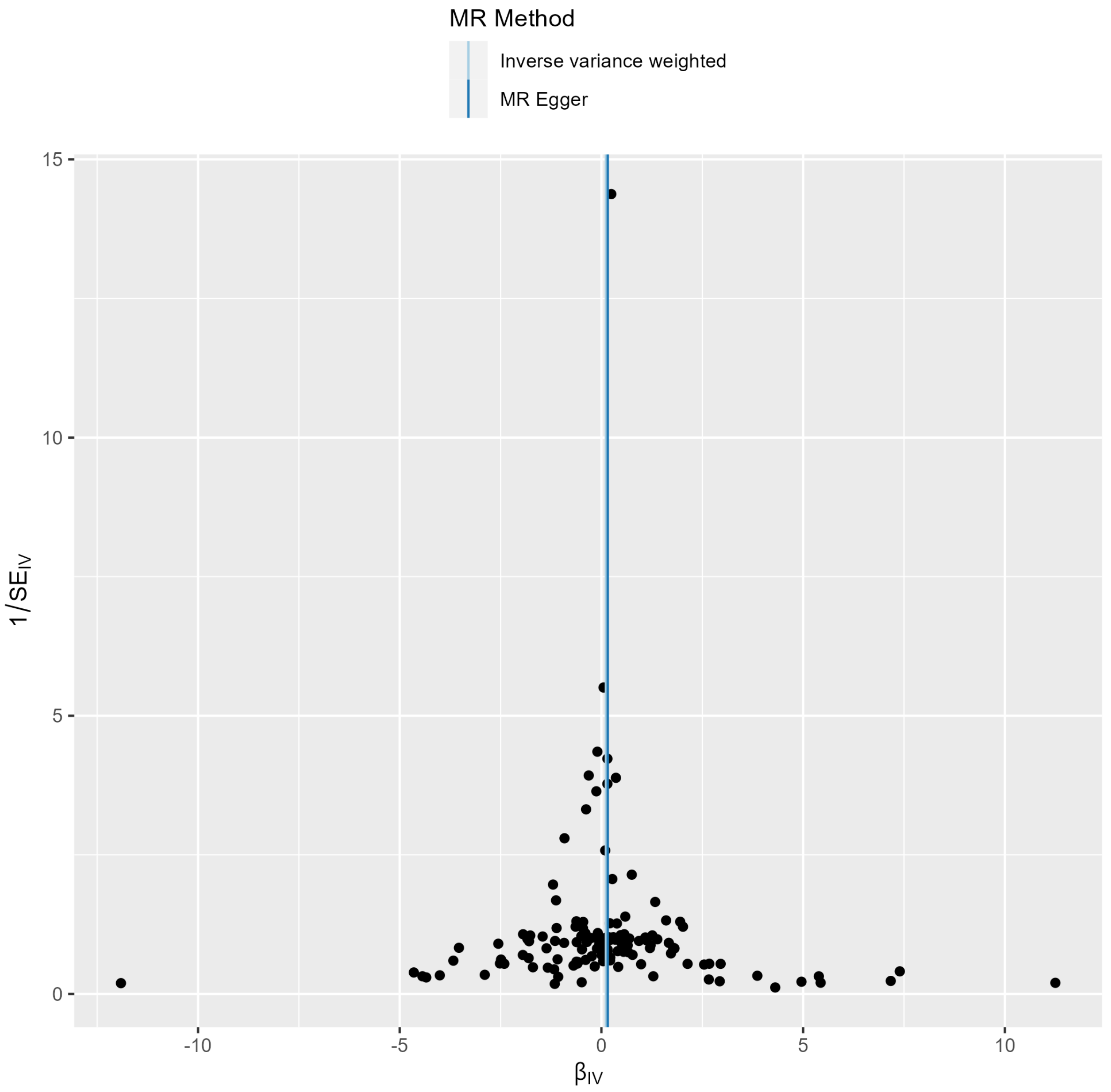

MR Method
Inverse variance weighted
MR Egger
15
10
5
0
-10
-5
0
5
10
1/SE_IV
β_IV

omegas-SVS

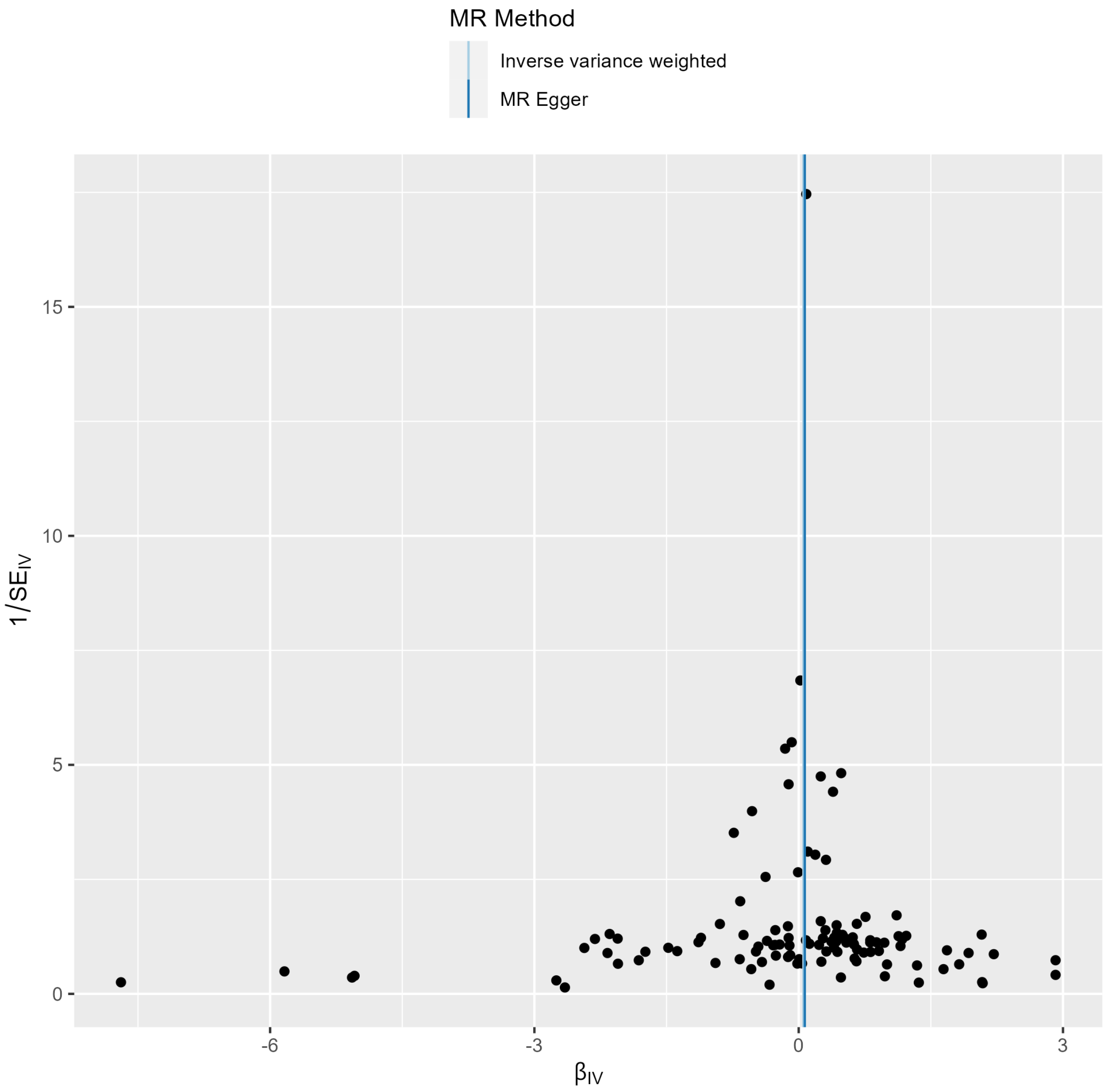
MR Method
Inverse variance weighted
MR Egger
15
10
5
0
1/SE$_{IV}$
-6
-3
0
3
β$_{IV}$

omegas-CES

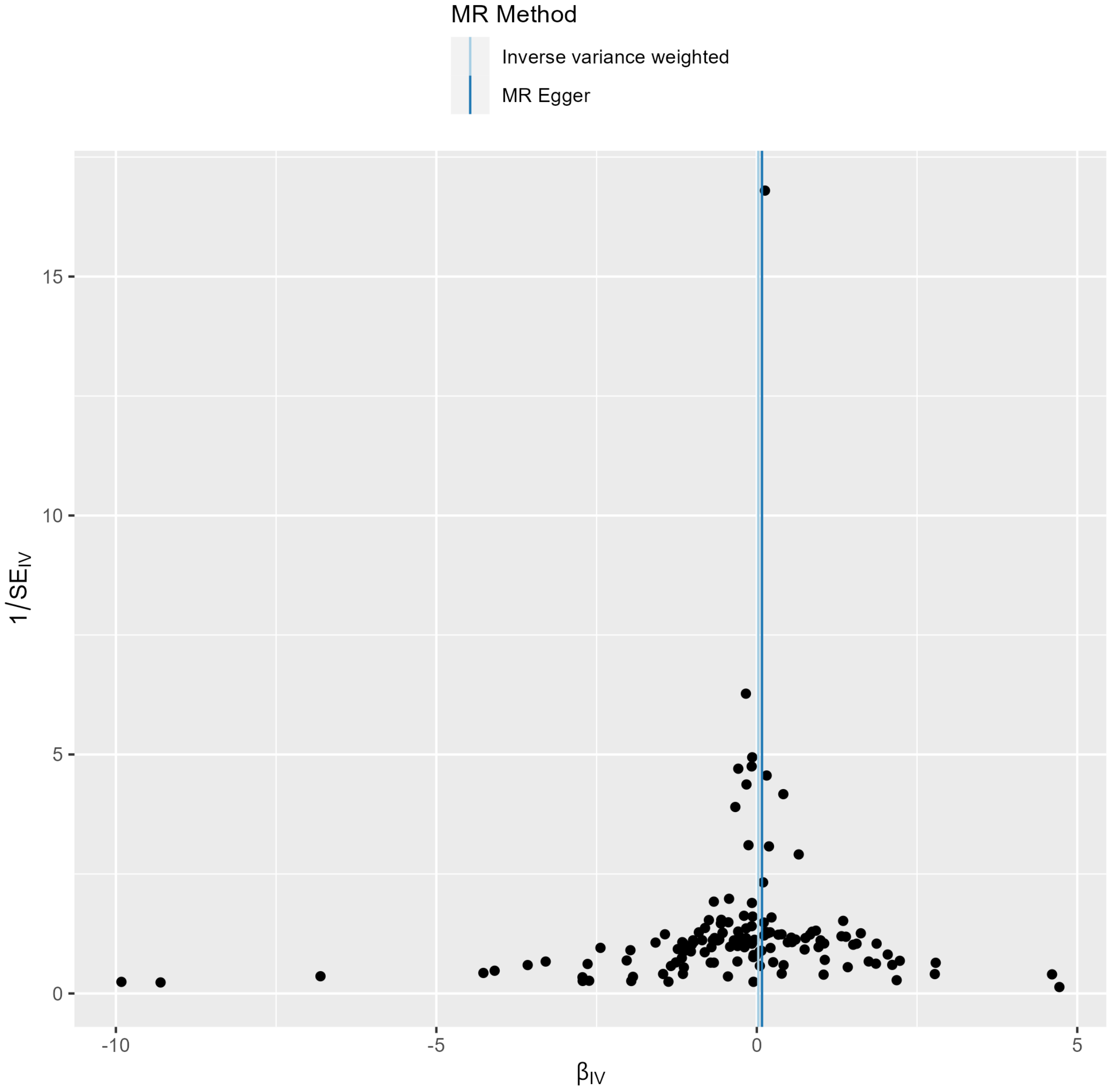

MR Method
Inverse variance weighted
MR Egger
15
10
5
0
1/SE_IV
-10
-5
0
5
β_IV

omegas-IS

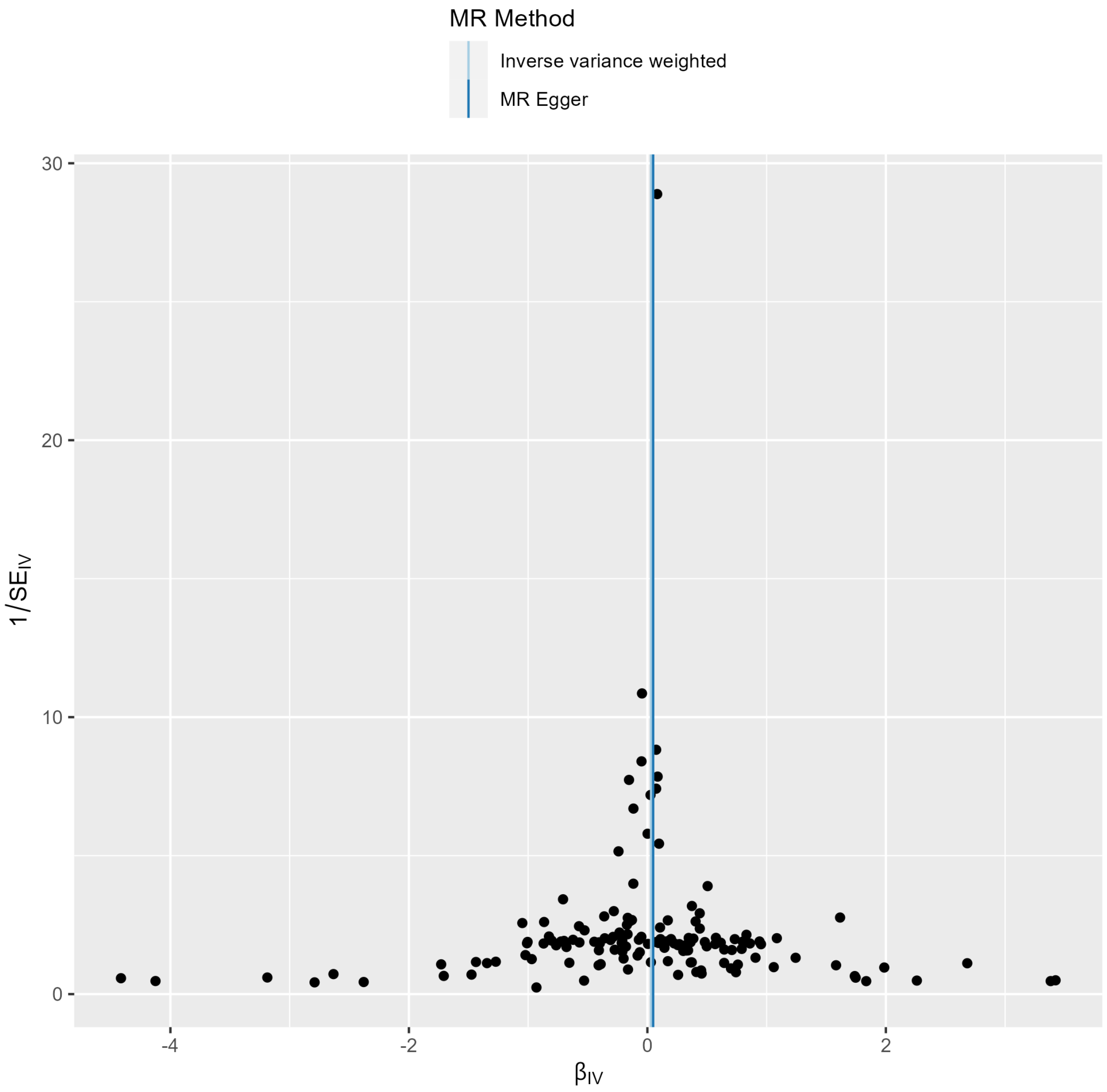
MR Method
Inverse variance weighted
MR Egger
30
20
10
0
$1/SE_{IV}$
-4
-2
0
2
β_{IV}

omegas-LS

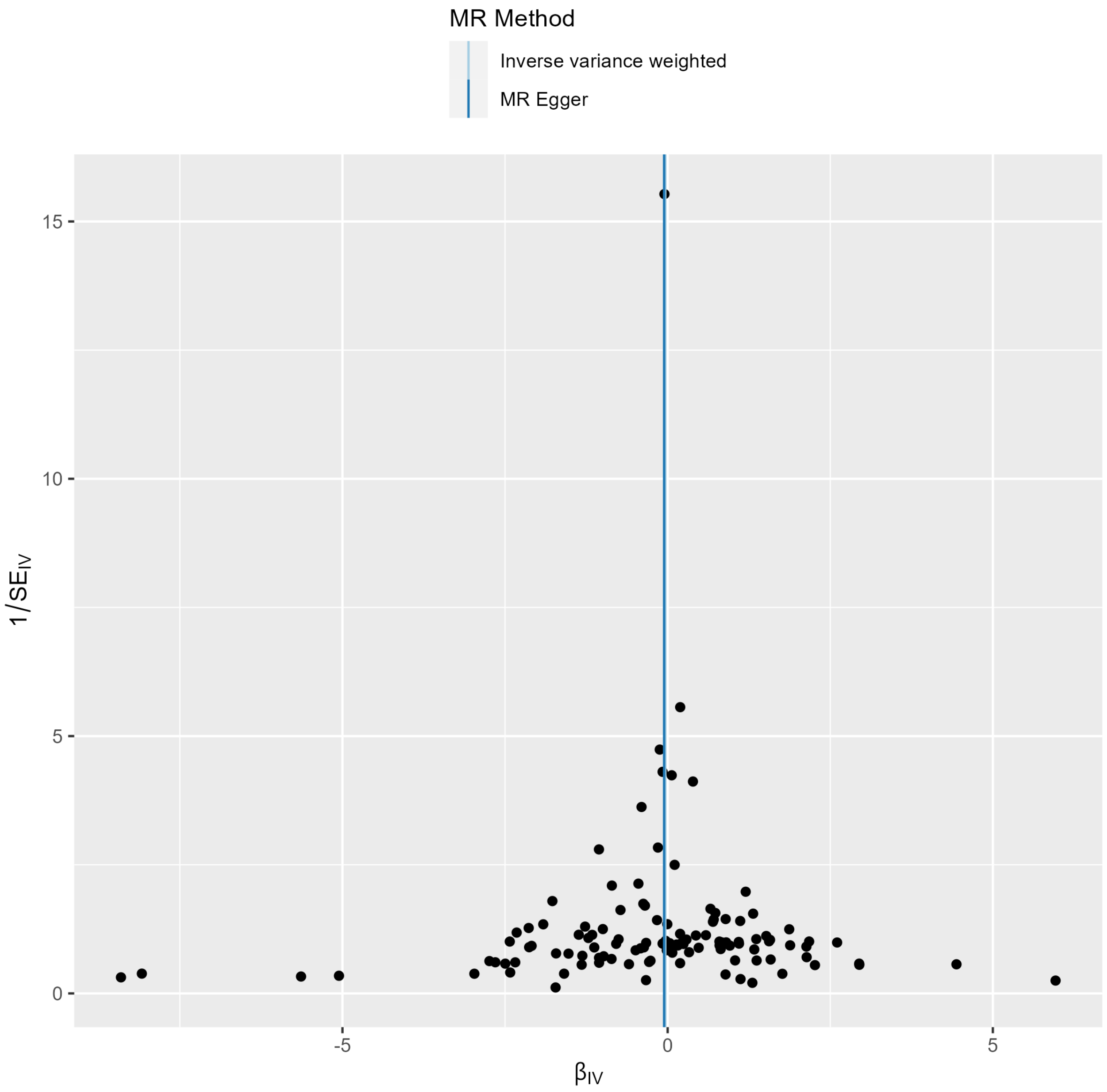

Ω-3-DBP leave-one-out

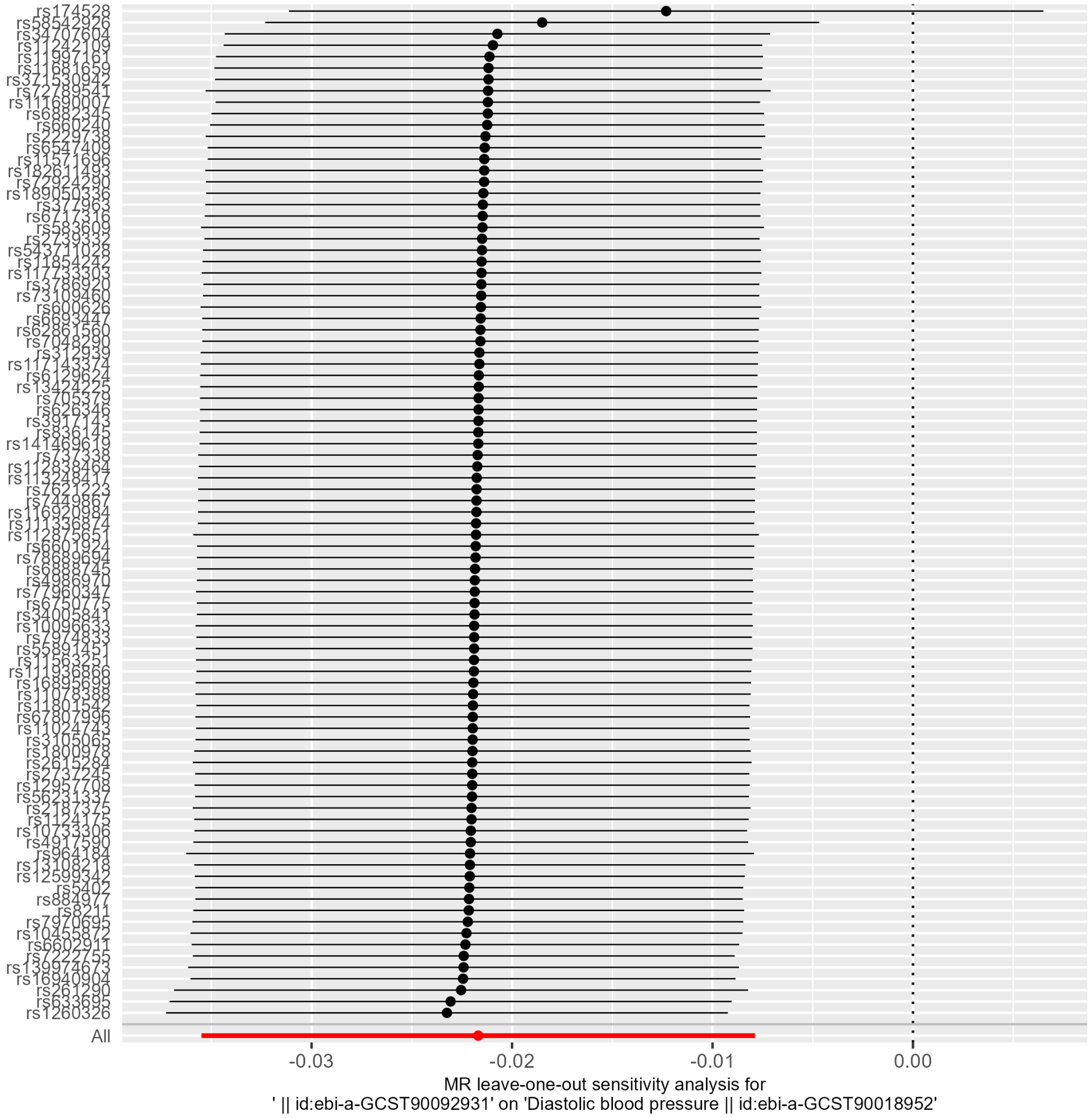

Ω-3-SBP leave-one-out

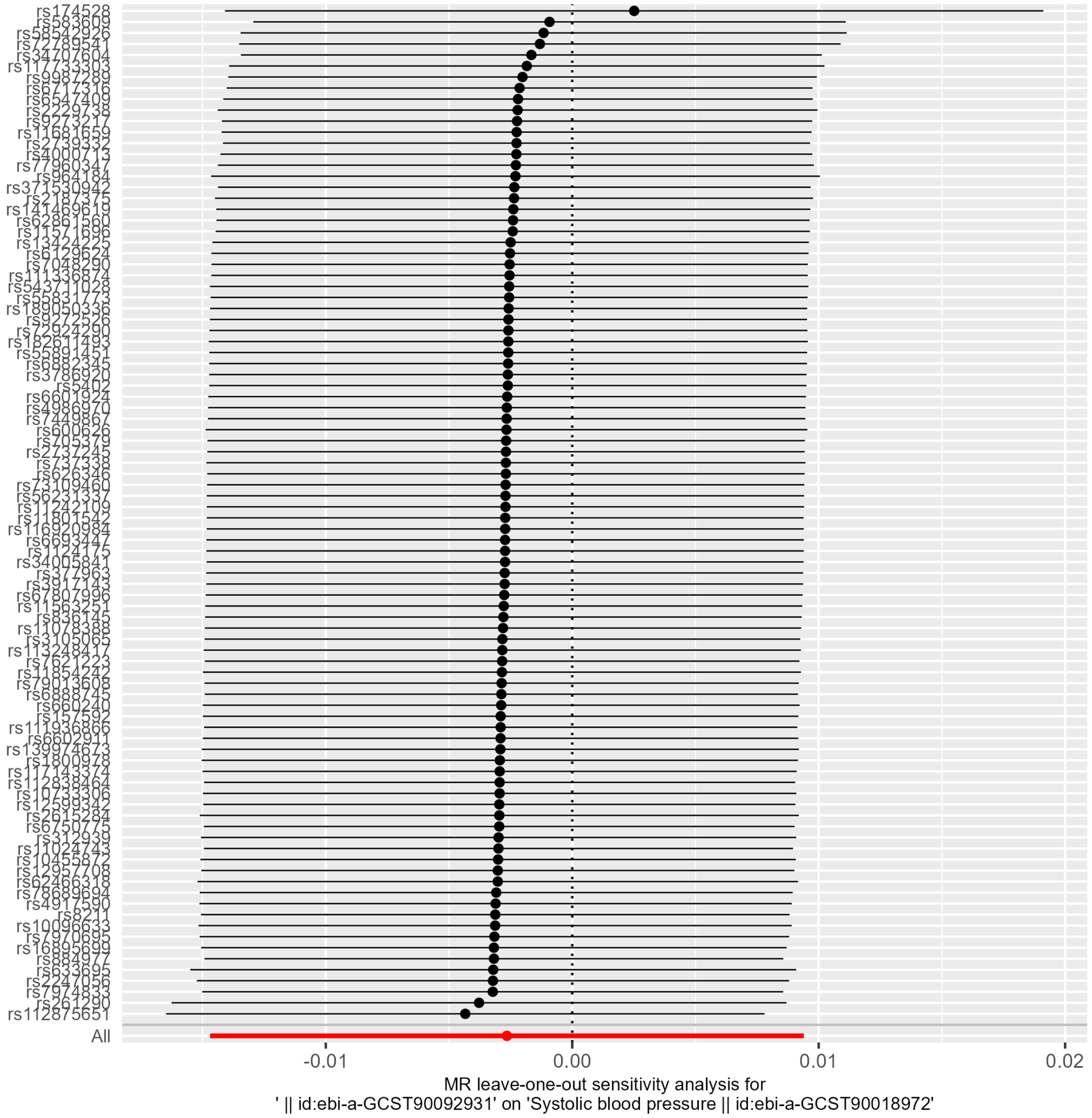

Ω-3-EH leave-one-out

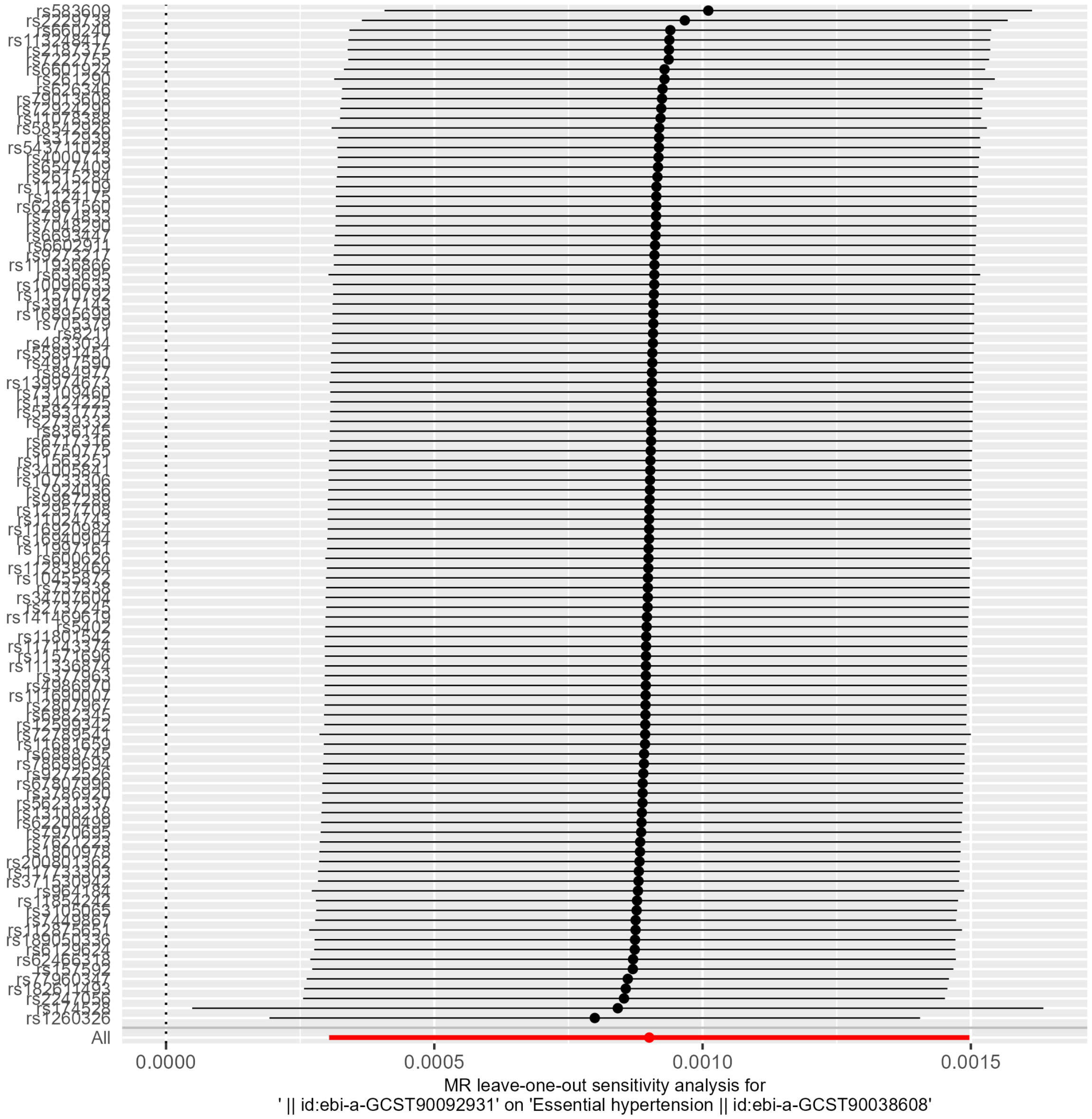

Ω-3-DBP scatter plot

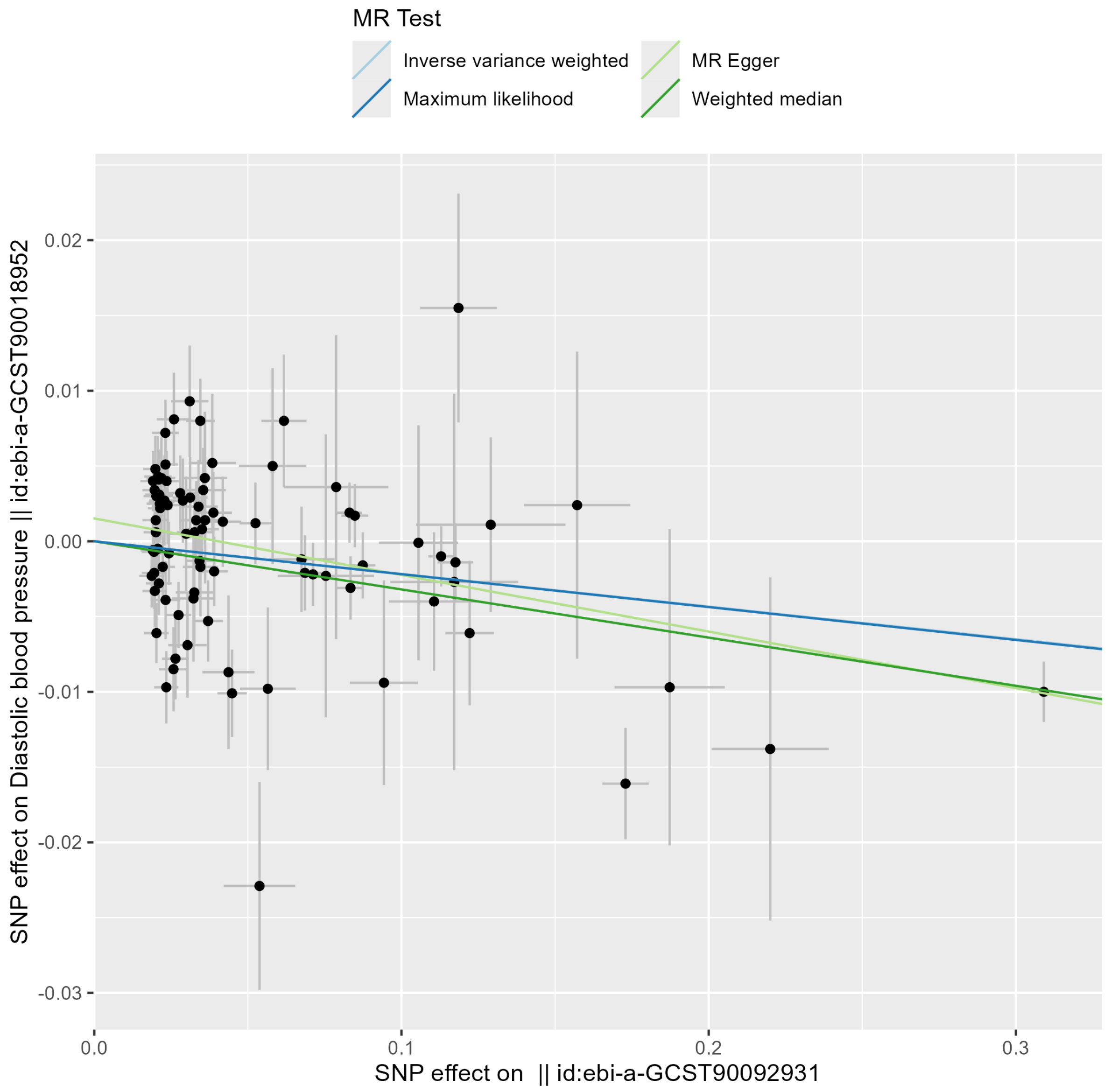

Ω-3-SBP scatter plot

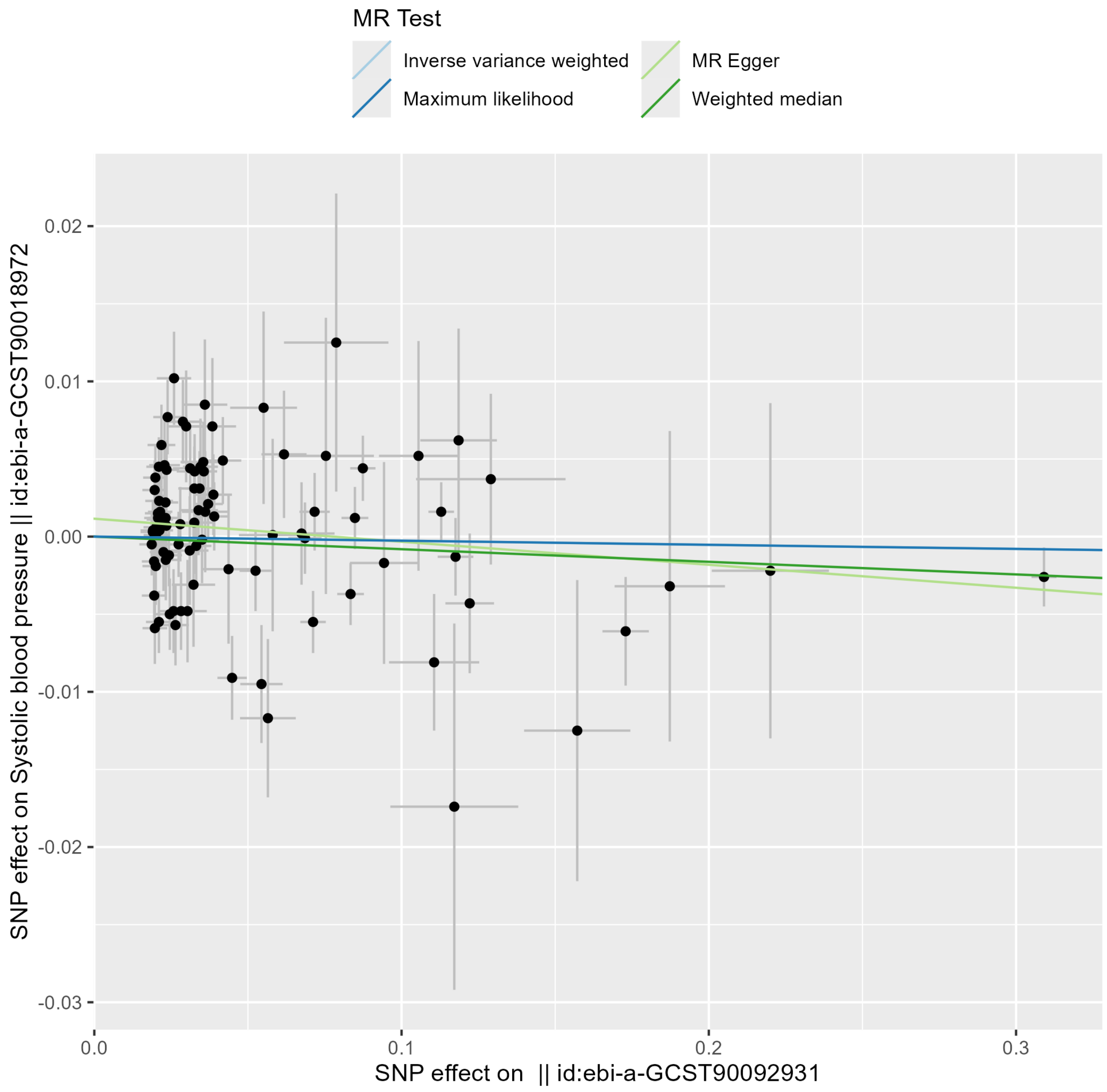

Ω-3-EH scatter plot

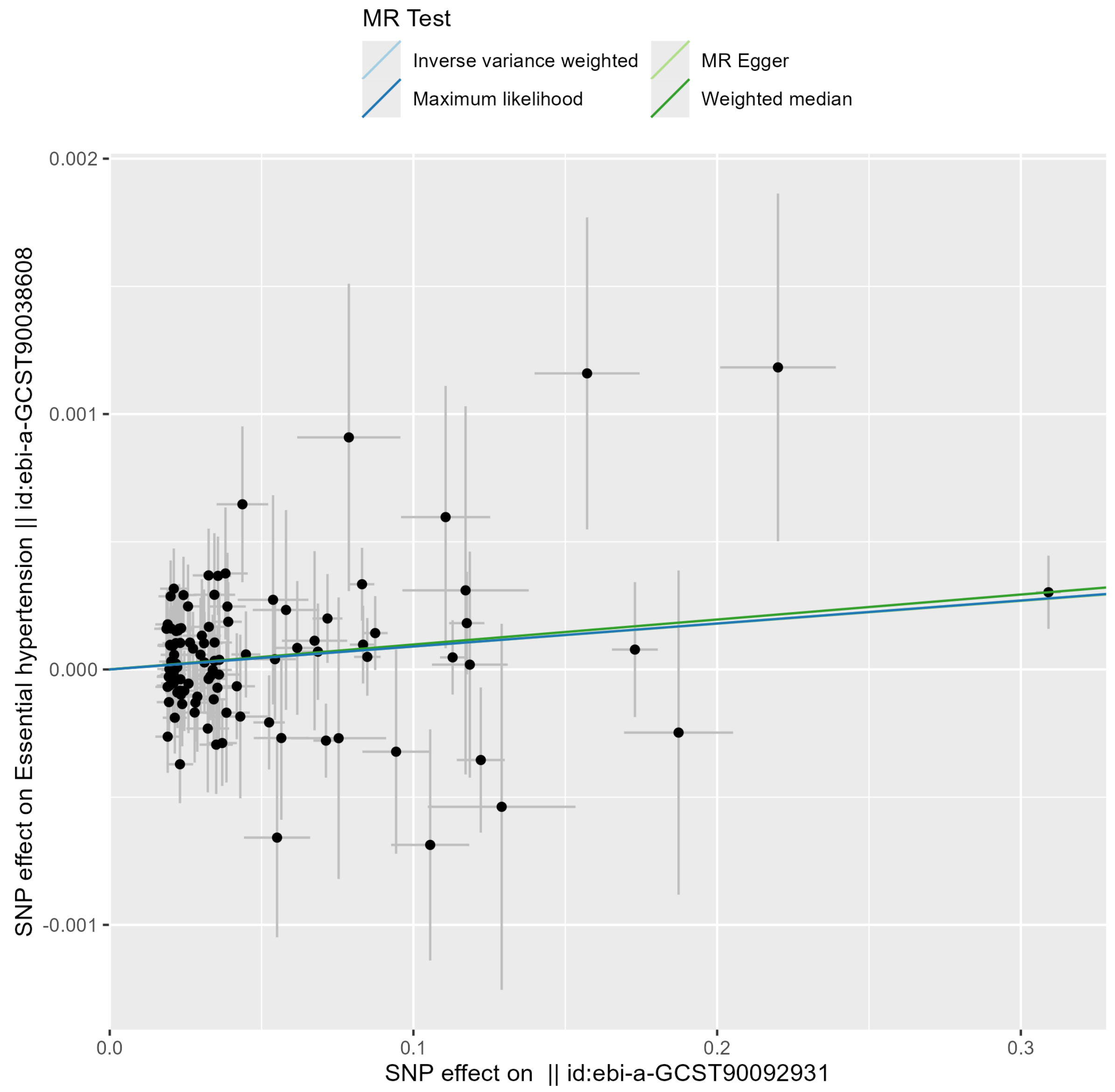

Ω-3-DBP forest plot

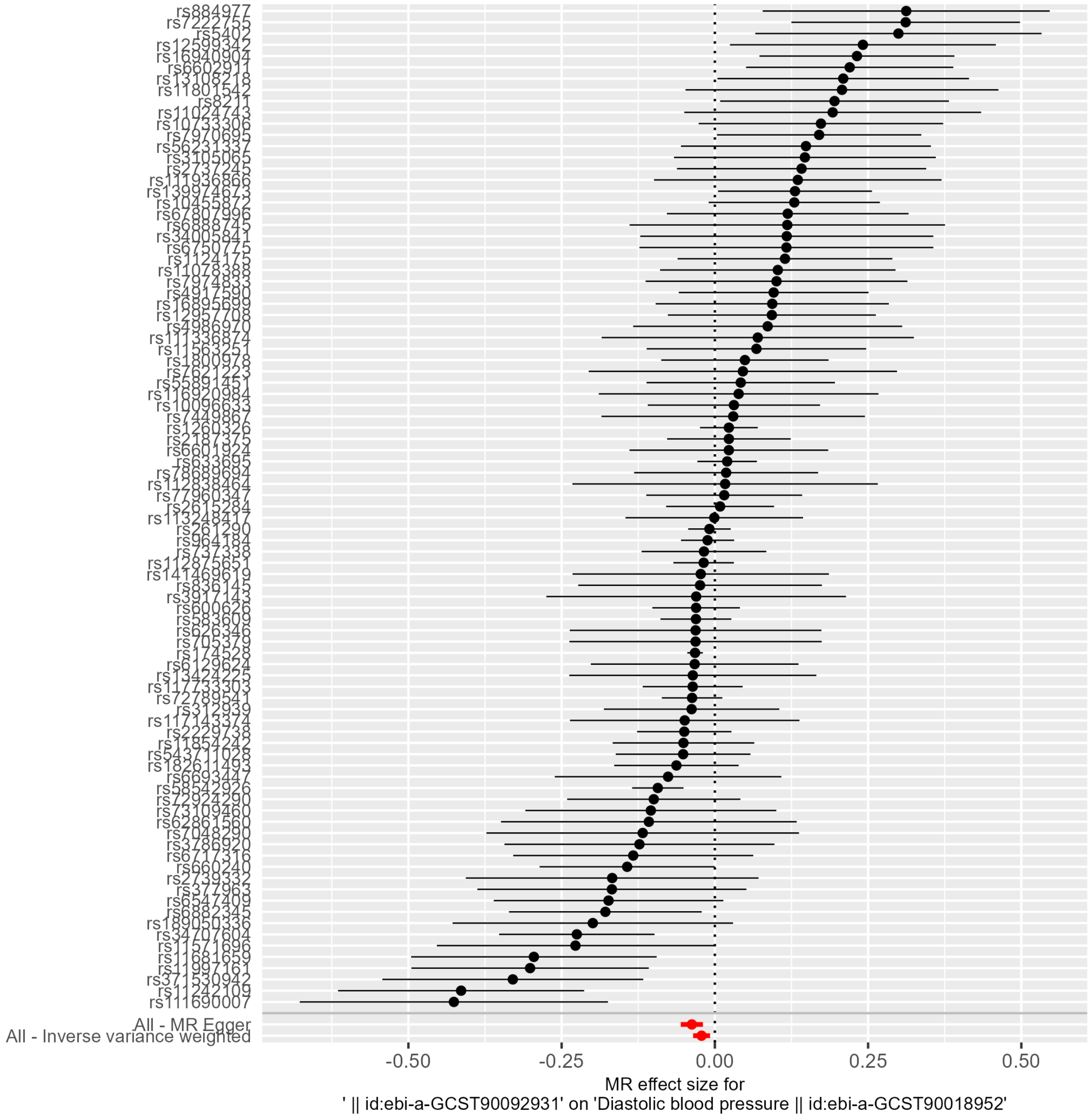

Ω-3-SBP forest plot

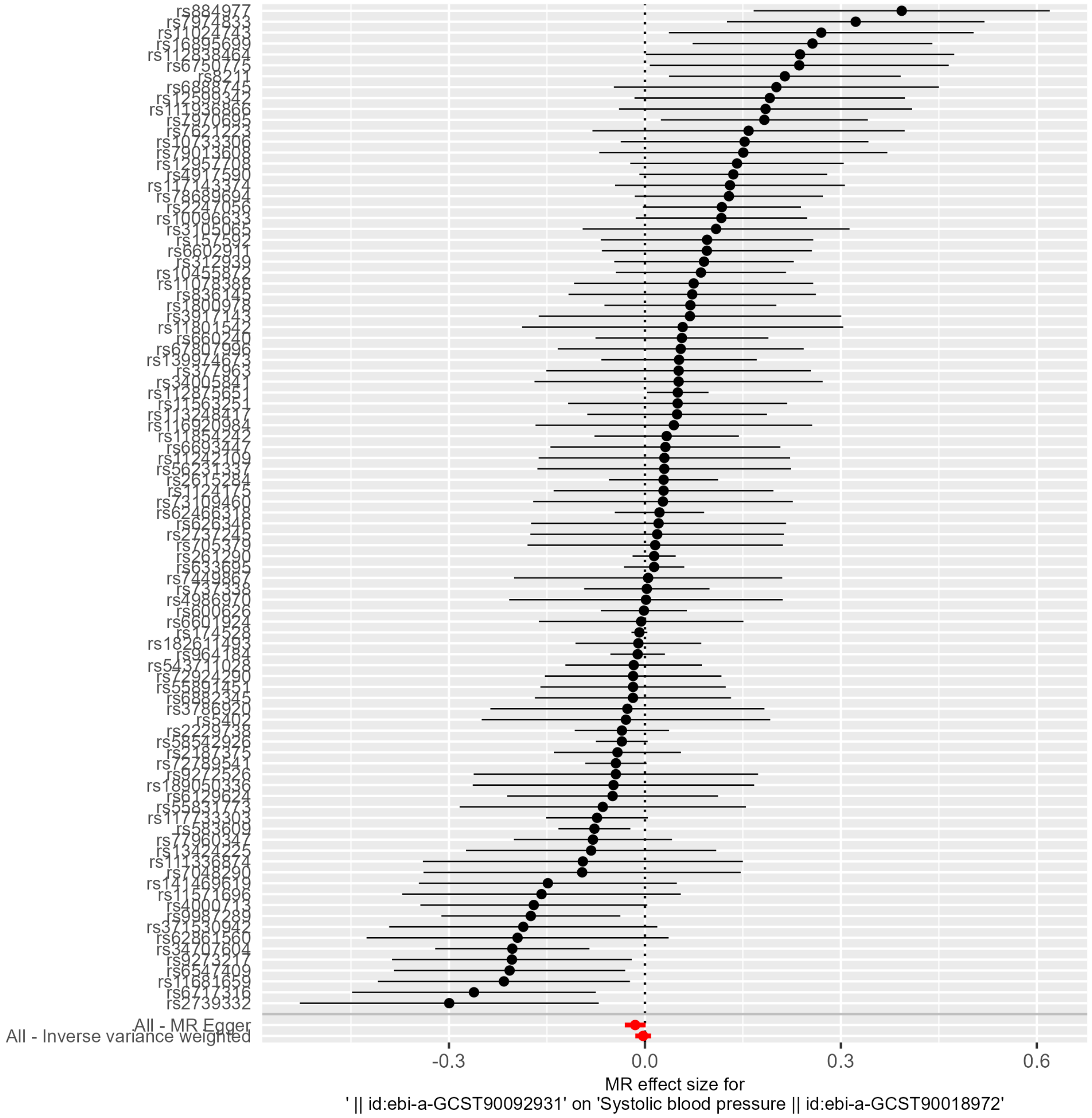

Ω-3-EH forest plot

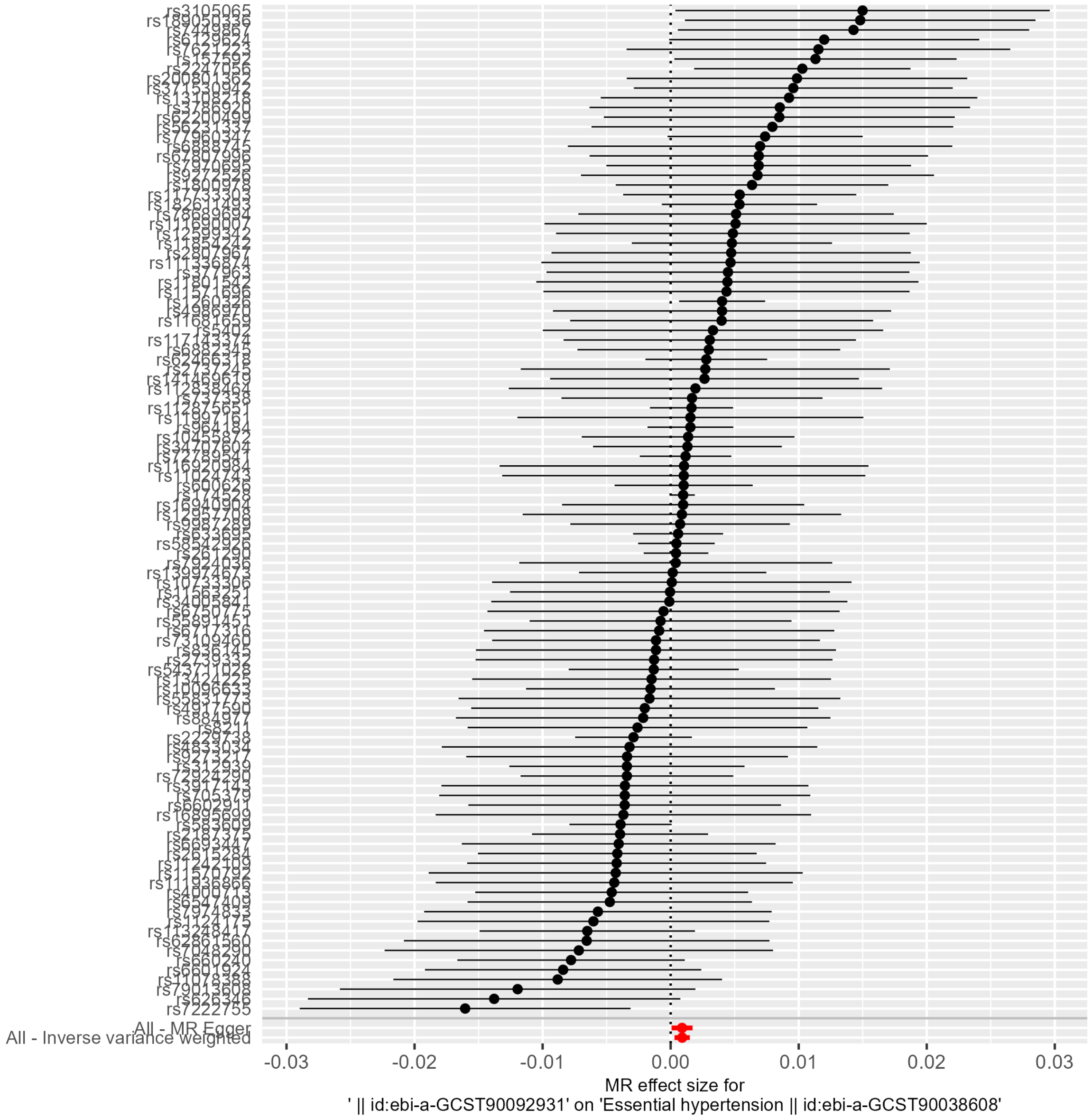

Ω-3-DBP funnel plot

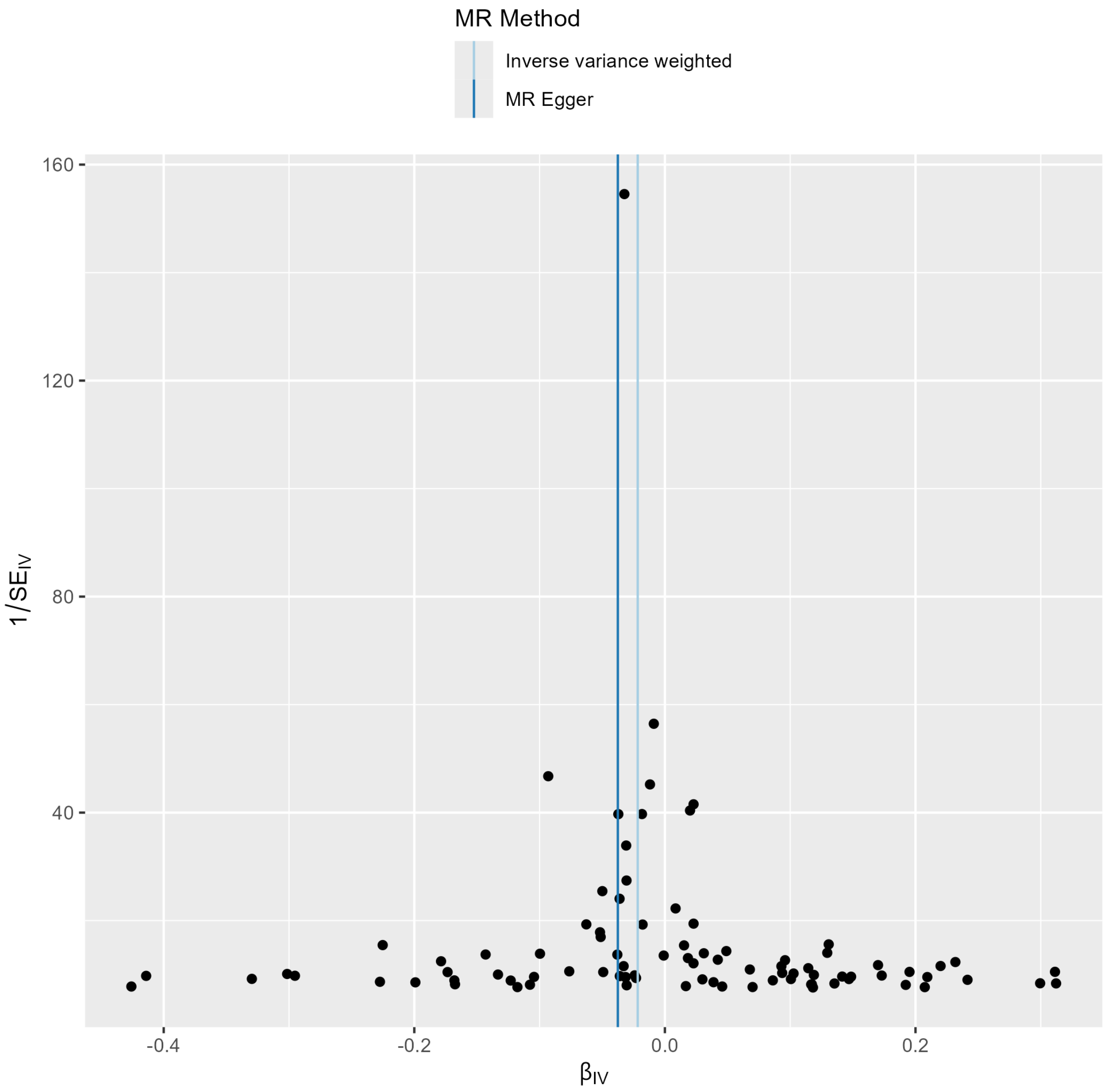

Ω-3-SBP funnel plot

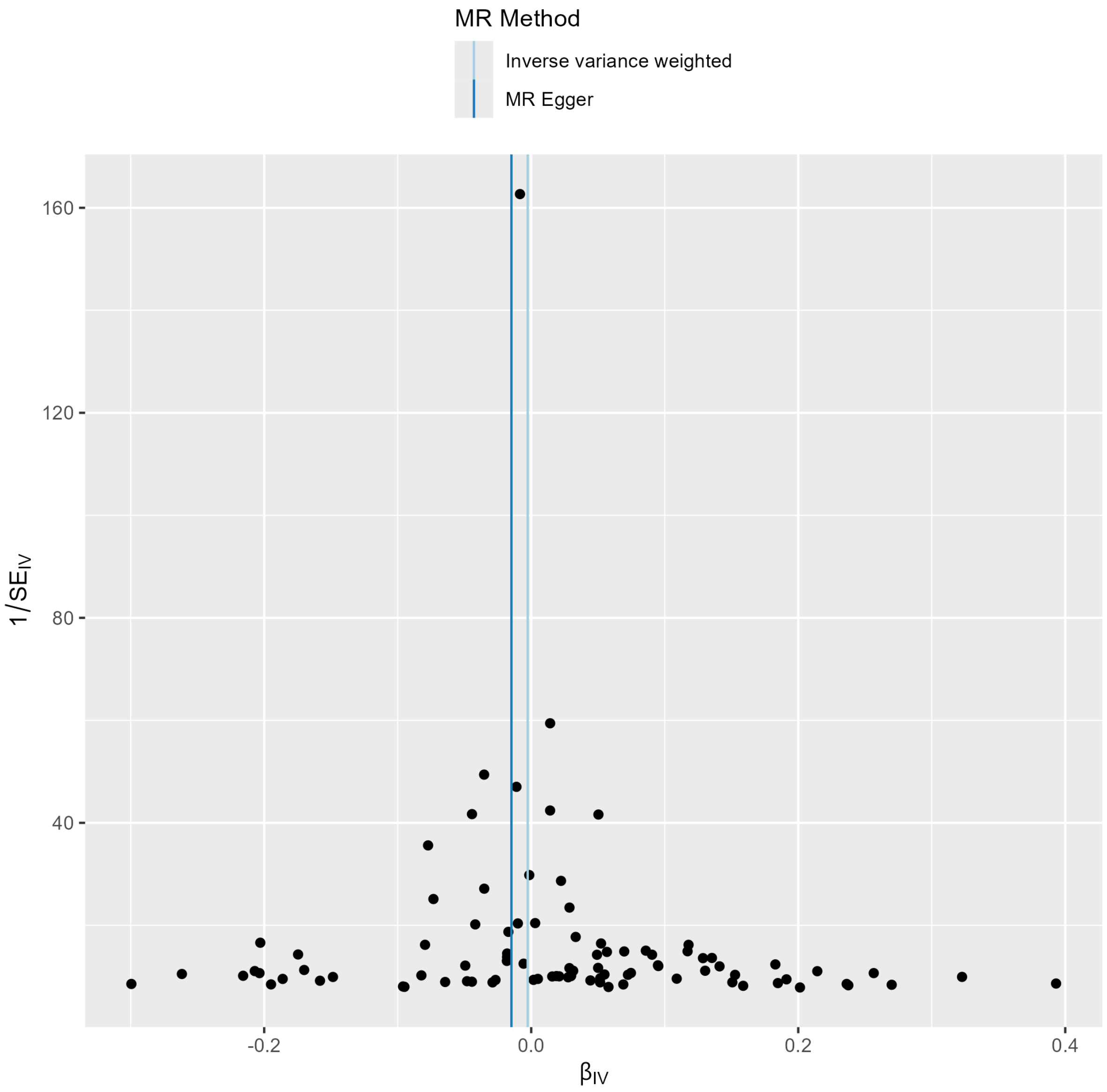

Ω-3-EH funnel plot

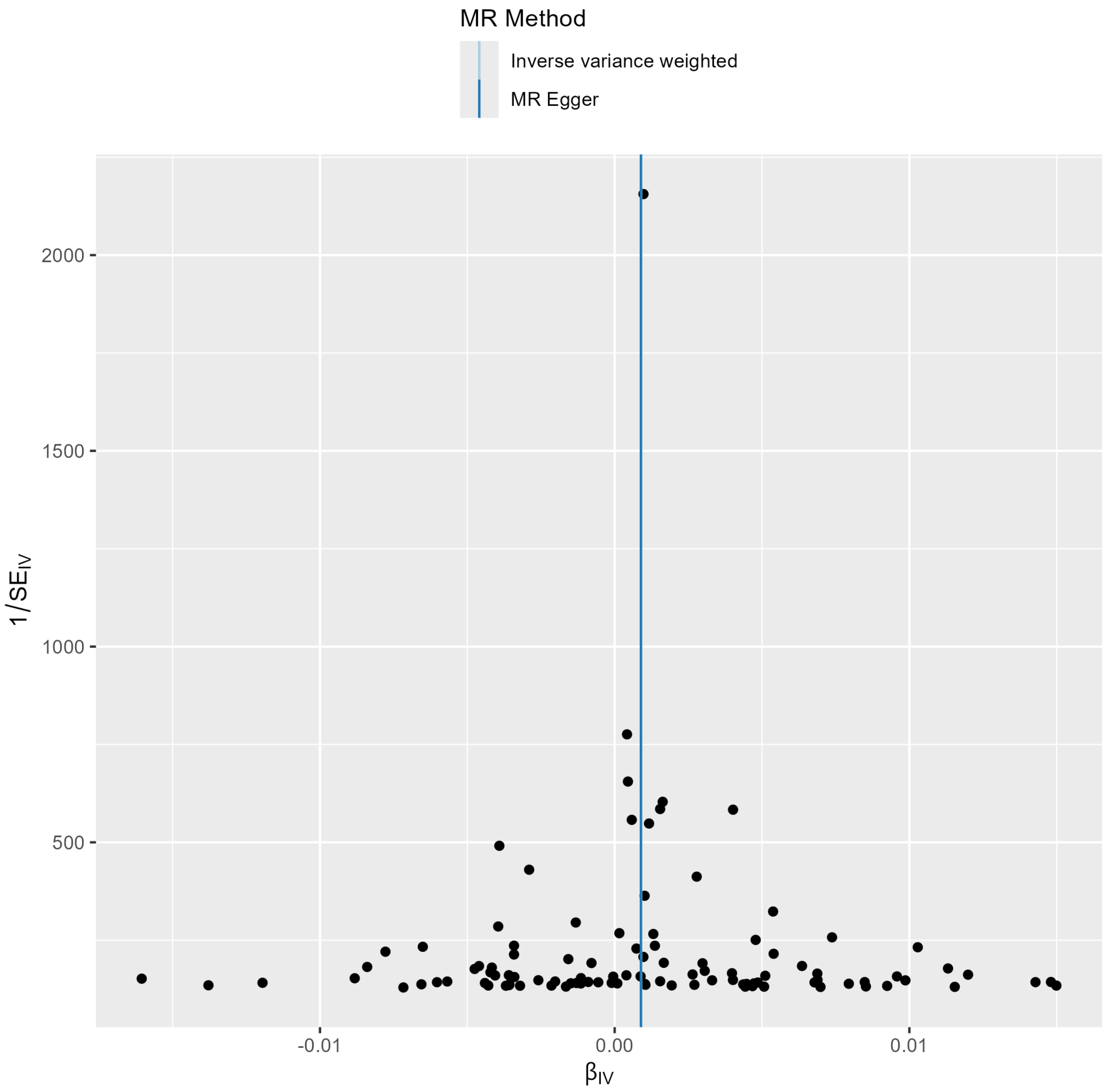

DBP-LAS leave-one-out

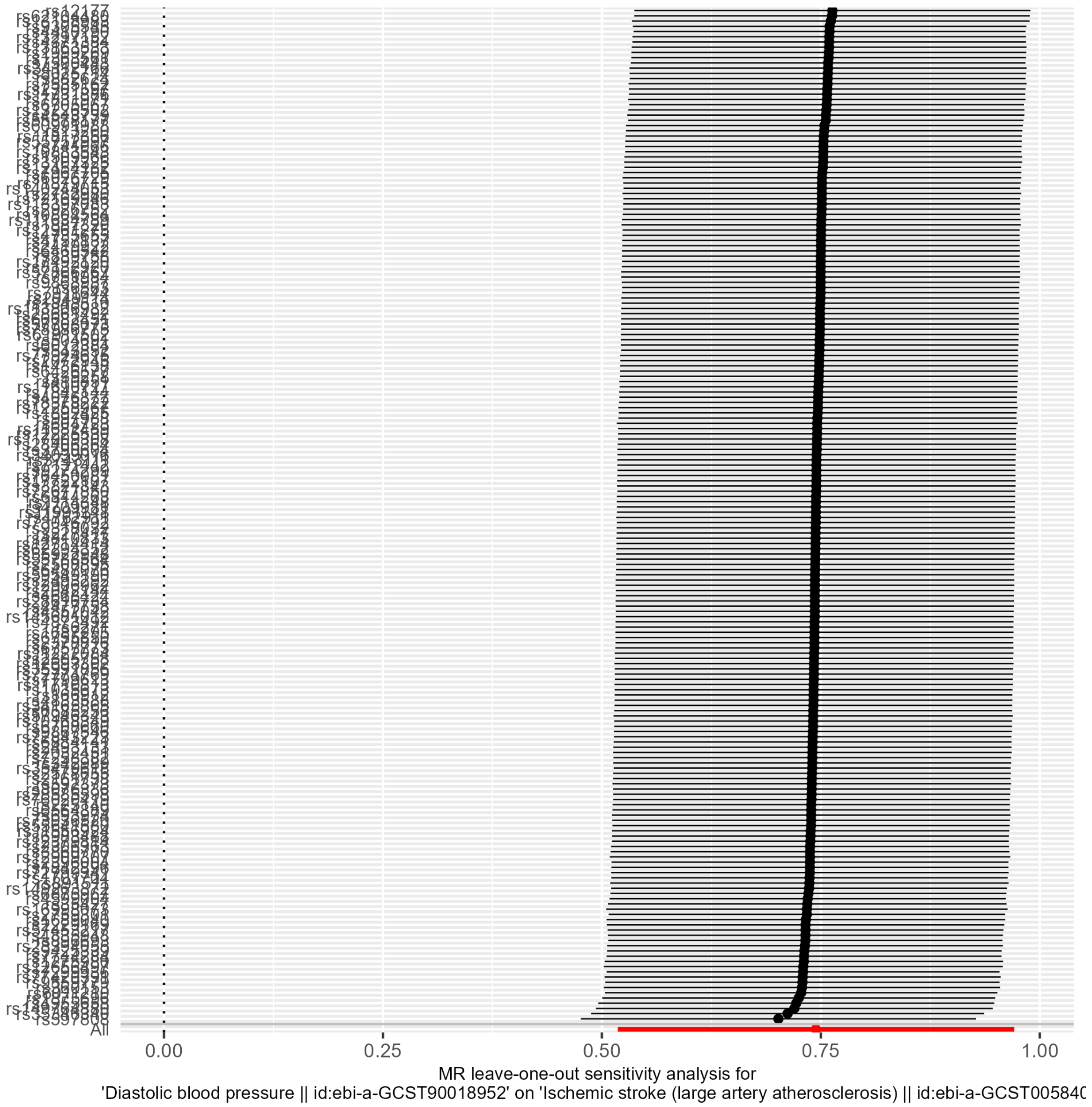

DBP-SVS leave-one-out

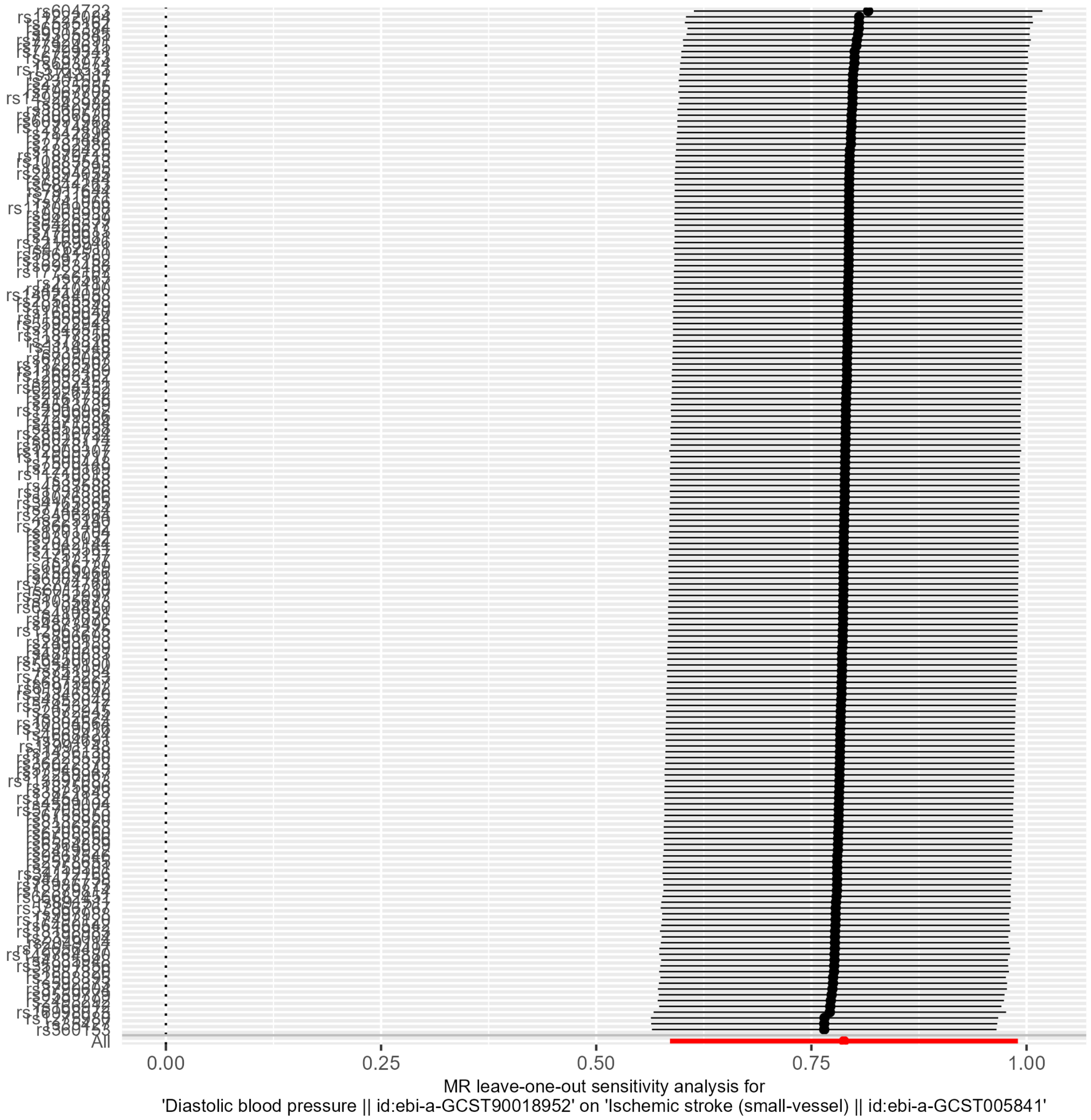

DBP-CES leave-one-out

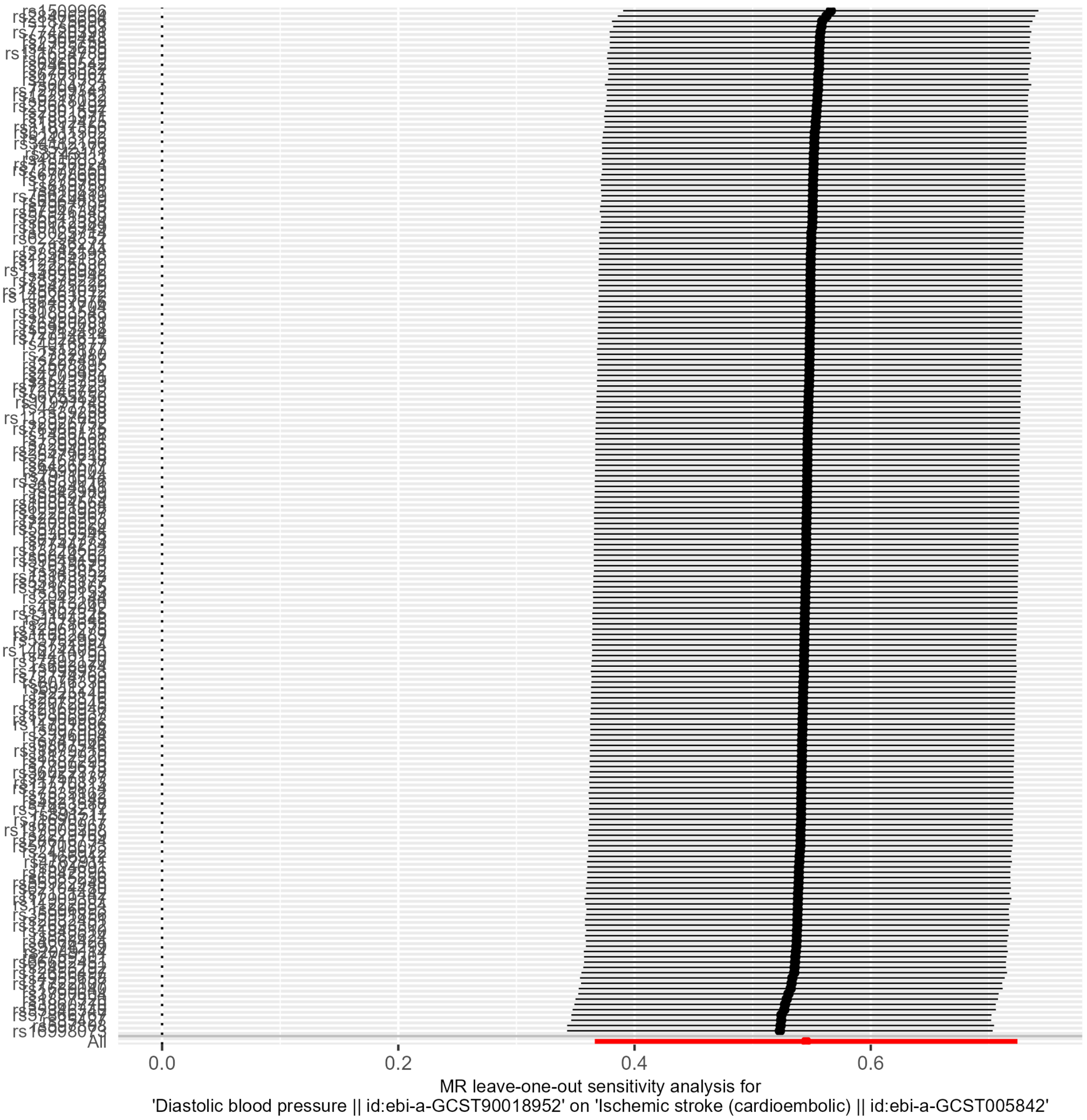

DBP-IS leave-one-out

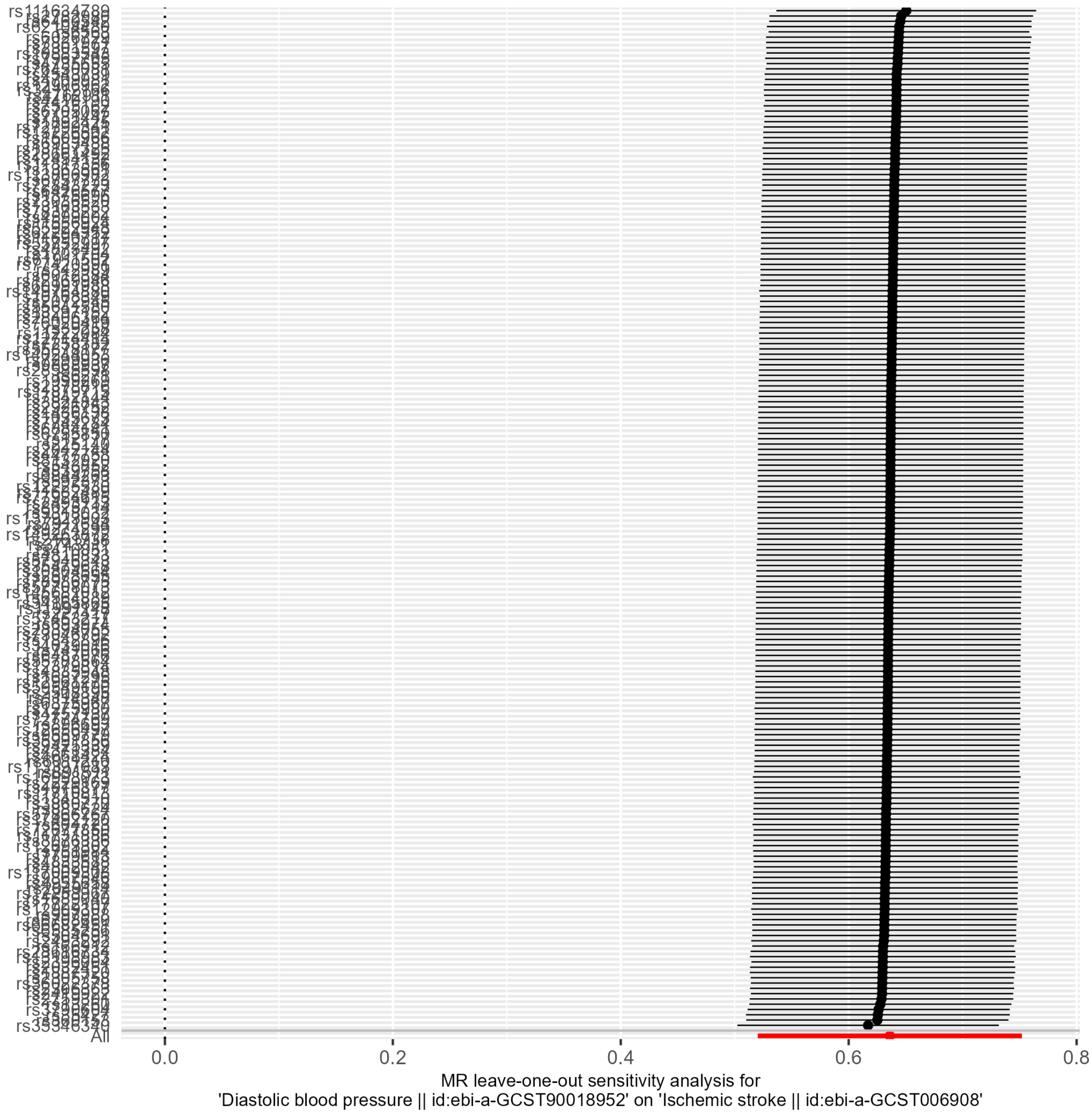

DBP-LS leave-one-out

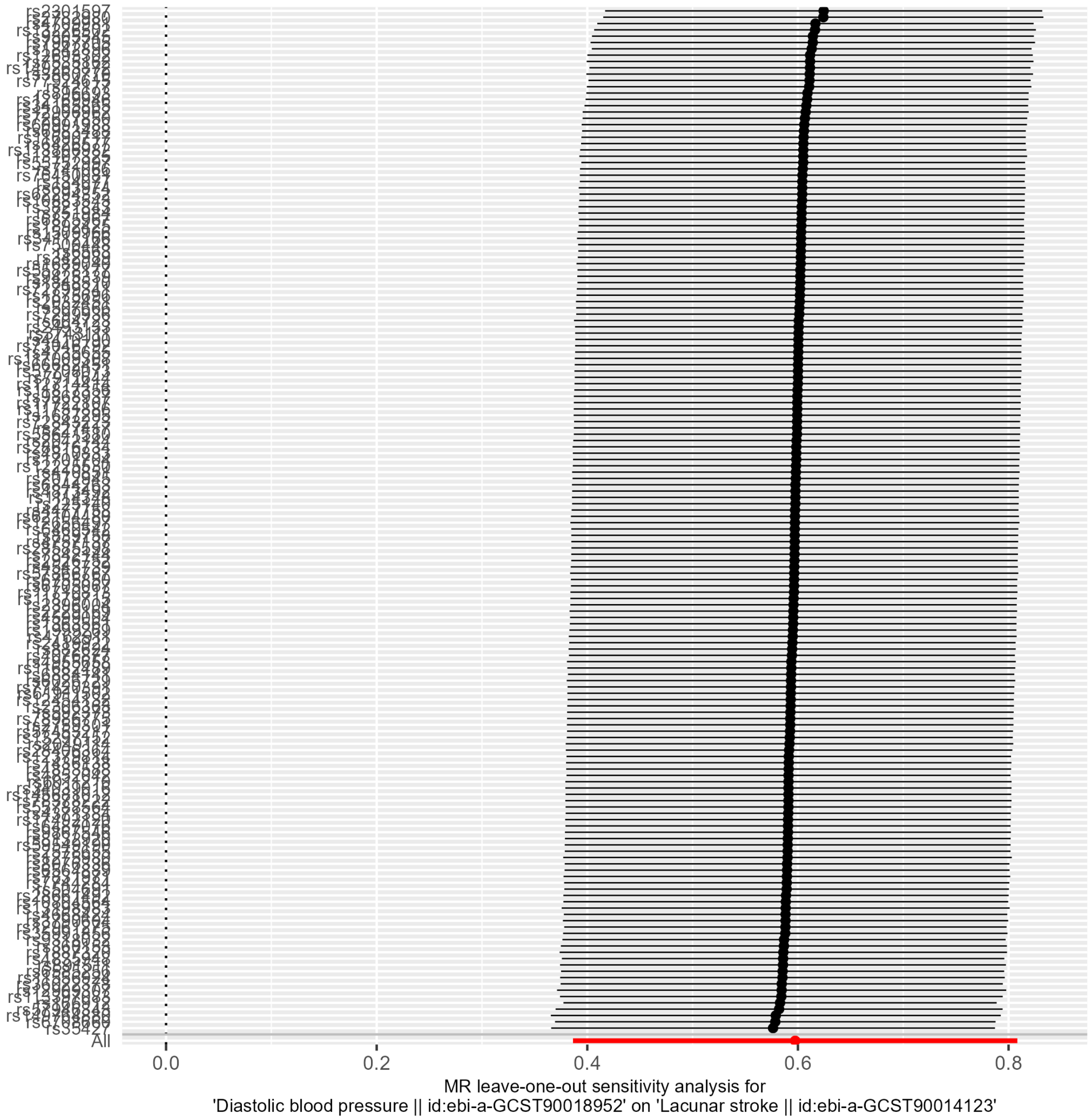

SBP-LAS leave-one-out

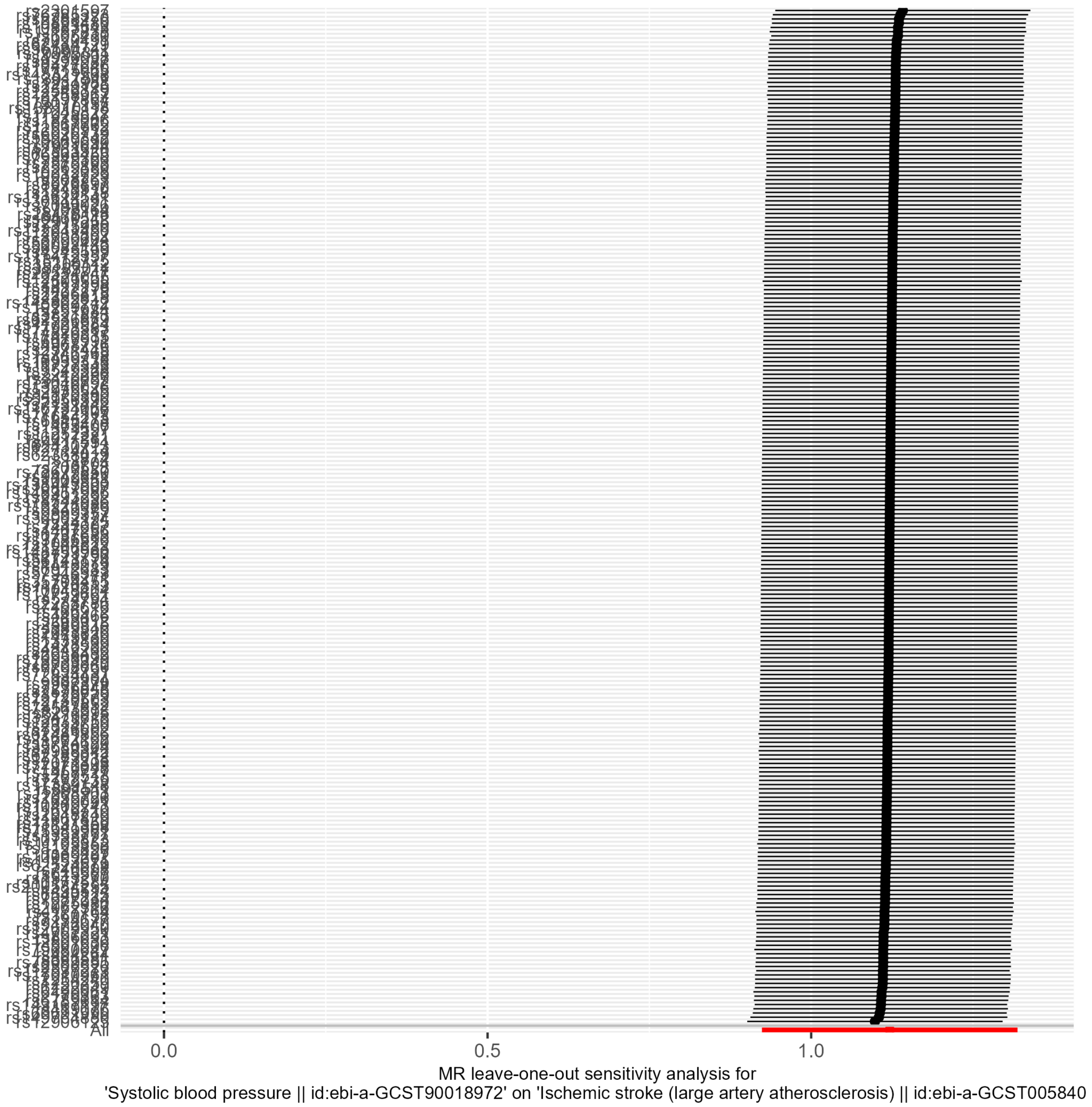

SBP-SVS leave-one-out

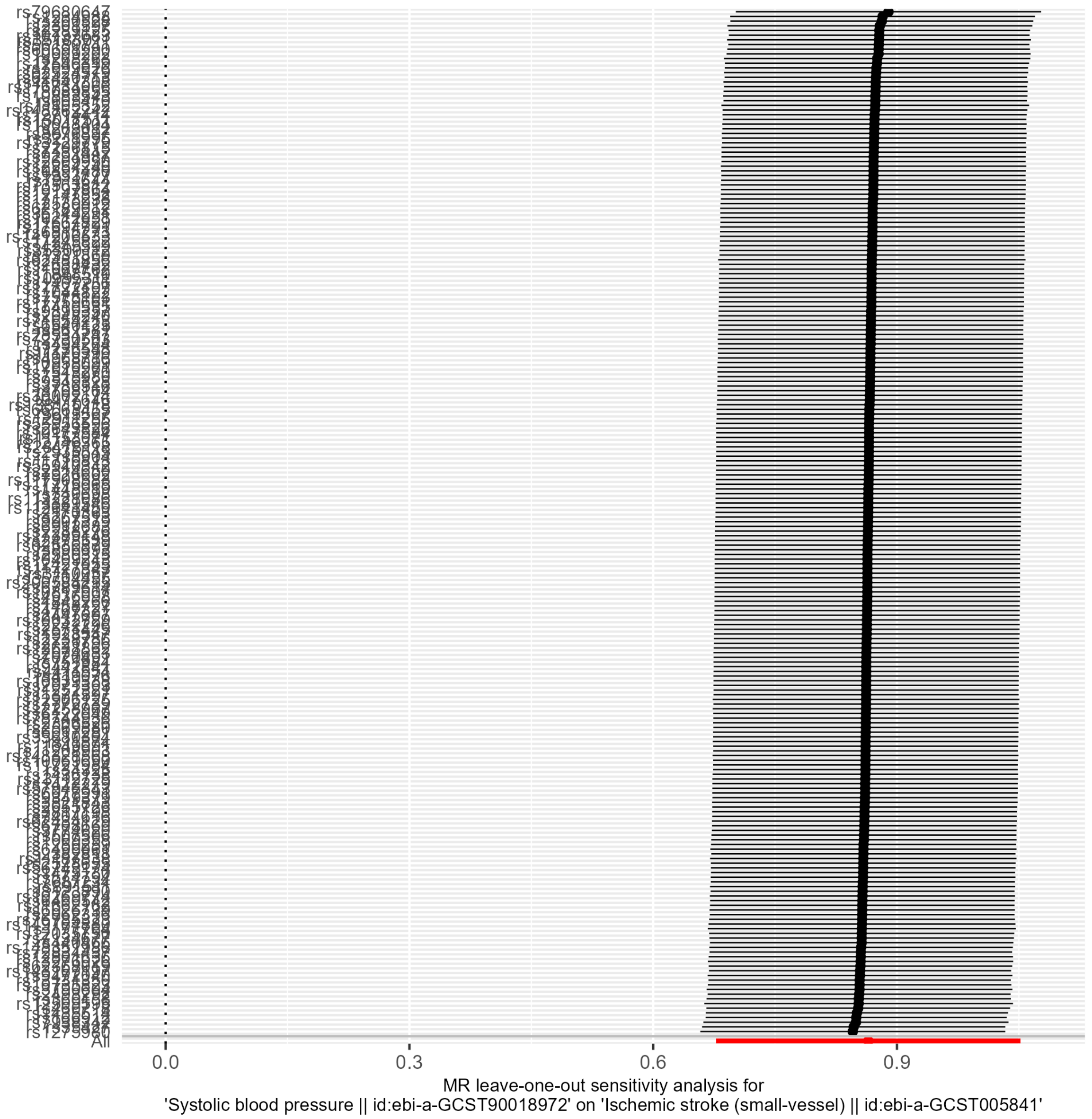

SBP-CES leave-one-out

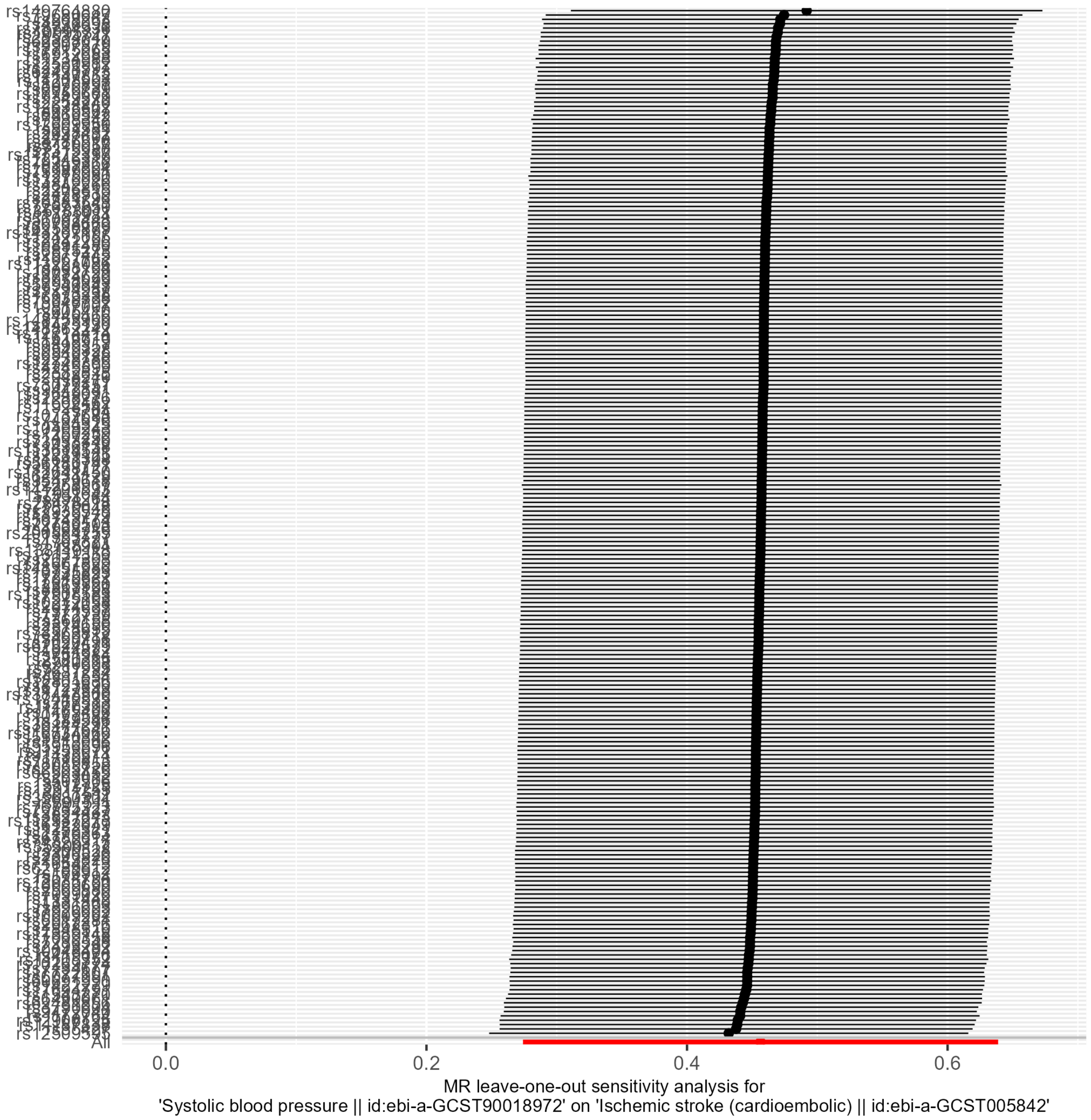

SBP-IS leave-one-out

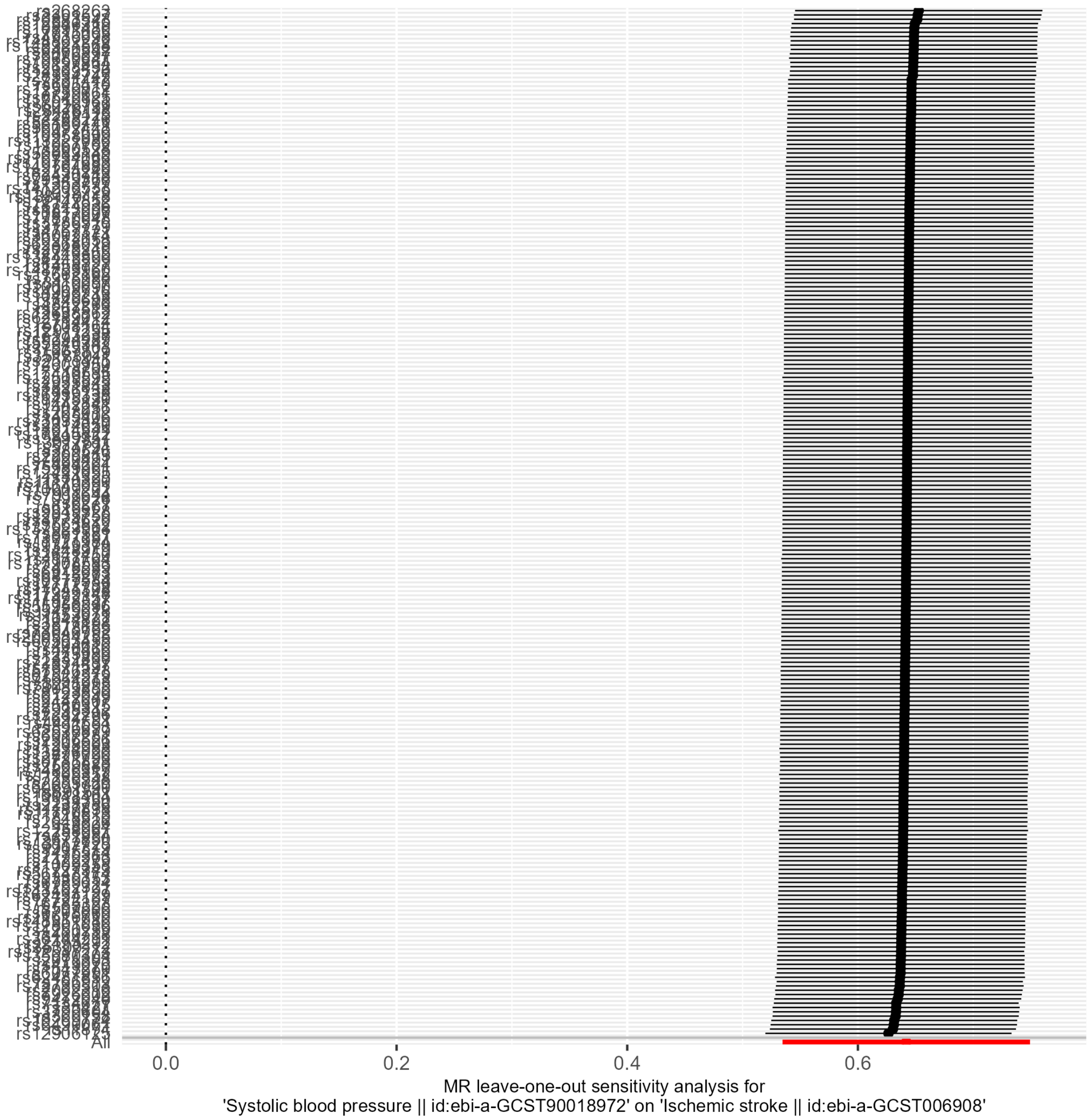

SBP-LS leave-one-out

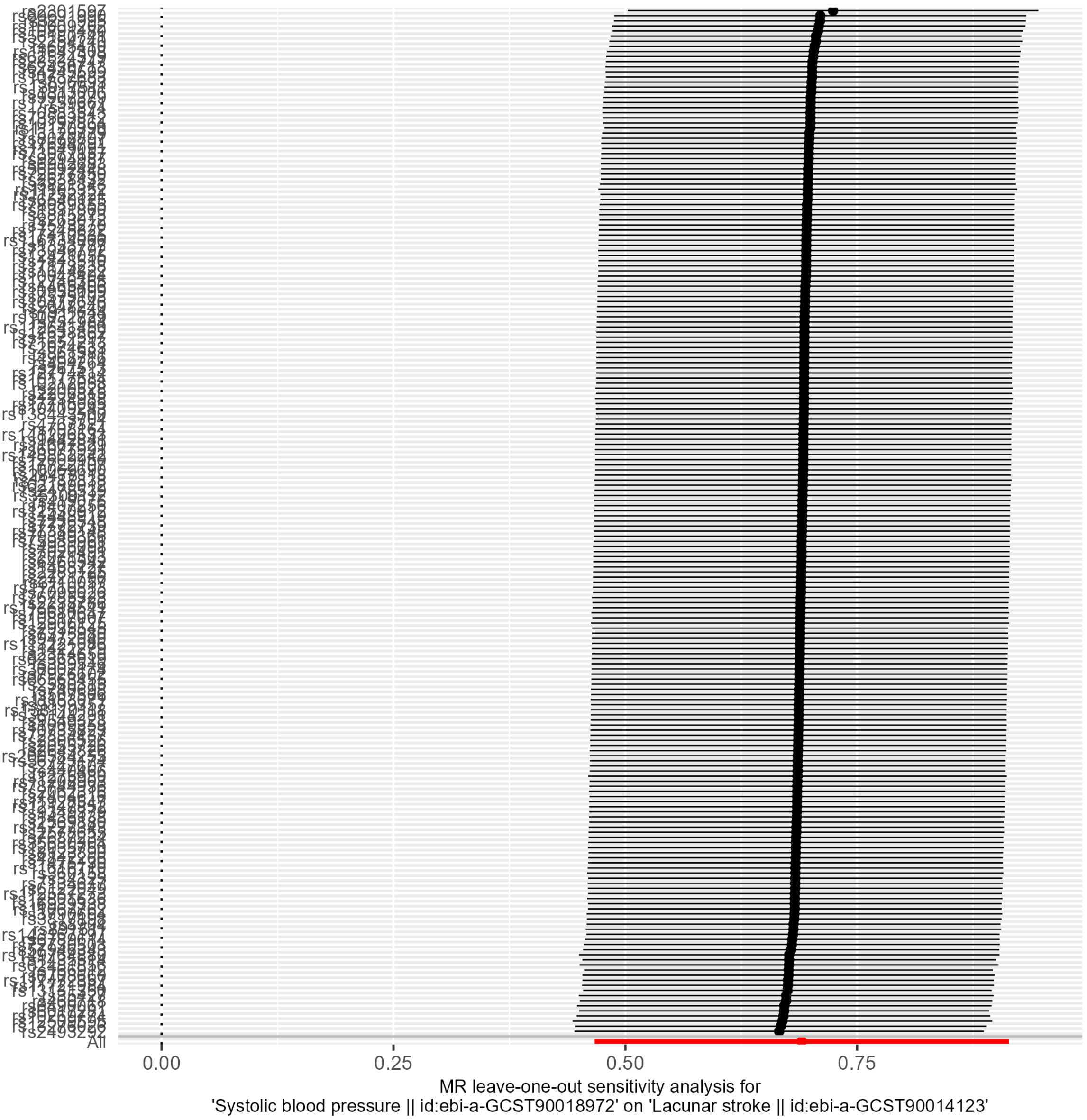

EH-LAS leave-one-out

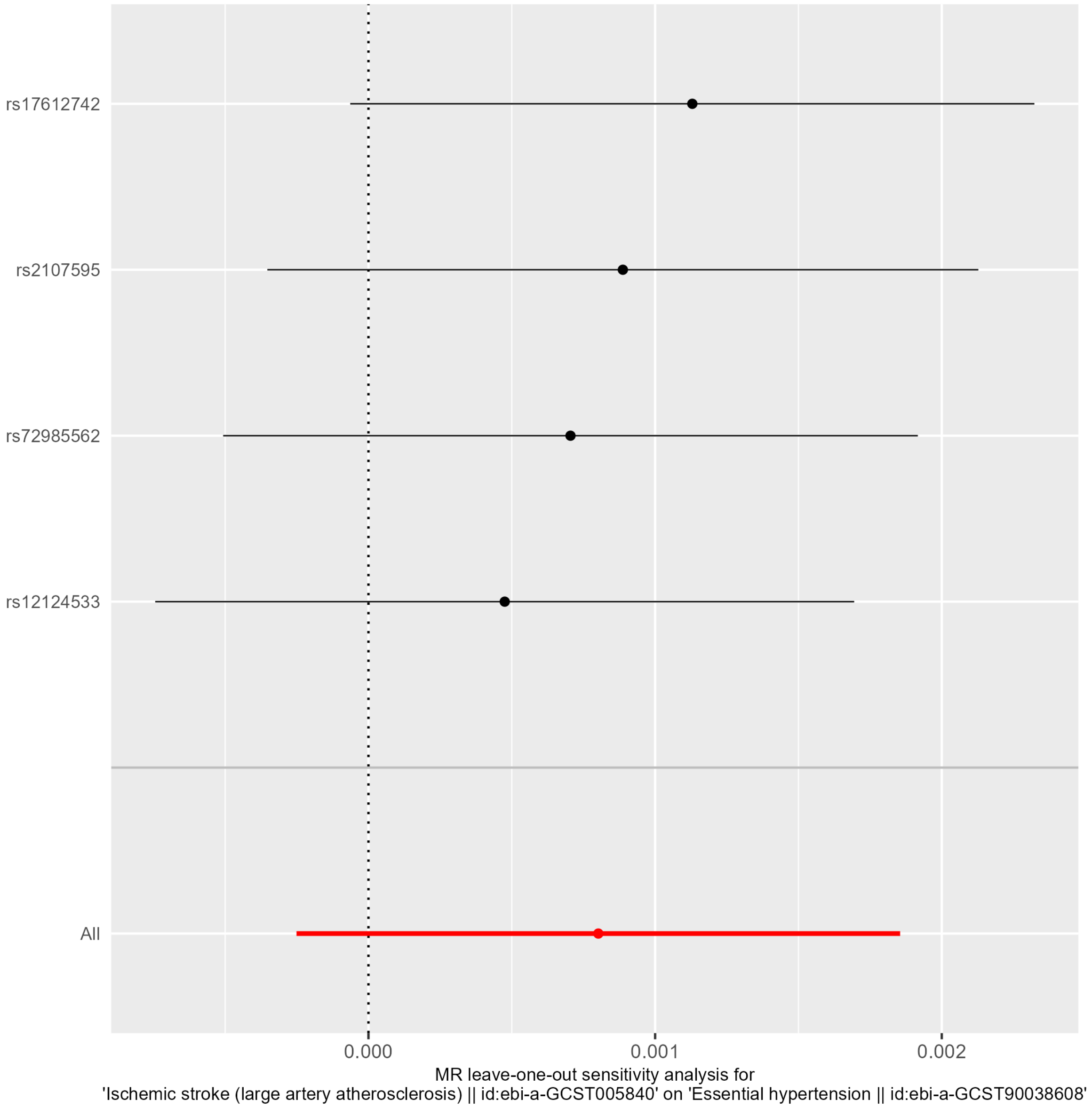

EH-SVS leave-one-out

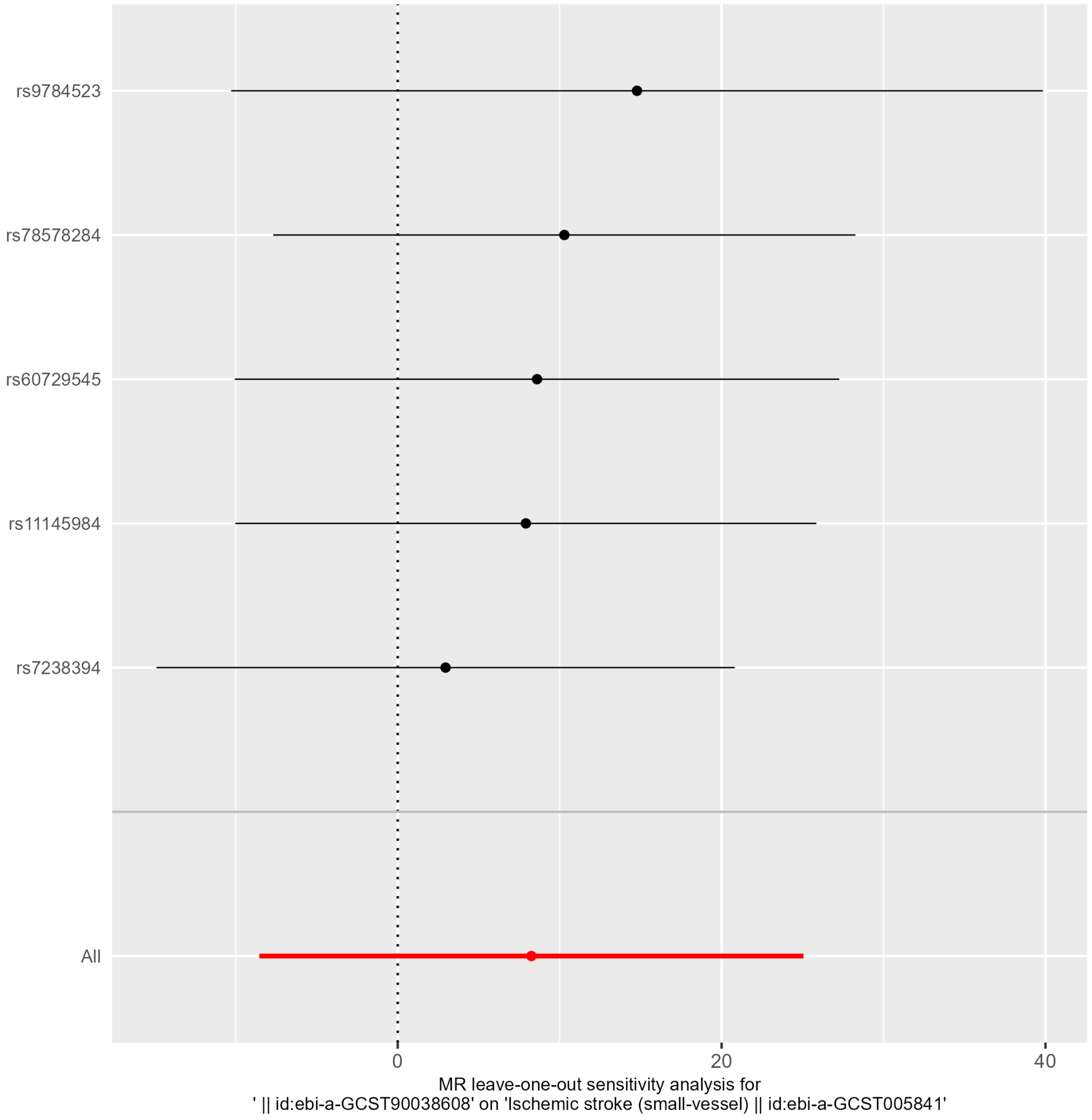

EH-CES leave-one-out

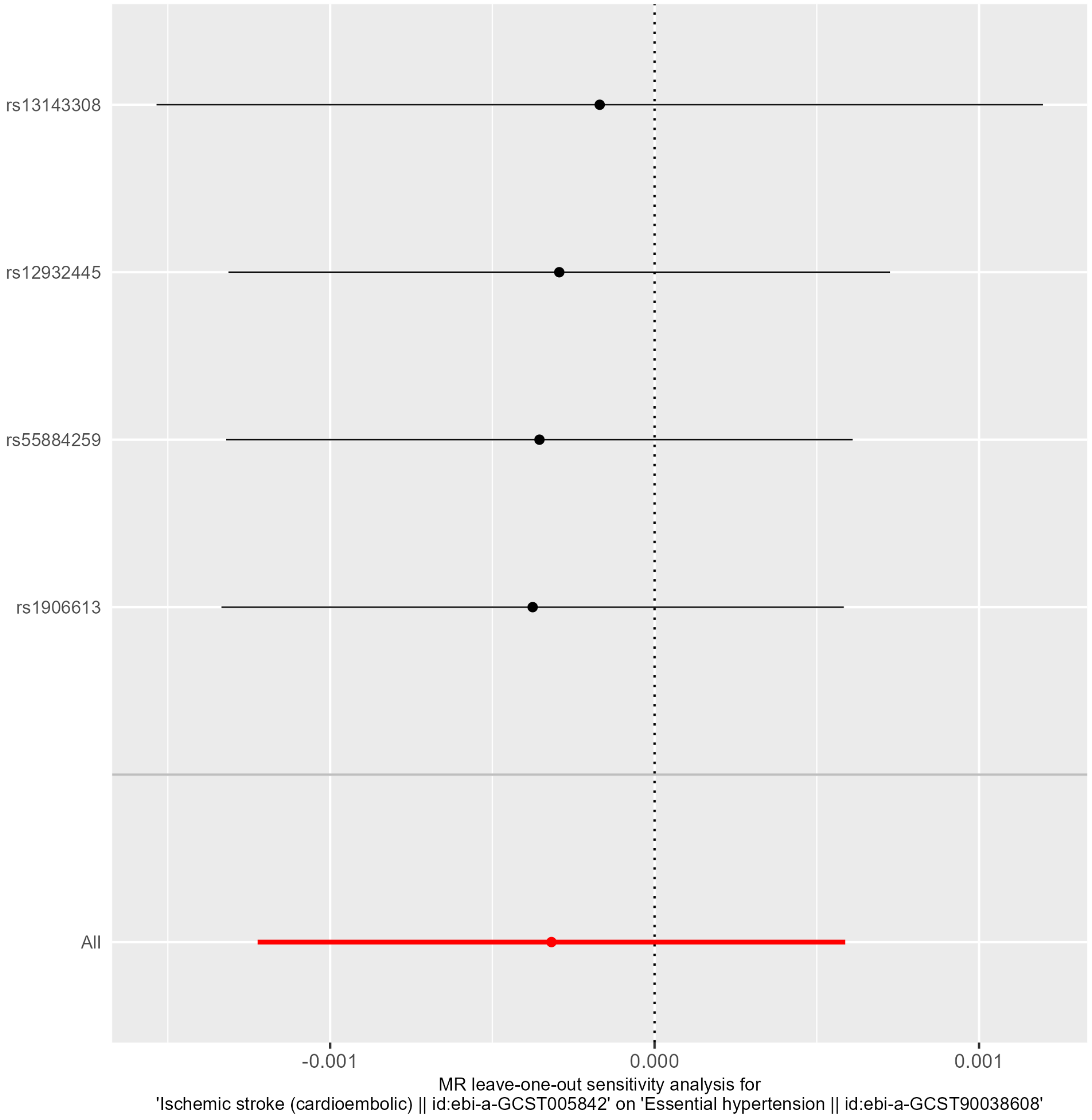

EH-IS leave-one-out

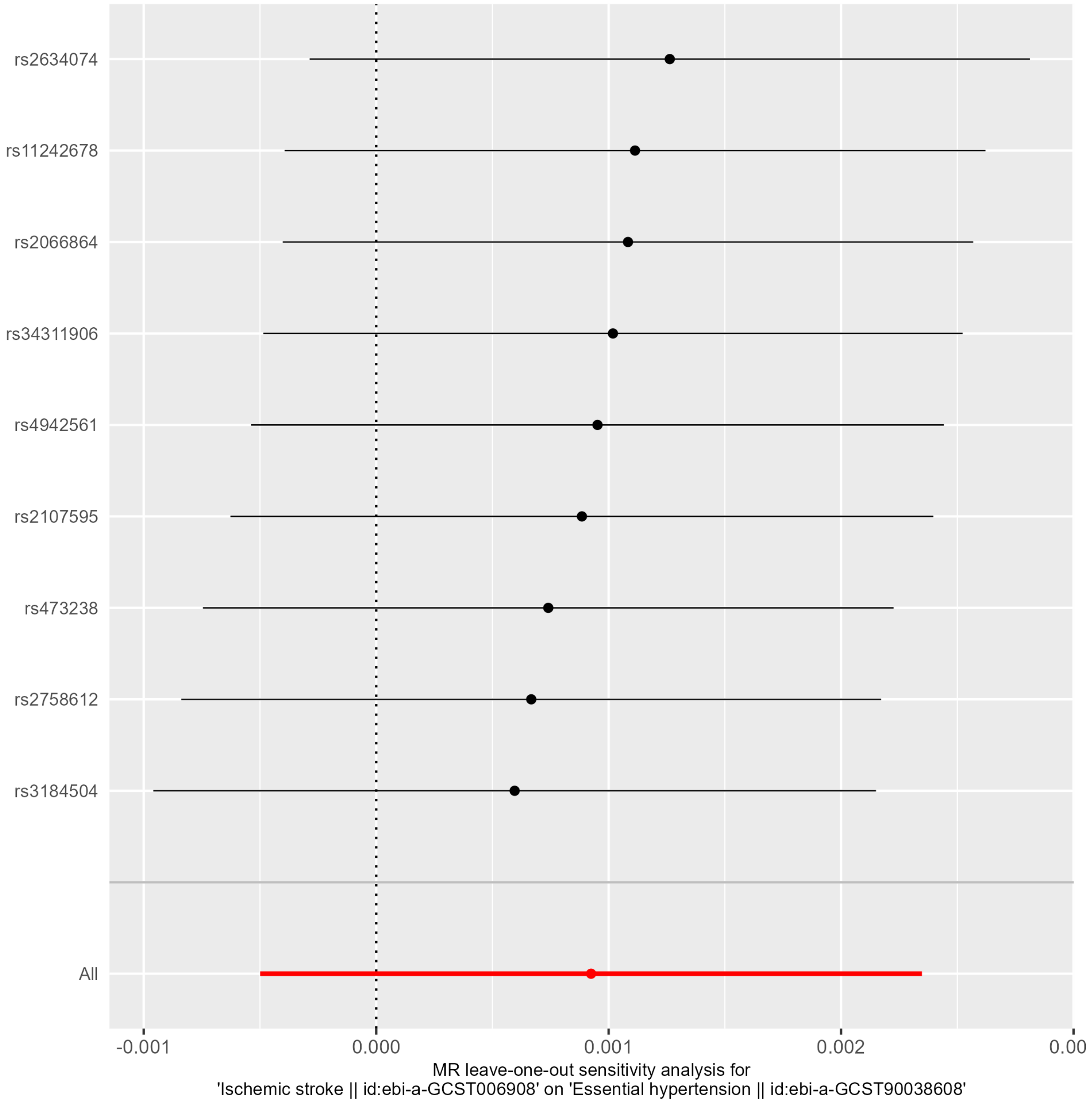

EH-LS leave-one-out

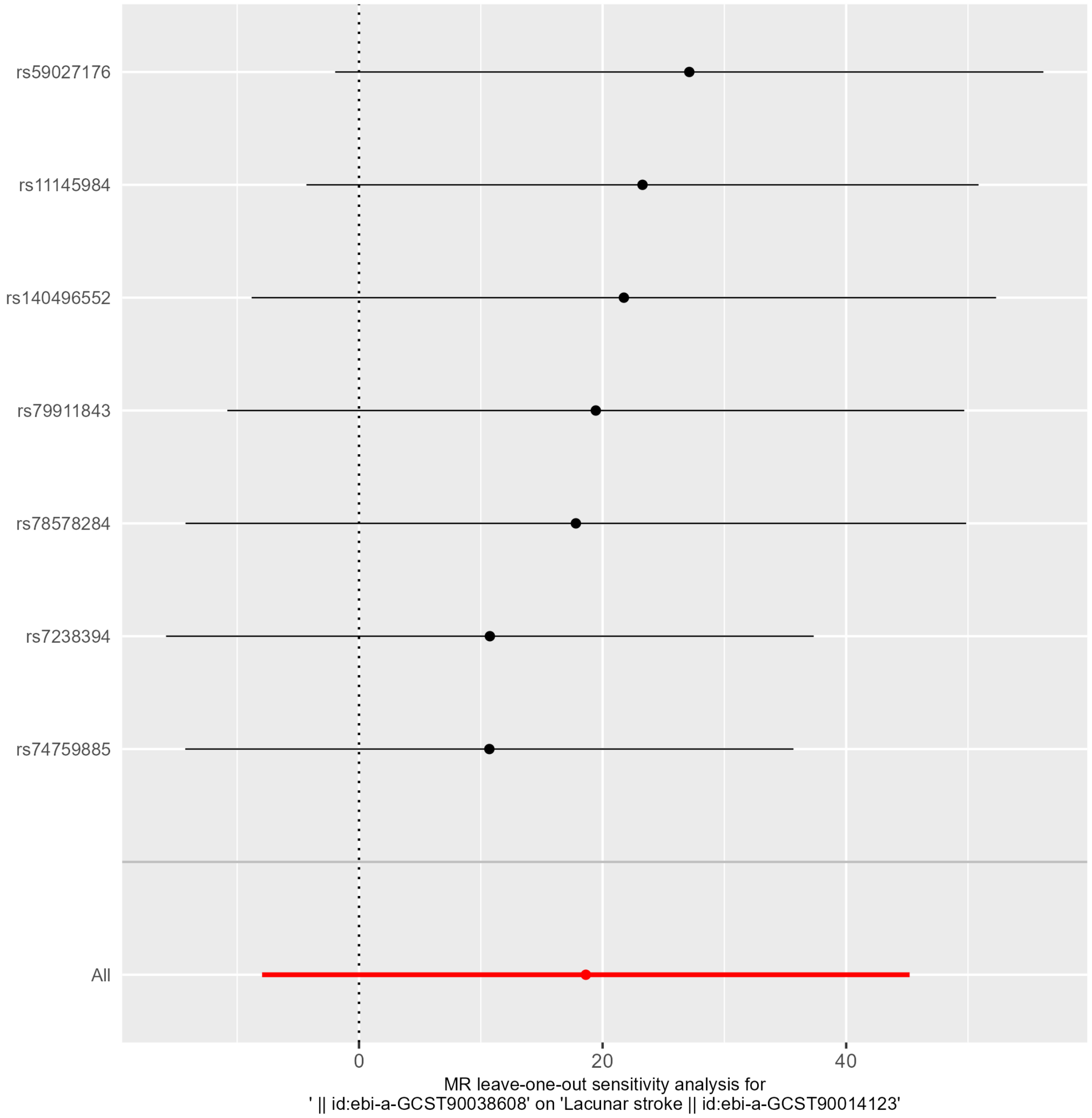

DBP-LAS scatter plot

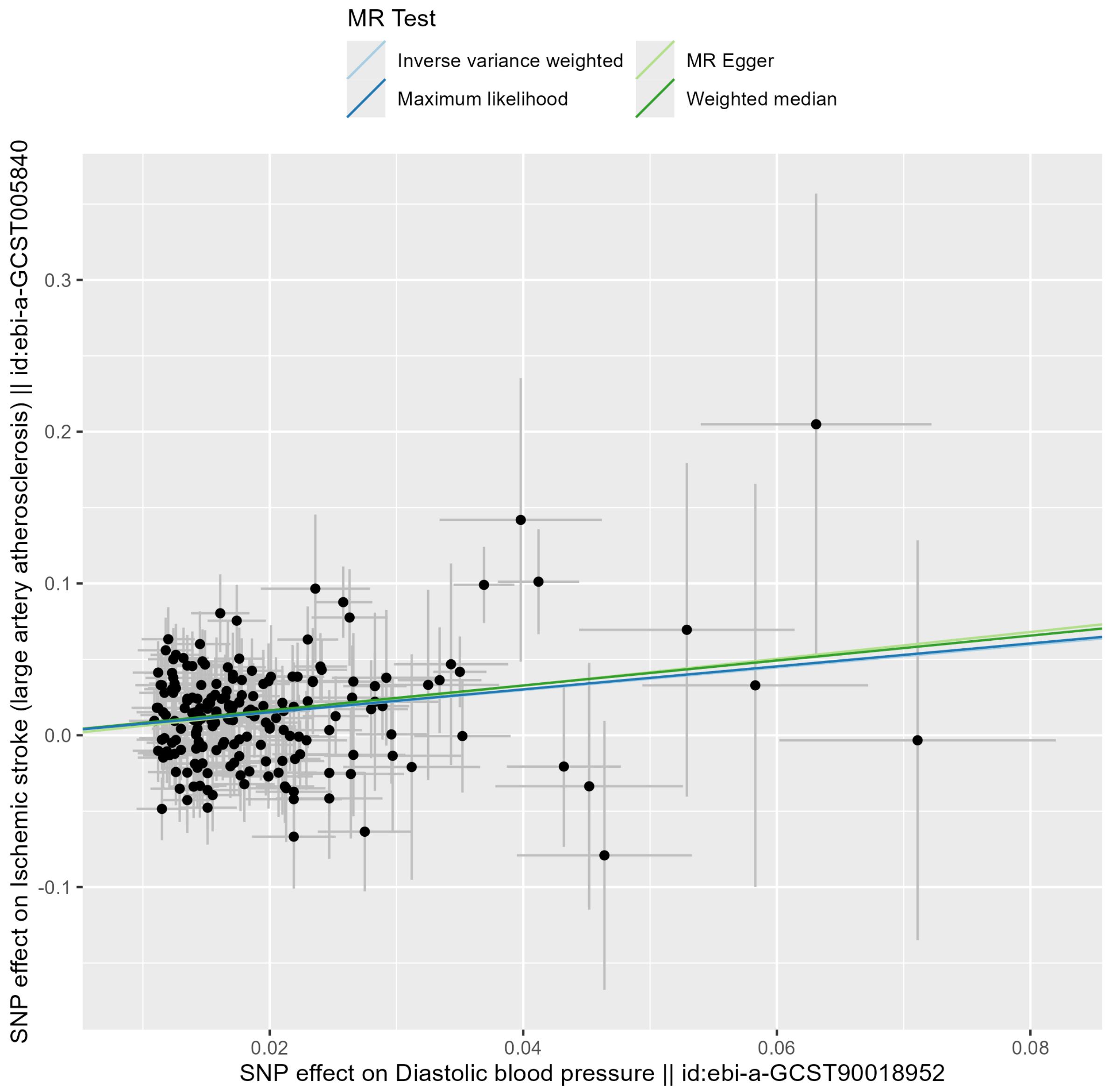

DBP-SVS scatter plot

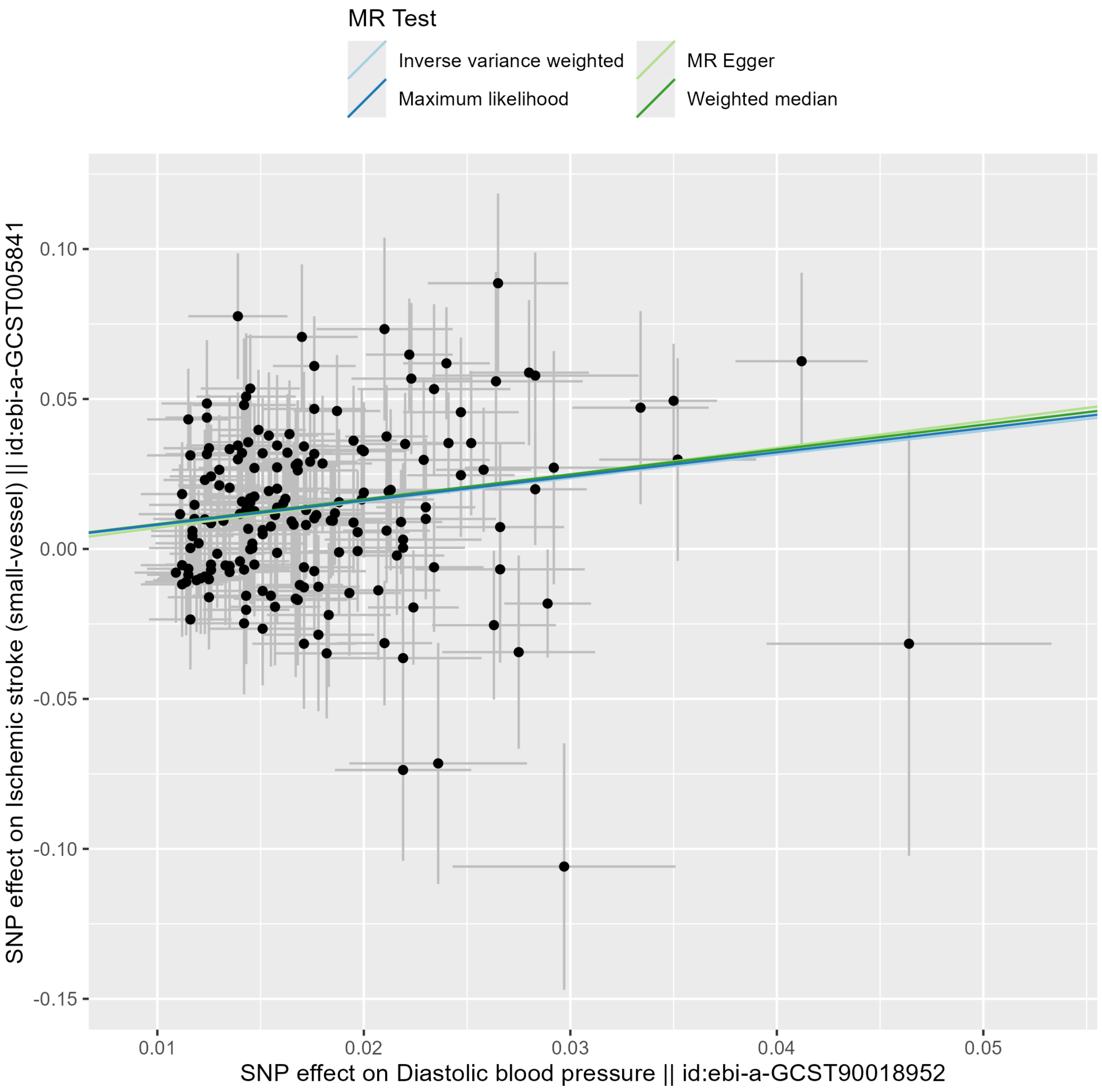

DBP-CES scatter plot

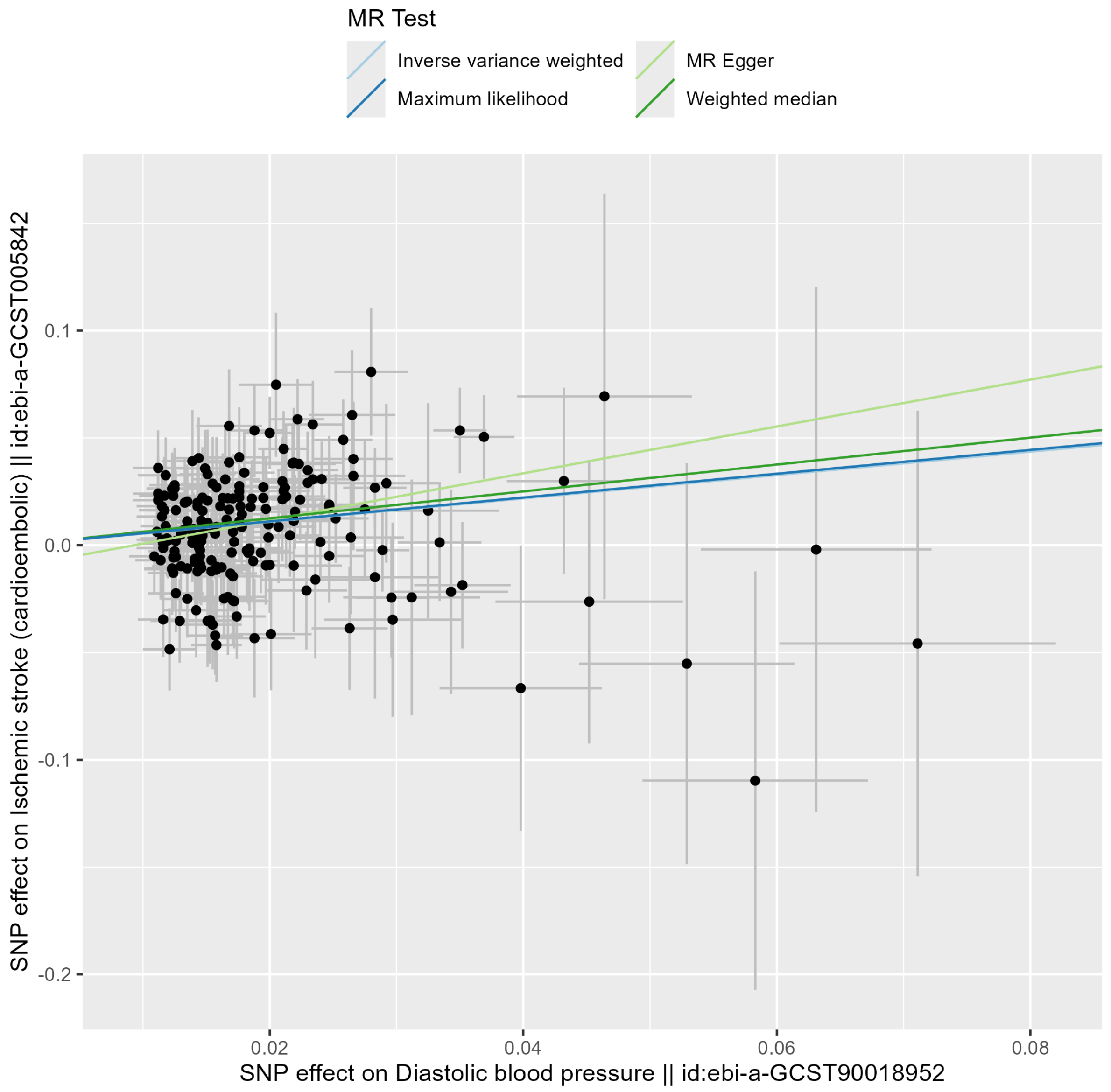

DBP-IS scatter plot

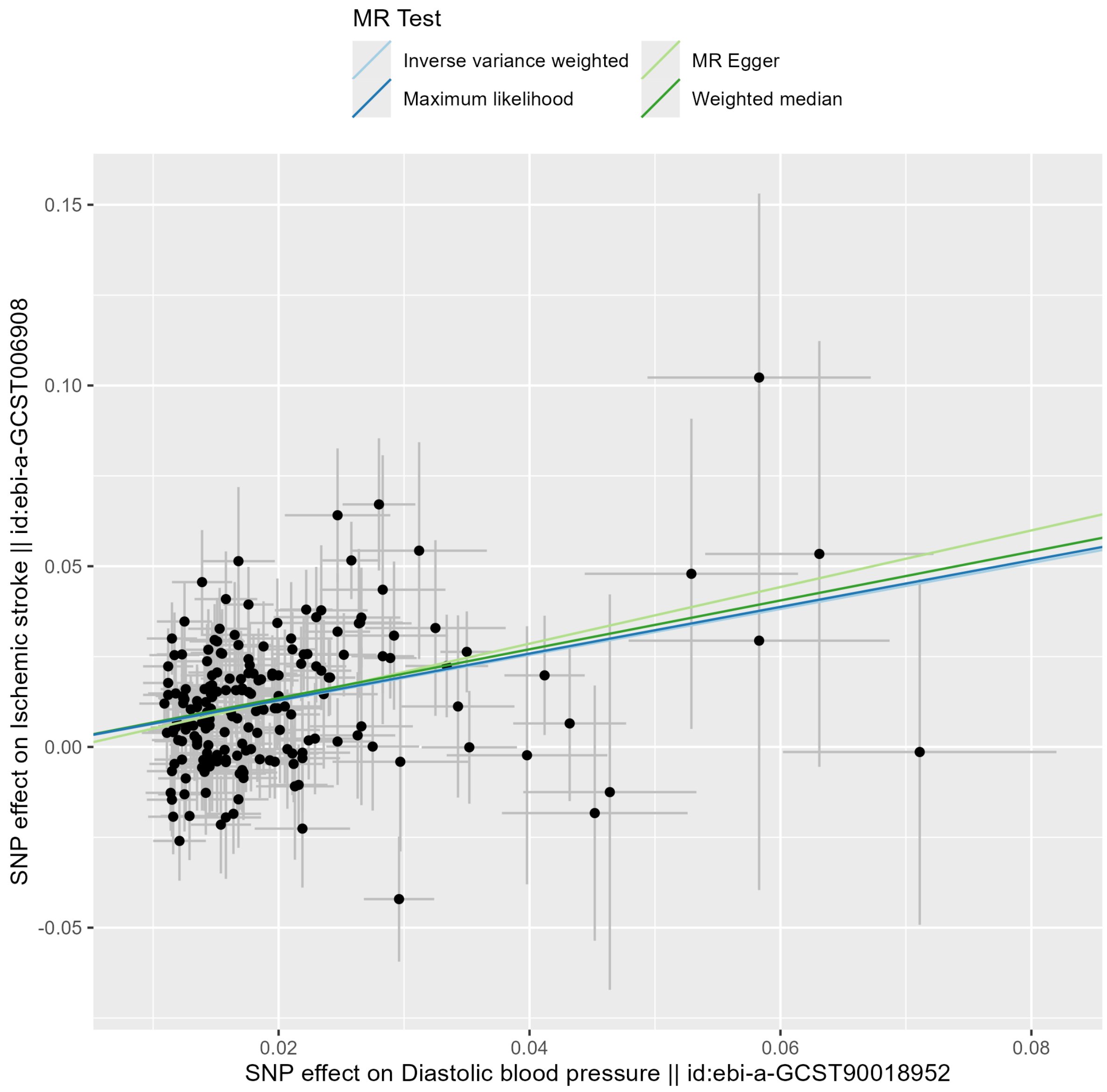

DBP-LS scatter plot

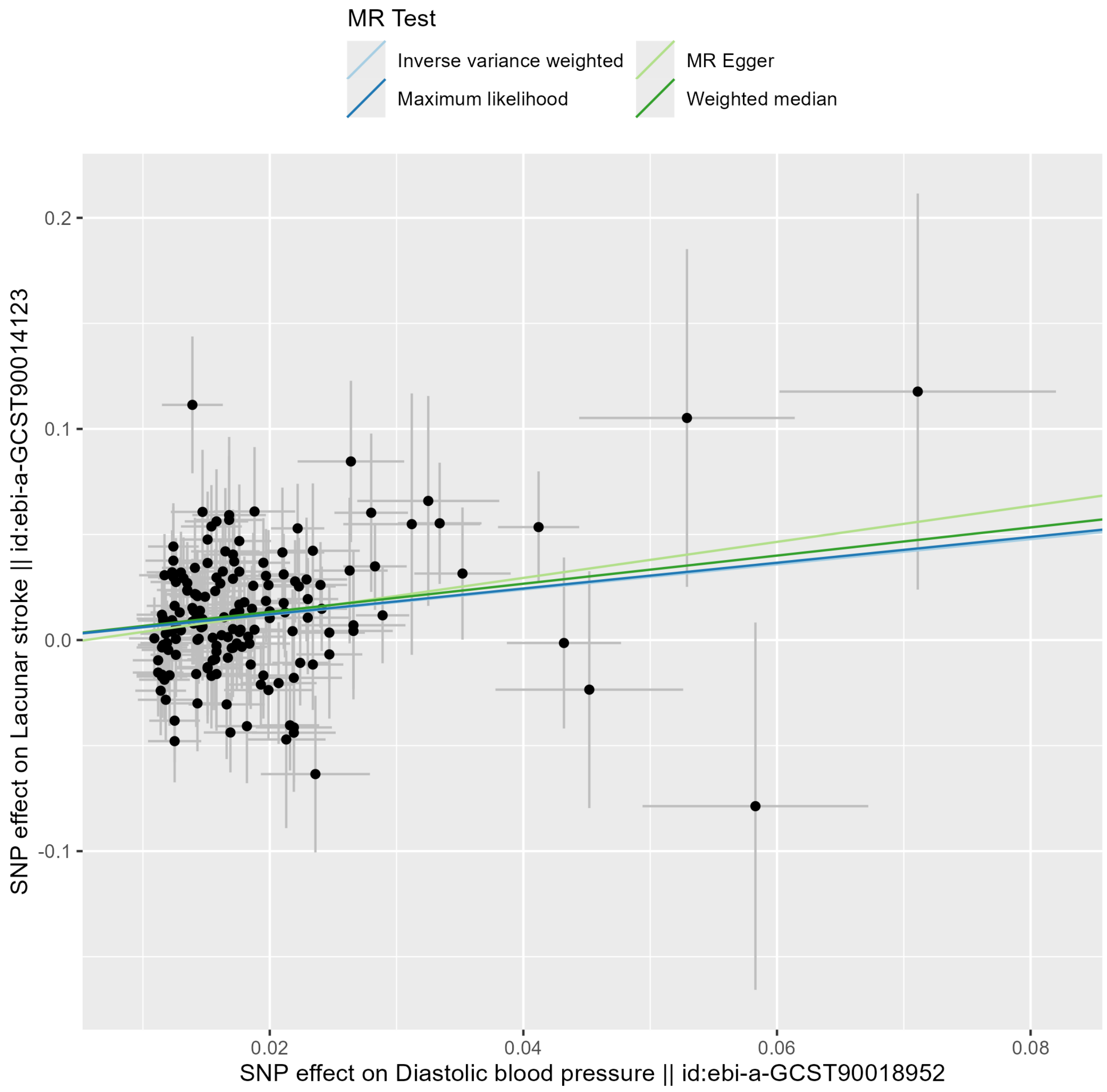

SBP-LAS scatter plot

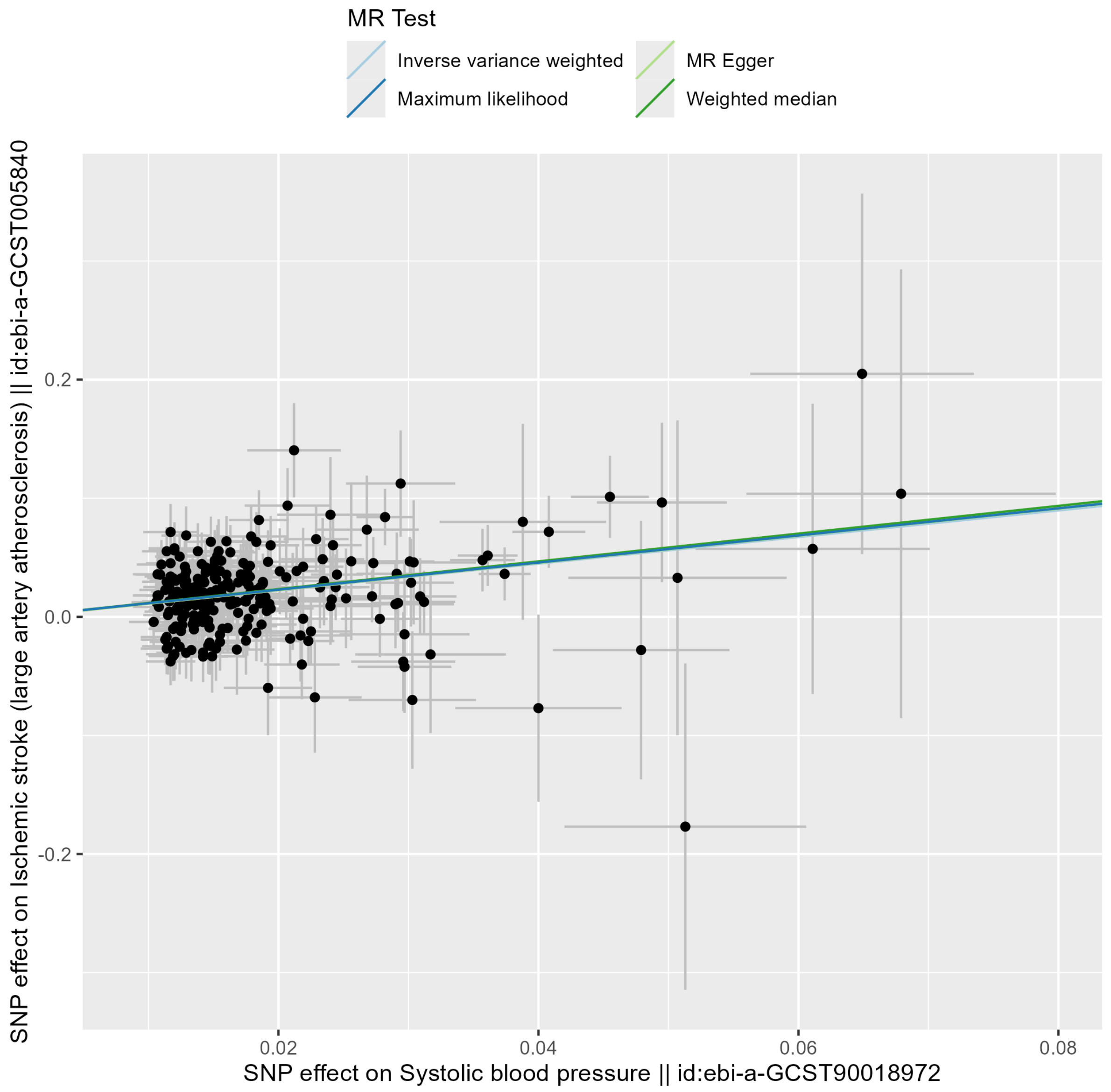

SBP-SVS scatter plot

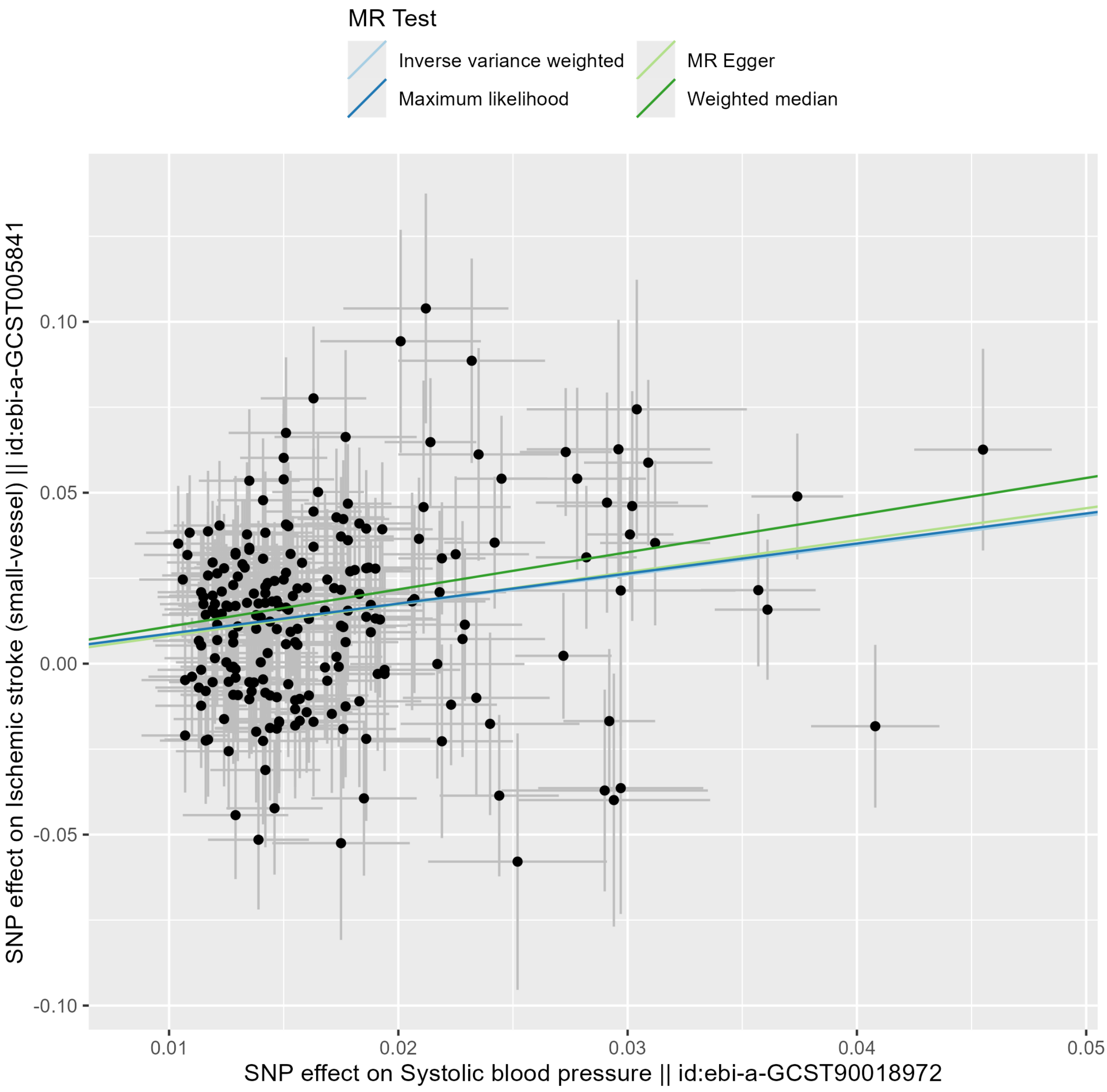

SBP-CES scatter plot

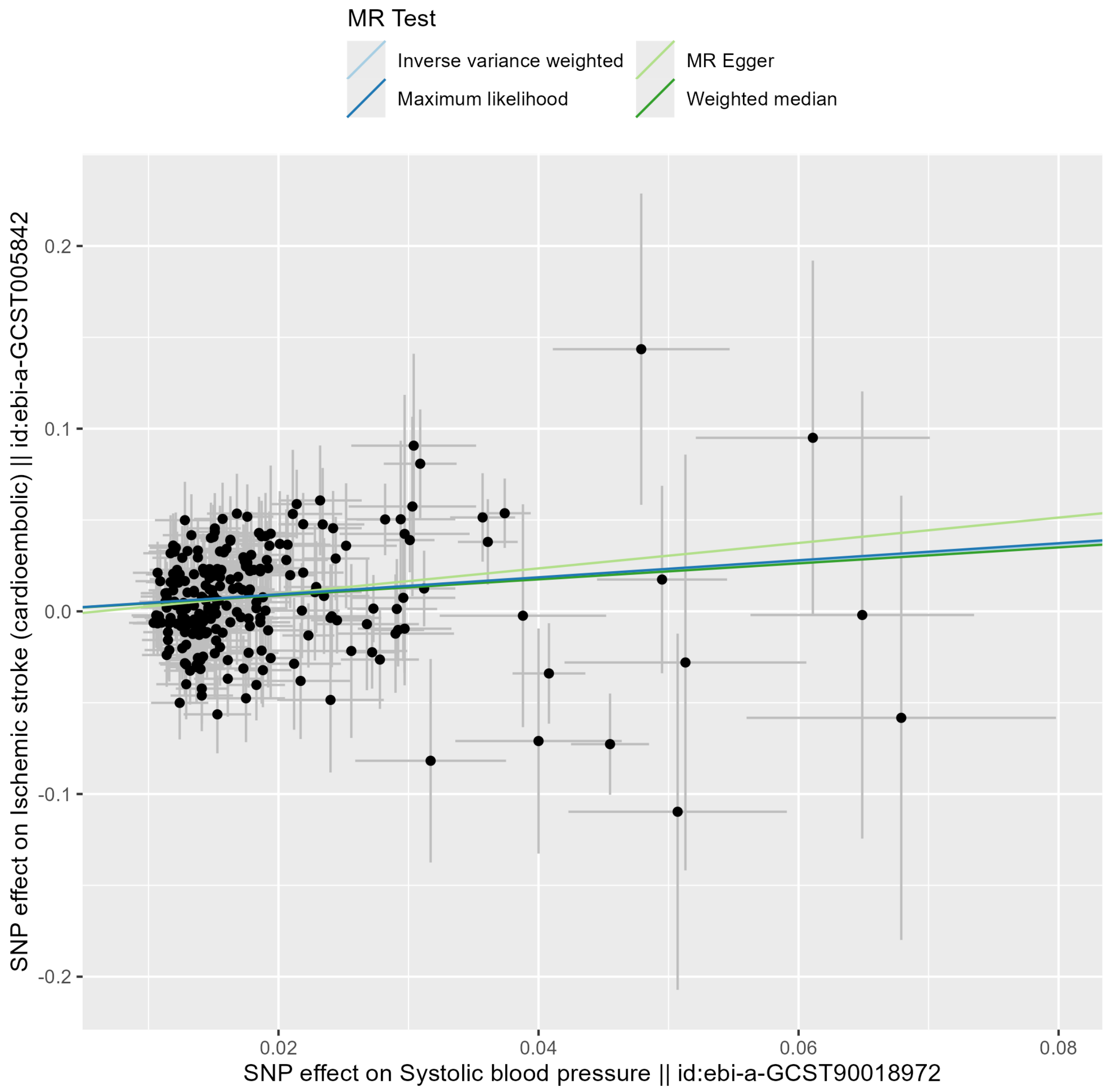

SBP-IS scatter plot

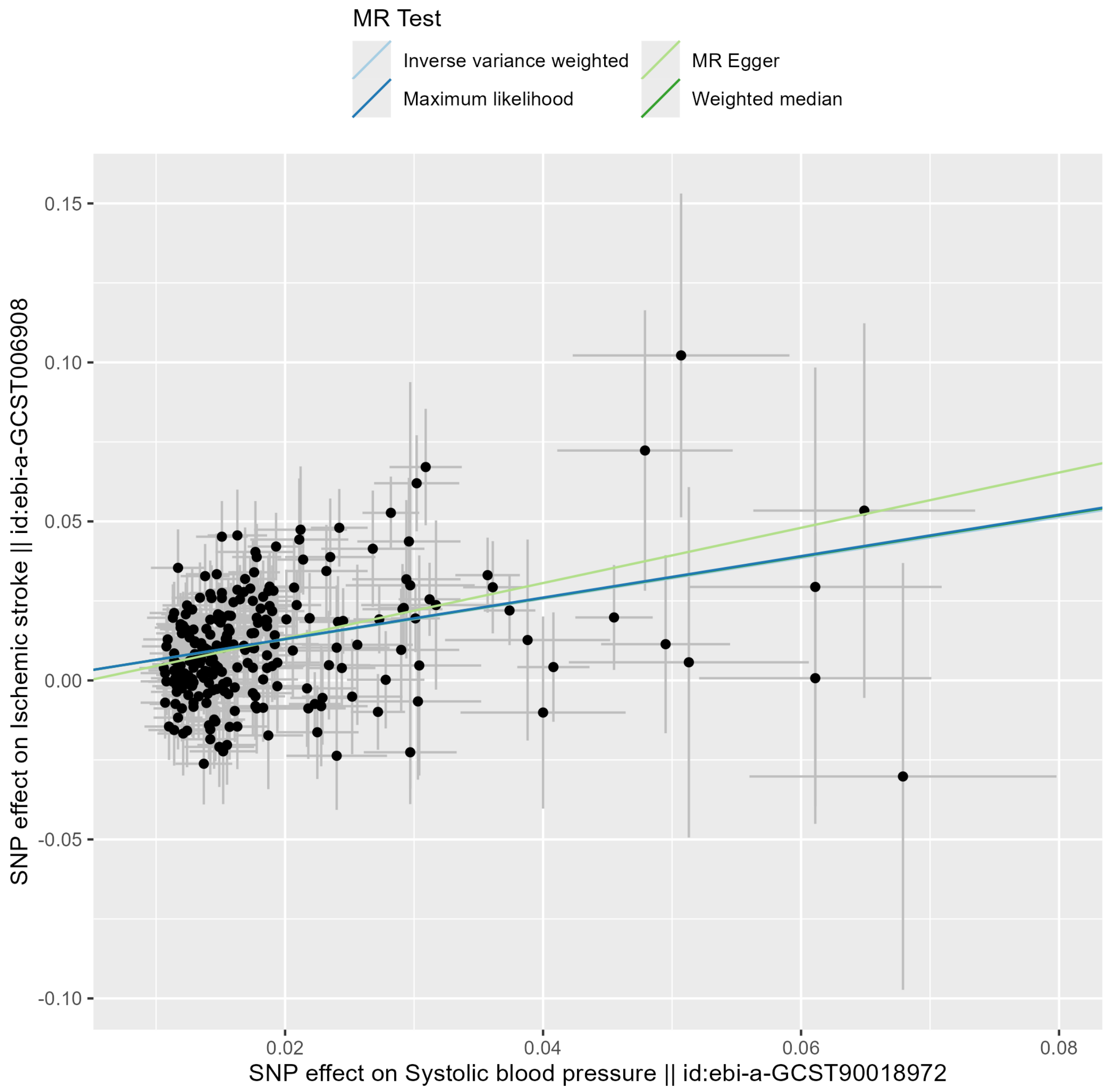

SBP-LS scatter plot

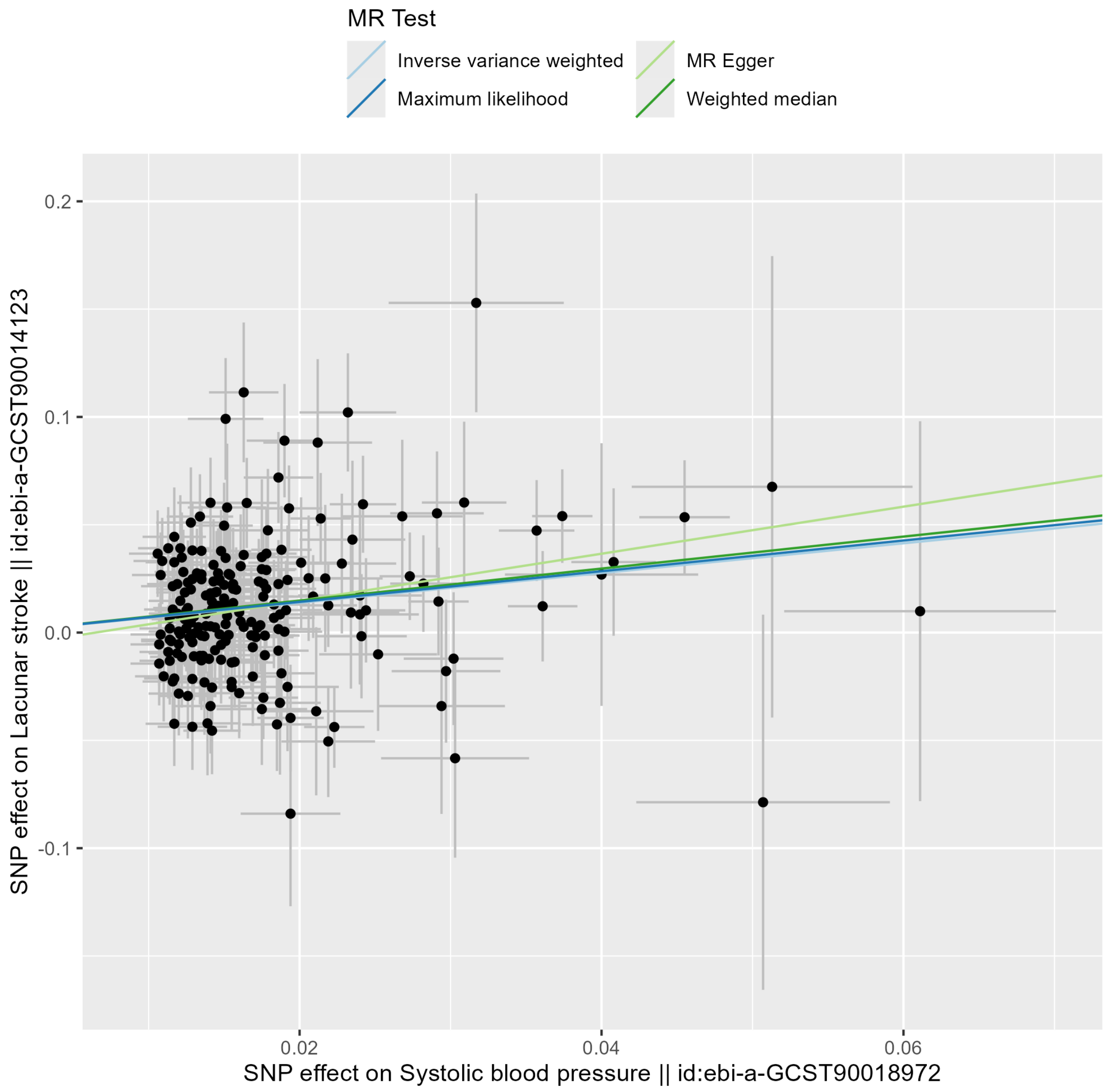

EH-LAS scatter plot

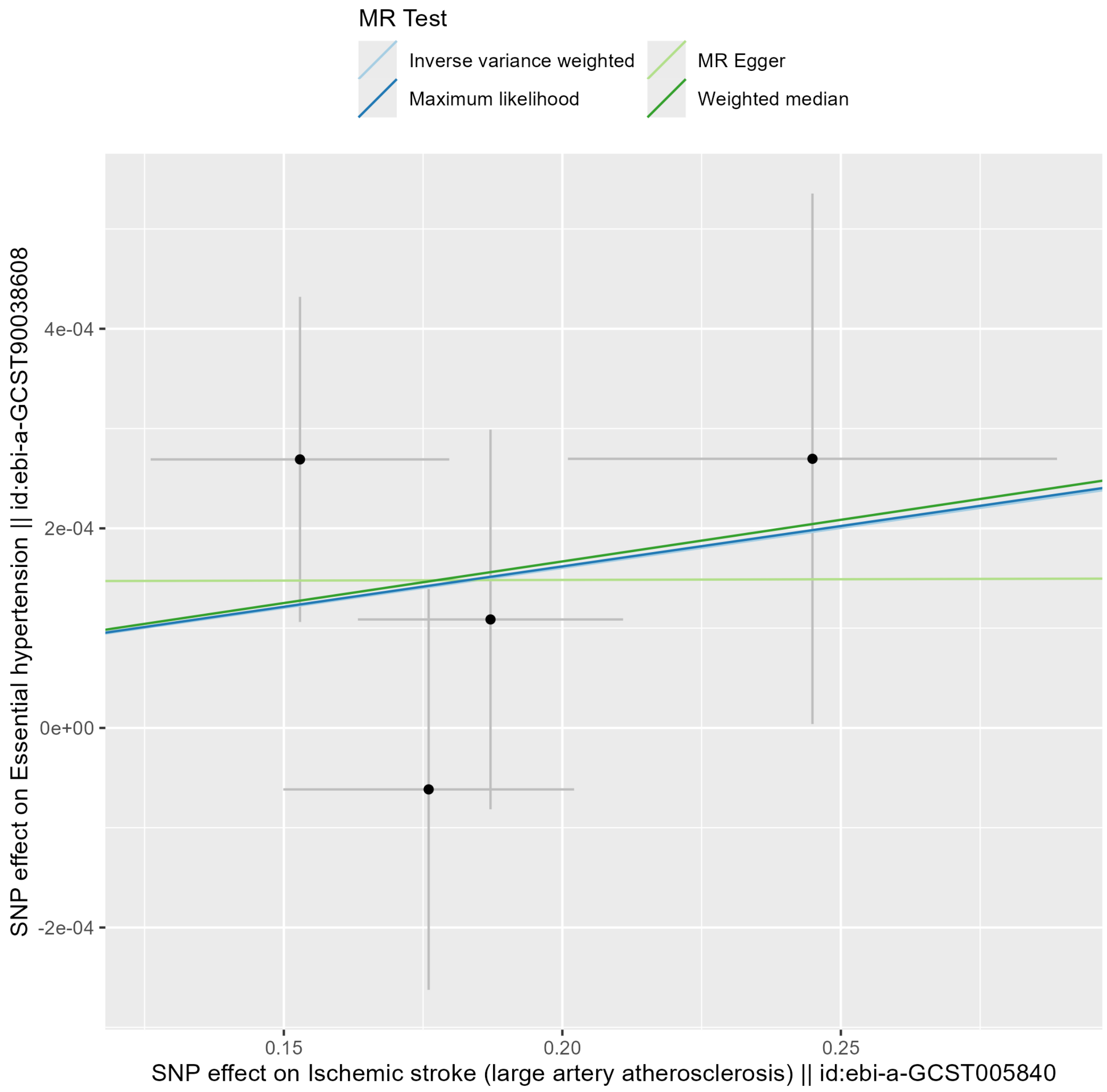

EH-SVS scatter plot

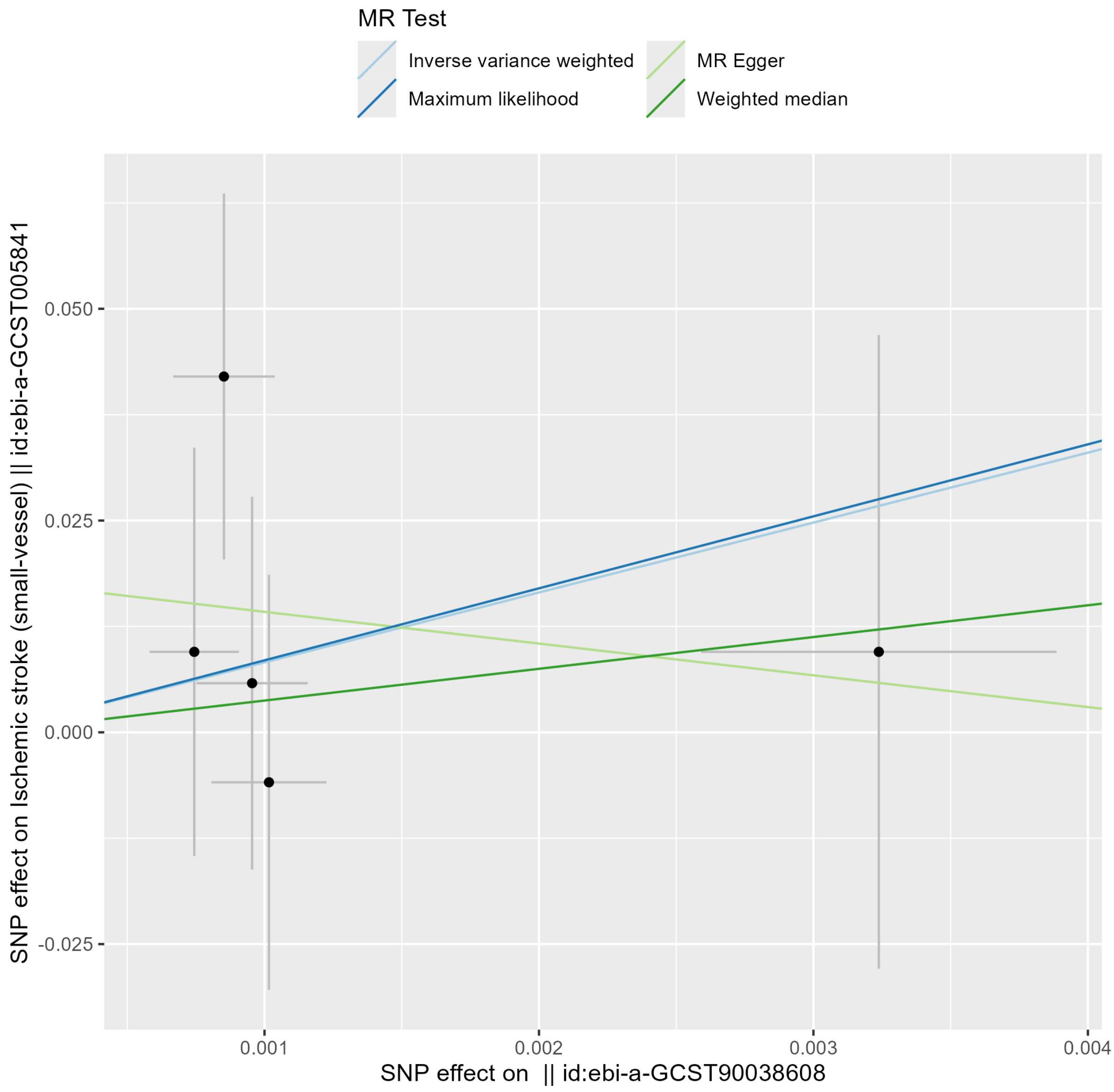

EH-CES scatter plot

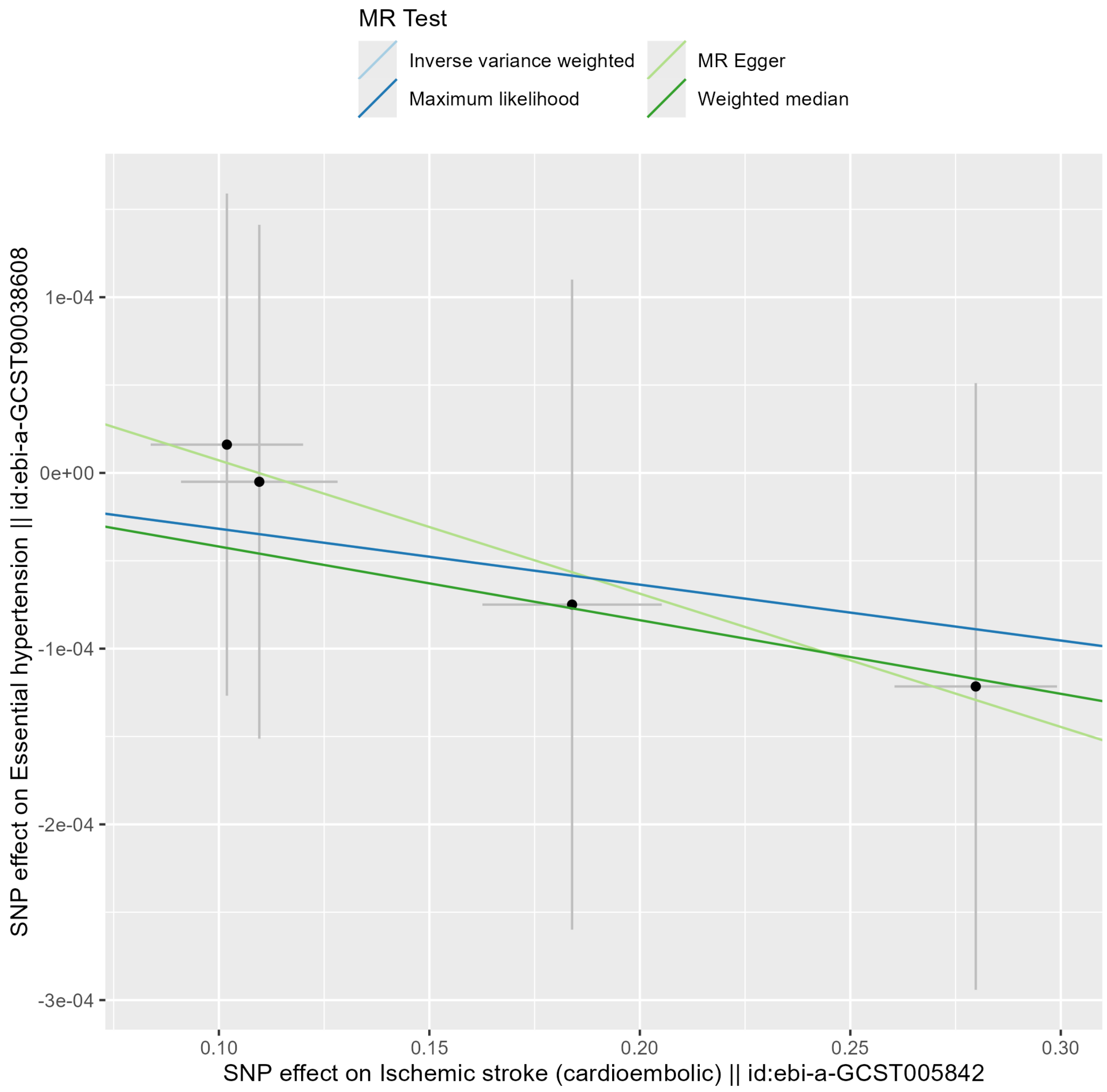

EH-IS scatter plot

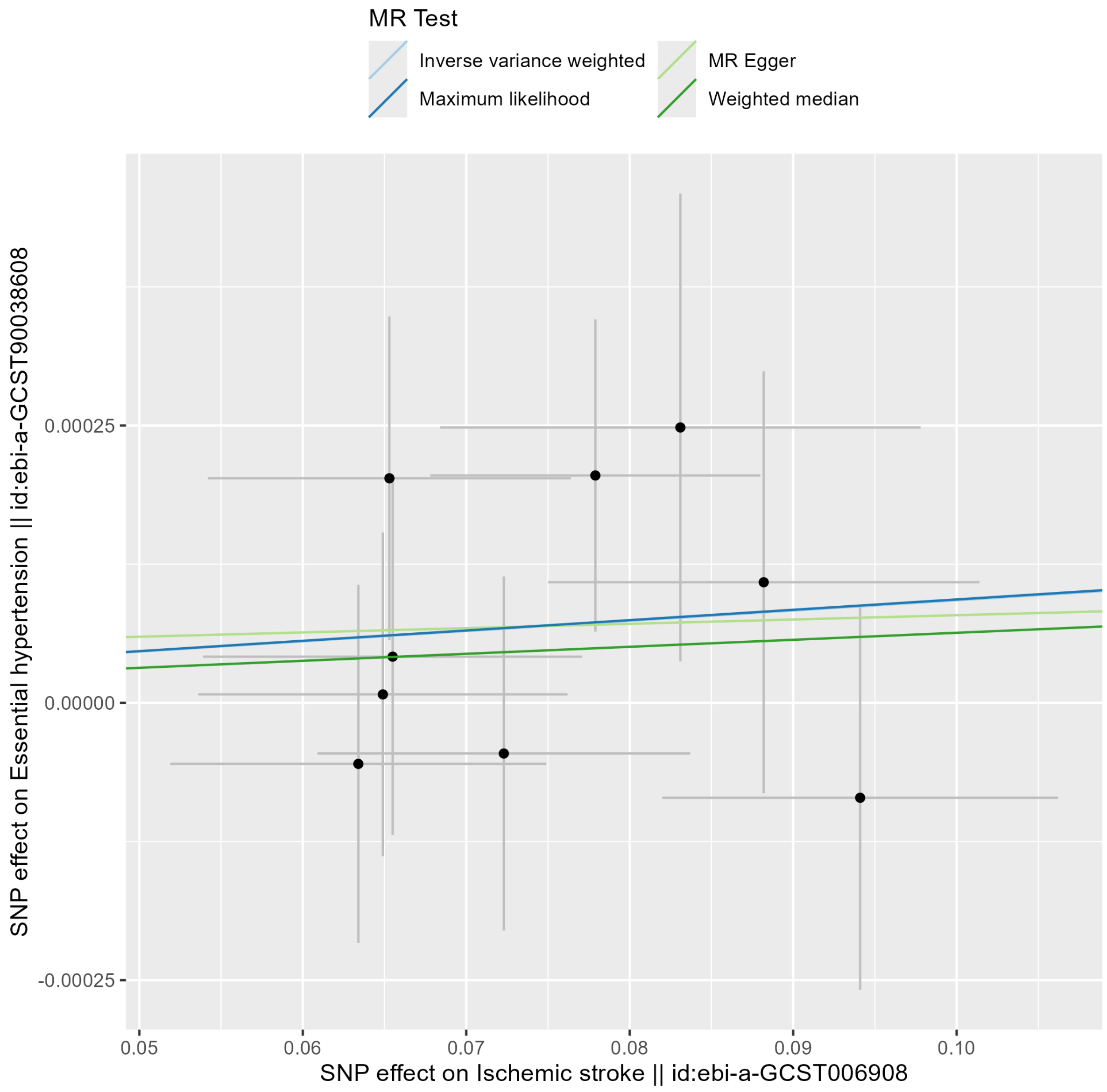

EH-LS scatter plot

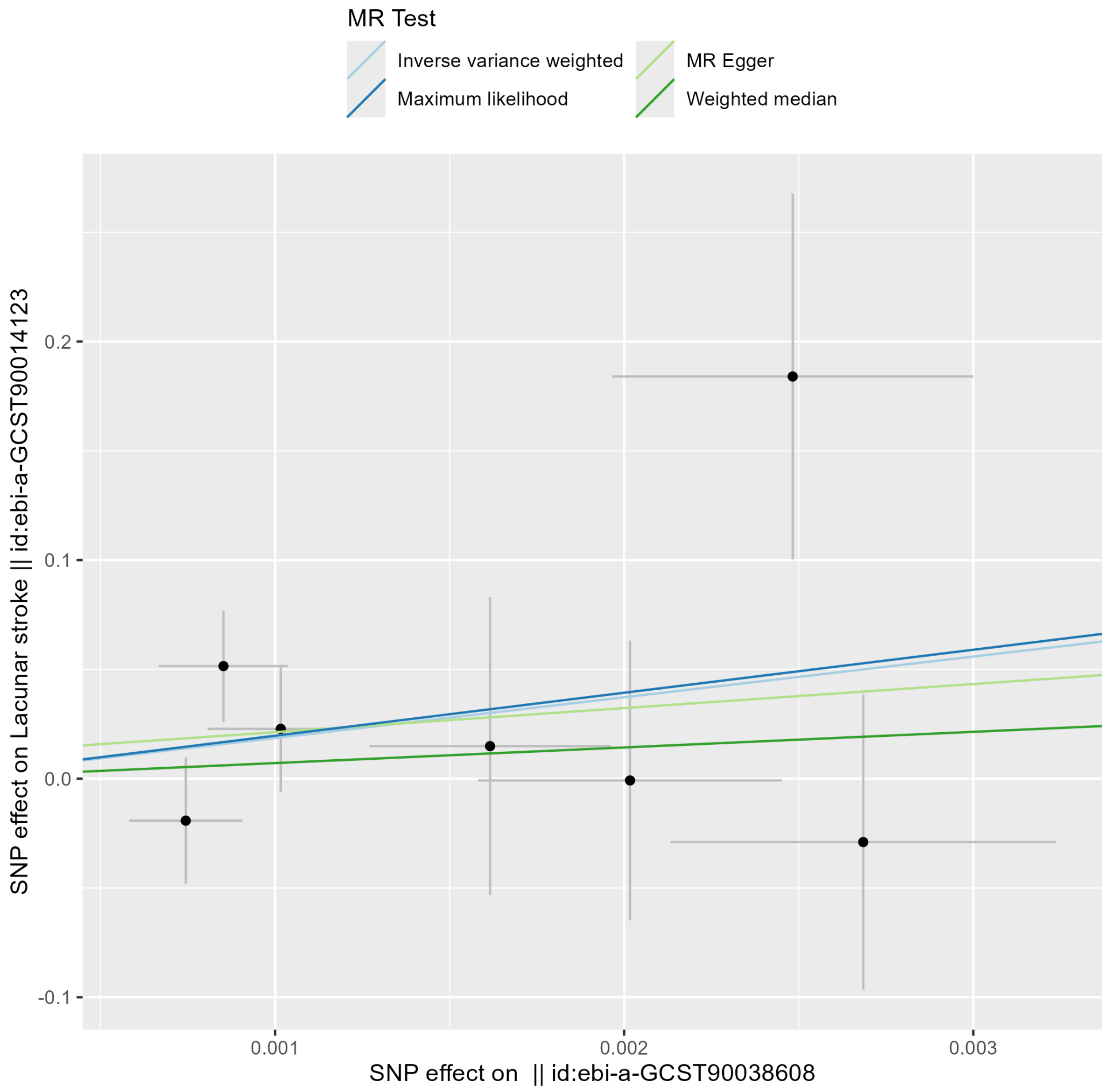

DBP-LAS forest plot

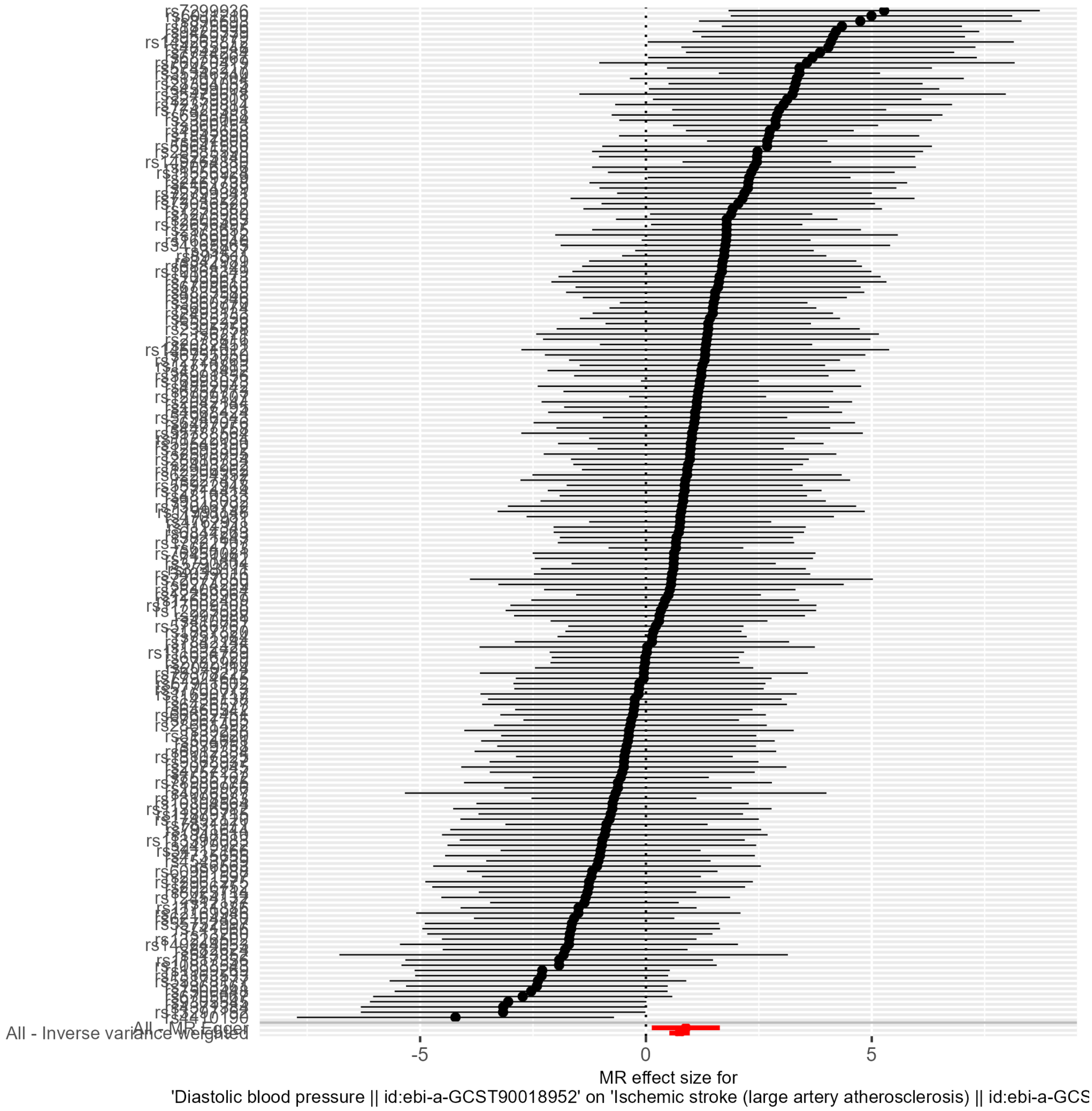

DBP-SVS forest plot

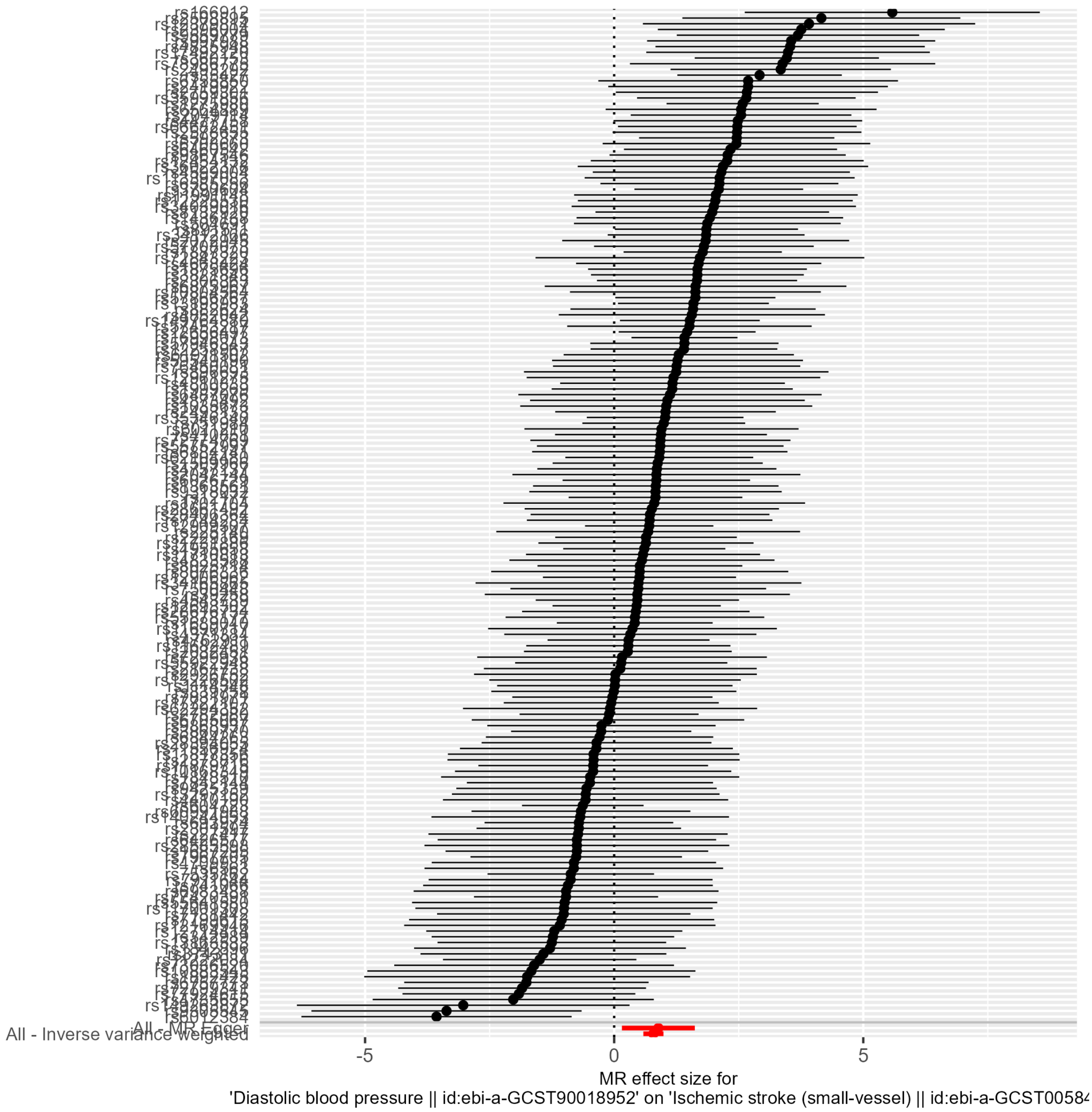

DBP-CES forest plot

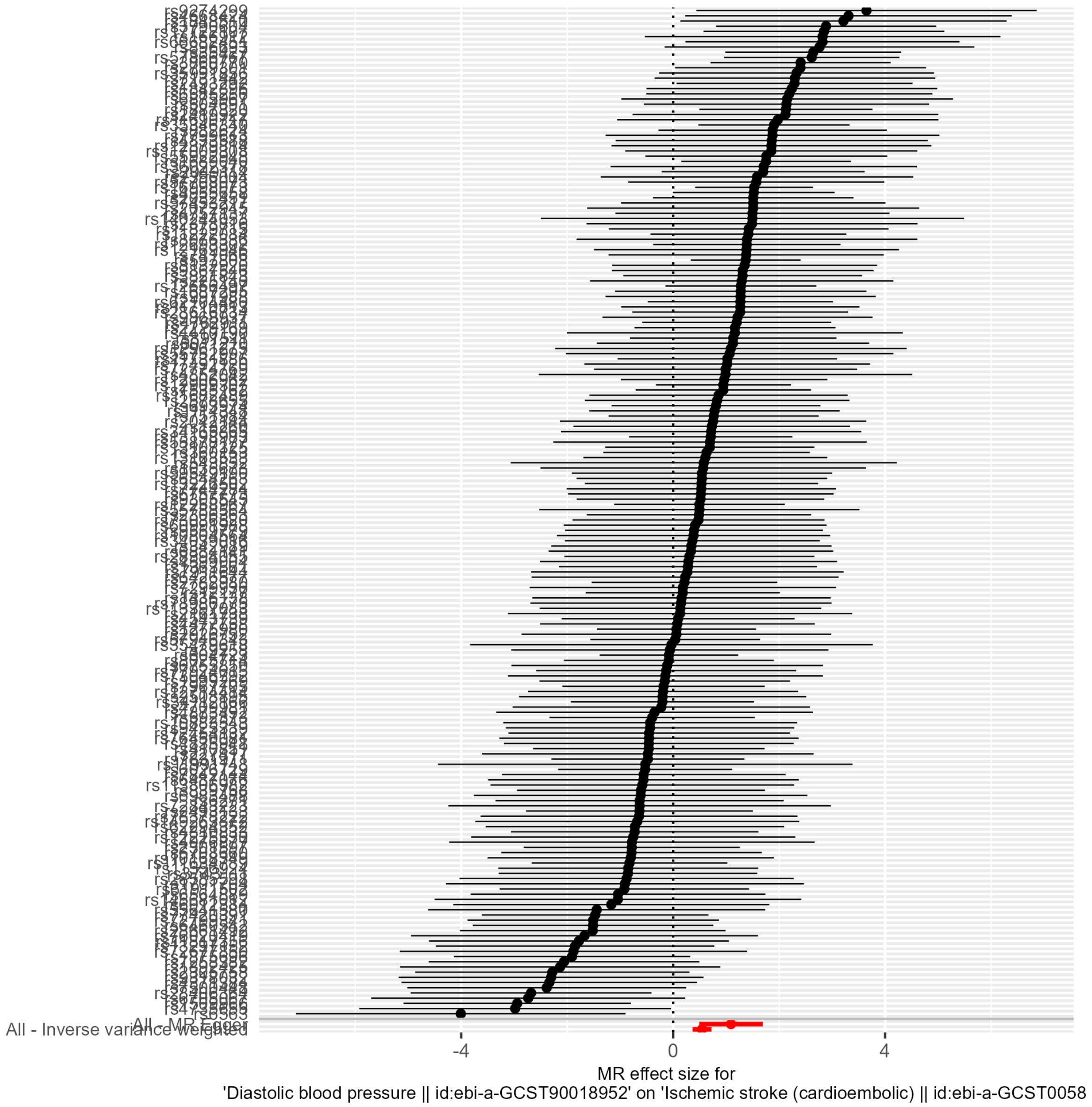

DBP-IS forest plot

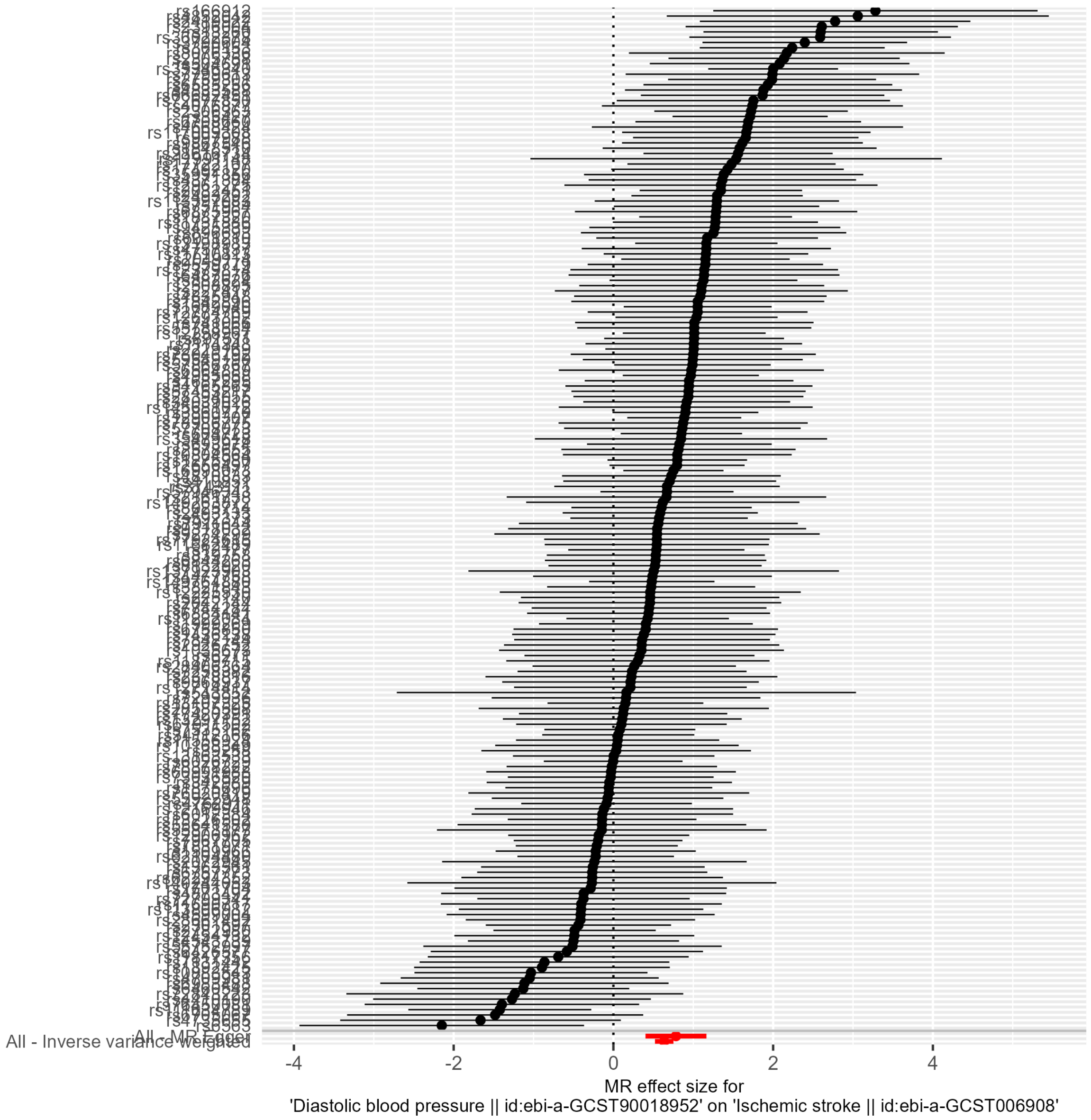

DBP-LS forest plot

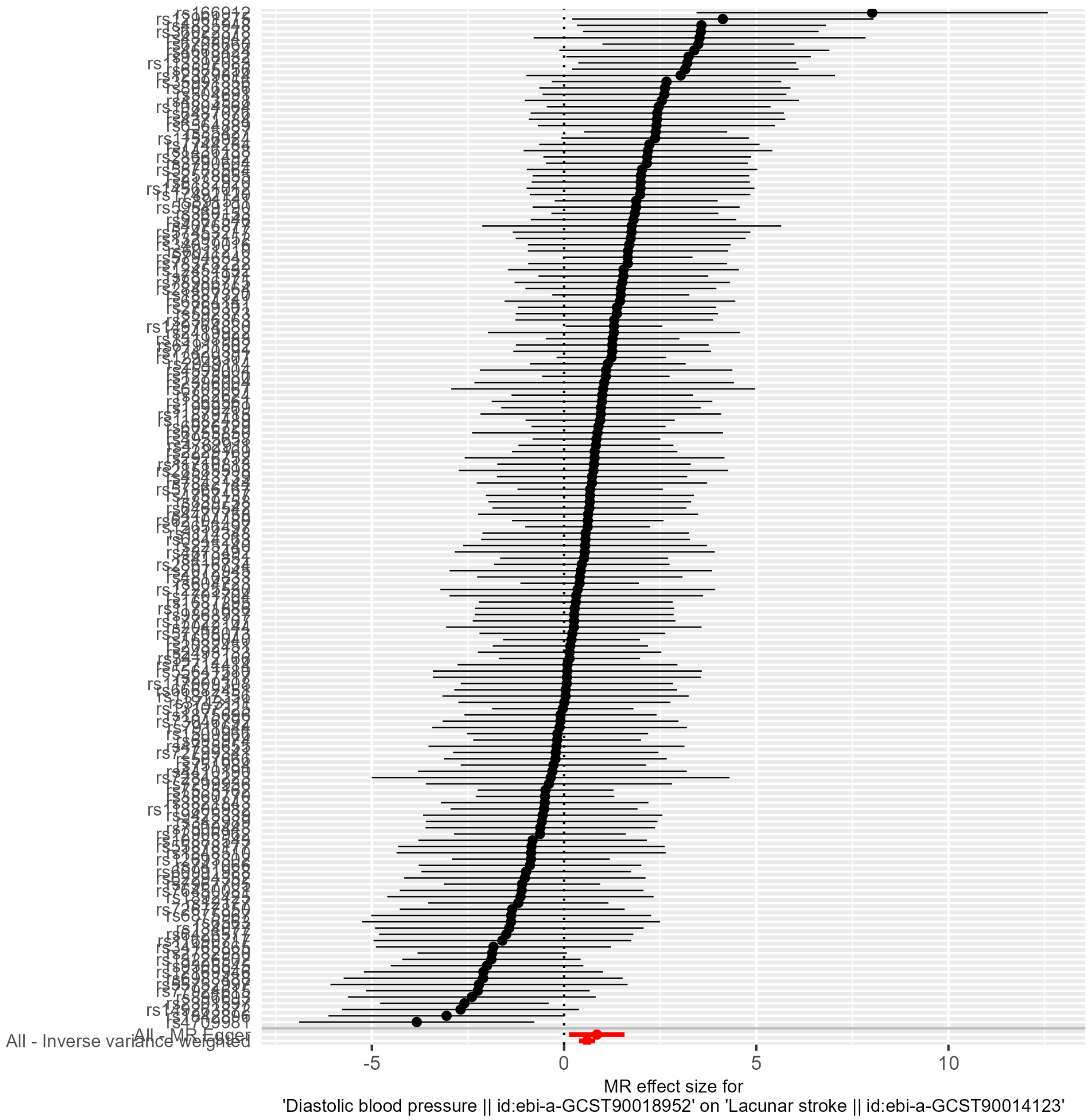

SBP-LAS forest plot

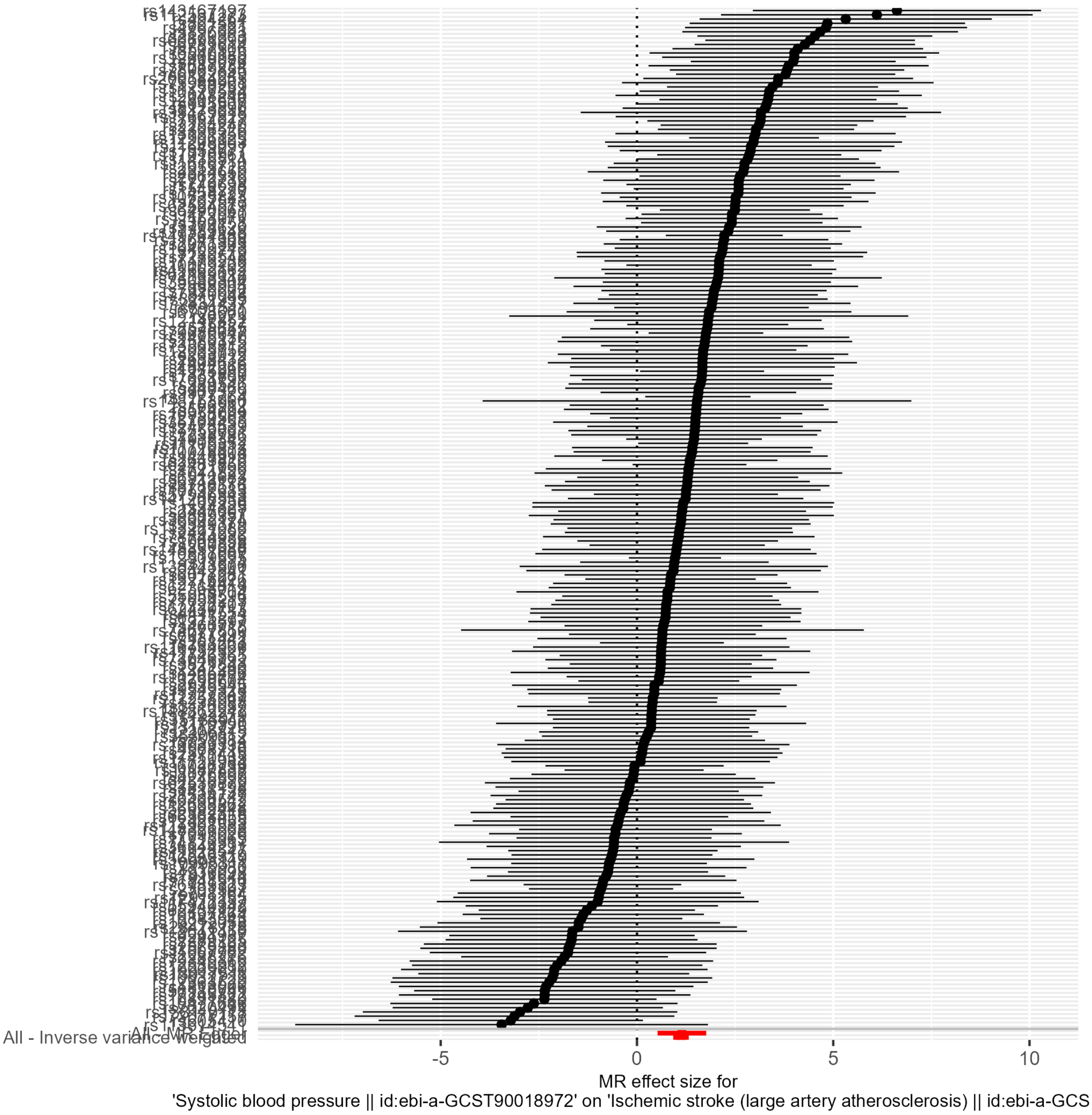

SBP-SVS forest plot

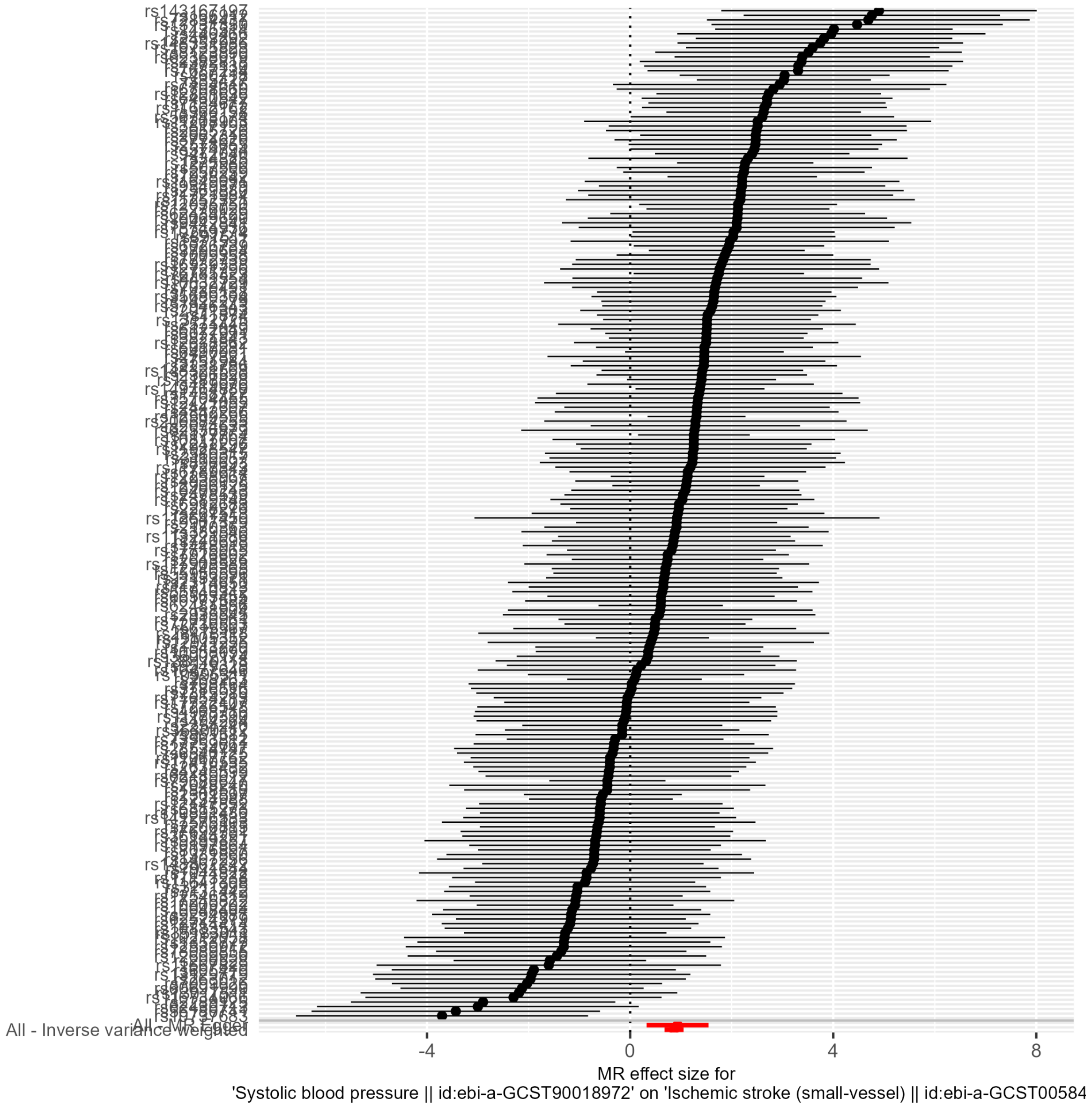

SBP-CES forest plot

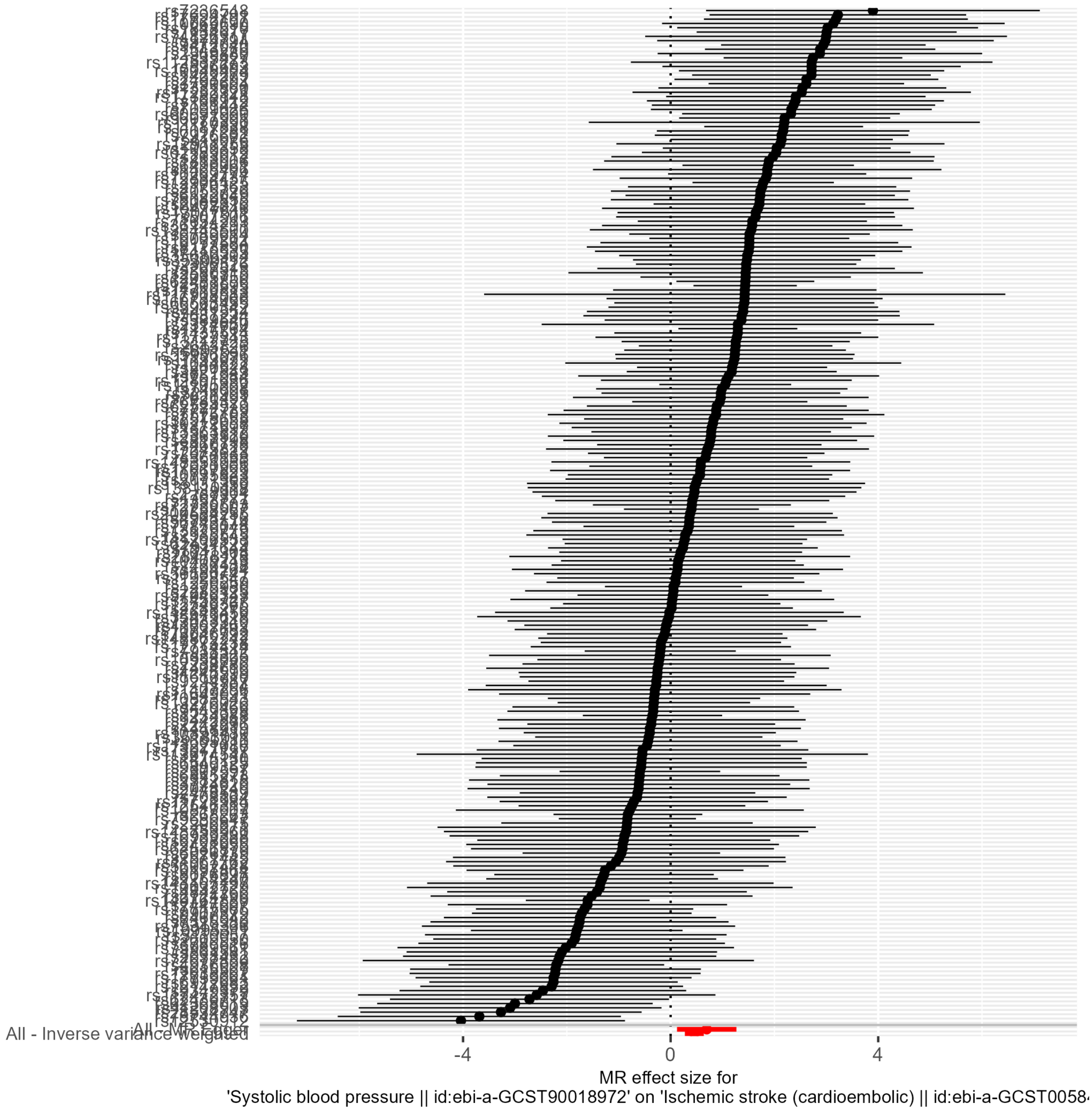

SBP-IS forest plot

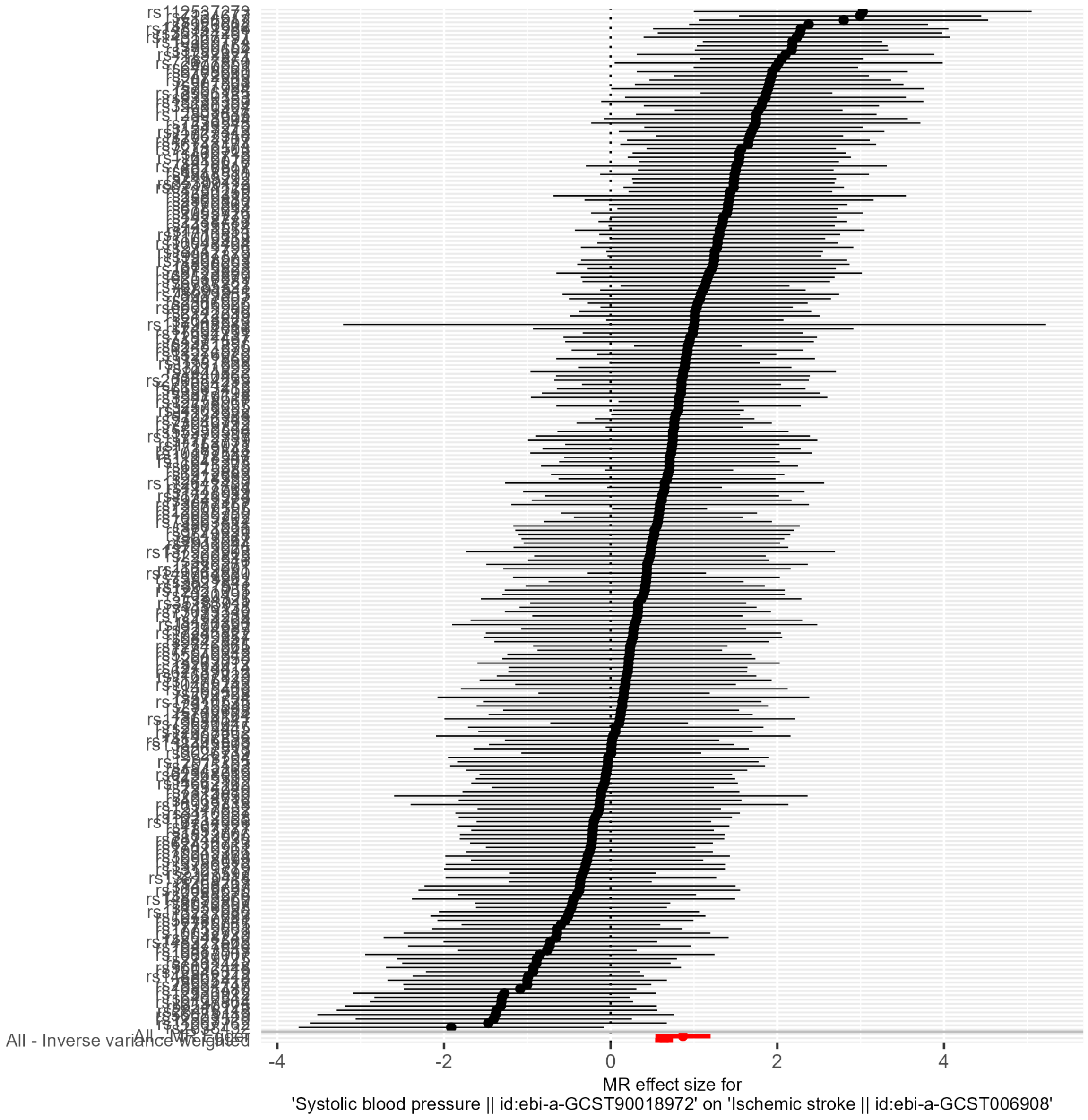

SBP-LS forest plot

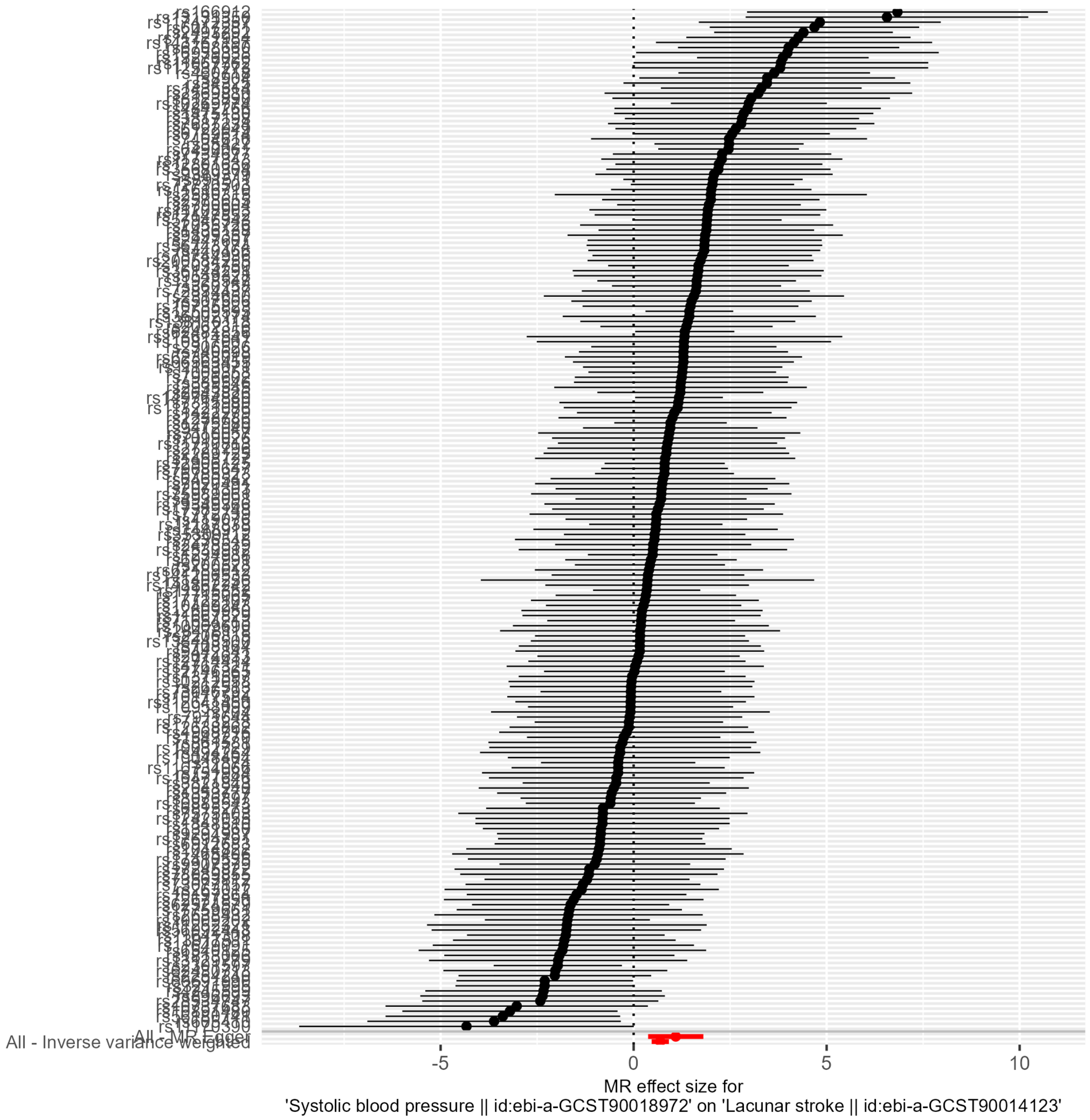

EH-LAS forest plot

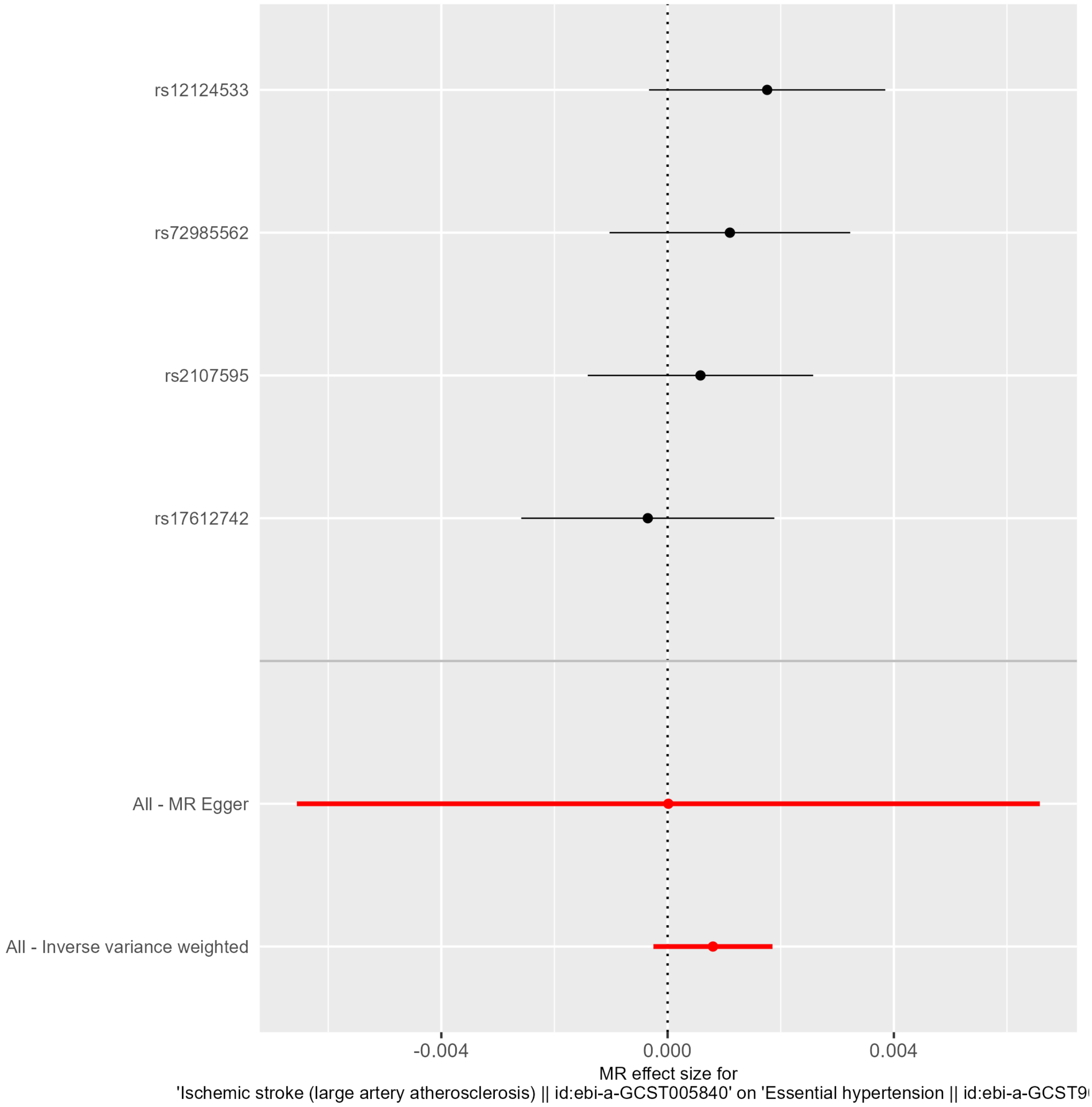

EH-SVS forest plot

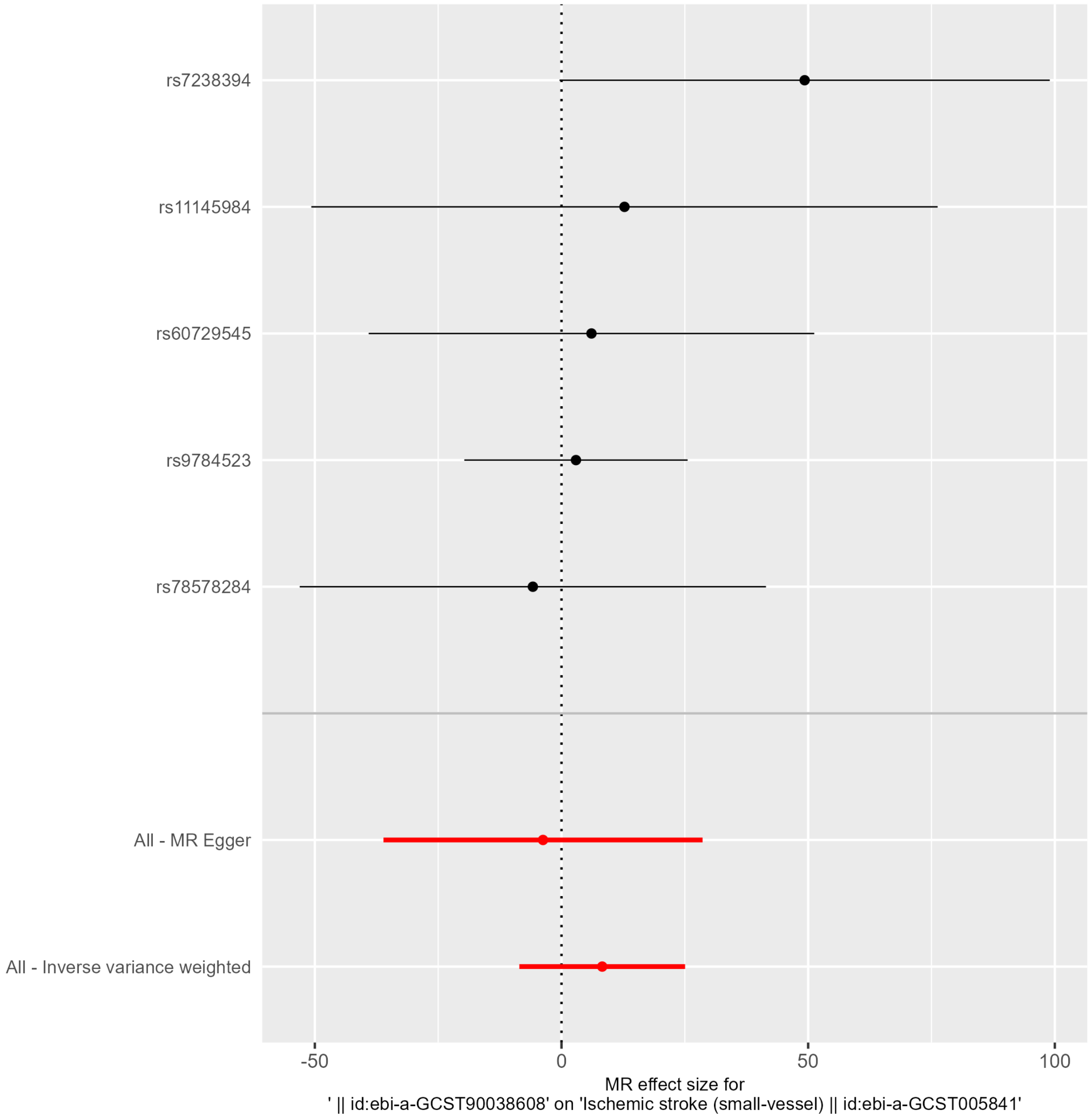

EH-CES forest plot

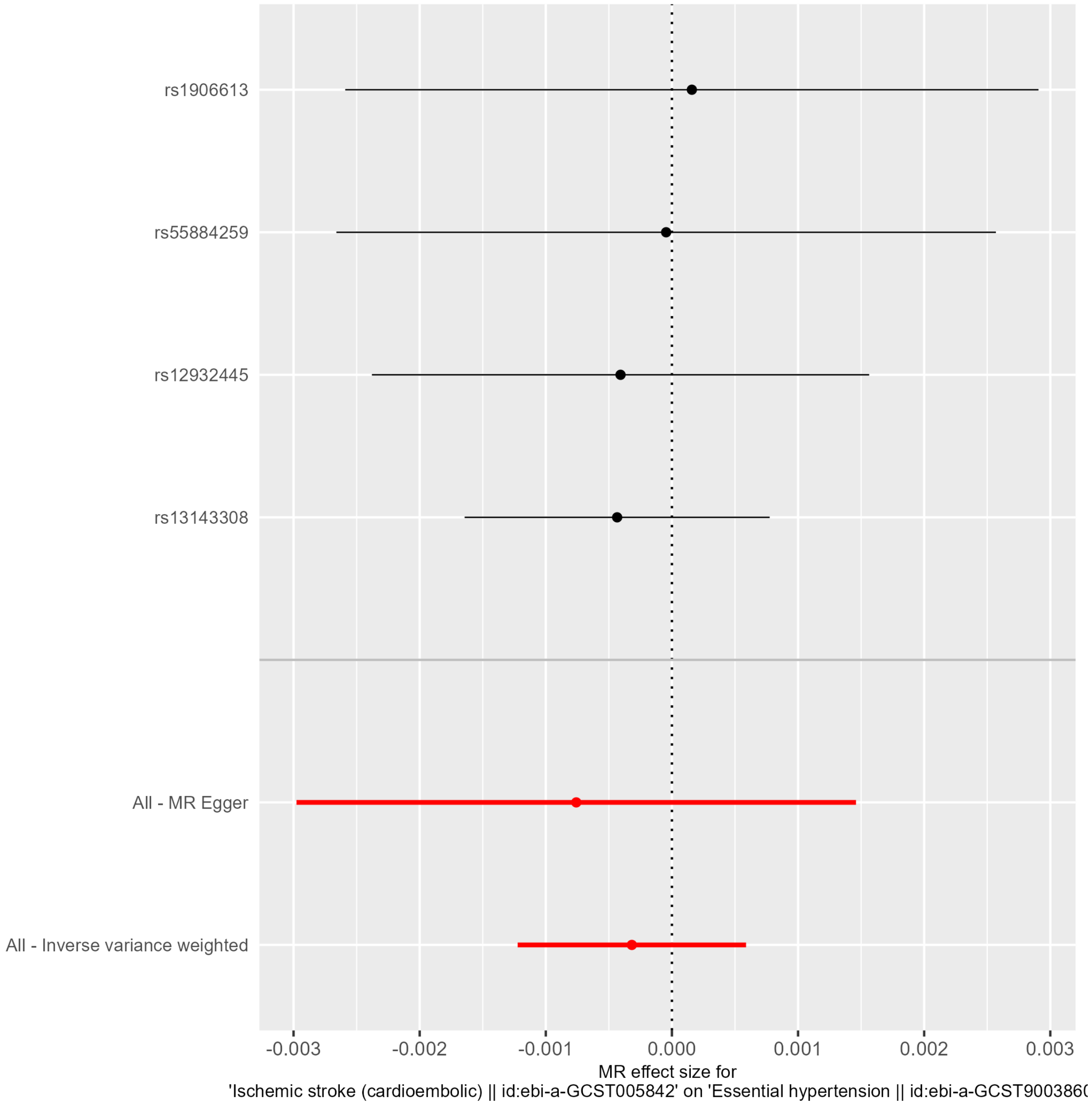

EH-IS forest plot

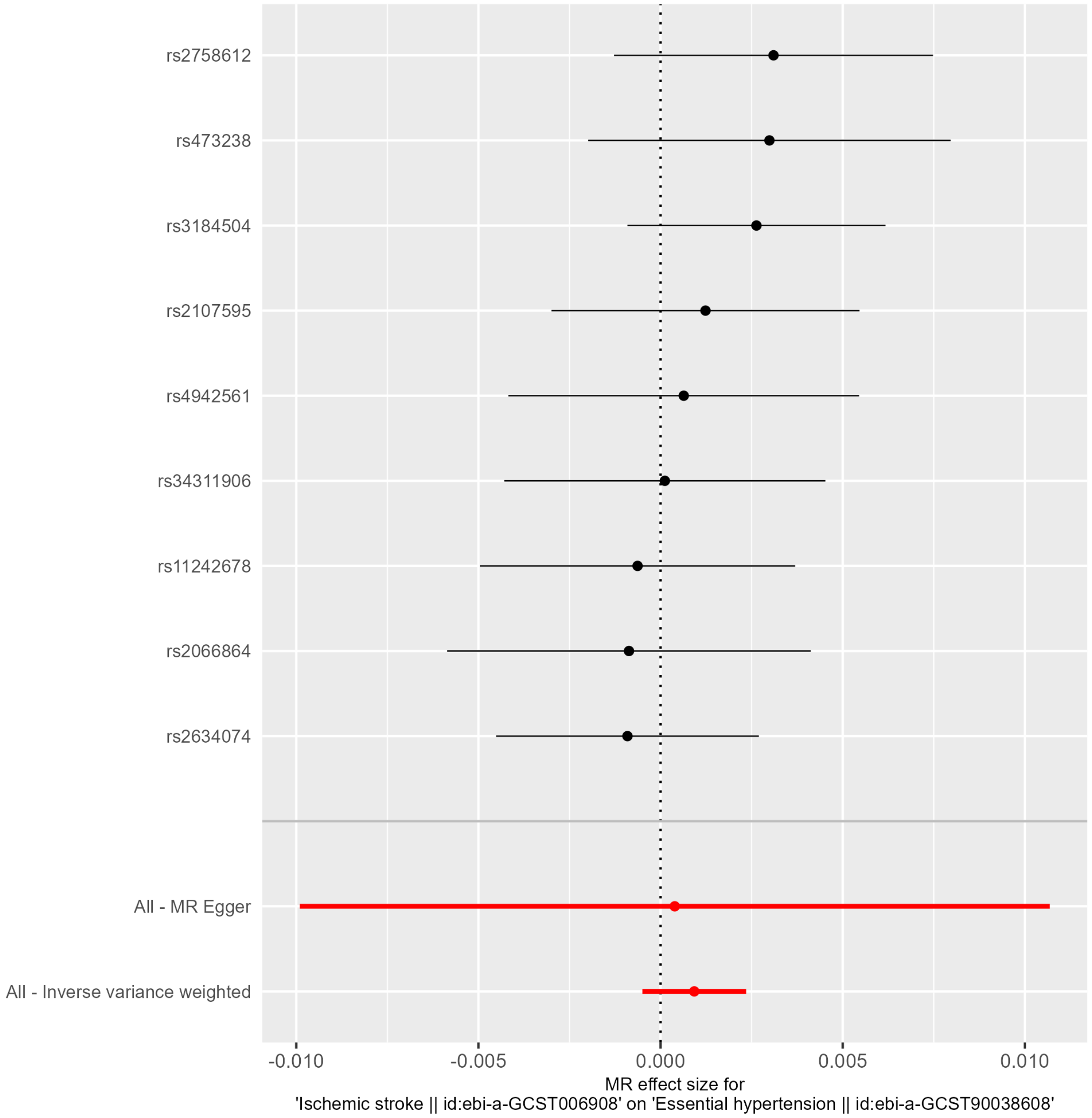

EH-LS forest plot

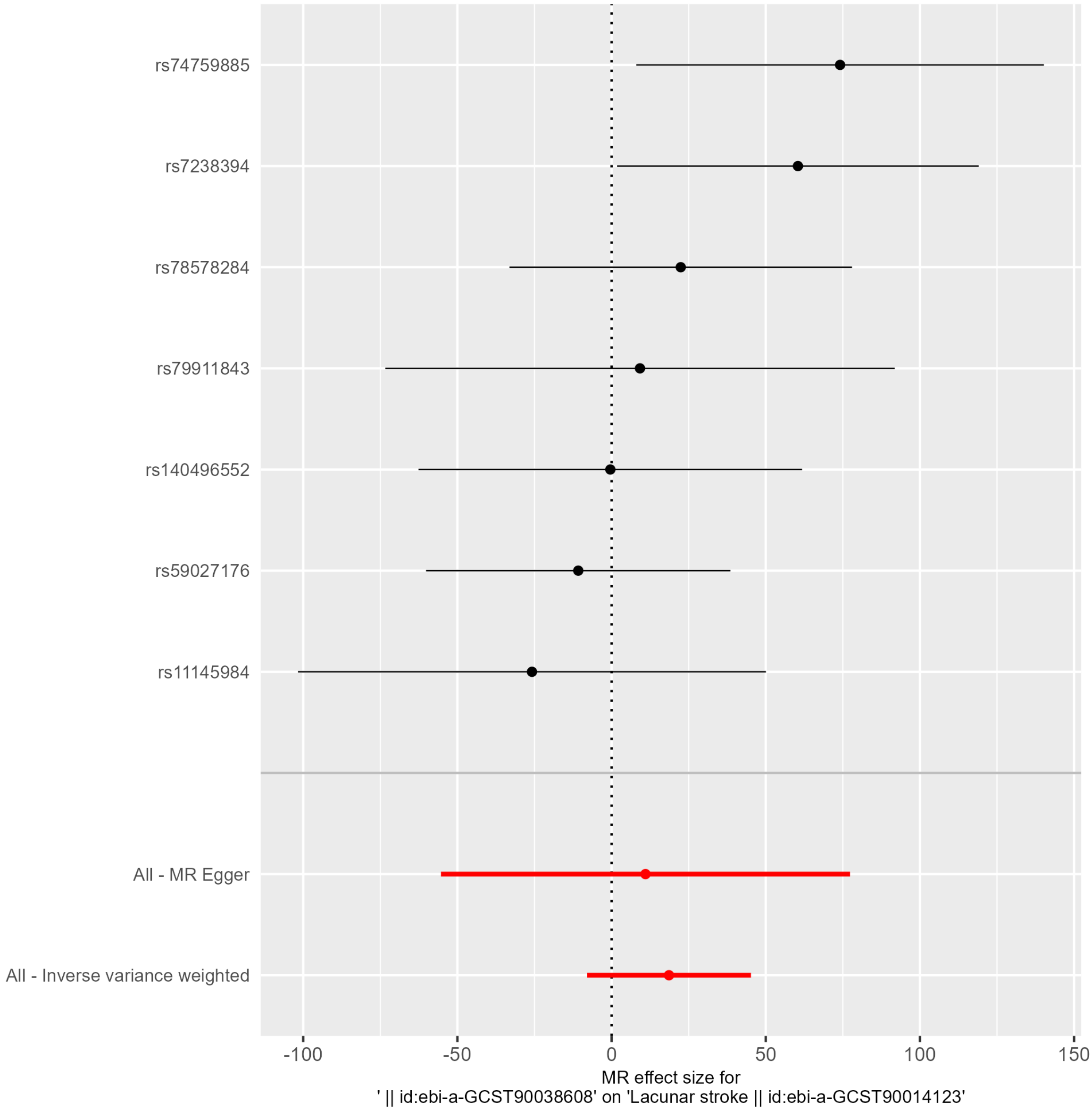

DBP-LAS funnel plot

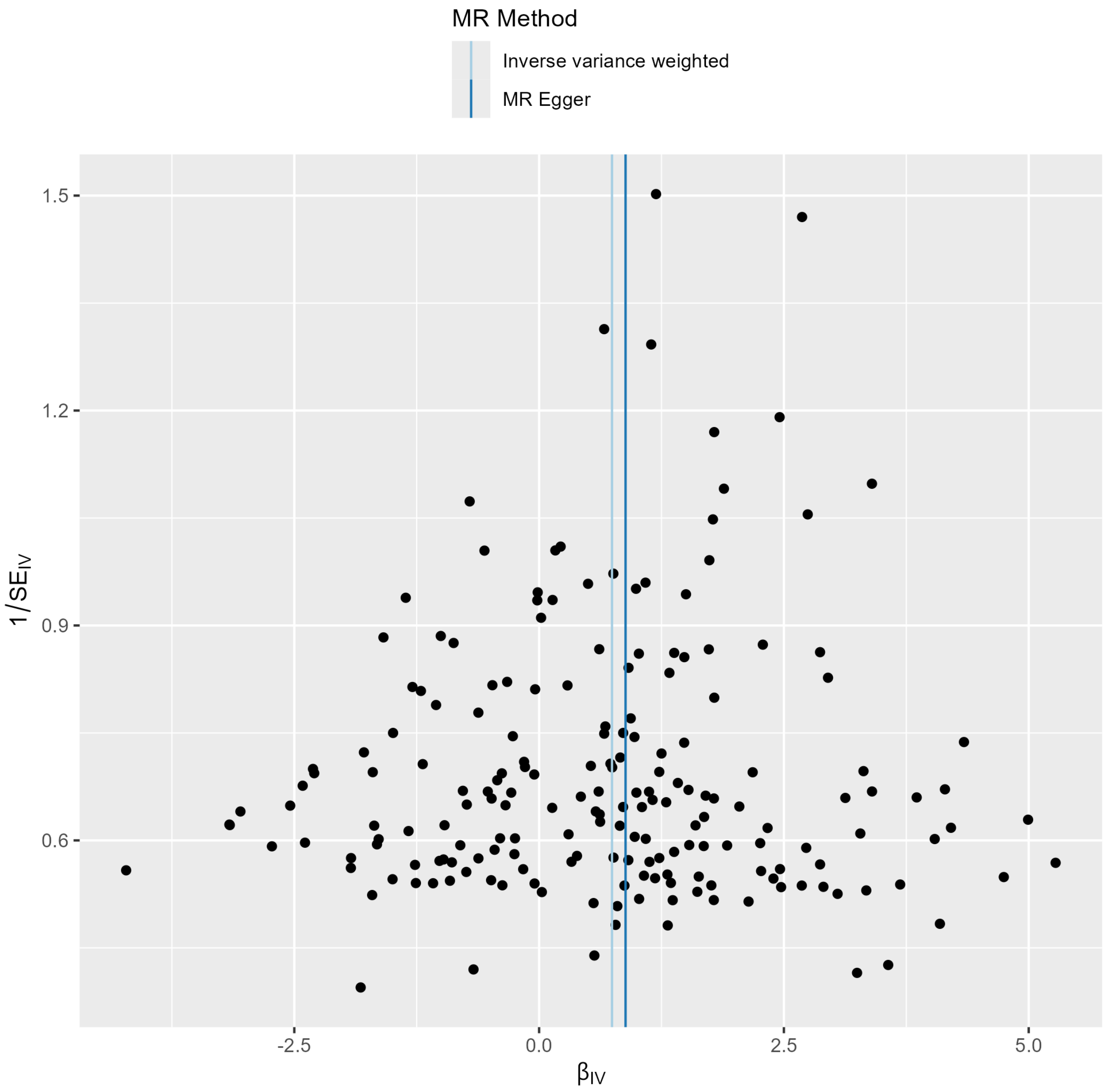

DBP-SVS funnel plot

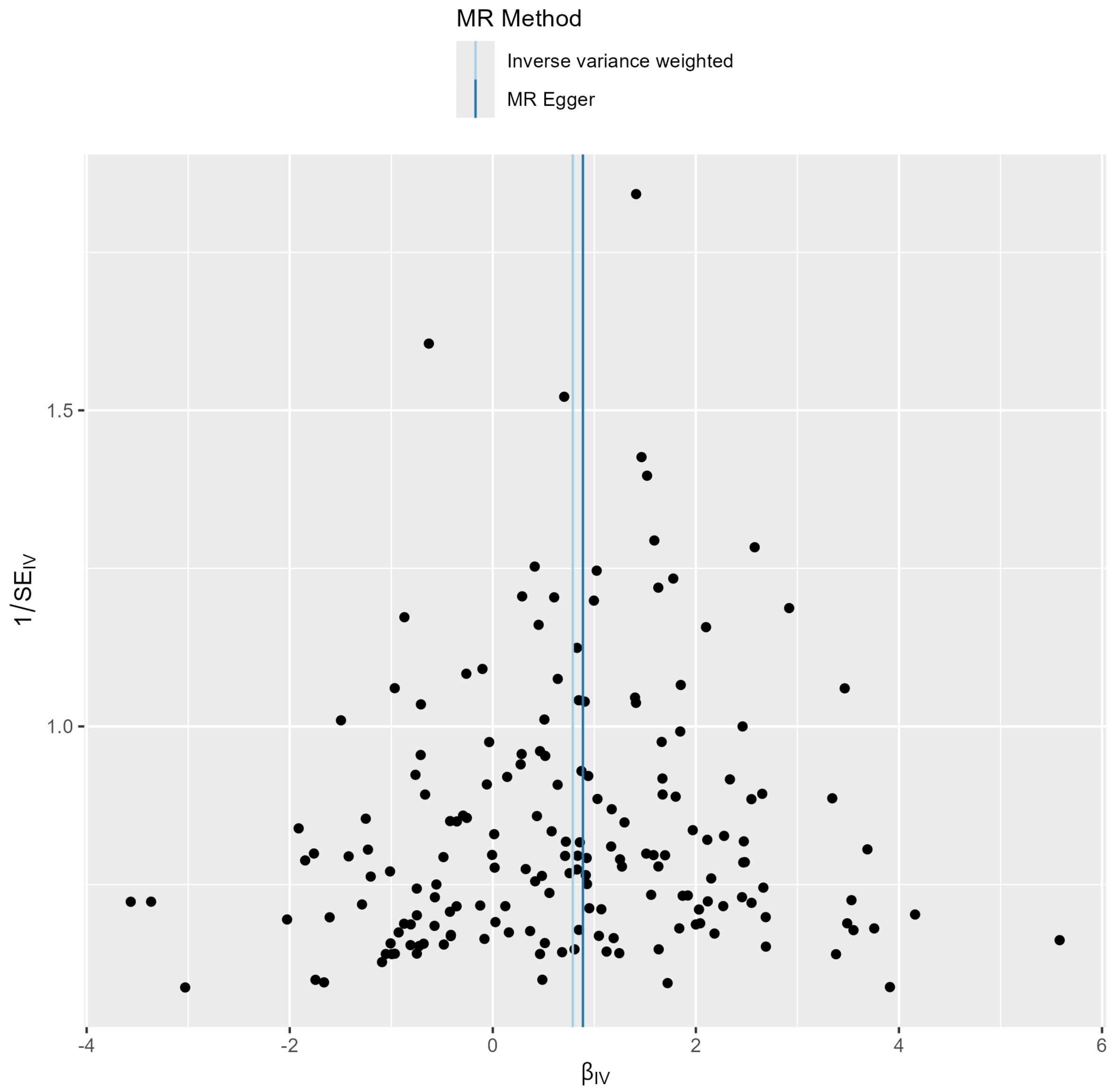

DBP-CES funnel plot

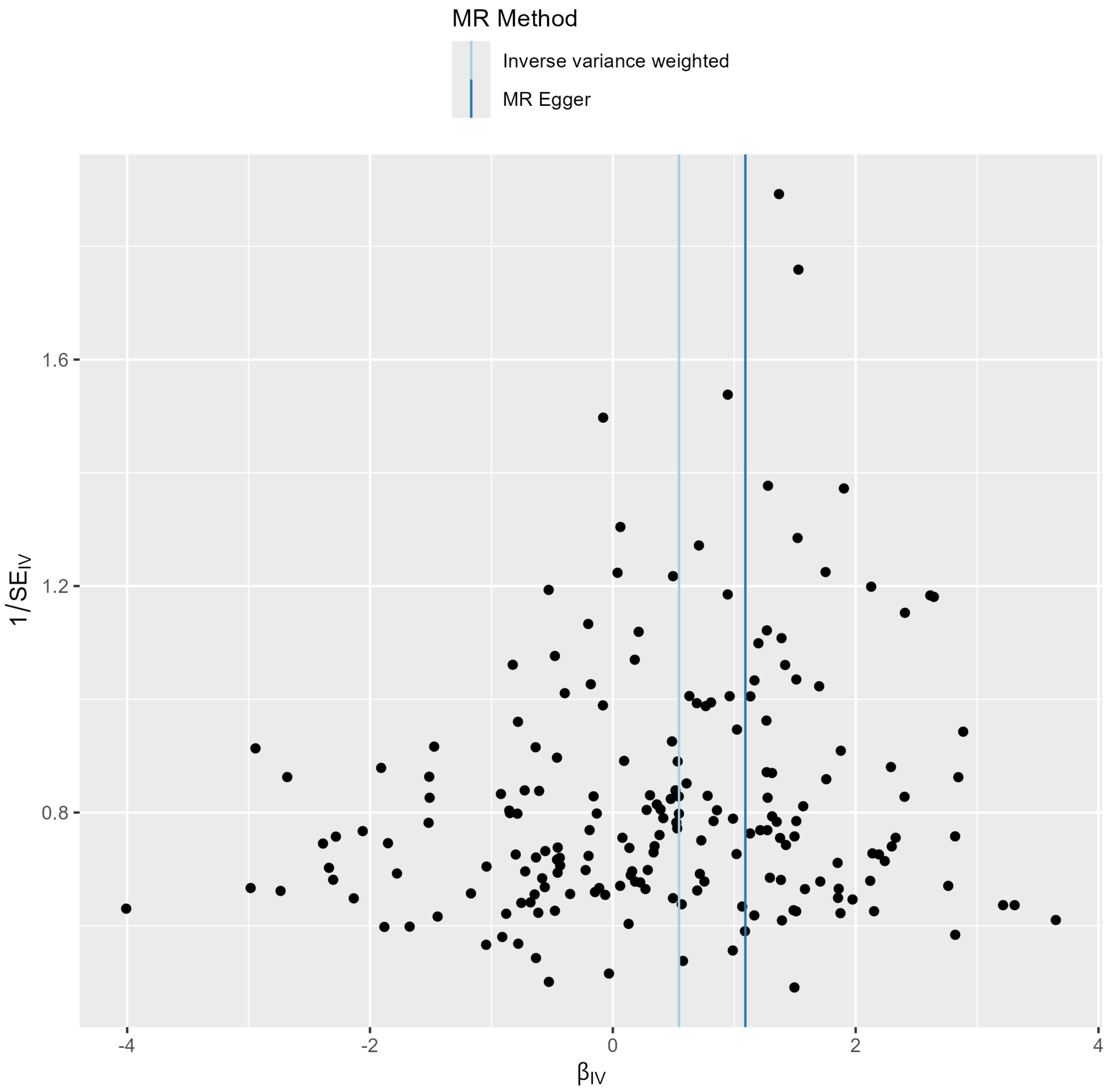

DBP-IS funnel plot

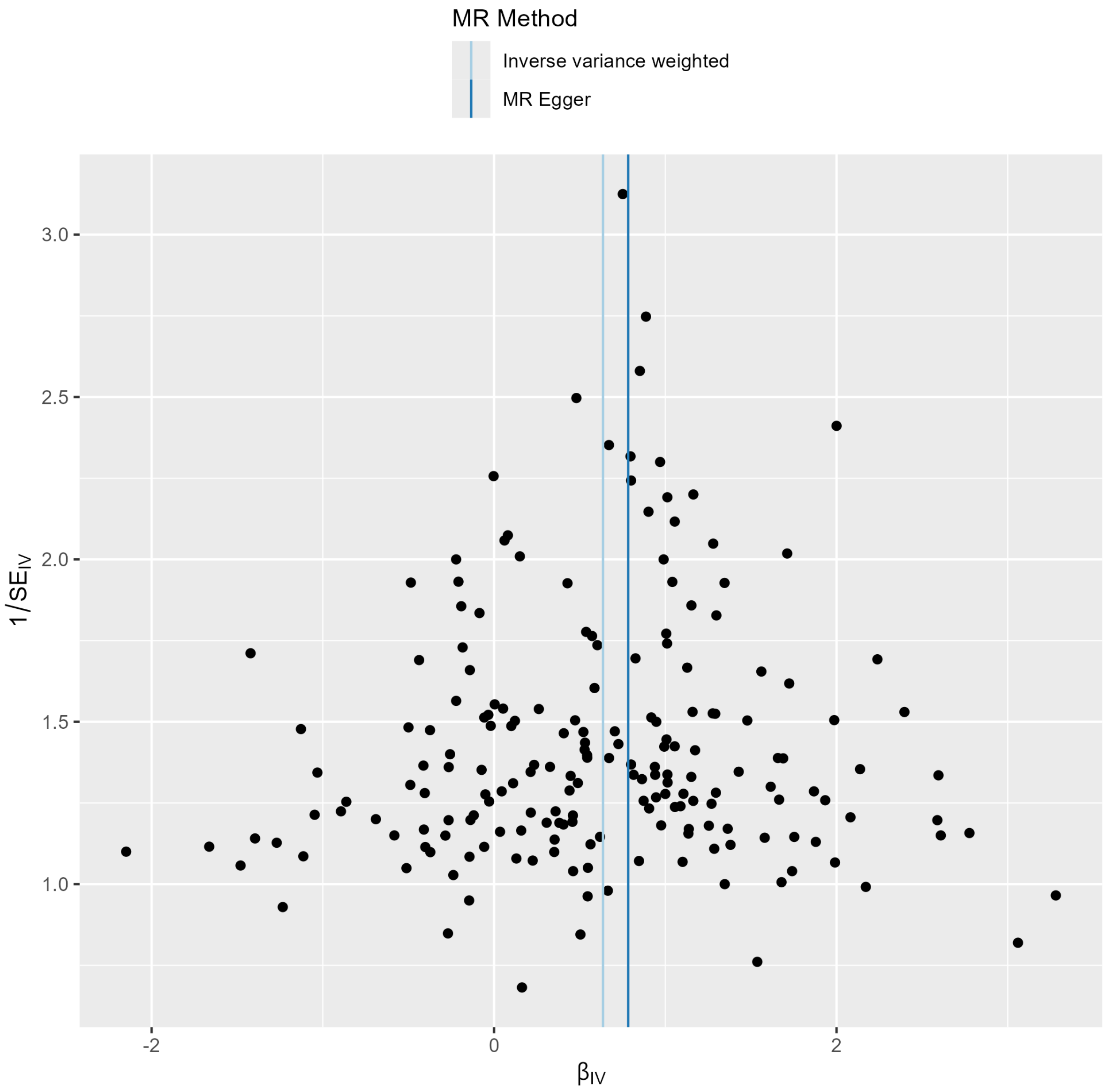

DBP-LS funnel plot

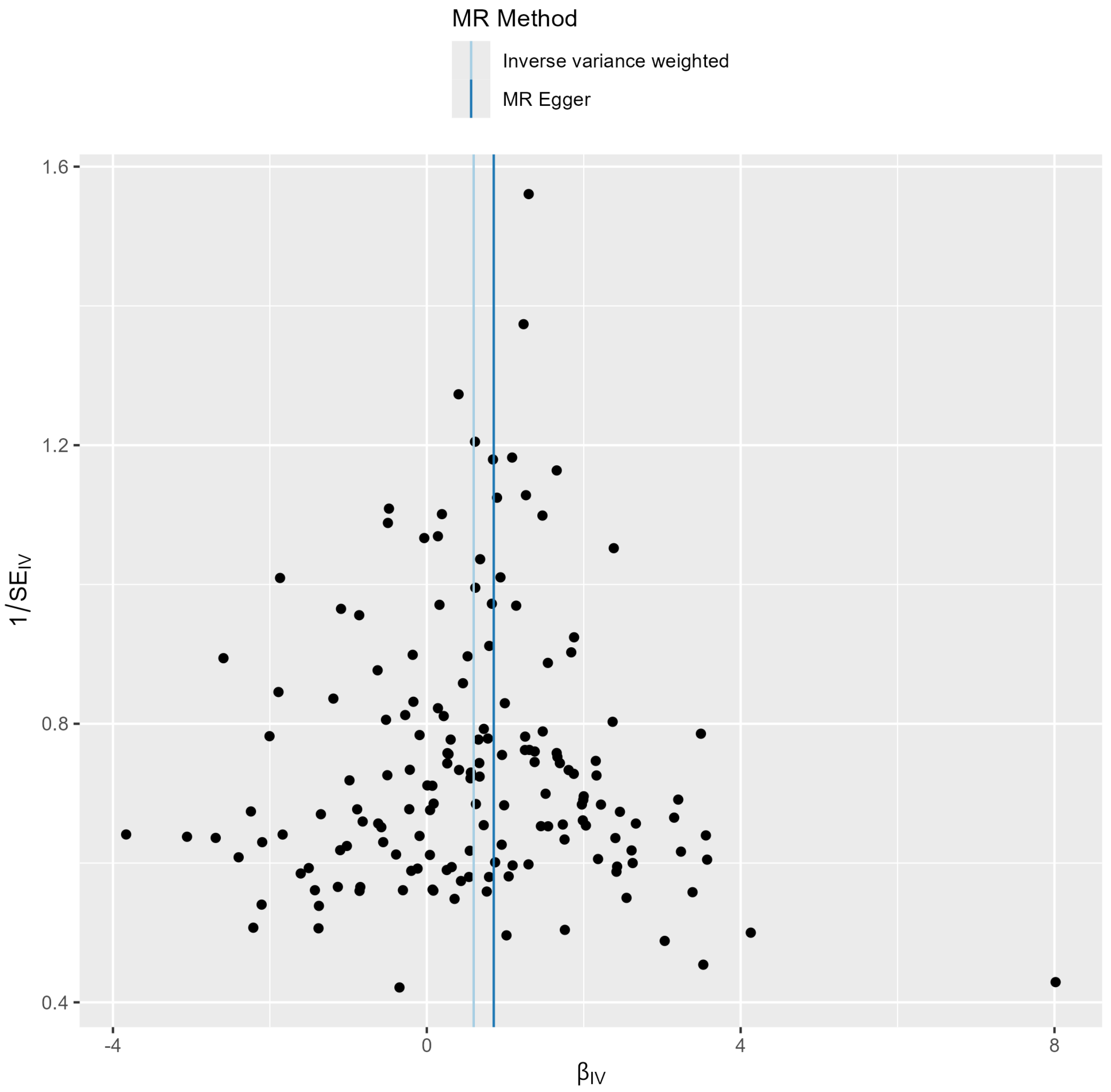

SBP-LAS funnel plot

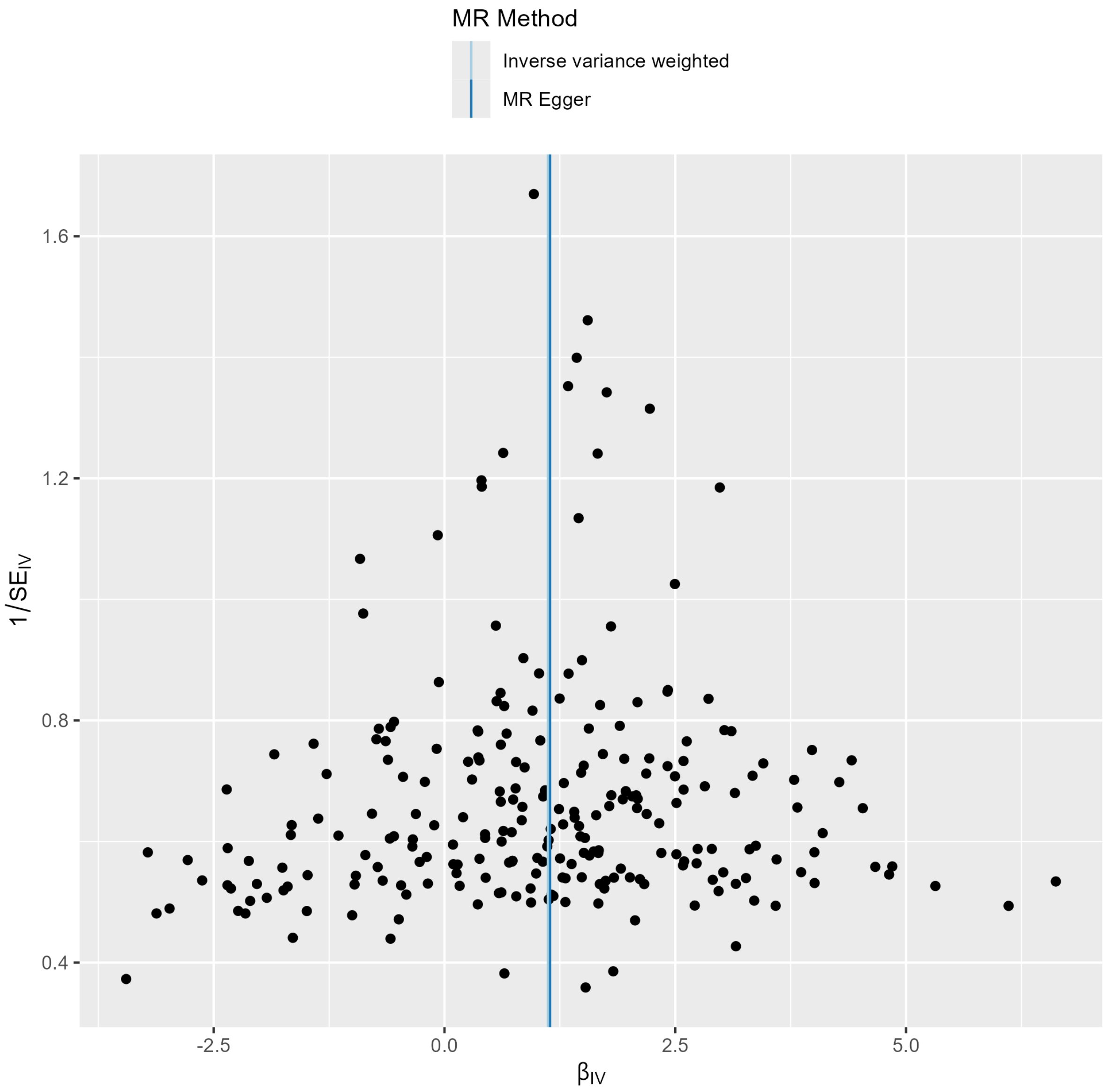

SBP-SVS funnel plot

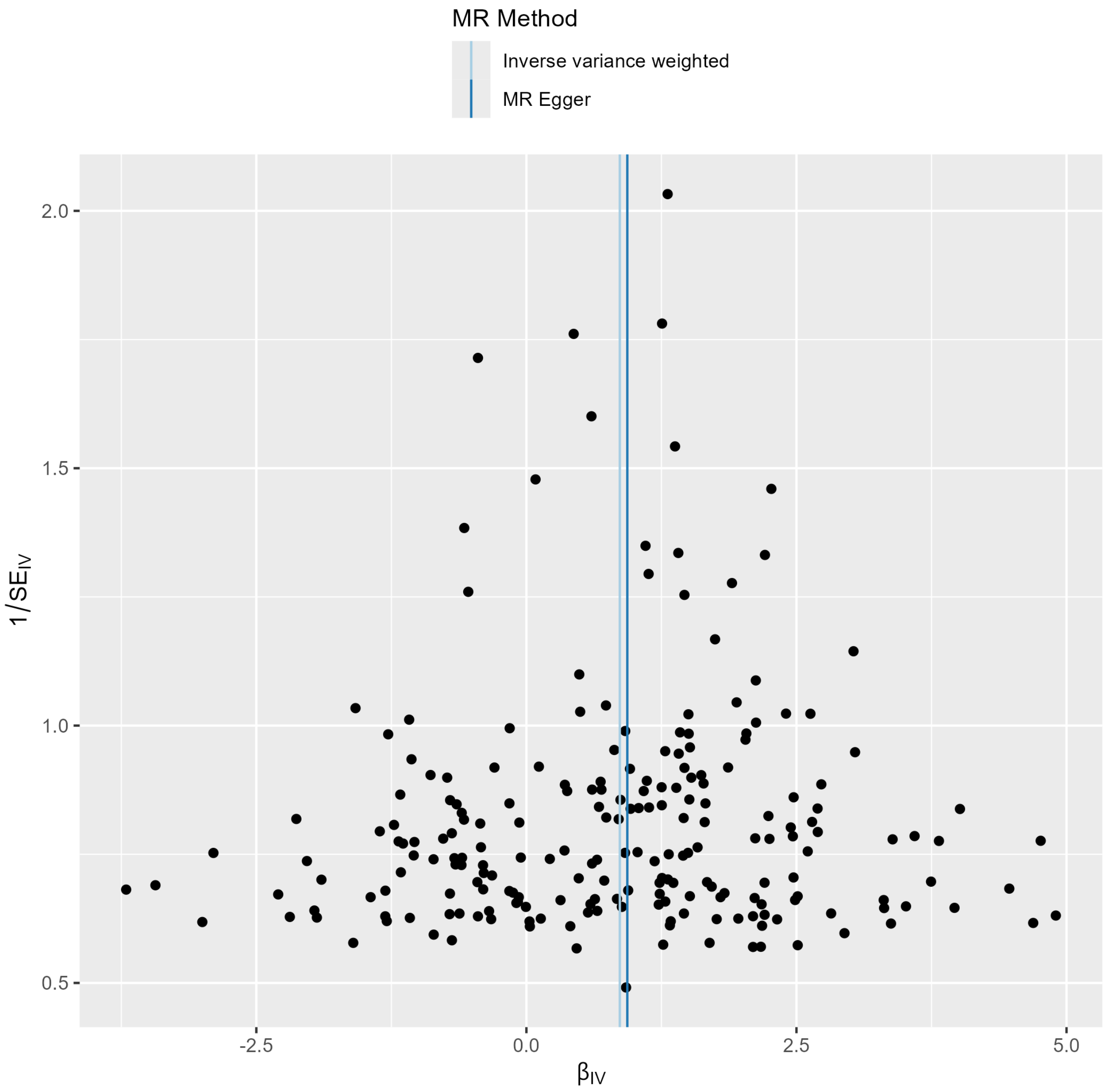

SBP-CES funnel plot

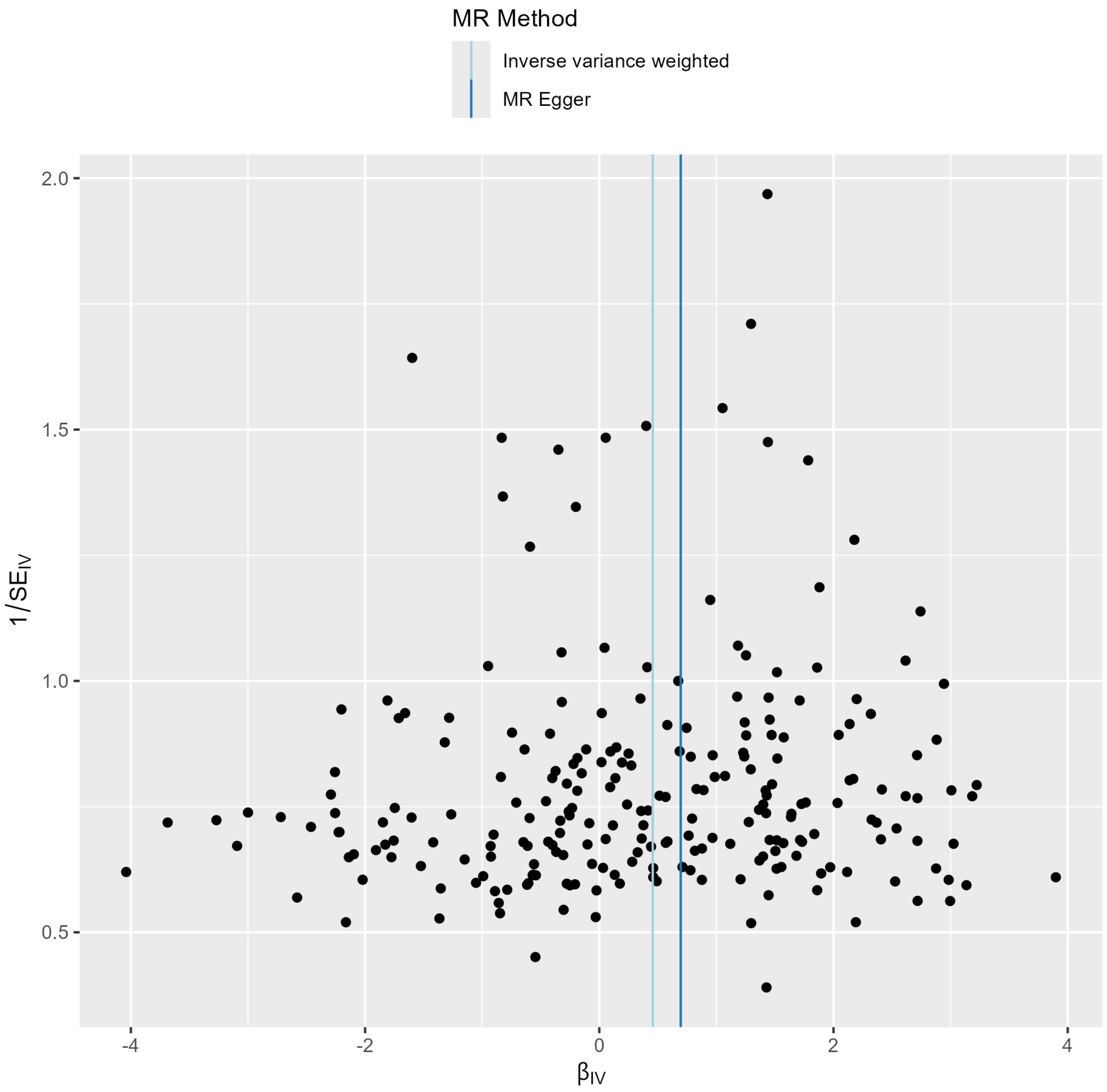

SBP-IS funnel plot

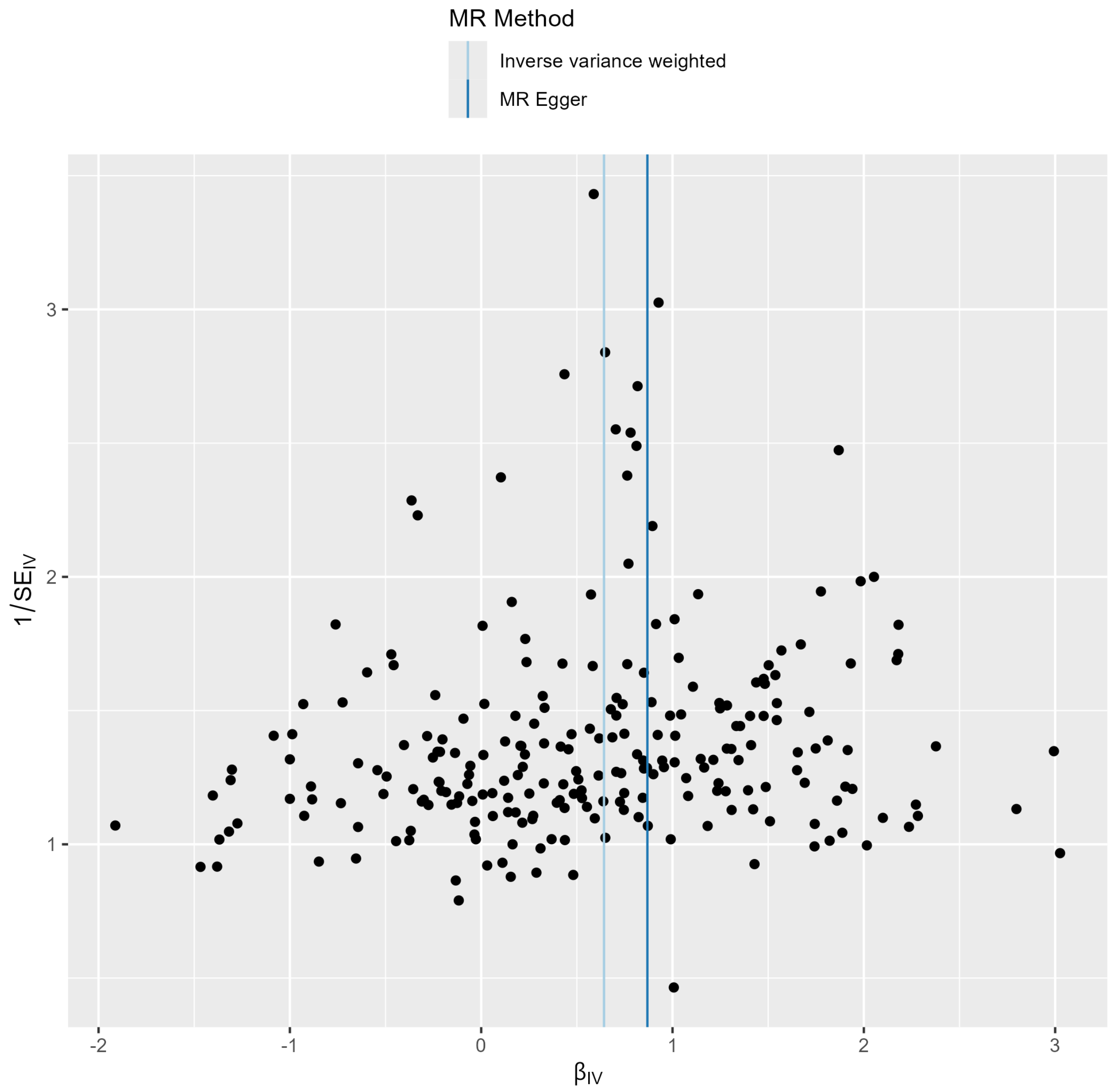

SBP-LS funnel plot

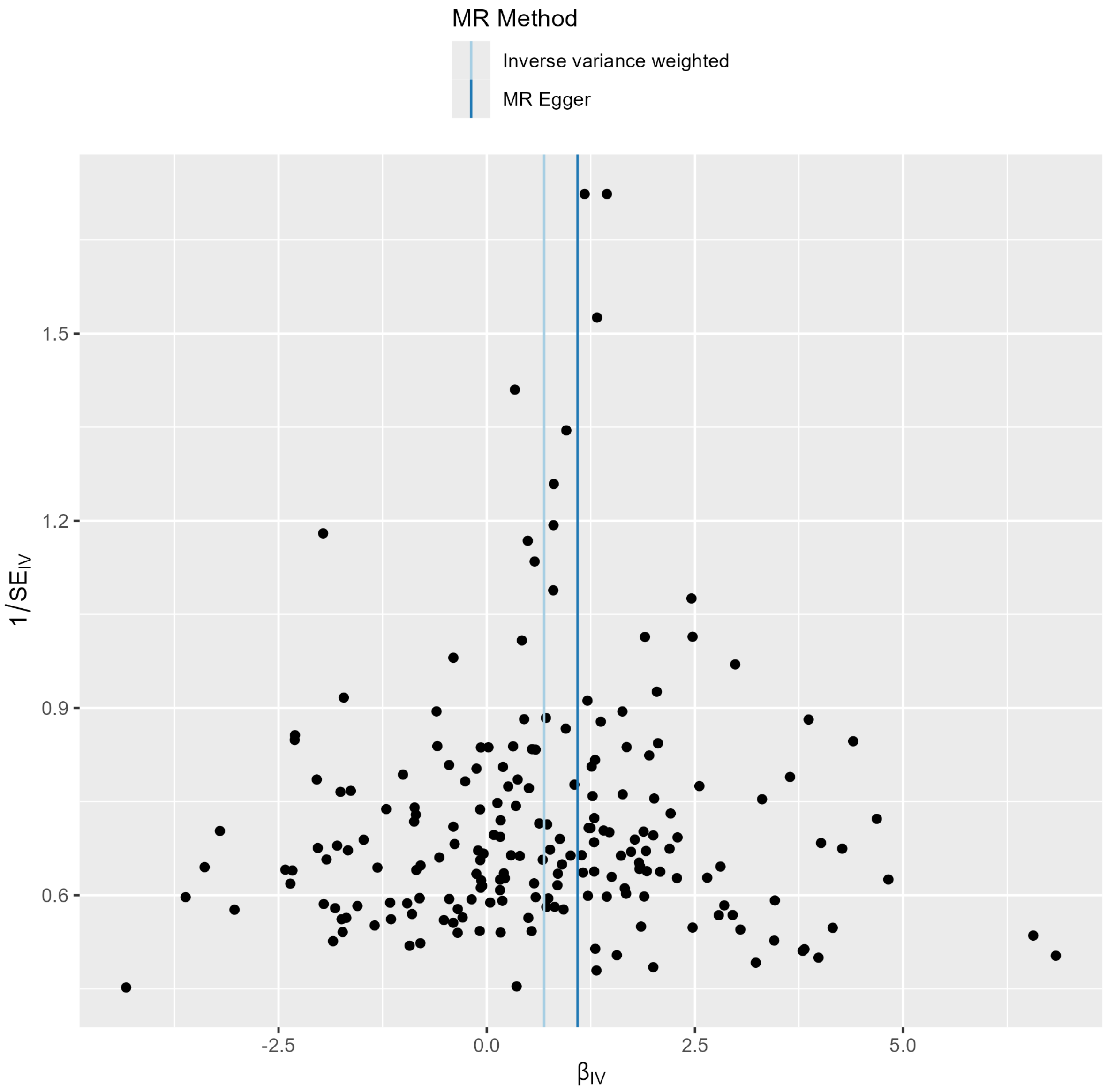

EH-LAS funnel plot

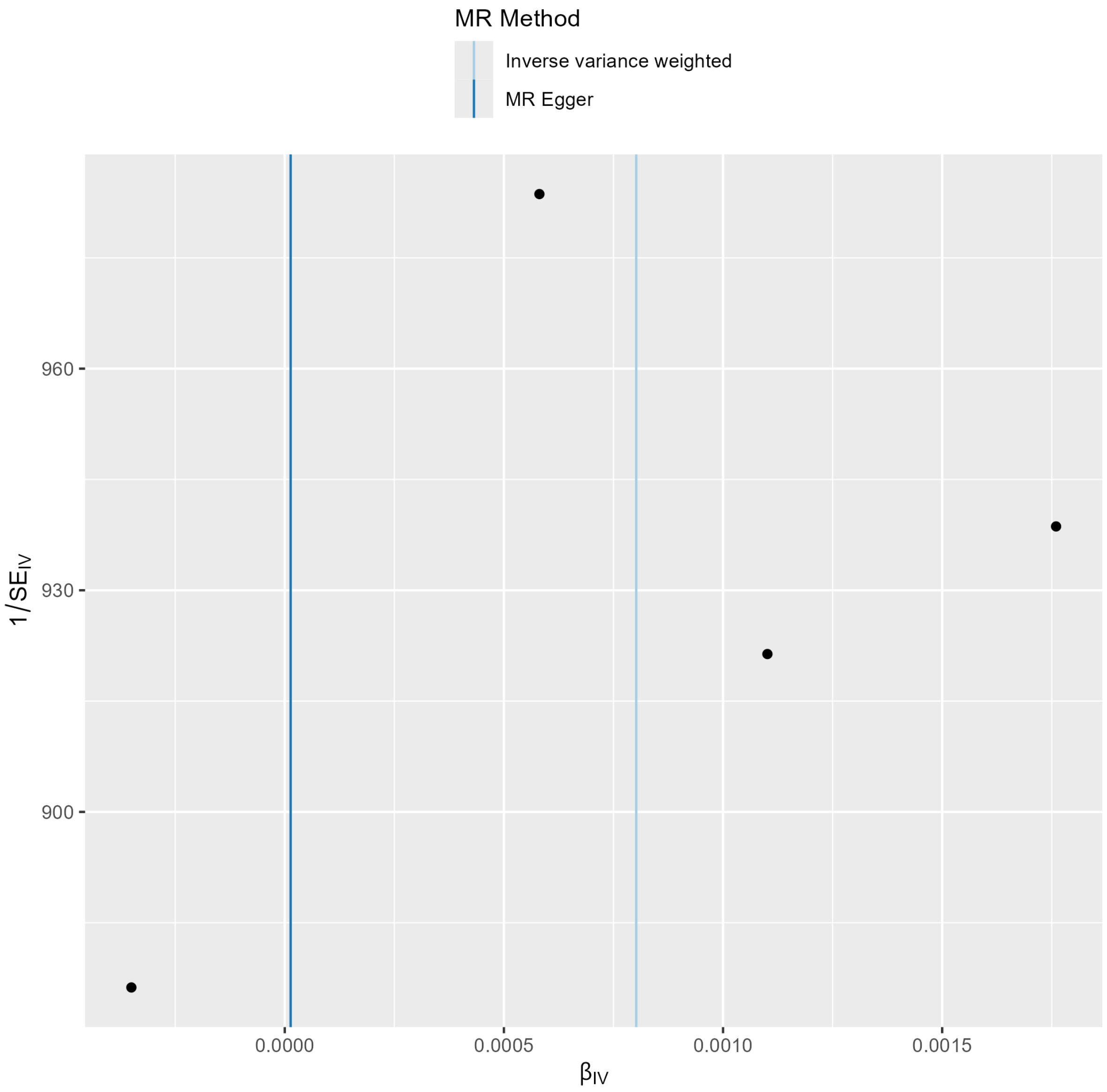

EH-SVS funnel plot

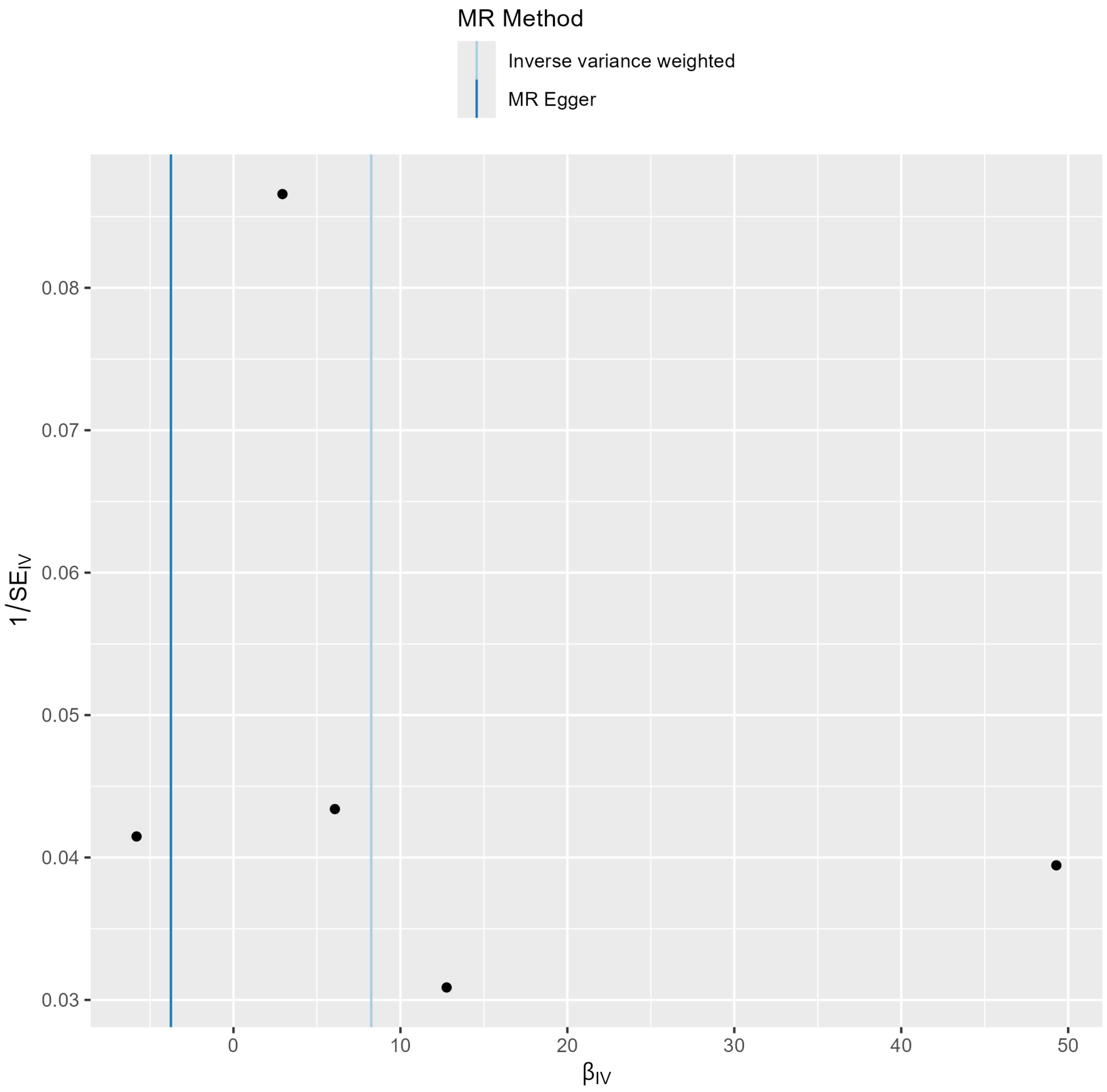

EH-CES funnel plot

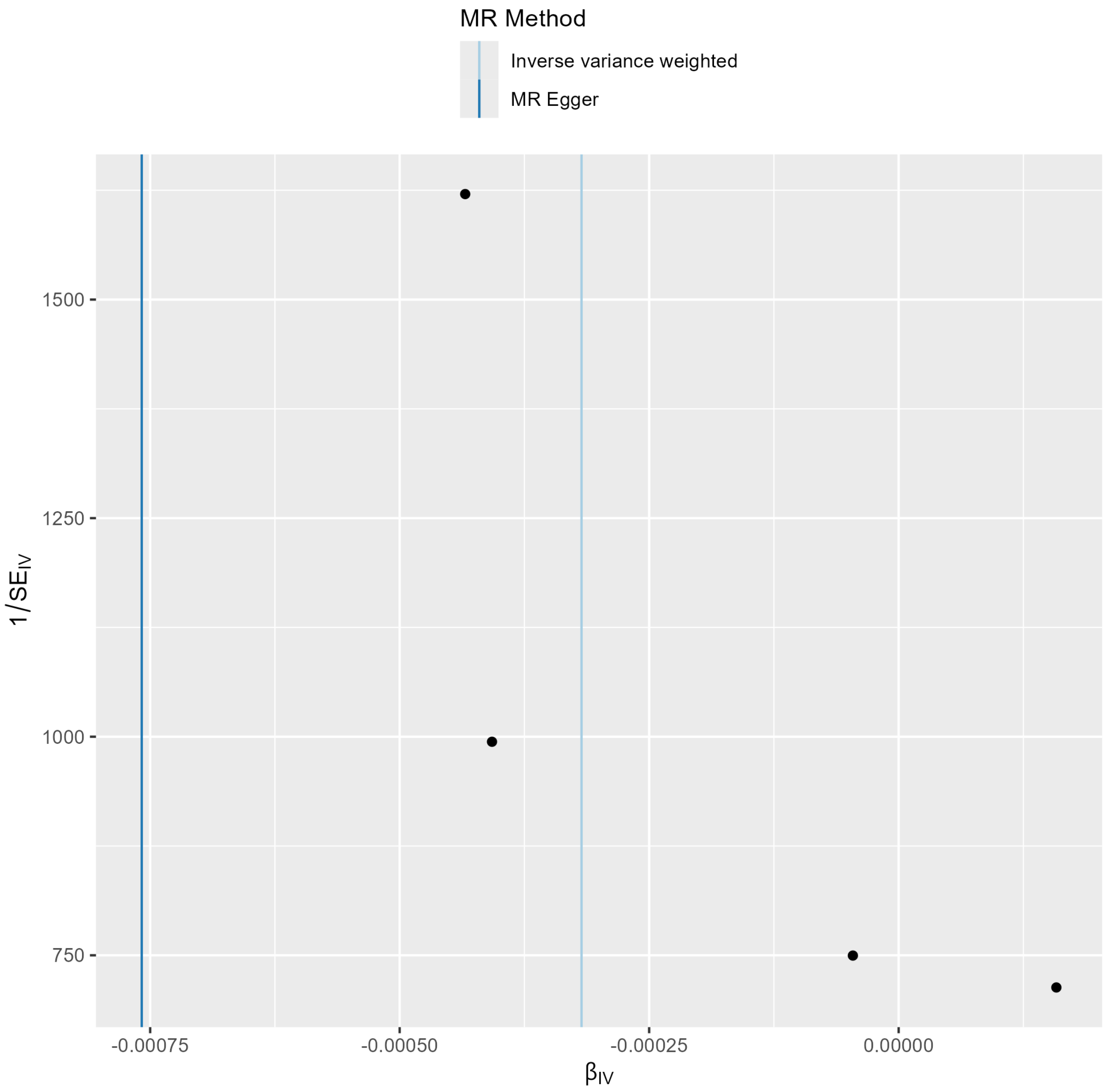

EH-IS funnel plot

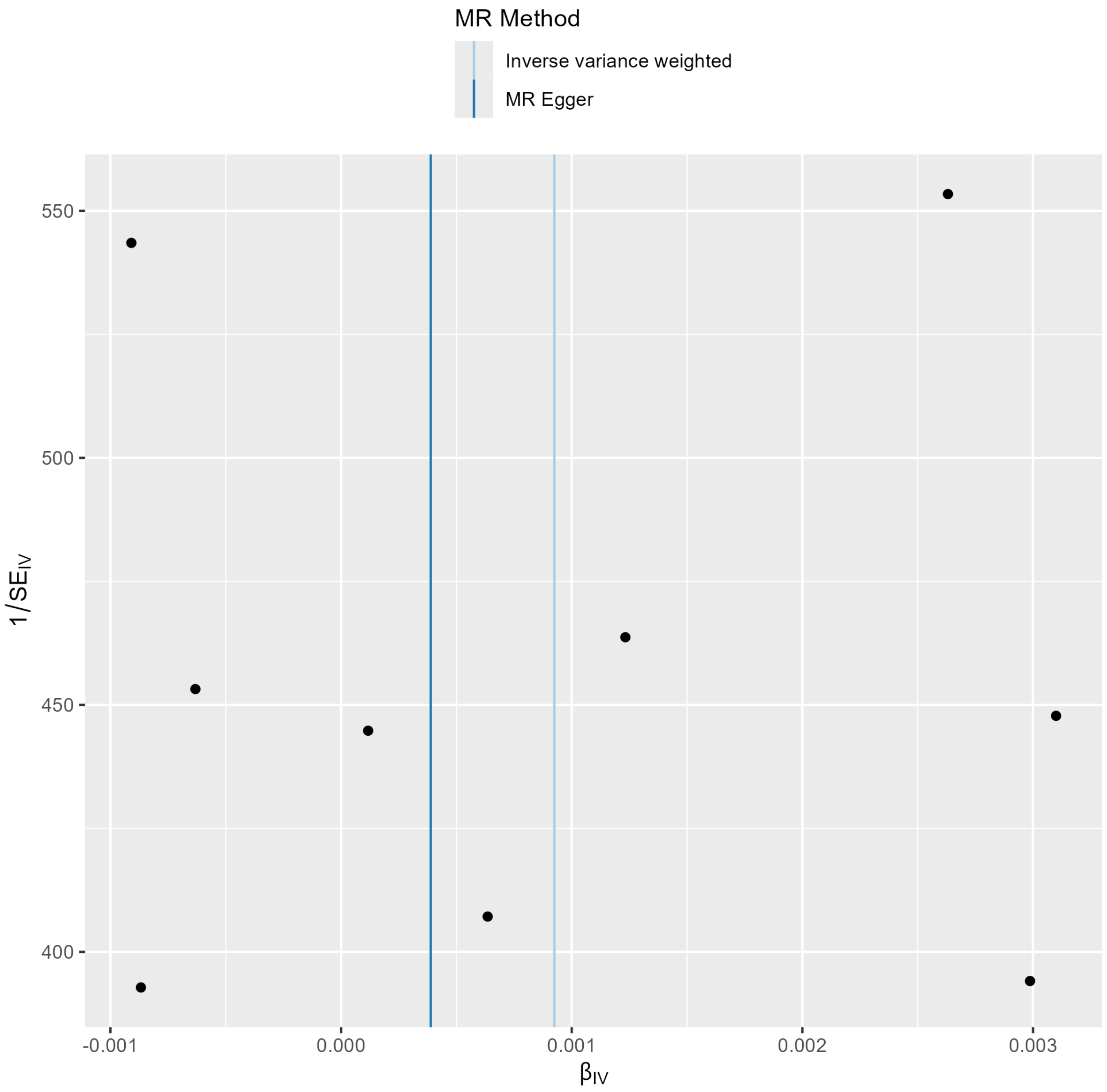

EH-LS funnel plot

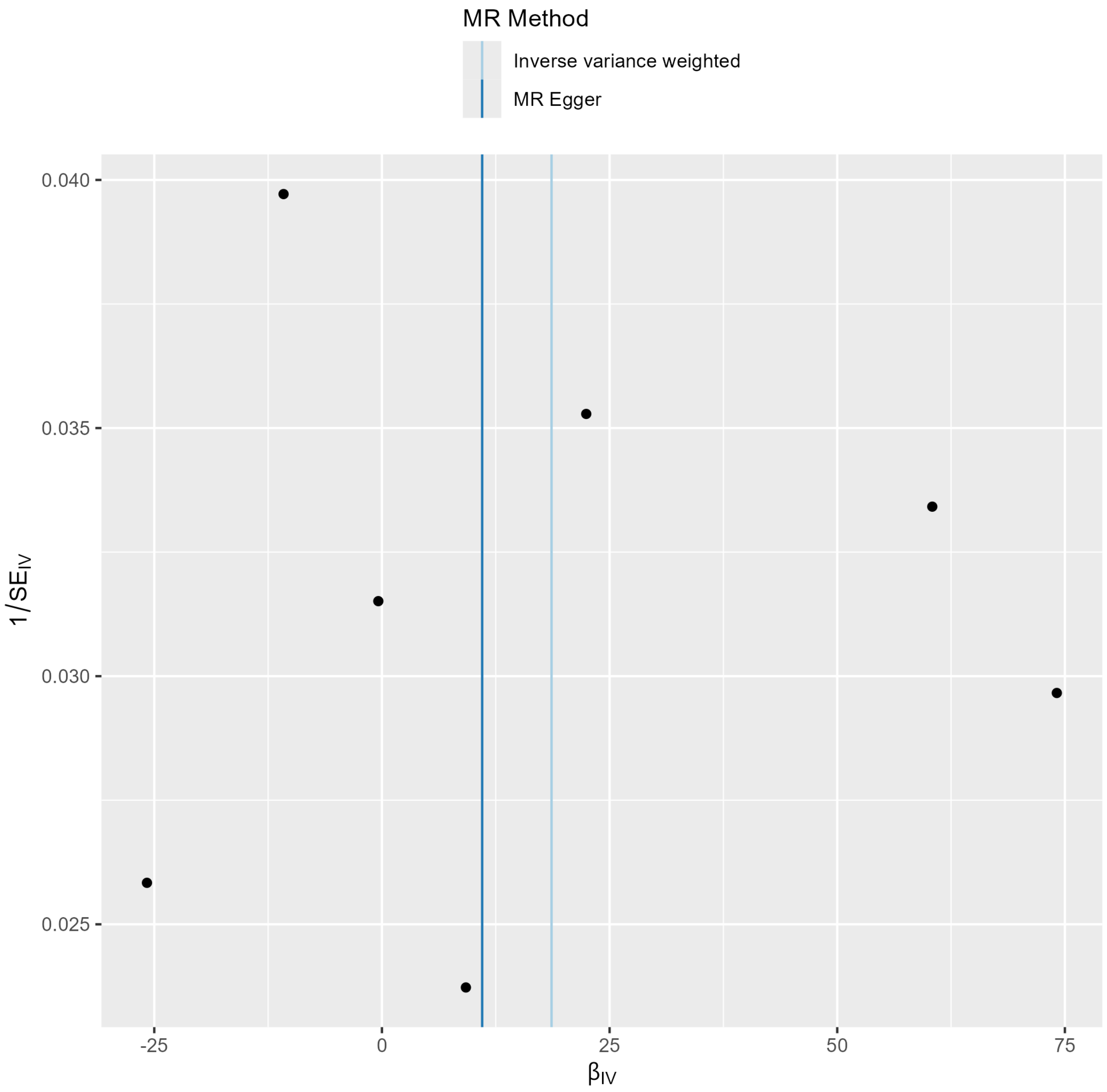

Ω-3-LAS leave-one-out

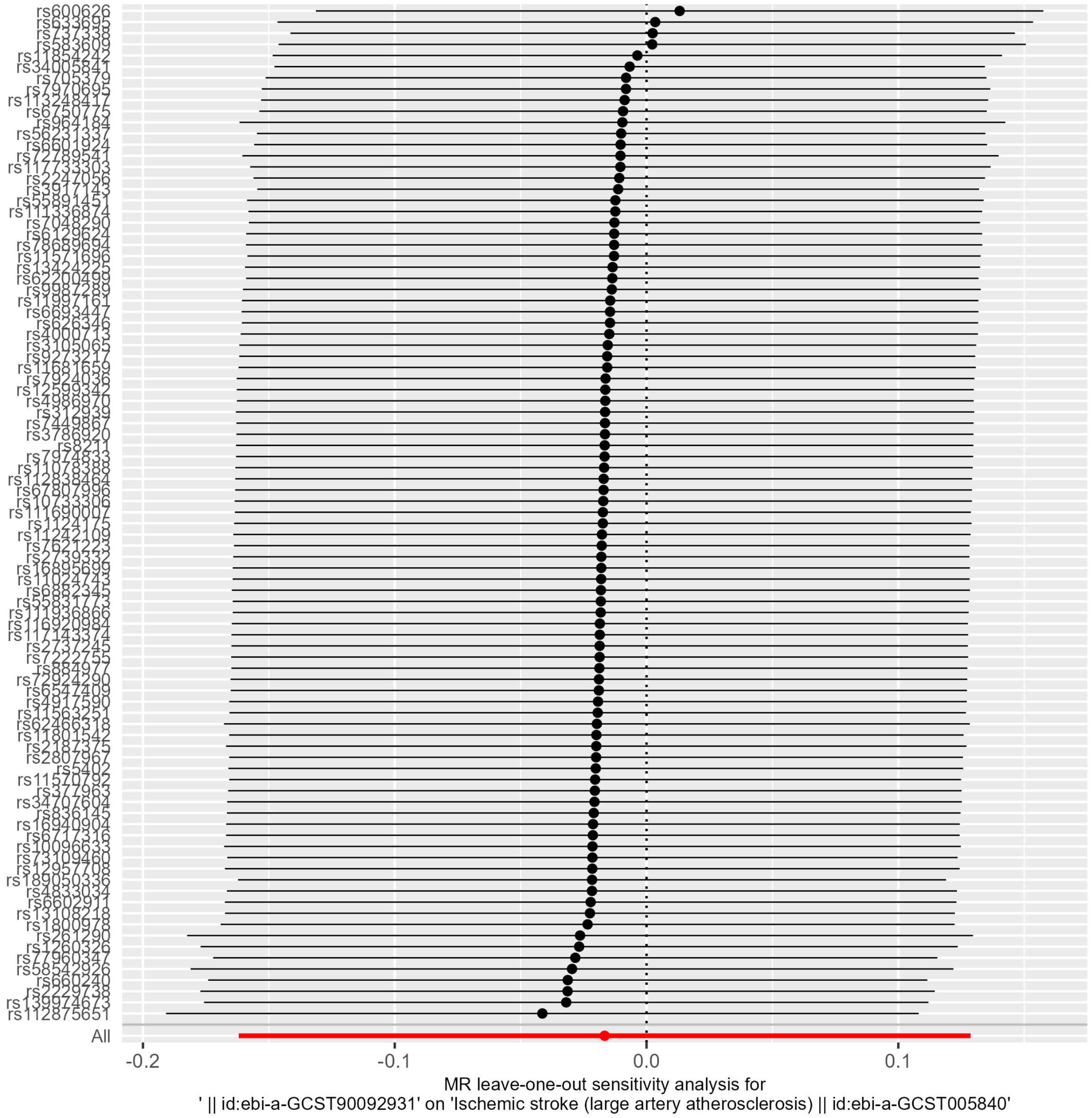

Ω-3-SVS leave-one-out

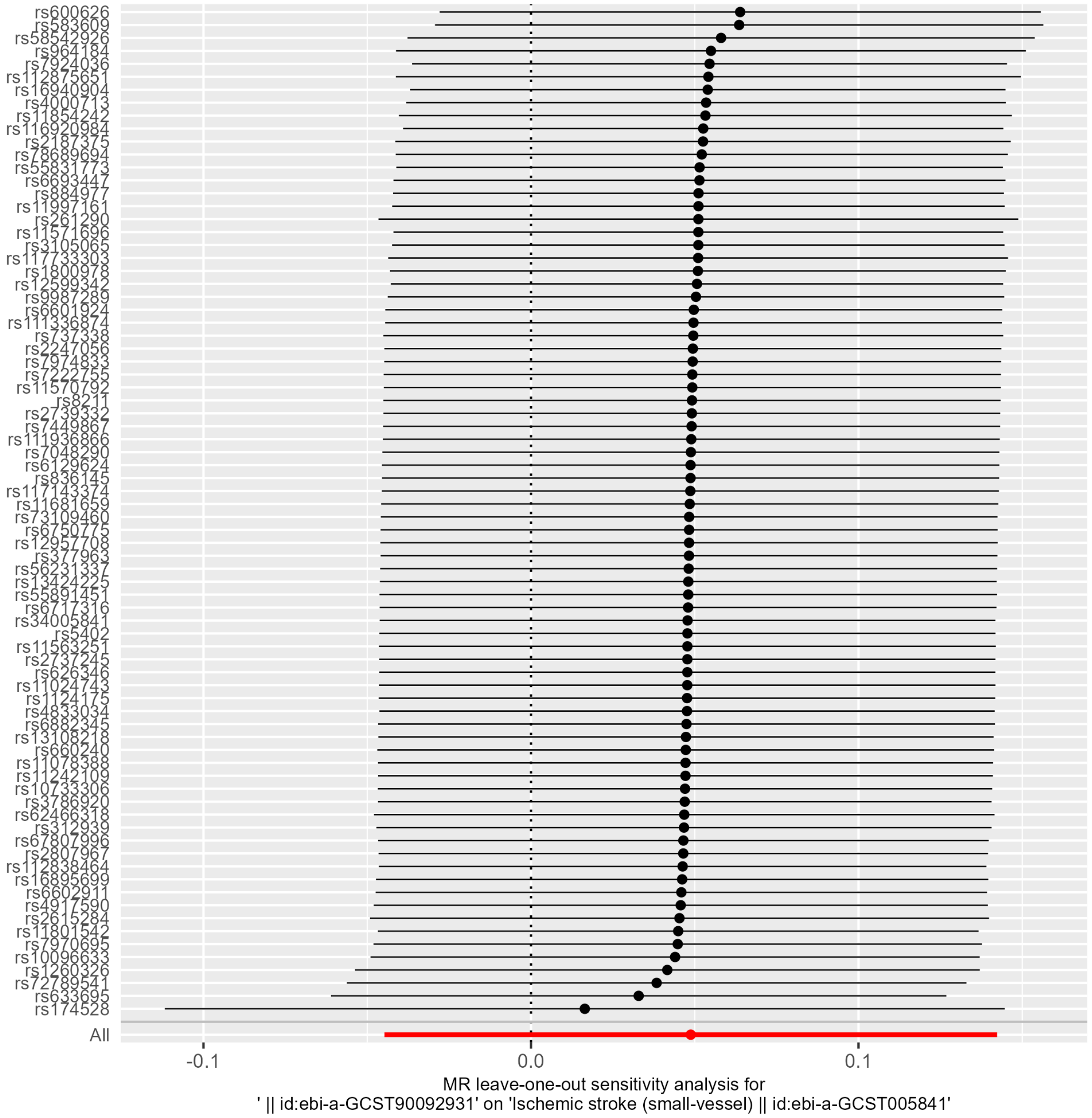

Ω-3-CES leave-one-out

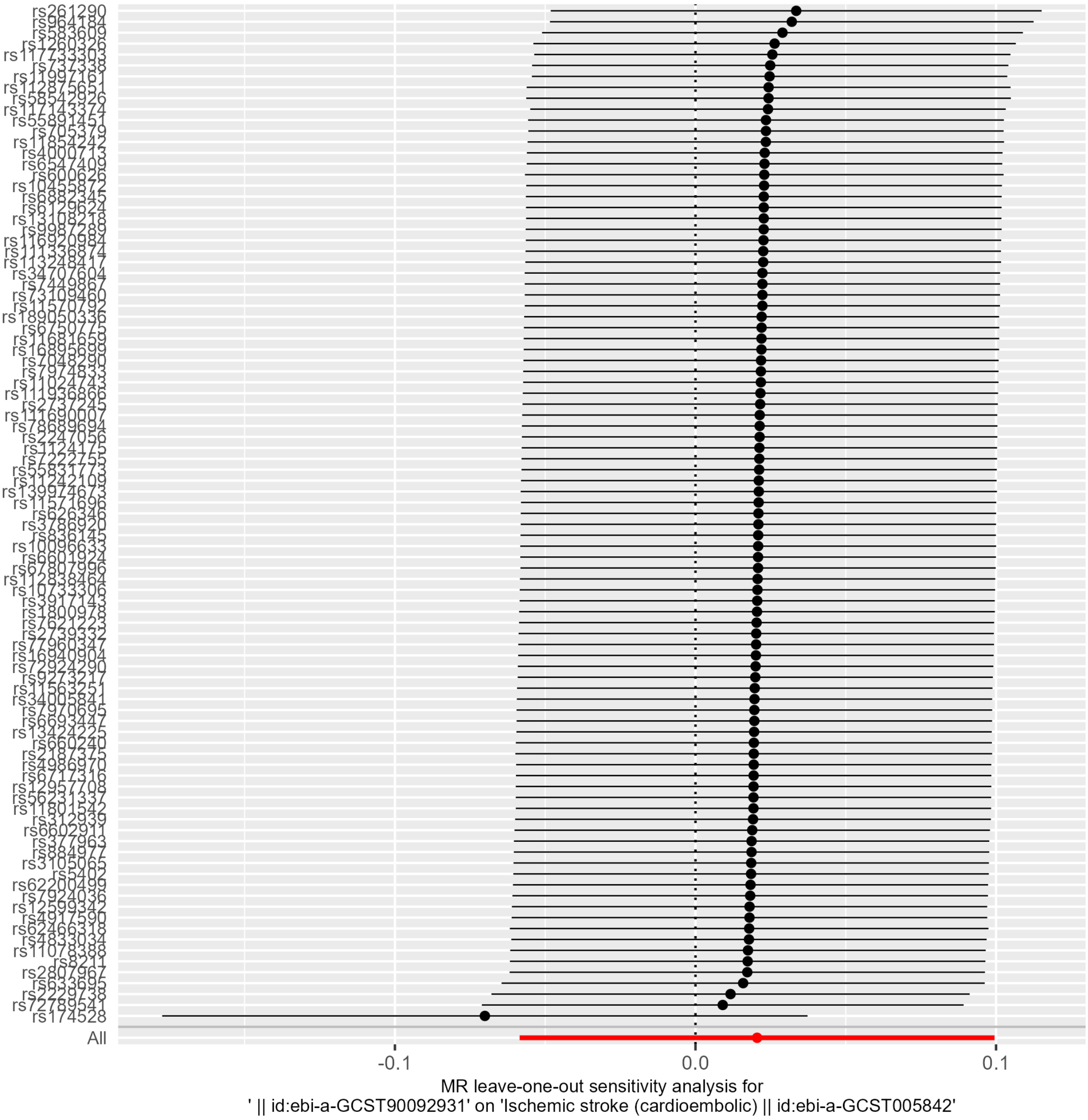

Ω-3-IS leave-one-out

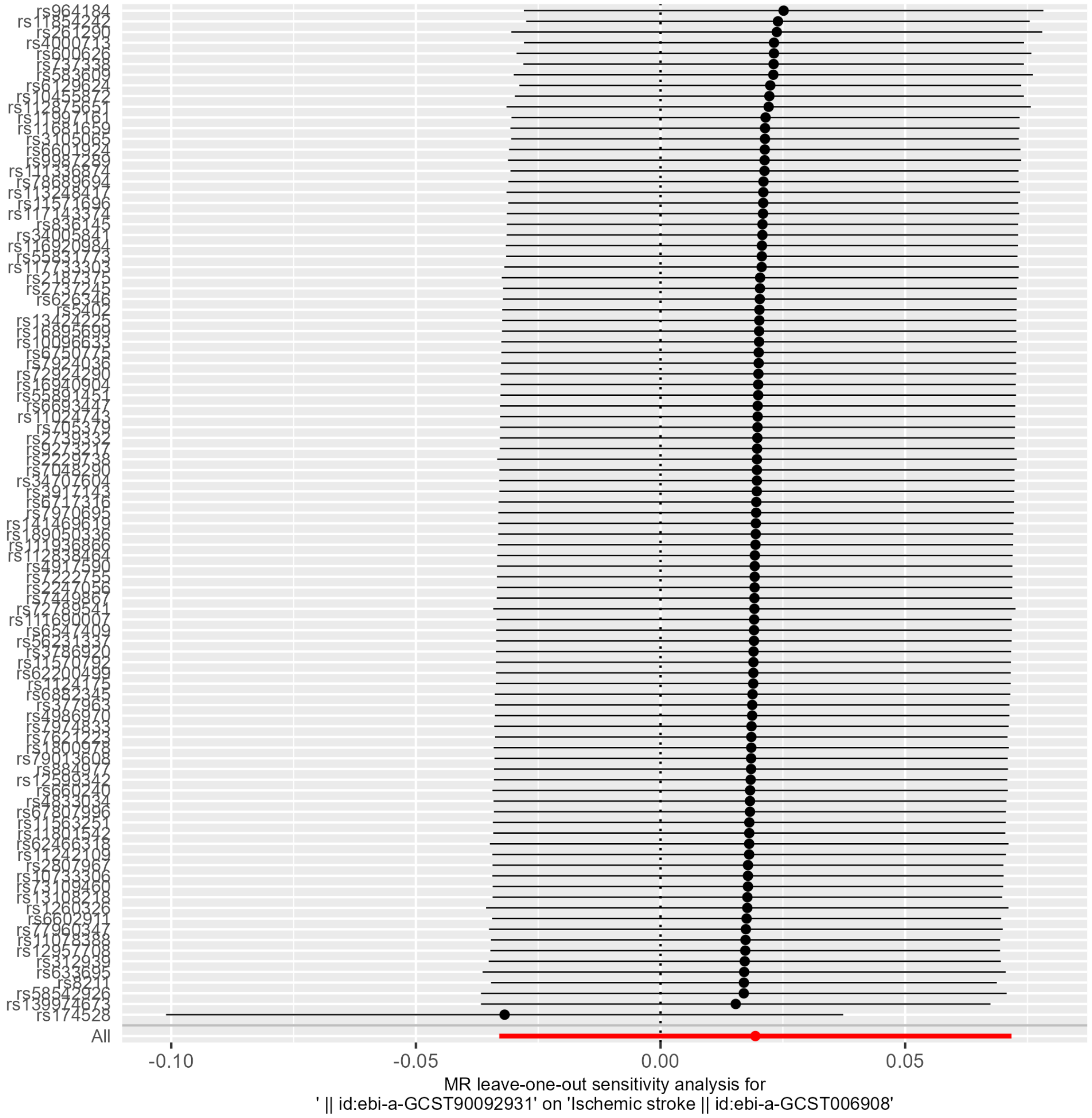

Ω-3-LS leave-one-out

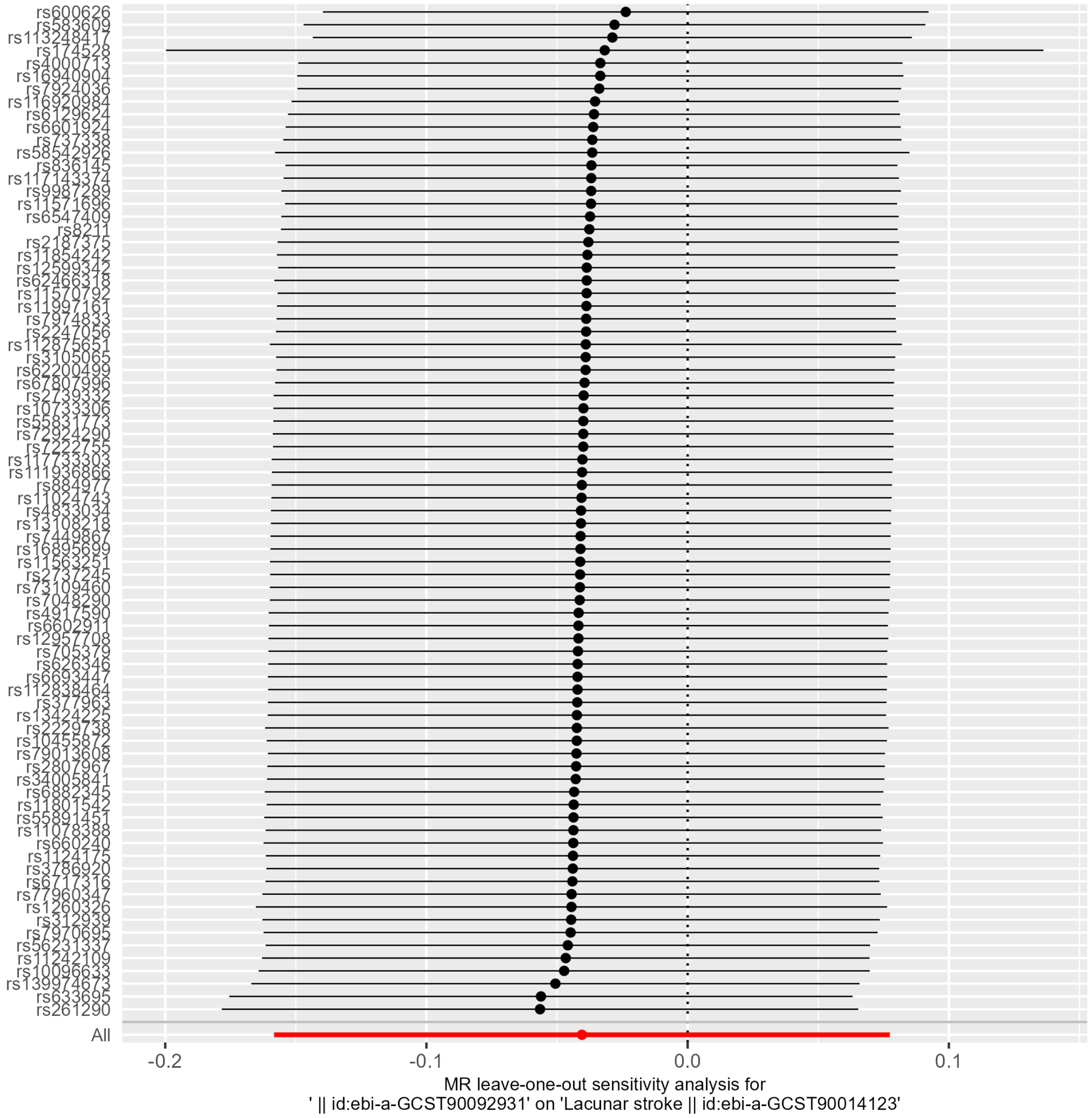

Ω-3-LAS scatter plot

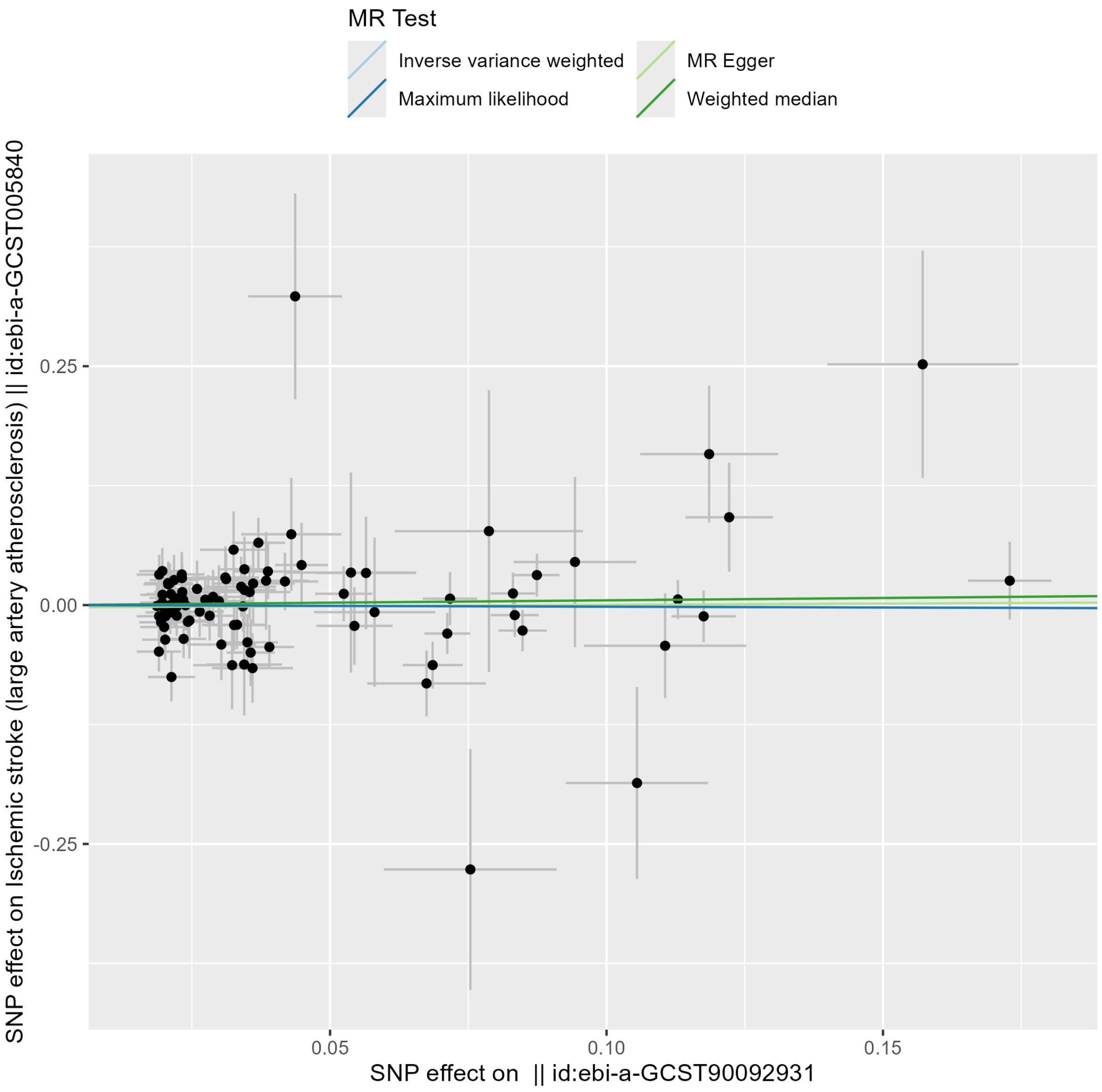

Ω-3-SVS scatter plot

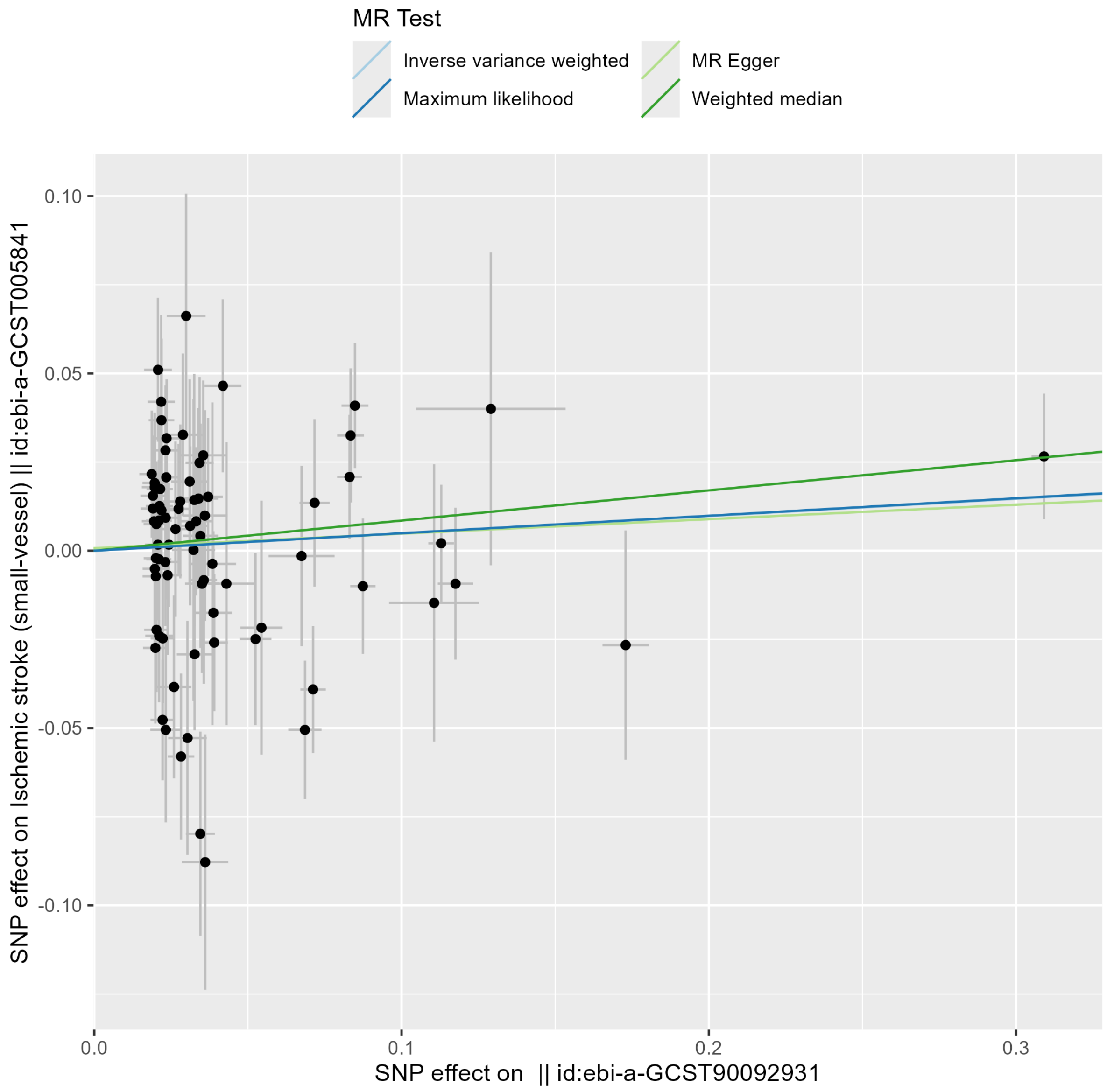

Ω-3-CES scatter plot

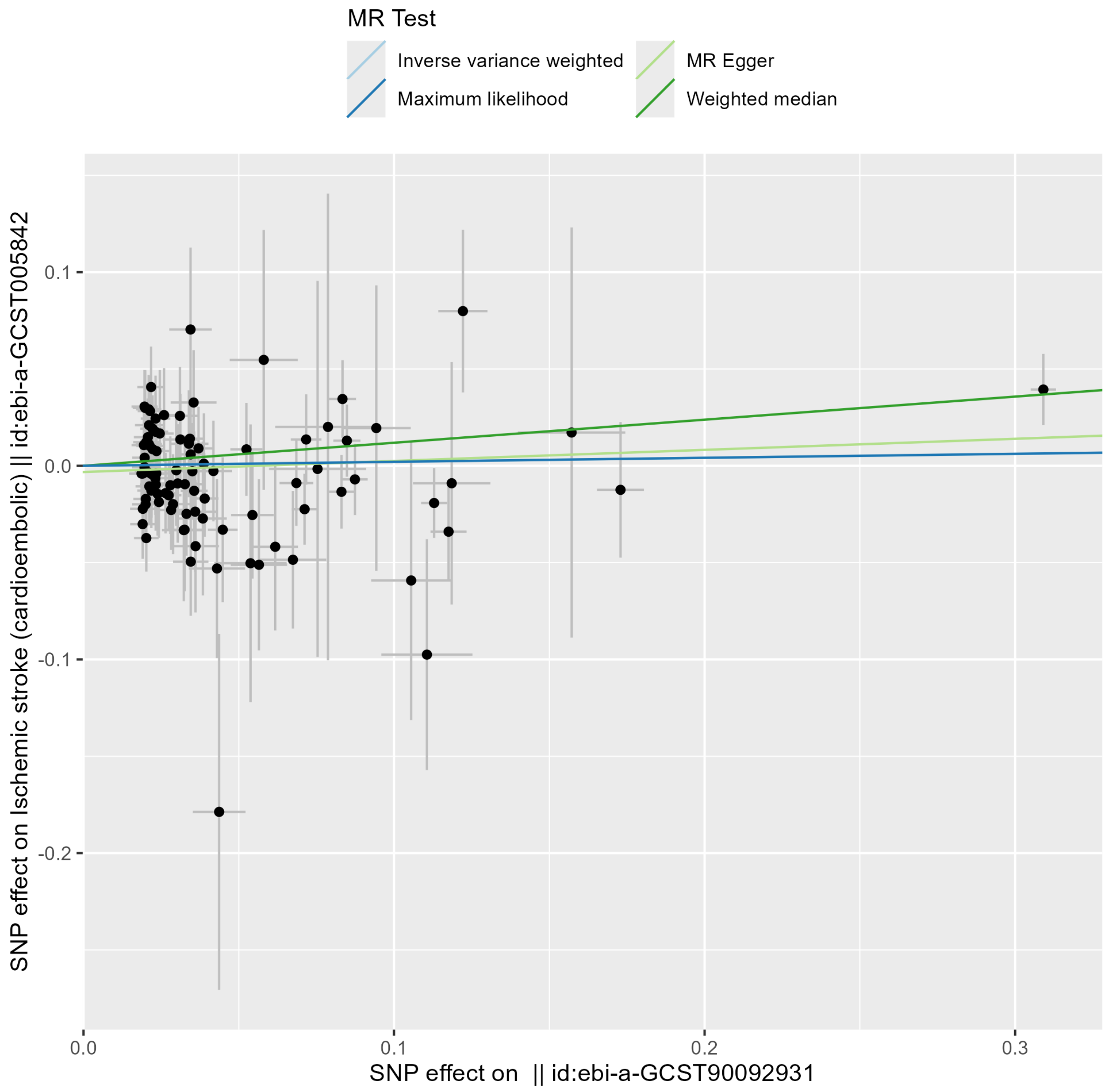

Ω-3-IS scatter plot

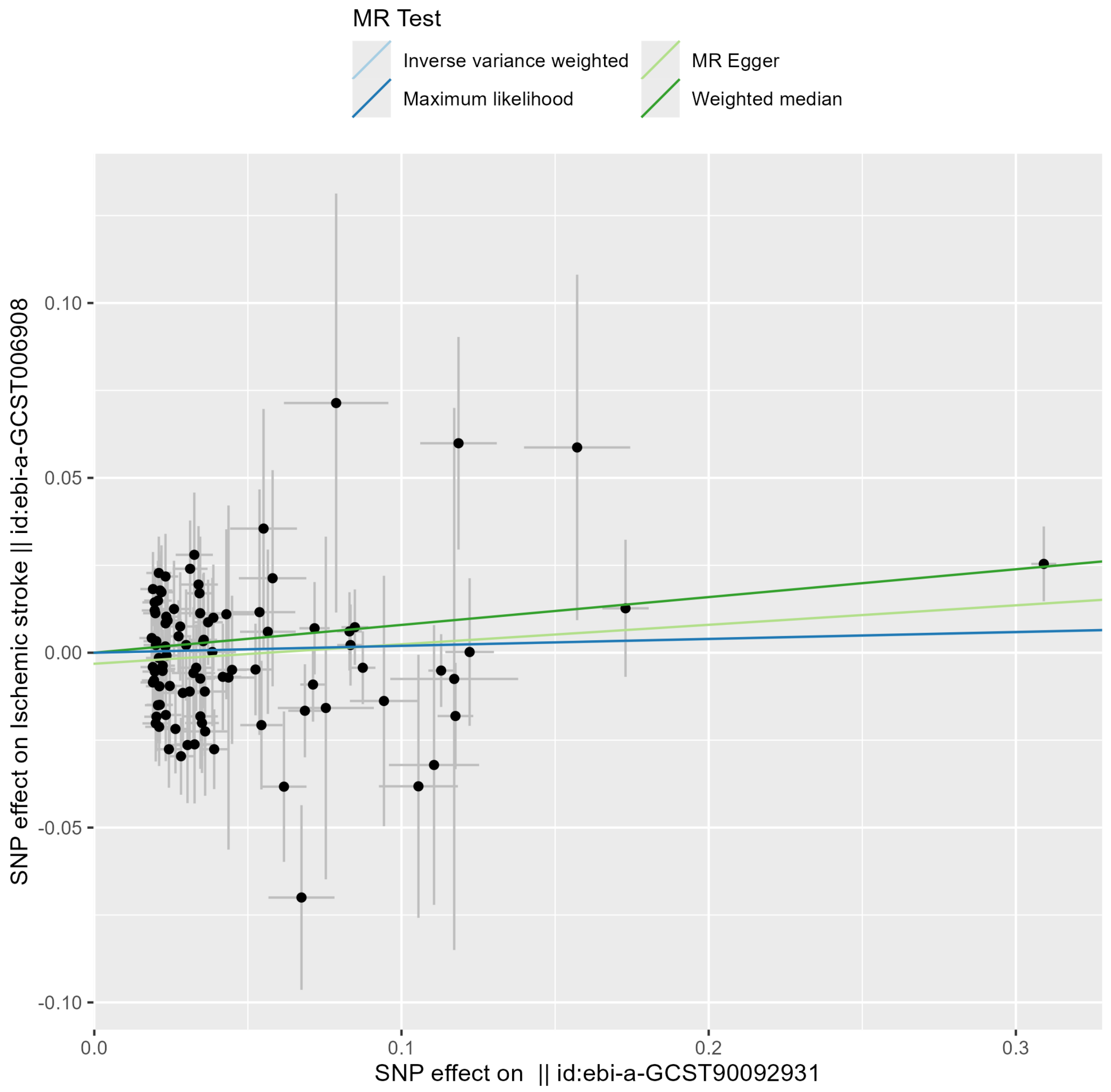

Ω-3-LS scatter plot

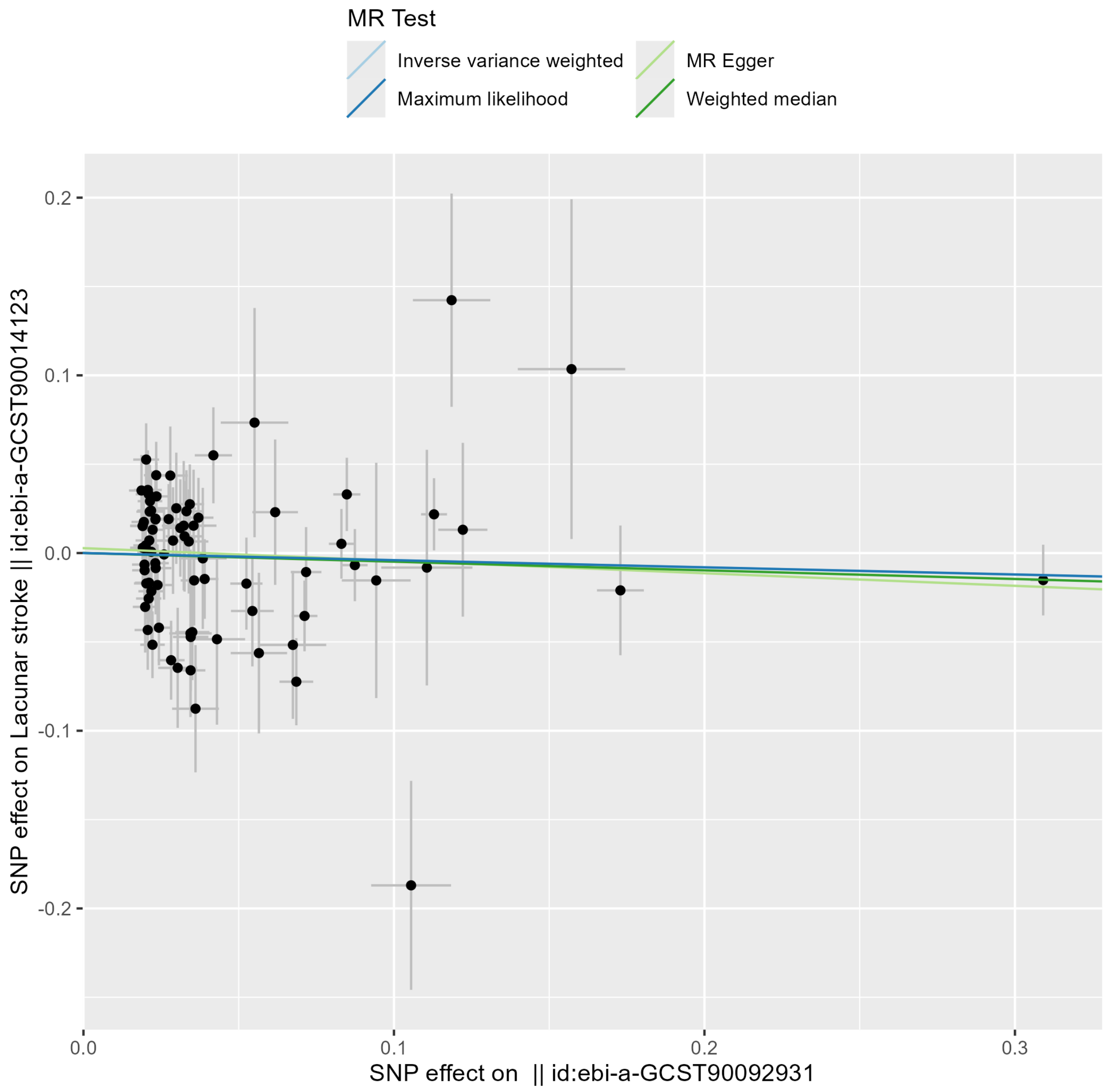

Ω-3-LAS forest plot

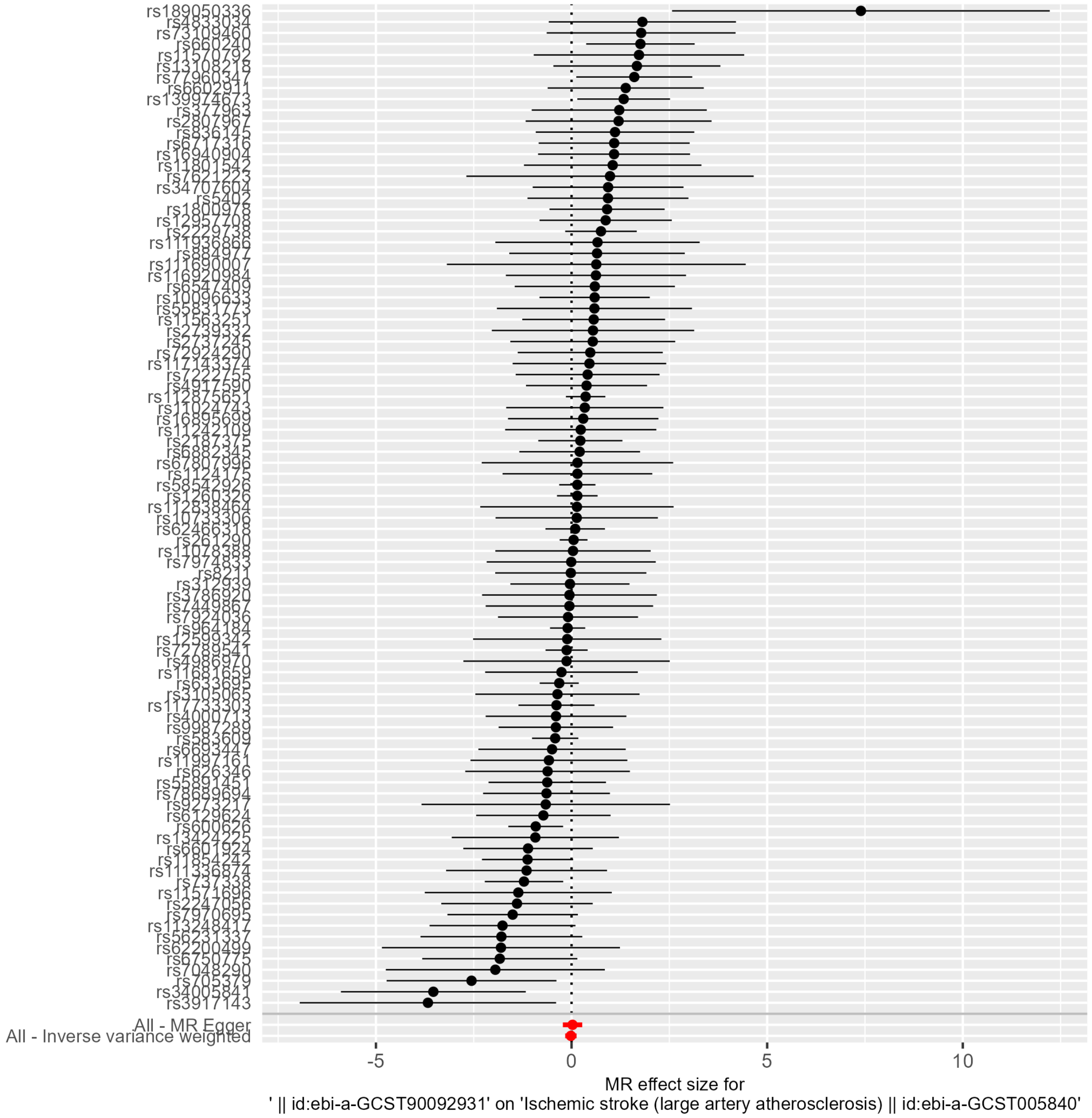

Ω-3-SVS forest plot

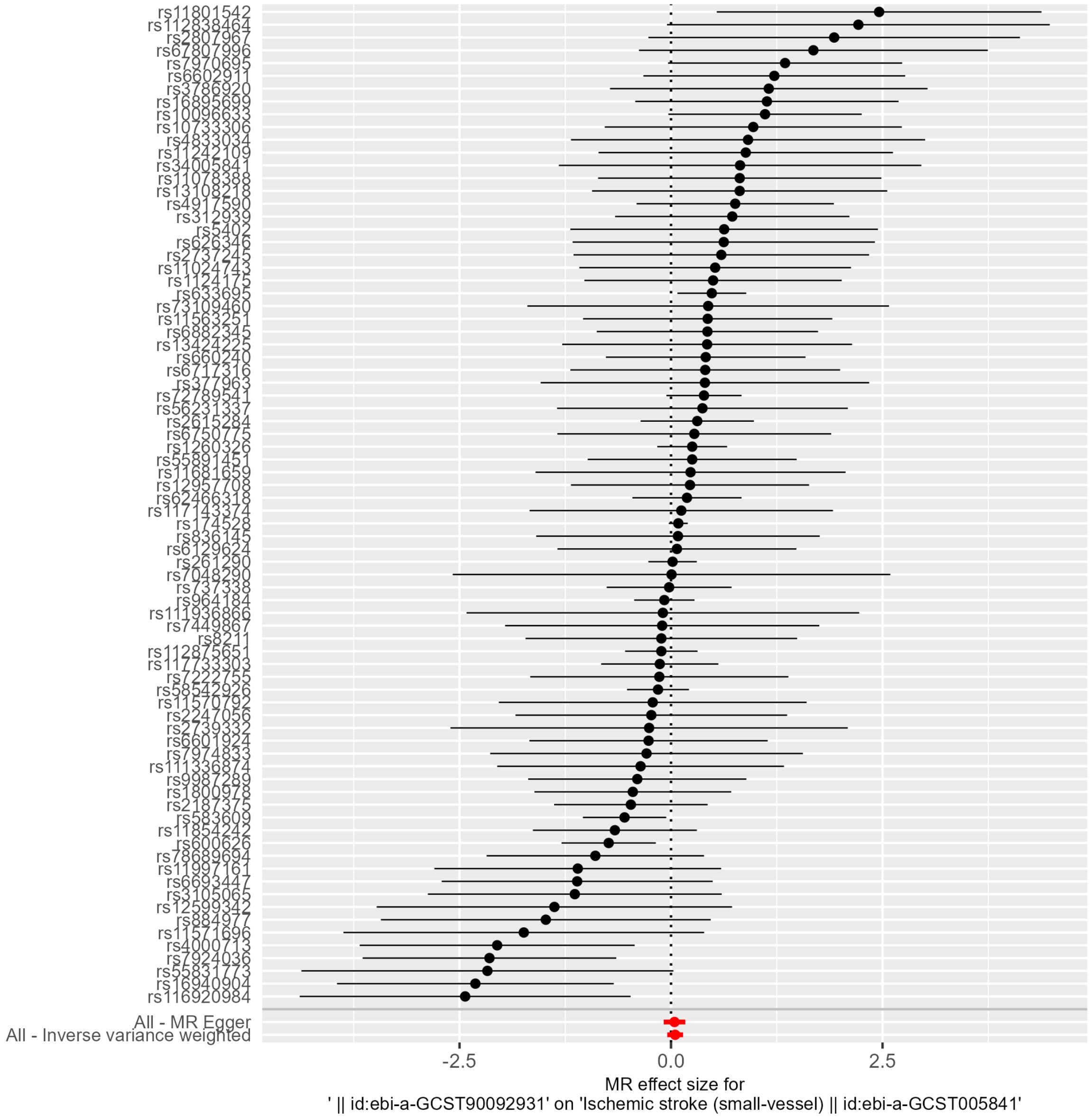

Ω-3-CES forest plot

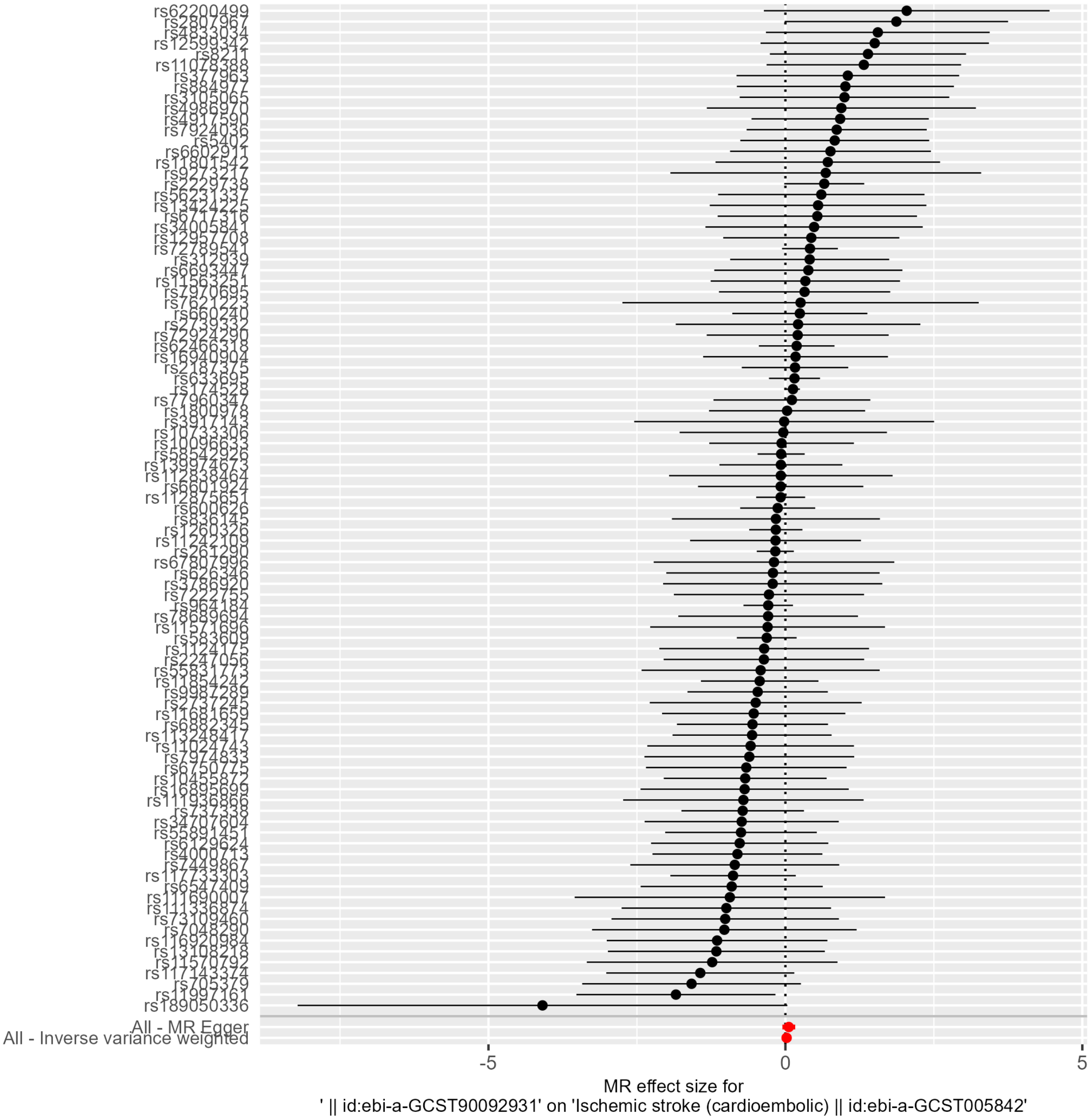

Ω-3-IS forest plot

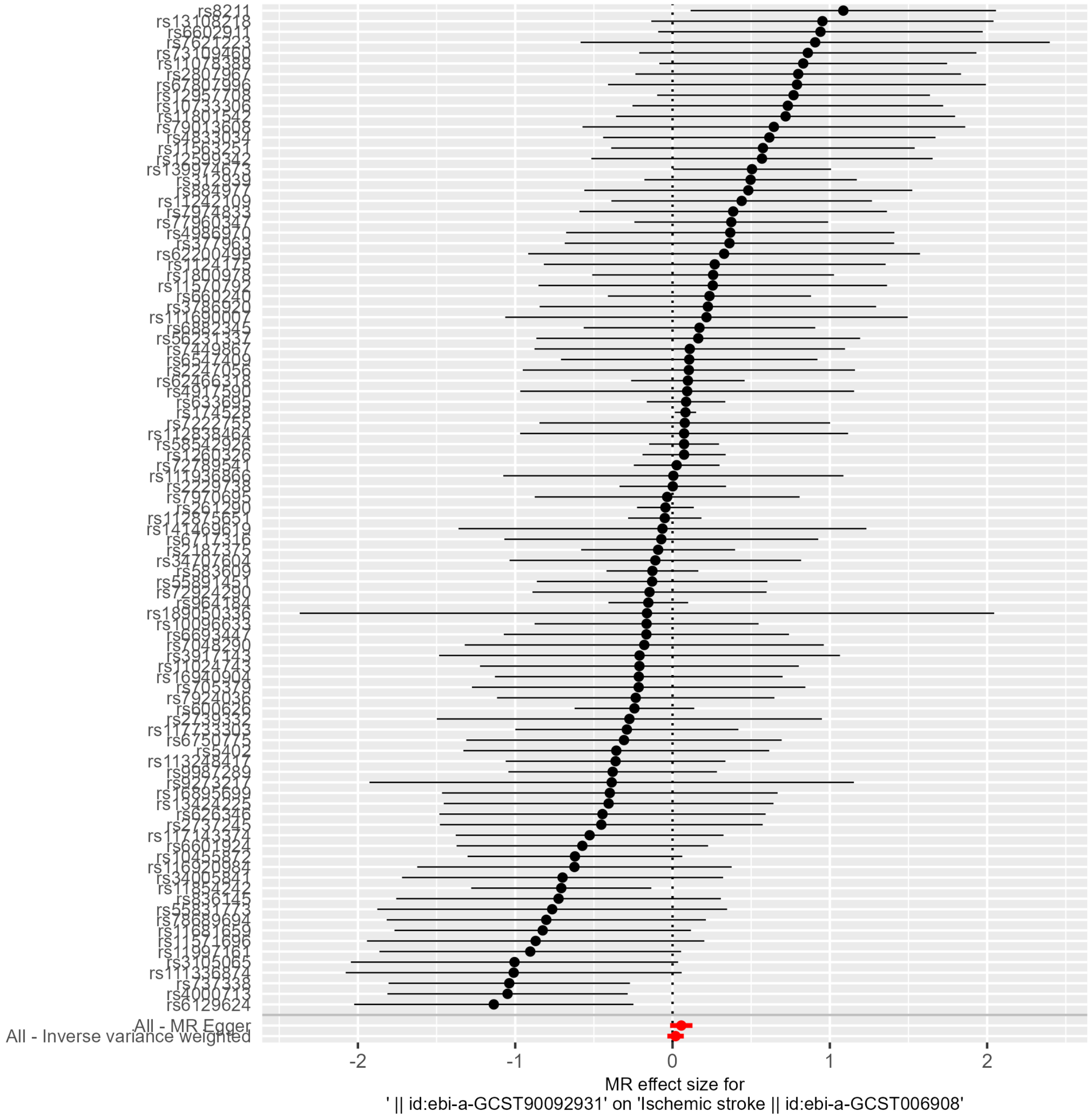

Ω-3-LS forest plot

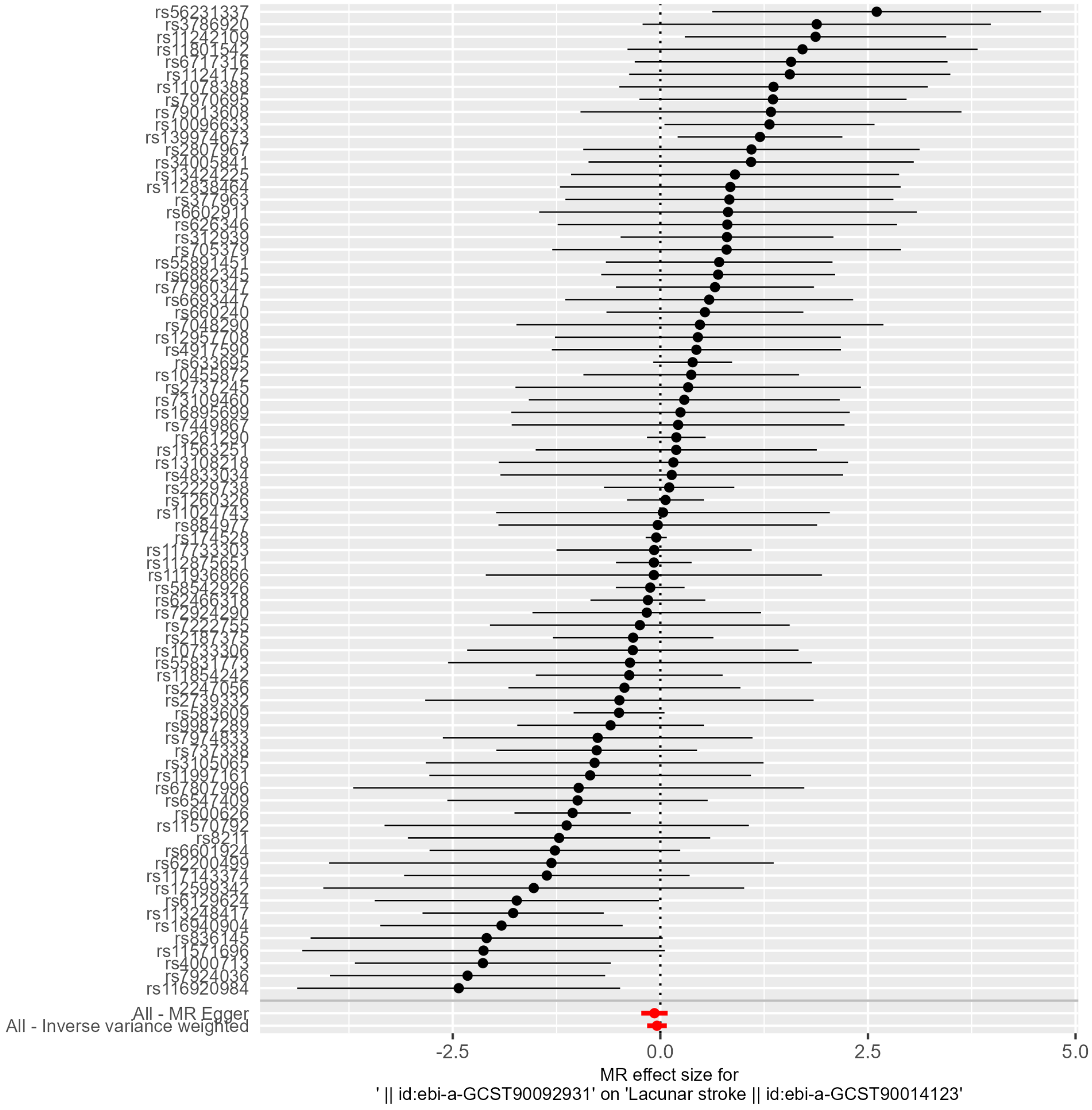

Ω-3-LAS funnel plot

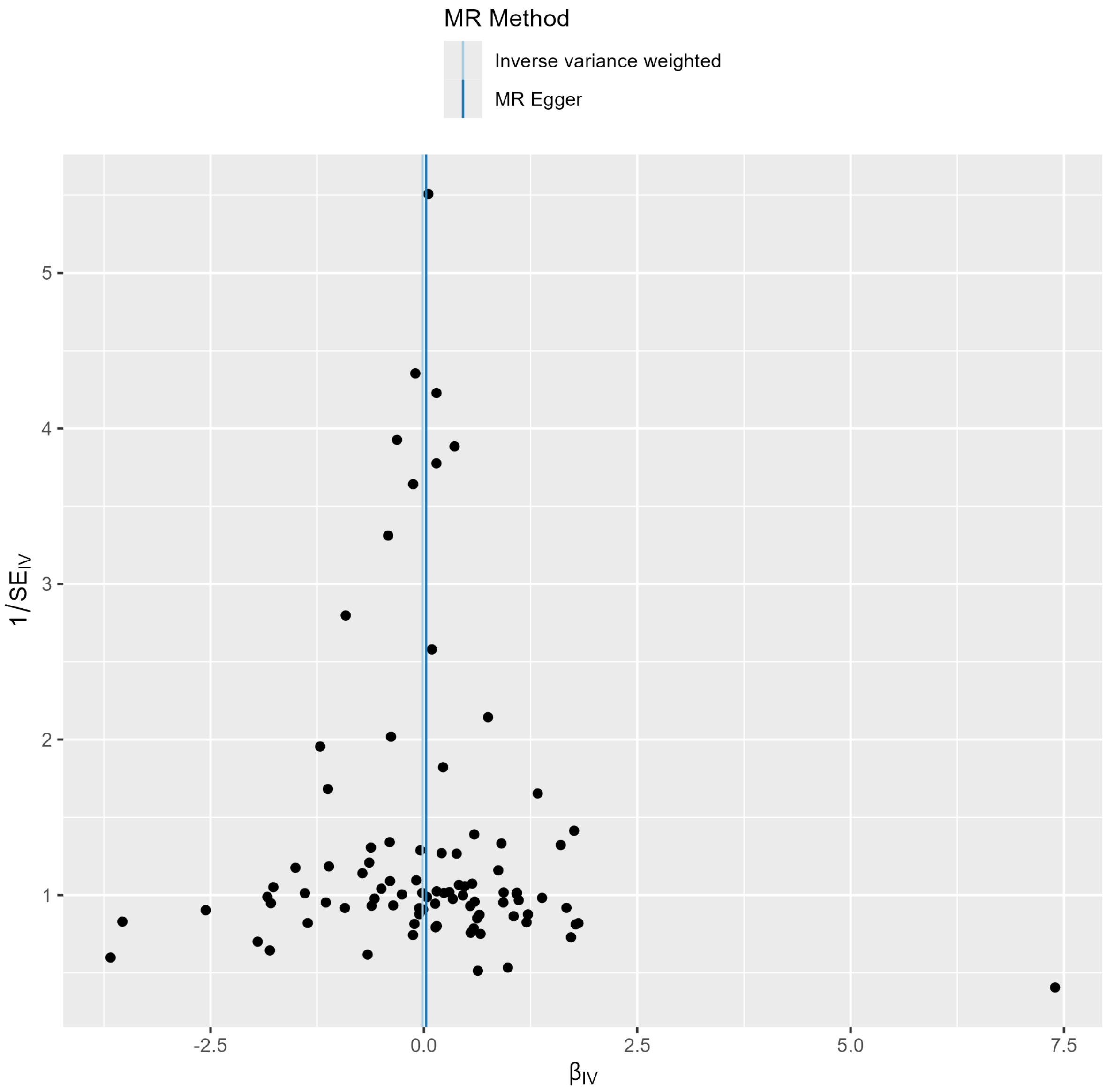

Ω-3-SVS funnel plot

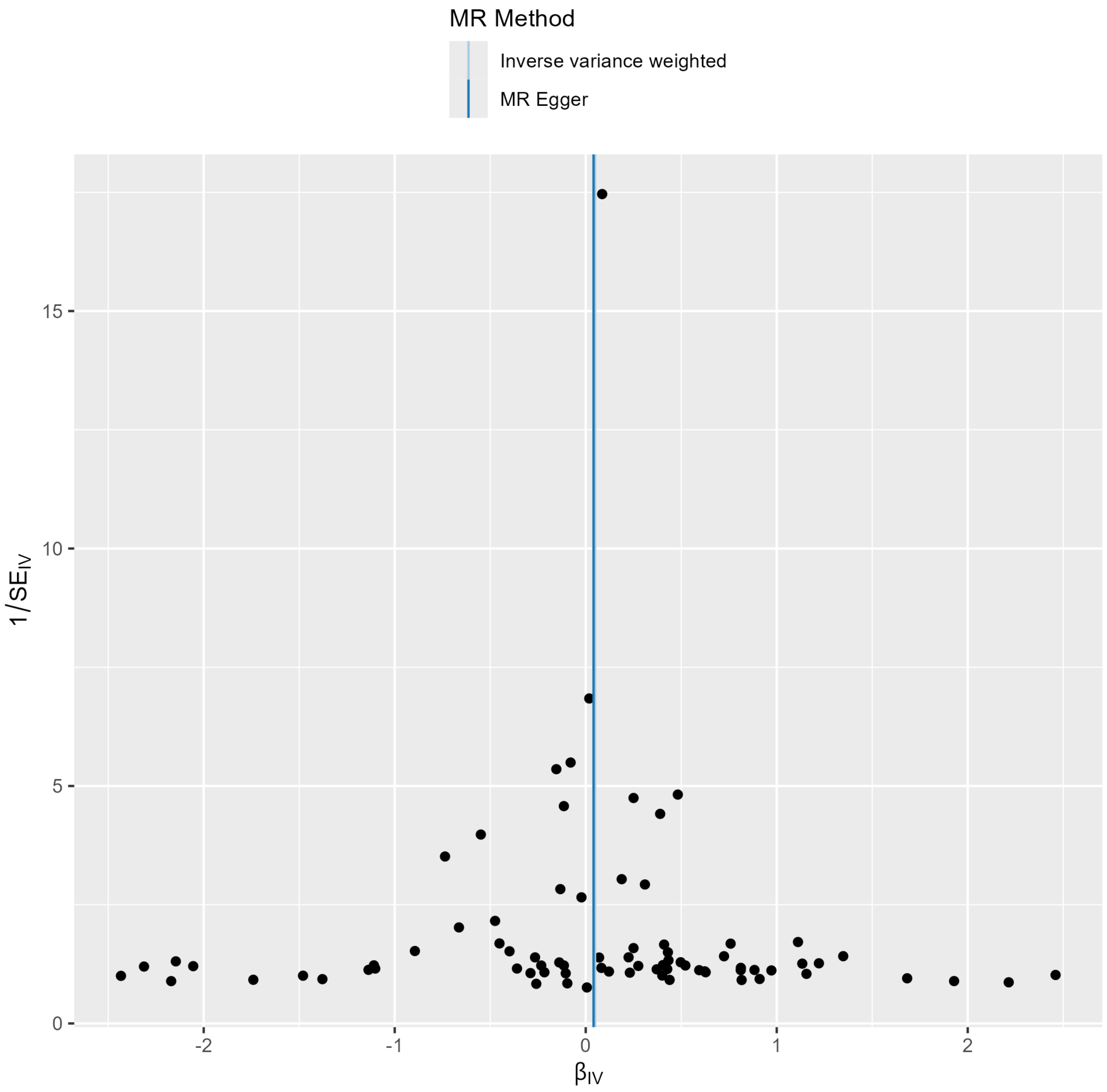

Ω-3-CES funnel plot

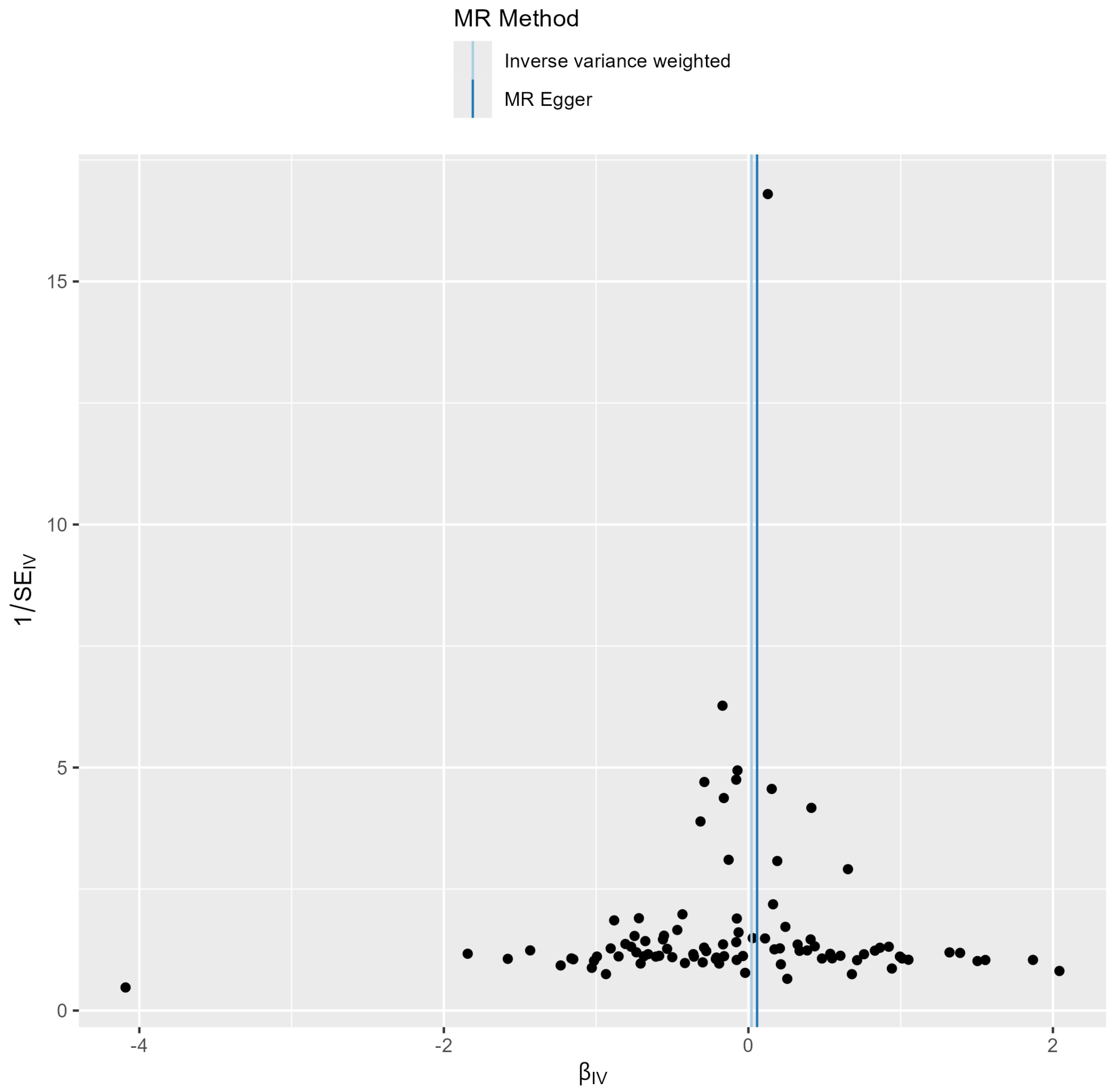

Ω-3-IS funnel plot

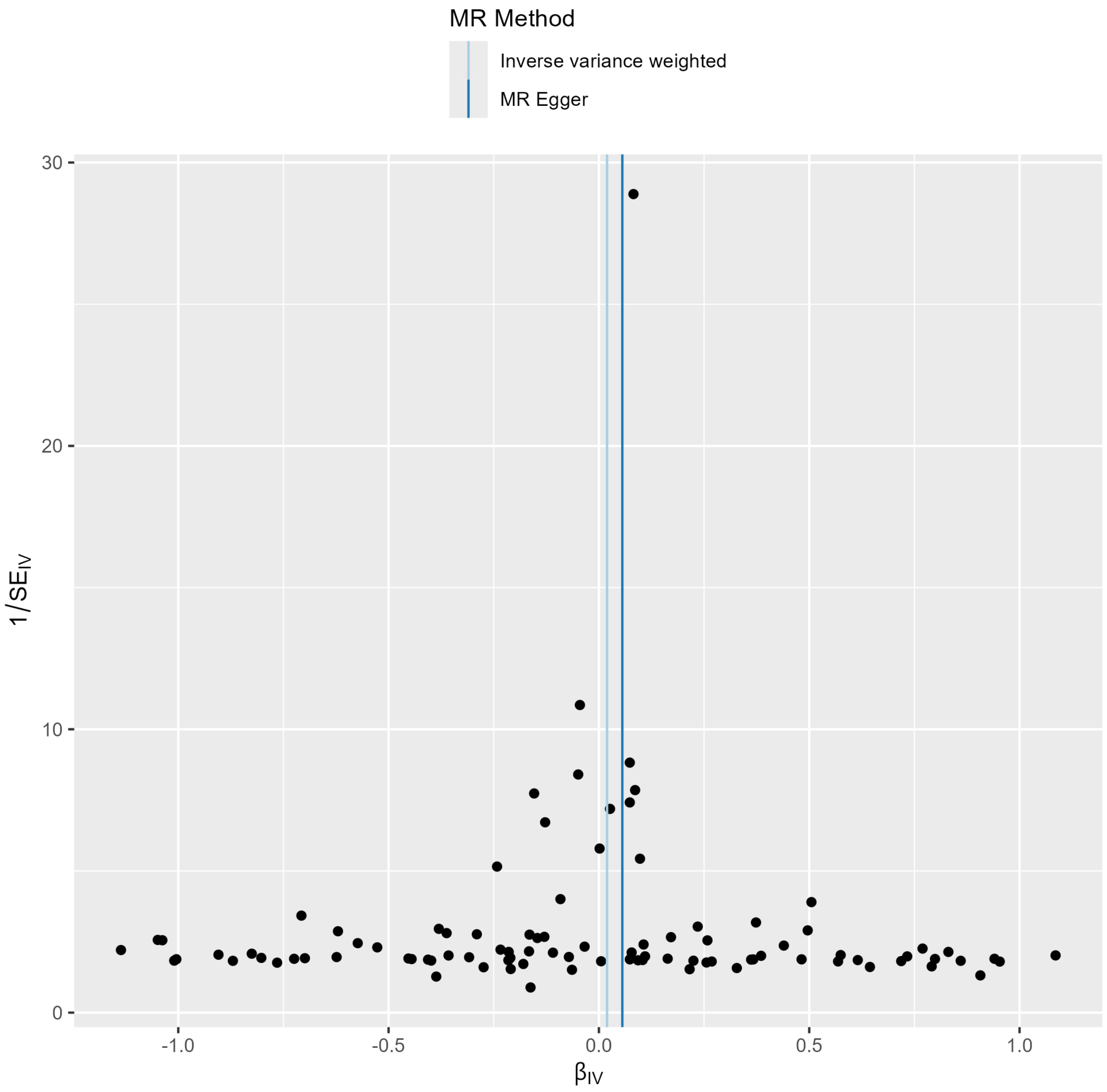

Ω-3-LS funnel plot

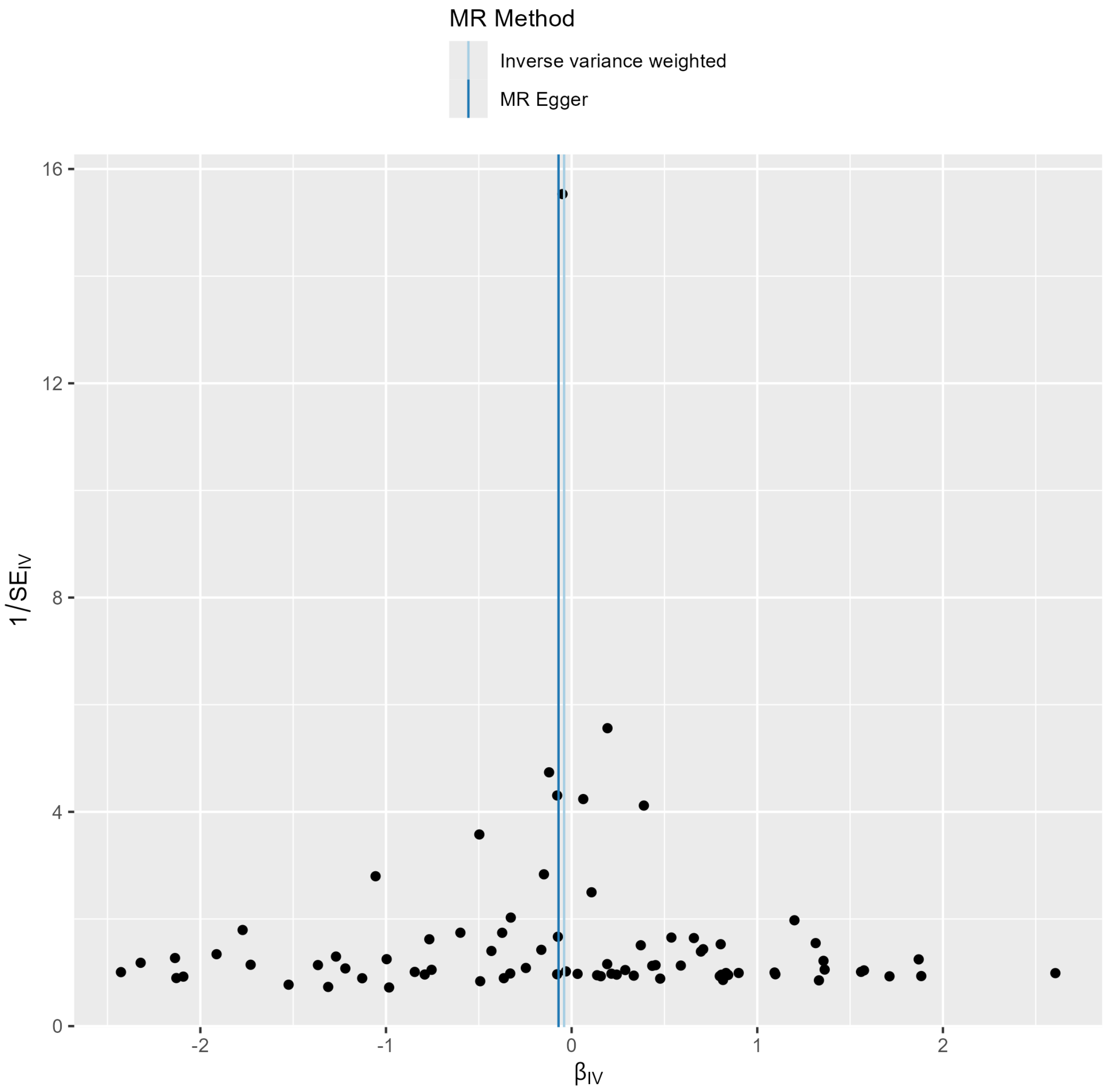

DHA-LAS leave-one-out

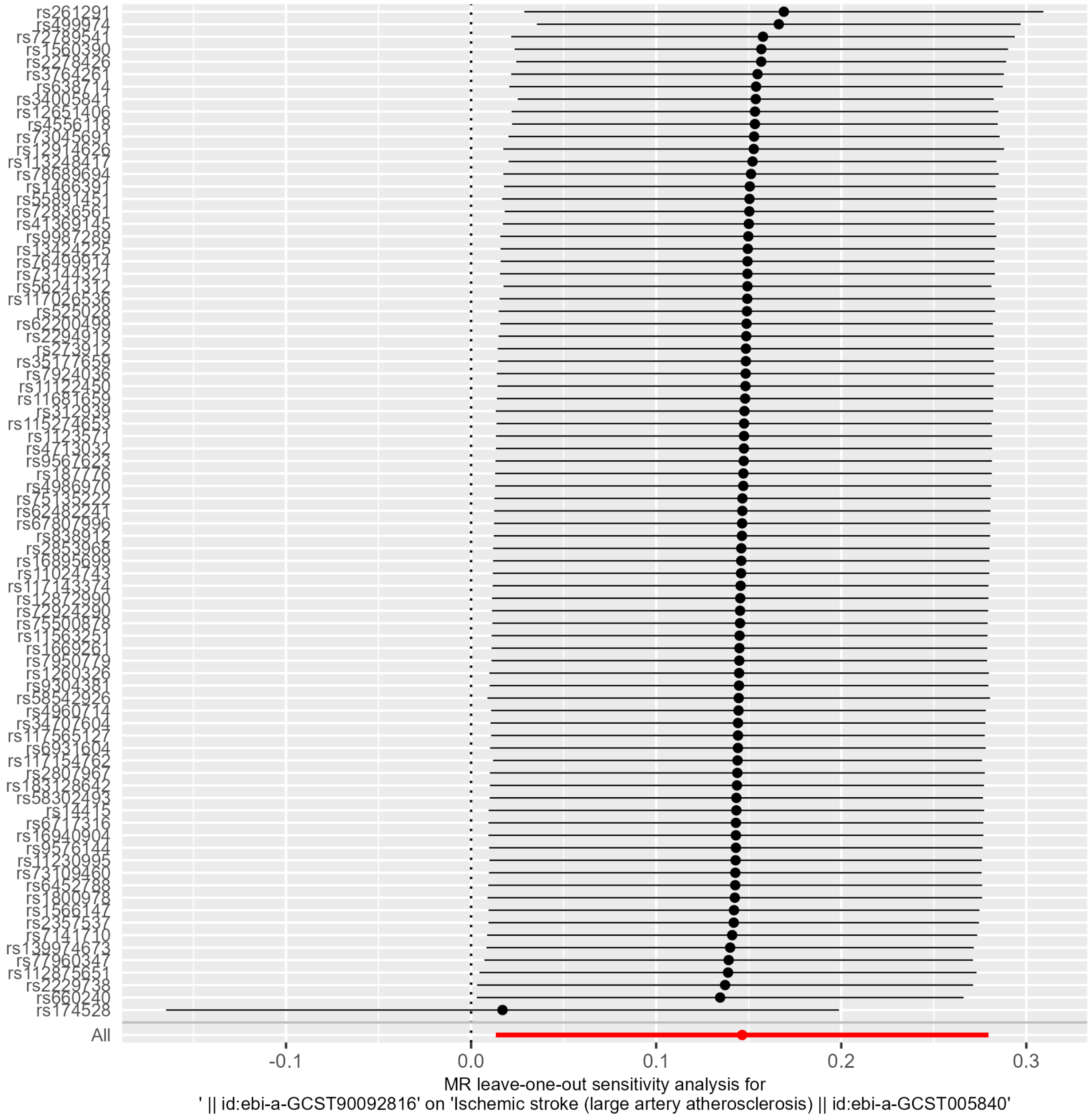

DHA-SVS leave-one-out

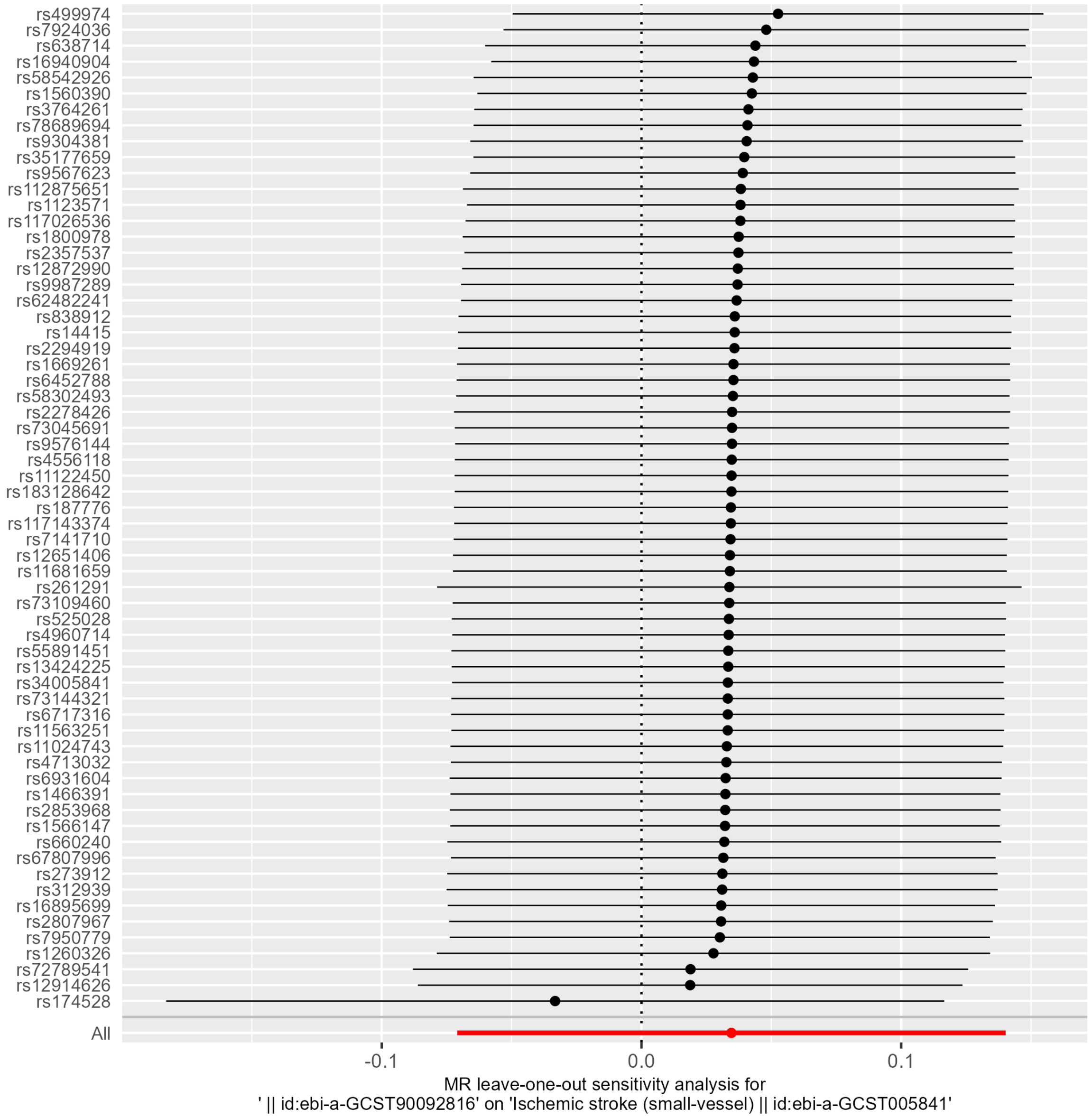

DHA-CES leave-one-out

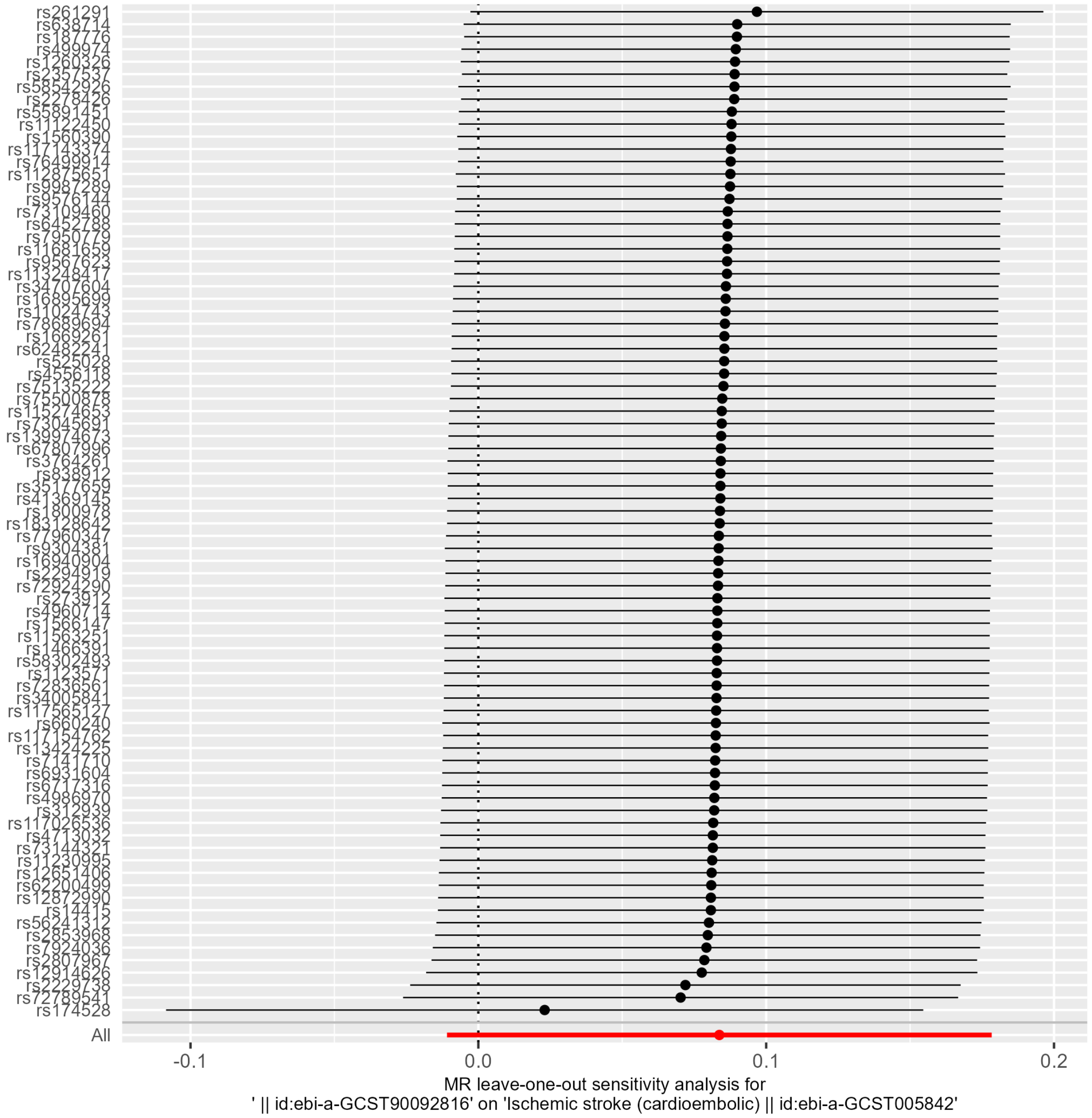

DHA-IS leave-one-out

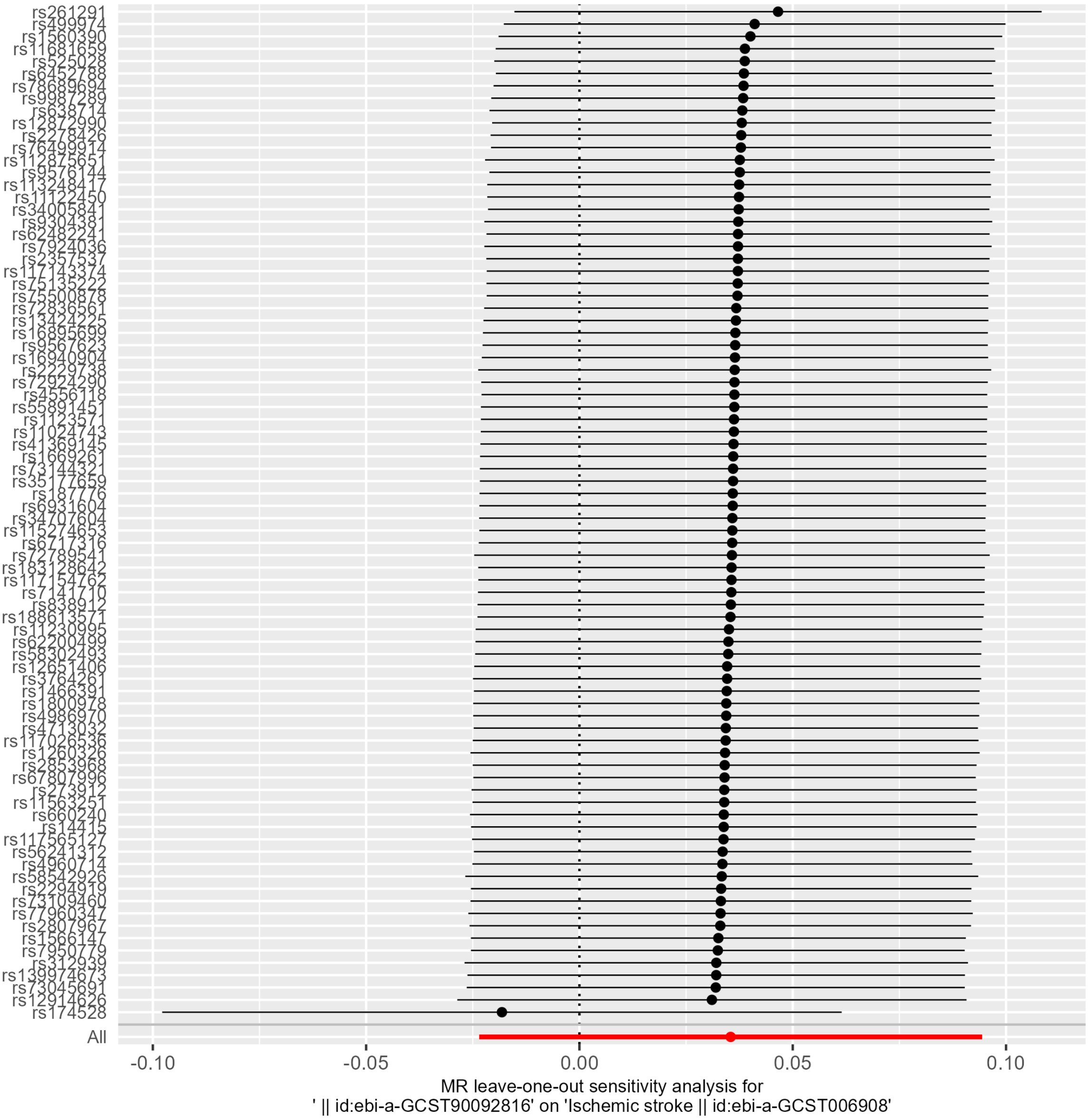

DHA-LS leave-one-out

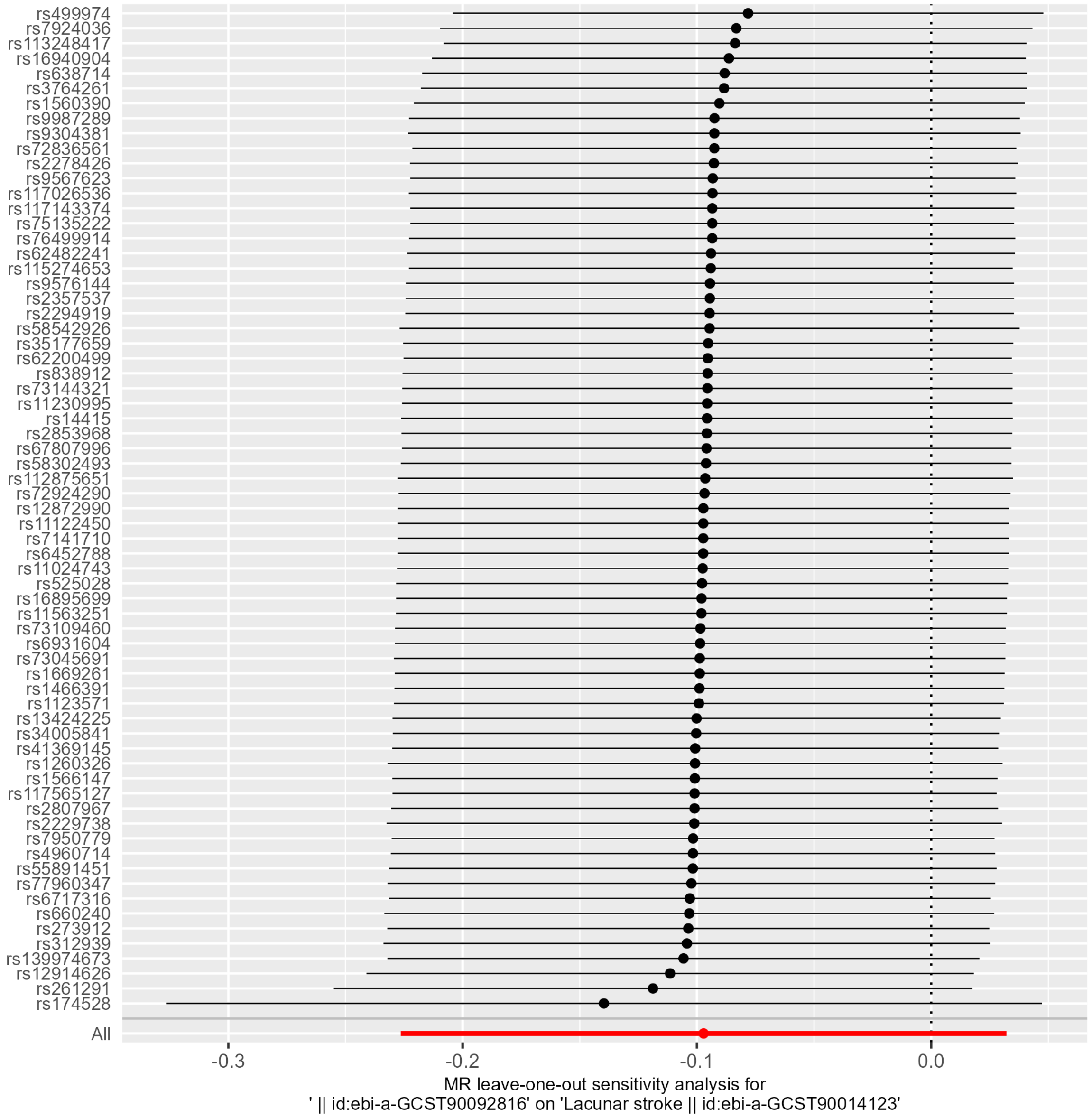

DHA-DBP leave-one-out

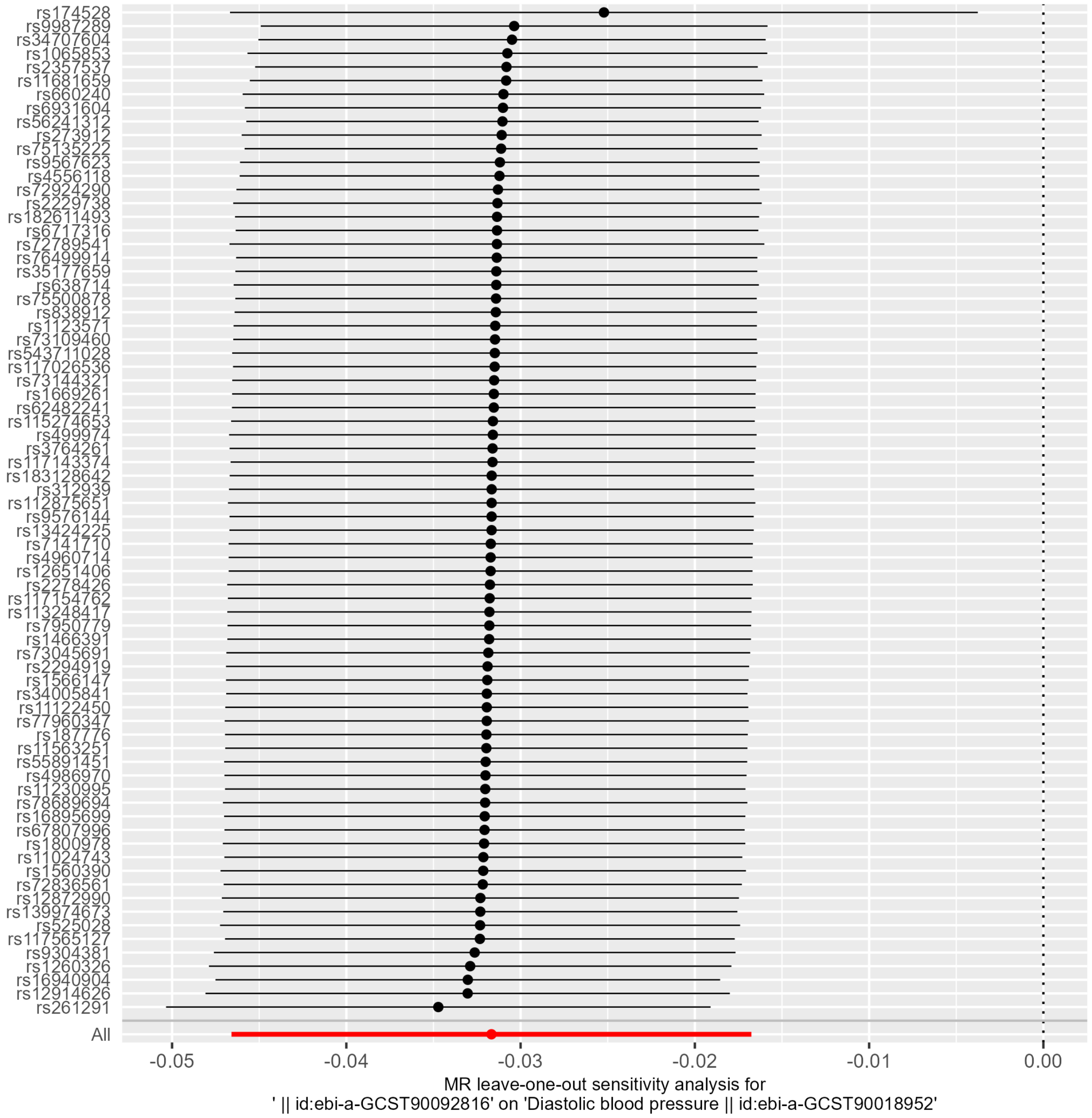

DHA-SBP leave-one-out

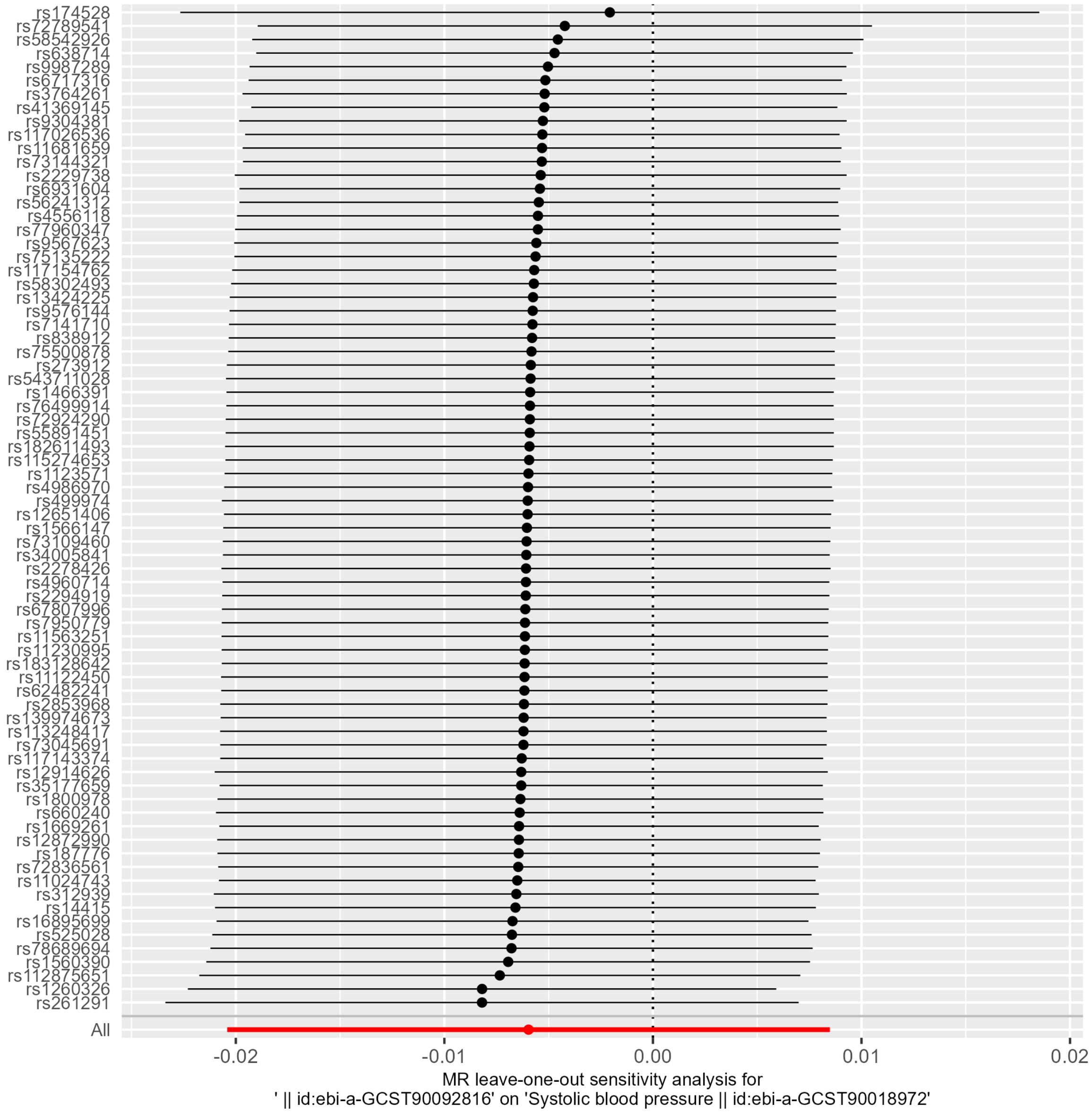

Ω-3 rate-LAS leave-one-out

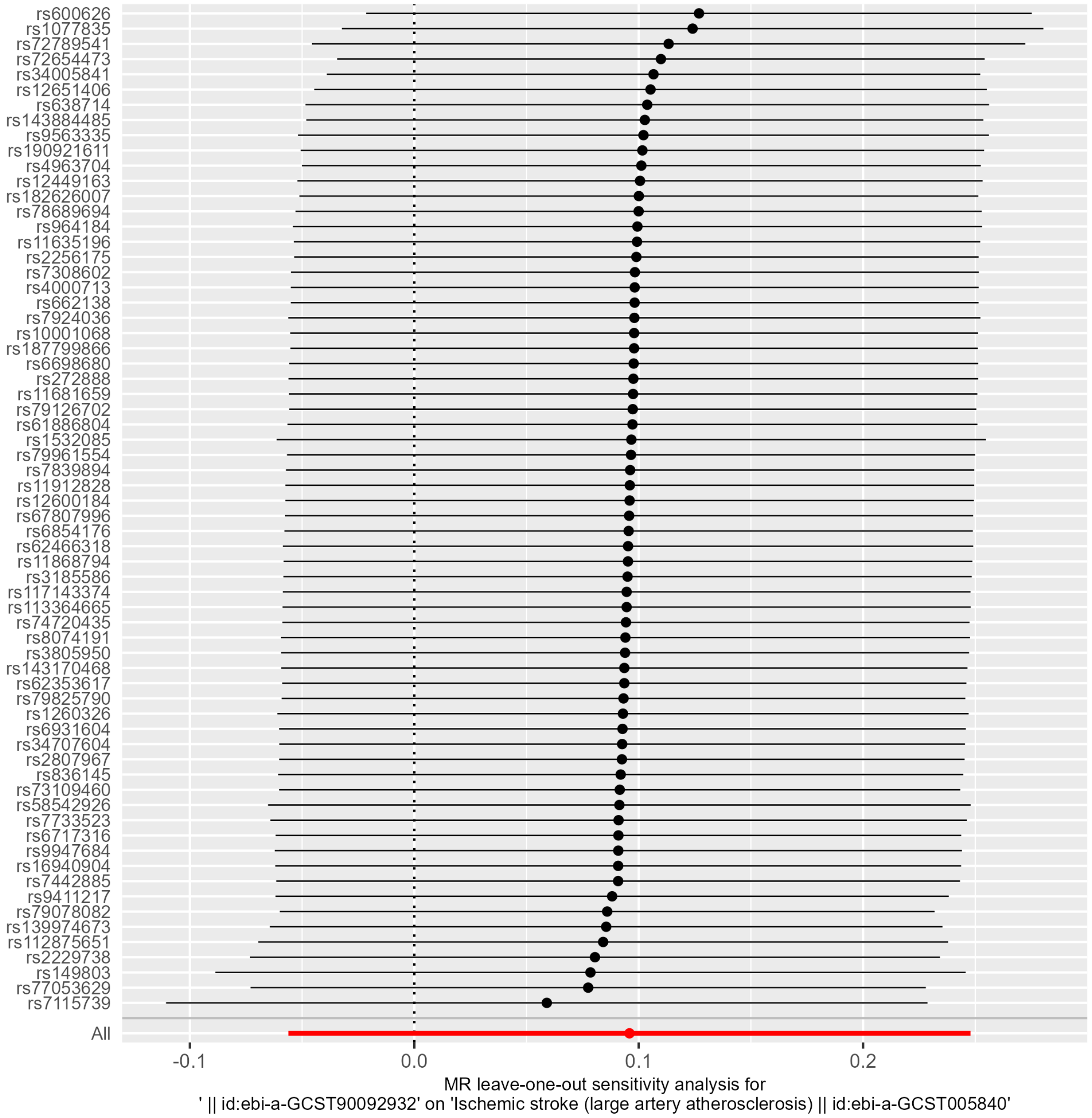

Ω-3 rate-SVS leave-one-out

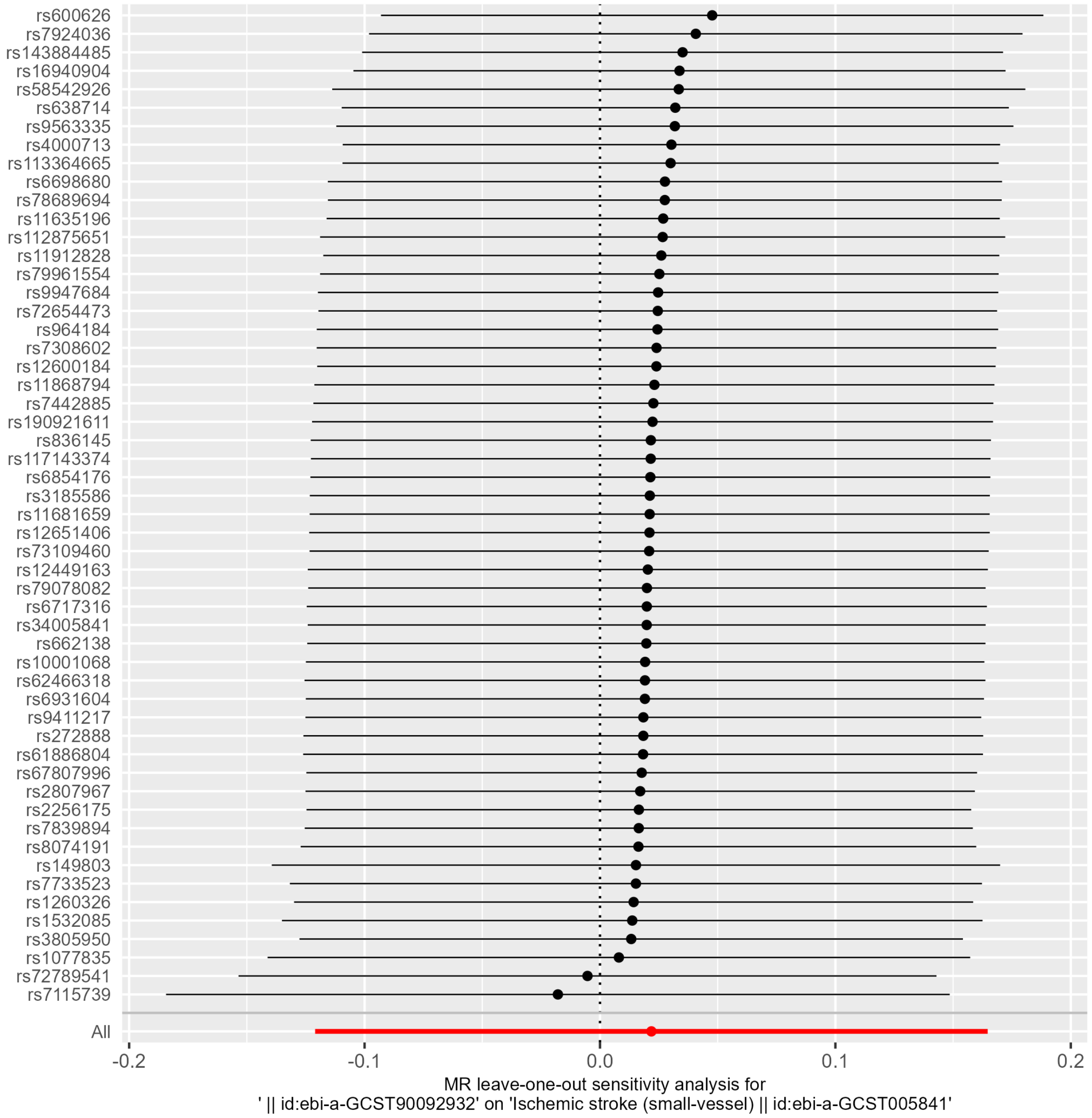

Ω-3 rate-CES leave-one-out

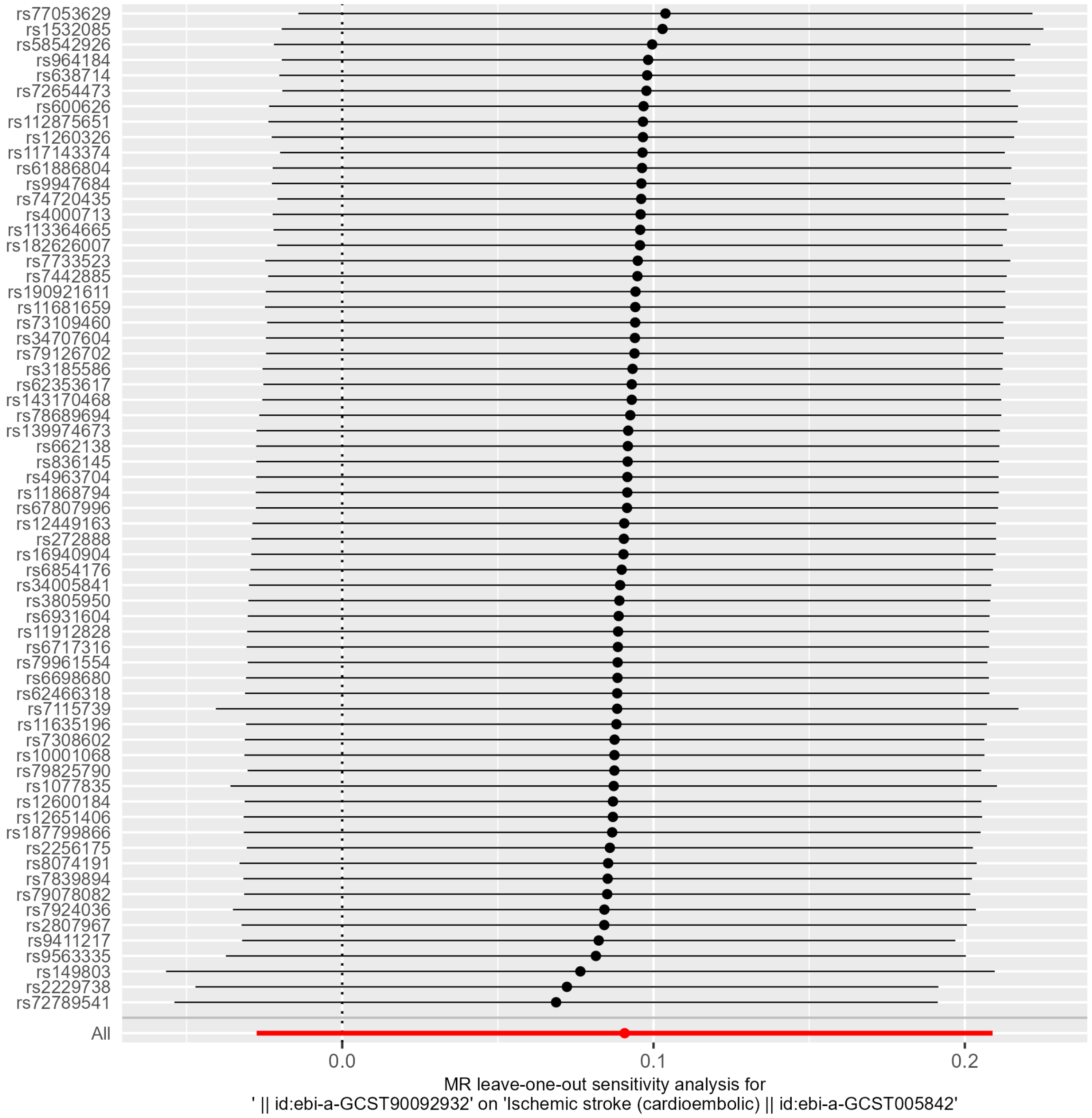

Ω-3 rate-IS leave-one-out

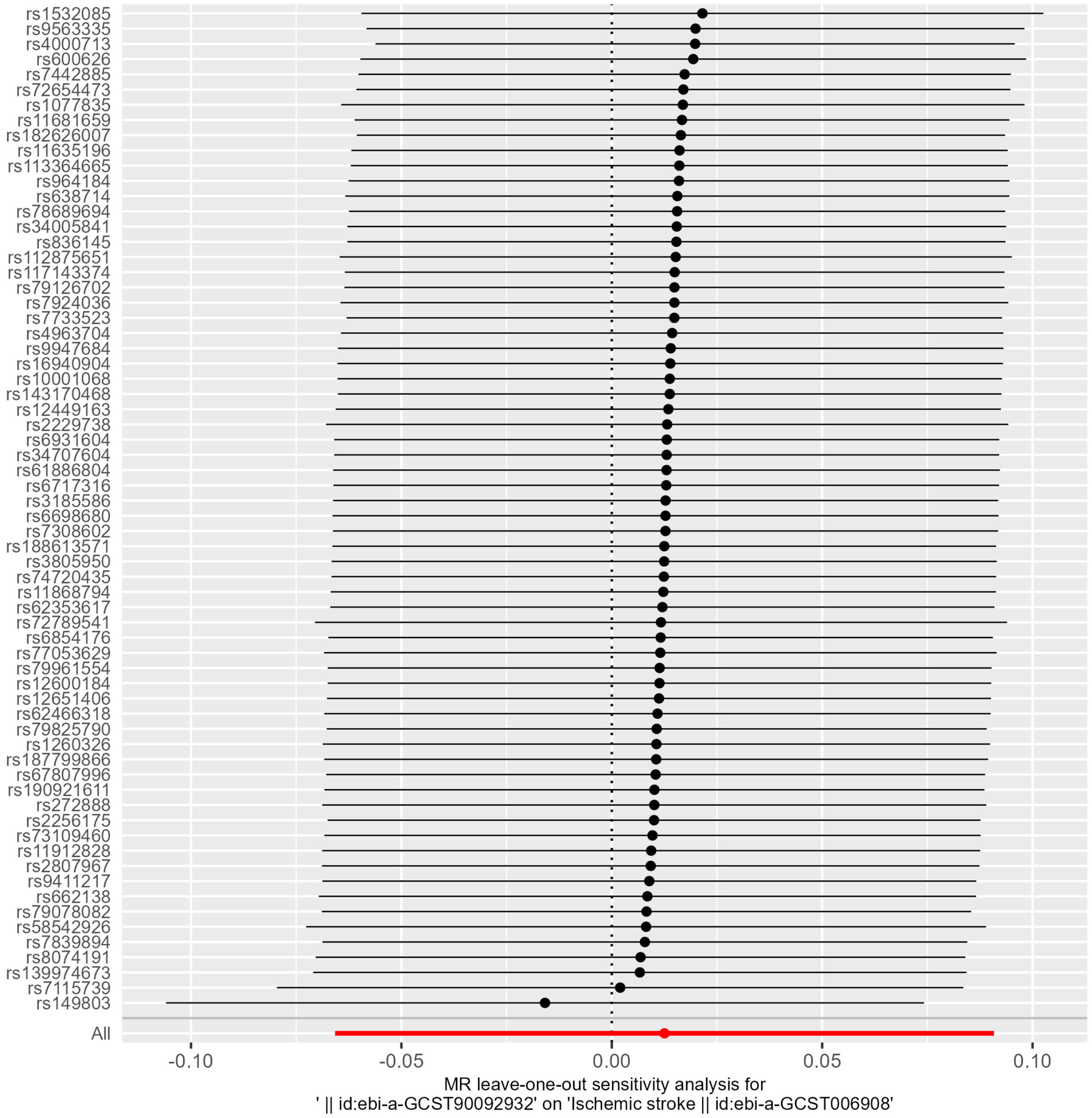

Ω-3 rate-LS leave-one-out

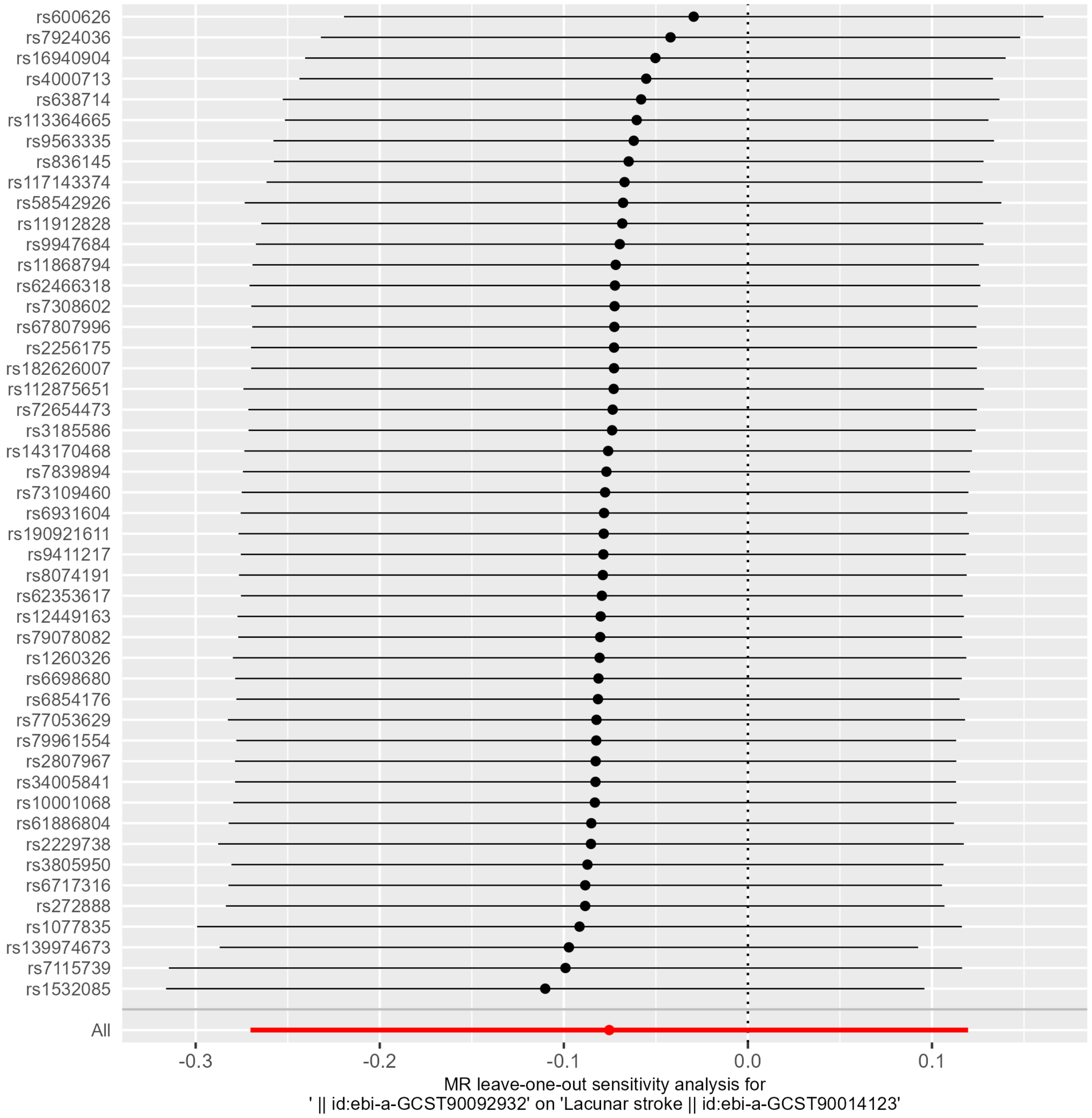

Ω-3 rate-DBP leave-one-out

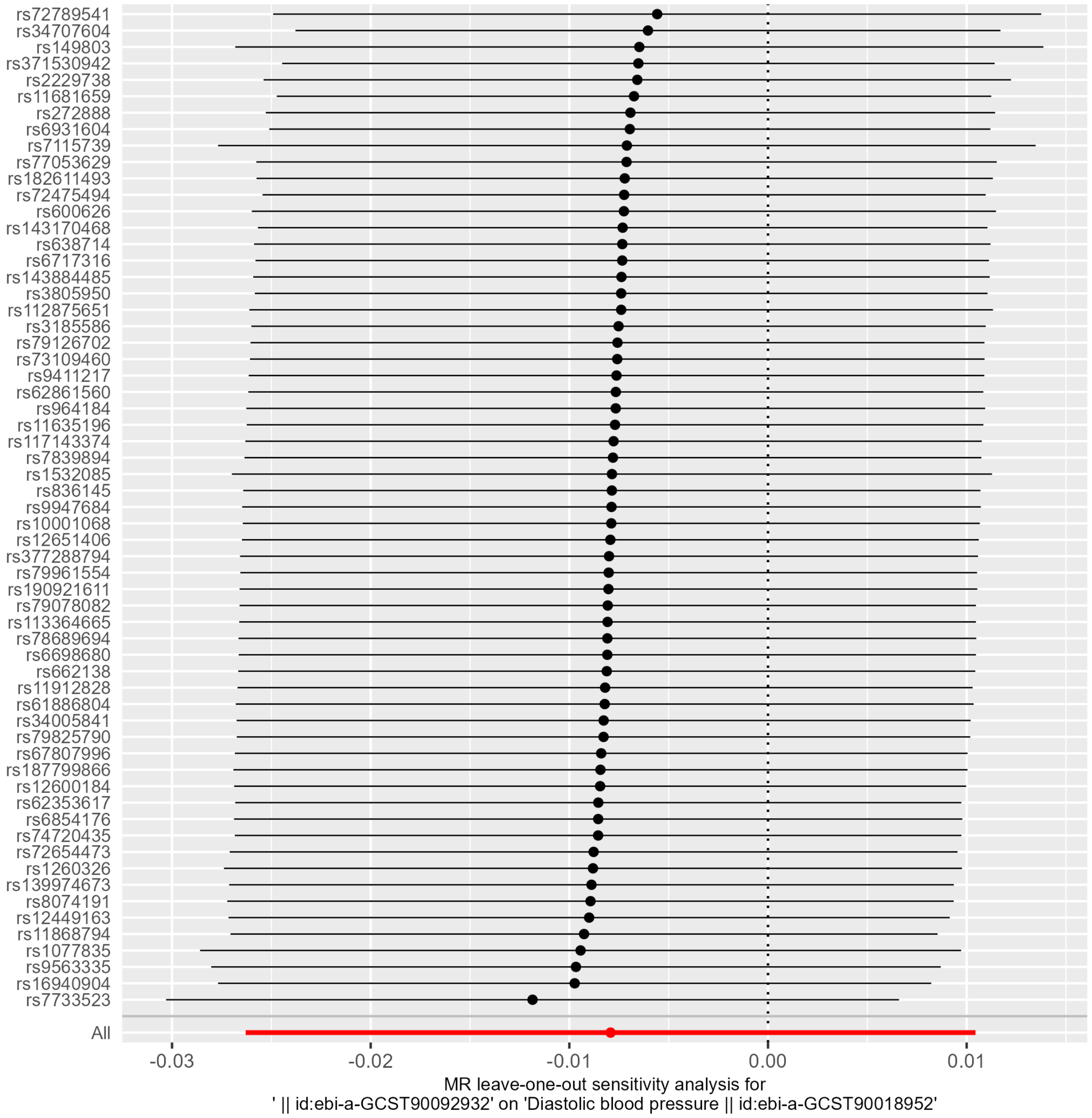

Ω-3 rate-SBP leave-one-out

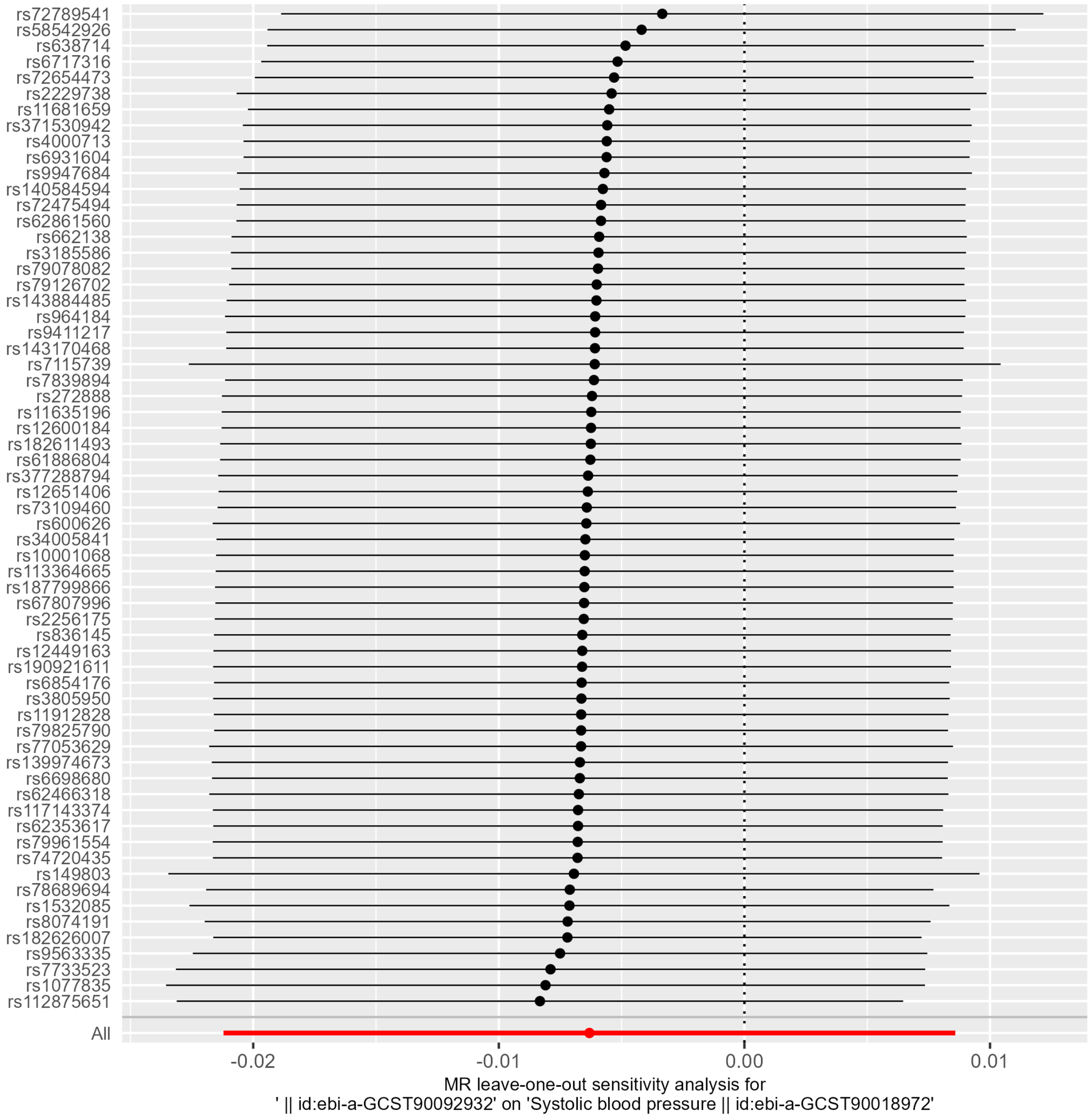

Ω-6|Ω-3-LAS leave-one-out

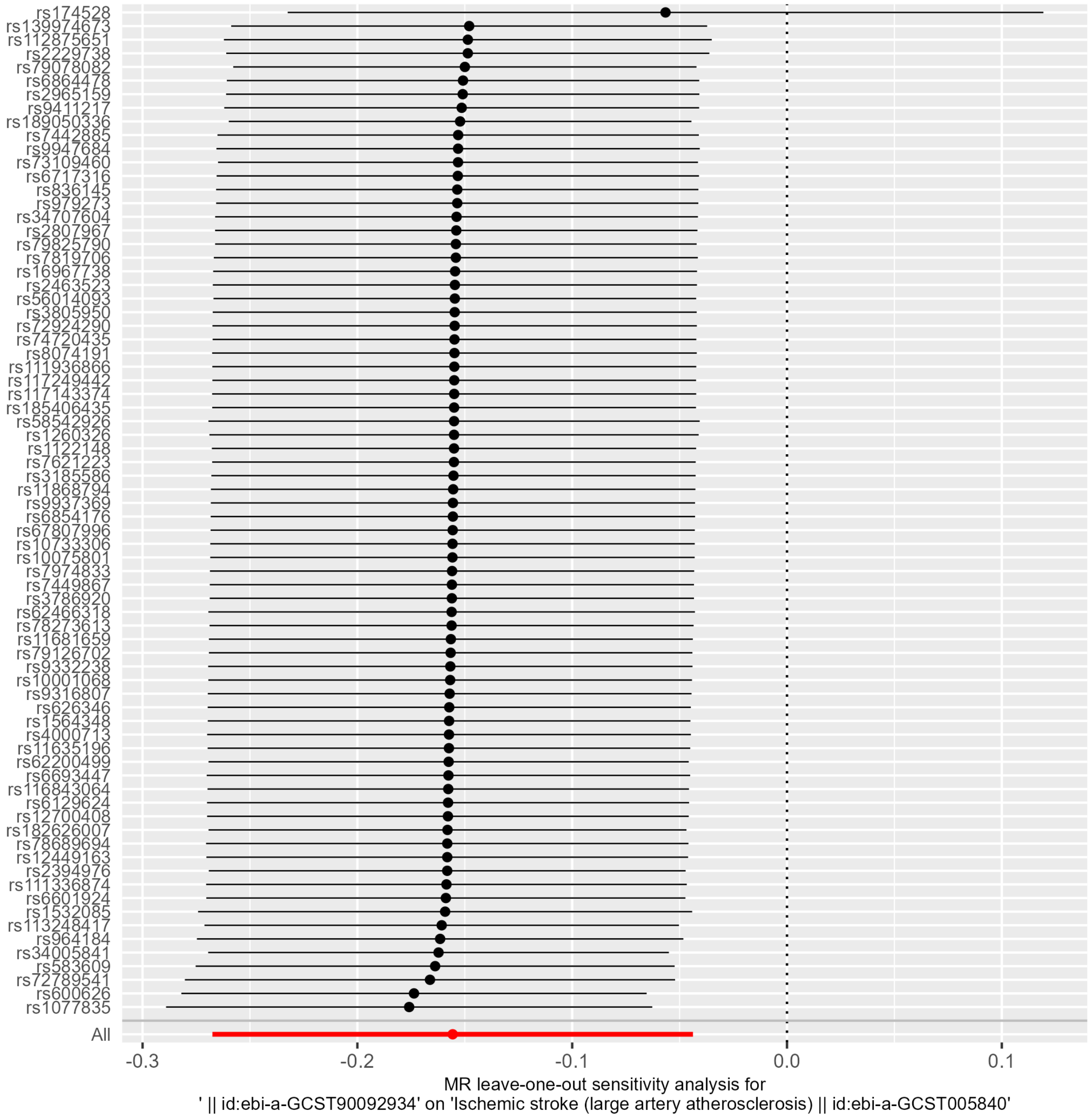

Ω-6|Ω-3-SVS leave-one-out

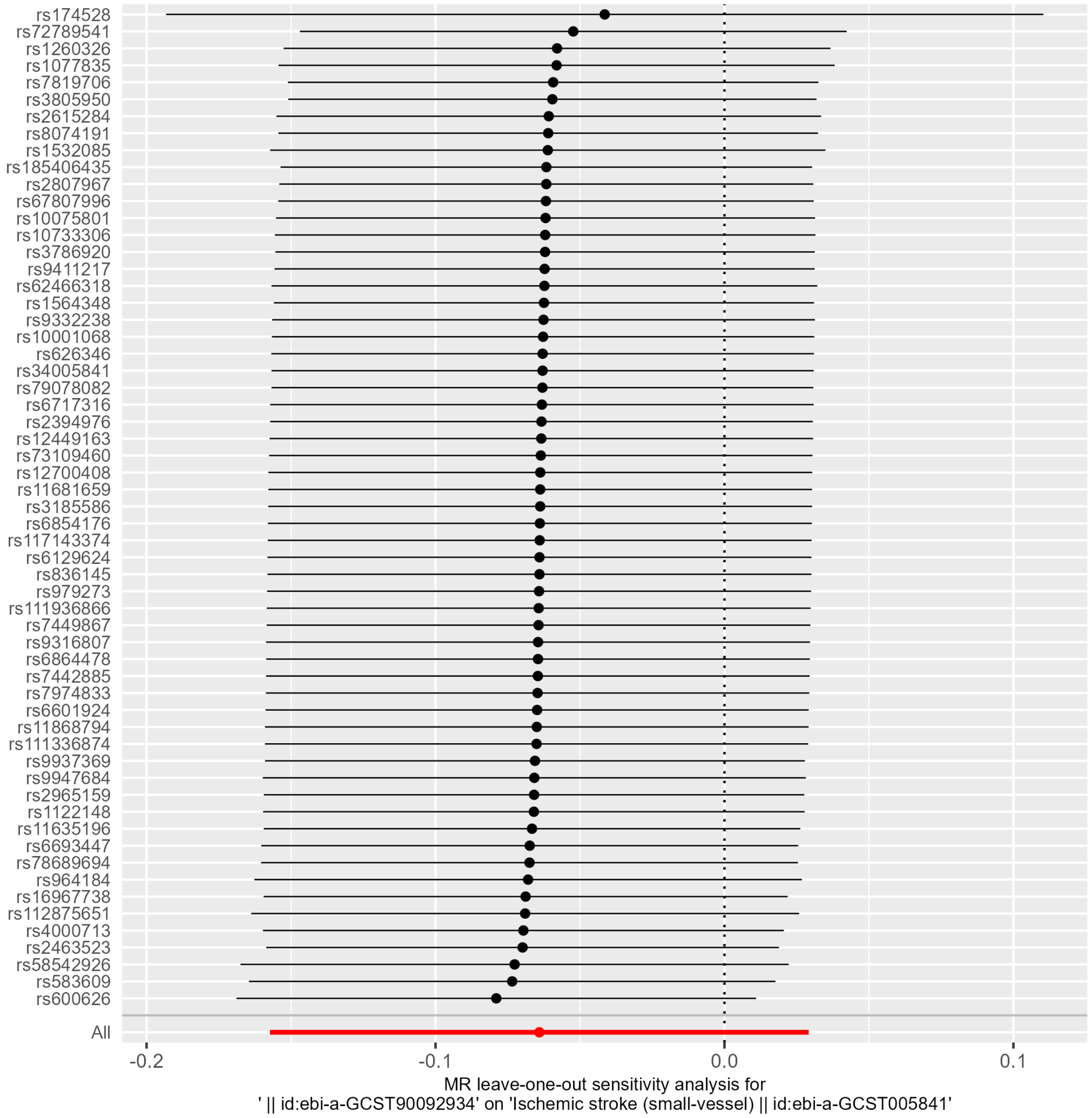

Ω-6|Ω-3-CES leave-one-out

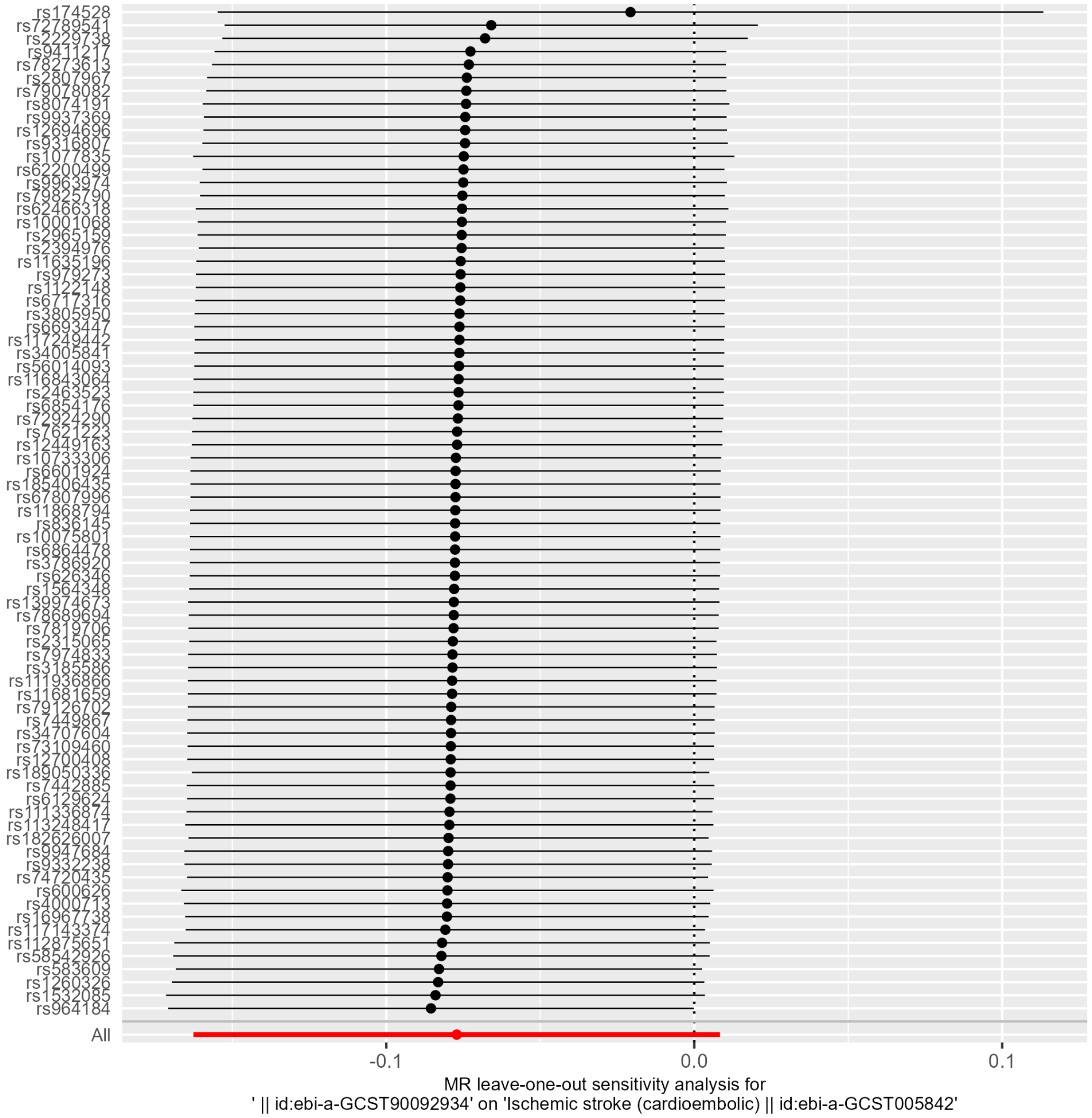

Ω-6|Ω-3-IS leave-one-out

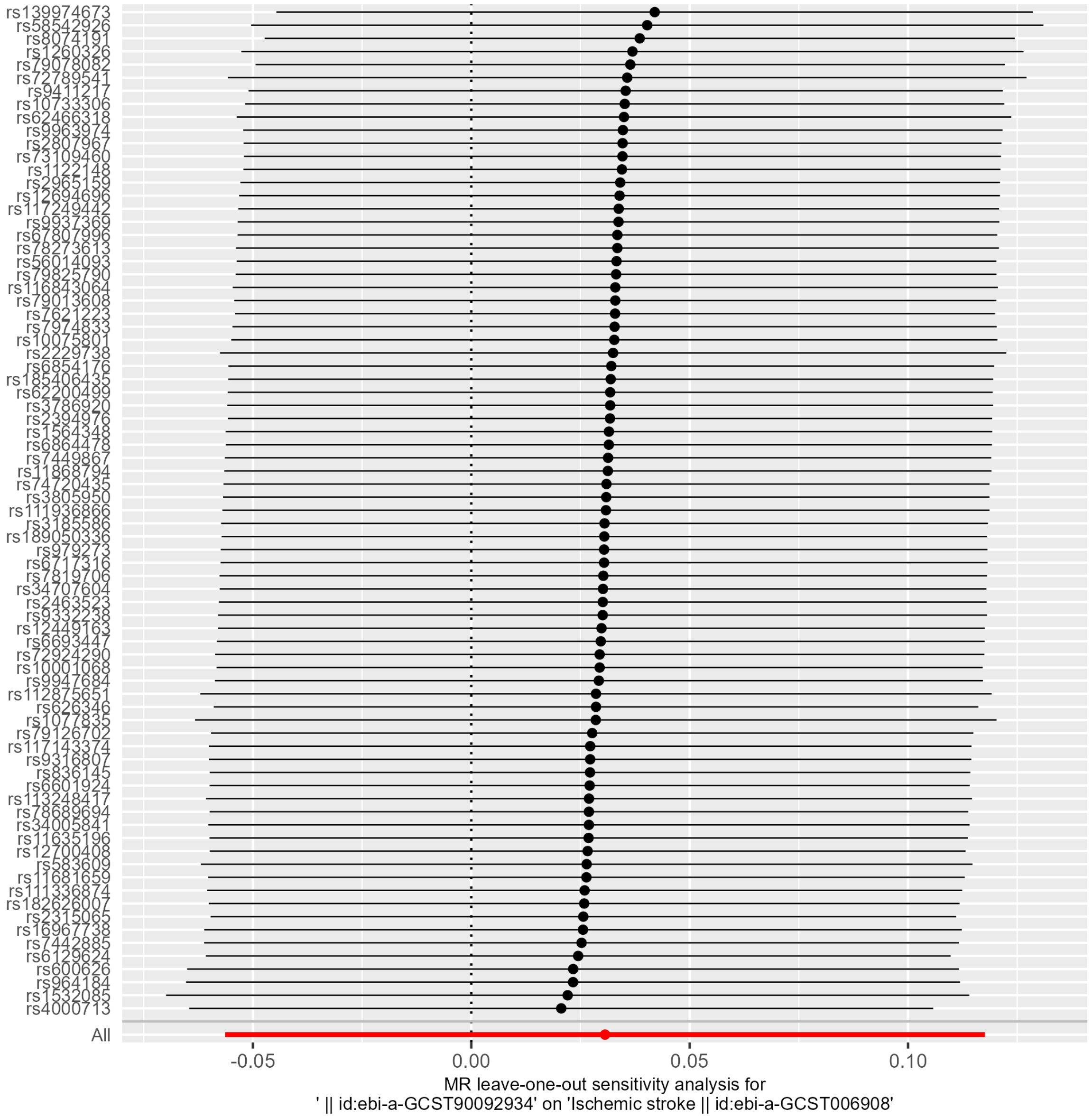

Ω-6|Ω-3-LS leave-one-out

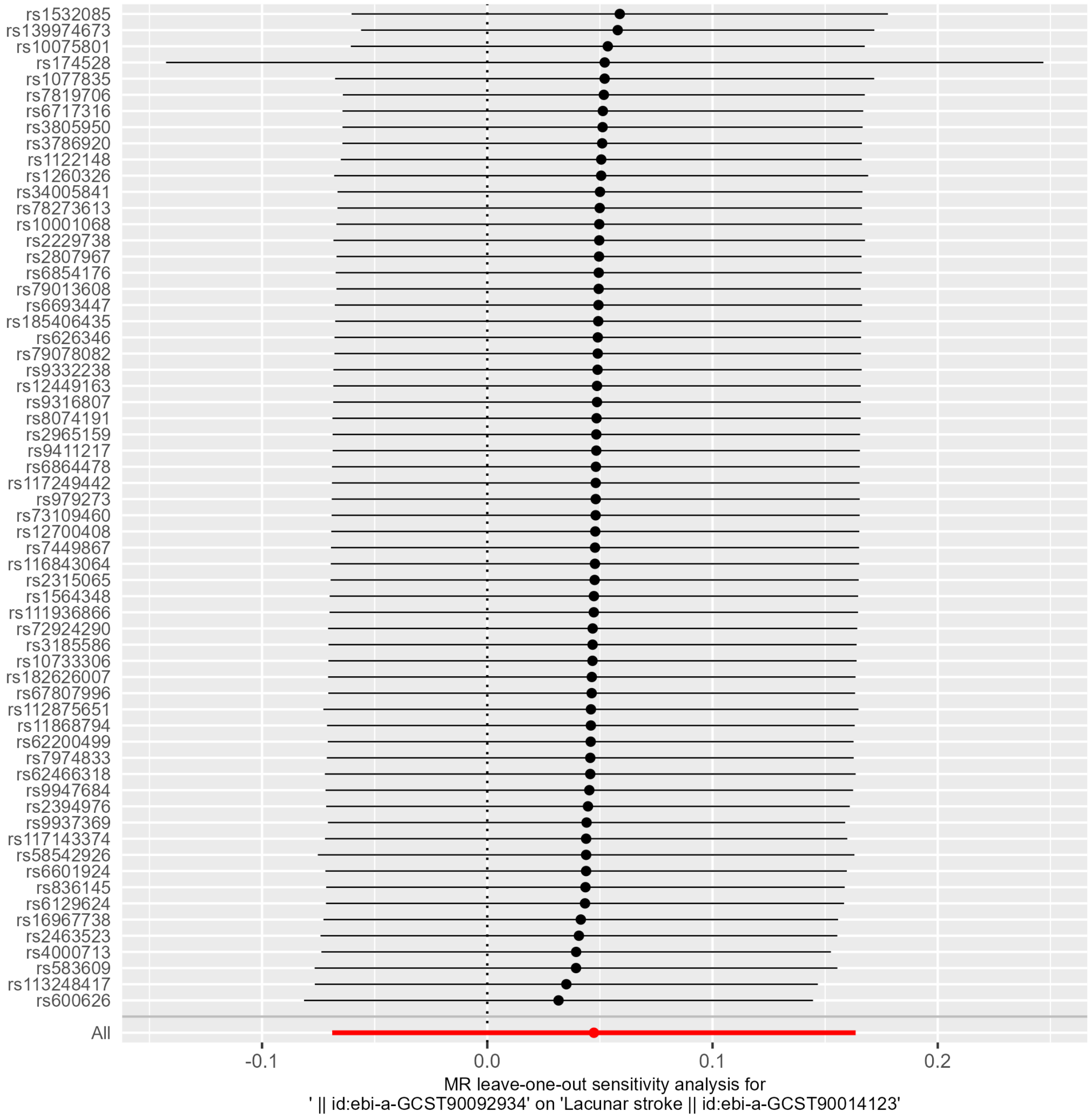

Ω-6|Ω-3-DBP leave-one-out

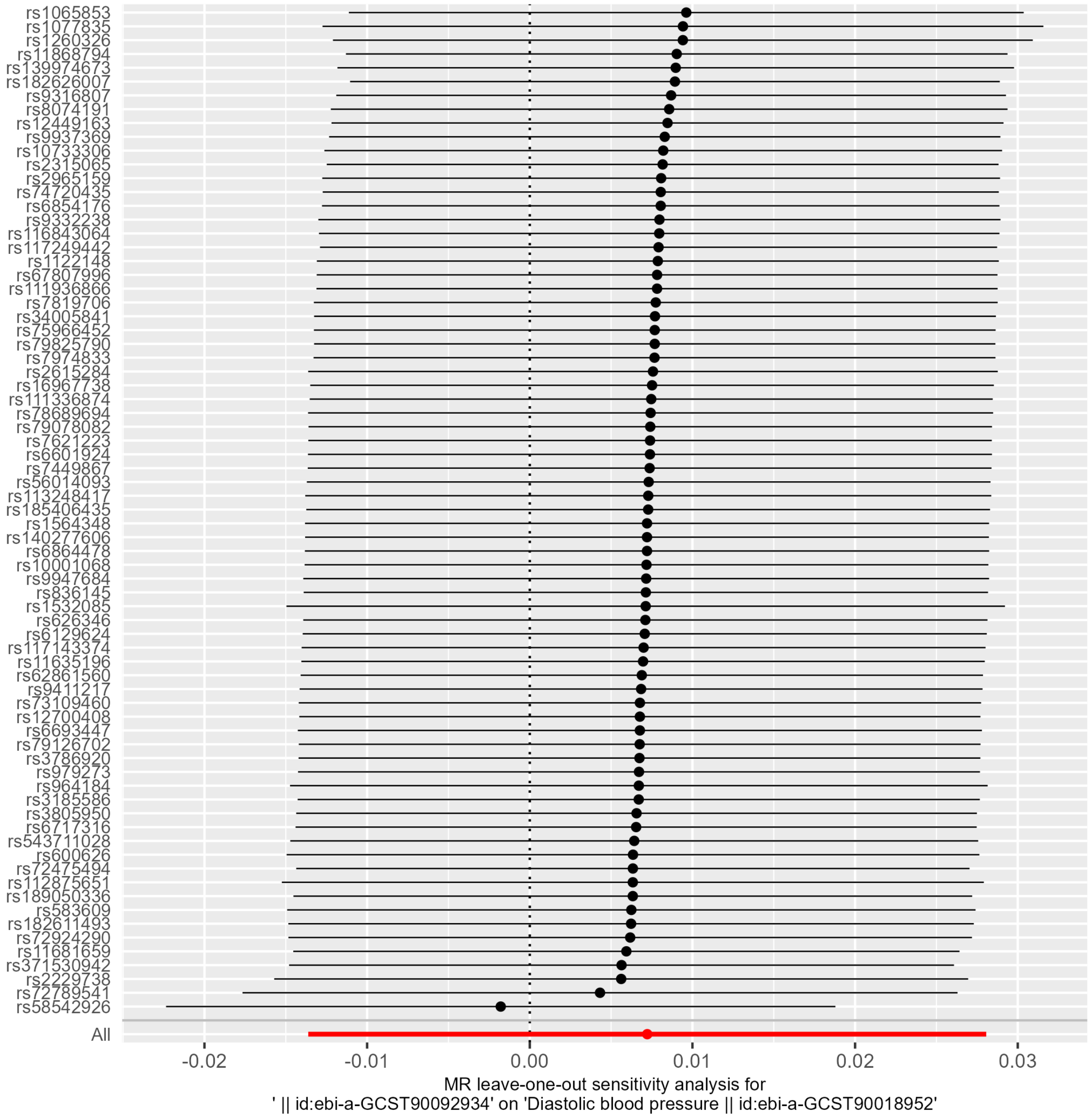

Ω-6|Ω-3-SBP leave-one-out

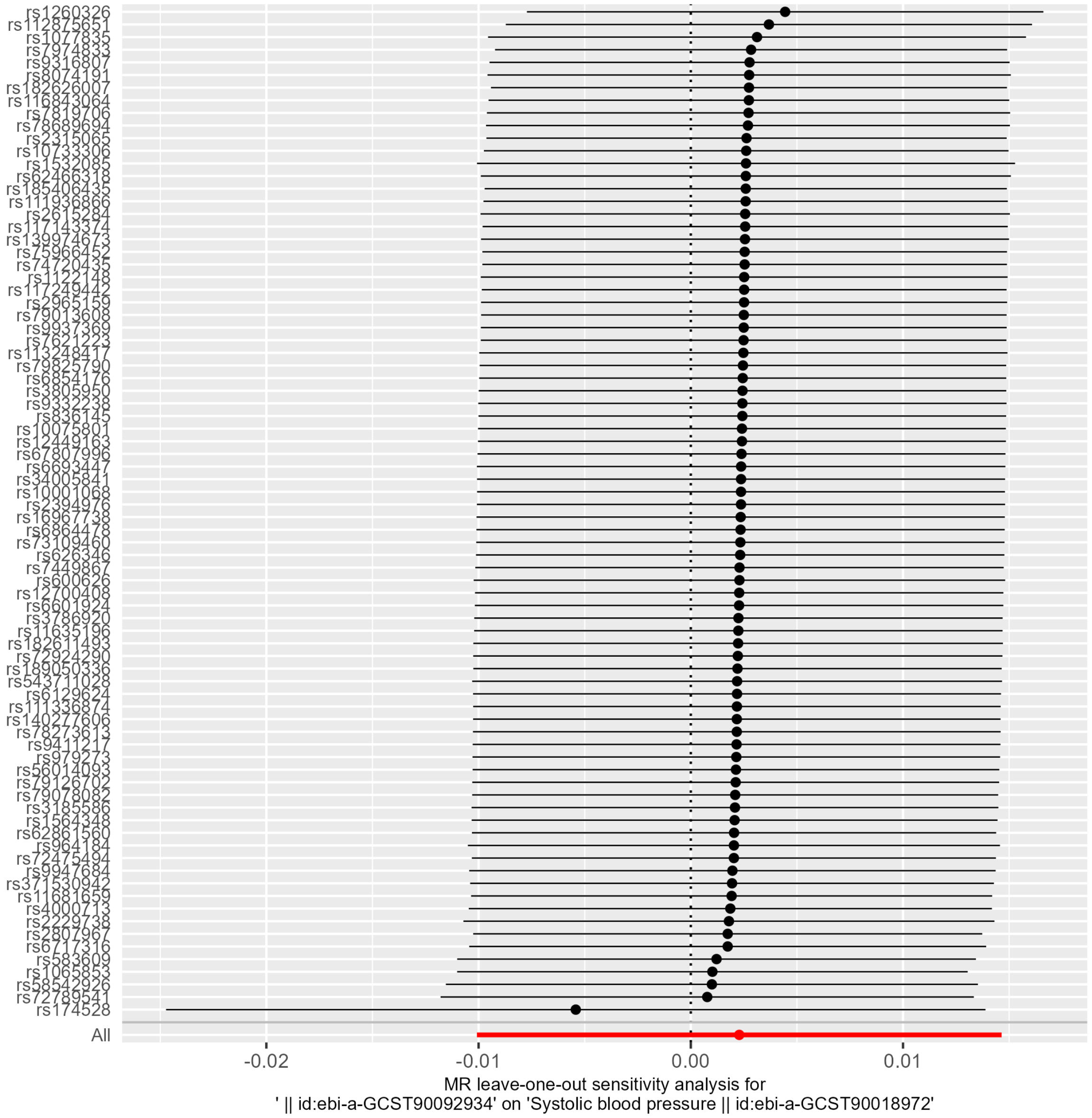

DHA-LAS scatter plot

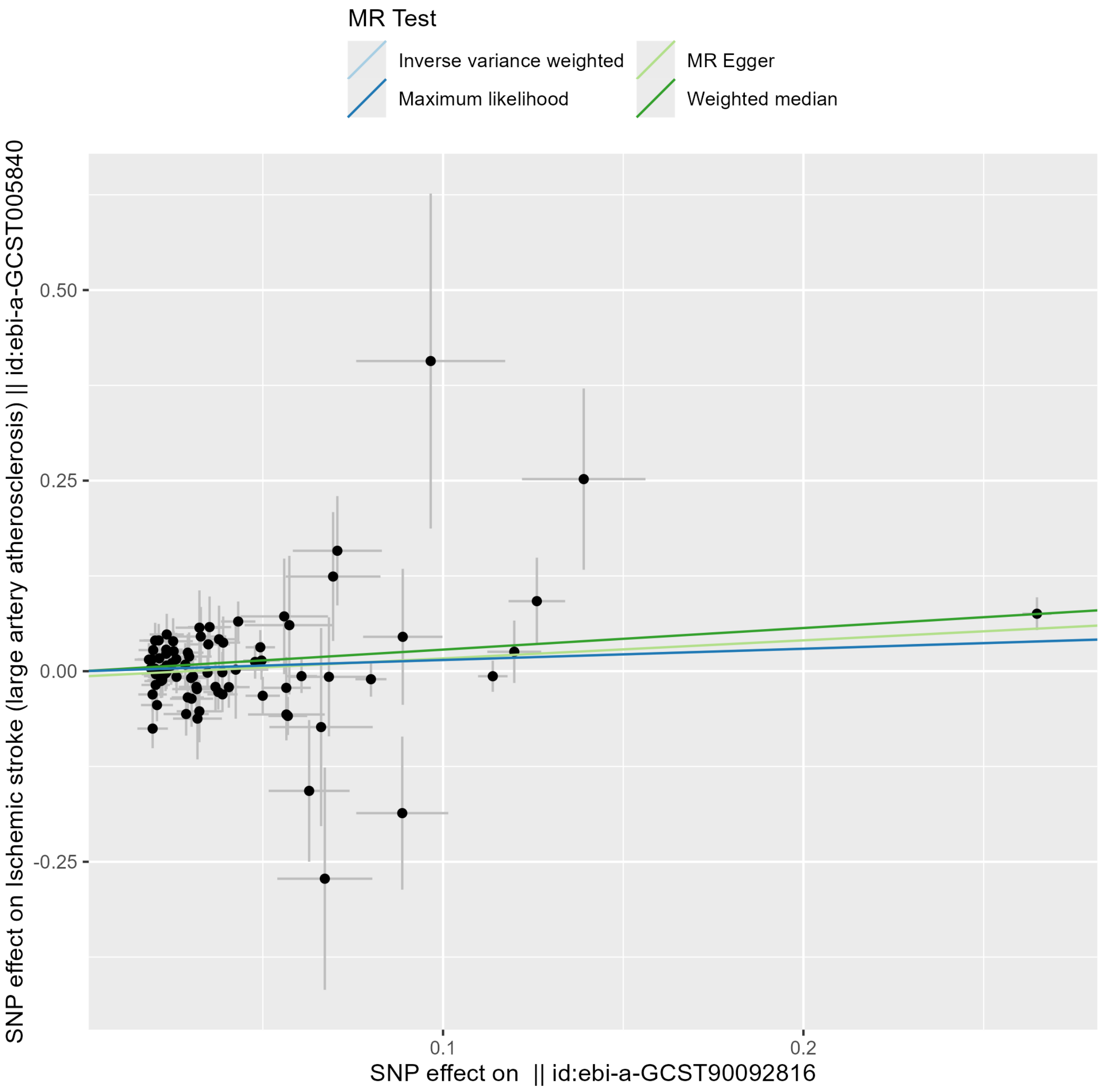

DHA-SVS scatter plot

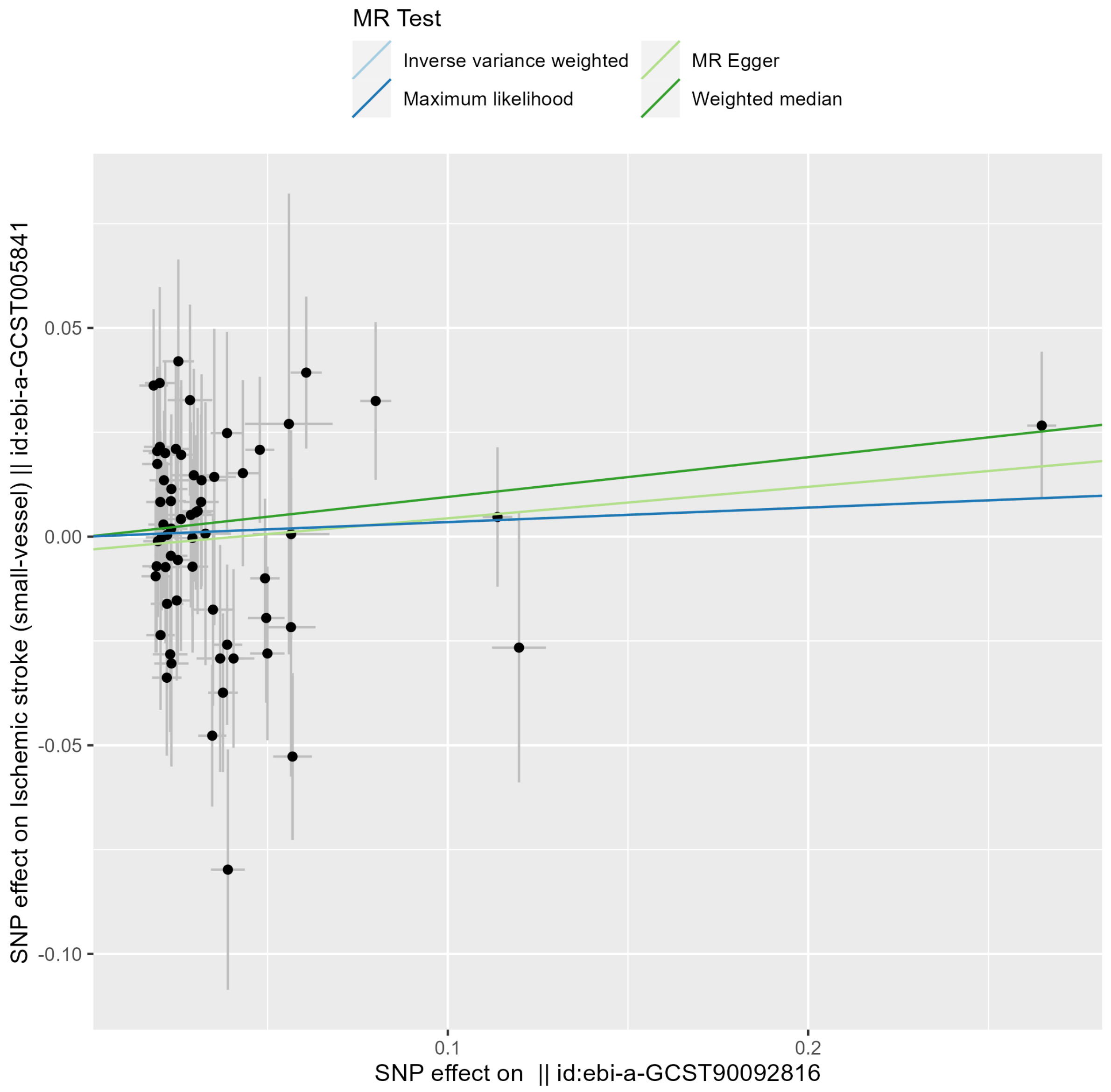

DHA-CES scatter plot

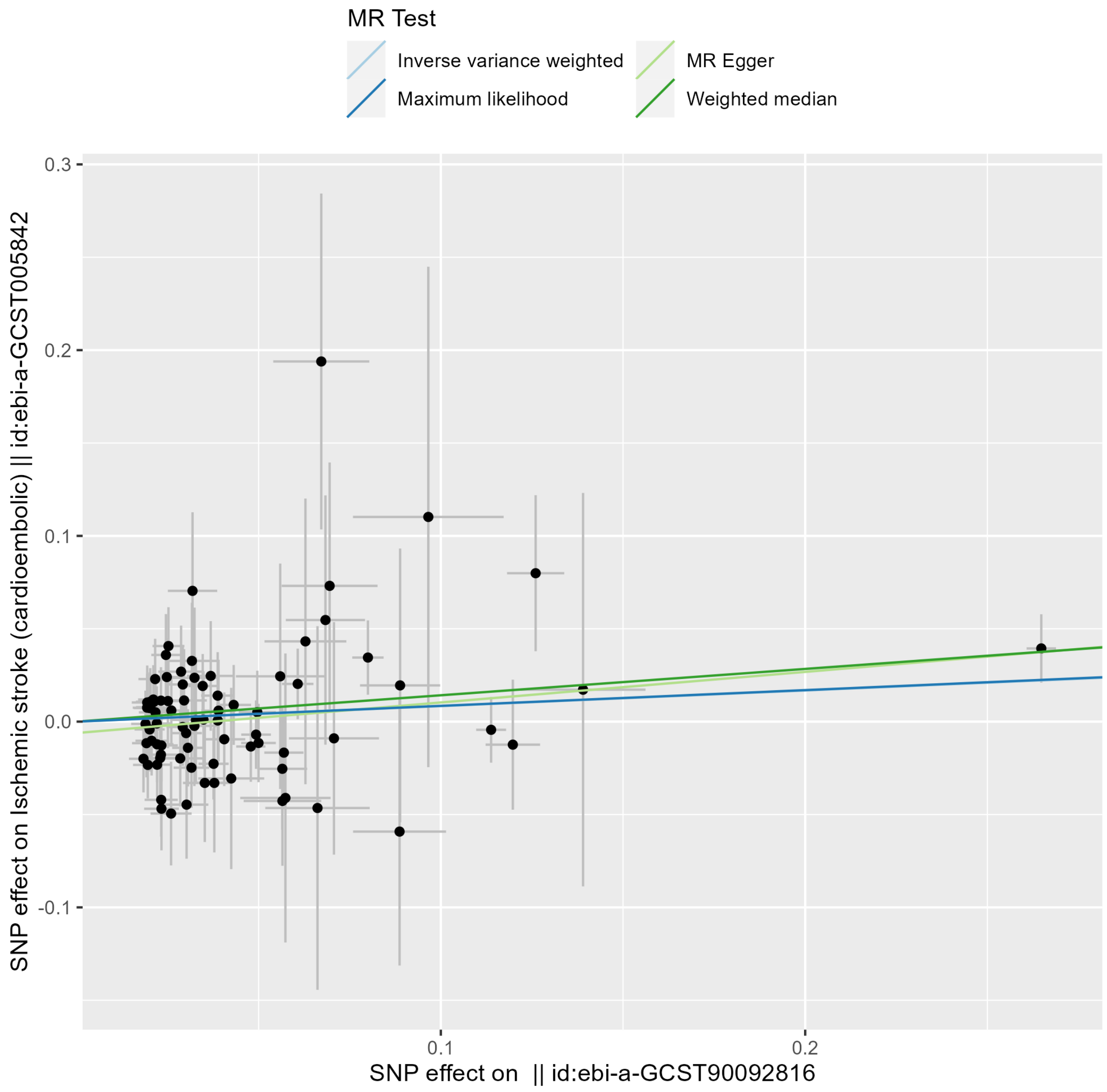

DHA-IS scatter plot

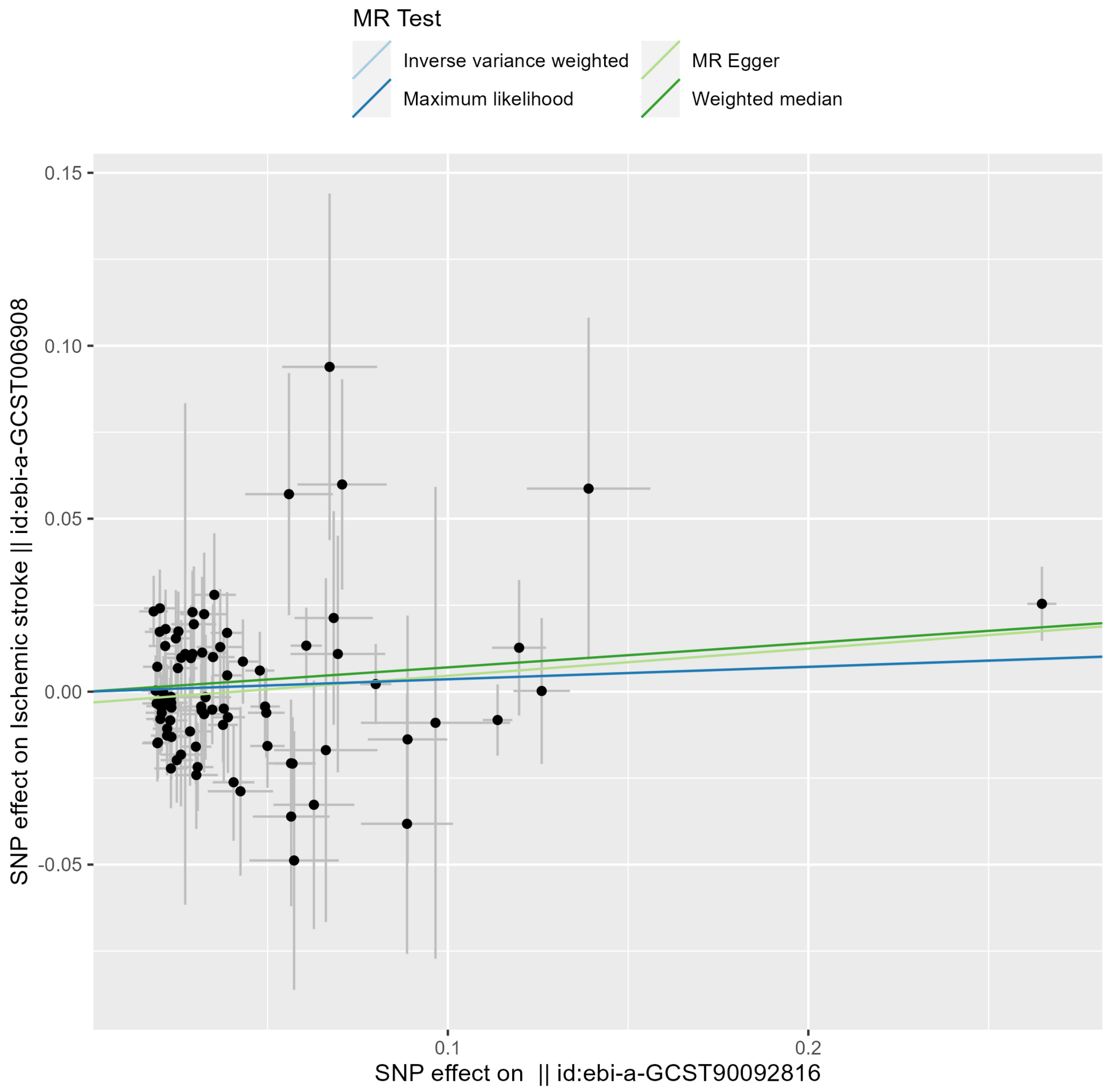

DHA-LS scatter plot

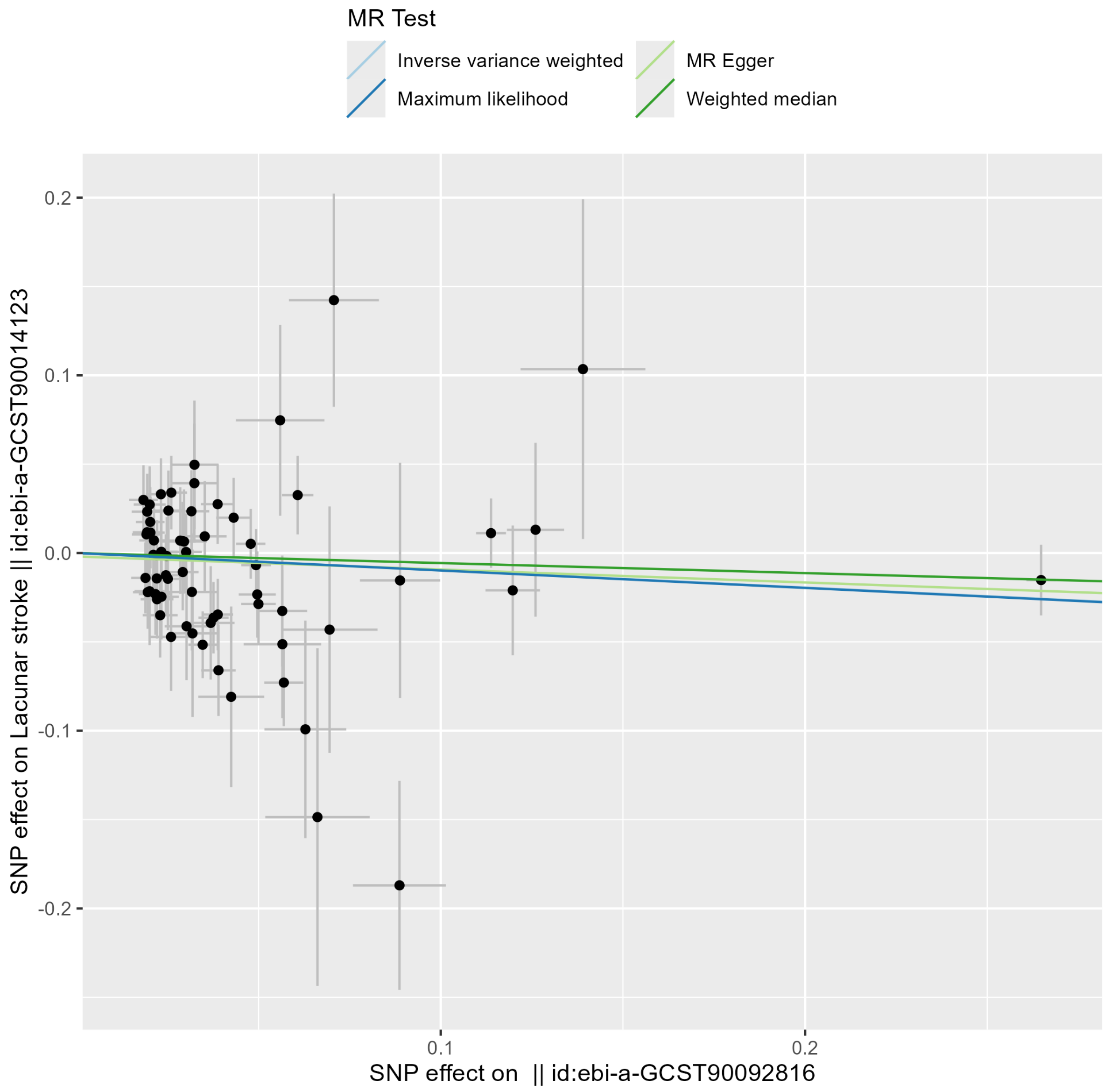

DHA-DBP scatter plot

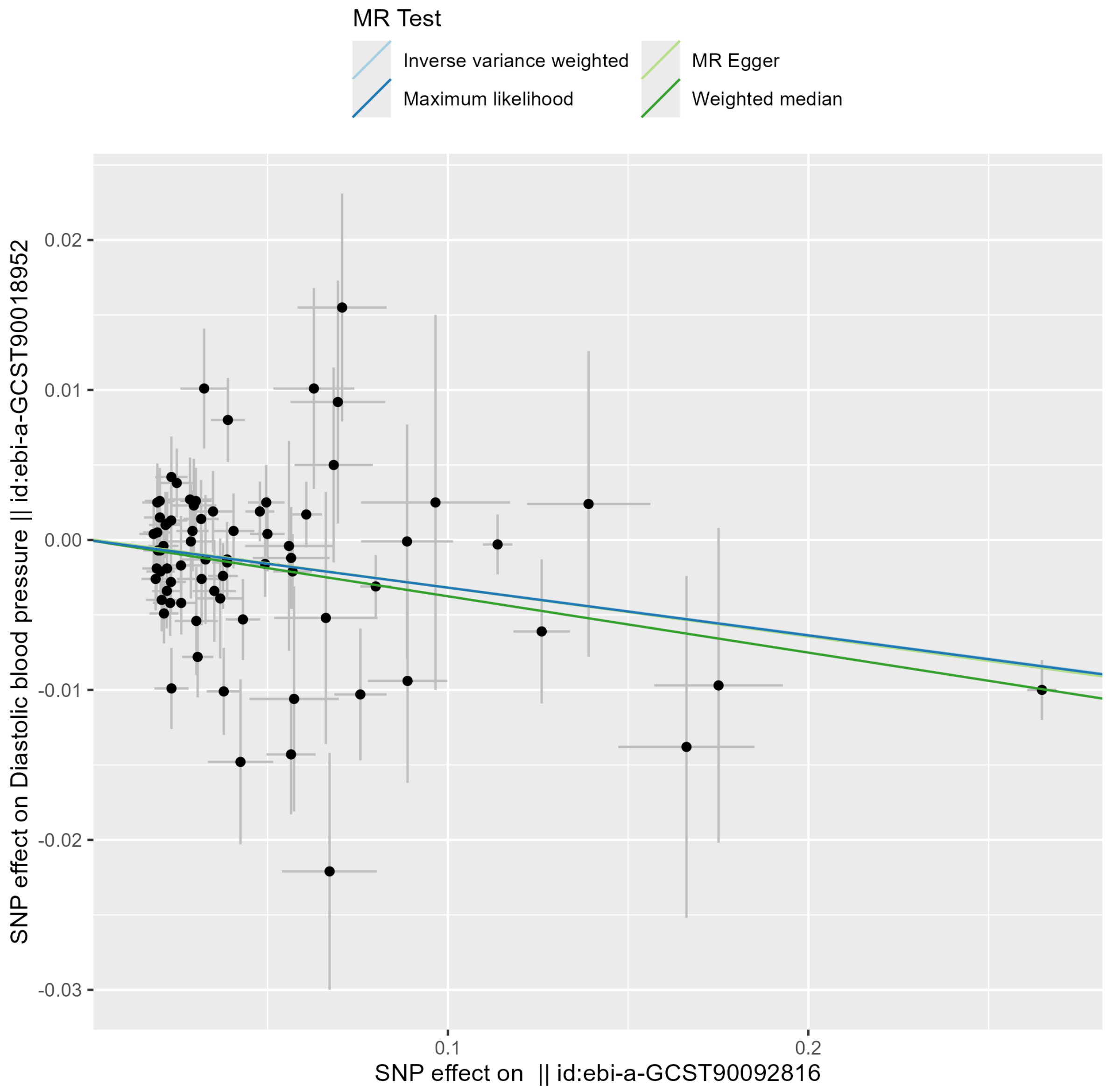

DHA-SBP scatter plot

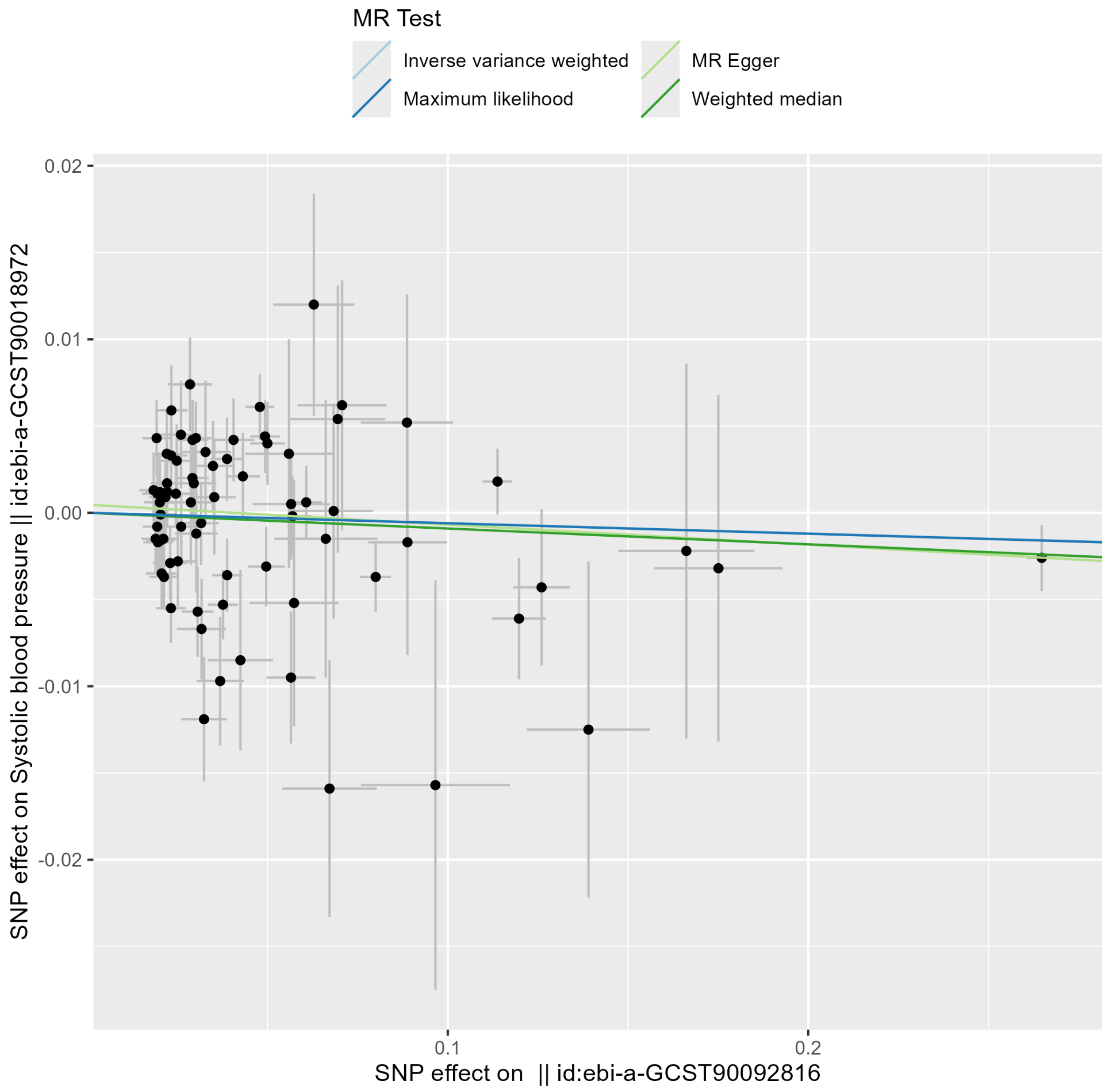

Ω-3 rate-LAS scatter plot

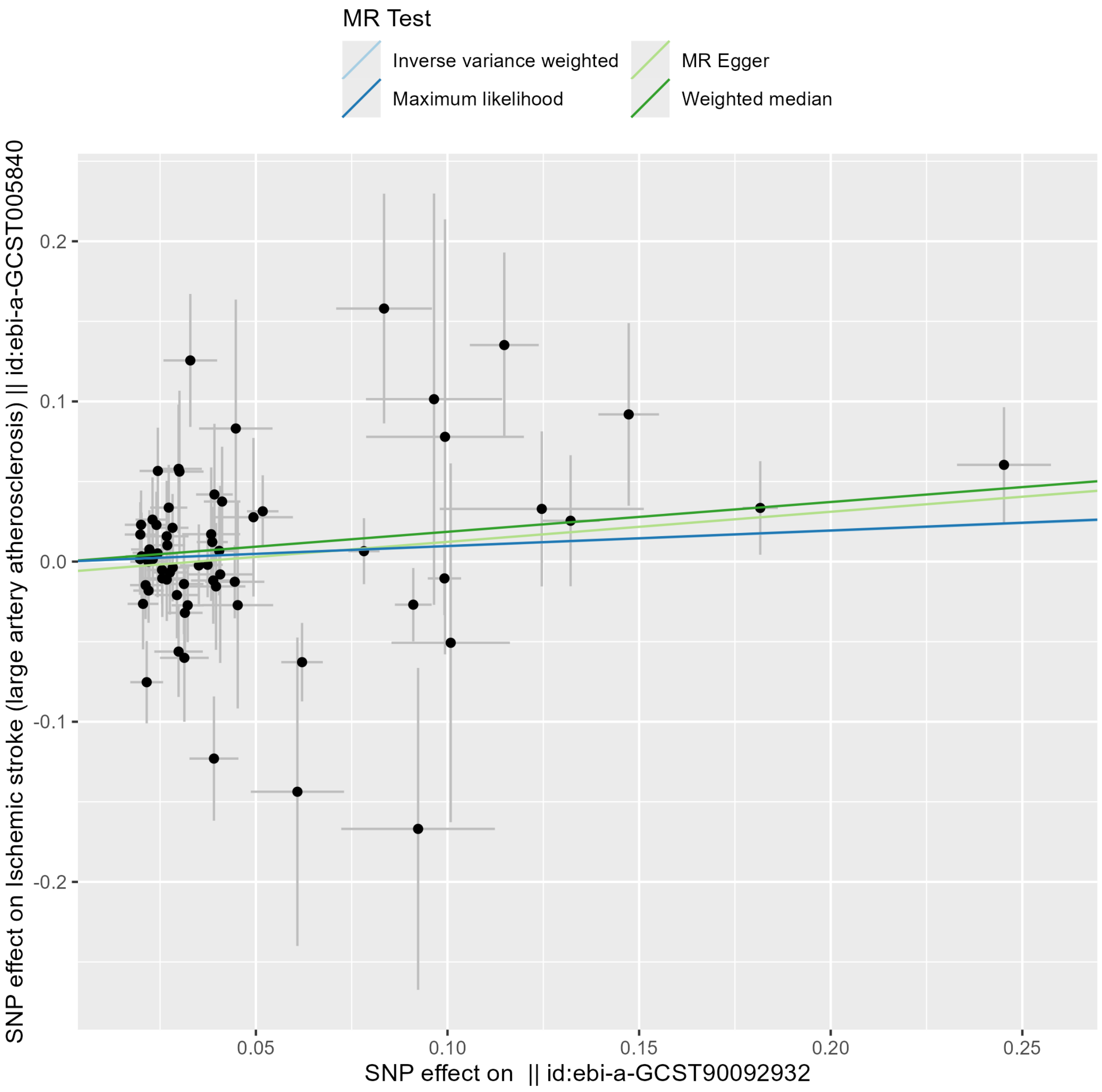

Ω-3 rate-SVS scatter plot

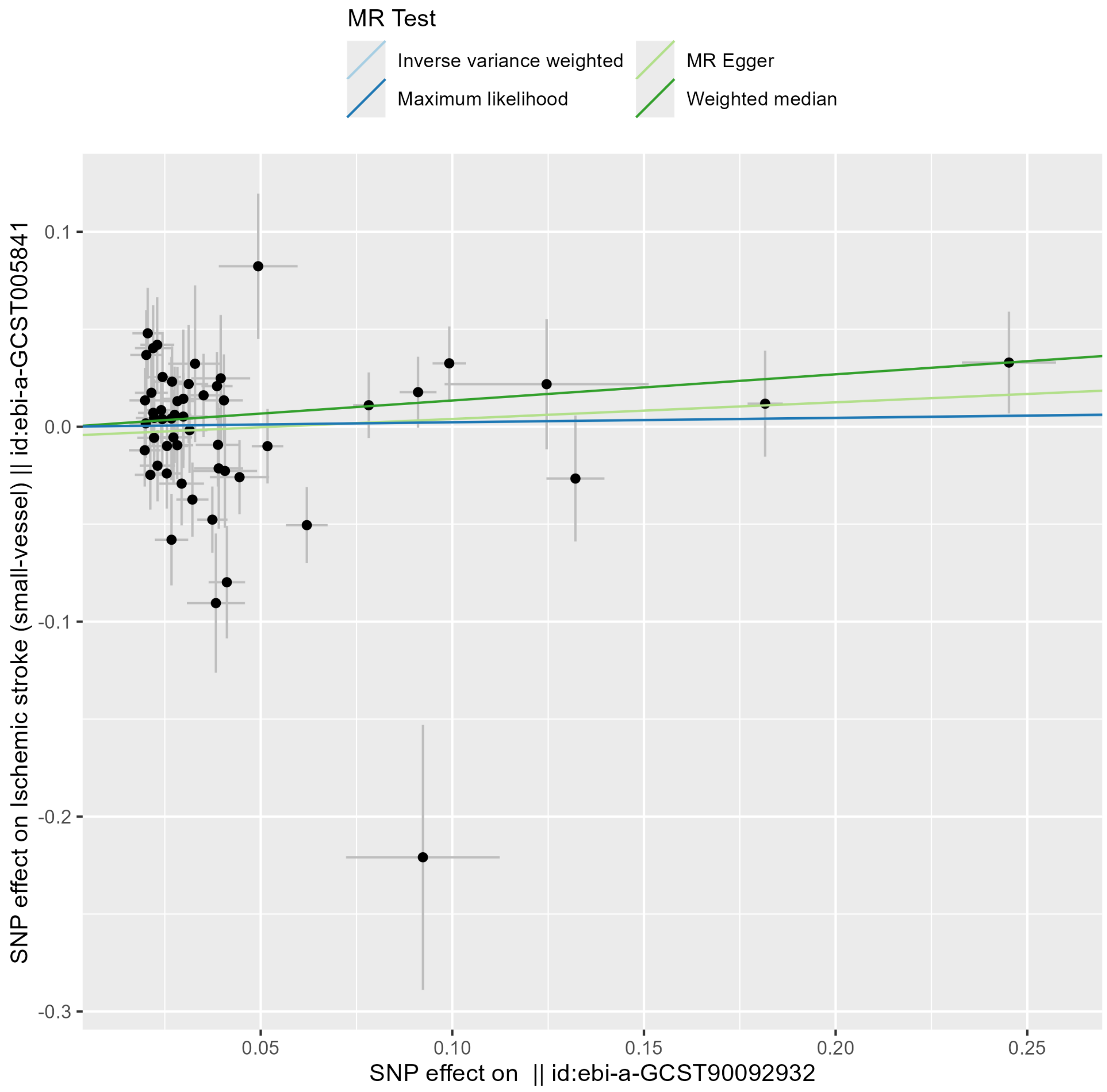

Ω-3 rate-CES scatter plot

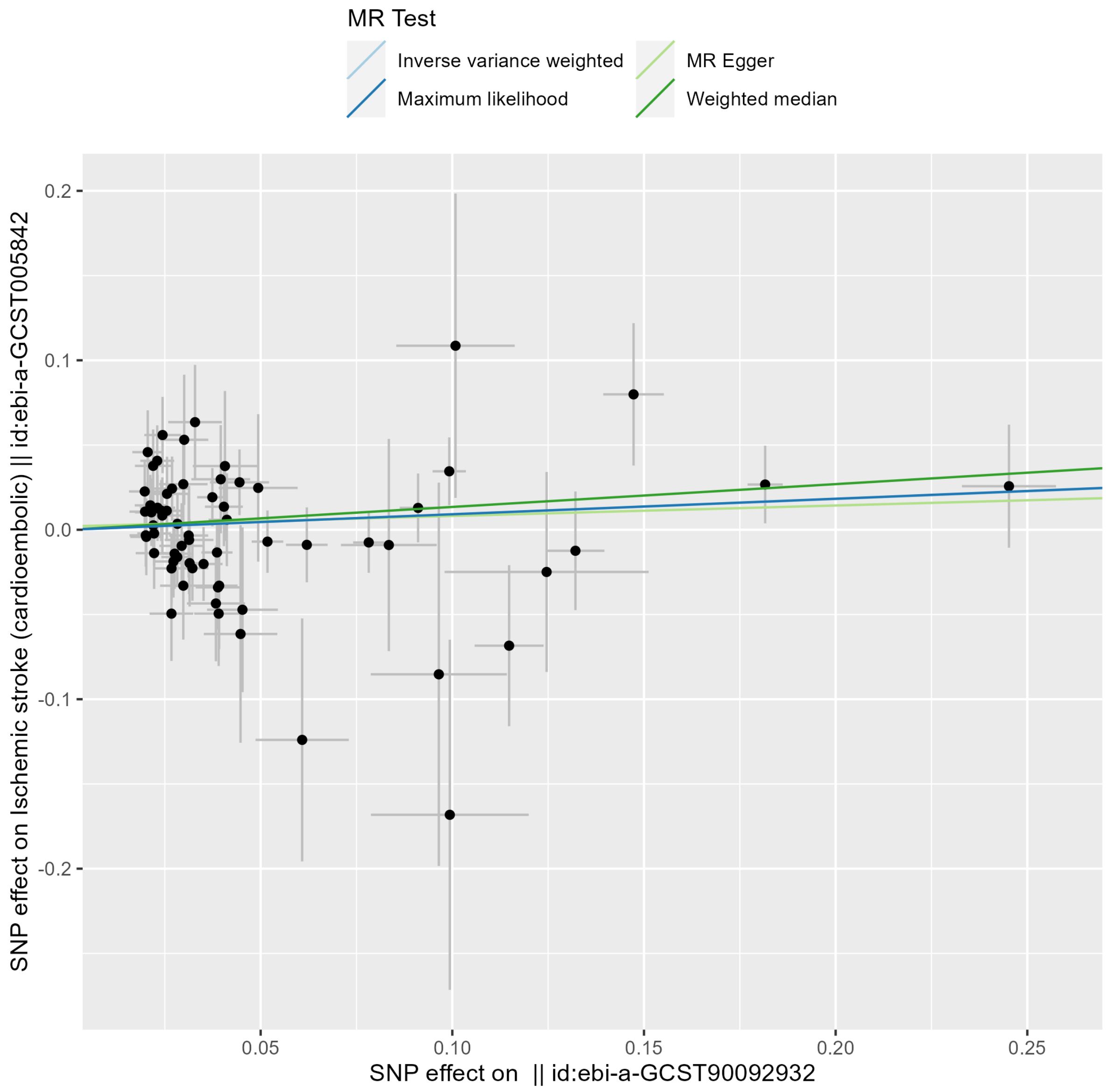

Ω-3 rate-IS scatter plot

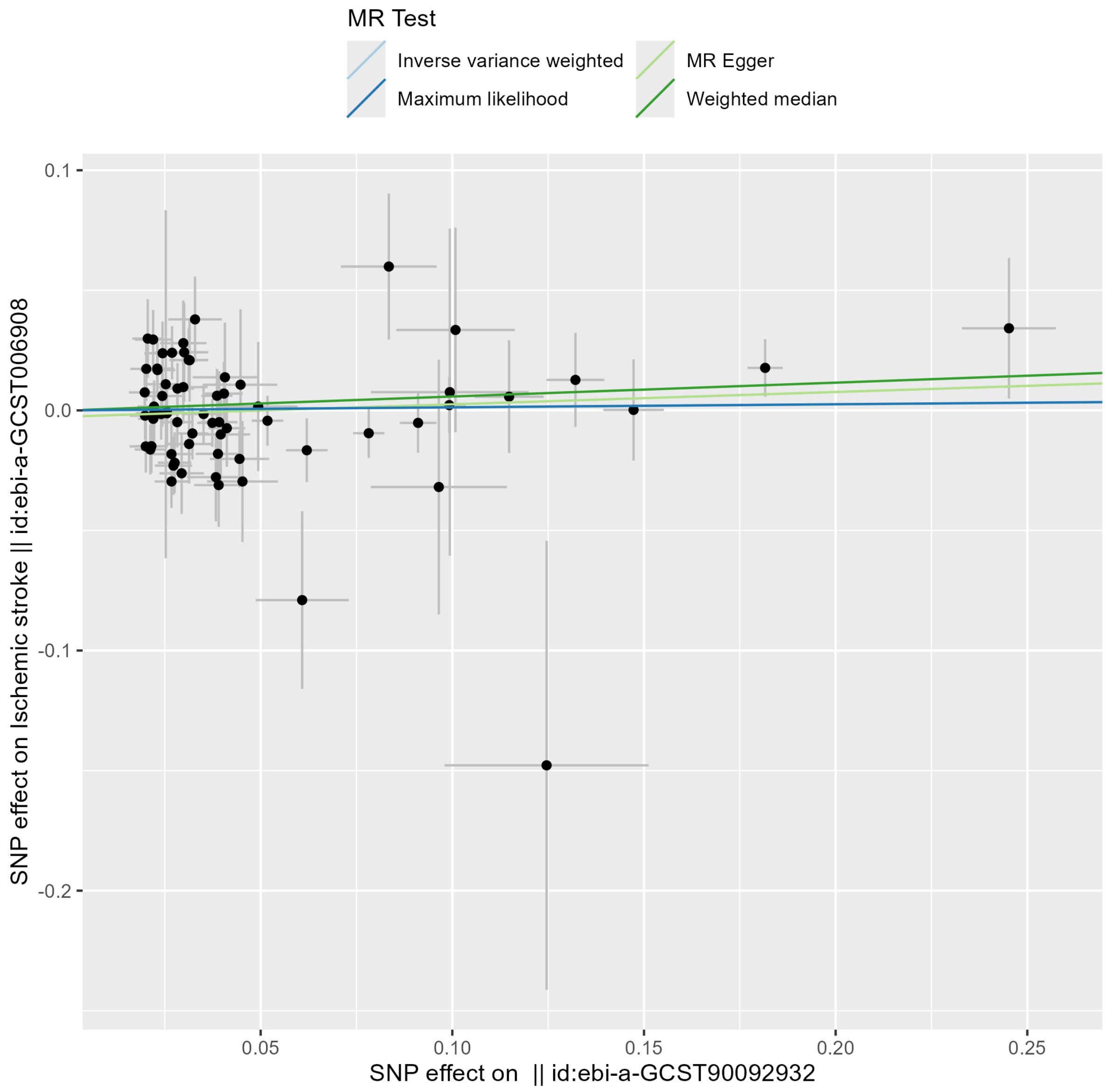

Ω-3 rate-LS scatter plot

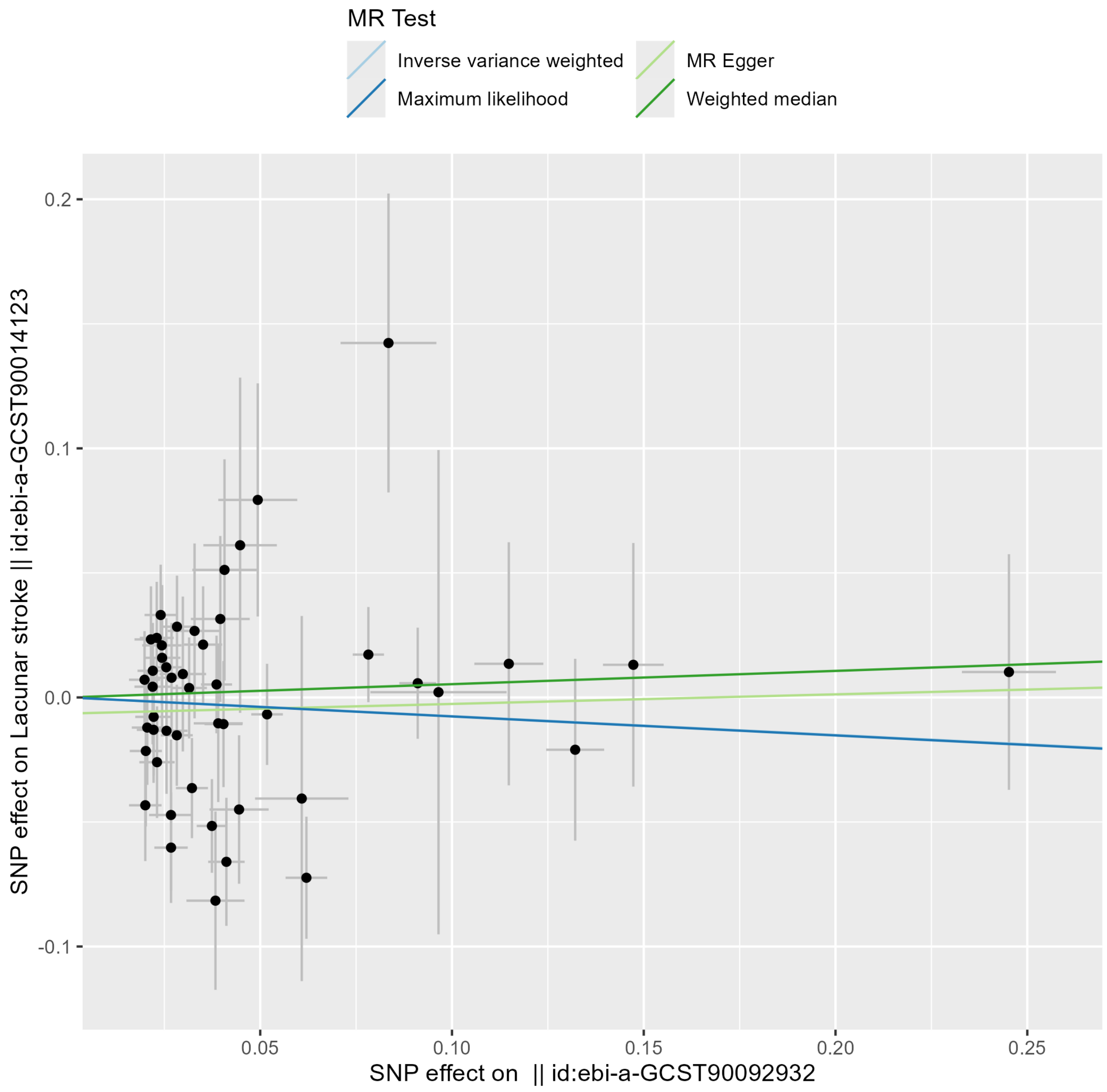

Ω-3 rate-DBP scatter plot

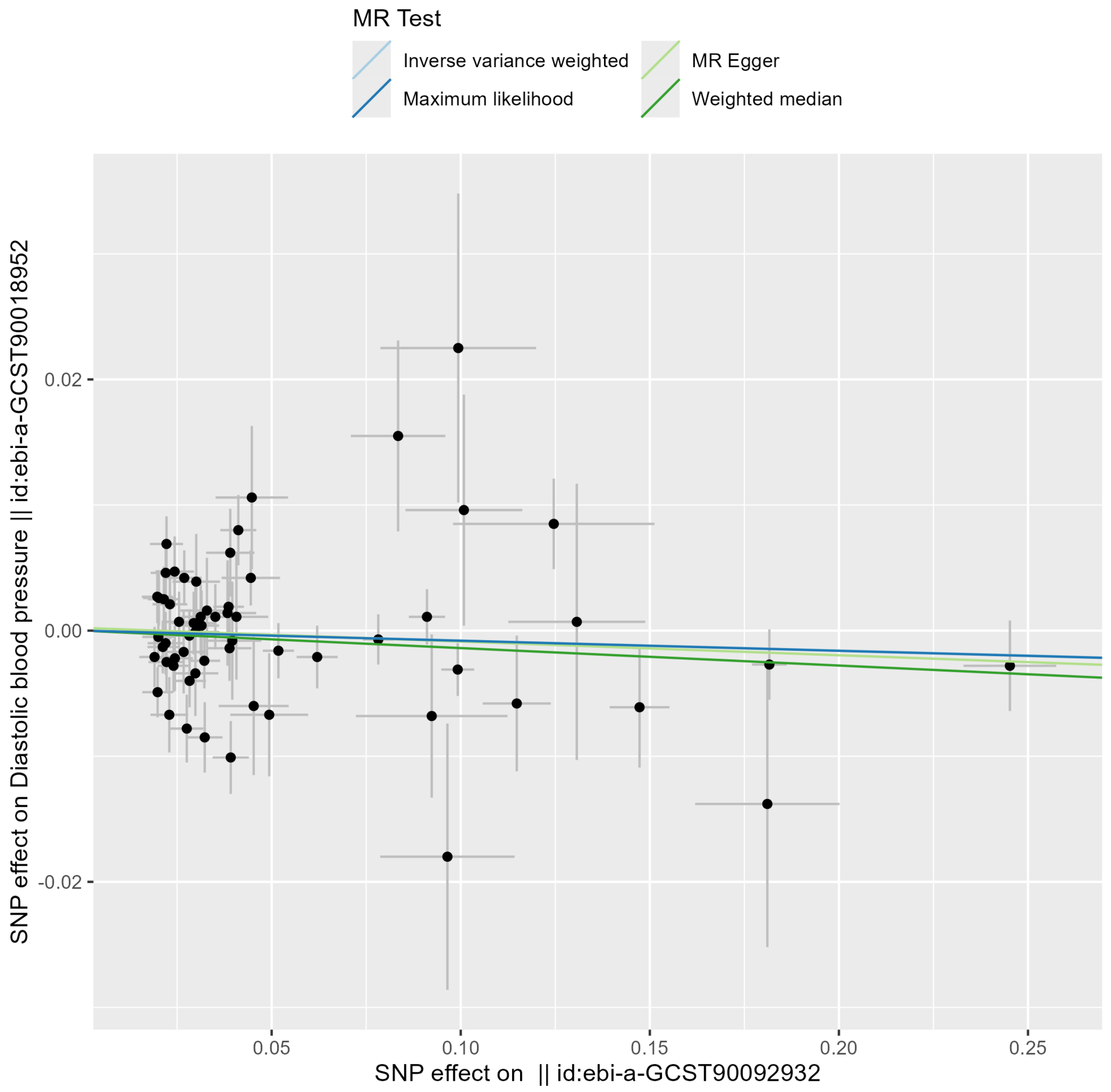

Ω-3 rate-SBP scatter plot

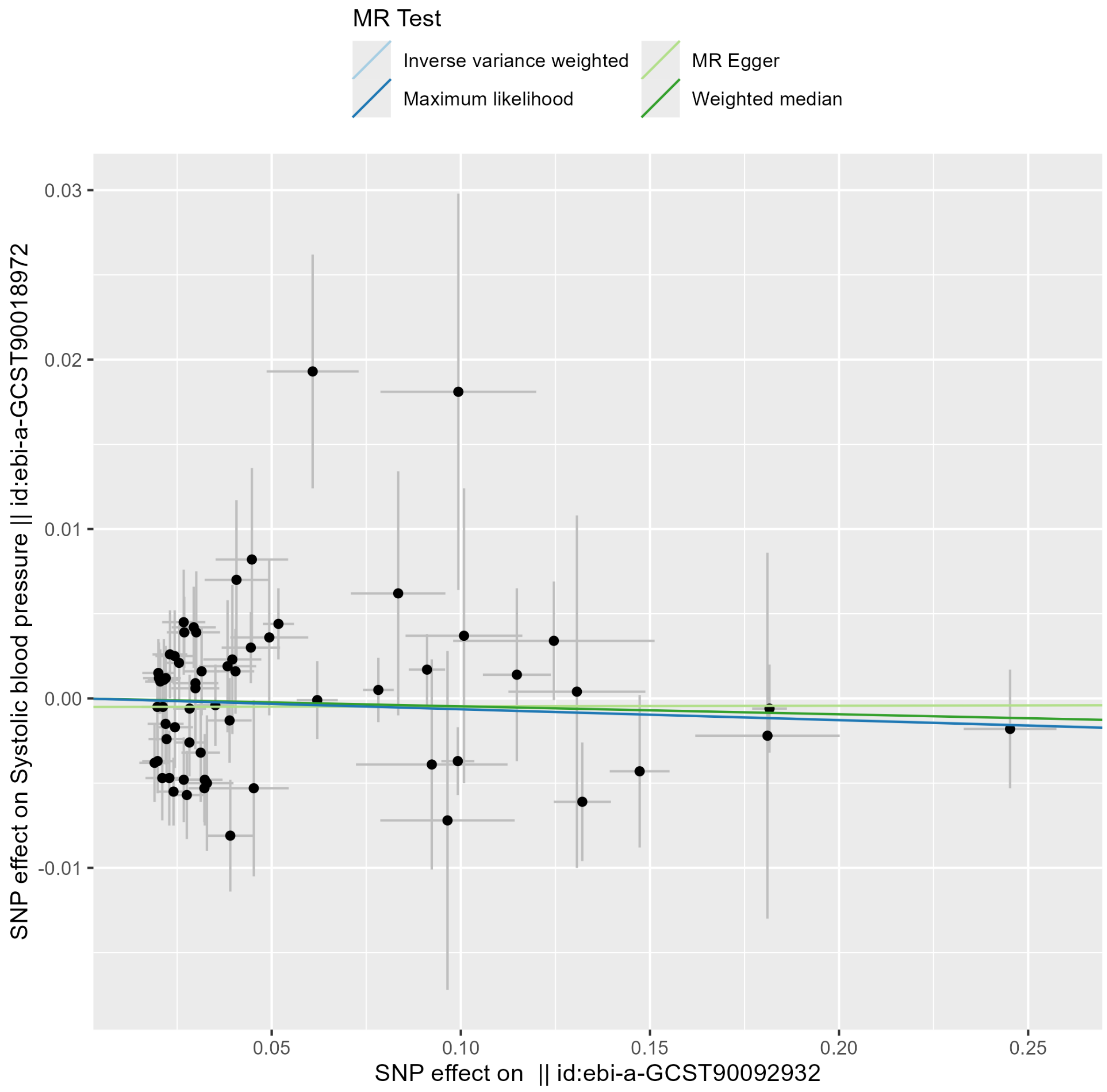

Ω-6|Ω-3-LAS scatter plot

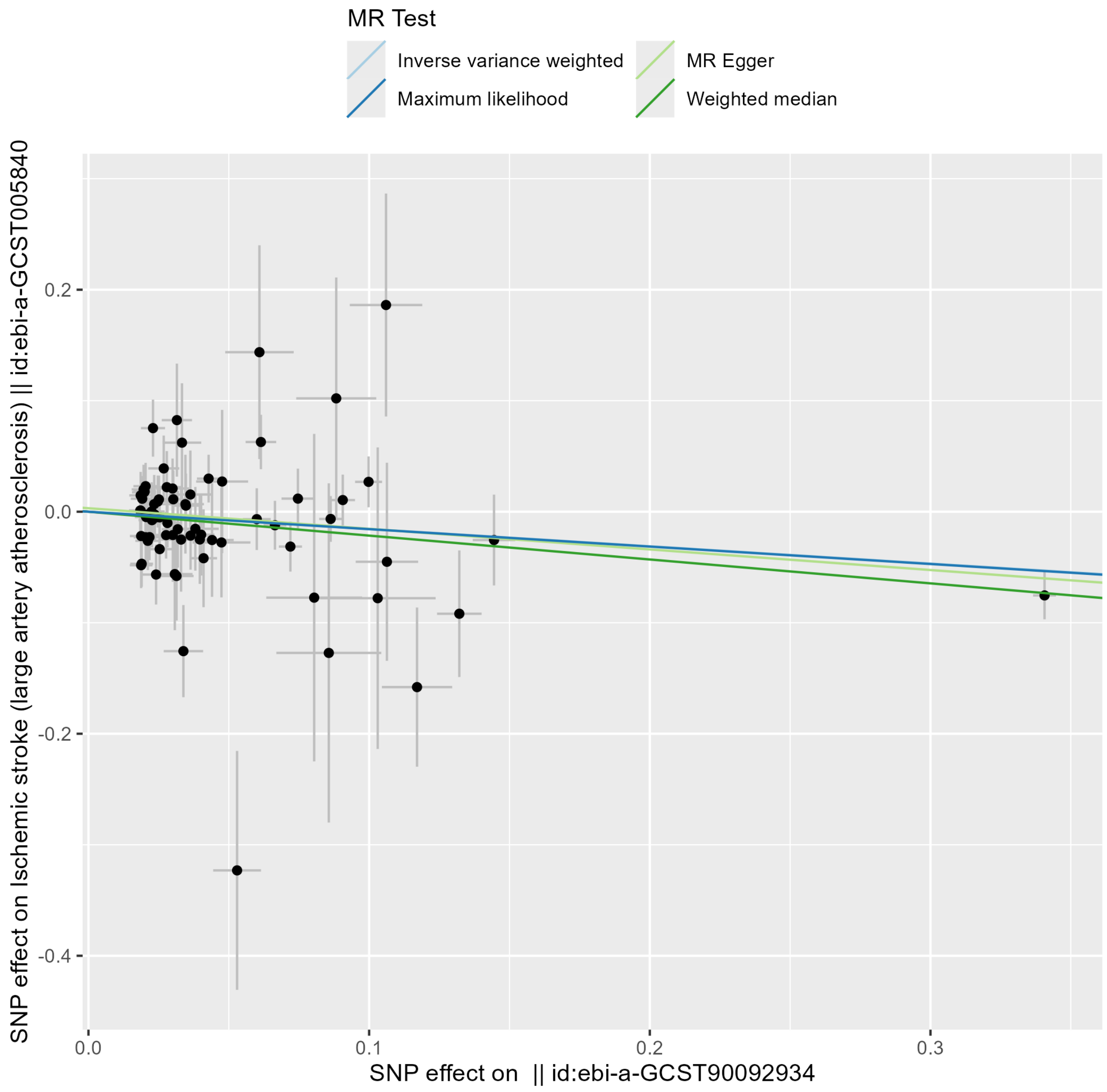

Ω-6|Ω-3-SVS scatter plot

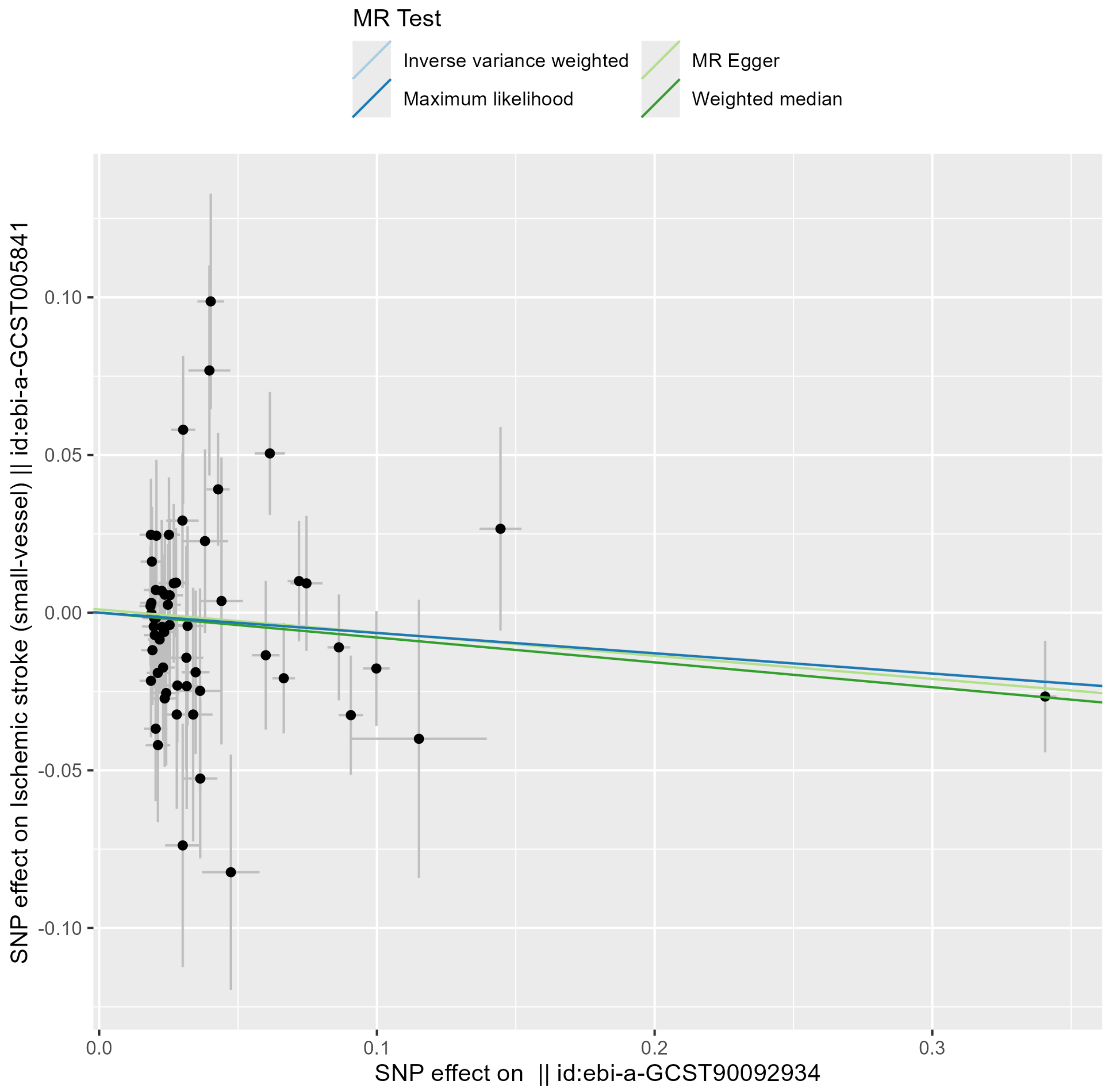

Ω-6|Ω-3-CES scatter plot

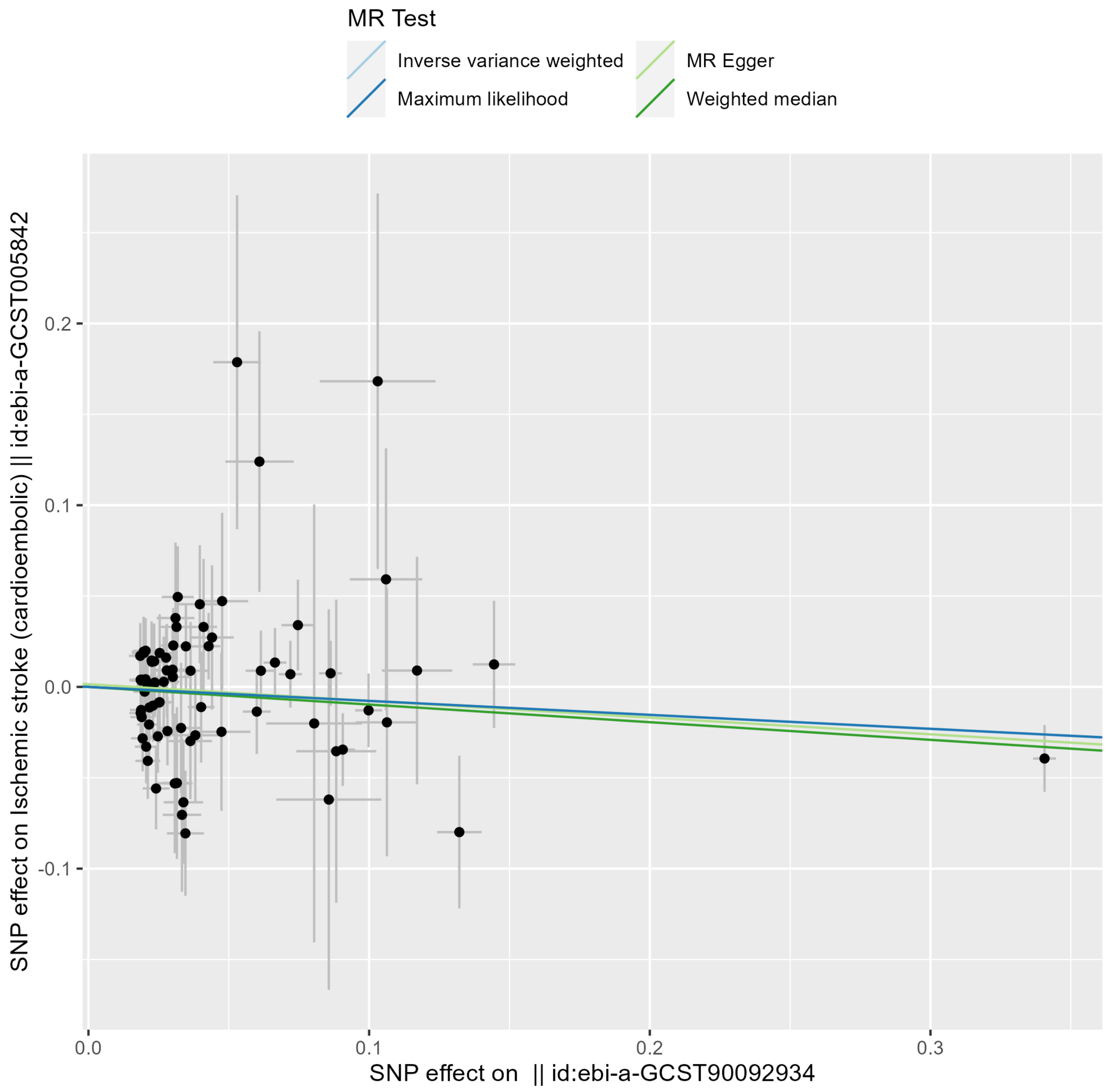

Ω-6|Ω-3-IS scatter plot

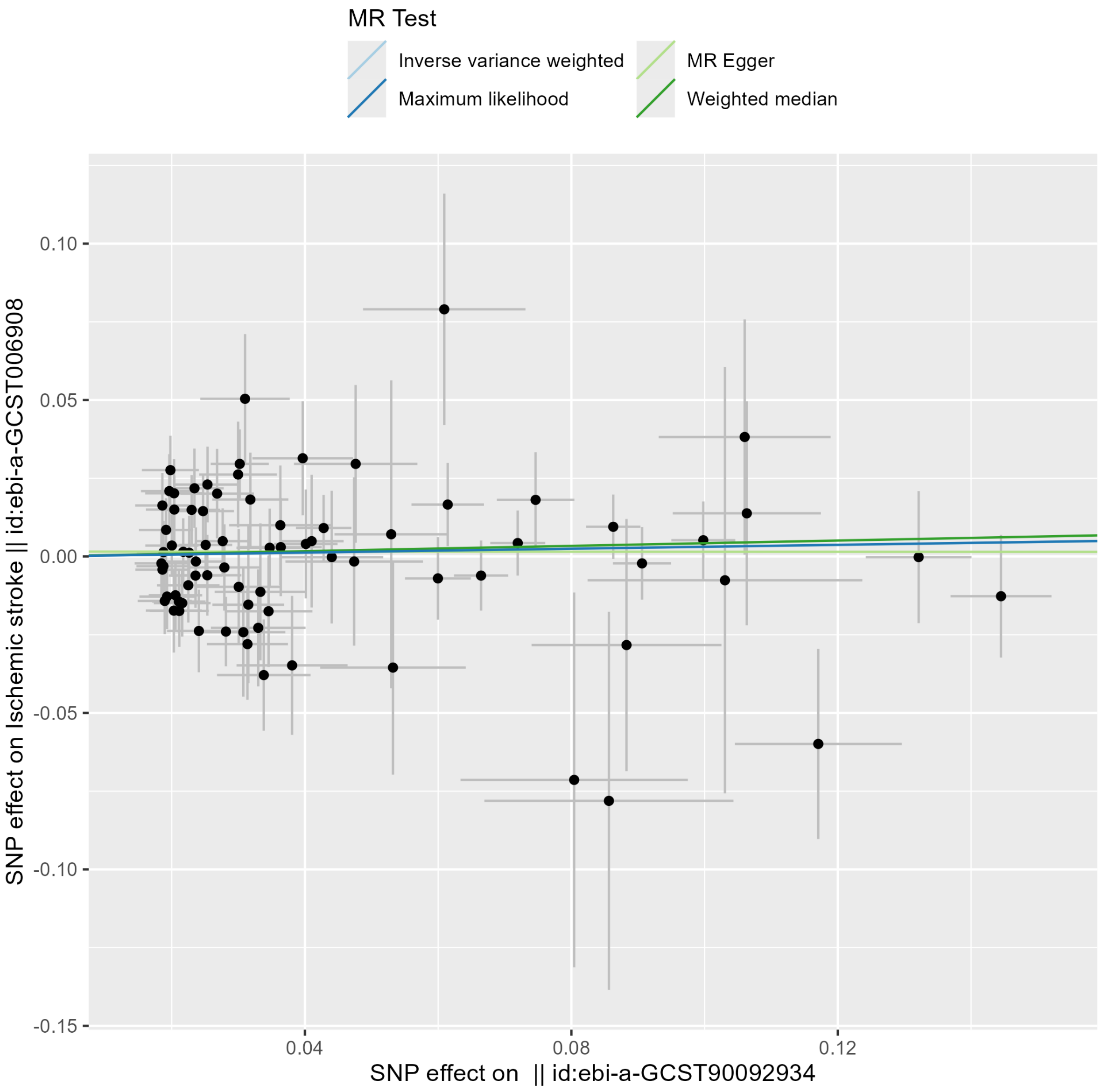

Ω-6|Ω-3-LS scatter plot

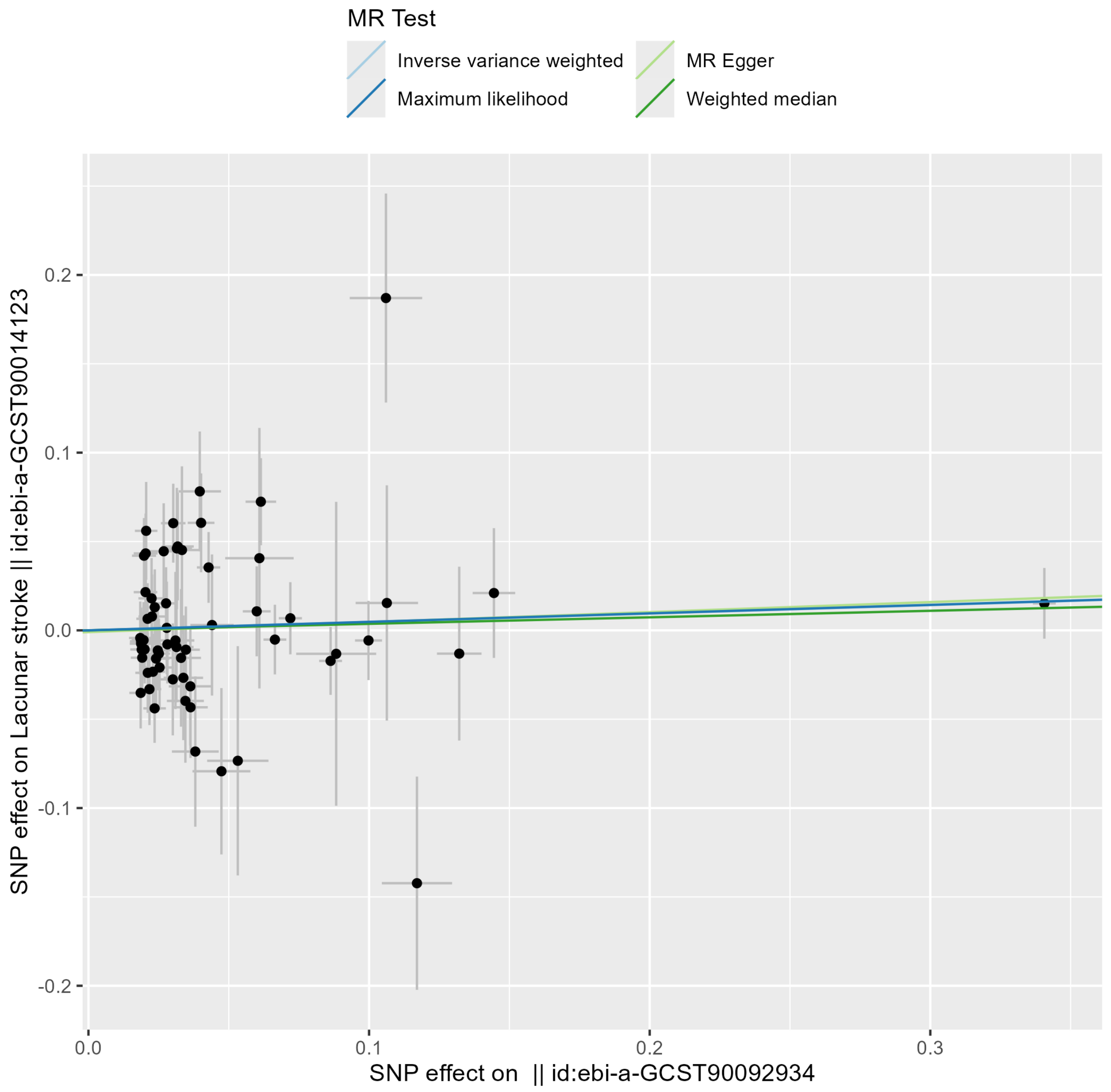

Ω-6|Ω-3-DBP scatter plot

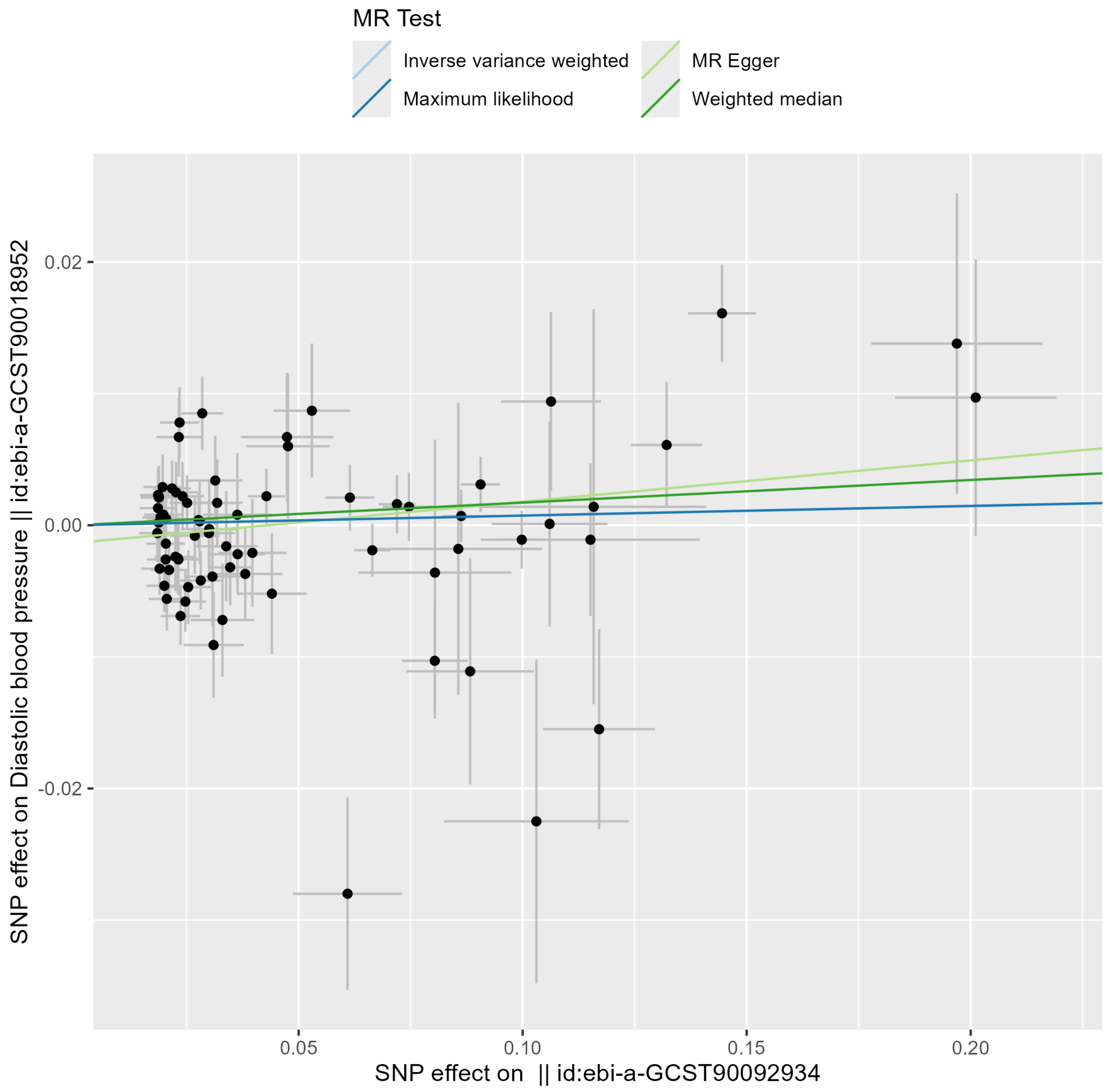

Ω-6|Ω-3-SBP scatter plot

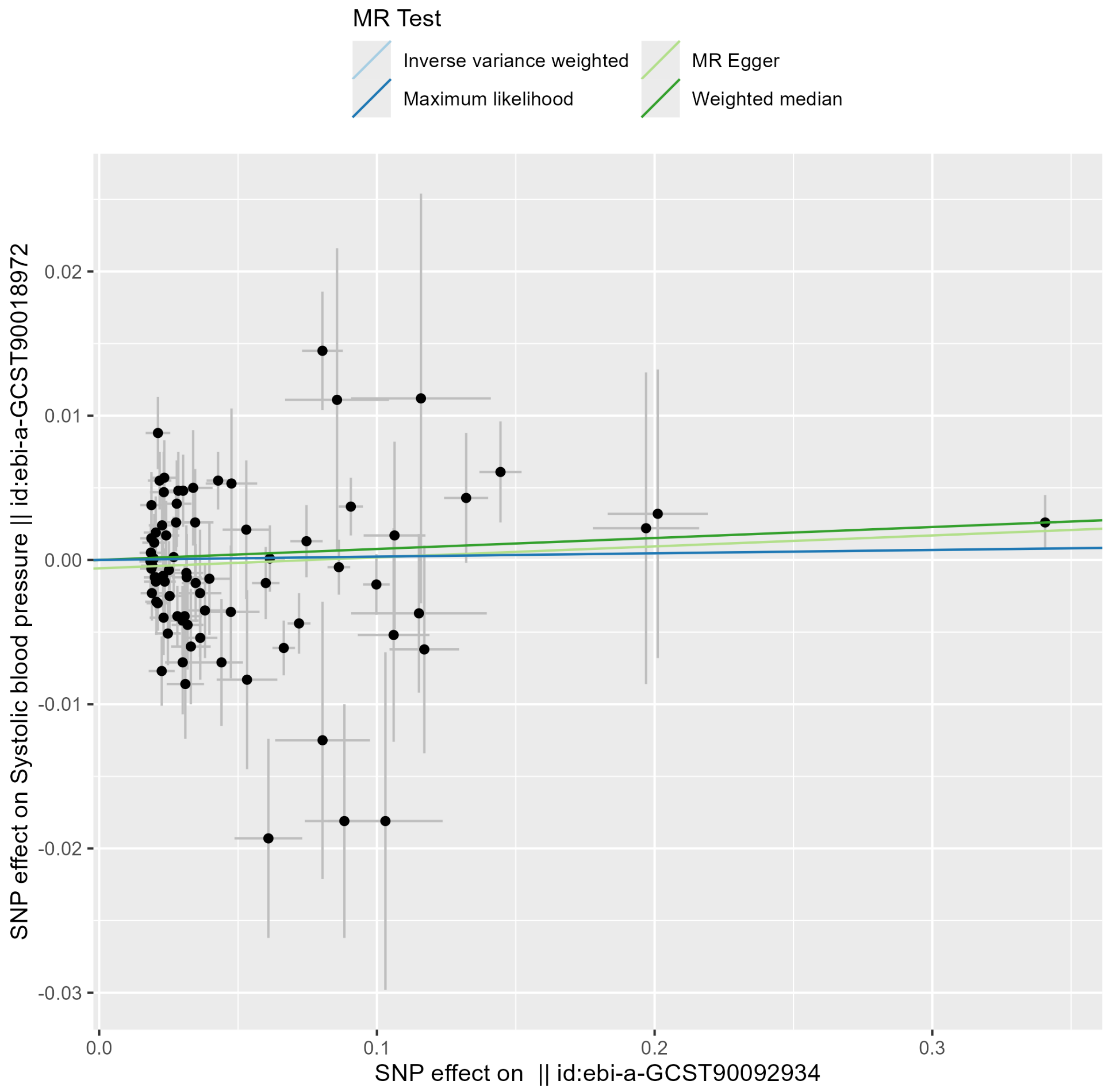

DHA-LAS forest plot

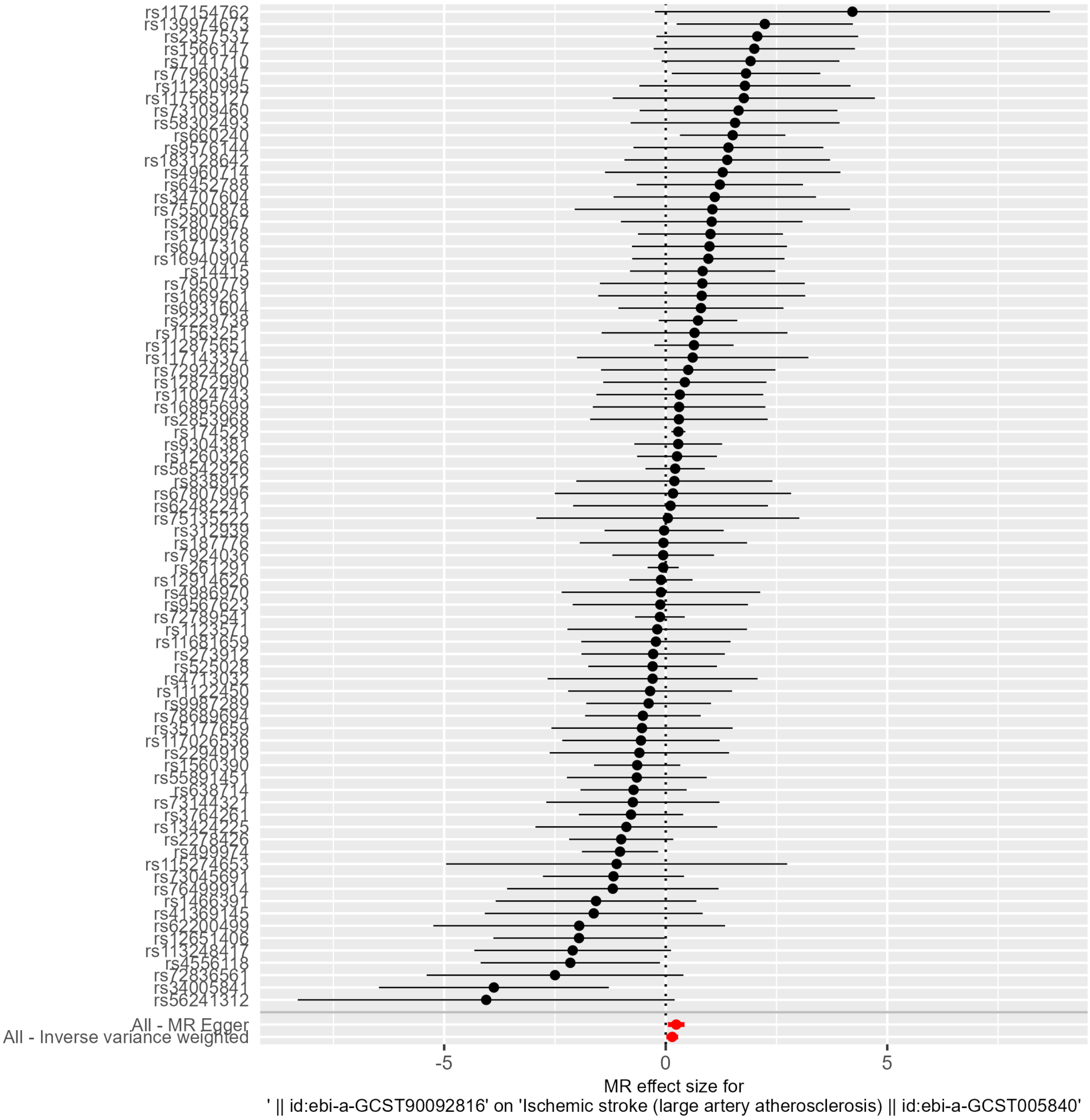

DHA-SVS forest plot

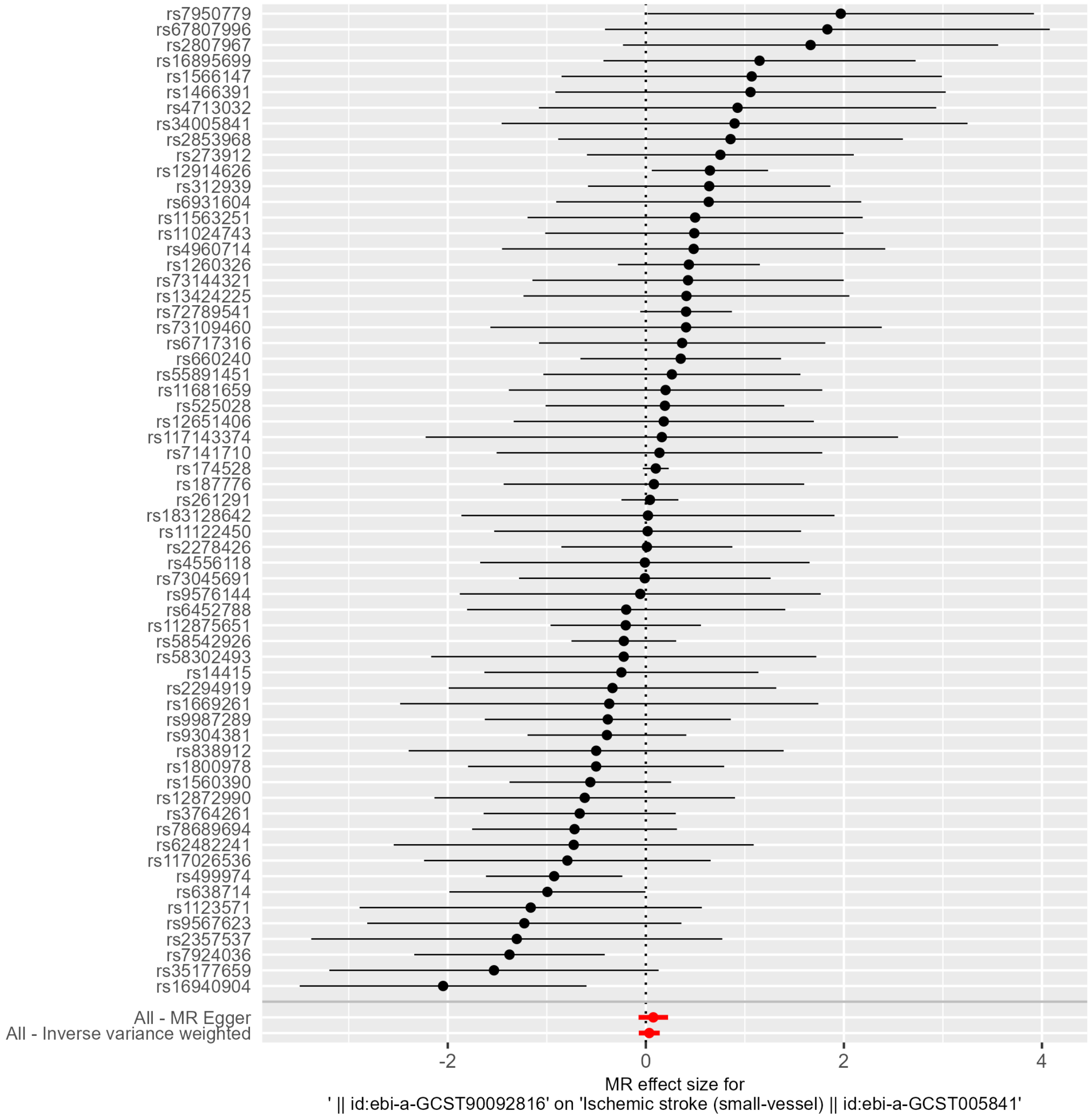

DHA-CES forest plot

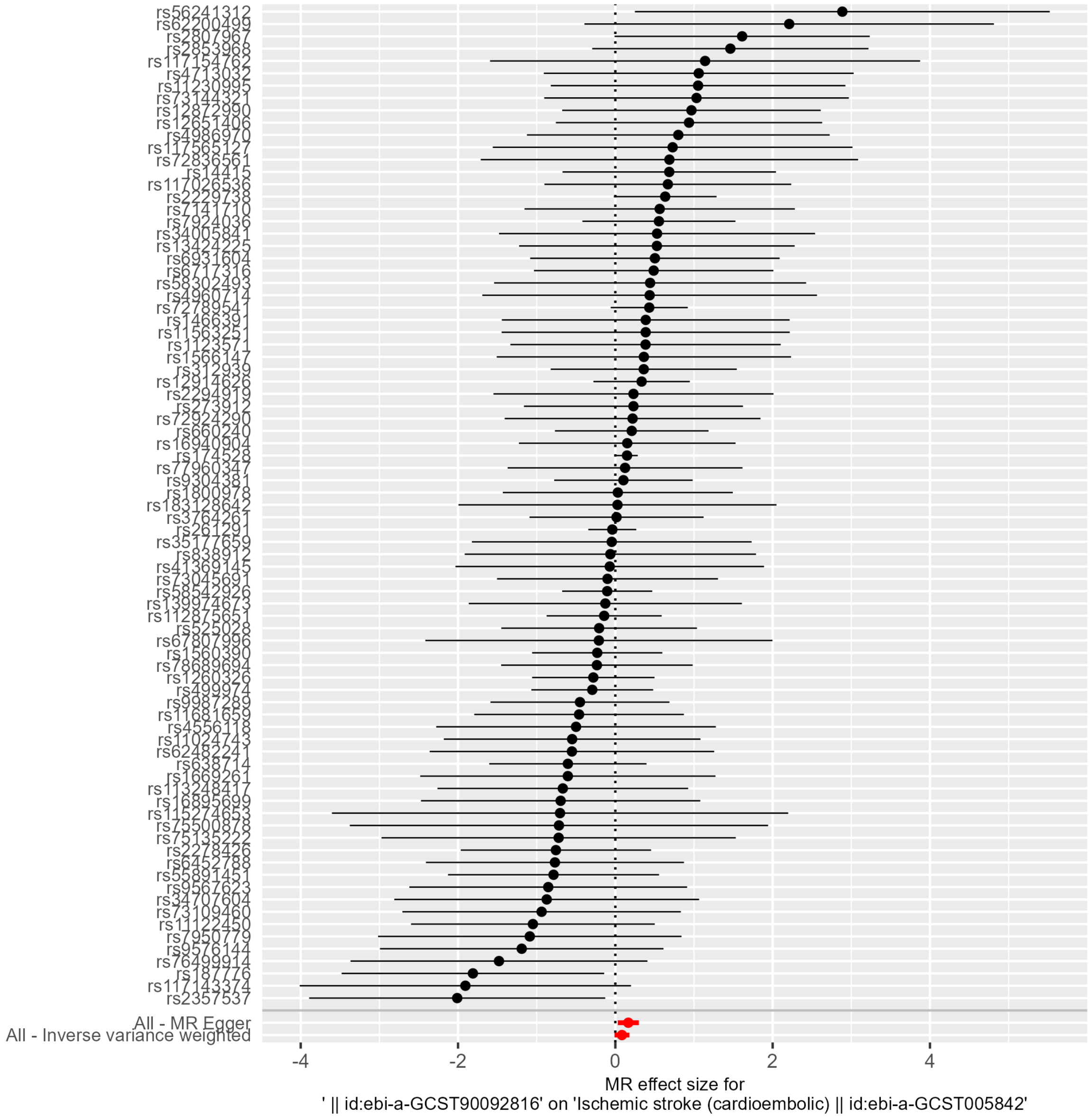

DHA-IS forest plot

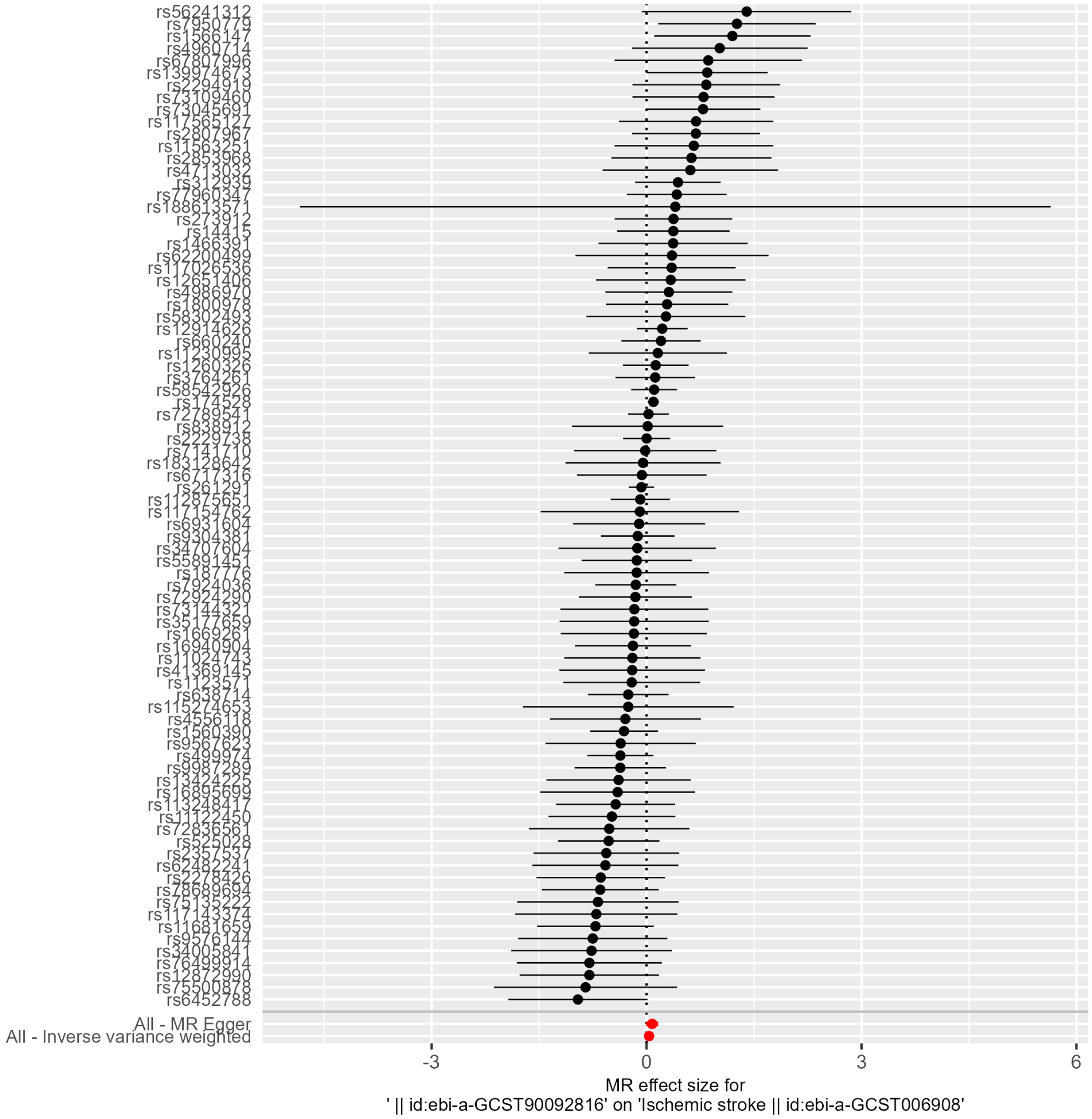

DHA-LS forest plot

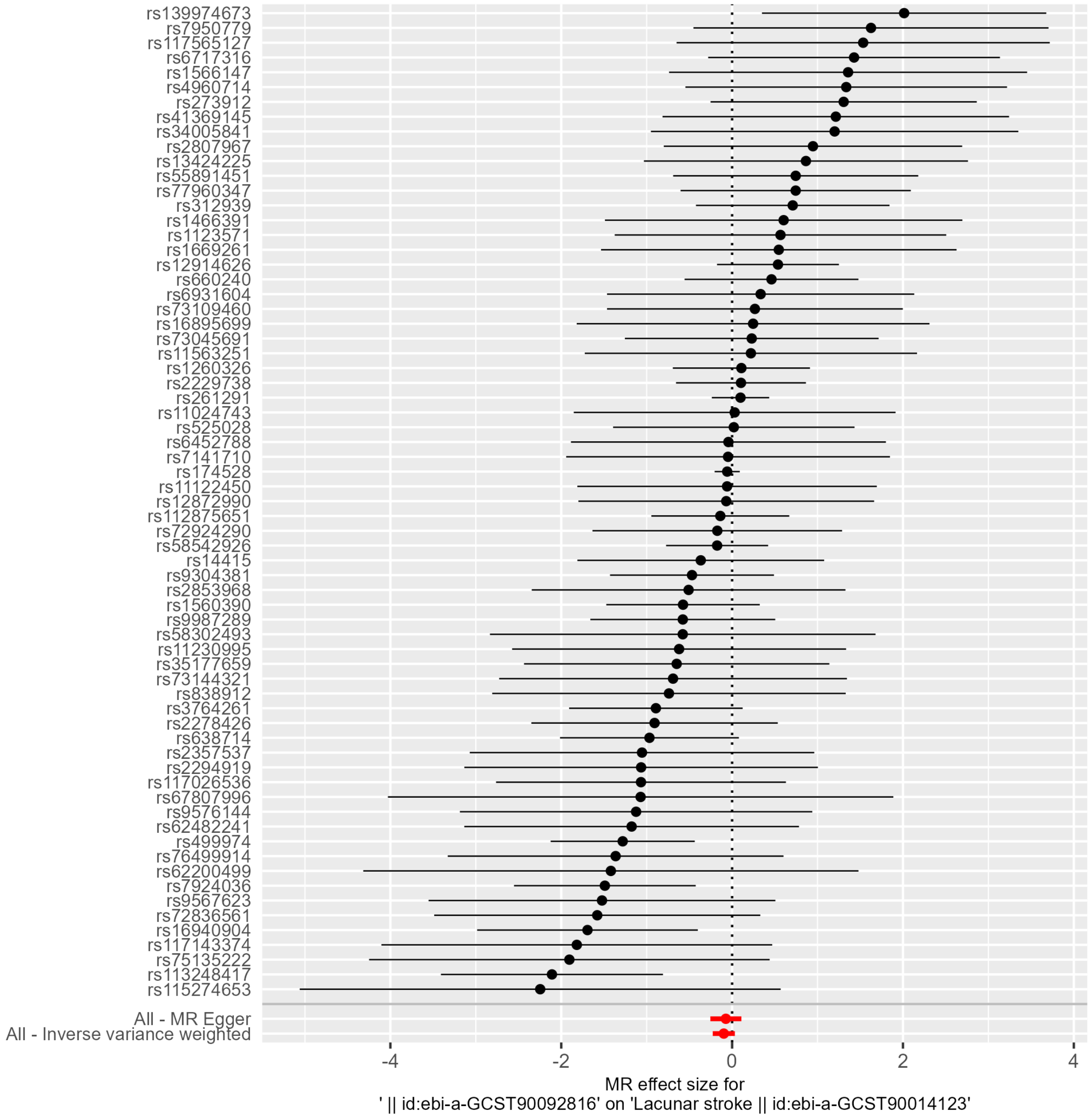

DHA-DBP forest plot

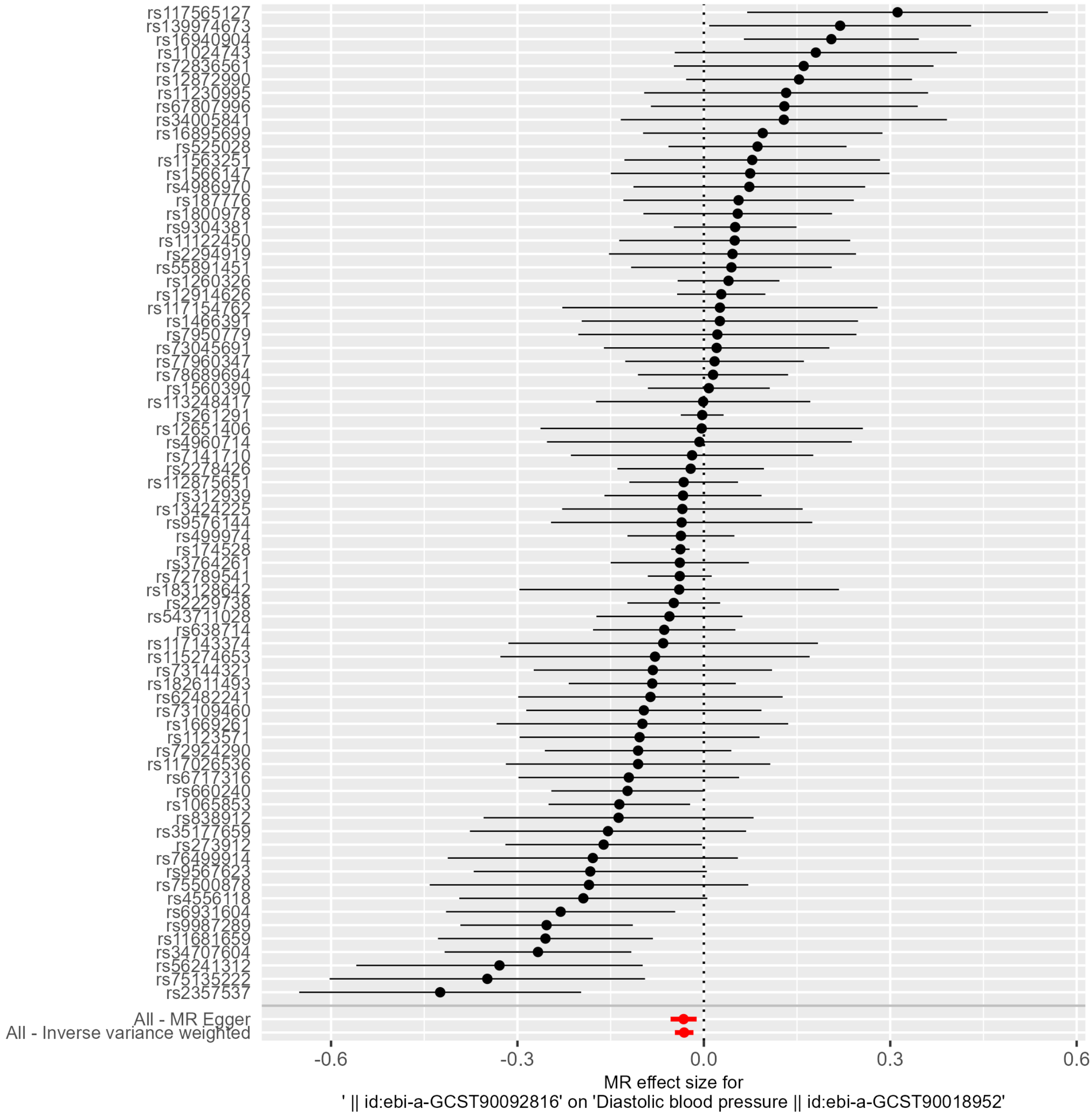

DHA-SBP forest plot

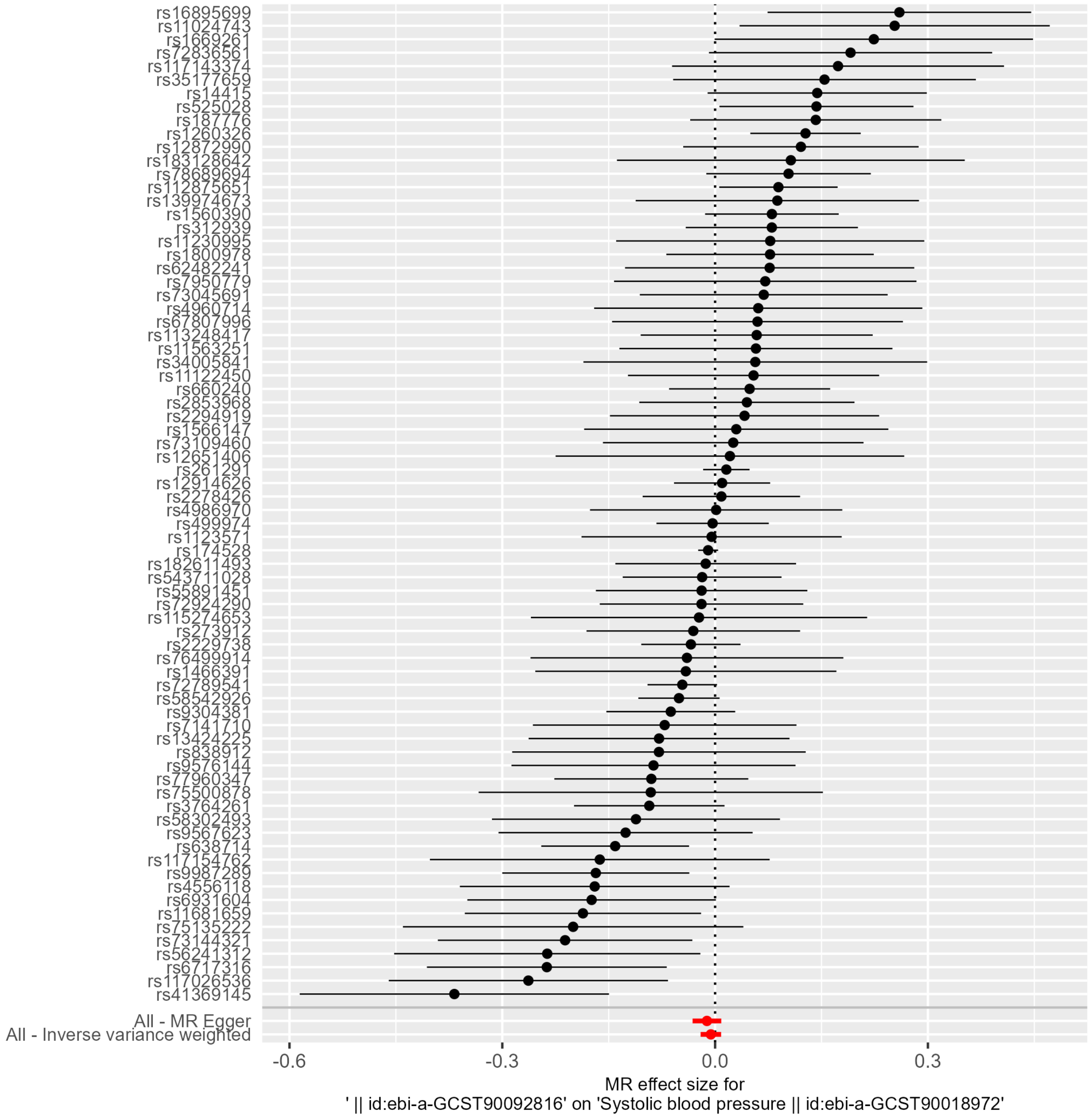

Ω-3 rate-LAS forest plot

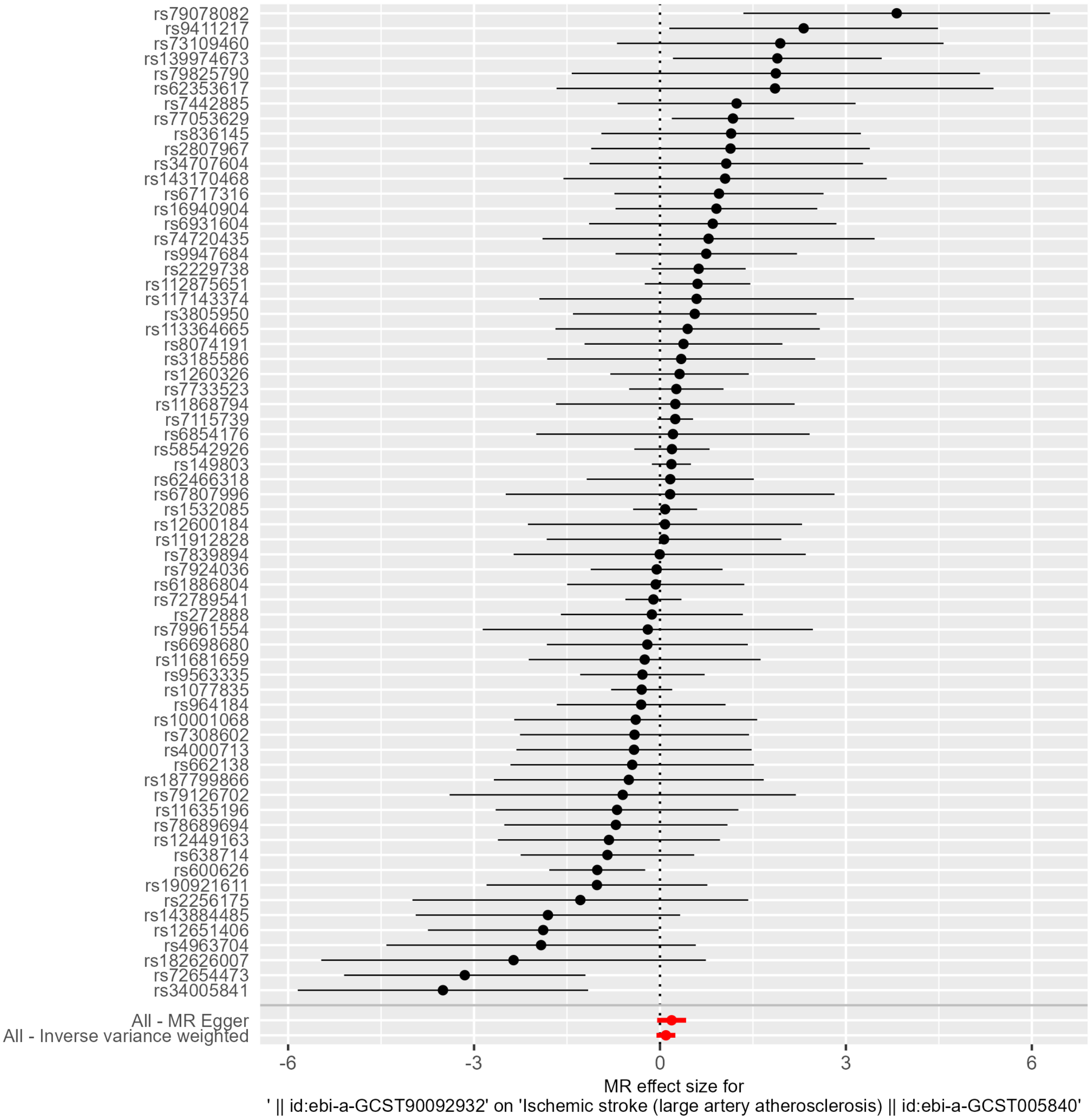

Ω-3 rate-SVS forest plot

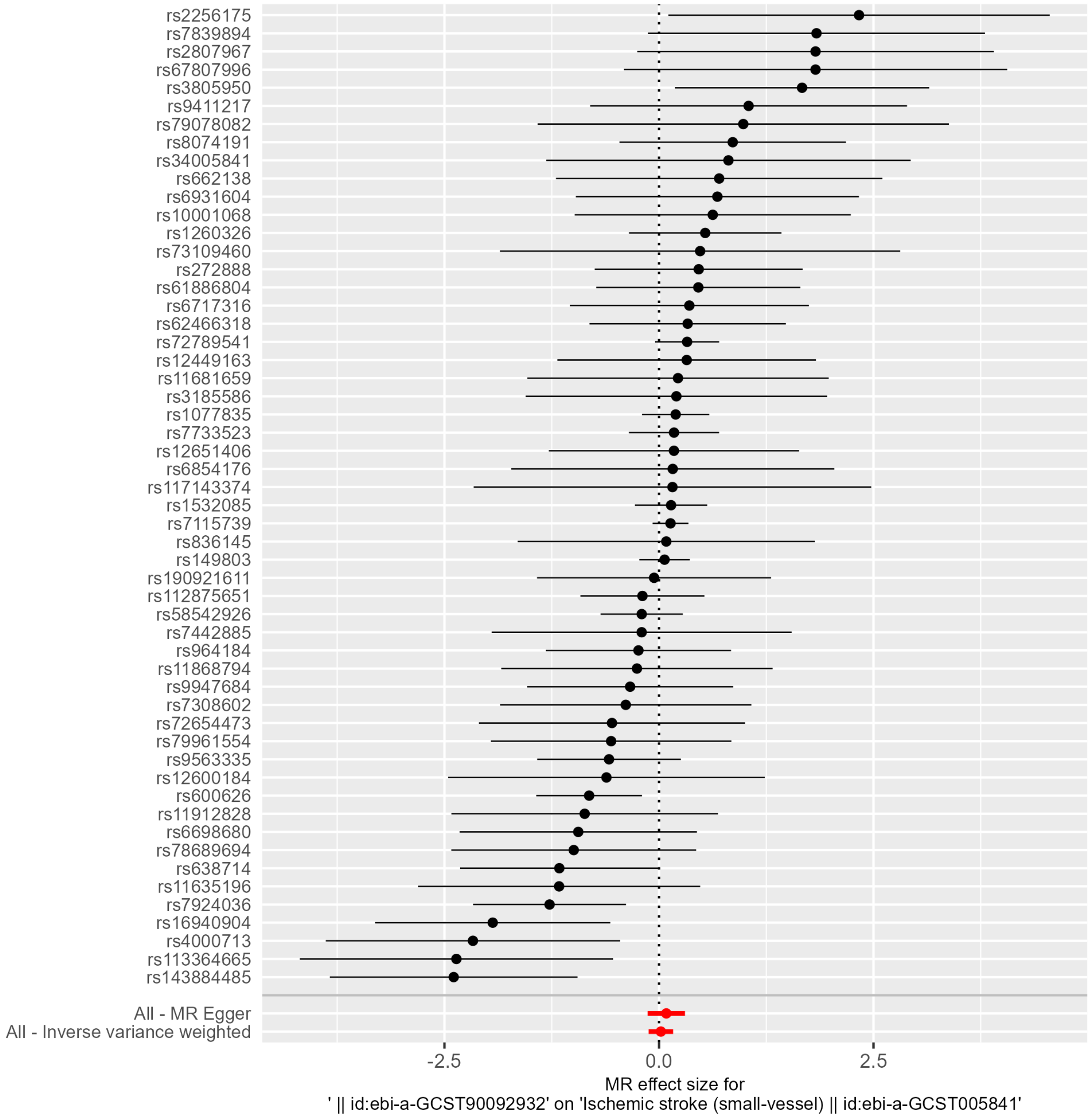

Ω-3 rate-CES forest plot

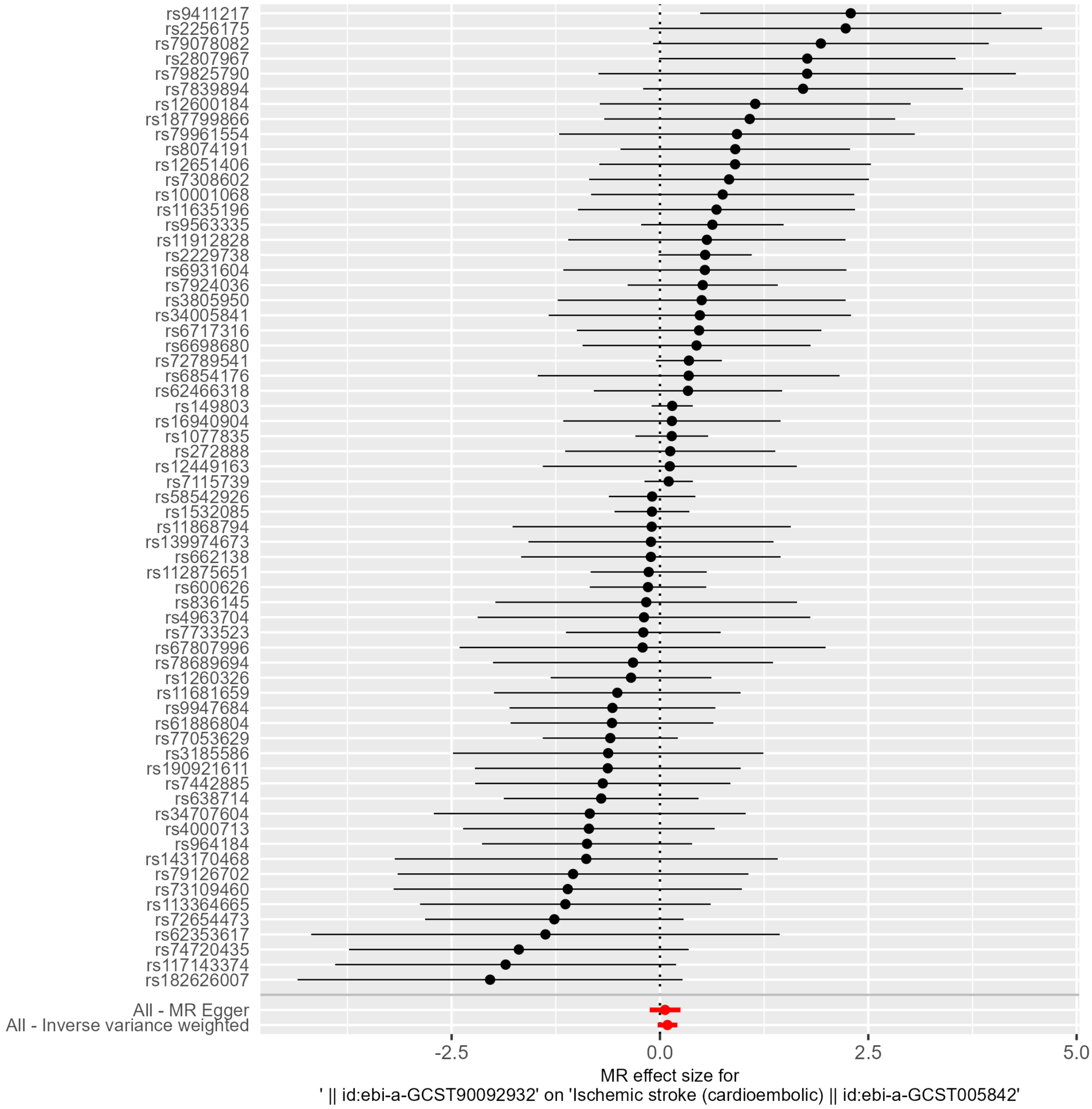

Ω-3 rate-IS forest plot

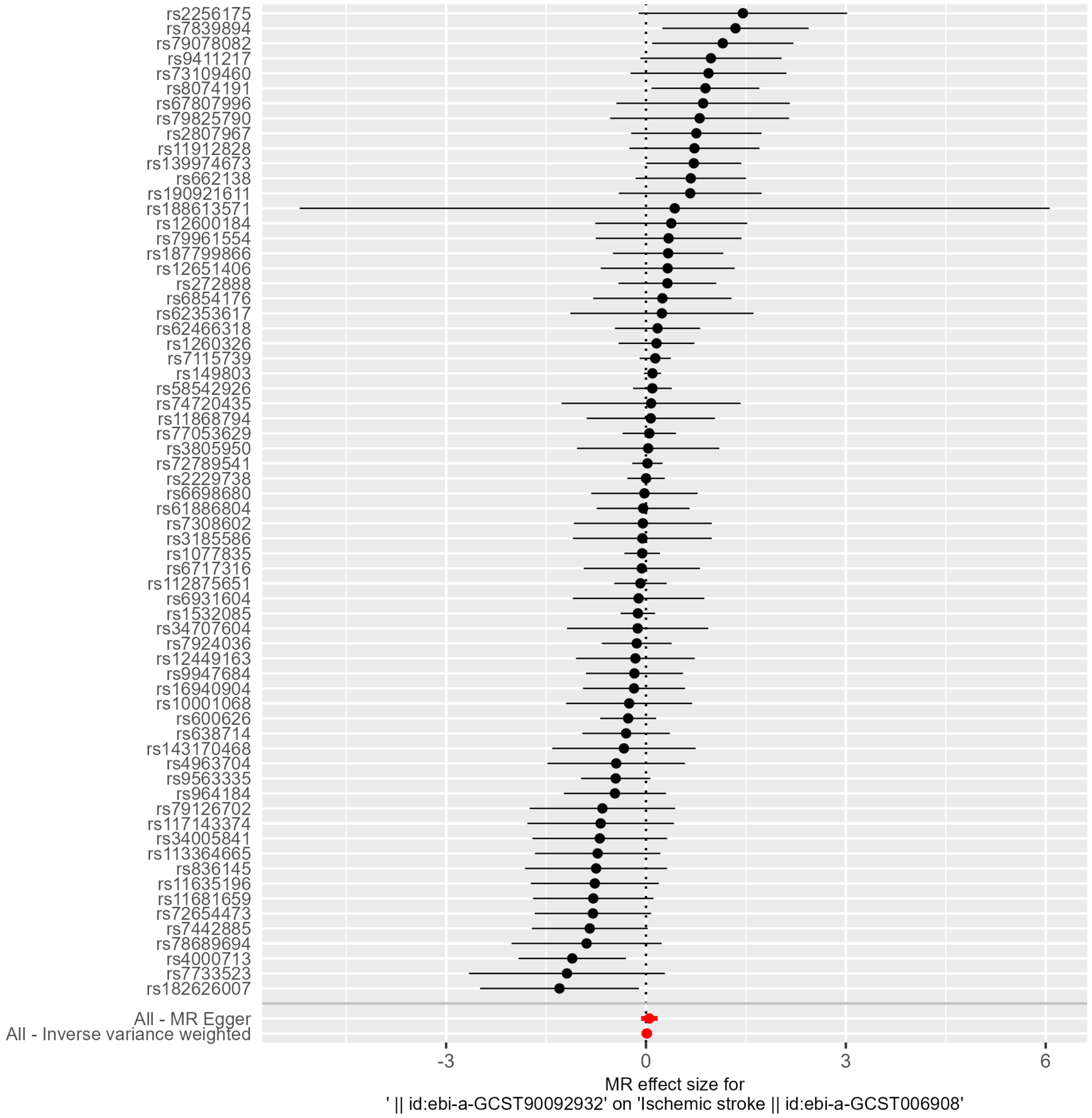

Ω-3 rate-LS forest plot

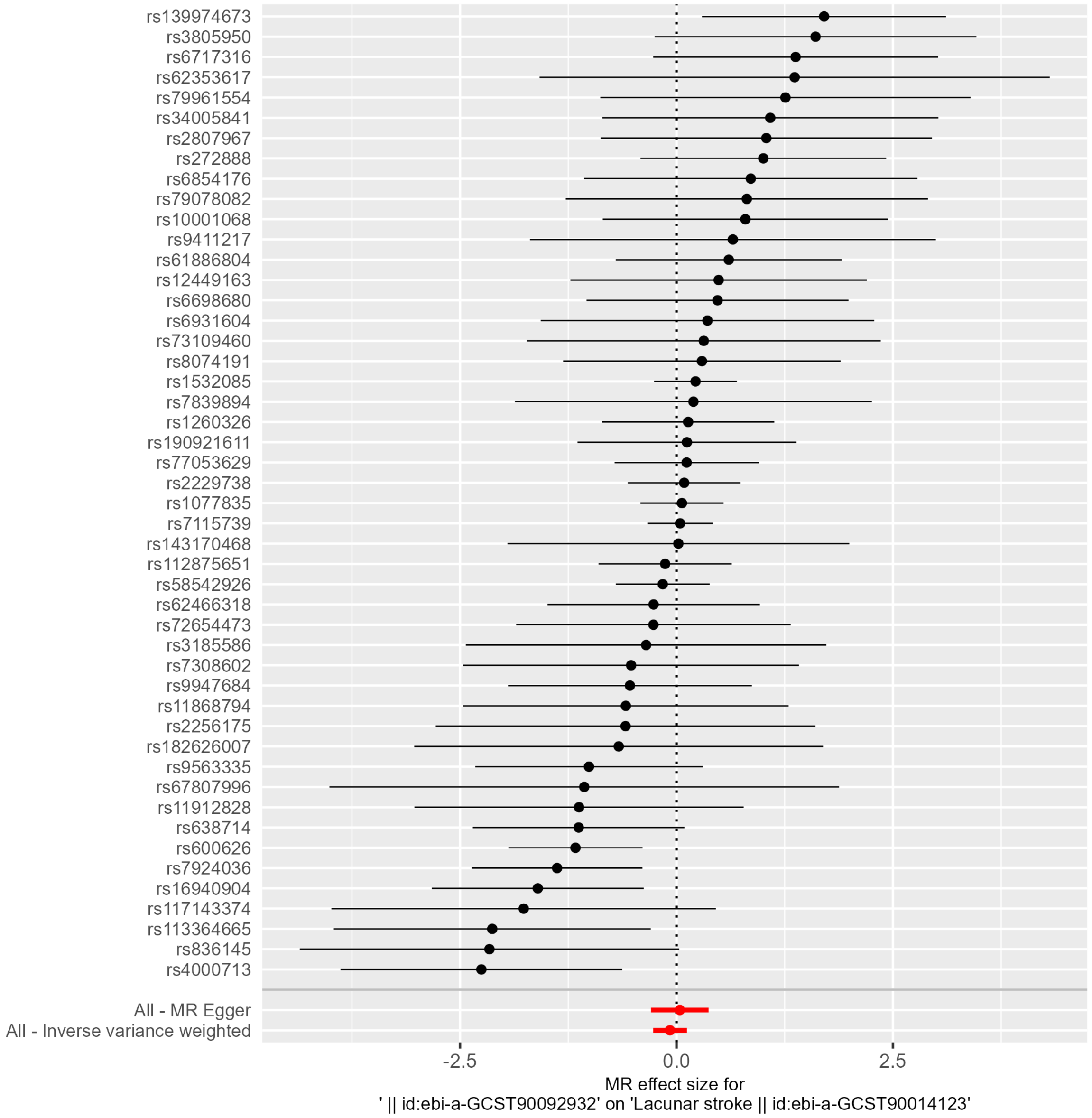

Ω-3 rate-DBP forest plot

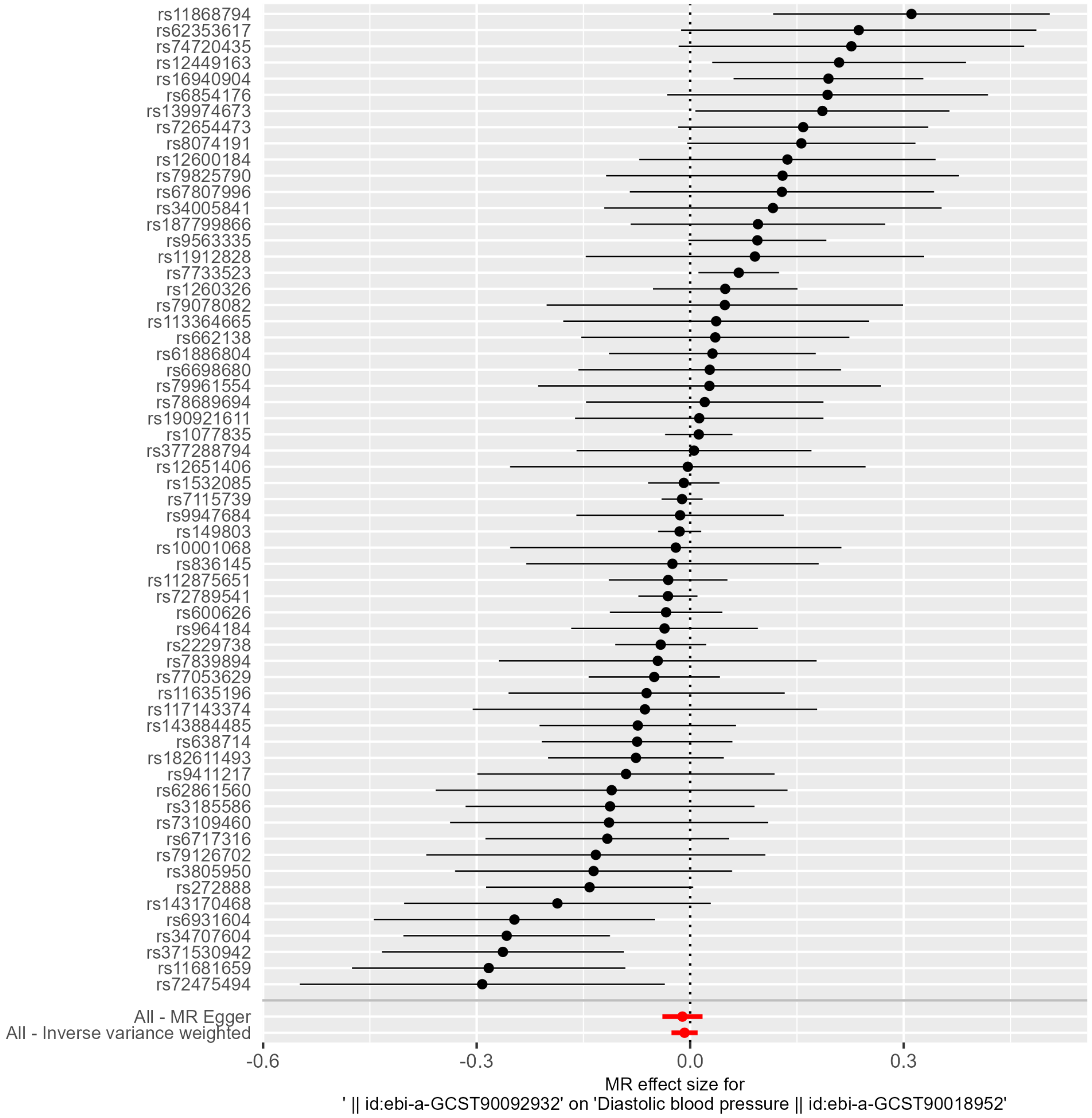

Ω-3 rate-SBP forest plot

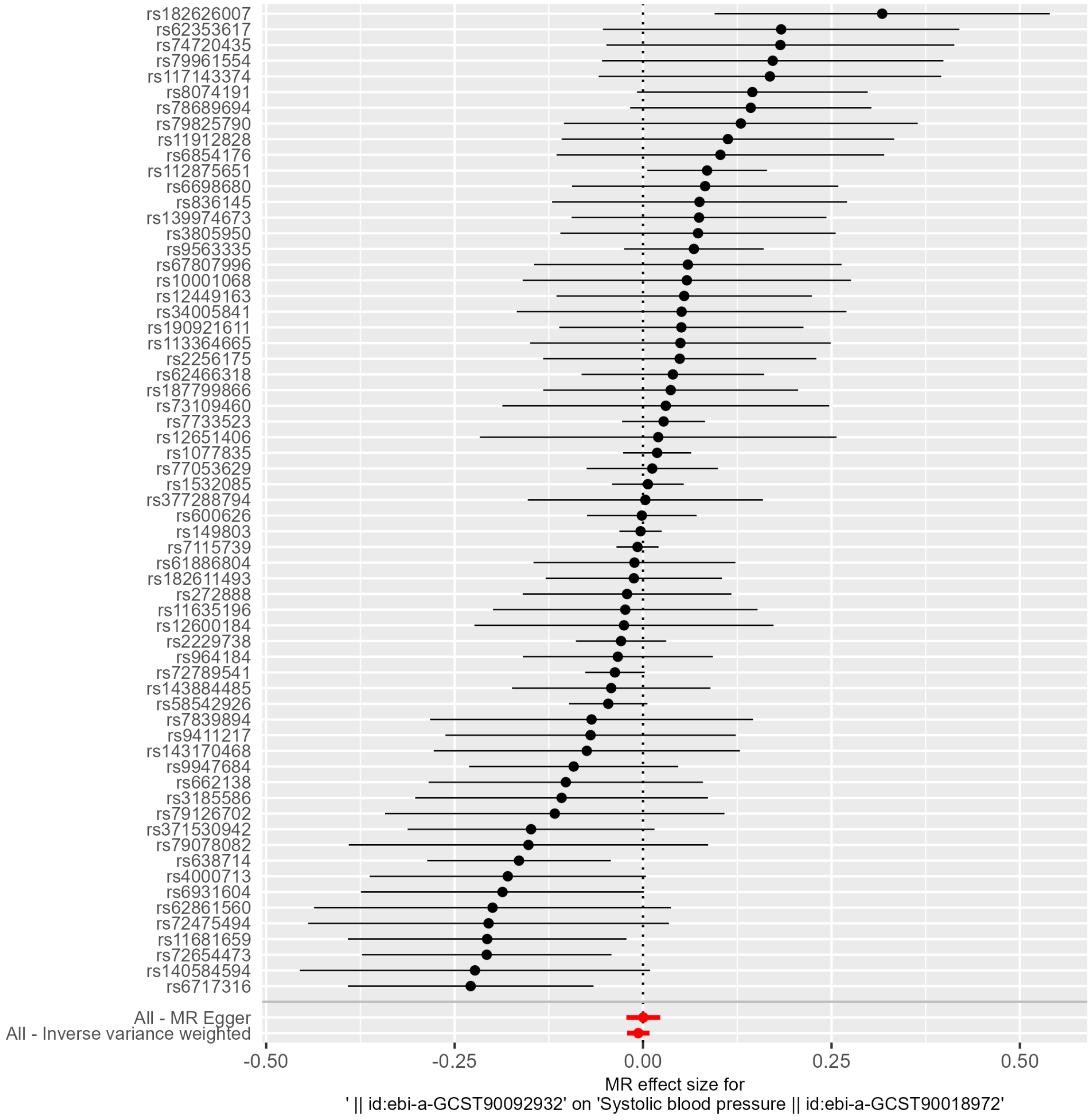

Ω-6|Ω-3-LAS forest plot

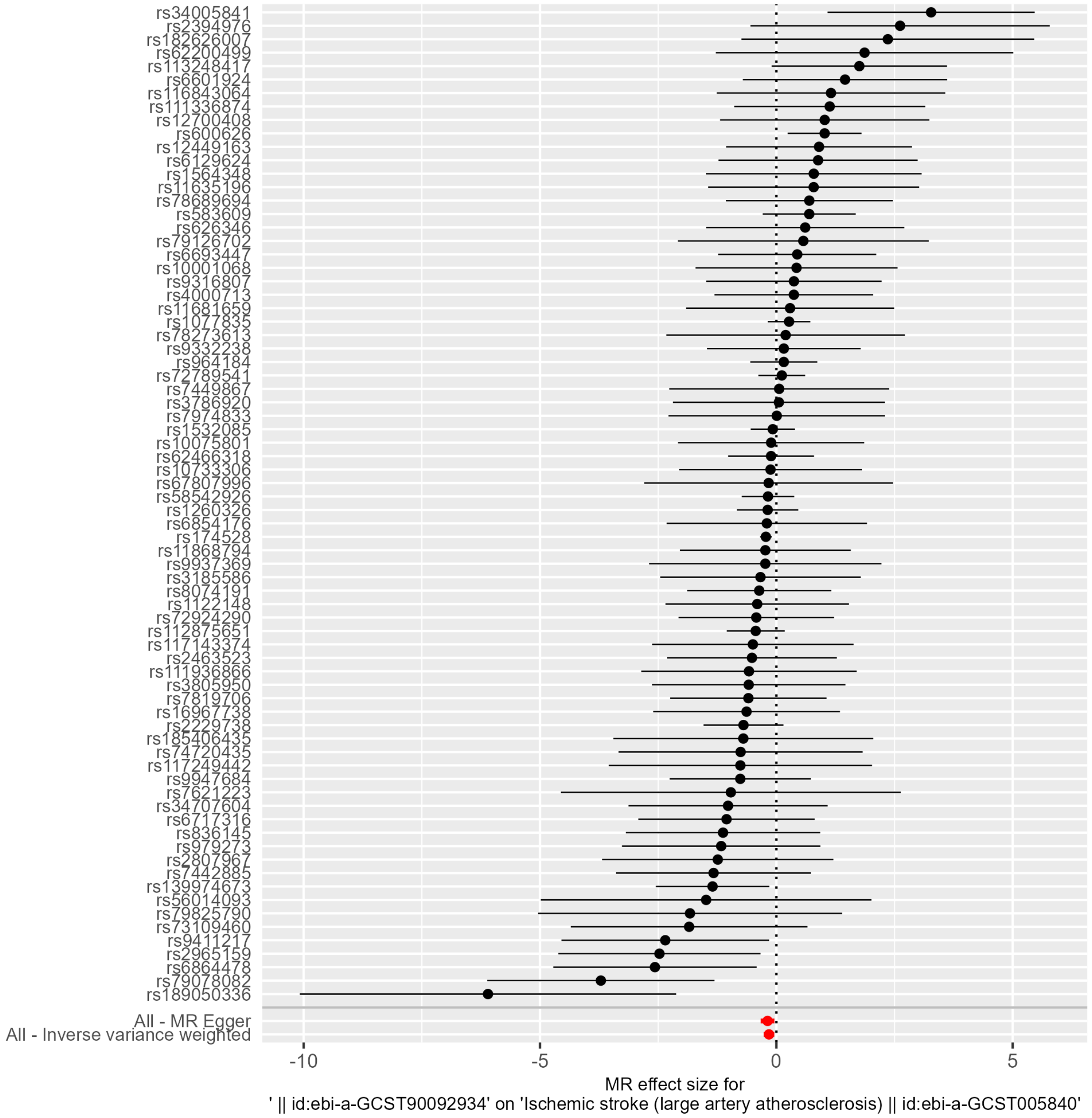

Ω-6|Ω-3-SVS forest plot

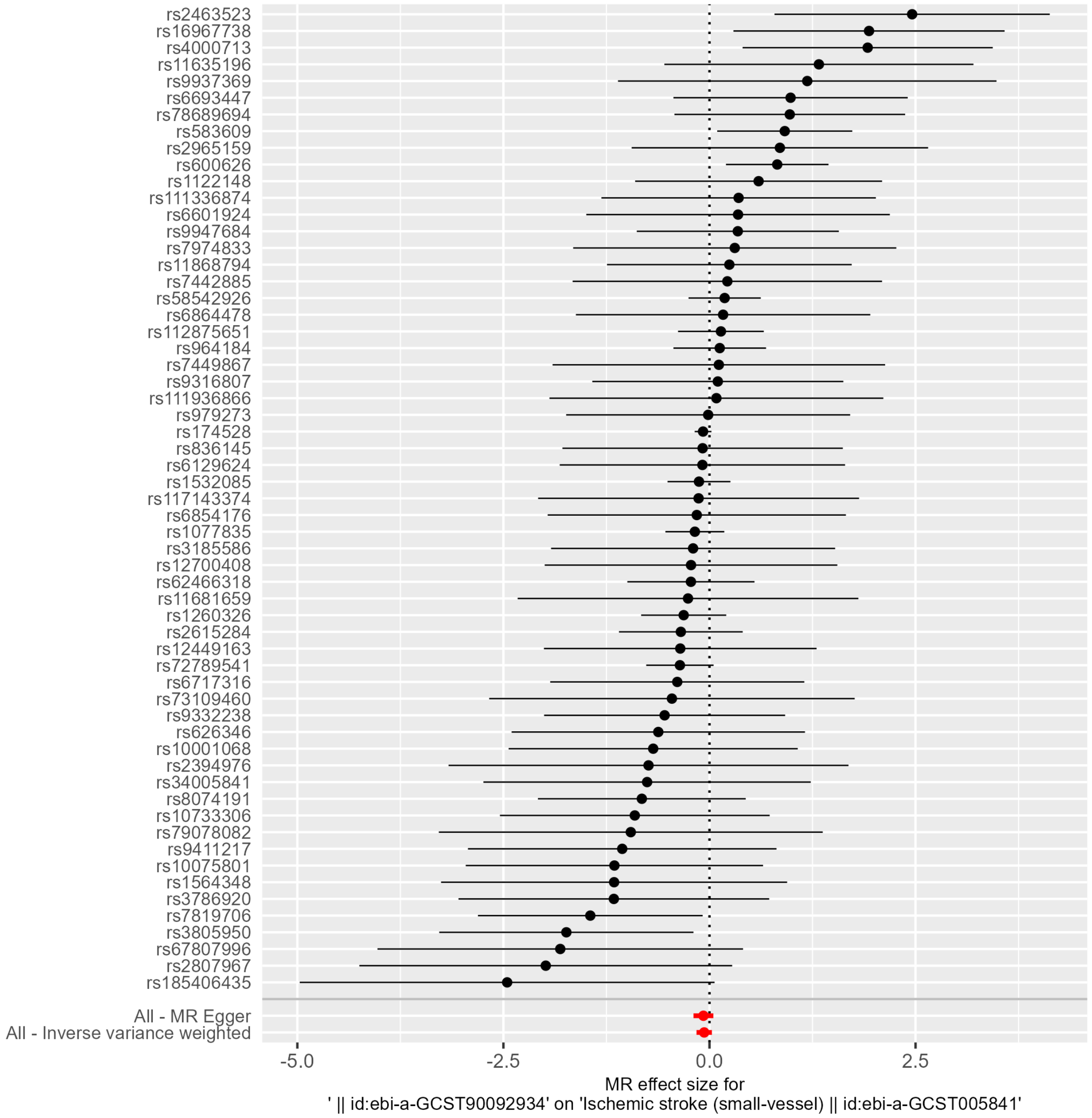

Ω-6|Ω-3-CES forest plot

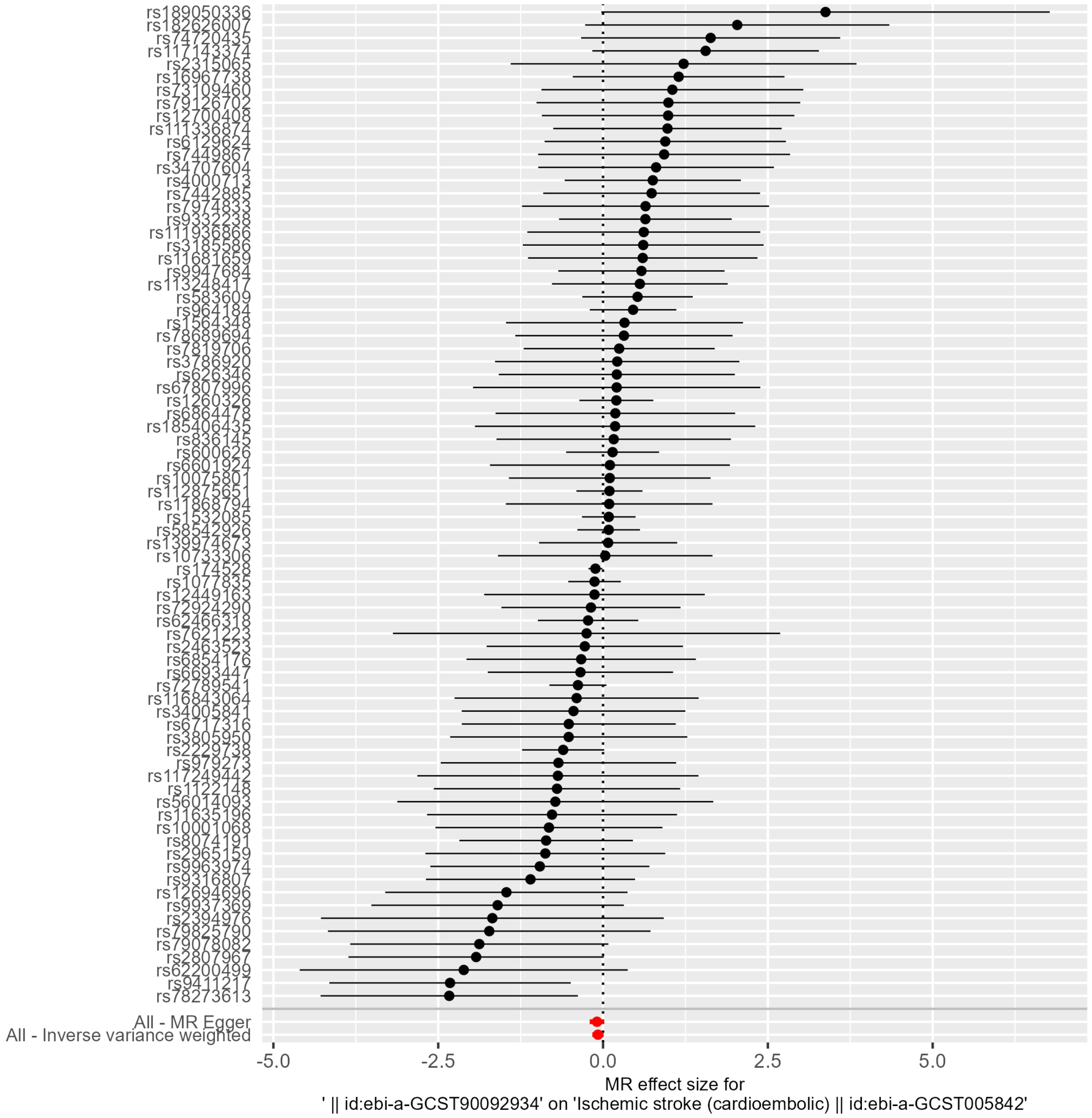

Ω-6|Ω-3-IS forest plot

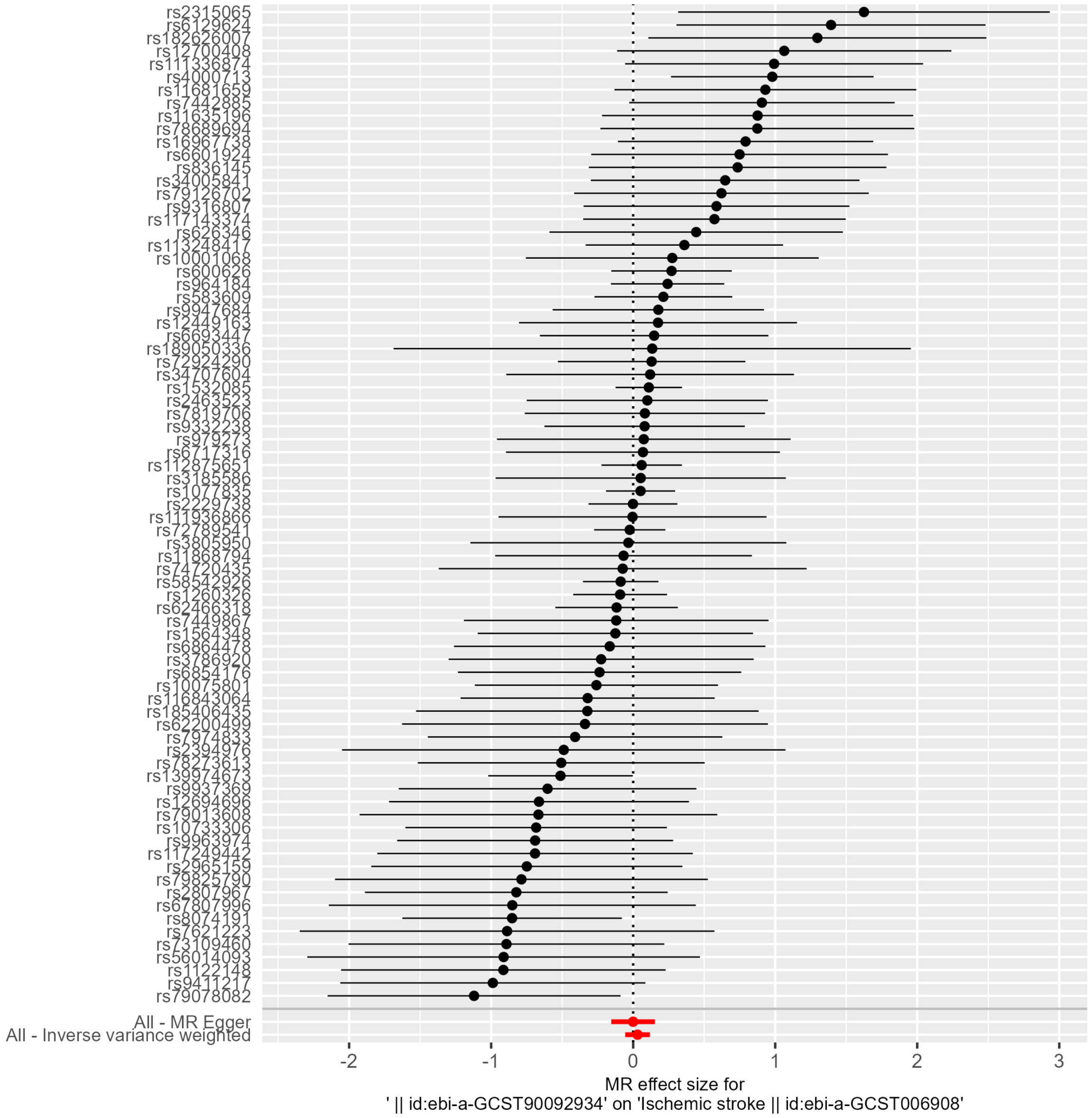

Ω-6|Ω-3-LS forest plot

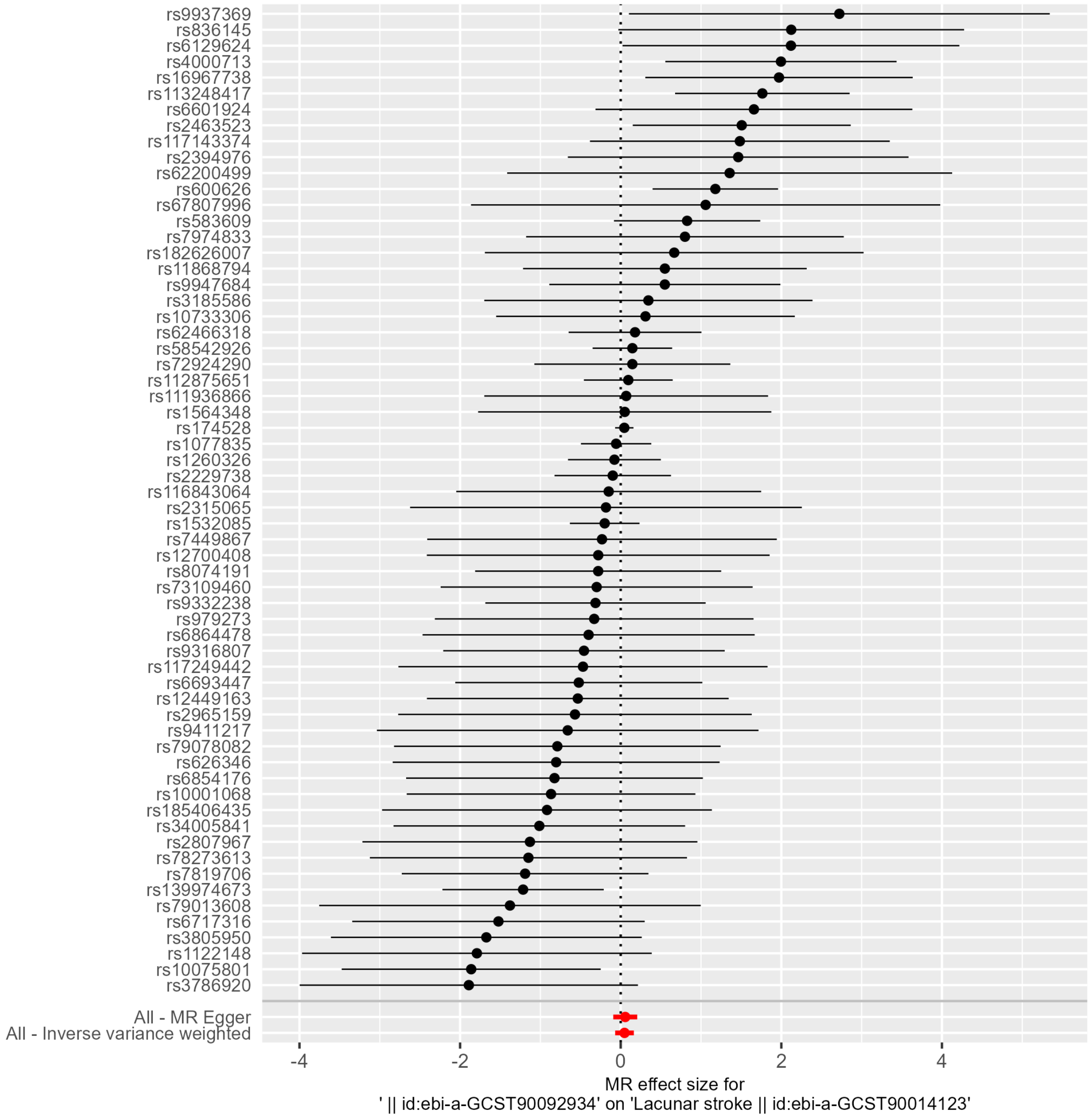

Ω-6|Ω-3-DBP forest plot

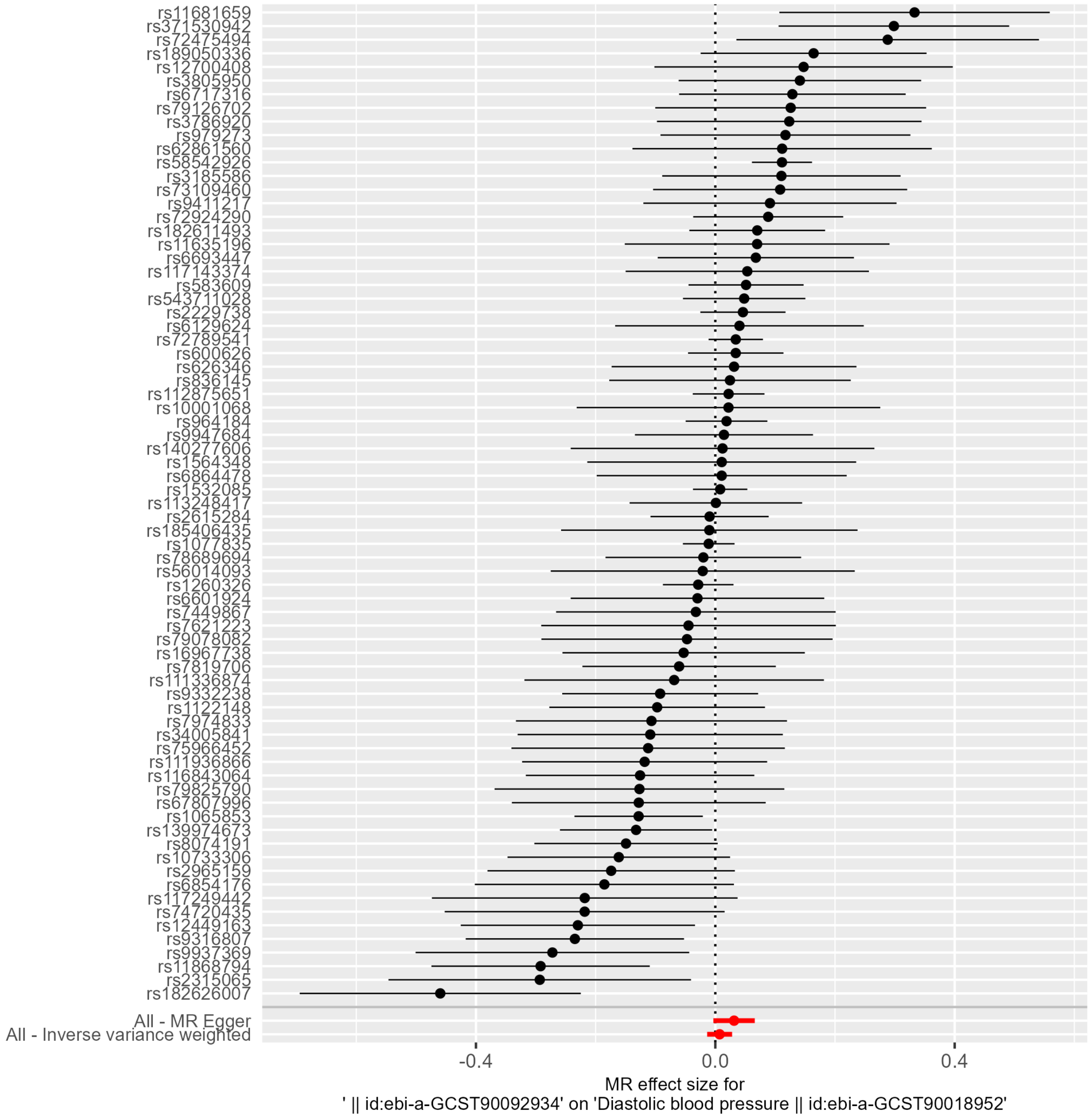

Ω-6|Ω-3-SBP forest plot

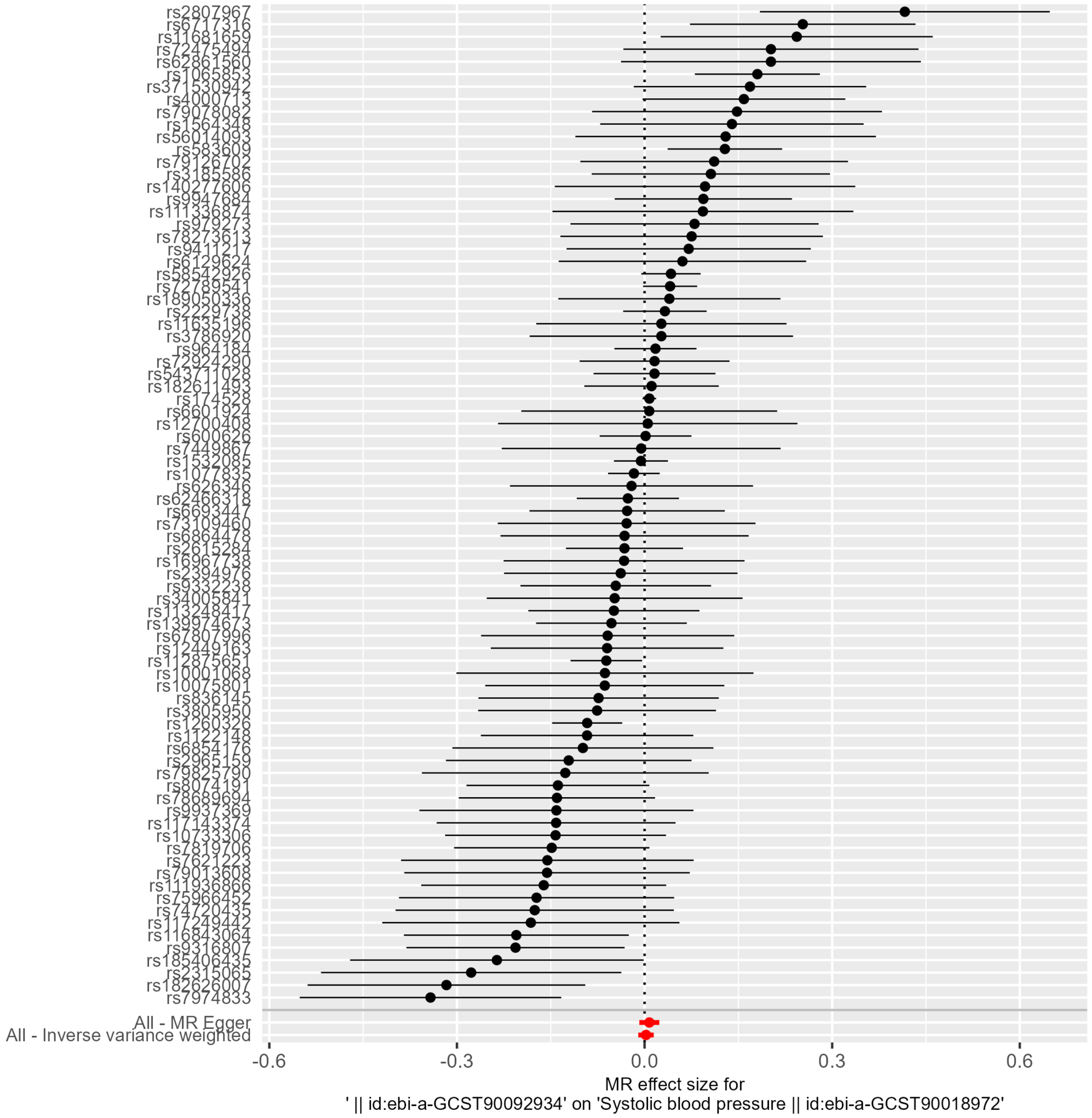

DHA-LAS funnel plot

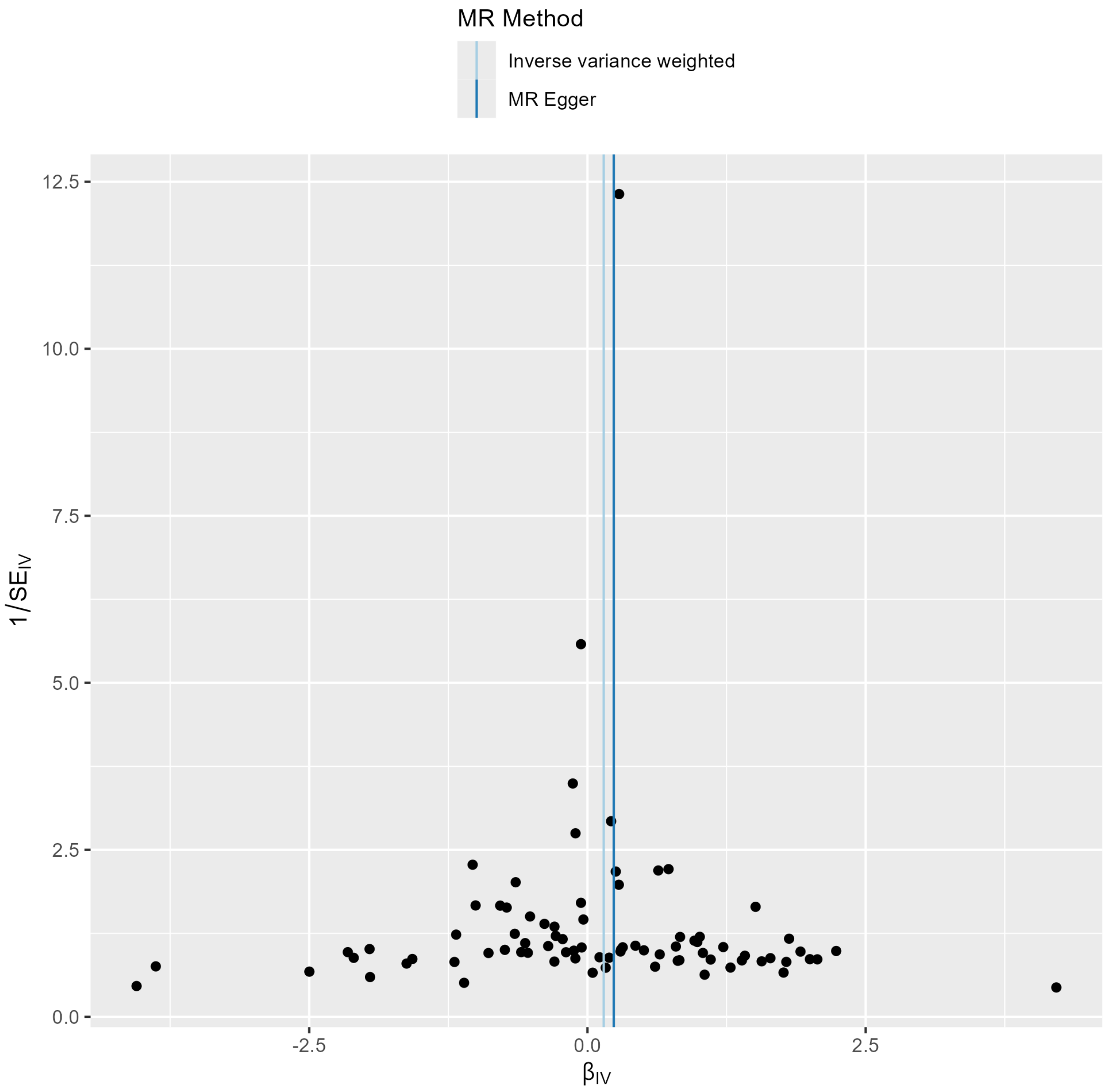

DHA-SVS funnel plot

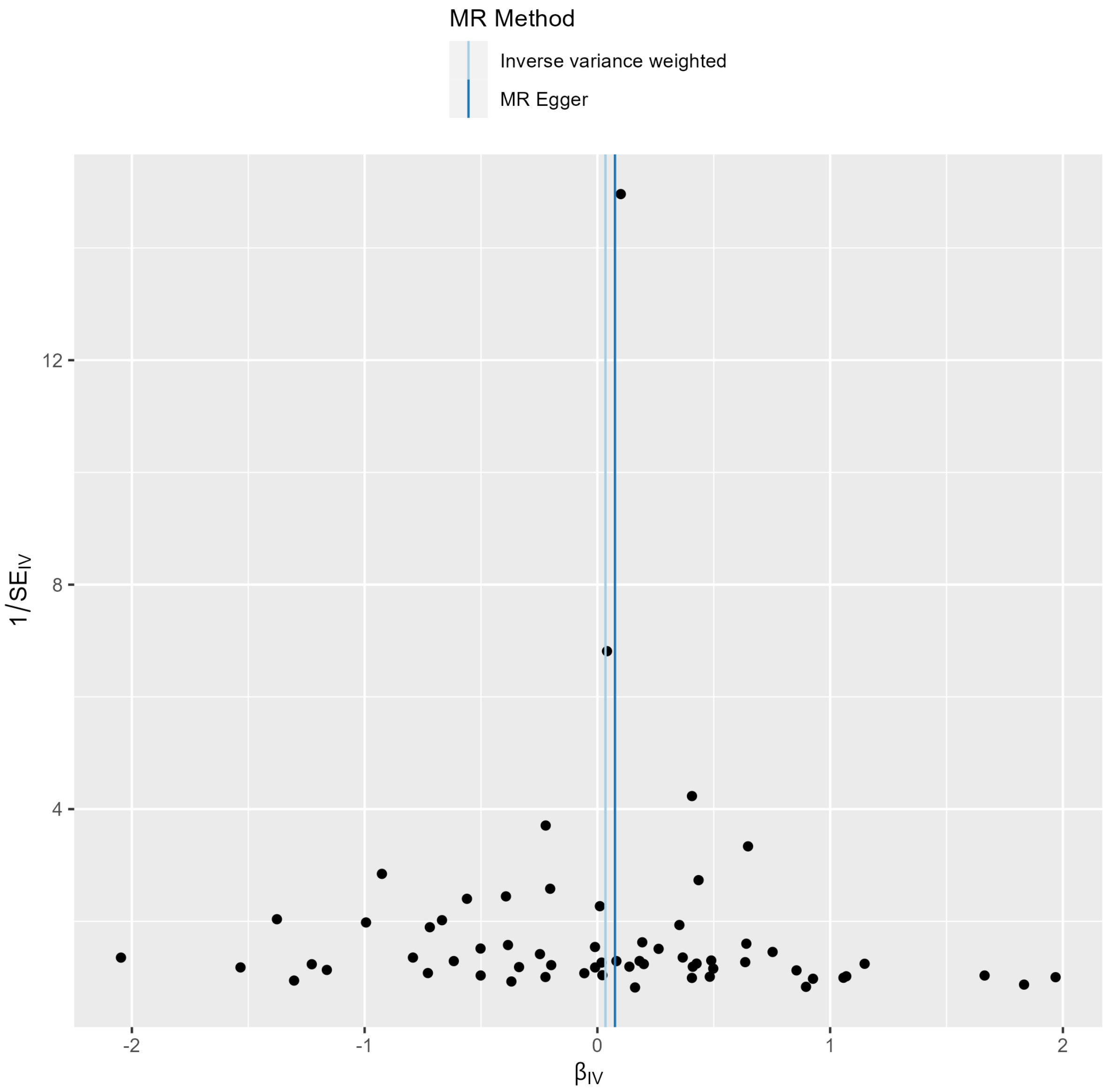

DHA-CES funnel plot

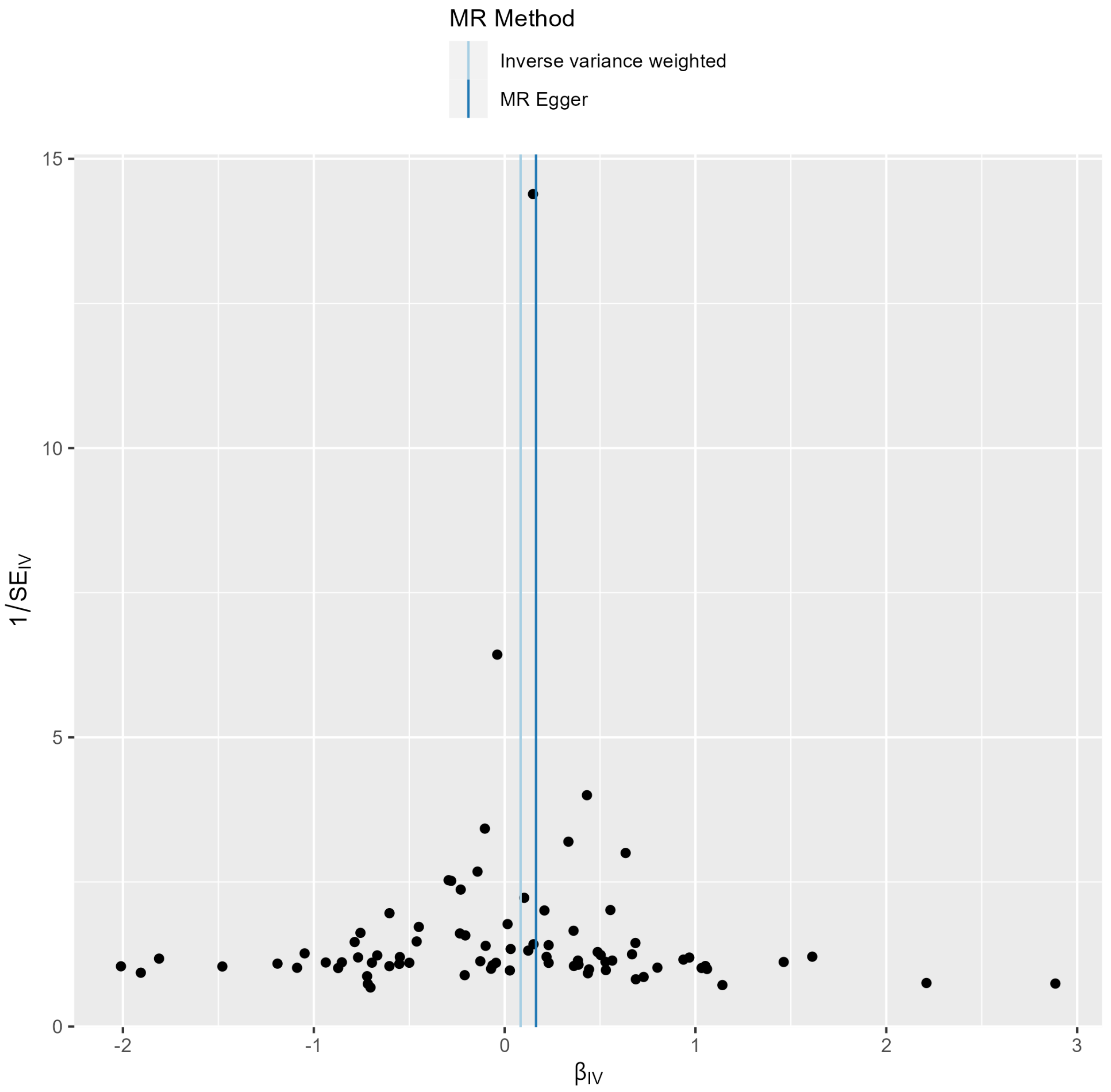

DHA-IS funnel plot

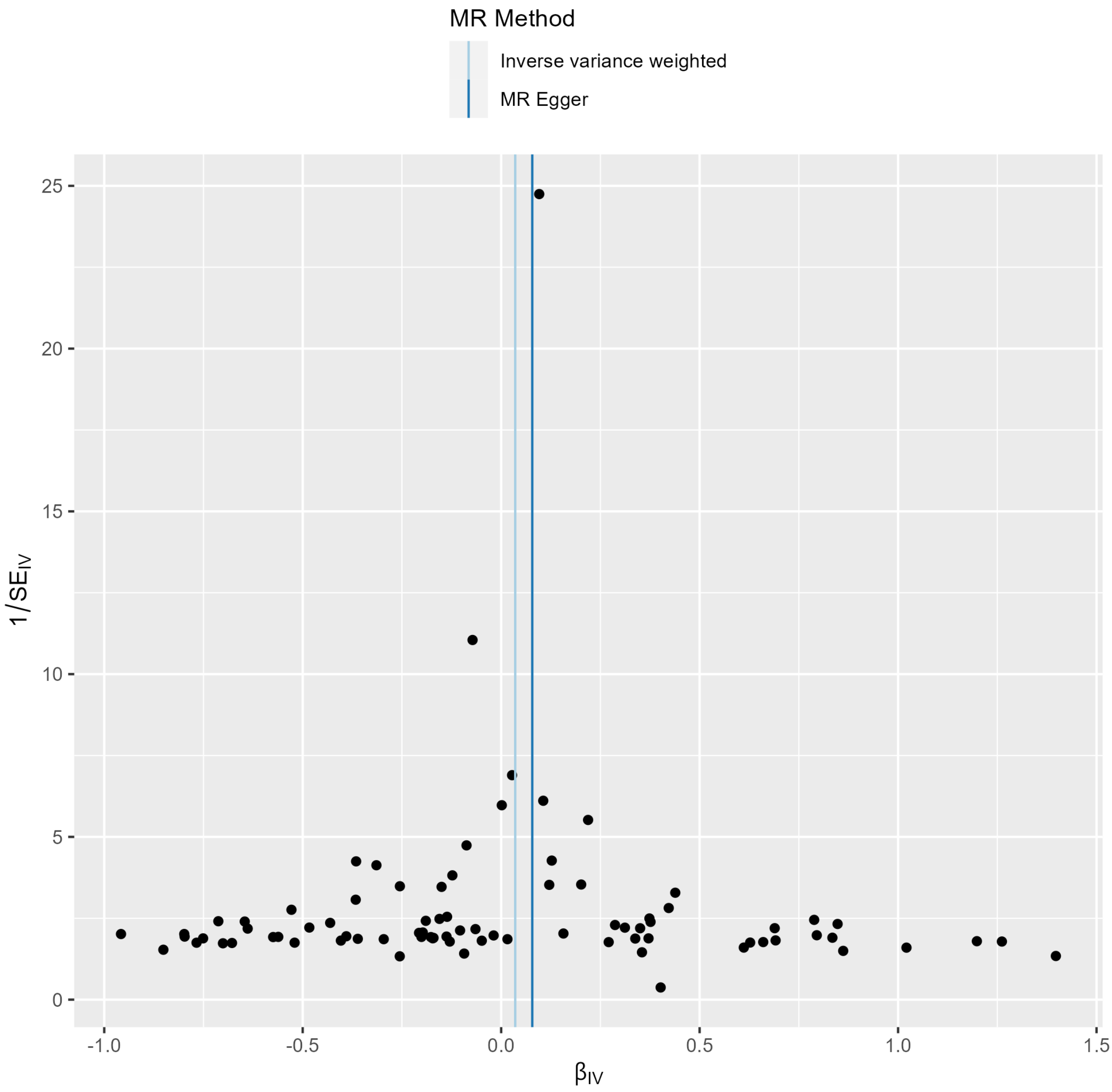

DHA-LS funnel plot

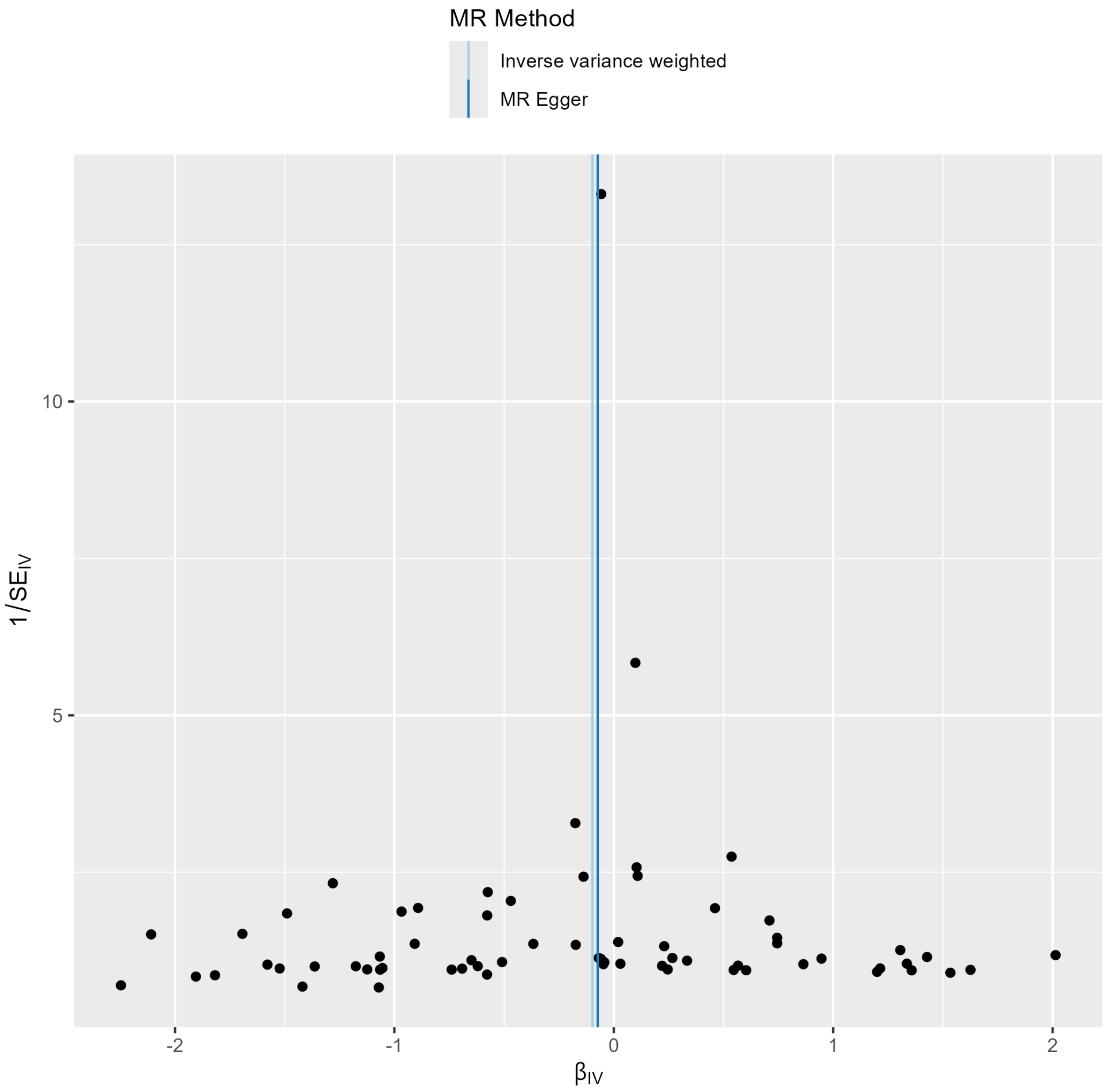

DHA-DBP funnel plot

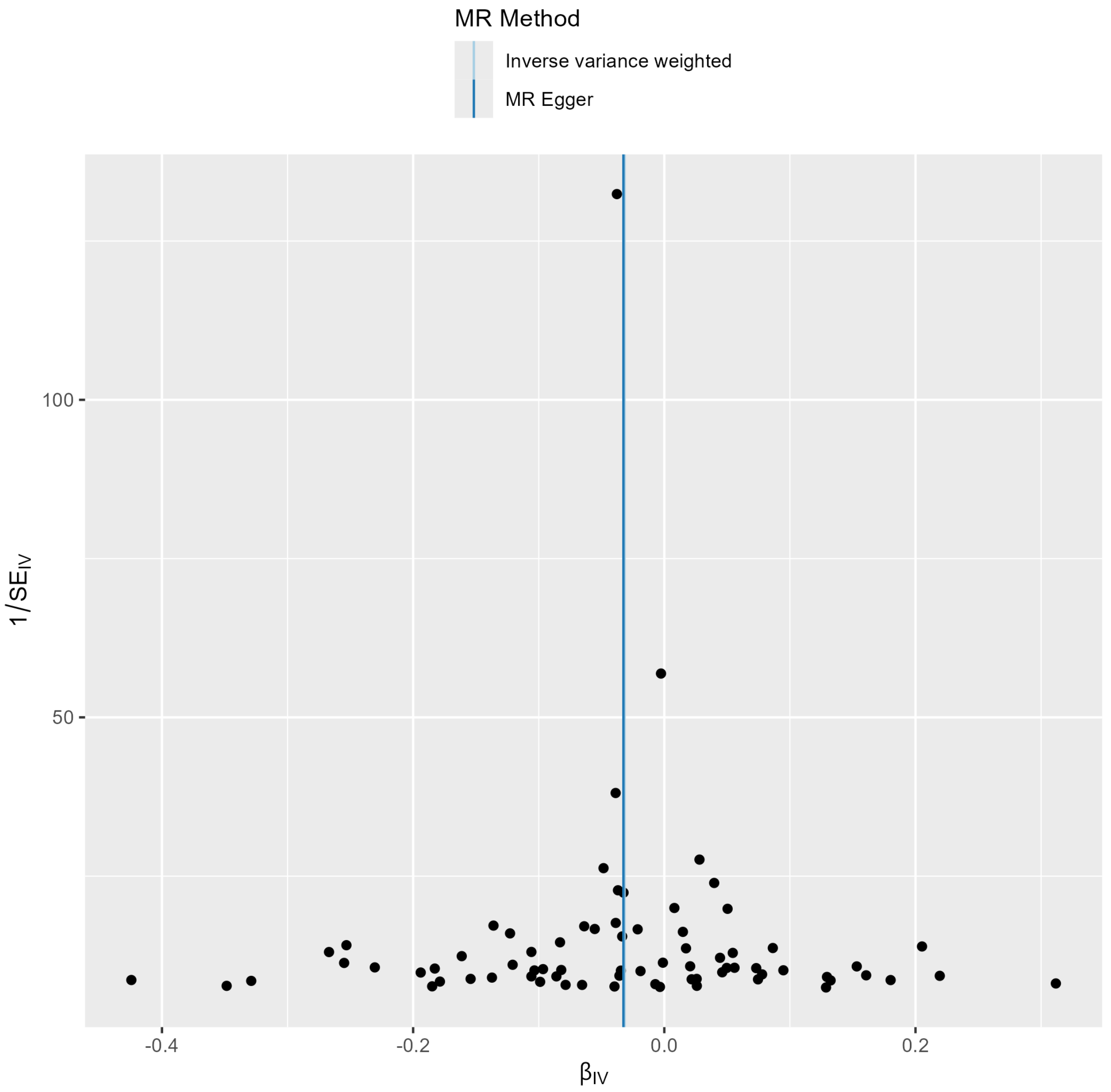

DHA-SBP funnel plot

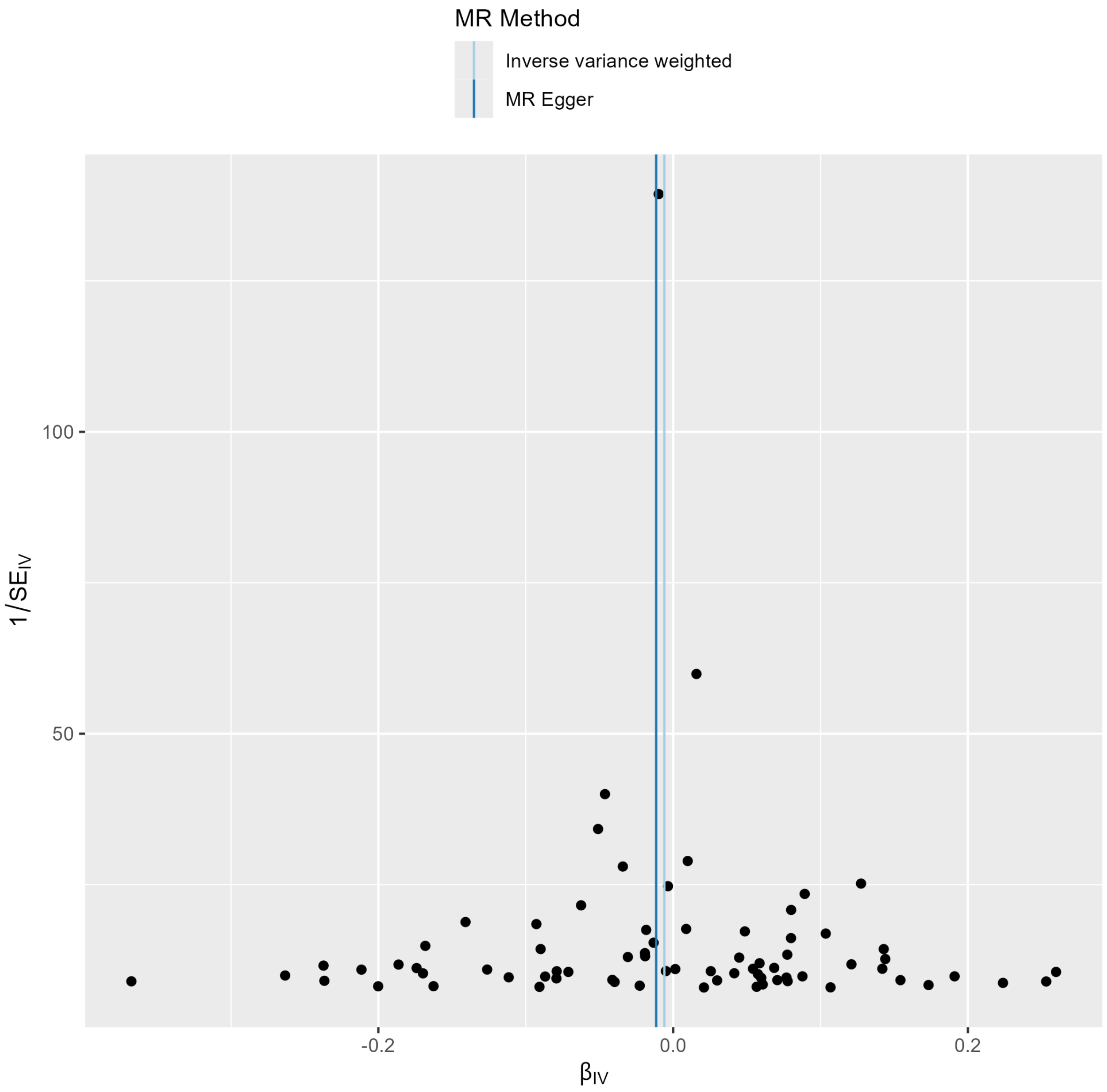

Ω-3 rate-LAS funnel plot

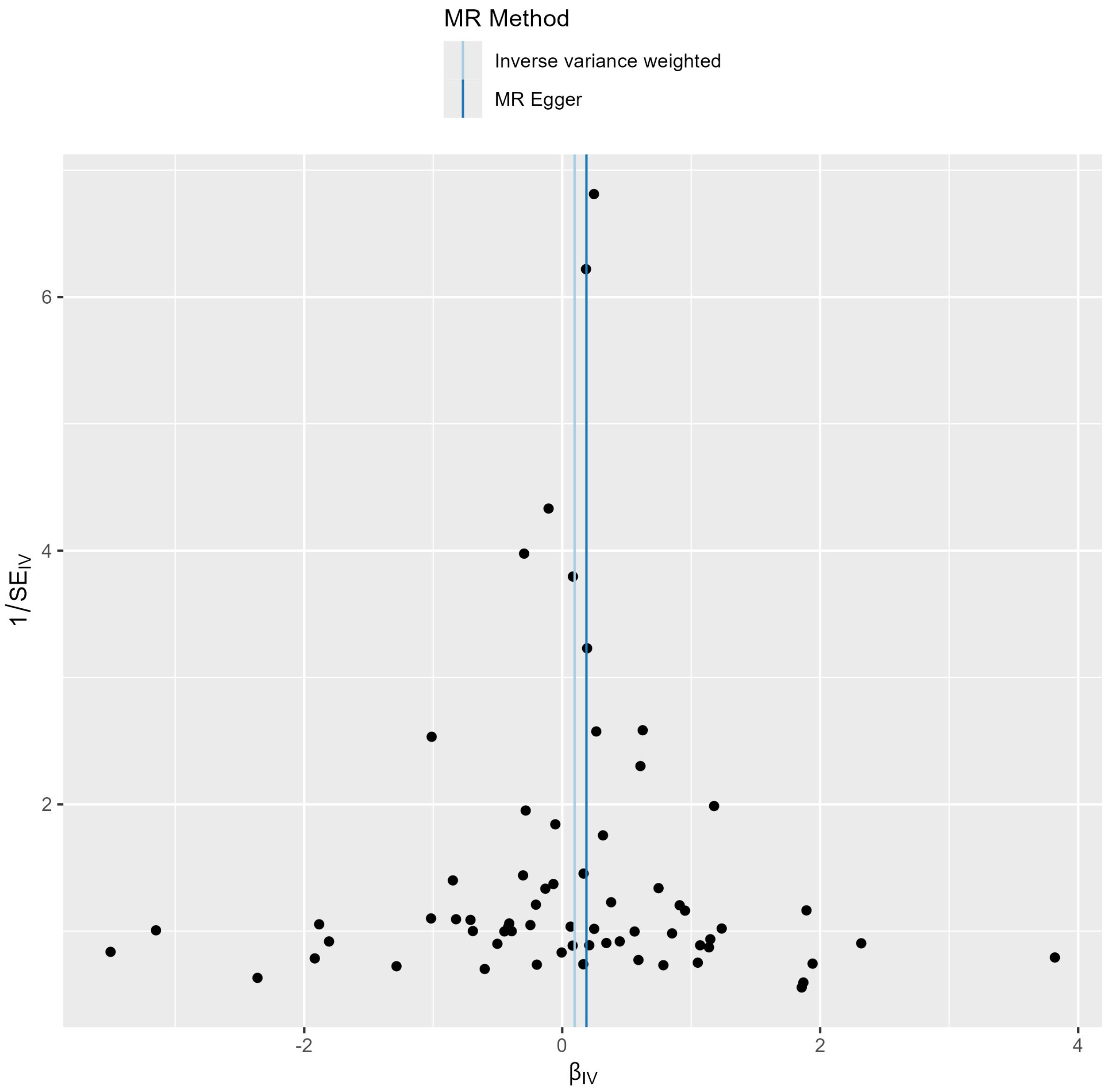

Ω-3 rate-SVS funnel plot

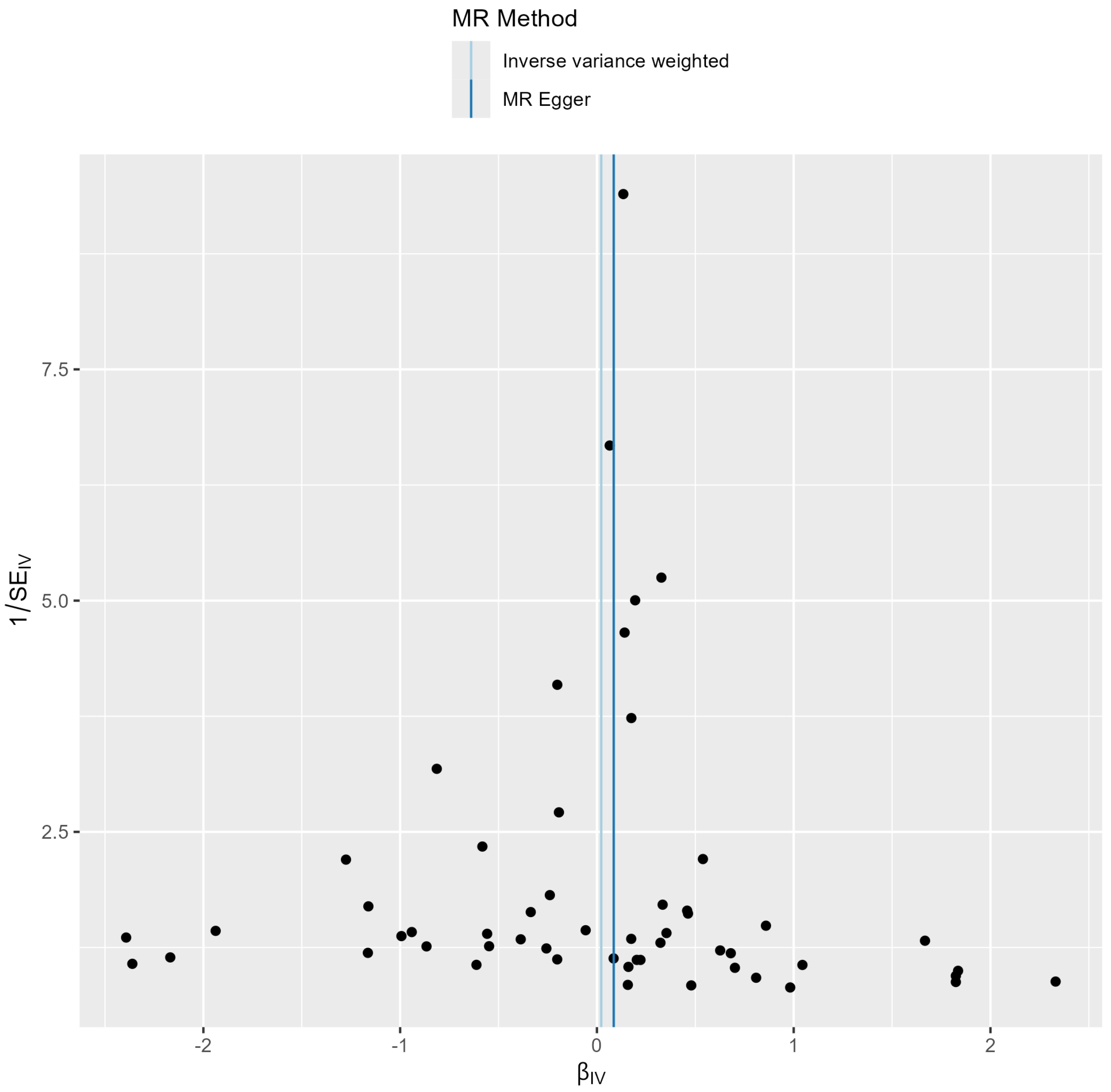

Ω-3 rate-CES funnel plot

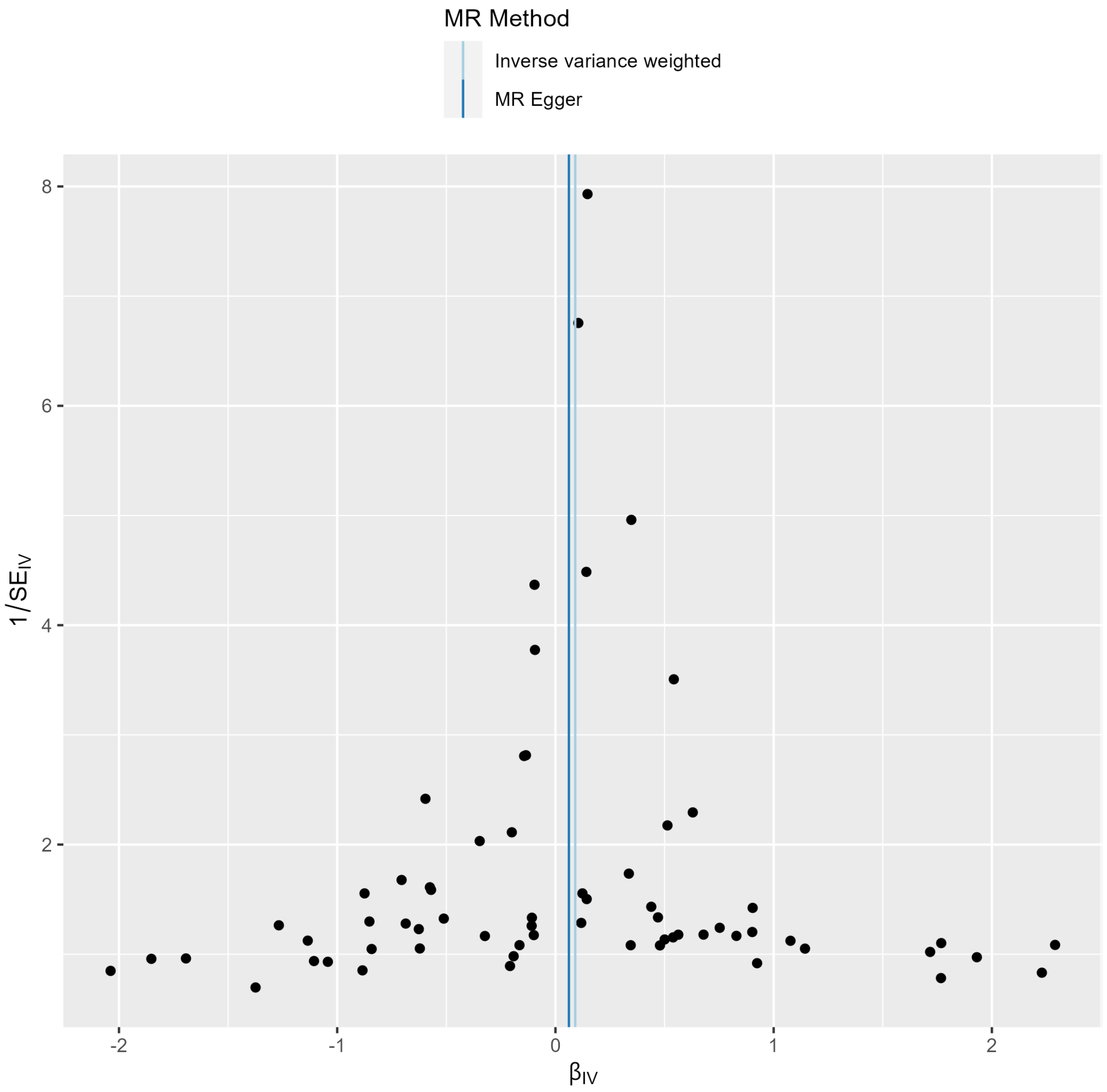

Ω-3 rate-IS funnel plot

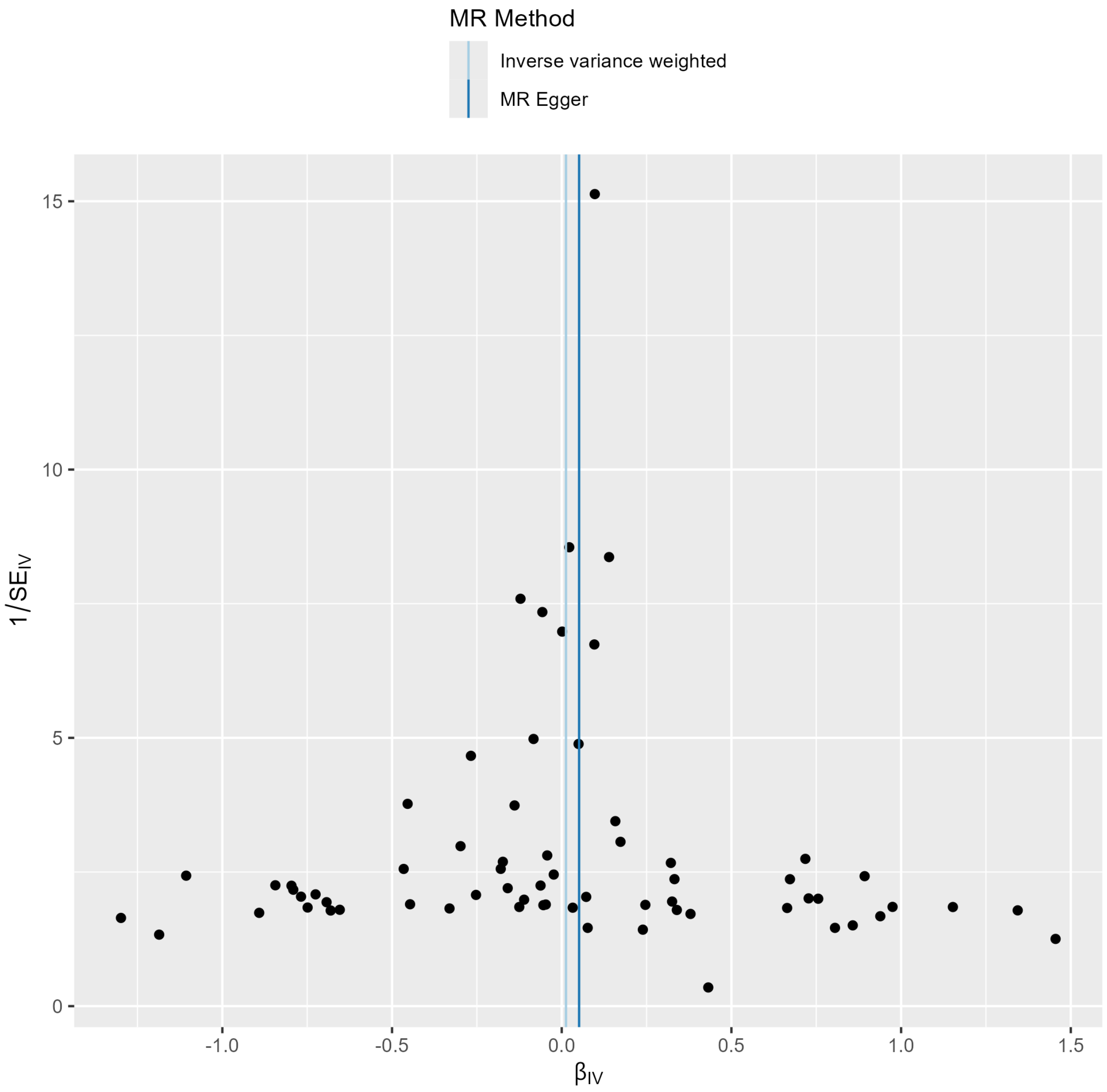

Ω-3 rate-LS funnel plot

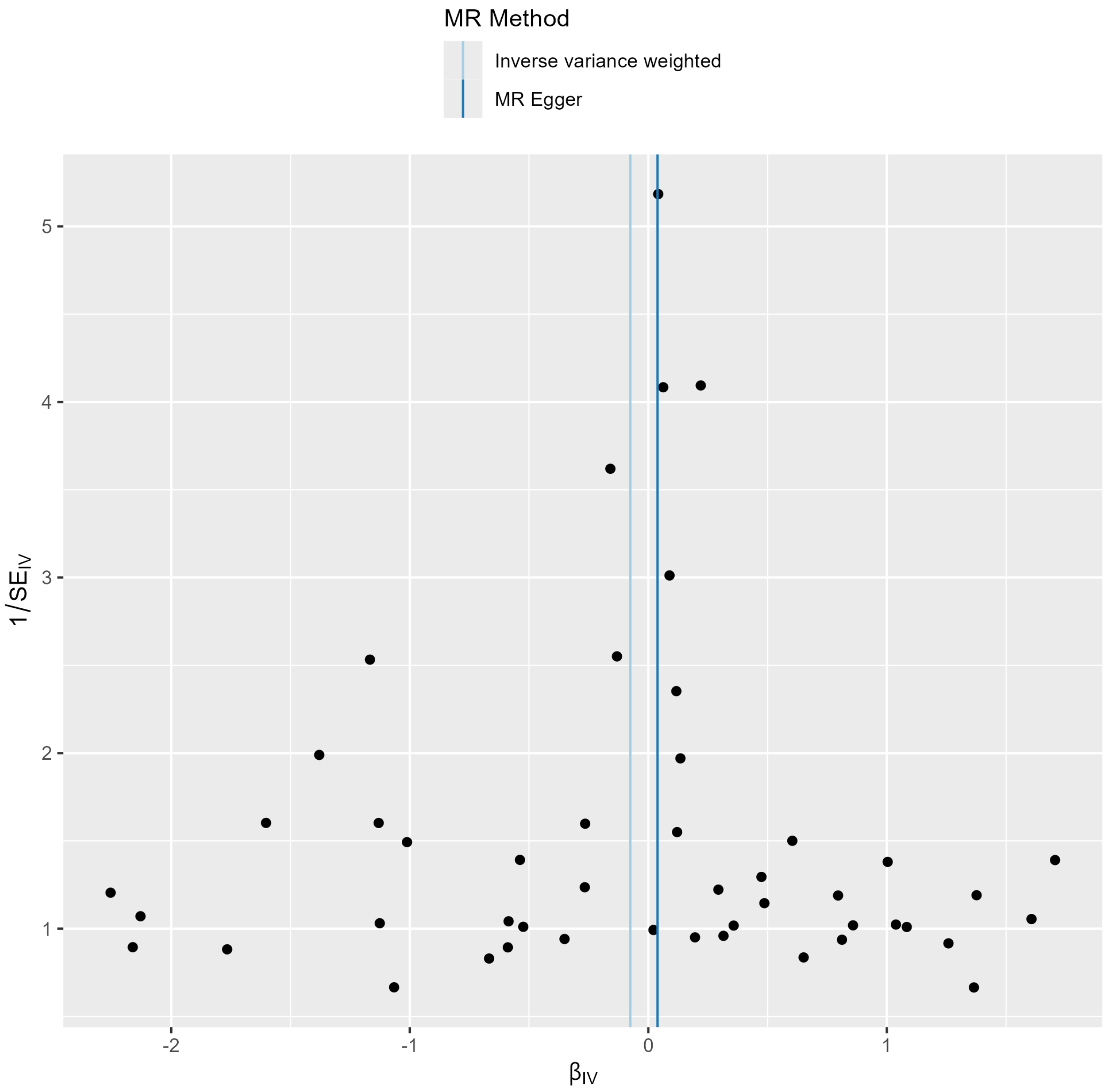

Ω-3 rate-DBP funnel plot

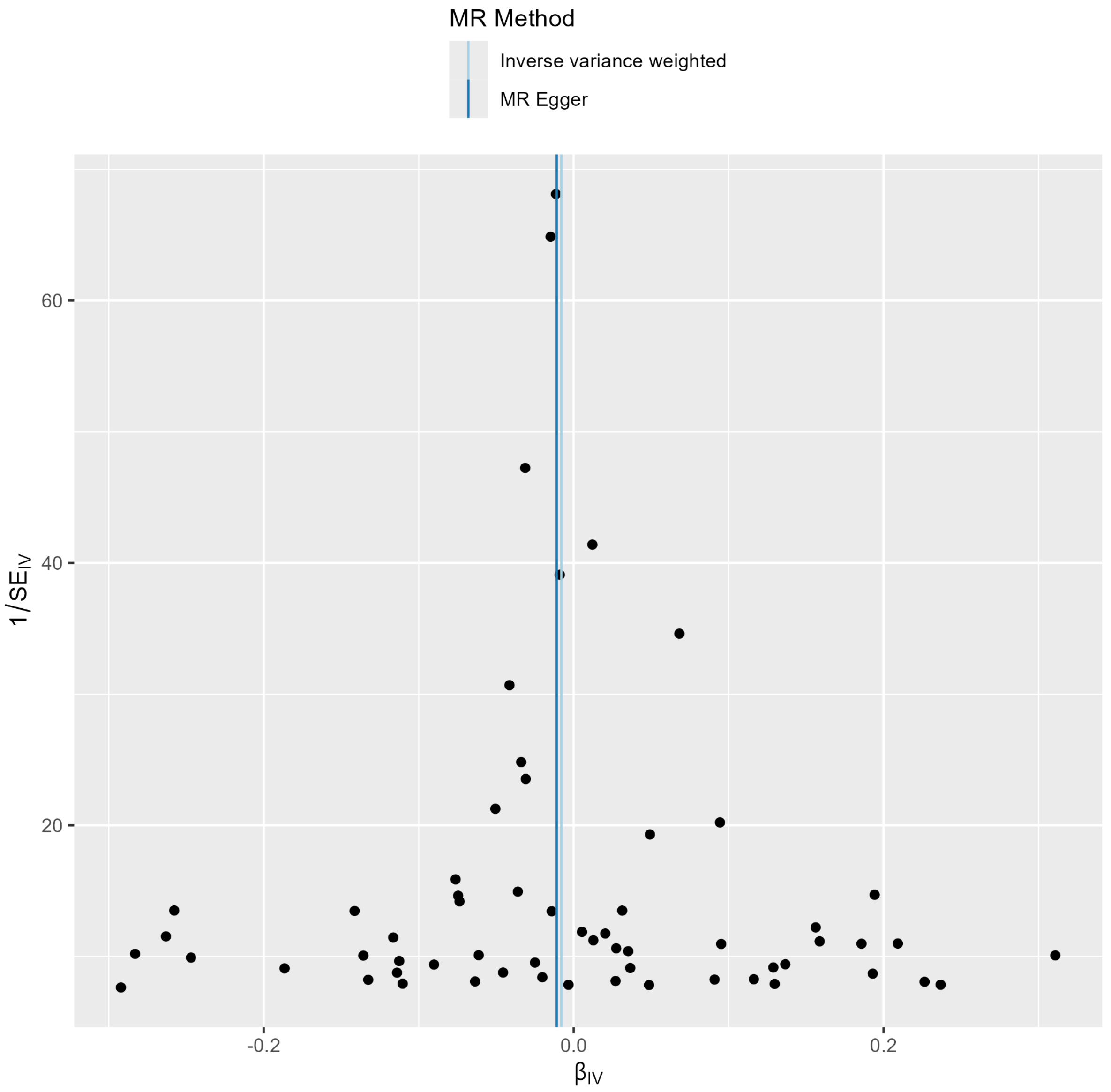

Ω-3 rate-SBP funnel plot

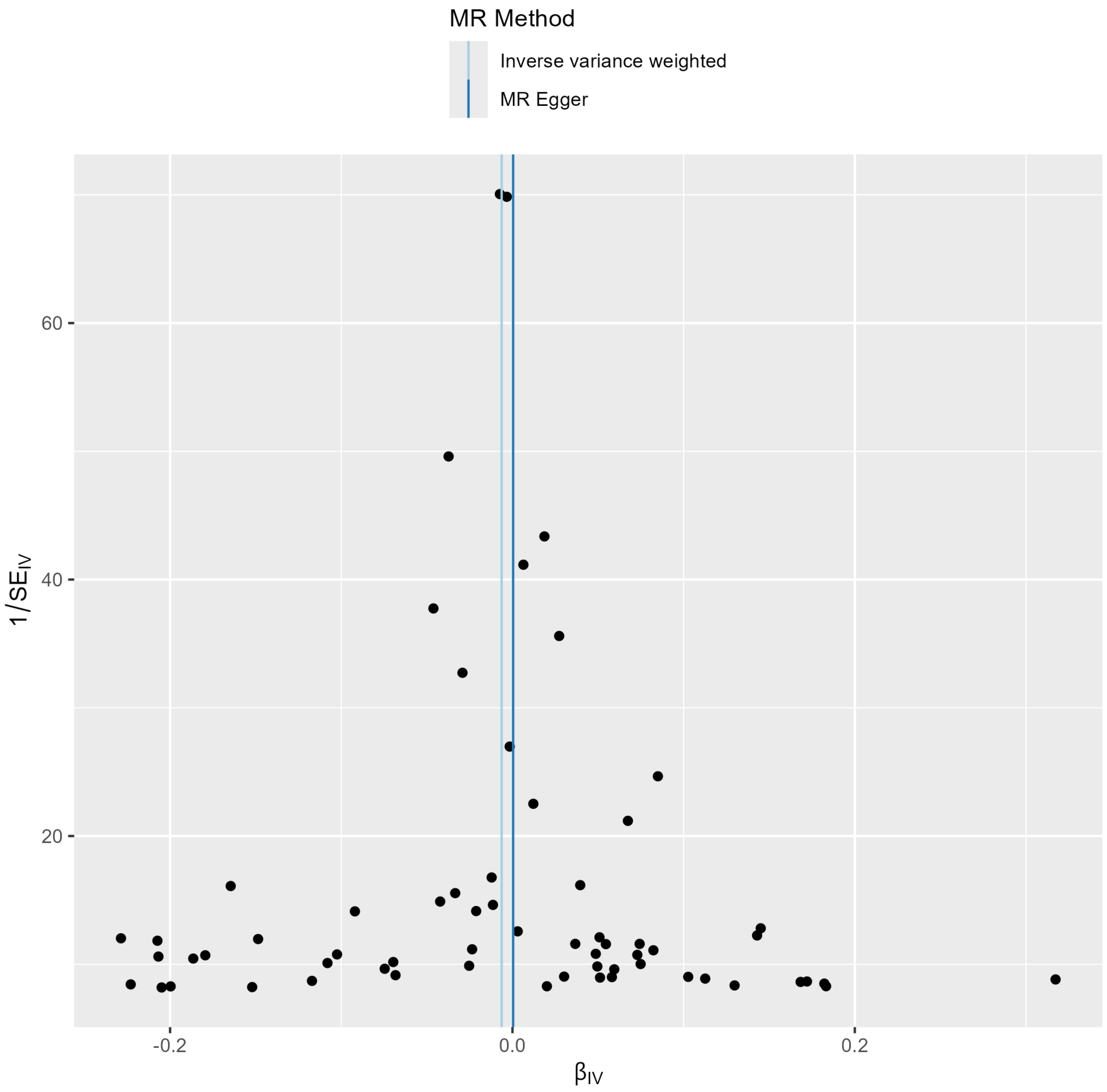

Ω-6|Ω-3-LAS funnel plot

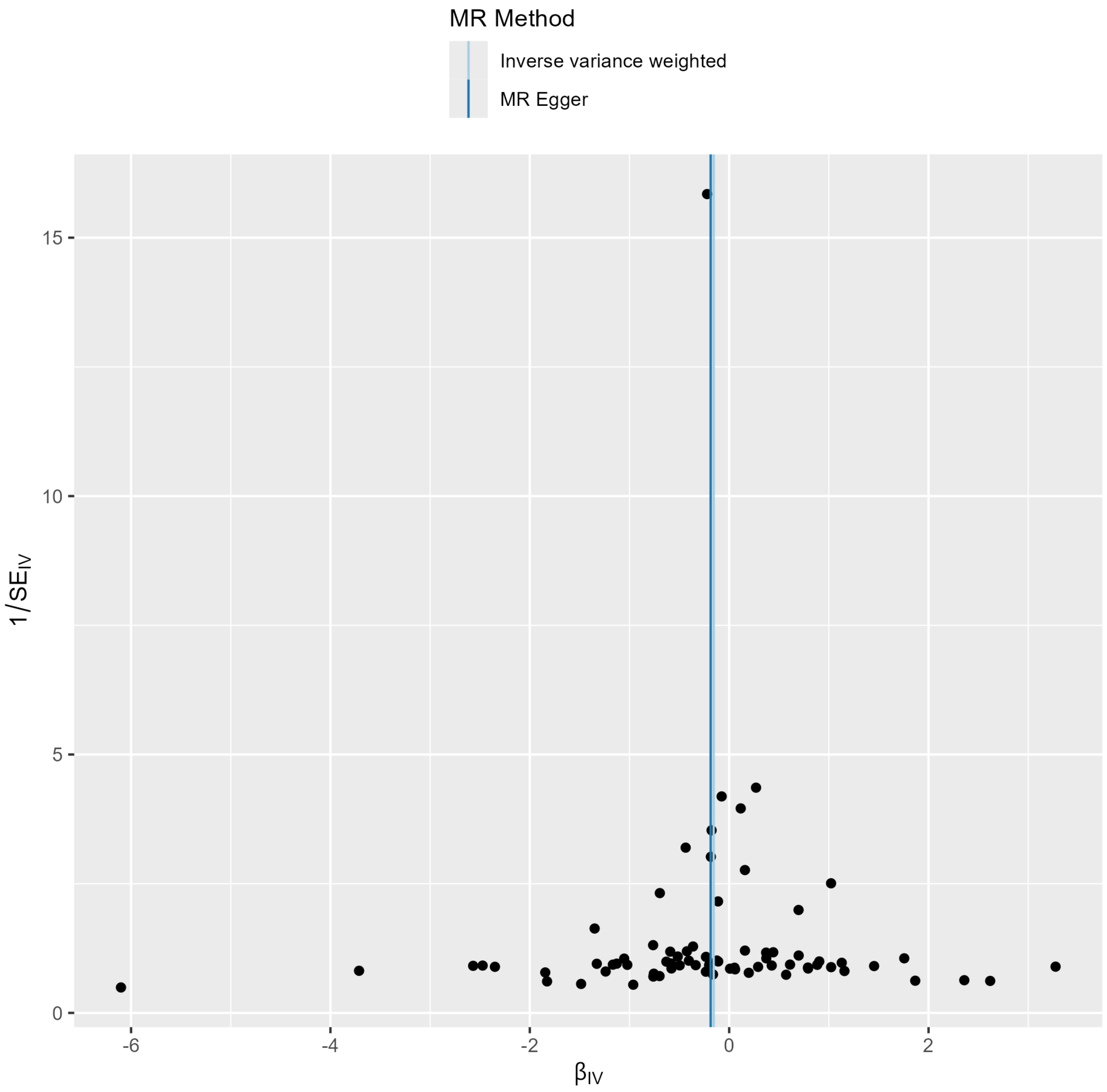

Ω-6|Ω-3-SVS funnel plot

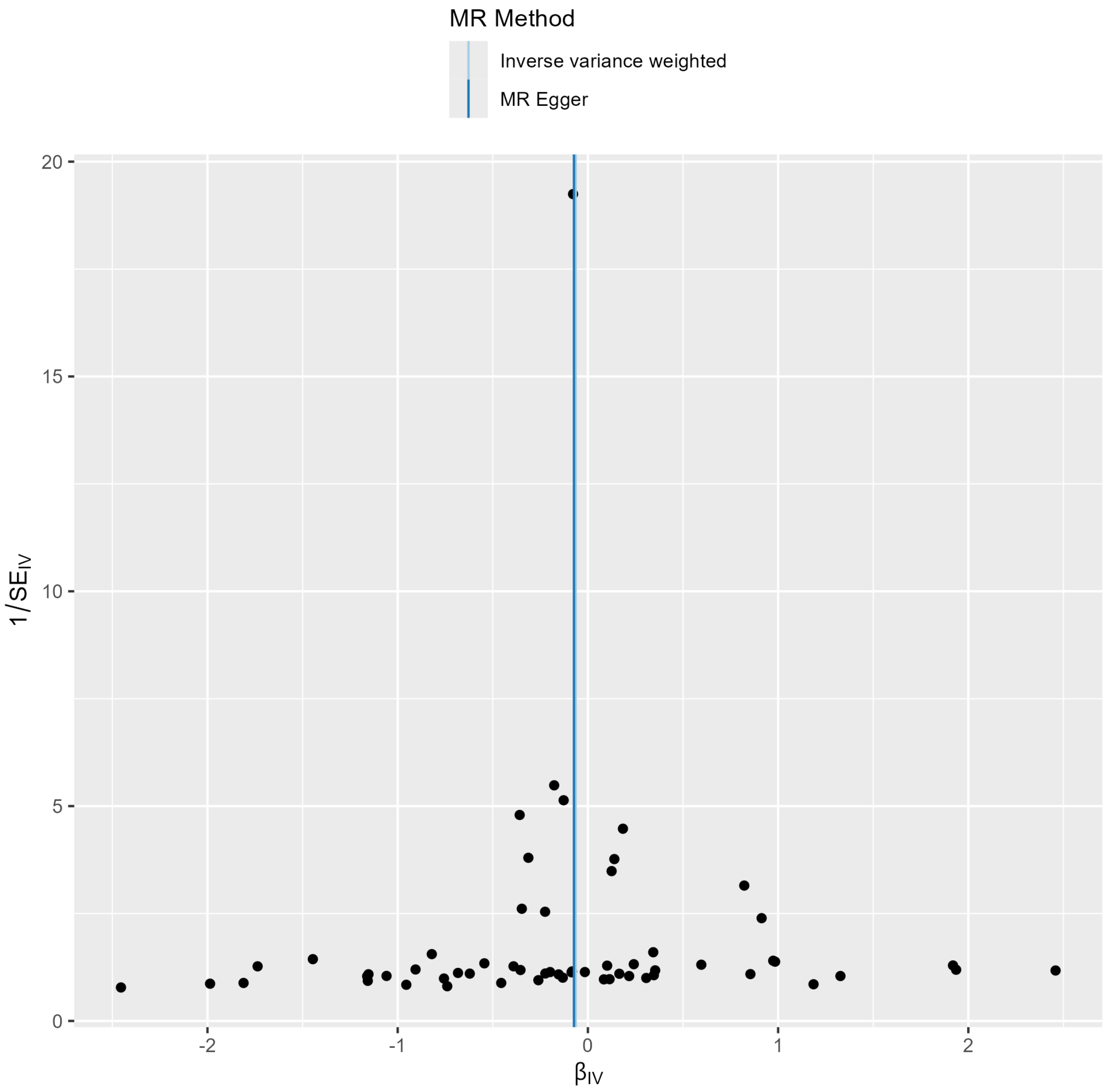

Ω-6|Ω-3-CES funnel plot

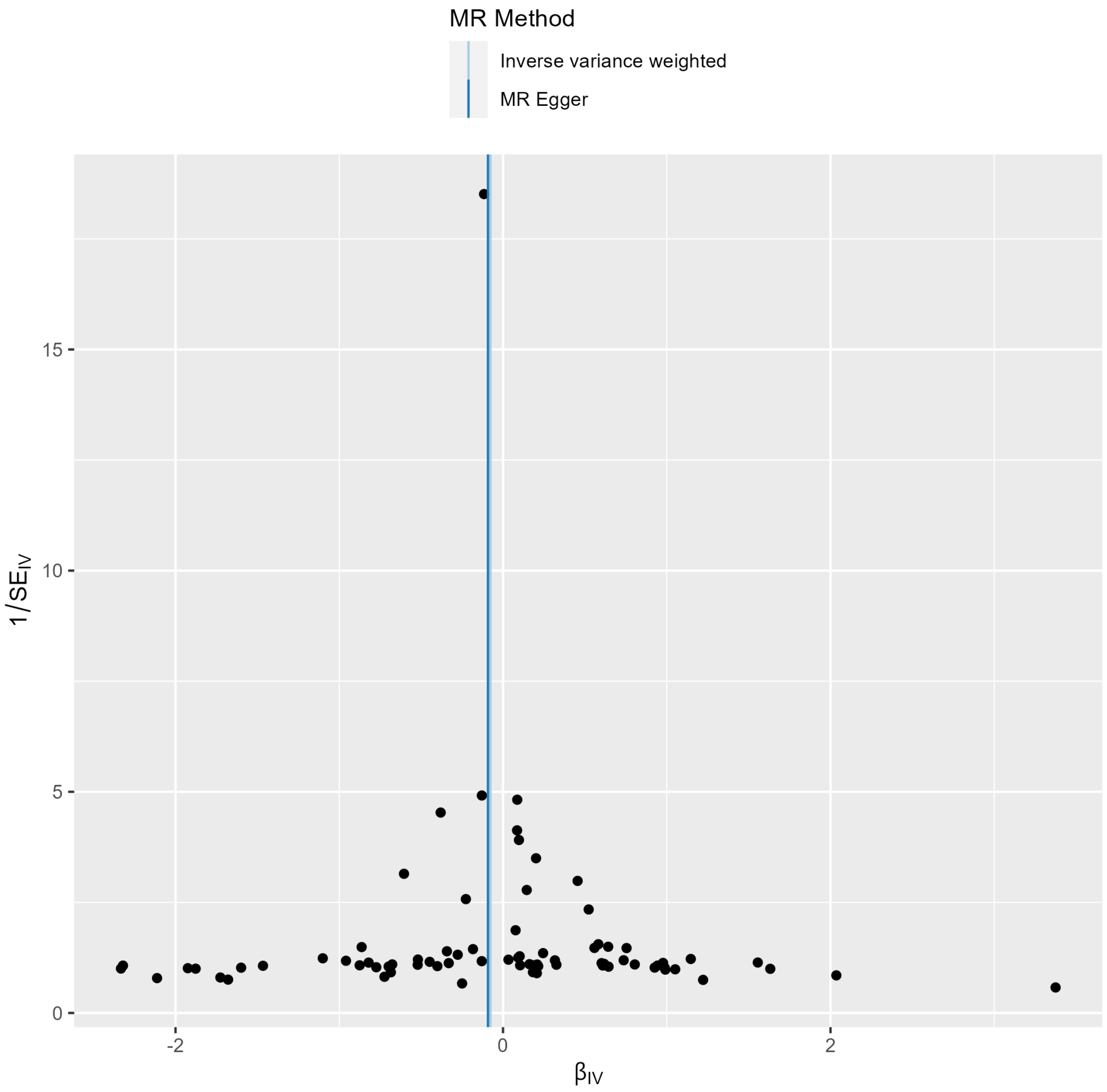

Ω-6|Ω-3-IS funnel plot

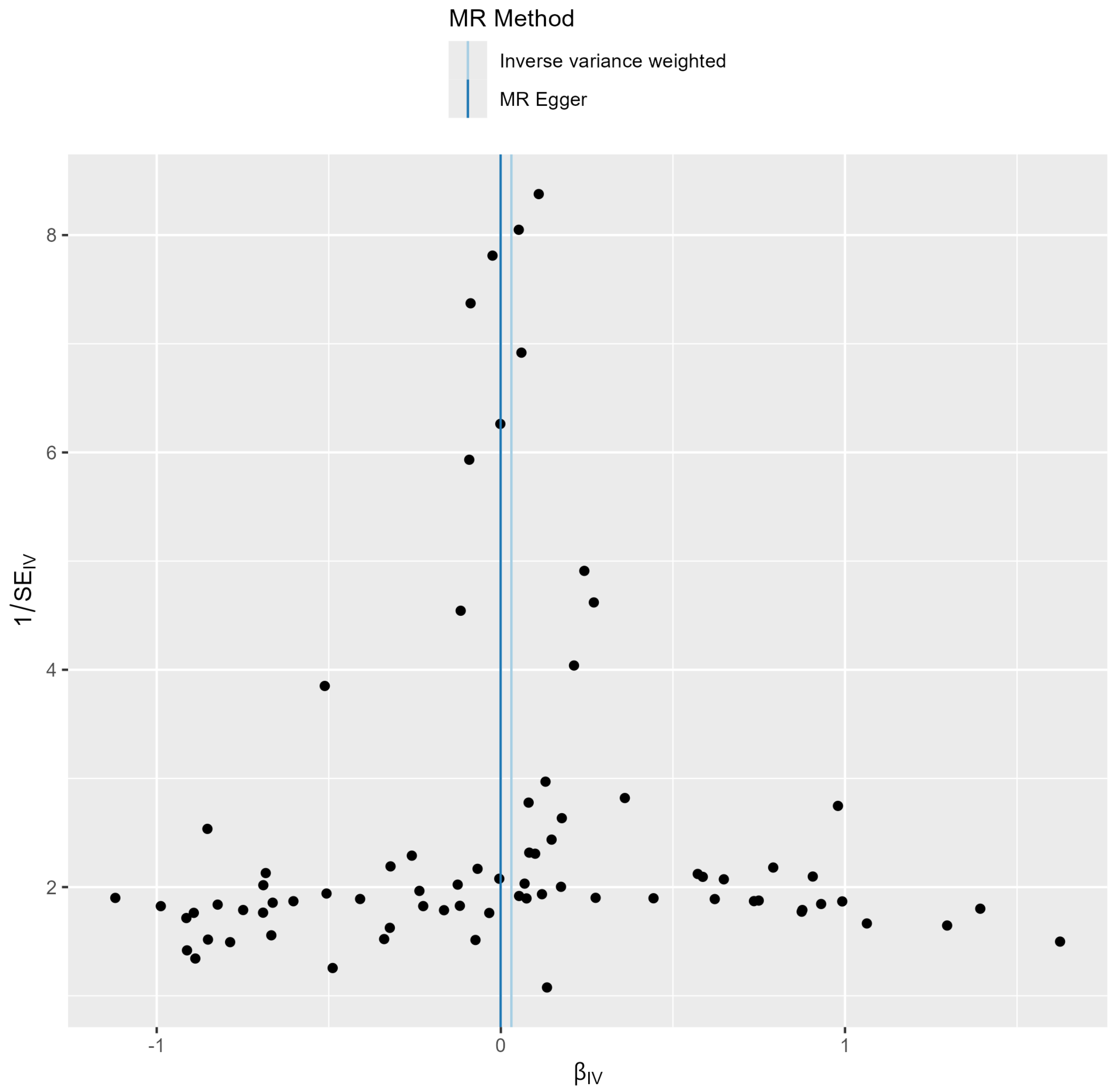

Ω-6|Ω-3-LS funnel plot

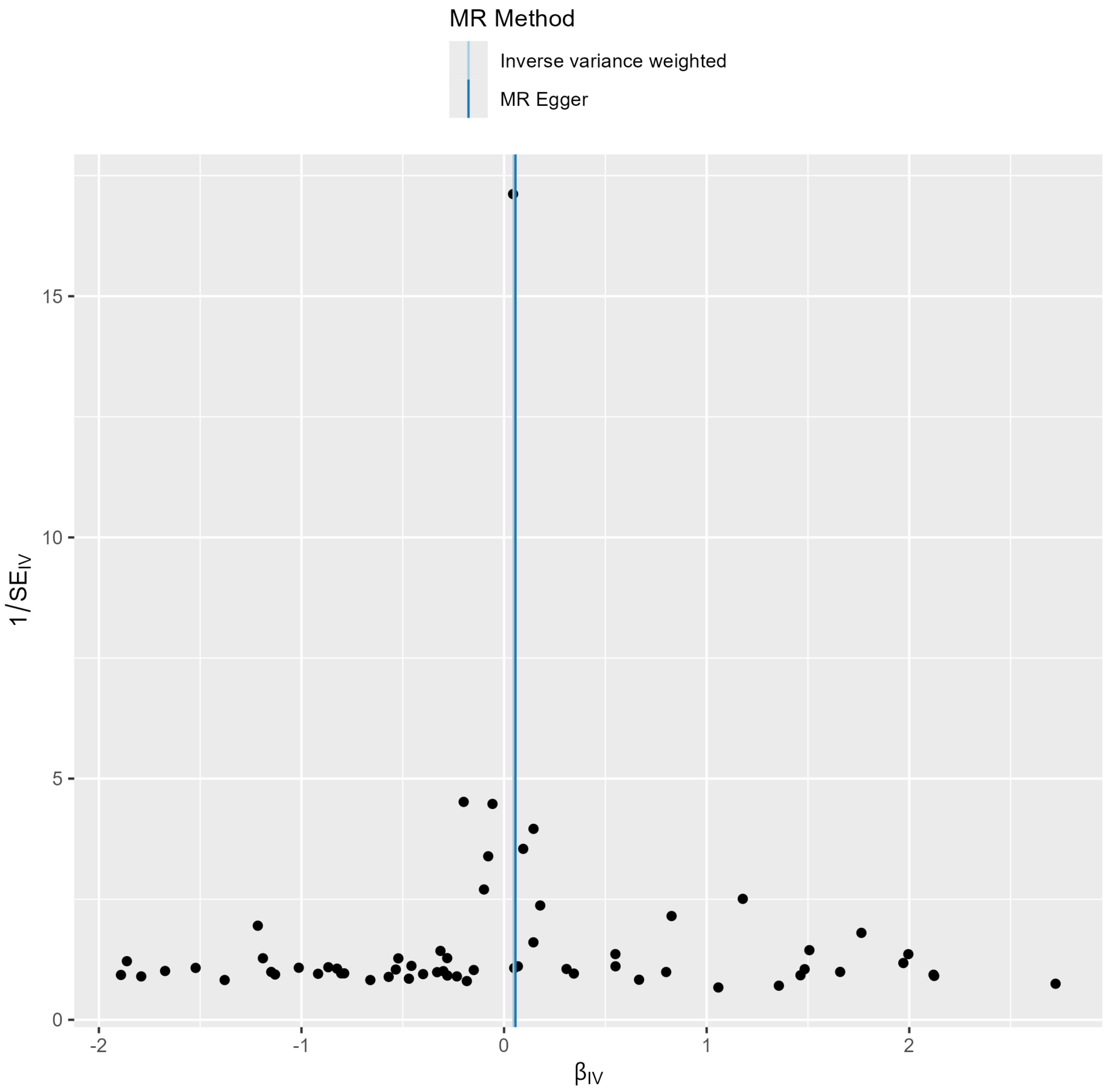

Ω-6|Ω-3-DBP funnel plot

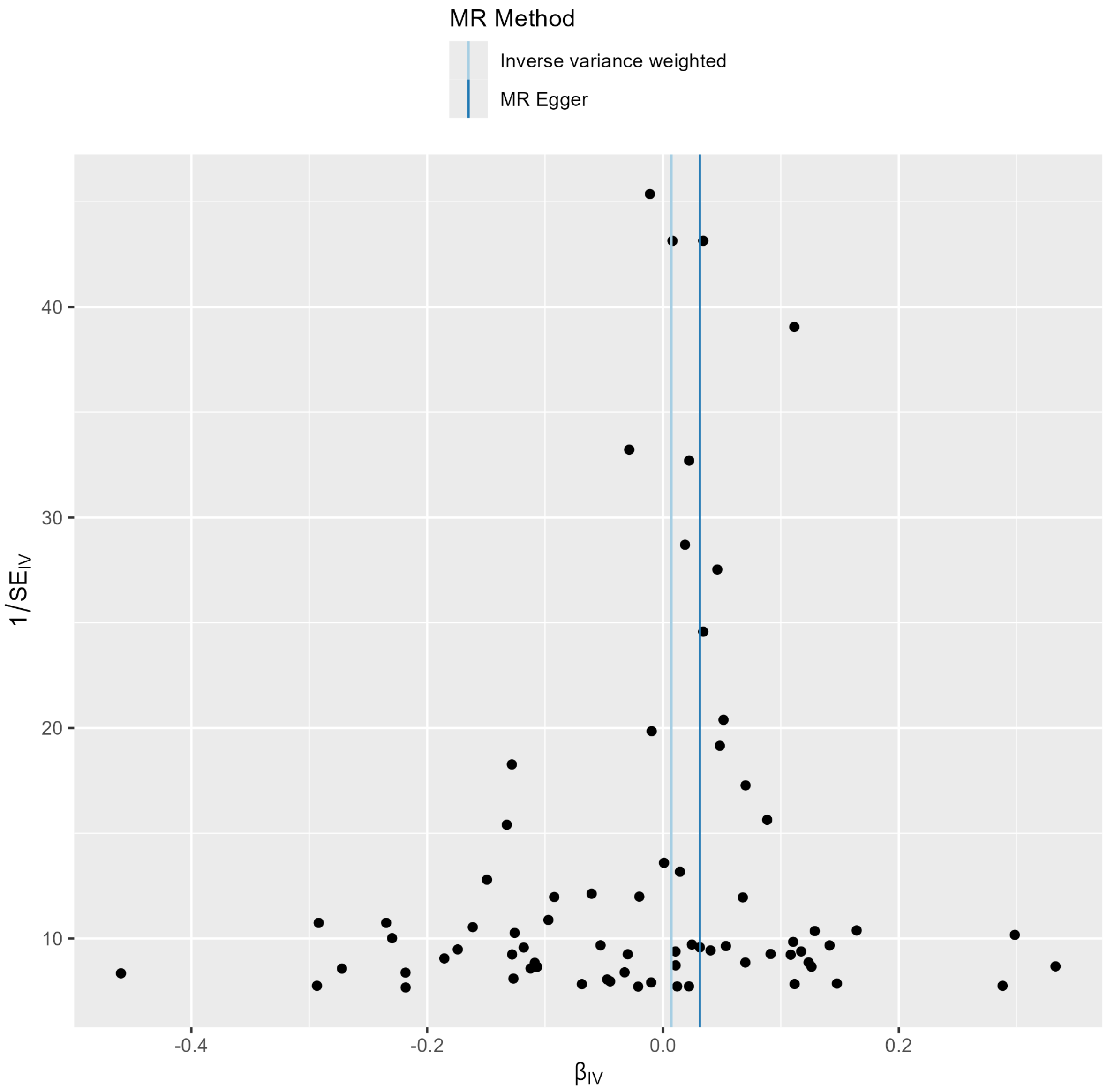

Ω-6|Ω-3-SBP funnel plot

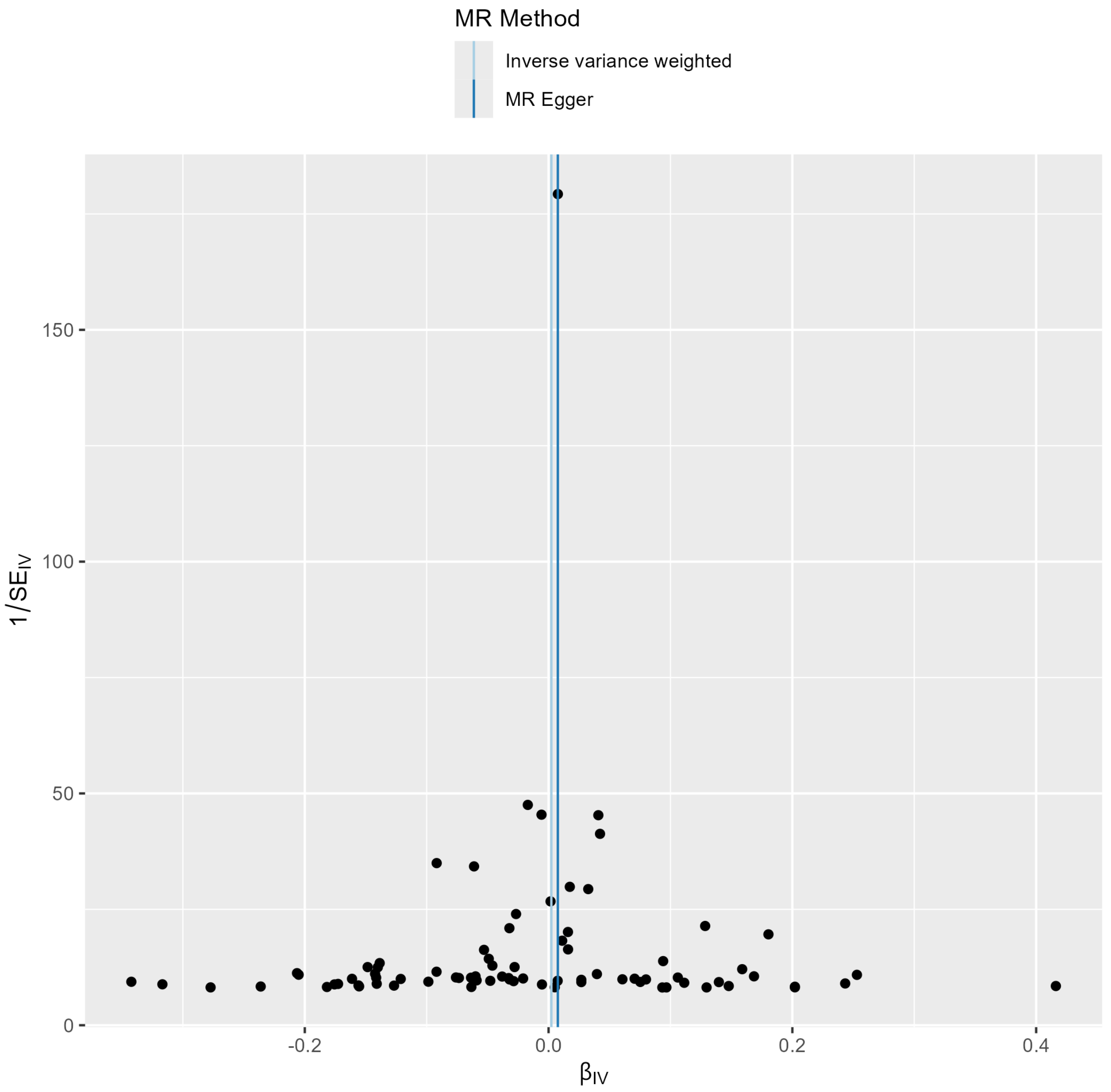

omegas-DBP leave-one-out

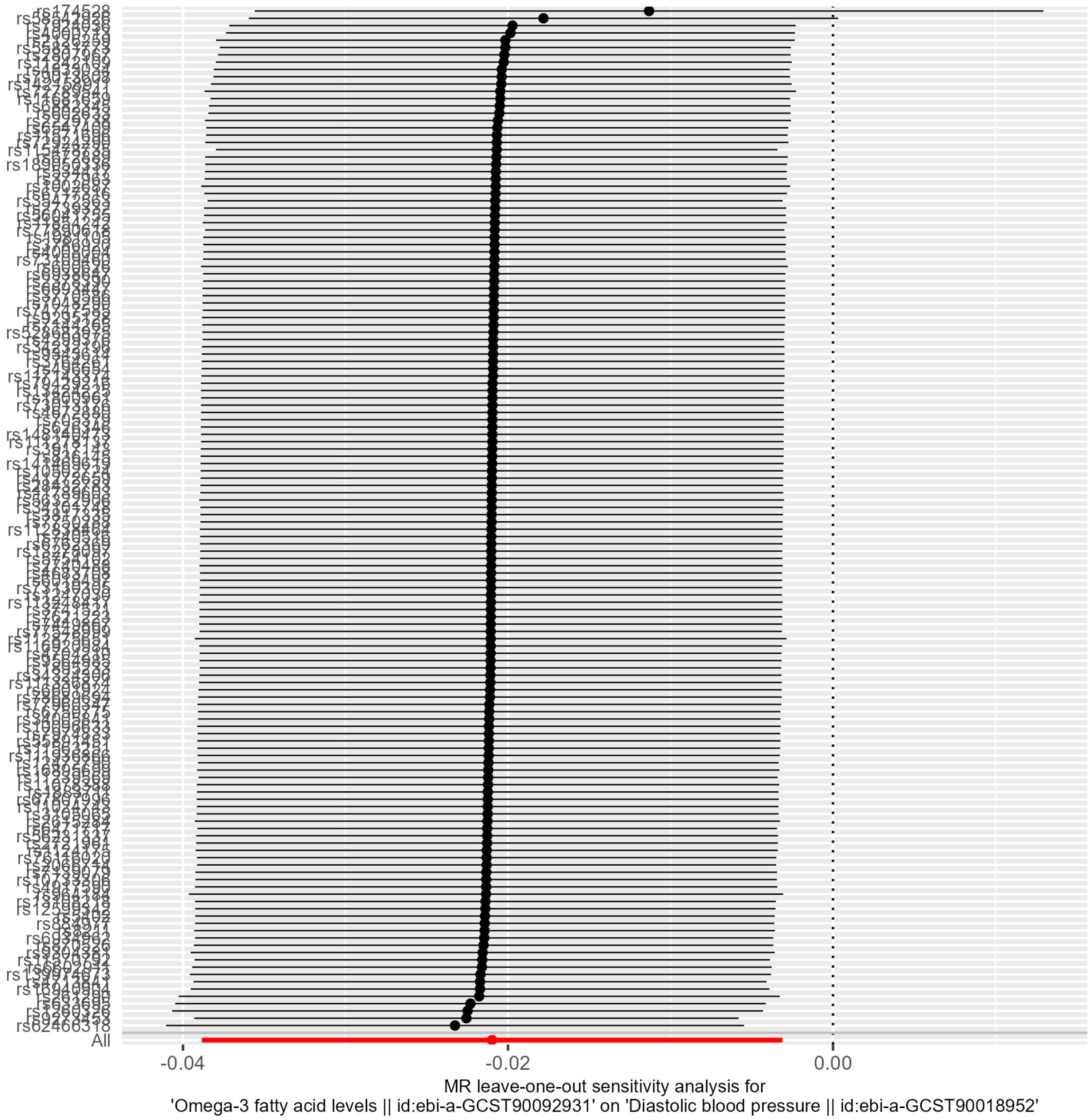

omegas-SBP leave-one-out

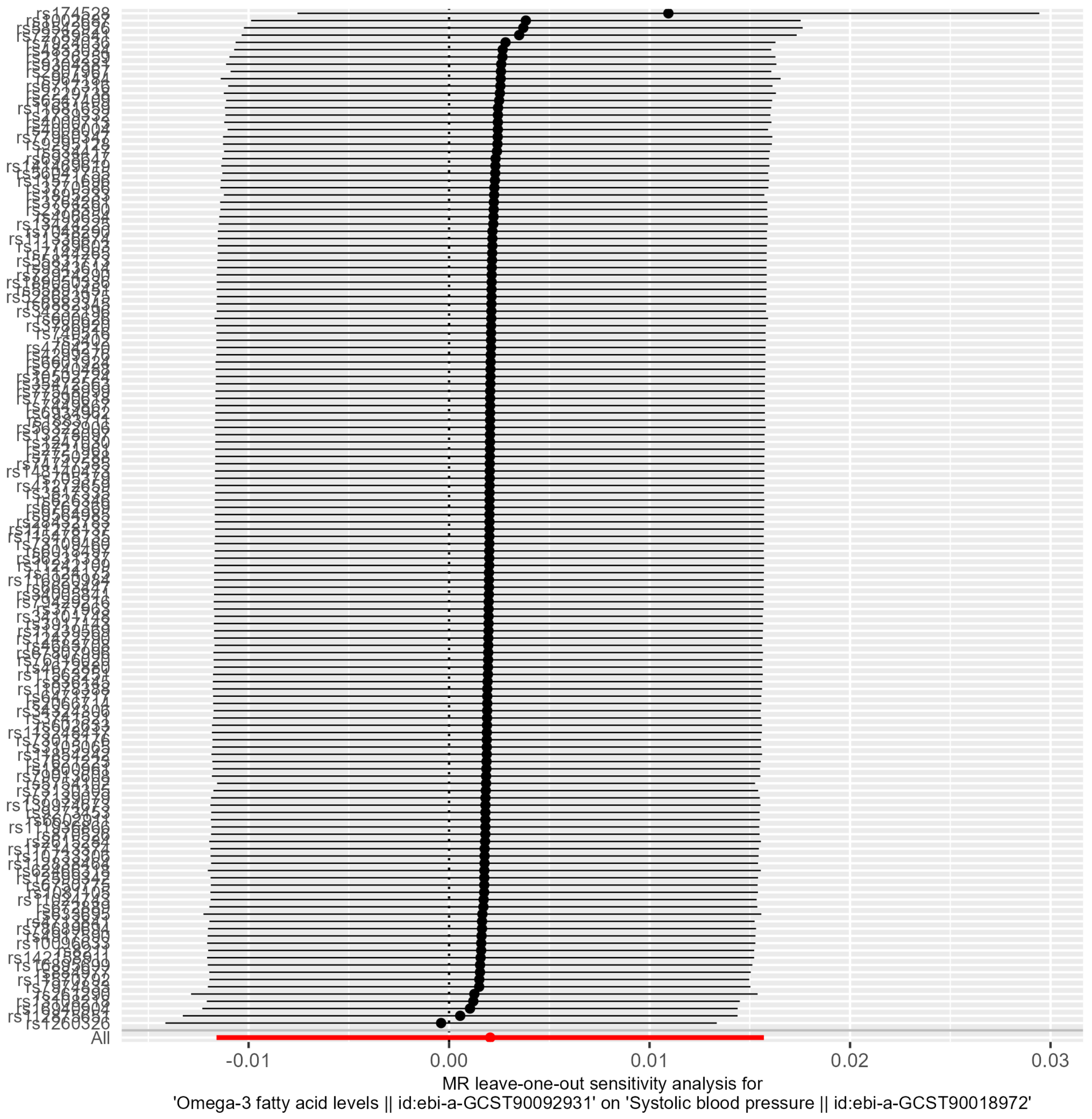

omegas-EH leave-one-out

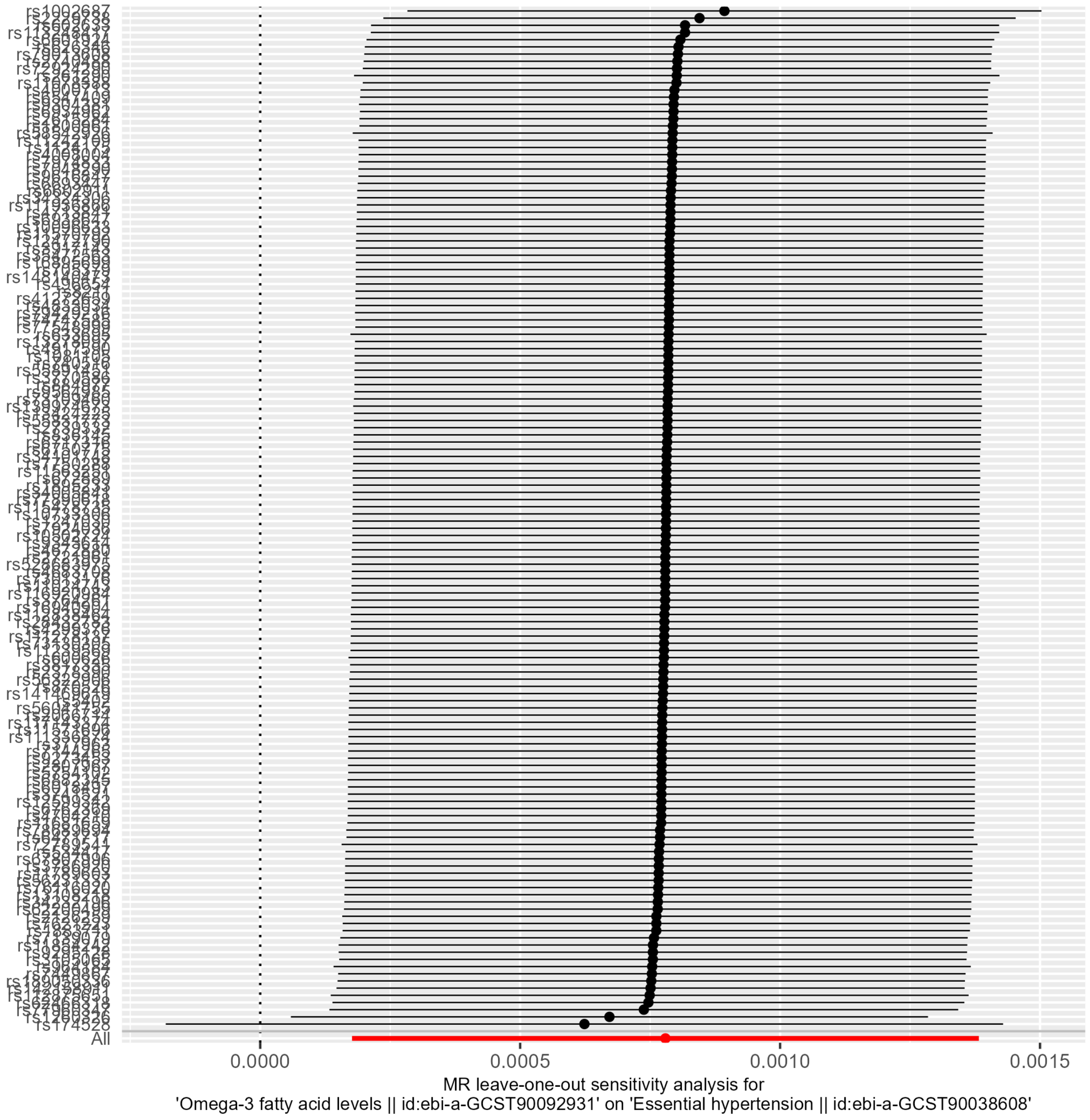

omegas-DBP scatter plot

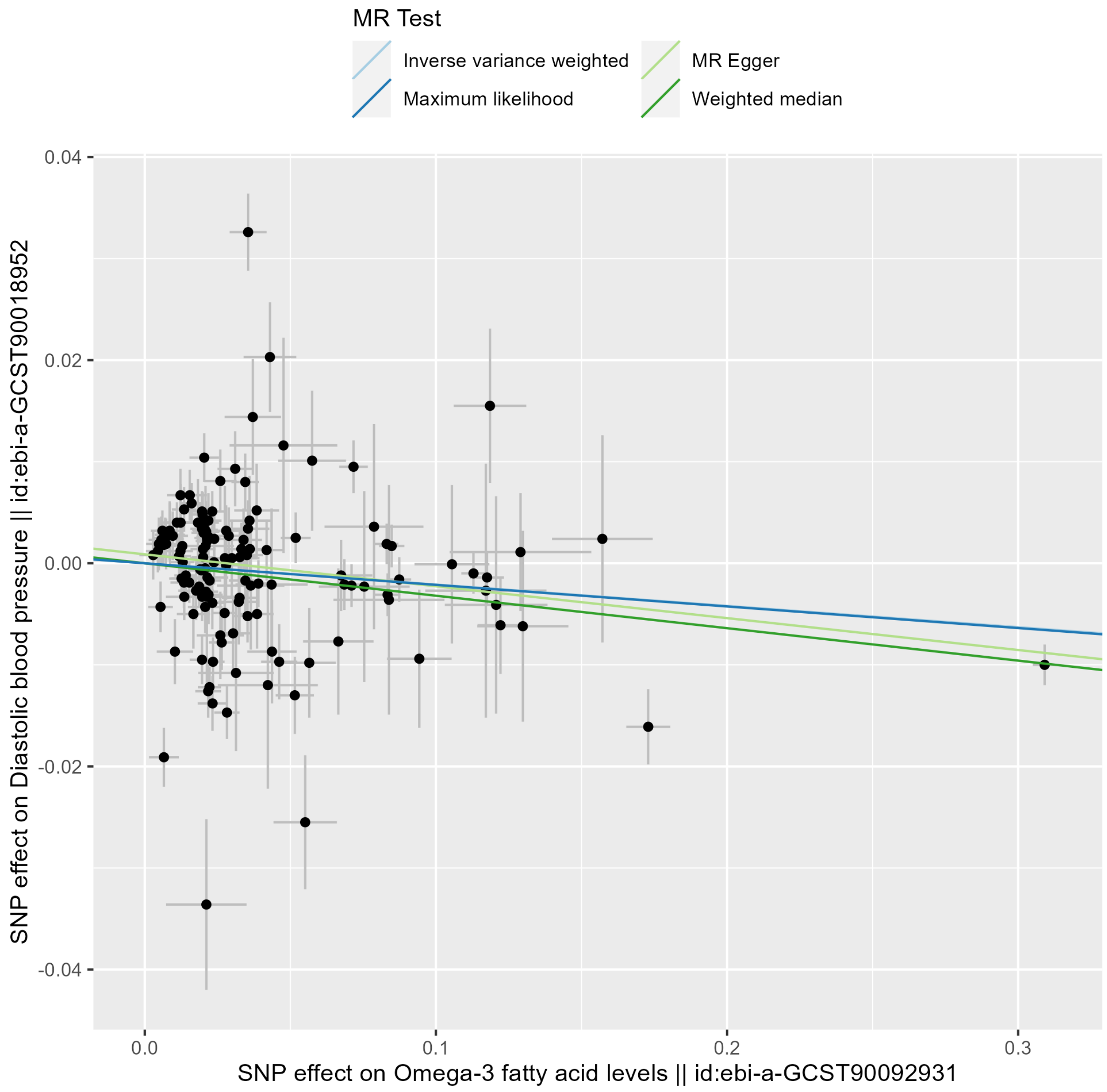

omegas-SBP scatter plot

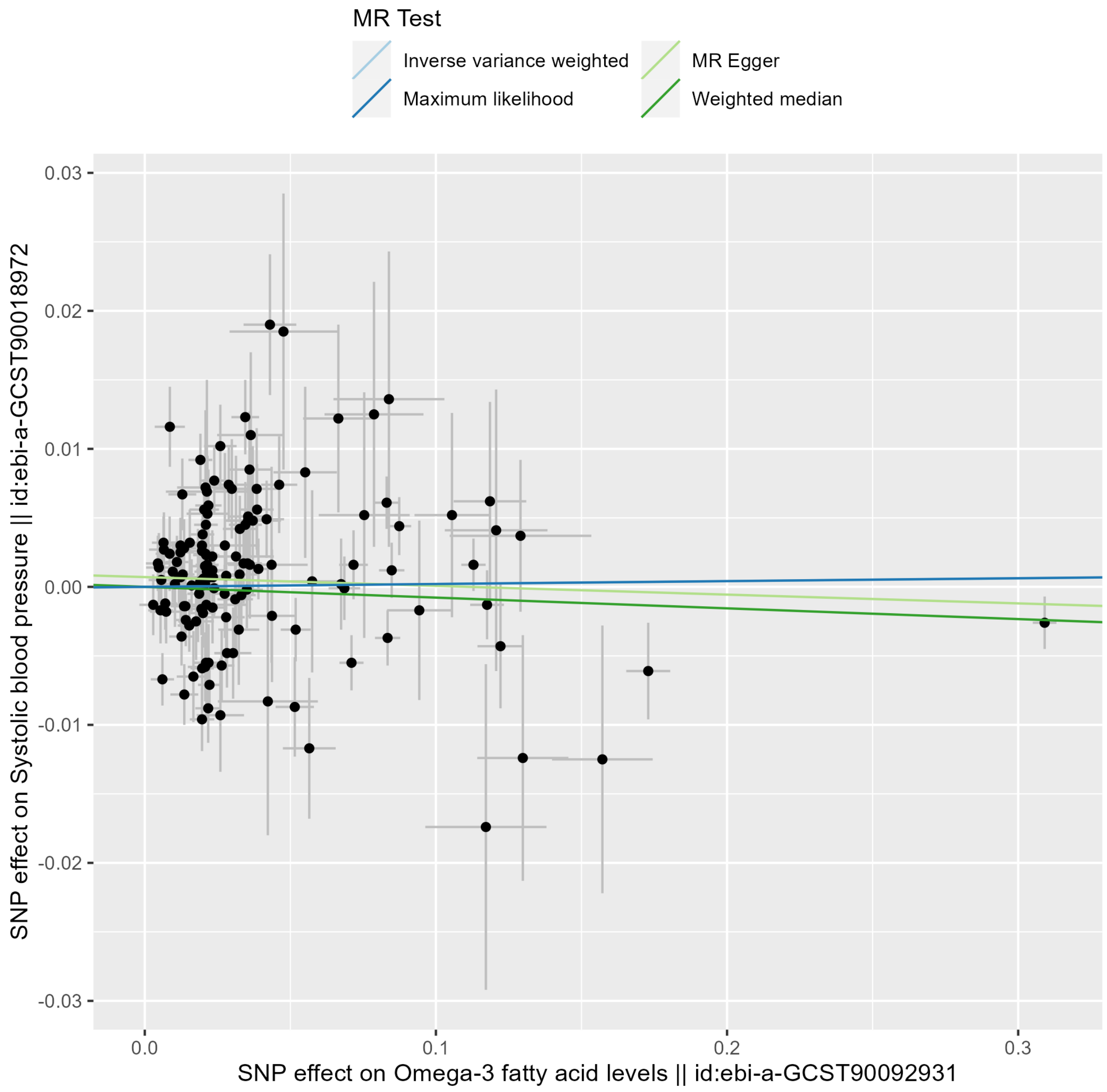

omegas-EH scatter plot

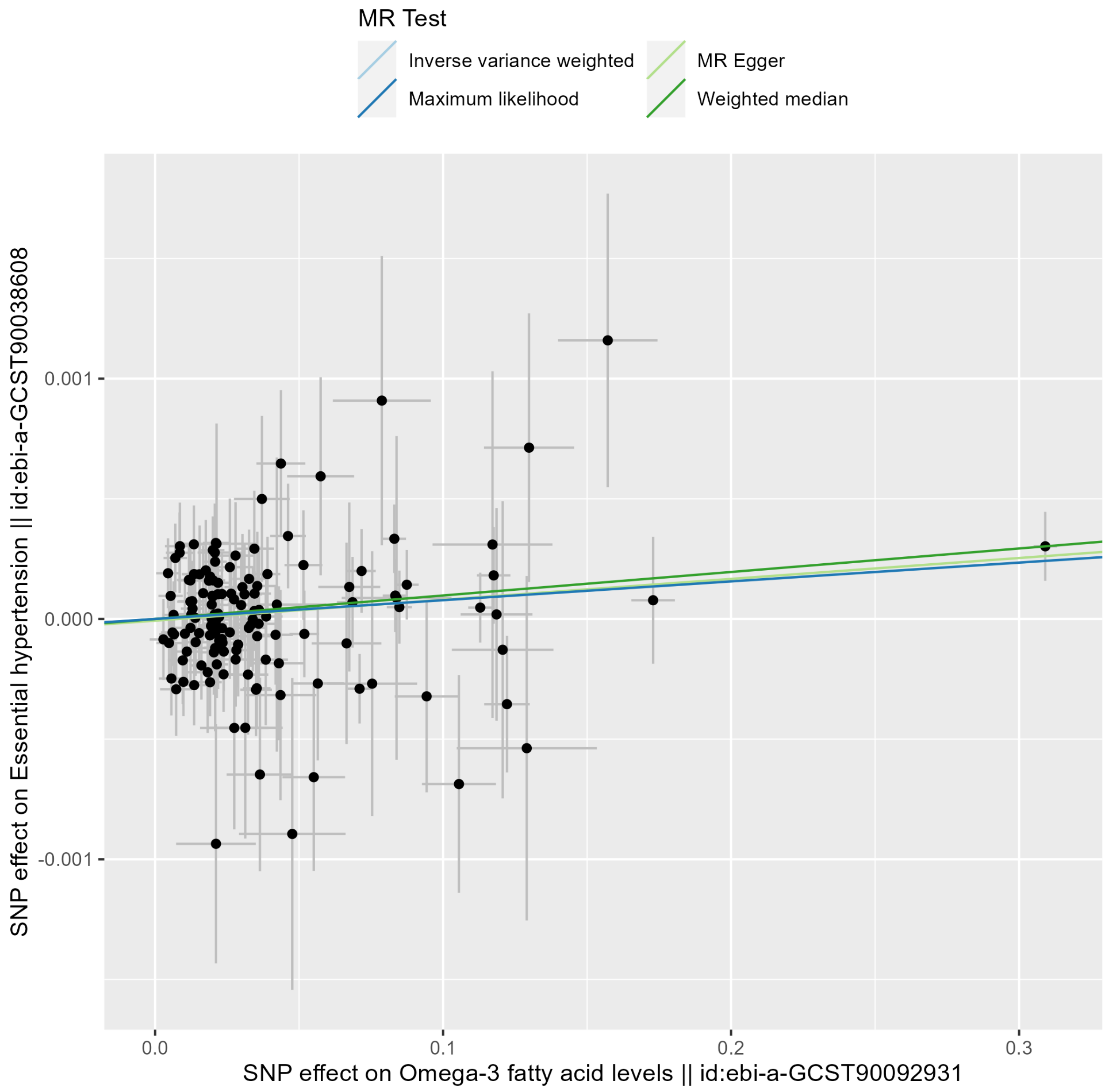

omegas-DBP forest plot

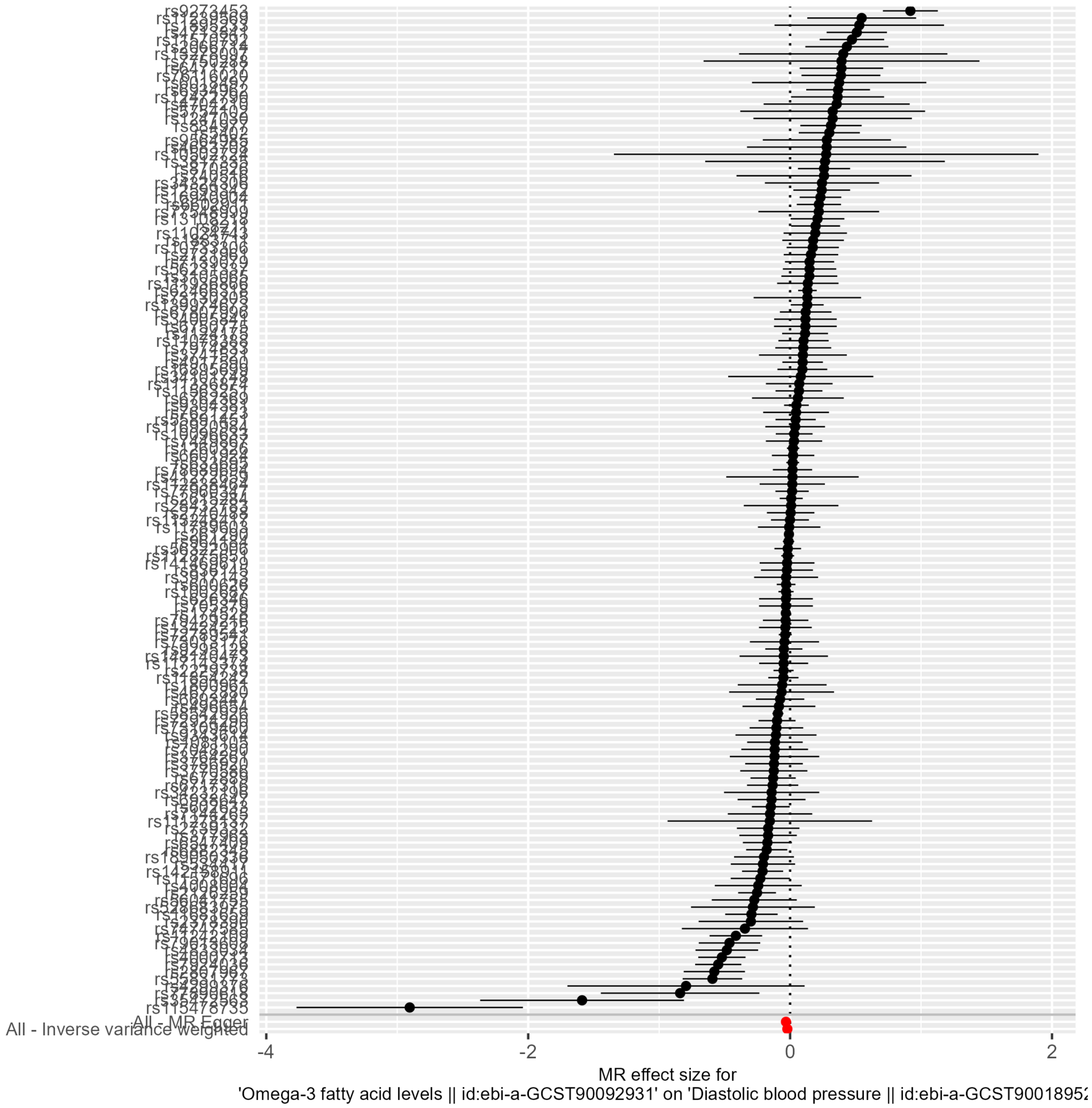

omegas-SBP forest plot

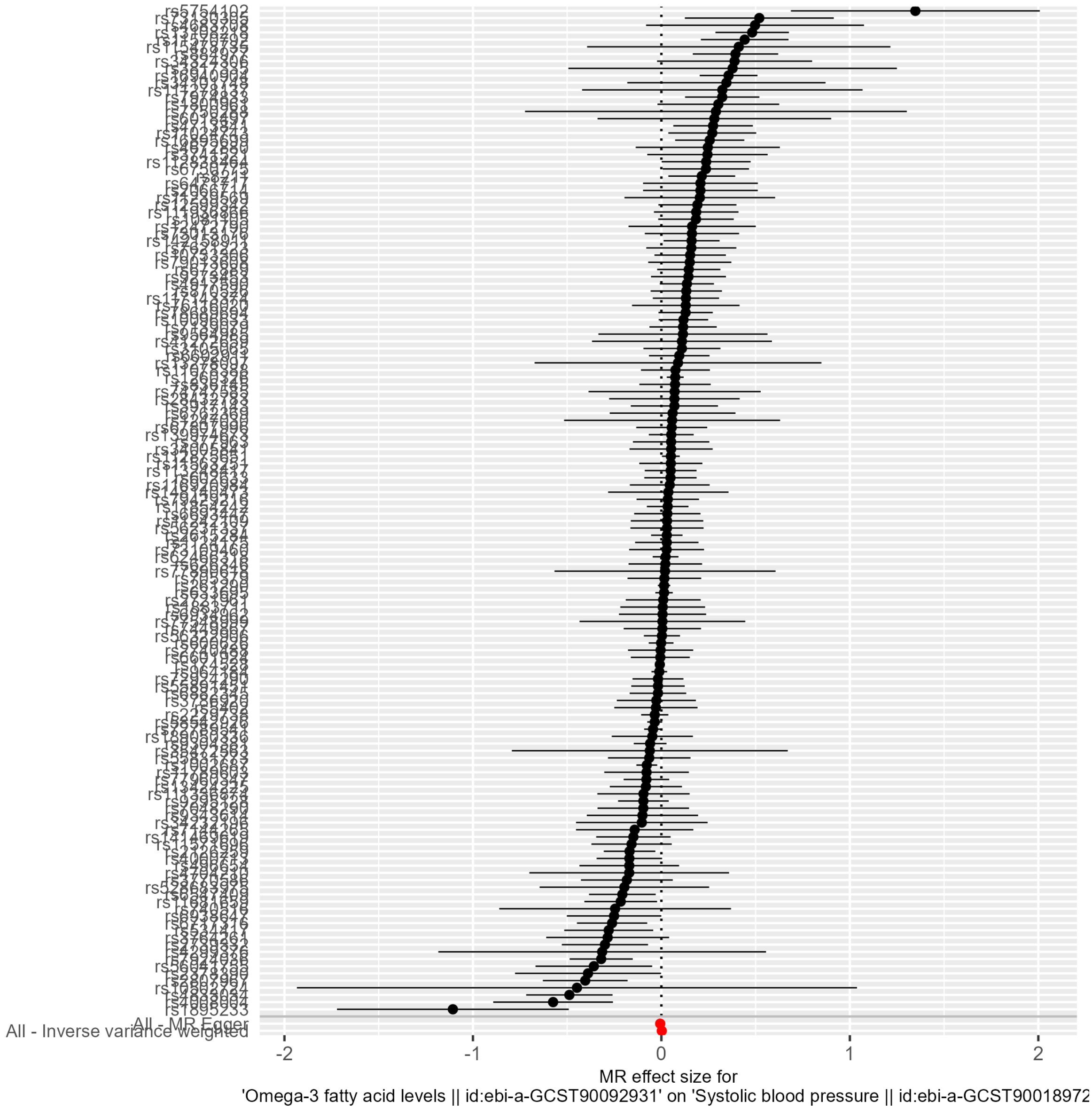

omegas-EH forest plot

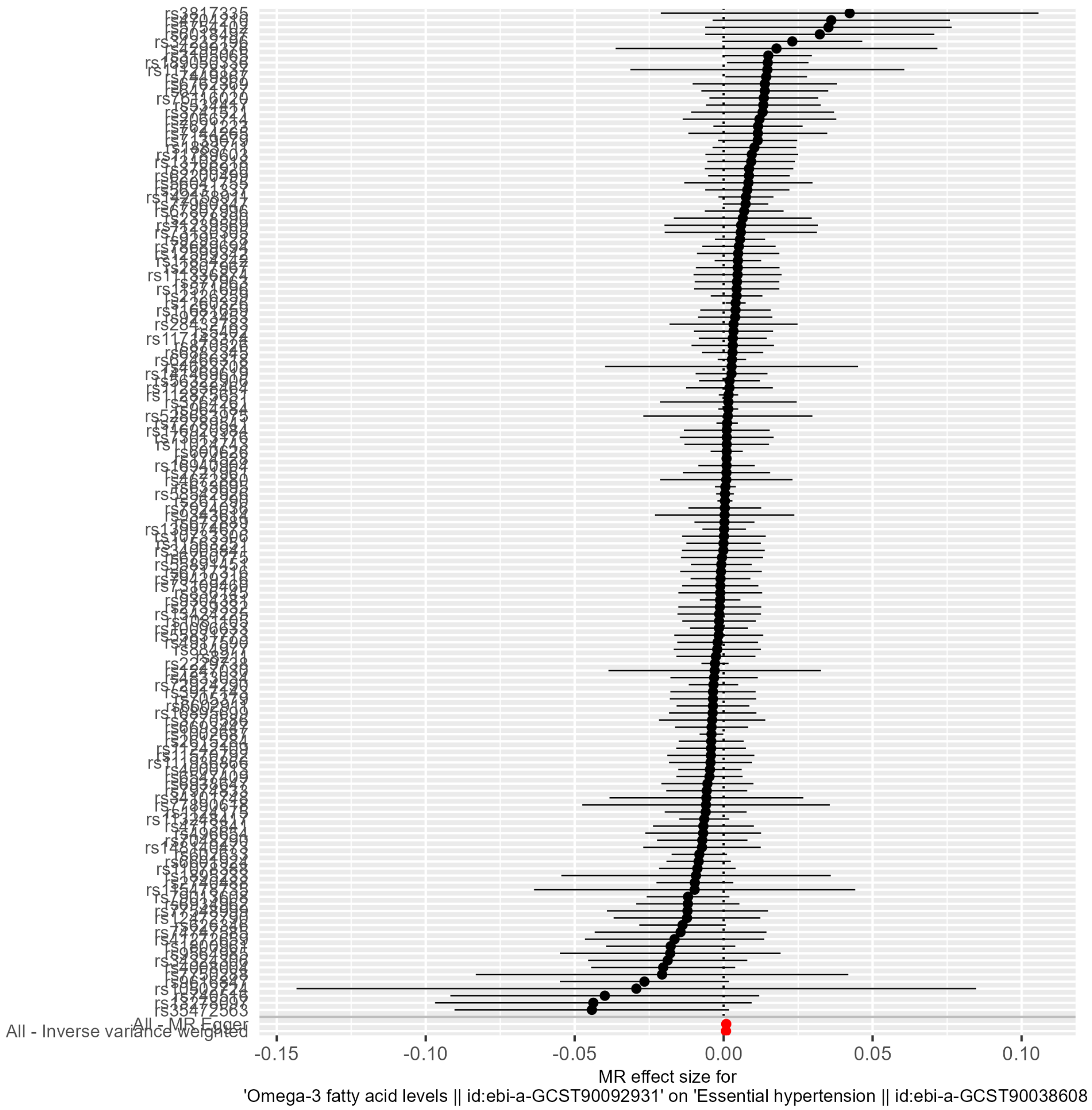

omegas-DBP funnel plot

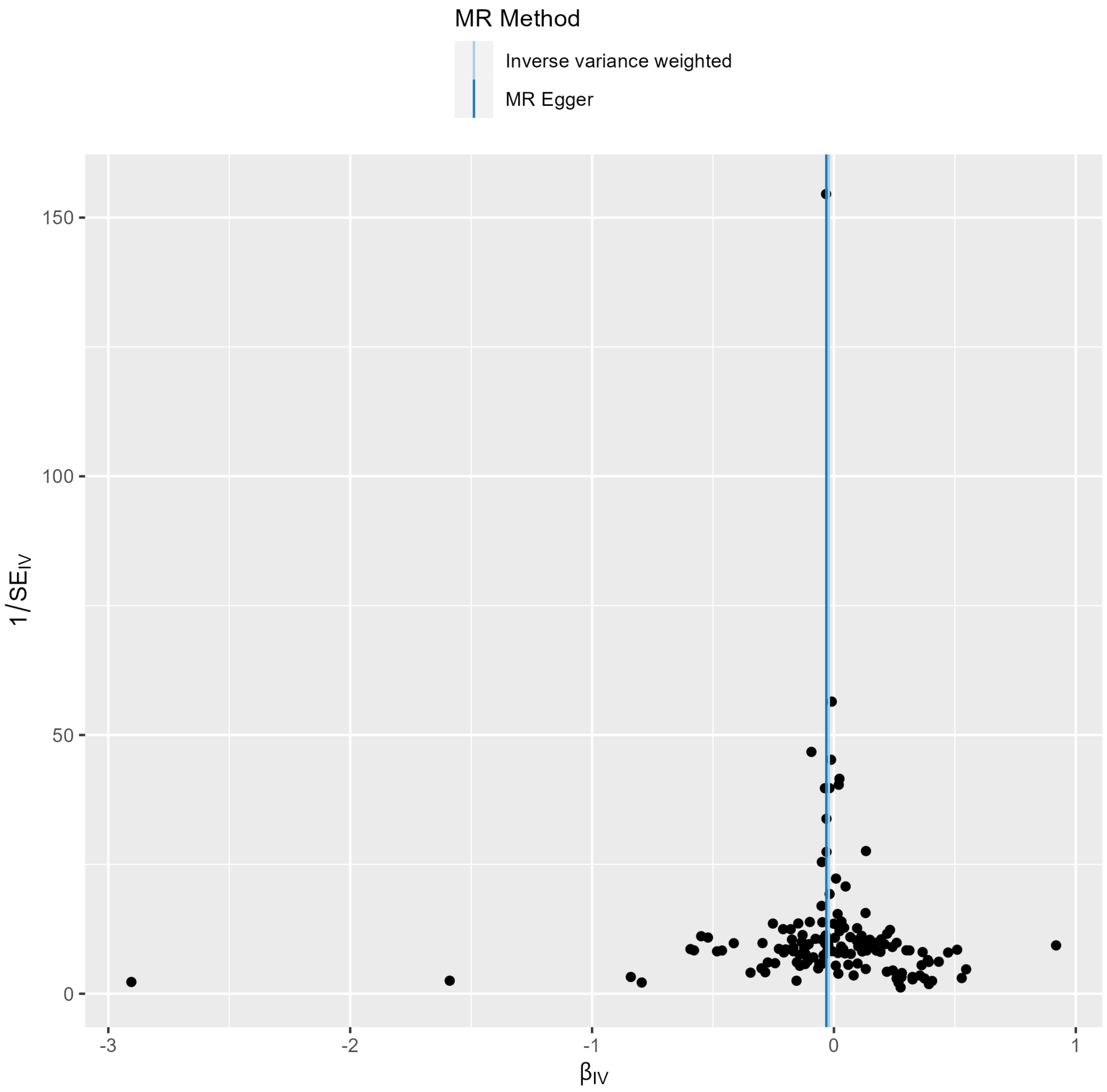

omegas-SBP funnel plot

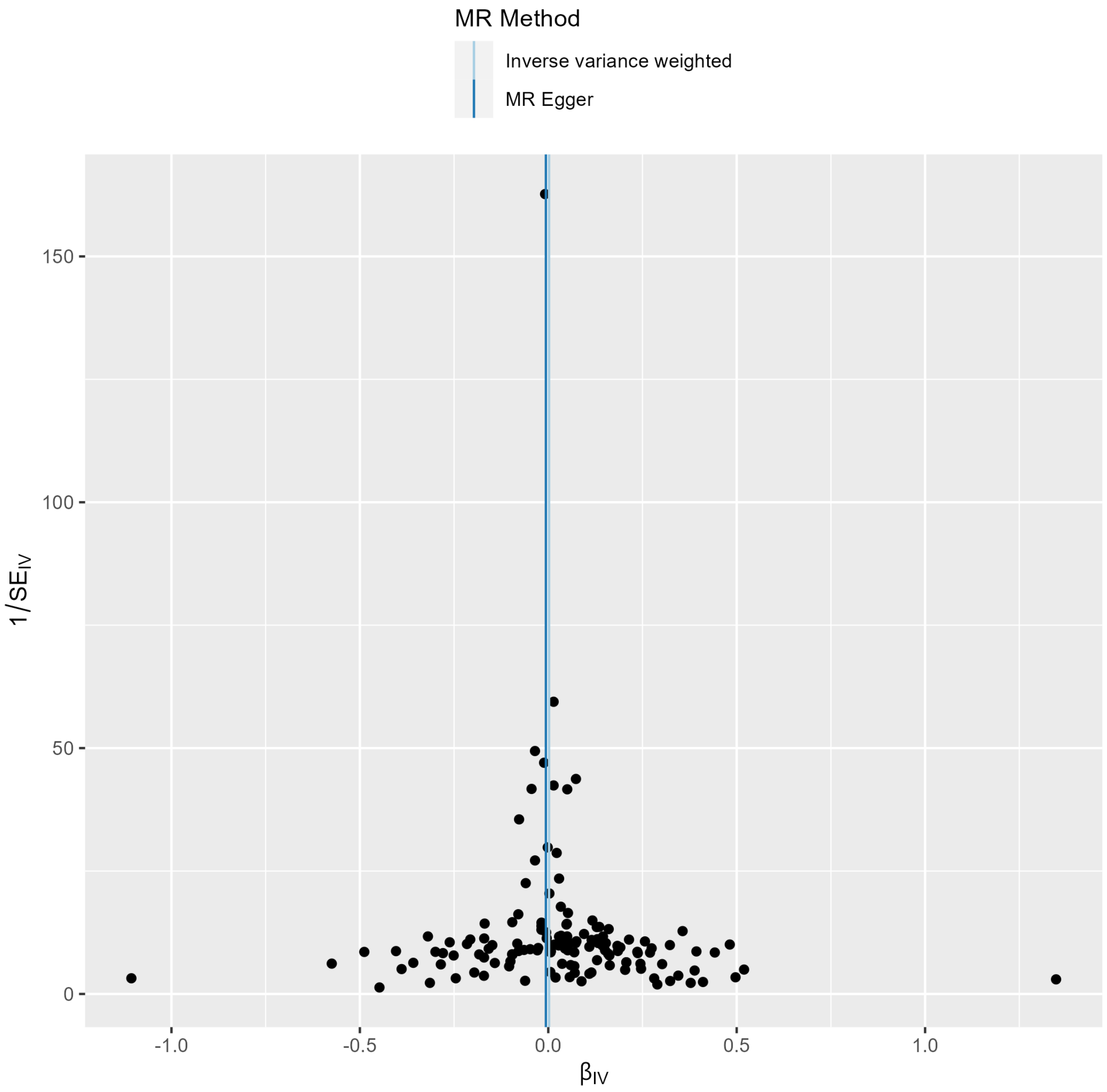

omegas-EH funnel plot

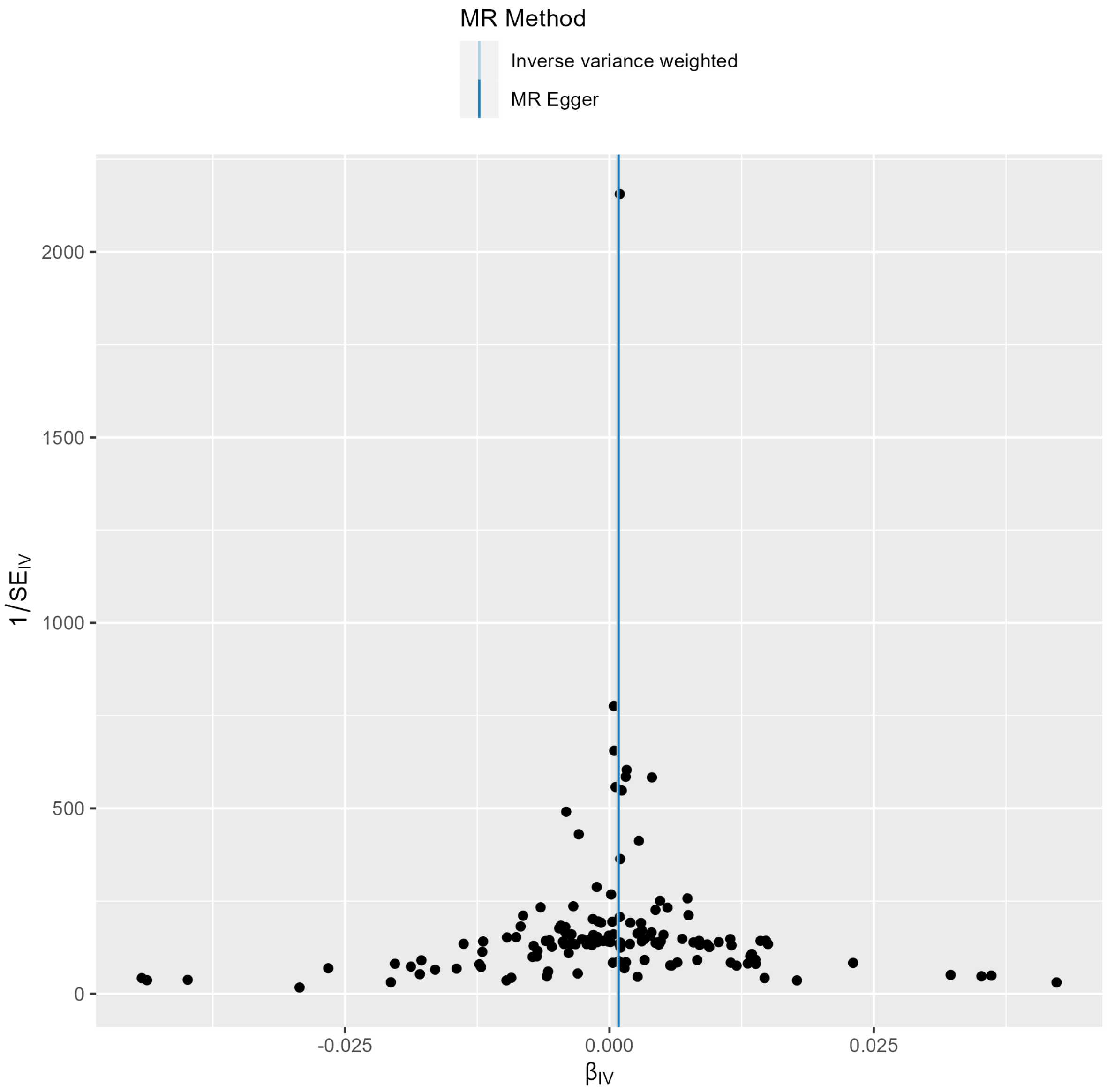

omegas-LAS leave-one-out

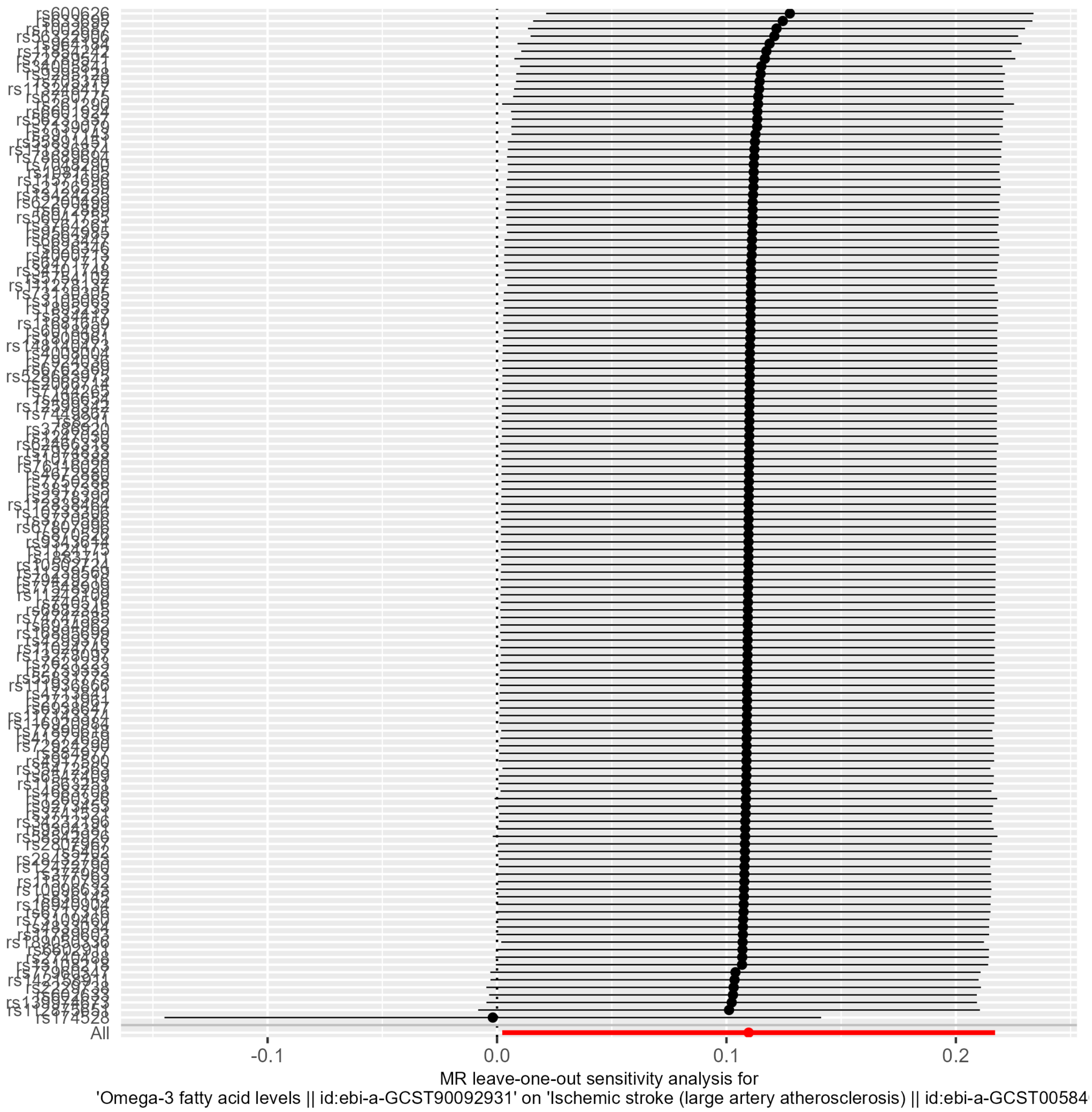

omegas-SVS leave-one-out

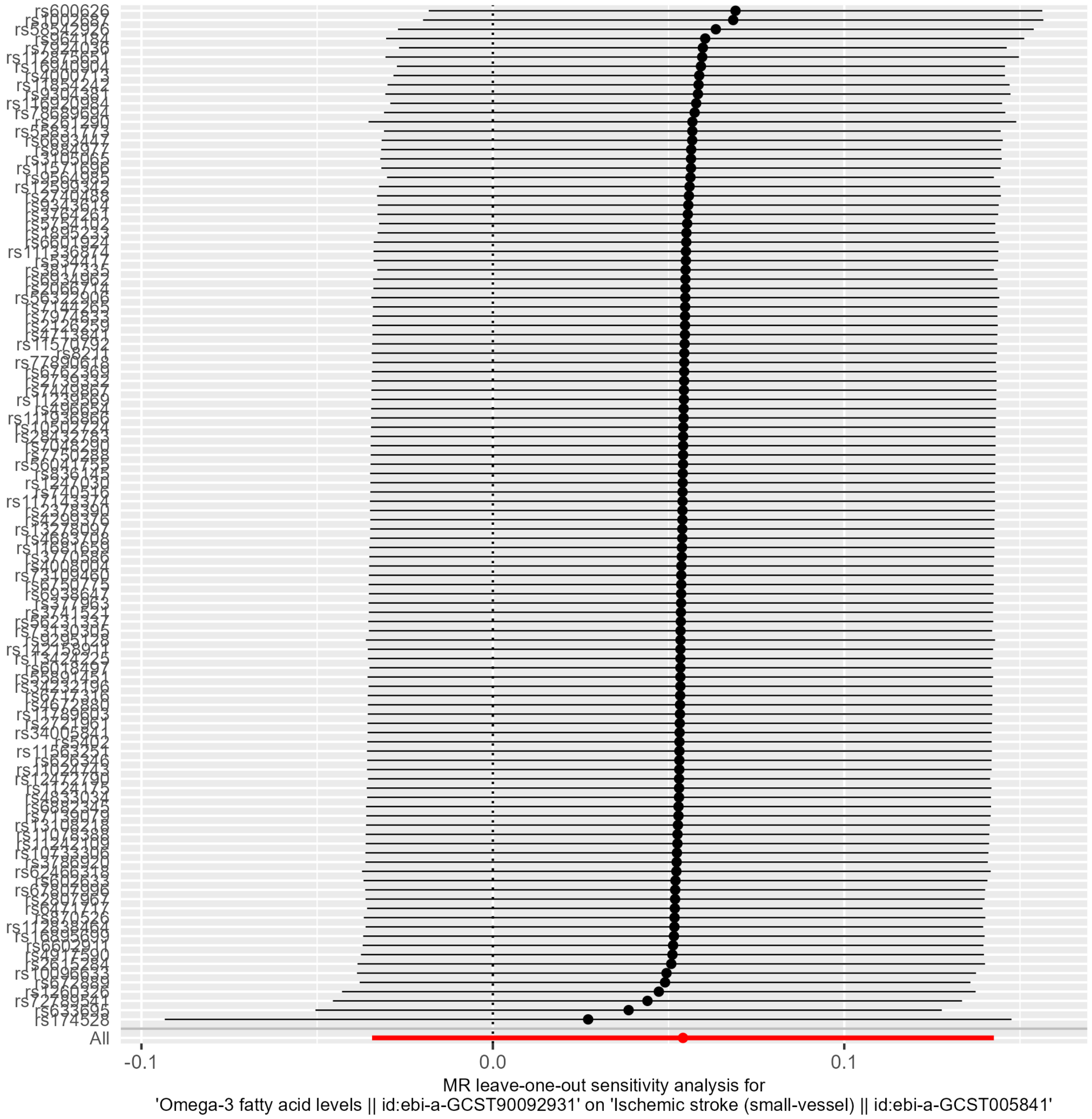

omegas-CES leave-one-out

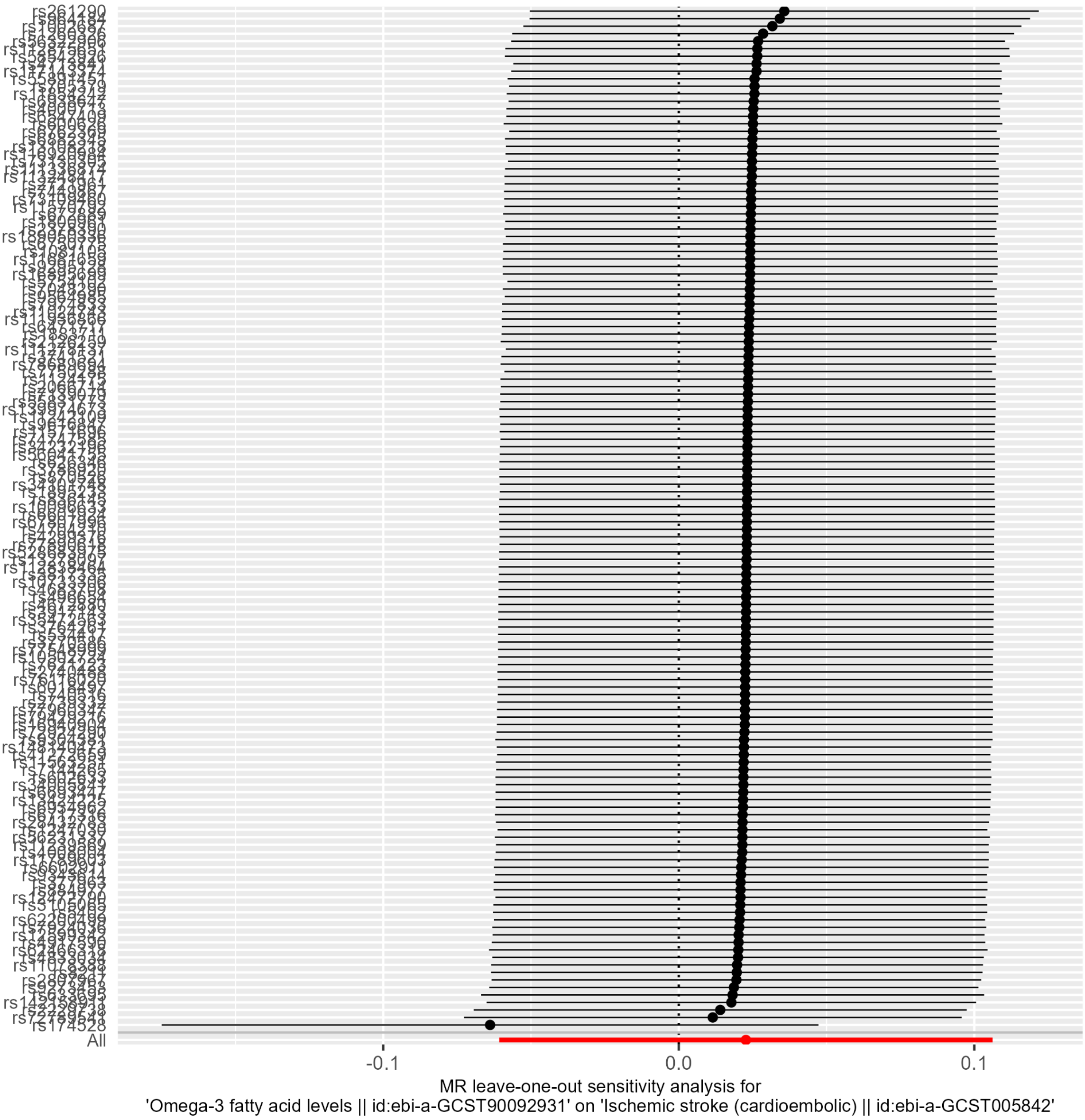

omegas-IS leave-one-out

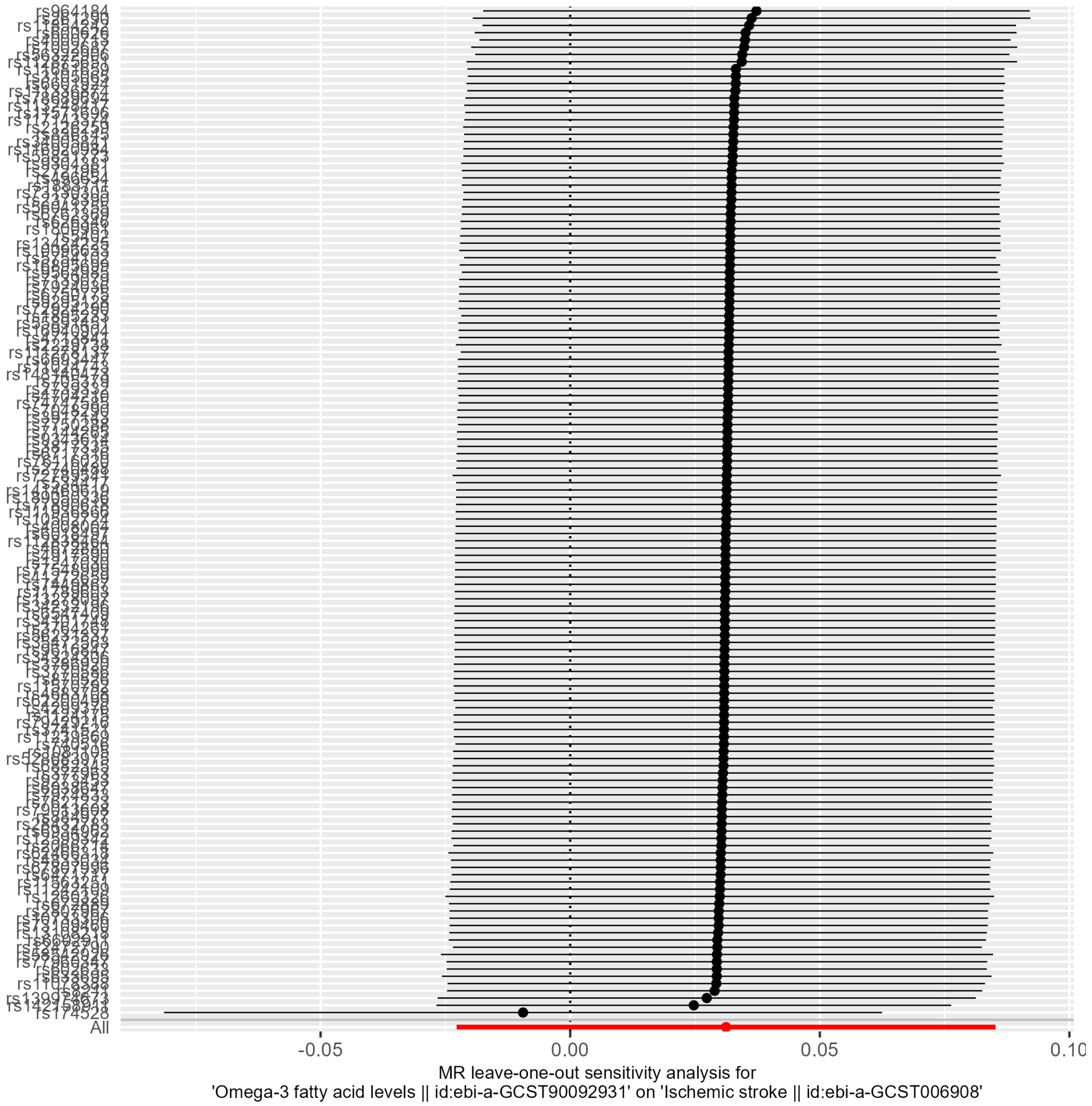

omegas-LS leave-one-out

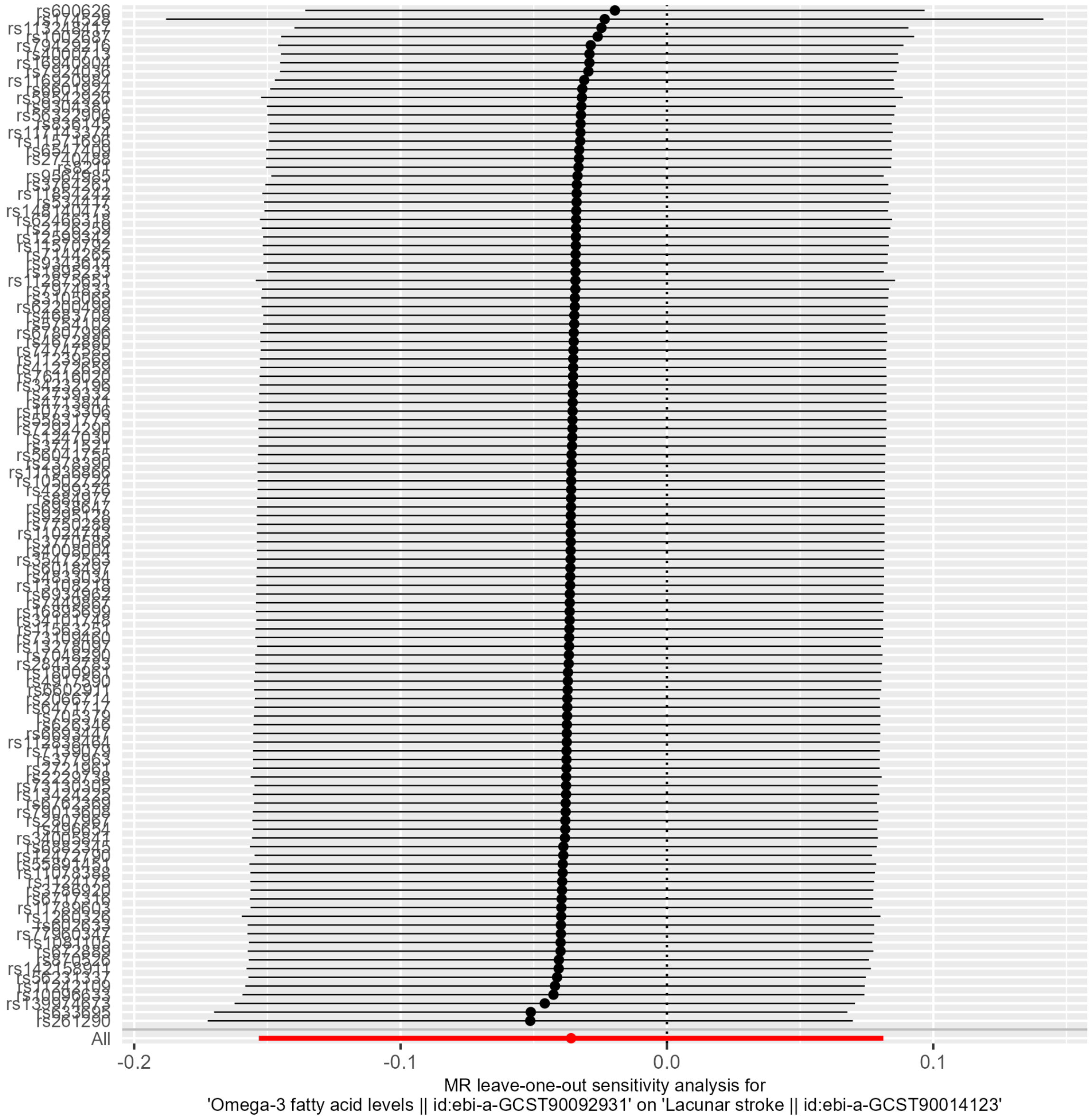

omegas-LAS scatter plot

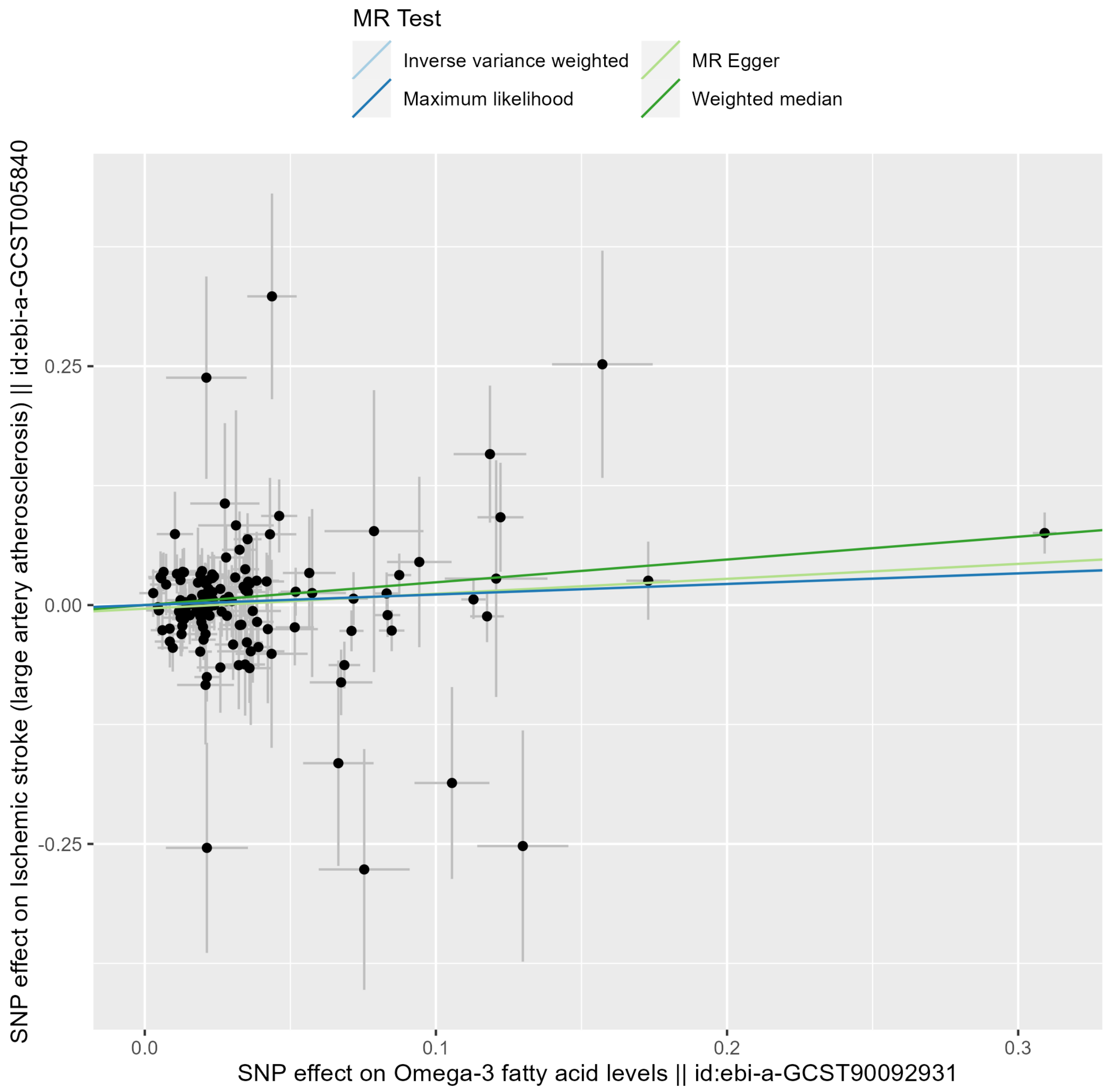

omegas-SVS scatter plot

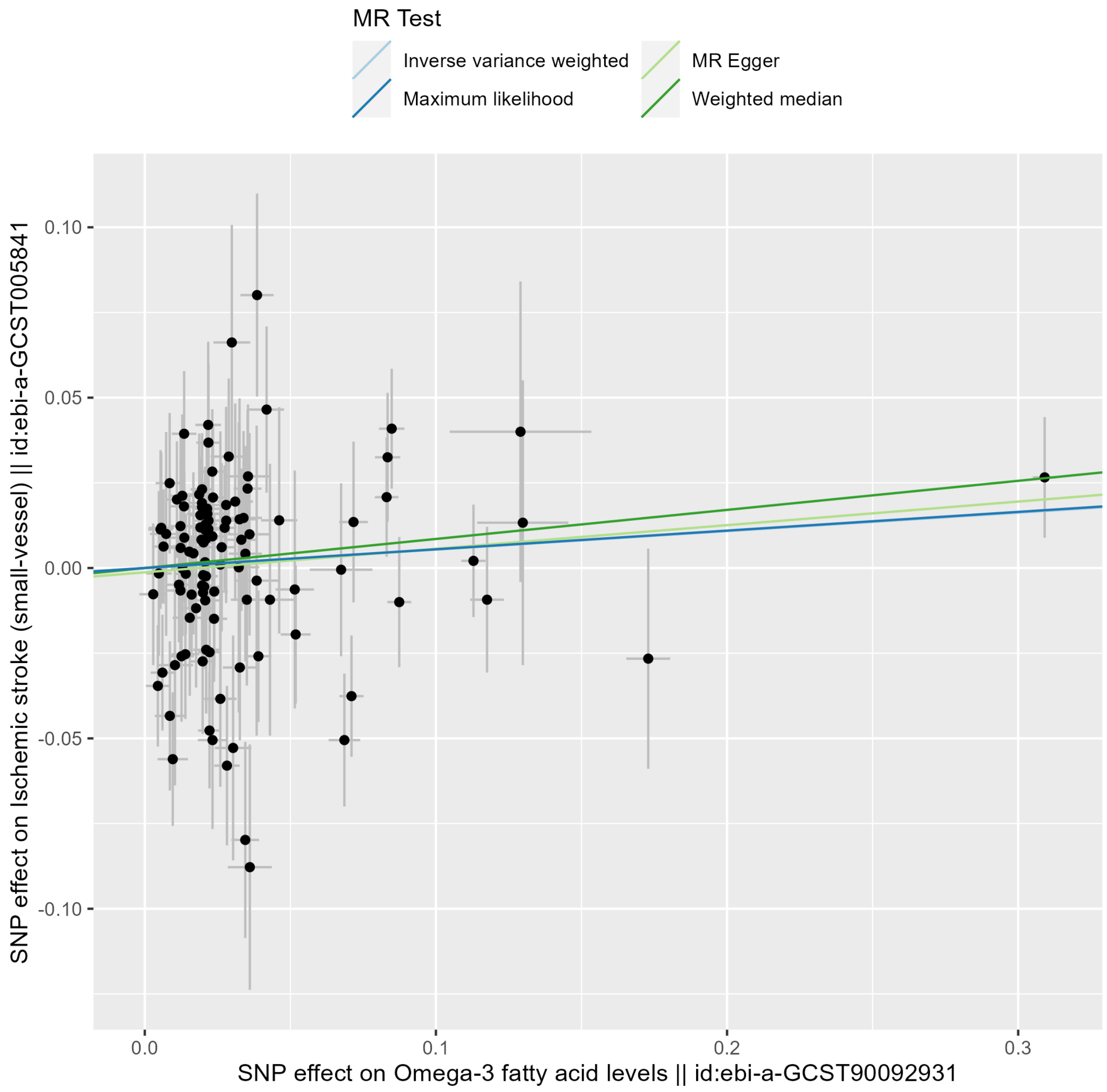

omegas-CES scatter plot

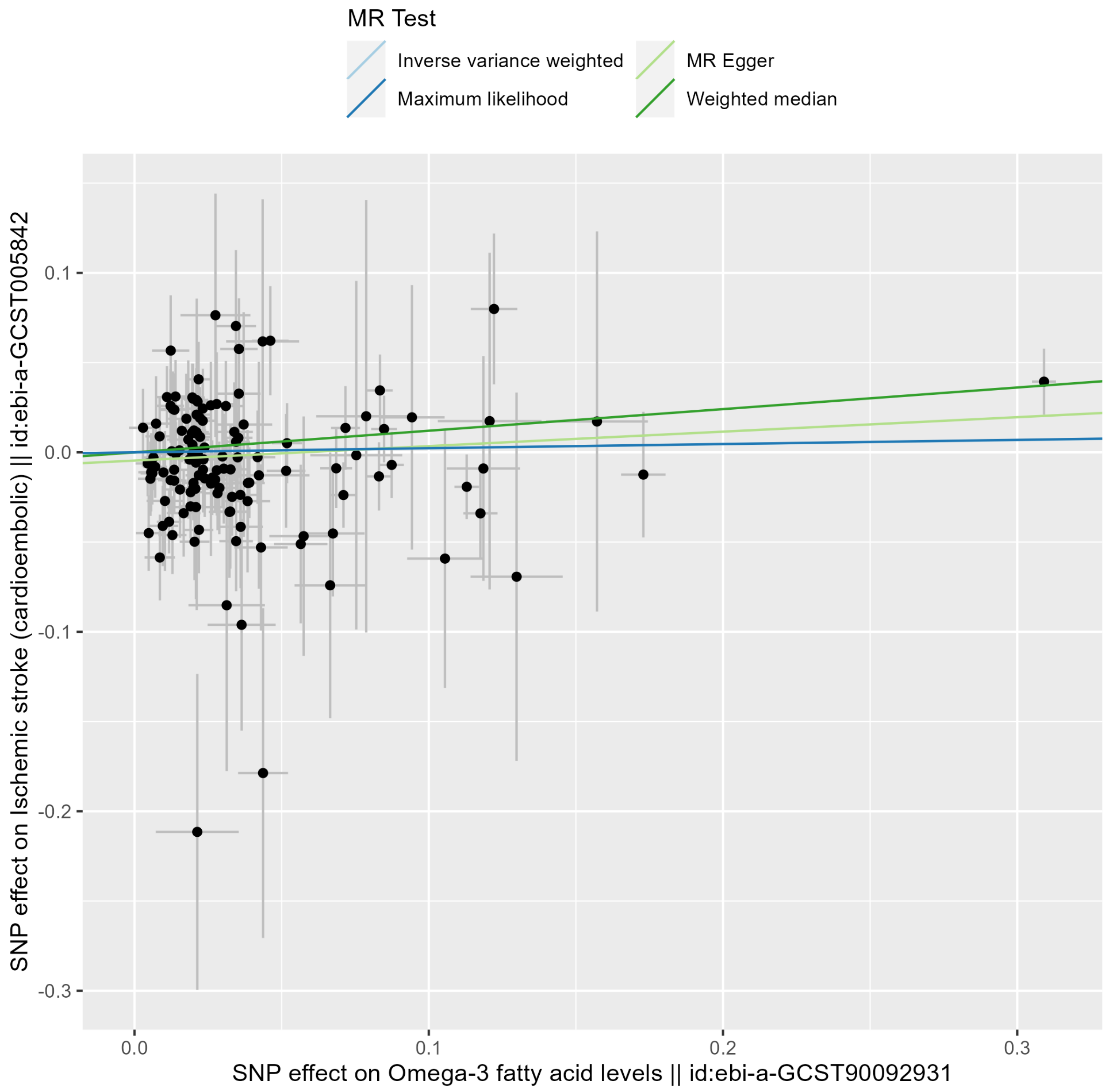

omegas-IS scatter plot

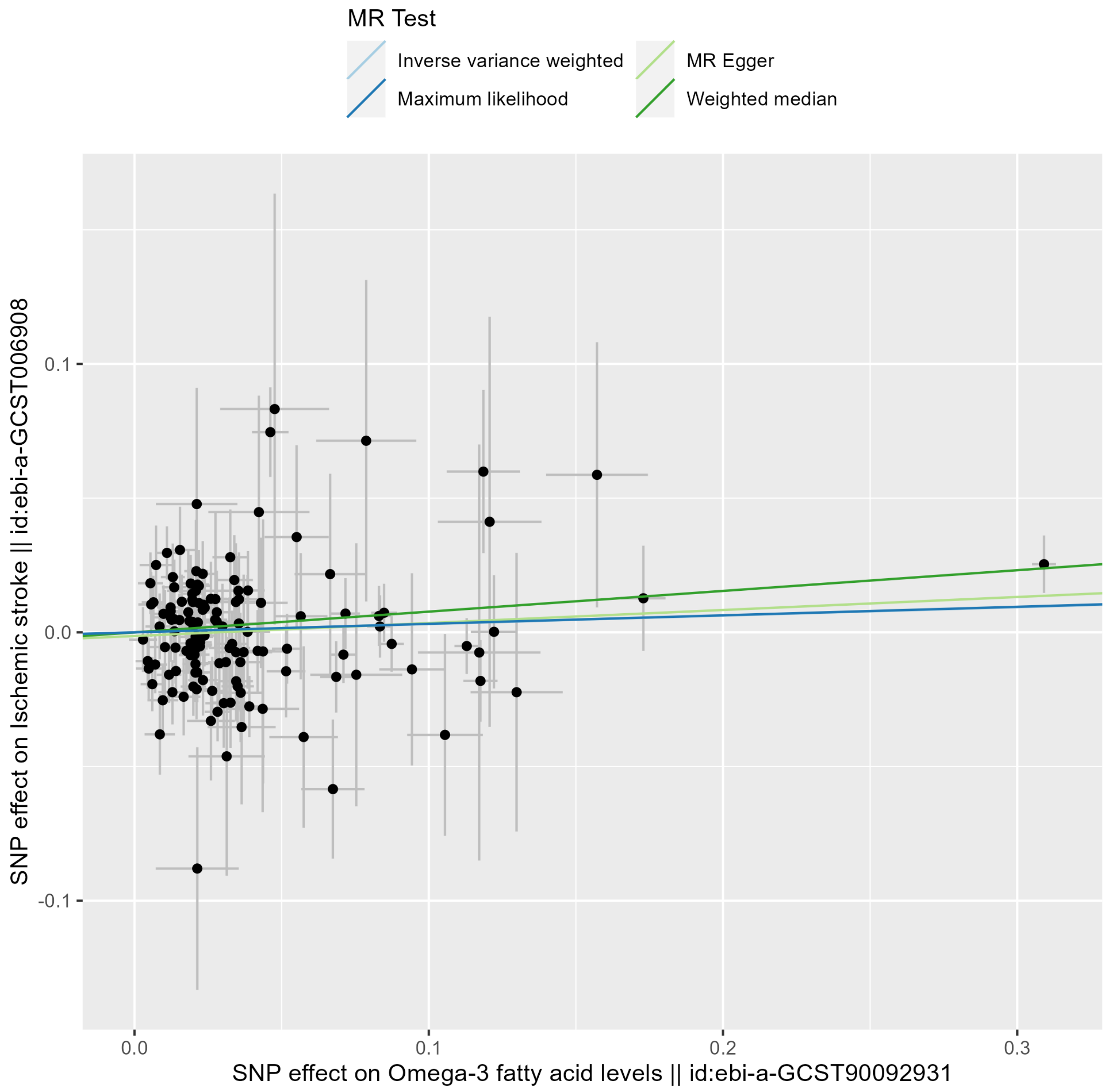

omegas-LS scatter plot

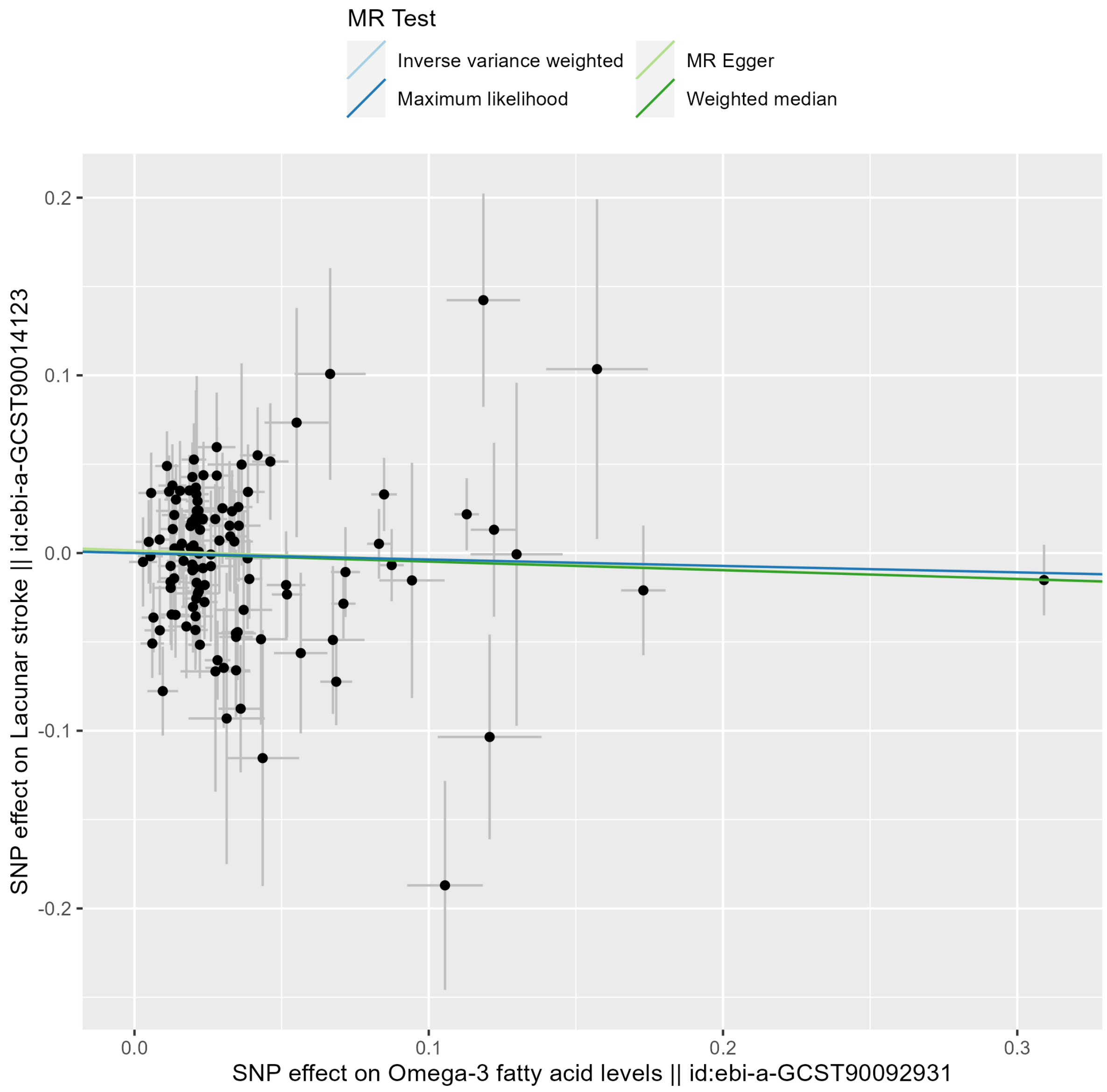

omegas-LAS forest plot

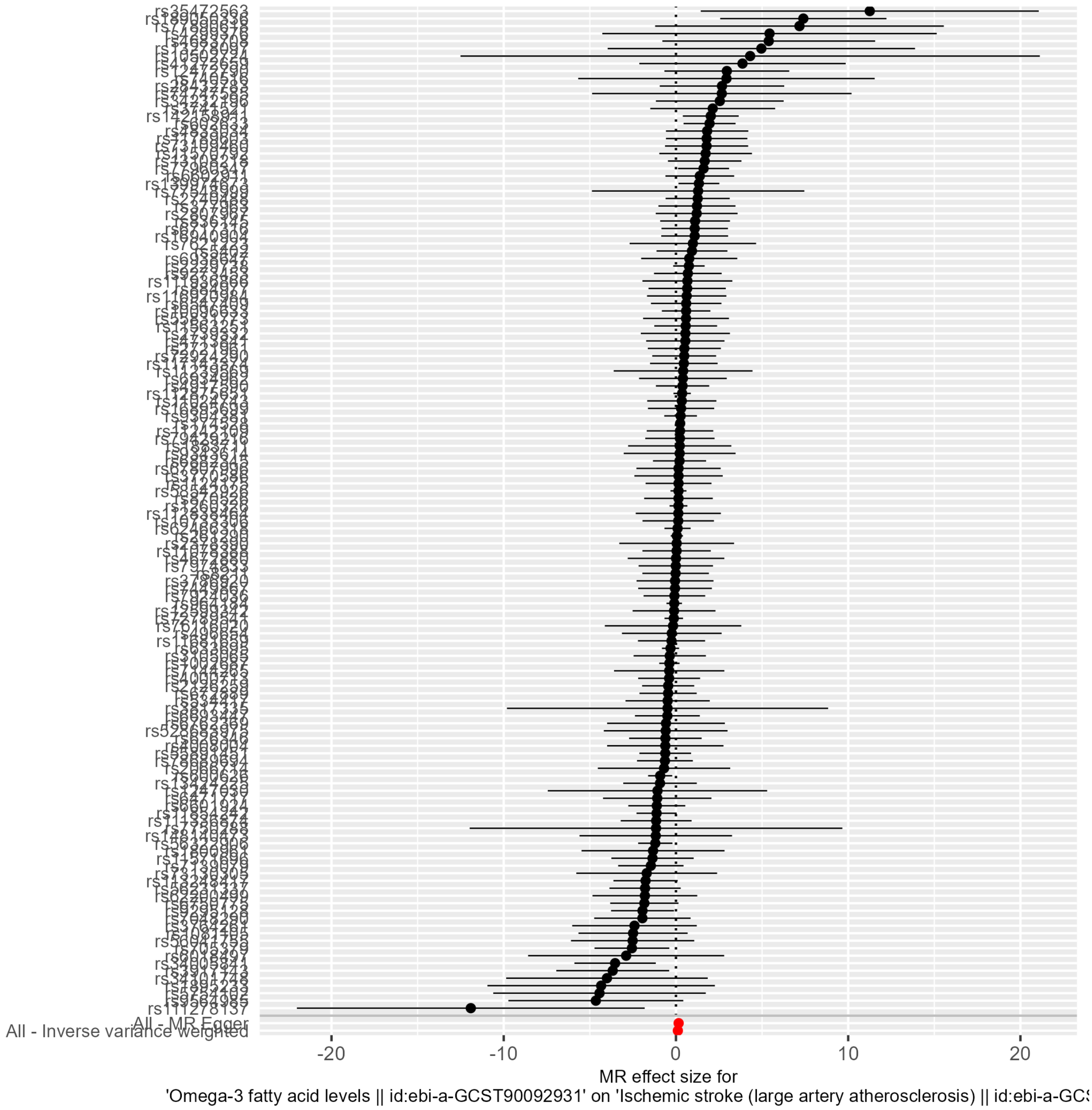

omegas-SVS forest plot

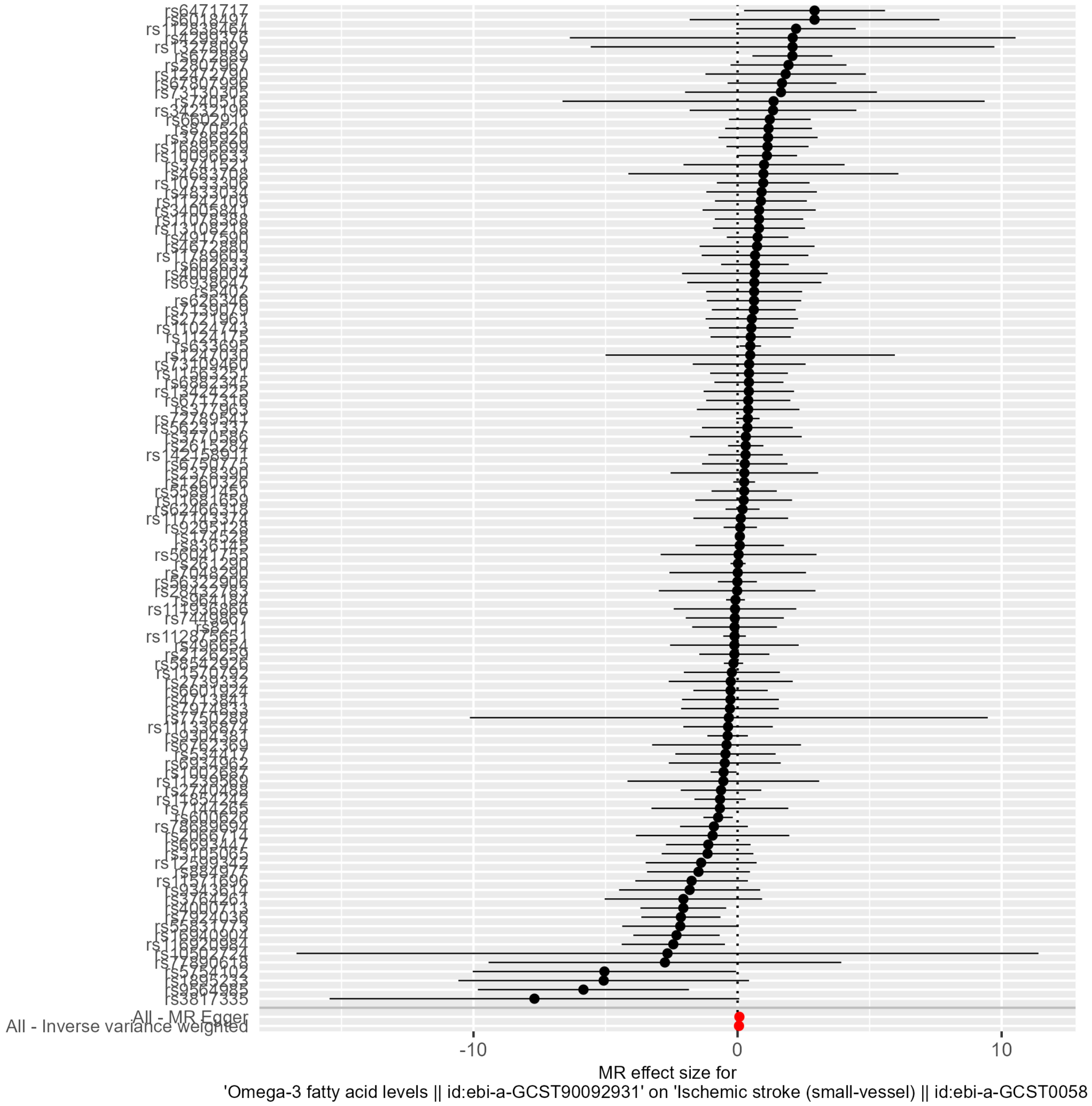

omegas-CES forest plot

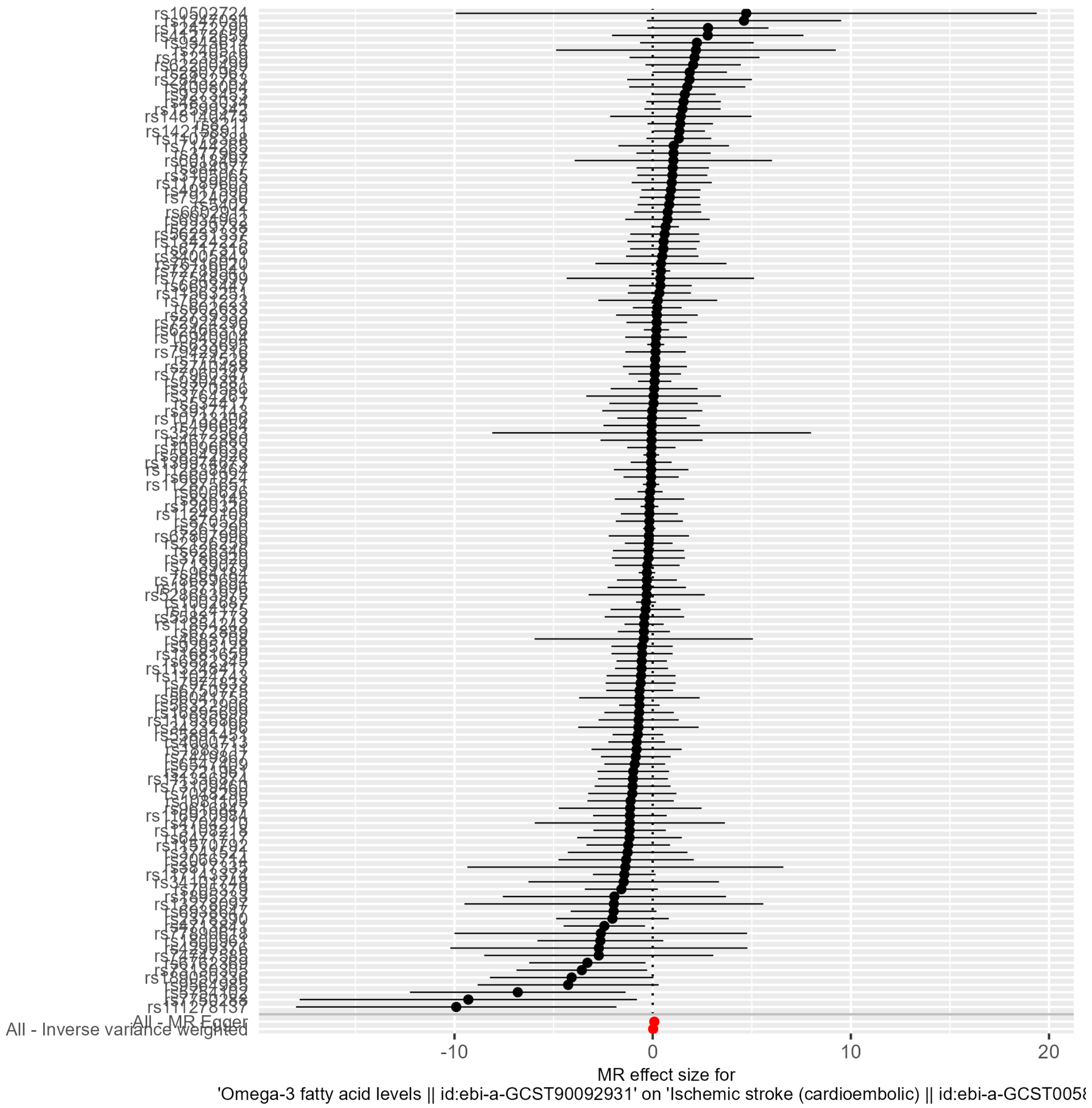

omegas-IS forest plot

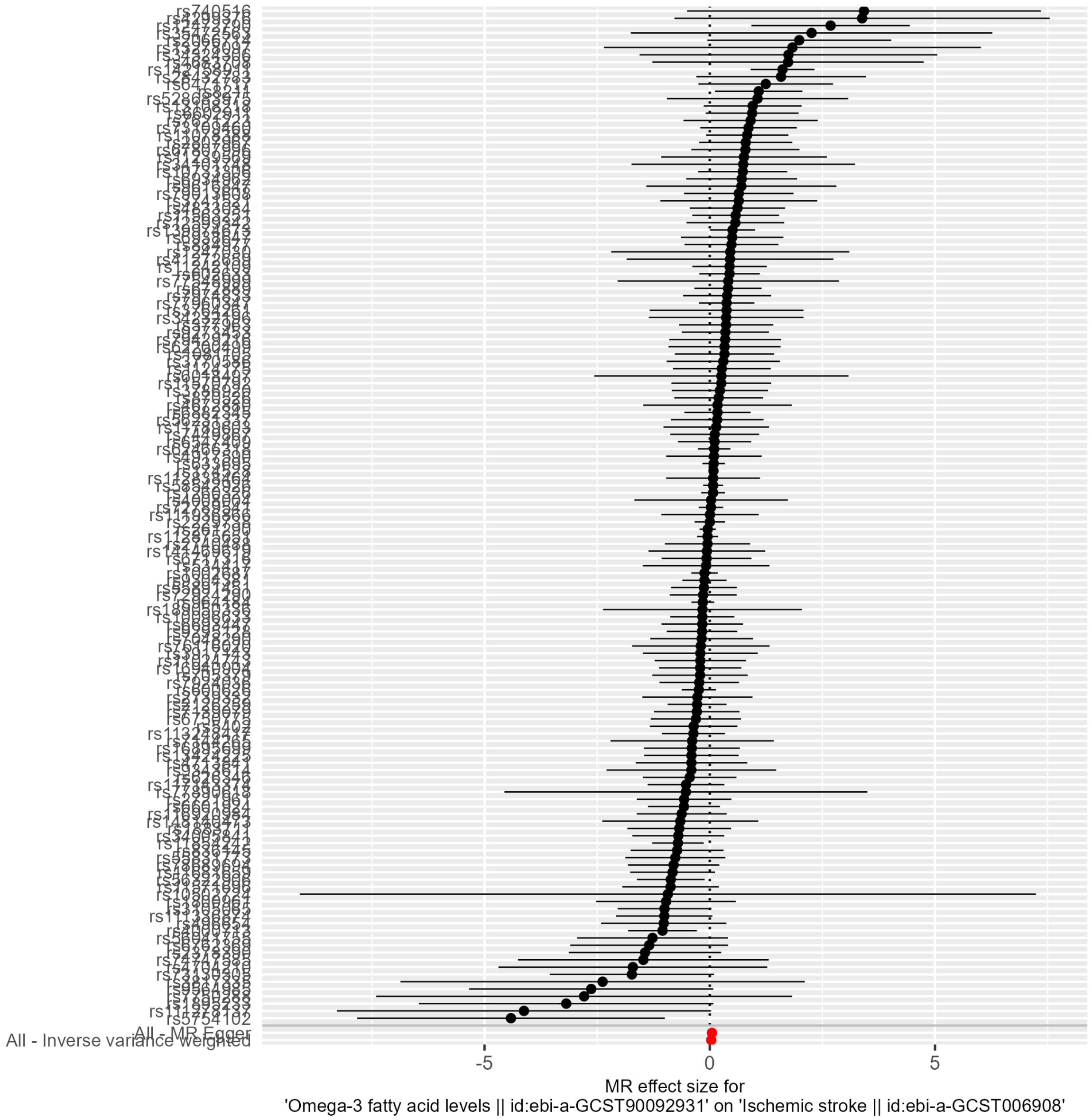

omegas-LS forest plot

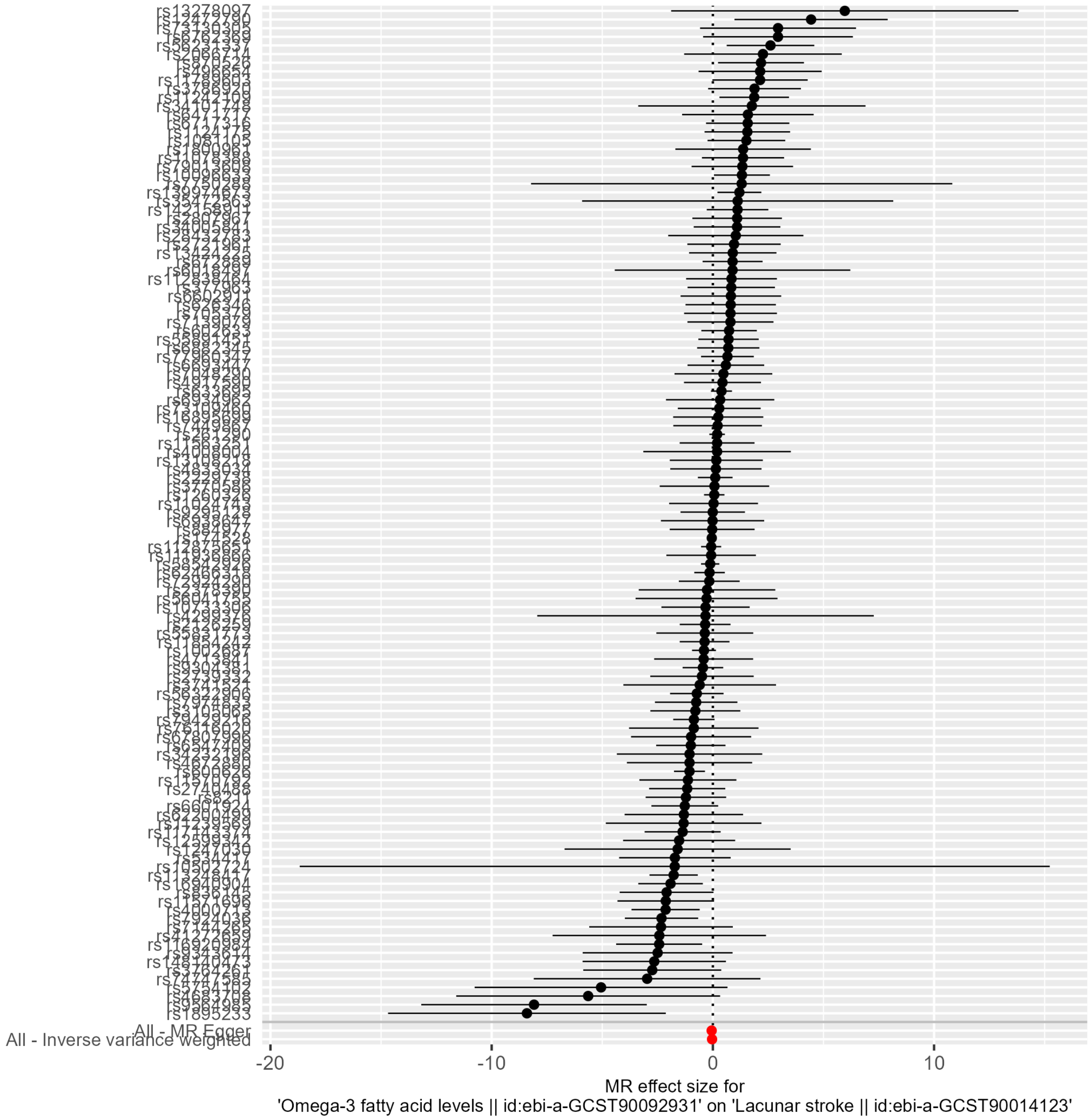

omegas-LAS funnel plot

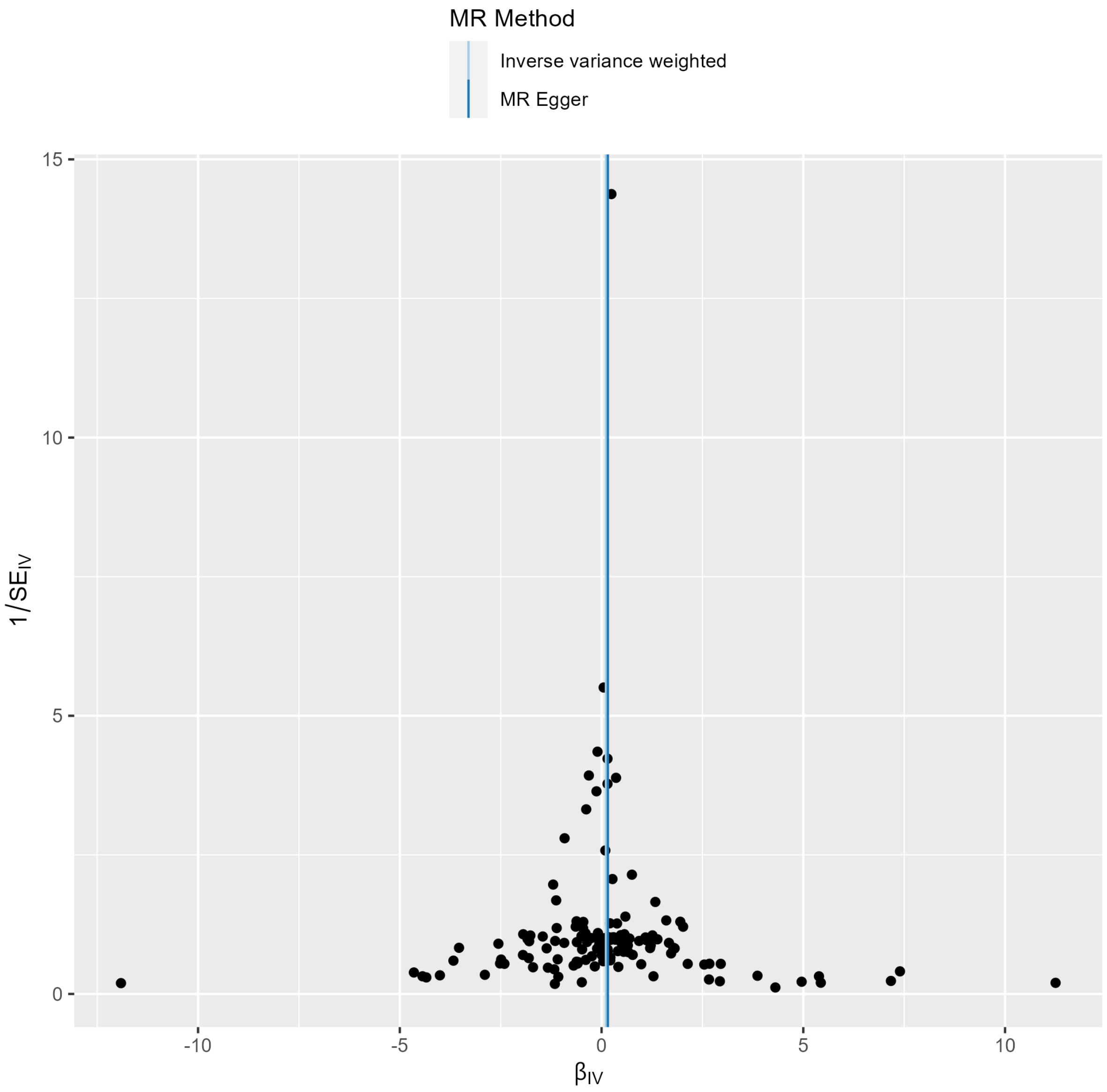

omegas-SVS funnel plot

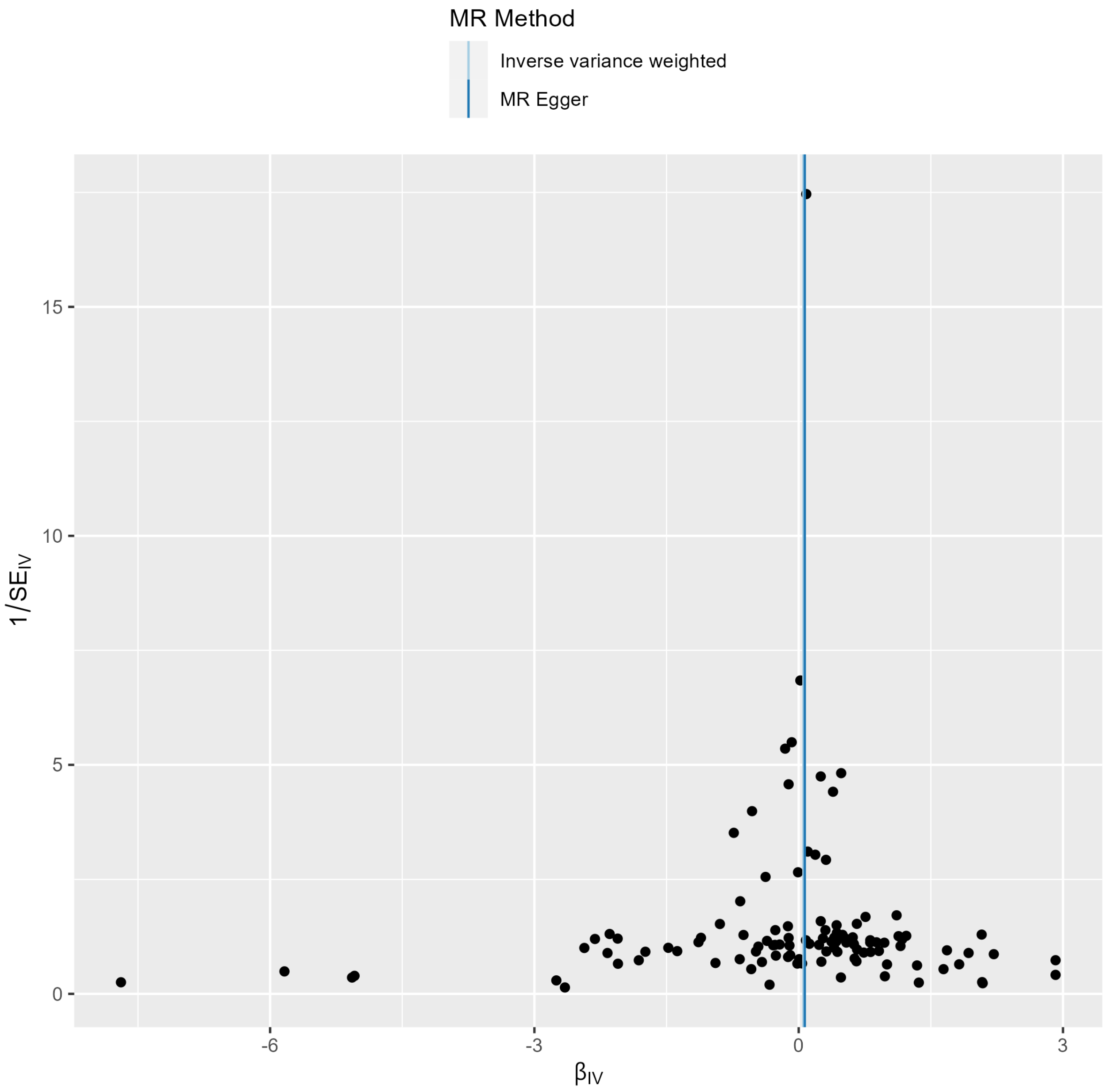

omegas-CES funnel plot

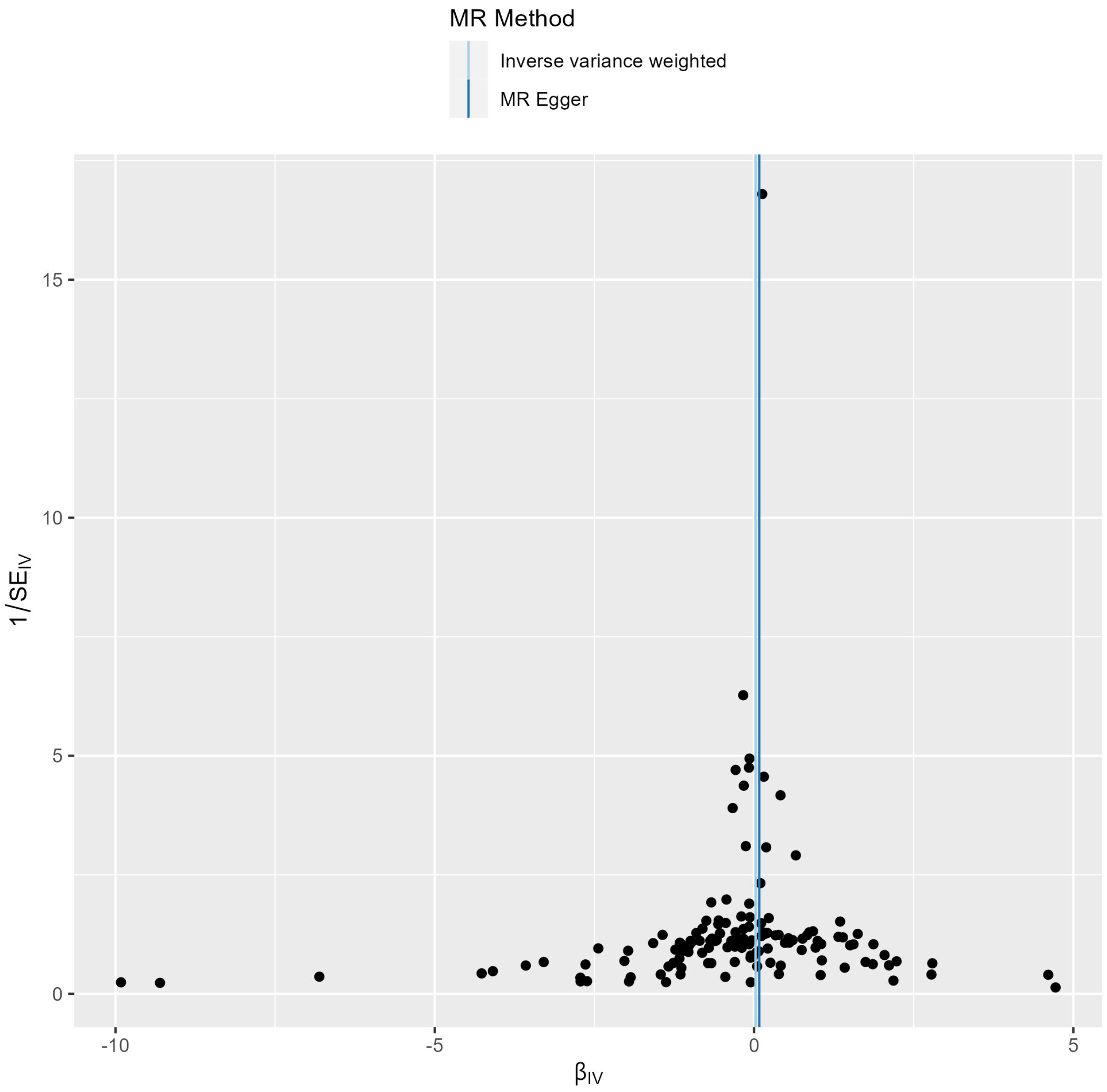

omegas-IS funnel plot

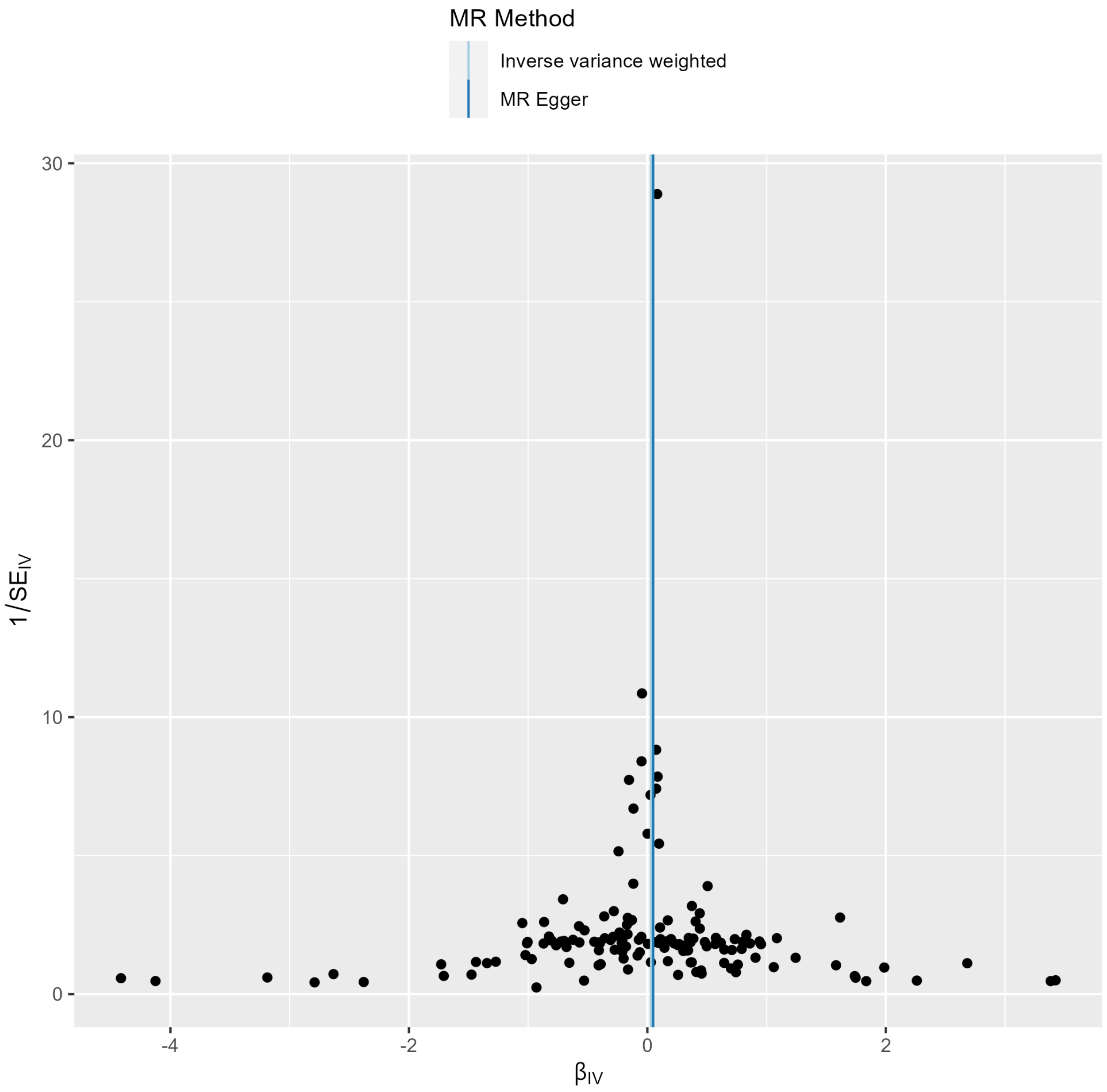

omegas-LS funnel plot

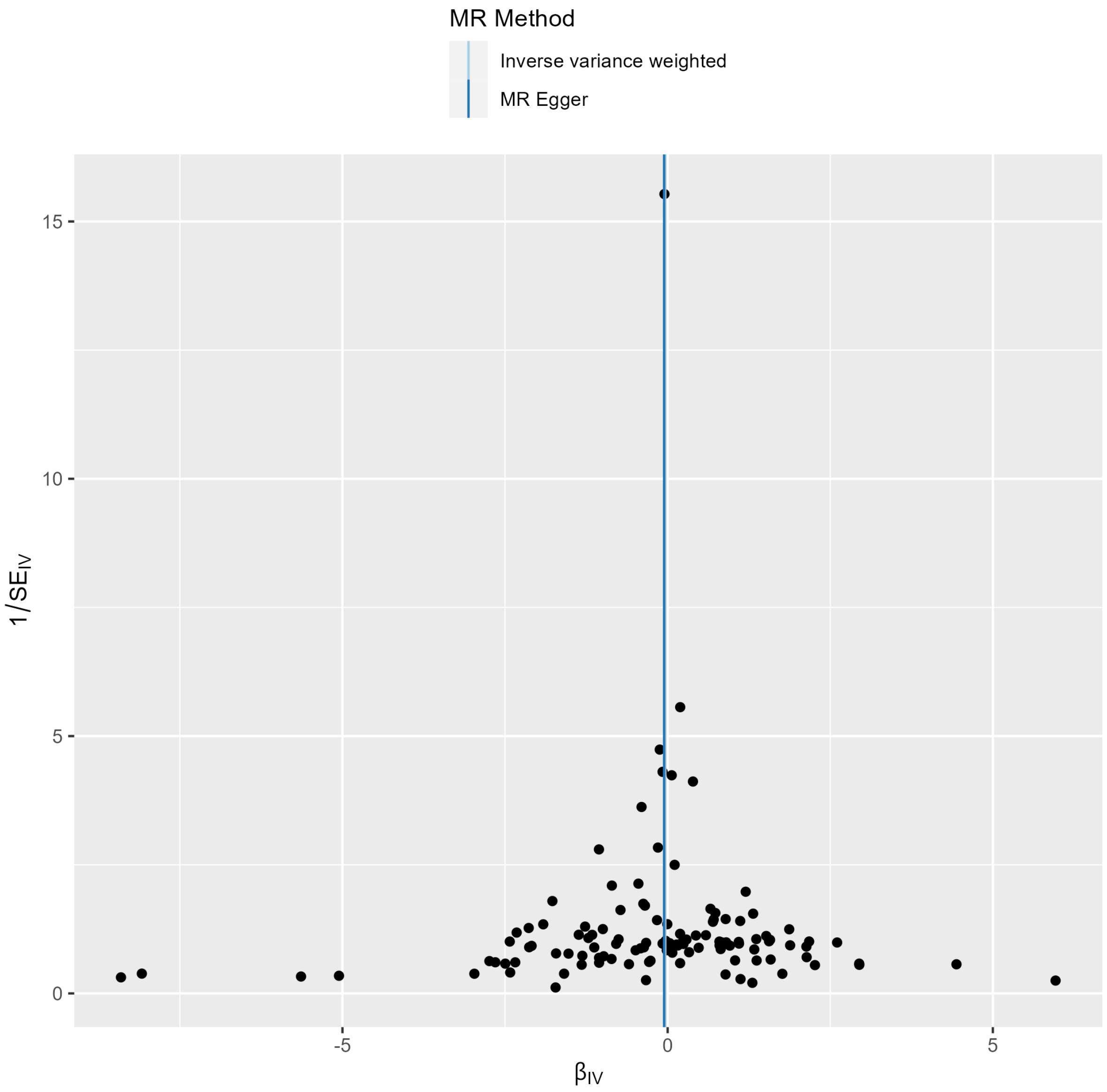
MR Method
Inverse variance weighted
MR Egger
15
10
5
0
1/SE_IV
-5
0
5
β_IV
